# Zolbetuximab plus CAPOX in CLDN18.2-positive gastric or gastroesophageal junction adenocarcinoma: the randomized, phase 3 GLOW trial

**DOI:** 10.1038/s41591-023-02465-7

**Published:** 2023-07-31

**Authors:** Manish A. Shah, Kohei Shitara, Jaffer A. Ajani, Yung-Jue Bang, Peter Enzinger, David Ilson, Florian Lordick, Eric Van Cutsem, Javier Gallego Plazas, Jing Huang, Lin Shen, Sang Cheul Oh, Patrapim Sunpaweravong, Hwoei Fen Soo Hoo, Haci Mehmet Turk, Mok Oh, Jung Wook Park, Diarmuid Moran, Pranob Bhattacharya, Ahsan Arozullah, Rui-Hua Xu

**Affiliations:** 1 Weill Cornell Medical College, New York City, NY USA; 2 Department of Gastrointestinal Oncology, National Cancer Center Hospital East, Kashiwa City, Japan; 3 The University of Texas MD Anderson Cancer Center, Houston, TX USA; 4 Department of Internal Medicine, Seoul National University College of Medicine, Seoul, Republic of Korea; 5 Center for Esophageal and Gastric Cancer, Dana-Farber Cancer Institute, Boston, MA USA; 6 Memorial Sloan Kettering Cancer Center, New York City, NY USA; 7 Department of Medicine and University Cancer Center Leipzig, University of Leipzig Medical Center, Leipzig, Germany; 8 Digestive Oncology, University Hospitals Gasthuisberg, Leuven and KULeuven, Leuven, Belgium; 9 Department of Medical Oncology, Hospital General Universitario de Elche, Elche, Spain; 10 Department of Medical Oncology, National Cancer Center / National Clinical Research Center for Cancer / Cancer Hospital, Chinese Academy of Medical Sciences & Peking Union Medical College, Beijing, China; 11 Department of Gastrointestinal Oncology, Key Laboratory of Carcinogenesis and Translational Research (Ministry of Education/Beijing), Peking University Cancer Hospital and Institute, Beijing, China; 12 Department of Internal Medicine, Korea University Guro Hospital, Seoul, Republic of Korea; 13 Department of Internal Medicine, Faculty of Medicine, Prince of Songkla University, Songkhla, Thailand; 14 Department of Oncology and Radiotherapy, Penang Hospital, Penang, Malaysia; 15 Department of Medical Oncology, Faculty of Medicine, Bezmialem Vakif University, Istanbul, Turkey; 16 Astellas Pharma Global Development, Inc., Northbrook, IL USA; 17 State Key Laboratory of Oncology in South China, Collaborative Innovation Center for Cancer Medicine, Sun Yat-Sen University Cancer Center, Guangzhou, China

**Keywords:** Gastric cancer, Oesophageal cancer, Randomized controlled trials, Cancer therapy

## Abstract

There is an urgent need for first-line treatment options for patients with human epidermal growth factor receptor 2 (HER2)-negative, locally advanced unresectable or metastatic gastric or gastroesophageal junction (mG/GEJ) adenocarcinoma. Claudin-18 isoform 2 (CLDN18.2) is expressed in normal gastric cells and maintained in malignant G/GEJ adenocarcinoma cells. GLOW (closed enrollment), a global, double-blind, phase 3 study, examined zolbetuximab, a monoclonal antibody that targets CLDN18.2, plus capecitabine and oxaliplatin (CAPOX) as first-line treatment for CLDN18.2-positive, HER2-negative, locally advanced unresectable or mG/GEJ adenocarcinoma. Patients (*n* = 507) were randomized 1:1 (block sizes of two) to zolbetuximab plus CAPOX or placebo plus CAPOX. GLOW met the primary endpoint of progression-free survival (median, 8.21 months versus 6.80 months with zolbetuximab versus placebo; hazard ratio (HR) = 0.687; 95% confidence interval (CI), 0.544–0.866; *P* = 0.0007) and key secondary endpoint of overall survival (median, 14.39 months versus 12.16 months; HR = 0.771; 95% CI, 0.615–0.965; *P* = 0.0118). Grade ≥3 treatment-emergent adverse events were similar with zolbetuximab (72.8%) and placebo (69.9%). Zolbetuximab plus CAPOX represents a potential new first-line therapy for patients with CLDN18.2-positive, HER2-negative, locally advanced unresectable or mG/GEJ adenocarcinoma. ClinicalTrials.gov identifier: NCT03653507.

## Abstract (web summary)

In the randomized, double-blind, phase 3 GLOW trial, capecitabine and oxaliplatin combined with zolbetuximab, a monoclonal antibody that targets CLDN18.2, significantly improved progression-free survival and overall survival in patients with CLDN18.2-positive, HER2-negative, untreated, locally advanced unresectable or metastatic gastric or gastroesophageal junction adenocarcinoma.

## Main

Gastric adenocarcinoma is the fifth most commonly diagnosed cancer worldwide, and incidence of gastroesophageal junction adenocarcinomas has markedly increased in the last few decades^1–3^. Because patients with early-stage disease are often asymptomatic, gastric or gastroesophageal junction (G/GEJ) adenocarcinomas are frequently diagnosed at an advanced or metastatic stage^1^. These cancers have some of the highest unmet medical needs^1^. Standard first-line therapy for patients with HER2-negative, locally advanced unresectable or metastatic G/GEJ (mG/GEJ) adenocarcinoma has been platinum-fluoropyrimidine chemotherapy; both folinic acid plus 5-fluorouracil and oxaliplatin (FOLFOX) and capecitabine plus oxaliplatin (CAPOX) are accepted standard chemotherapy regimens, although oral regimens are more convenient and preferred in Asia^1,4–8^. Patients receiving standard oxaliplatin-based doublet regimens survive approximately 1 year^1,4,6–8^.

The combination of targeted therapies or immunotherapies with chemotherapy can improve overall survival (OS) in some patients with metastatic disease^8–11^. Trastuzumab is approved for the approximately 15% of patients with HER2-positive disease^3–6,9,12–15^. Targeting programmed death-1 (PD-1) with nivolumab is approved as first-line therapy in more than 50 countries; efficacy has been mainly limited to patients with a programmed death-ligand 1 (PD-L1) combined positive score (CPS) ≥5, which occurs in approximately 20–60% of patients^3–6,10,11,14,15^. In some countries, targeting vascular endothelial growth factor receptor 2 with ramucirumab is approved alone or in combination with paclitaxel as second-line therapy^1,3,4,6,16,17^. An unmet need remains to develop additional targeted therapies to treat patients with HER2-negative, locally advanced unresectable or mG/GEJ adenocarcinoma^1,3,18^.

CLDN18.2 is a tight junction protein exclusively expressed in normal gastric mucosa cells and is retained in most G/GEJ adenocarcinomas^14,19–24^. In normal gastric mucosa, CLDN18.2 is typically buried within tight junctions^19^. During malignant transformation, loss of gastric mucosa cell polarity may result in CLDN18.2 becoming more exposed and, thus, accessible to therapeutic antibodies^15,20–25^.

Zolbetuximab is a first-in-class immunoglobulin G1 monoclonal antibody that targets CLDN18.2 and mediates antibody-dependent cellular cytotoxicity and complement-dependent cytotoxicity in CLDN18.2-positive G/GEJ adenocarcinoma cells^20–22,26,27^. The phase 2b FAST study demonstrated that the combination of zolbetuximab plus chemotherapy prolonged progression-free survival (PFS) and OS when compared with chemotherapy alone; the benefit was further enhanced in patients whose tumors had higher expression of CLDN18.2 (ref. ^22^). The recently reported primary results of SPOTLIGHT (NCT03504397)—a global, phase 3 study in patients with CLDN18.2-positive (≥75% of tumor cells with moderate-to-strong claudin-18 (CLDN18) membranous staining), HER2-negative disease—demonstrated that both PFS and OS were significantly prolonged in patients treated with first-line zolbetuximab plus a modified FOLFOX regimen (mFOLFOX6) compared with placebo plus mFOLFOX6 (ref. ^28^). The GLOW study (NCT03653507) was conducted simultaneously with SPOTLIGHT to confirm the efficacy of the addition of zolbetuximab to chemotherapy in the first-line treatment setting and to examine the addition of zolbetuximab with an alternative first-line chemotherapy backbone that has a different schedule and toxicity profile^1,28,29^. In addition, it was appropriate to assess the combination of zolbetuximab with mFOLFOX6 or CAPOX in patients from different geographic regions where one or the other chemotherapy regimen may be preferred^3–6^.

Here we report the primary analysis of GLOW, a global, randomized, double-blind, phase 3 study, which evaluated the efficacy and safety of first-line treatment with zolbetuximab plus CAPOX compared to placebo plus CAPOX in patients with CLDN18.2-positive, HER2-negative, locally advanced unresectable or mG/GEJ adenocarcinoma.

## Results

### Patients and treatment

**Fig. 1:**
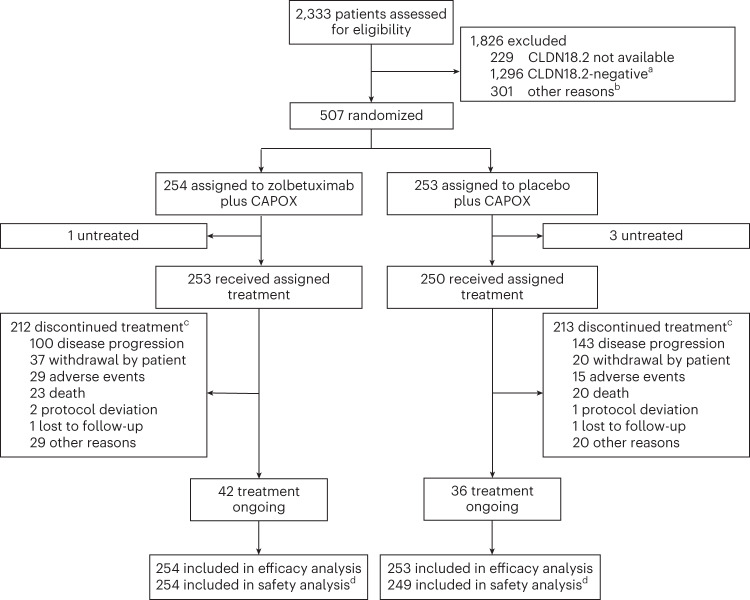
CONSORT diagram of GLOW study. ^a^‘CLDN18.2-positive’ was defined as ≥75% of tumor cells with moderate-to-strong membranous CLDN18 staining as determined by central immunohistochemistry using the investigational VENTANA CLDN18 (43-14A) RxDx Assay. ^b^‘Other’ represents patients whose tumors were CLDN18.2-positive but failed screening for other reasons, including laboratory findings, HER2 expression status, Eastern Cooperative Oncology Group (ECOG) performance status score, other exclusion criteria or withdrawal by patient. ^c^If patients discontinued from both zolbetuximab or placebo and CAPOX on the same day, all reasons for discontinuation were summarized; the sum of values for individual reasons for discontinuation is more than 212 for the zolbetuximab group and more than 213 for the placebo group. ^d^One patient randomized to the placebo plus CAPOX group received one dose of zolbetuximab as a protocol deviation and was moved to the zolbetuximab plus CAPOX group for the safety analysis set.

Between 28 November 2018 and 18 February 2022, a total of 2,333 patients with previously untreated, locally advanced unresectable or mG/GEJ adenocarcinoma were screened at 166 sites in 18 countries. CLDN18.2 tumor status was assessed in 2,104 patients, of whom 808 (38.4%) had tumors that met the cutoff for CLDN18.2 positivity (≥75% of tumor cells with moderate-to-strong CLDN18 membranous staining as determined by central immunohistochemistry using the investigational VENTANA CLDN18 (43-14A) RxDx assay). Among patients whose tumors were assessed for CLDN18.2 status, 1,701 had tumors that were HER2-negative; 729 of 1,701 (42.9%) patients had tumors that met the cutoff for CLDN18.2 positivity. Of screened patients with tumors that were HER2-negative and met the cutoff for CLDN18.2 positivity, 222 were not randomized owing to failure to meet other inclusion criteria or the patient’s decision to withdraw from the study. Ultimately, 507 patients with CLDN18.2-positive tumors were randomly assigned to receive either zolbetuximab plus CAPOX (*n* = 254, hereafter ‘zolbetuximab’) or placebo plus CAPOX (*n* = 253, hereafter ‘placebo’) across 131 study sites (Fig. 1 and Supplementary Table 1). The recruitment period was from 21 January 2019 (first patient randomized) to 18 February 2022 (last patient randomized). At the data cutoff on 7 October 2022, the median trial follow-up was 12.62 months versus 12.09 months for PFS and 17.71 months versus 18.43 months for OS in patients randomized to receive zolbetuximab versus placebo, respectively. Demographic and baseline characteristics were generally well balanced between groups (Table 1 and Supplementary Table 2). As an ad hoc analysis, PD-L1 expression was evaluated using the Dako PD-L1 IHC 28-8 pharmDx assay in a subset of randomized patients for whom consented samples were available for testing; 225 of 288 (78.1%) patients were determined to have tumors with a PD-L1 CPS <5.

**Table 1:** Demographics and clinical characteristics of the ITT population at baseline

Characteristic	Overall (*n* = 507)	Zolbetuximab plus CAPOX (*n* = 254)	Placebo plus CAPOX (*n* = 253)
Median age (range), years	60.0 (21–83)	61.0 (22–82)	59.0 (21–83)
Male sex^a^, *n* (%)	315 (62.1)	159 (62.6)	156 (61.7)
Region, *n* (%)			
Asia	315 (62.1)	157 (61.8)	158 (62.5)
Non-Asia	192 (37.9)	97 (38.2)	95 (37.5)
Organs with metastases, *n* (%)			
0–2	377 (74.4)	189 (74.4)	188 (74.3)
≥3	130 (25.6)	65 (25.6)	65 (25.7)
Prior gastrectomy, *n* (%)			
Yes	150 (29.6)	75 (29.5)	75 (29.6)
No	357 (70.4)	179 (70.5)	178 (70.4)
Primary site, *n* (%)			
Stomach	428 (84.4)	219 (86.2)	209 (82.6)
GEJ	79 (15.6)	35 (13.8)	44 (17.4)
Lauren classification, *n* (%)			
Diffuse	187 (37.0)	87 (34.4)	100 (39.5)
Intestinal	77 (15.2)	36 (14.2)	41 (16.2)
Mixed	41 (8.1)	20 (7.9)	21 (8.3)
Unknown^b^	140 (27.7)	76 (30.0)	64 (25.3)
Other	61 (12.1)	34 (13.4)	27 (10.7)
Missing	1	1	0
ECOG performance status score, *n* (%)			
0	216 (42.9)	108 (42.7)	108 (43.2)
1	287 (57.1)	145 (57.3)	142 (56.8)
Missing^c^	4	1	3
Measurable disease^d^, *n* (%)			
Yes	400 (78.9)	195 (76.8)	205 (81.0)
No	107 (21.1)	59 (23.2)	48 (19.0)

^a^Sex was reported by study site staff through an interactive response technology system with options ‘male’ or ‘female’.

^b^Patients with Lauren classification ‘unknown’ had adenocarcinoma without Lauren classification.

^c^Baseline measurements were reported at cycle 1, day 1. Patients reported as ‘Missing’ did not receive any treatment; thus, no baseline was defined per the Statistical Analysis Plan. However, at screening, these patients had an Eastern Cooperative Oncology Group (ECOG) performance status score of 0 or 1 and were, thus, eligible for enrollment.

^d^Based on central assessment.

The primary endpoint was PFS per Response Evaluation Criteria in Solid Tumors (RECIST) version 1.1 as determined by an independent review committee (IRC). A key secondary endpoint was OS; additional secondary endpoints were objective response rate (ORR) and duration of response (DOR) per RECIST version 1.1 as determined by an IRC and safety and tolerability of zolbetuximab. Time to confirmed deterioration in scores for European Organization for Research and Treatment of Cancer global health status and quality of life, physical functioning and abdominal pain and discomfort assessments as a key secondary endpoint was not reported in this manuscript owing to the pending clinically meaningful threshold from the ongoing exit survey study per protocol. Additional secondary endpoints not reported in this manuscript were additional patient-reported outcomes and pharmacokinetics and immunogenicity of zolbetuximab. Efficacy endpoints were assessed in the intent-to-treat (ITT) population, which included all randomized patients. Safety was assessed in the safety analysis set, which included all patients who received at least one dose of any study drug.

### PFS

**Fig. 2:**
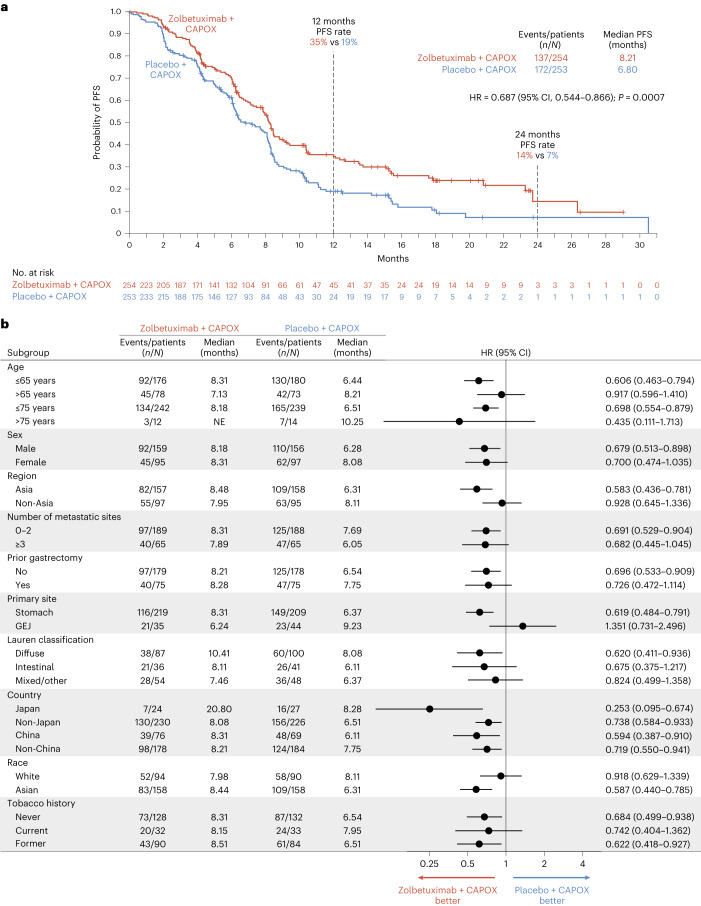
PFS in the ITT population. **a**, Kaplan–Meier PFS curves of all patients. **b**, Forest plots of PFS by subgroups.

**Extended Data Fig. 1:**
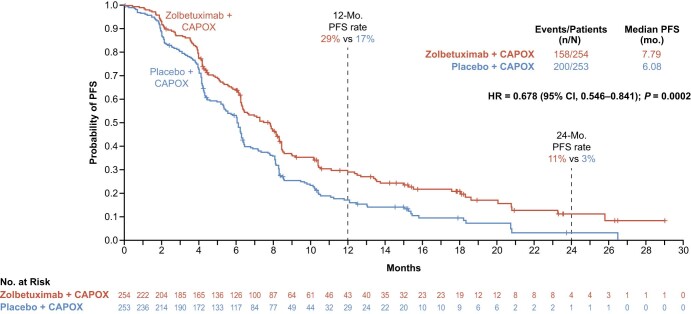
PFS in the ITT population by investigator. Kaplan-Meier curve of PFS in all patients. CAPOX, capecitabine plus oxaliplatin regimen; CI, confidence interval; HR, hazard ratio; ITT, intent-to-treat; mo., months; No., number; PFS, progression-free survival.

PFS as the primary endpoint was statistically significantly prolonged in patients randomized to receive zolbetuximab versus placebo (median, 8.21 months versus 6.80 months, respectively; hazard ratio (HR) = 0.687; 95% confidence interval (CI), 0.544–0.866; *P* = 0.0007) (Fig. 2a). The estimated 12-month PFS rates were 35% in the zolbetuximab arm versus 19% in the placebo arm, and the 24-month PFS rates were 14% versus 7%, respectively, consistently favoring zolbetuximab. PFS was consistently longer in patients in the zolbetuximab arm versus the placebo arm across most of the pre-specified subgroups (Fig. 2b). As a sensitivity analysis, PFS per investigator assessment was also statistically significantly prolonged in patients in the zolbetuximab arm versus the placebo arm (median, 7.79 months versus 6.08 months, respectively; HR = 0.678; 95% CI, 0.546–0.841; *P* = 0.0002) (Extended Data Fig. 1).

### OS

**Fig. 3:**
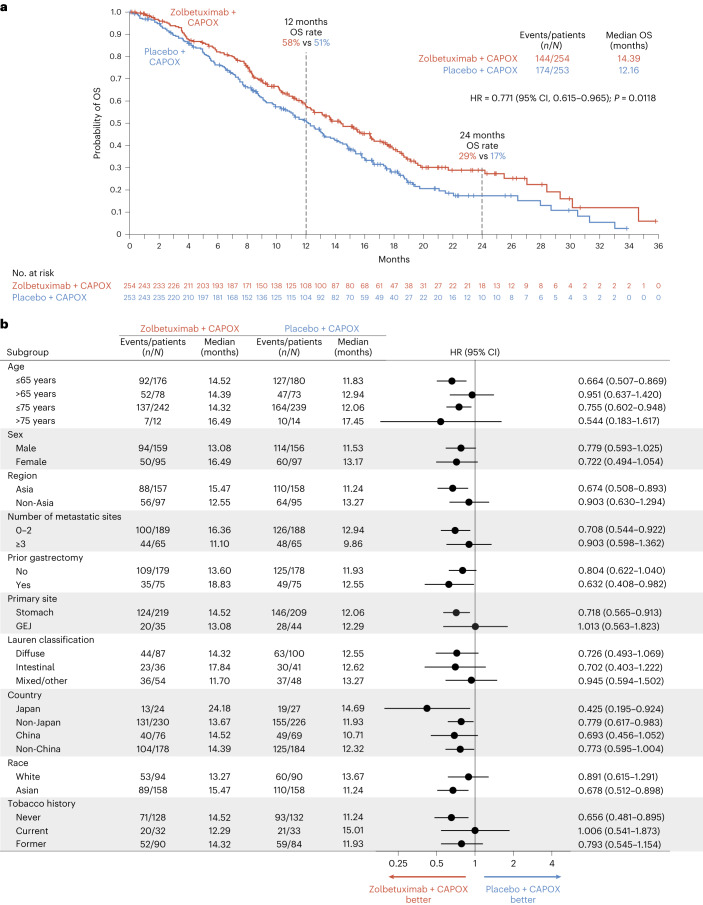
OS in the ITT population. **a**, Kaplan–Meier OS curves of all patients. **b**, Forest plots of OS by subgroups.

At the interim analysis, 318 out of 507 (62.7%) patients had died: 144 of the 254 (56.7%) patients randomized to receive zolbetuximab, and 174 of the 253 (68.8%) patients randomized to receive placebo. OS as a key secondary endpoint was statistically significantly prolonged in patients in the zolbetuximab arm versus the placebo arm (median, 14.39 months versus 12.16 months, respectively; HR = 0.771; 95% CI, 0.615–0.965; *P* = 0.0118) (Fig. 3a). The estimated 12-month OS rates were 58% in the zolbetuximab arm versus 51% in the placebo arm, and the 24-month OS rates were 29% versus 17%, respectively. OS was consistently longer in patients in the zolbetuximab arm versus the placebo arm across most of the pre-specified subgroups (Fig. 3b).

Subsequent anti-cancer therapies were administered to 118 of 254 (46.5%) patients in the zolbetuximab arm versus 140 of 253 (55.3%) patients in the placebo arm (Extended Data Table 1). The types of therapies were well balanced between the zolbetuximab and placebo arms.

### Radiographic response

In the ITT population, as assessed by an IRC as secondary endpoints, ORR was 42.5% (108 of 254 patients; 95% CI, 36.36–48.85) in the zolbetuximab arm versus 40.3% (102 of 253 patients; 95% CI, 34.22–46.64) in the placebo arm, and DOR was 6.14 months (95% CI, 5.03–8.08) versus 6.08 months (95% CI, 4.44–6.34), respectively (Table 2). As an ad hoc analysis, in patients with measurable lesions, as assessed by an IRC, the ORR was 53.8% (105 of 195 patients; 95% CI, 46.58–60.99) in the zolbetuximab arm versus 48.8% (100 of 205 patients; 95% CI, 41.76–55.84) in the placebo arm; a complete response was observed in 3.1% versus 1.5% of patients, and a partial response was observed in 50.8% versus 47.3% of patients, respectively (Extended Data Table 2). In patients with measurable lesions, the median DOR was 6.28 months (95% CI, 5.39–8.28) in the zolbetuximab arm versus 6.18 months (95% CI, 4.53–6.41) in the placebo arm (Extended Data Table 2). As a sensitivity analysis, in the ITT population, as assessed by investigators, the ORR was 40.2% (102 of 254 patients; 95% CI, 34.08–46.47) in the zolbetuximab arm versus 38.3% (97 of 253 patients; 95% CI, 32.32–44.64) in the placebo arm; the median DOR was 6.34 months (95% CI, 5.19–10.12) versus 5.55 months (95% CI, 4.24–6.24), respectively (Extended Data Table 3).

**Table 2:** Anti-tumor activity in the ITT population

Variable	Zolbetuximab plus CAPOX (*n* = 254)	Placebo plus CAPOX (*n* = 253)
ORR^a^		
No. of patients	108	102
% (95% CI)	42.5 (36.36–48.85)	40.3 (34.22–46.64)
BOR, *n* (%)	210 (82.7)	226 (89.3)
CR	9 (3.5)	5 (2.0)
PR	99 (39.0)	97 (38.3)
SD	46 (18.1)	57 (22.5)
PD	11 (4.3)	28 (11.1)
Non-CR/Non-PD	40 (15.7)	33 (13.0)
NE	1 (0.4)	5 (2.0)
ND	4 (1.6)	1 (0.4)
Missing^b^	44	27
Median DOR^a,c^, months (range)	6.14 (5.03–8.08)	6.08 (4.44–6.34)

^a^Per RECIST version 1.1 by IRC.

^b^Patients with missing data had no post-baseline imaging assessment.

^c^DOR is reported among patients with an objective response.

BOR, best overall response; CR, complete response; ND, no disease; NE, not evaluable; PD, progressive disease; PR, partial response; SD, stable disease.

### Safety

Safety was assessed as a secondary endpoint, and the severity of adverse events was graded according to the National Cancer Institute Common Terminology Criteria for Adverse Events version 4.03. The mean duration of exposure to zolbetuximab was 6.40 months (s.d., 6.30) and to placebo was 5.81 months (s.d., 4.88); the mean duration of exposure to CAPOX was similar between the zolbetuximab and placebo arms (Extended Data Table 4). The median duration of exposure to zolbetuximab or placebo, and CAPOX, was also similar between the zolbetuximab and placebo arms (Extended Data Table 4). Treatment-emergent adverse events (TEAEs) of grade ≥3 occurred in 185 of 254 (72.8%) patients in the zolbetuximab arm versus 174 of 249 (69.9%) patients in the placebo arm (Table 3); the most common grade ≥3 TEAEs, based on TEAEs in the zolbetuximab arm, were vomiting (12.2% versus 3.6% of patients, respectively), anemia (10.6% versus 11.2%), neutrophil count decreased (10.2% versus 9.6%) and nausea (8.7% versus 2.4%).

**Table 3:** Adverse events in the safety analysis set

Events, *n* (%)	Zolbetuximab plus CAPOX (*n* = 254)		Placebo plus CAPOX (*n* = 249)	
All-grade TEAEs	251 (98.8)		244 (98.0)	
Grade ≥3 TEAEs	185 (72.8)		174 (69.9)	
Serious TEAEs	120 (47.2)		124 (49.8)	
TEAEs leading to discontinuation of any study drug	79 (31.1)		63 (25.3)	
TRAEs leading to discontinuation of any study drug	55 (21.7)		39 (15.7)	
TEAEs leading to discontinuation of zolbetuximab or placebo	51 (20.1)		36 (14.5)	
TRAEs leading to discontinuation of zolbetuximab or placebo	18 (7.1)		11 (4.4)	
TEAEs leading to death	27 (10.6)		32 (12.9)	
TRAEs leading to death	6 (2.4)		7 (2.8)	
**TEAEs**^**a**^ **by preferred terms**^**b**^	**All grade**	**Grade** **≥3**	**All grade**	**Grade** **≥3**
Nausea	174 (68.5)	22 (8.7)	125 (50.2)	6 (2.4)
Vomiting	168 (66.1)	31 (12.2)	77 (30.9)	9 (3.6)
Decreased appetite	105 (41.3)	17 (6.7)	84 (33.7)	4 (1.6)
Anemia	90 (35.4)	27 (10.6)	91 (36.5)	28 (11.2)
Diarrhea	80 (31.5)	15 (5.9)	86 (34.5)	18 (7.2)
Neutrophil count decreased	70 (27.6)	26 (10.2)	59 (23.7)	24 (9.6)
Aspartate aminotransferase increased	63 (24.8)	6 (2.4)	72 (28.9)	7 (2.8)
Platelet count decreased	61 (24.0)	19 (7.5)	60 (24.1)	20 (8.0)
Hypoalbuminemia	57 (22.4)	8 (3.1)	35 (14.1)	4 (1.6)
Peripheral sensory neuropathy	56 (22.0)	1 (0.4)	56 (22.5)	6 (2.4)
White blood cell count decreased	51 (20.1)	5 (2.0)	39 (15.7)	9 (3.6)
Neutropenia	50 (19.7)	18 (7.1)	35 (14.1)	7 (2.8)
Weight decreased	50 (19.7)	1 (0.4)	25 (10.0)	1 (0.4)
Alanine aminotransferase increased	48 (18.9)	2 (0.8)	52 (20.9)	7 (2.8)
Palmar-plantar erythrodysesthesia syndrome	41 (16.1)	4 (1.6)	49 (19.7)	9 (3.6)
Abdominal pain	40 (15.7)	1 (0.4)	54 (21.7)	4 (1.6)
Constipation	39 (15.4)	−	52 (20.9)	−
Hypokalemia	36 (14.2)	14 (5.5)	36 (14.5)	16 (6.4)
Fatigue	34 (13.4)	7 (2.8)	42 (16.9)	9 (3.6)
Pyrexia	34 (13.4)	1 (0.4)	23 (9.2)	0
Asthenia	33 (13.0)	7 (2.8)	32 (12.9)	3 (1.2)
Malaise	31 (12.2)	1 (0.4)	22 (8.8)	0
Hypoesthesia	30 (11.8)	1 (0.4)	30 (12.0)	0
Thrombocytopenia	28 (11.0)	7 (2.8)	31 (12.4)	7 (2.8)
Insomnia	27 (10.6)	−	16 (6.4)	−
Edema peripheral	26 (10.2)	1 (0.4)	6 (2.4)	0

^a^The all-grade TEAEs reported here occurred in ≥10% of patients in either treatment arm.

^b^Preferred terms were defined according to the Medical Dictionary for Regulatory Activities terminology version 25.0.

**Extended Data Fig. 2:**
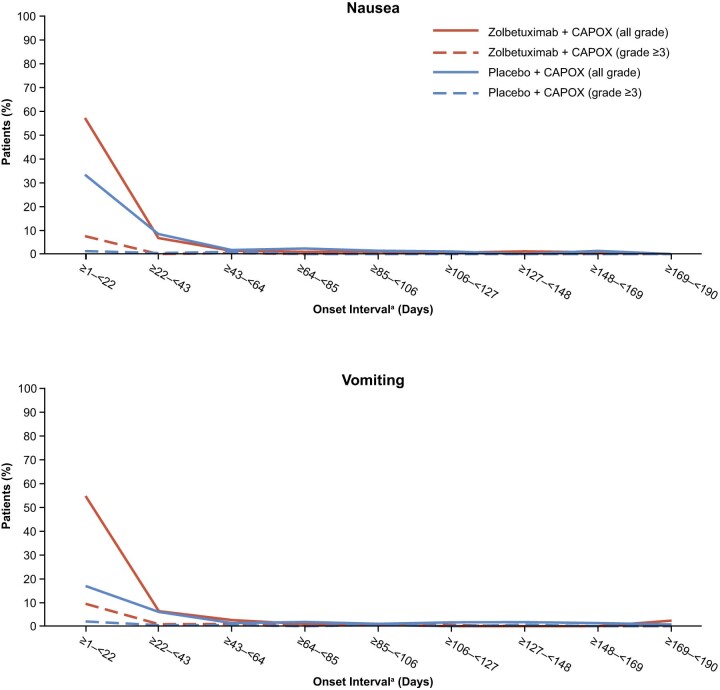
First occurrence of nausea and vomiting in the safety analysis set. Events were counted if they did not occur in any previous onset interval. Individual data points are reported in Supplementary Tables 3 and 4. ^a^The onset day in the onset interval was defined as the date of onset minus the date of first dose plus 1. CAPOX, capecitabine plus oxaliplatin regimen.

**Extended Data Fig. 3:**
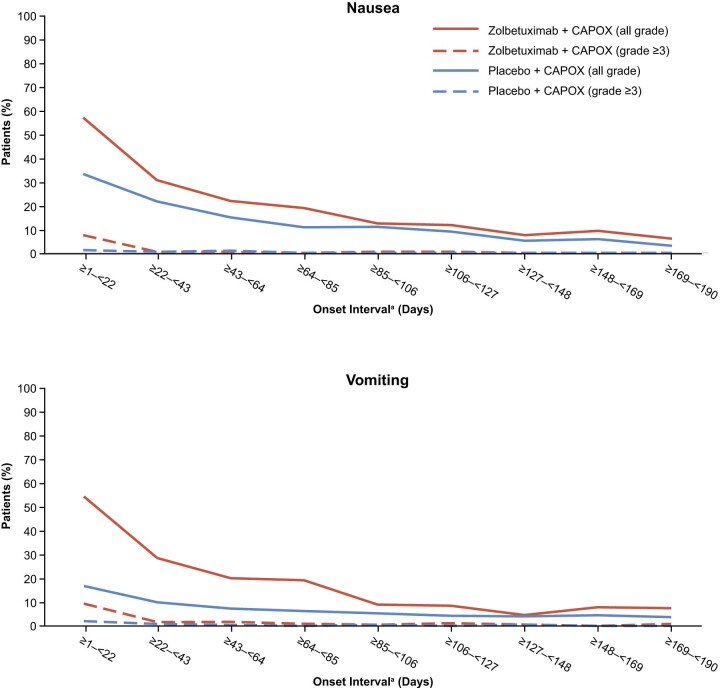
All occurrence of nausea and vomiting in the safety analysis set. Individual data points are reported in Supplementary Tables 5 and 6. ^a^The onset day in the onset interval was defined as the date of onset minus the date of first dose plus 1. CAPOX, capecitabine plus oxaliplatin regimen.

Nausea and vomiting were the only all-grade TEAEs with a more than 10% difference in incidence between patients receiving zolbetuximab versus placebo (nausea, 68.5% versus 50.2%, respectively; vomiting, 66.1% versus 30.9%; Table 3). The incidences of nausea and vomiting were most common during the first treatment cycle and decreased in subsequent cycles (Extended Data Figs. 2 and 3 and Supplementary Tables 3–6). In patients with prior gastrectomy, as either TEAEs or treatment-related adverse events (TRAEs), there was a more than 10% difference in the incidence of vomiting but not nausea in the zolbetuximab compared with the placebo arm, whereas, in patients without prior gastrectomy, there was a more than 10% difference in the incidence of both nausea and vomiting in the zolbetuximab arm compared with the placebo arm (Extended Data Table 5). In patients receiving zolbetuximab but not in patients receiving placebo, nausea and vomiting as TEAEs or as TRAEs were more frequent in patients without prior gastrectomy (Extended Data Table 5).

TRAEs led to discontinuation of zolbetuximab in 18 of 254 (7.1%) patients versus discontinuation of placebo in 11 of 249 (4.4%) patients (Table 3); individual events are reported in Extended Data Table 6. Grade 5 TRAEs occurred in six (2.4%) patients receiving zolbetuximab versus seven (2.8%) patients receiving placebo (Table 3); individual events are reported in Extended Data Table 7.

## Discussion

In GLOW, the addition of first-line zolbetuximab to CAPOX significantly improved PFS and OS in patients with CLDN18.2-positive, HER2-negative, locally advanced unresectable or mG/GEJ adenocarcinoma. GLOW confirmed the increased survival benefit of adding zolbetuximab to chemotherapy observed in previous phase 2 and 3 studies^22,28^. GLOW and SPOTLIGHT showed a similar reduction in the risk of disease progression or death (~31% and ~25%, respectively) and a similar reduction in the risk of death (~25% in both studies) with the addition of zolbetuximab to chemotherapy when compared with placebo plus chemotherapy^28^. As both PFS and OS are time-to-event endpoints, the interpretation of efficacy was based on the HR; the consistent HRs observed in both GLOW and SPOTLIGHT represent a clinically meaningful benefit. The survival benefit for both PFS and OS with zolbetuximab plus CAPOX compared with placebo plus CAPOX was consistently maintained across most of the pre-specified subgroups in GLOW, consistent with SPOTLIGHT^28^. Patients still on trial will continue to be followed-up to collect survival data.

Although the control arm in SPOTLIGHT performed better than expected, in GLOW, the control arm performed as expected, which is in line with other studies that suggest that CLDN18.2 is not a prognostic biomarker^14,28,30^. Notably, SPOTLIGHT enrolled more patients from Japan and South Korea, whereas GLOW enrolled more patients from mainland China, whose disease course tends to be more similar to that of patients from Western countries, with a lower OS than patients from Japan^28,31^. In the ATTRACTION-4 study, which enrolled patients from Japan, South Korea and Taiwan, the control arm demonstrated a longer median OS compared with other global studies^10,32^. Although delay in the separation of the survival curves in GLOW occurred to a lesser degree than in SPOTLIGHT, in both studies, possible explanations for this observation include (1) the mechanism of action of zolbetuximab in inducing the innate immune system through antibody-dependent cellular cytotoxicity or (2) early discontinuation of patients in the zolbetuximab arm due to nausea and vomiting^28^. Taken together, the consistent survival benefits in both GLOW and SPOTLIGHT validate CLDN18.2 as a new target and demonstrate that zolbetuximab prolongs PFS and OS when combined with chemotherapy in patients with CLDN18.2-positive, HER2-negative, locally advanced unresectable or mG/GEJ adenocarcinoma^28^. The clinical responses in the ITT population as evaluated by an IRC were similar between treatment arms in GLOW. Similarly, in SPOTLIGHT, the ORR and DOR were similar in patients treated with zolbetuximab plus mFOLFOX6 compared to placebo plus mFOLFOX6 (ref. ^28^). In GLOW, in patients with measurable lesions, there was a 5% benefit in ORR for patients treated with zolbetuximab plus chemotherapy compared to placebo plus chemotherapy (53.8% versus 48.8%, respectively). The phase 2 FAST study showed a 14% benefit in ORR for patients treated with zolbetuximab plus chemotherapy compared with chemotherapy alone (39% versus 25%, respectively)^22^. In the phase 2 MONO study, zolbetuximab monotherapy demonstrated a 14% ORR in patients whose tumors had high CLDN18.2 expression^23^. The study designs of GLOW and SPOTLIGHT were different from those of FAST and MONO, and, therefore, comparisons across studies should be made with caution^22,23,28^. However, it is possible that zolbetuximab may prolong the duration of disease stabilization when combined with chemotherapy, leading to increased PFS and OS benefits, as opposed to tumor shrinkage; the reason that zolbetuximab did not improve response rates in GLOW and SPOTLIGHT is unclear at this time. Although the response rates between treatment arms were similar in GLOW, the PFS and OS benefits observed in GLOW were clinically meaningful and statistically significant; it is not uncommon for response rates to not correlate with clinical benefits in PFS and OS^33^.

GLOW demonstrated that CLDN18.2 is a prevalent biomarker in HER2-negative, locally advanced unresectable or mG/GEJ adenocarcinoma. In GLOW, 38.4% of screened patients with tumors assessable for CLDN18.2 expression had tumors that met the cutoff for CLDN18.2 positivity. In SPOTLIGHT, 38.4% of screened patients with tumors assessable for CLDN18.2 expression also had CLDN18.2-positive tumors^28^. Notably, the same prevalence rate was observed in both GLOW and SPOTLIGHT despite differences in the representation of countries in these two studies^28^. Previous retrospective studies have suggested that there is no significant correlation between CLDN18.2 positivity and expression of biomarkers such as HER2 and PD-L1; these data suggest similar prevalence of HER2 and PD-L1 in CLDN18.2-positive and CLDN18.2-negative G/GEJ adenocarcinoma^14,30^. In GLOW and SPOTLIGHT, the overlap of CLDN18.2 and PD-L1 tumor expression was evaluated as an ad hoc analysis in a subset of randomized patients; in GLOW, 21.9% of assessed patients had tumors with a PD-L1 CPS ≥5, and, in SPOTLIGHT, 13.2% of assessed patients had tumors with a PD-L1 CPS ≥5 (ref. ^28^). Together, these studies establish CLDN18.2 as a prevalent and unique biomarker that defines a population of patients with CLDN18.2-positive tumors who benefit from targeted therapy with zolbetuximab in combination with chemotherapy. Specifically, targeting of CLDN18.2 with zolbetuximab may fulfill an unmet need among a subset of HER2-negative patients.

In GLOW, the most common TEAEs observed in patients who received zolbetuximab plus CAPOX were nausea and vomiting; these events occurred at a more than 10% difference compared with patients who received placebo plus CAPOX. These results are consistent with the safety profile of zolbetuximab monotherapy and zolbetuximab plus chemotherapy observed in previous phase 1, 2 and 3 studies^21–23,28^. Nausea and vomiting were mostly observed during the first cycle of zolbetuximab treatment, similar to SPOTLIGHT, and the incidences of grade ≥3 events were reduced to less than 2% in later cycles; nausea and vomiting were managed by antiemetics, dose interruptions and infusion rate adjustments^28^. The rate of significant (grade ≥3) nausea and vomiting was lower in GLOW than in SPOTLIGHT in both arms, possibly due to the alignment of the chemotherapy and zolbetuximab administration schedules in GLOW^28^. The effect of nausea and vomiting on patient quality of life will be formally evaluated when the results of time to confirmed deterioration and other patient-reported outcomes data are mature. The manageable safety profile and significant survival benefit indicate a favorable benefit–risk profile for zolbetuximab plus chemotherapy.

This study had some limitations. First, this study was underpowered to statistically determine the effectiveness of zolbetuximab plus CAPOX in the pre-specified subgroups. In all cases, these subgroups were relatively small, and so interpretations should be made with caution. Next, this study did not evaluate the combination of zolbetuximab with nivolumab. Chemotherapy was selected as a relevant comparator arm in GLOW because nivolumab was not approved in this patient population at the time of study initiation. Furthermore, there is a substantial number of patients in this population whose disease does not respond to nivolumab. The ILUSTRO study (NCT03505320) is currently evaluating the efficacy and safety of targeting CLDN18.2 with zolbetuximab plus targeting PD-1 with nivolumab in combination with chemotherapy as first-line treatment for patients with CLDN18.2-positive, HER2-negative, locally advanced unresectable or mG/GEJ adenocarcinoma.

Overall, treatment with zolbetuximab plus CAPOX led to statistically significantly prolonged PFS and OS compared to placebo plus CAPOX in patients with CLDN18.2-positive, HER2-negative, locally advanced unresectable or mG/GEJ adenocarcinoma. These results further confirm the survival benefits observed in the phase 3 SPOTLIGHT study^28^. Together, these studies support the consideration of biomarker testing for tumor expression of CLDN18.2 and the use of zolbetuximab as a first-line therapy in combination with chemotherapy as a new potential standard of care in this biomarker-selected patient population.

## Methods

### Trial oversight

Astellas (the study sponsor) collaborated with the academic authors on the design of the study and on the collection and interpretation of the data after analysis. The protocol and all amendments were approved by the appropriate independent ethics committee (IEC) or institutional review board (IRB) at each participating institution (Supplementary Table 1). Patients provided written informed consent before participating in the trial. All authors attest that the trial was conducted in accordance with the Declaration of Helsinki and the standards of Good Clinical Practice. At pre-specified intervals during study conduct, an independent data monitoring committee reviewed unblinded efficacy and safety data. All authors had access to the study data, were involved in the writing or review and editing of the manuscript and vouch for the fidelity of the trial to the protocol and the completeness and accuracy of the data reported. The manuscript was written by the authors with assistance from a medical writer employed by the sponsor. The trial was registered at ClinicalTrials.gov (NCT03653507).

### Patients

Eligible patients were adults according to local regulations and had CLDN18.2-positive (defined as ≥75% of tumor cells with moderate-to-strong membranous CLDN18 staining as determined by central immunohistochemistry using the investigational VENTANA CLDN18 (43-14A) RxDx Assay), HER2-negative (per local or central testing), previously untreated, locally advanced unresectable or mG/GEJ tumors with radiologically evaluable disease according to RECIST version 1.1. Patients had an ECOG performance status score of 0 or 1 and adequate organ function. Full inclusion and exclusion criteria are as follows:

### Inclusion criteria

General criteria:

1. IRB/IEC-approved written informed consent and privacy language as per national regulations (for example, Health Insurance Portability and Accountability Act (HIPAA) authorization for US sites) must be obtained from the patient or legally authorized representative (if applicable) before any study-related procedures.
2. Patient is considered an adult (for example, ≥18 years of age in the USA) according to local regulation at the time of signing the informed consent.
3. A female patient is eligible to participate if she is not pregnant (negative serum pregnancy test at screening; female patients with elevated serum beta human chorionic gonadotropin (βhCG) and a demonstrated non-pregnant status through additional testing are eligible) and at least one of the following conditions applies.

- Not a woman of childbearing potential (WOCBP) as defined in Protocol Appendix 12.3 Contraception Requirements
- OR
- WOCBP who agrees to follow the contraceptive guidance as defined in Protocol Appendix 12.3 Contraception Requirements throughout the treatment period and for 9 months after the final administration of oxaliplatin and 6 months after the final administration of all other study drugs

4. Female patient must agree not to breastfeed starting at screening and throughout the study period and for 6 months after the final study treatment administration.
5. Female patient must not donate ova starting at screening and throughout the study period and for 9 months after the final administration of oxaliplatin and for 6 months after the final administration of all other study drugs.
6. Male patient with female partner(s) of childbearing potential must agree to use contraception as detailed in Protocol Appendix 12.3 Contraception Requirements during the treatment period and for 6 months after the final study treatment administration.
7. Male patient must not donate sperm during the treatment period and for 6 months after the final study treatment administration.
8. Male patient with a pregnant or breastfeeding partner(s) must agree to remain abstinent or use a condom for the duration of the pregnancy or time partner is breastfeeding throughout the study period and for 6 months after the final study treatment administration.
9. Patient agrees not to participate in another interventional study while receiving study drug in the present study.

Disease-specific criteria:

10. Patient has histologically confirmed diagnosis of G/GEJ adenocarcinoma.
11. Patient has radiologically confirmed locally advanced unresectable or metastatic disease within 28 days before randomization.
12. Patient has radiologically evaluable disease (measurable and/or non-measurable) according to RECIST version 1.1, per local assessment, ≤28 days before randomization. For patients with only one evaluable lesion and prior radiotherapy ≤3 months before randomization, the lesion must either be outside the field of prior radiotherapy or have documented progression after radiation therapy.
13. Patient’s tumor expresses CLDN18.2 in ≥75% of tumor cells, demonstrating moderate-to-strong CLDN18 membranous staining as determined by central immunohistochemistry testing.
14. Patient has a HER2-negative tumor as determined by local or central testing on a G/GEJ tumor specimen.

Physical or laboratory findings:

15. Patient has a ECOG performance status score 0 or 1.
16. Patient has predicted life expectancy ≥12 weeks, in the opinion of the investigator.
17. Patient must meet all of the following criteria based on the centrally or locally analyzed laboratory tests collected within 14 days before randomization. In the case of multiple sample collections within this period, the most recent sample collection with available results should be used to determine eligibility.

- Hemoglobin ≥9 g dl^−1^. Patients requiring transfusions are eligible if they have a post-transfusion hemoglobin ≥9 g dl^−1^.
- Absolute neutrophil count (ANC) ≥1.5 × 10^9^/l
- Platelets ≥100 × 10^9^/l
- Albumin ≥2.5 g dl^−1^
- Total bilirubin ≤1.5× upper limit of normal (ULN) without liver metastases (or <3.0× ULN if liver metastases are present)
- Aspartate aminotransferase and alanine aminotransferase ≤2.5× ULN without liver metastases (or ≤5× ULN if liver metastases are present)
- Estimated creatinine clearance ≥30 ml min^−1^
- Prothrombin time/international normalized ratio and partial thromboplastin time ≤1.5× ULN (except for patients receiving anti-coagulation therapy)

### Exclusion criteria

Prohibited treatment or therapies:

1. Patient has received prior systemic chemotherapy for locally advanced unresectable or mG/GEJ adenocarcinoma. However, patient may have received either neoadjuvant or adjuvant chemotherapy, immunotherapy or other systemic anti-cancer therapies as long as it was completed at least 6 months before randomization.
2. Patient has received radiotherapy for locally advanced unresectable or mG/GEJ adenocarcinoma ≤14 days before randomization and has not recovered from any related toxicity.
3. Patient has received treatment with herbal medications or other treatments that have known anti-tumor activity within 28 days before randomization.
4. Patient has received systemic immunosuppressive therapy, including systemic corticosteroids, within 14 days before randomization. Patient using a physiologic replacement dose of hydrocortisone or its equivalent (defined as up to 30 mg per day of hydrocortisone or up to 10 mg per day of prednisone), receiving a single dose of systemic corticosteroids or receiving systemic corticosteroids as premedication for radiologic imaging contrast use is eligible.
5. Patient has received other investigational agents or devices within 28 days before randomization.

Medical history or concurrent disease:

6. Patient has prior severe allergic reaction or intolerance to known ingredients of zolbetuximab or other monoclonal antibodies, including humanized or chimeric antibodies.
7. Patient has known immediate or delayed hypersensitivity, intolerance or contraindication to any component of study treatment.
8. Patient has prior severe allergic reaction or intolerance to any component of CAPOX.
9. Patient has known dihydropyrimidine dehydrogenase deficiency. (Note that screening for dihydropyrimidine dehydrogenase deficiency should be conducted per local requirements.)
10. Patient has a complete gastric outlet syndrome or a partial gastric outlet syndrome with persistent/recurrent vomiting.
11. Per investigator judgment, patient has significant gastric bleeding and/or untreated gastric ulcers that exclude the patient from participation.
12. Patient has a known history of a positive test for human immunodeficiency virus (HIV) infection or known active hepatitis B (HB; positive HB surface antigen (HBs Ag)) or hepatitis C infection. (Note: Screening for these infections should be conducted per local requirements.)

- For patients who are negative for HBs Ag but HB core antibody (HBc Ab) positive, an HB DNA test will be performed, and, if positive, the patient will be excluded.
- Patients with positive hepatitis C virus (HCV) serology but negative HCV RNA test are eligible.
- Patients treated for HCV with undetectable viral load results are eligible.

13. Patient has an active autoimmune disease that has required systemic treatment within the past 3 months before randomization.
14. Patient has an active infection requiring systemic therapy that has not completely resolved within 7 days before randomization.
15. Patient has significant cardiovascular disease, including any of the following.

- Congestive heart failure (defined as New York Heart Association class III or IV), myocardial infarction, unstable angina, coronary angioplasty, coronary stenting, coronary artery bypass graft, cerebrovascular accident or hypertensive crisis within 6 months before randomization
- History of clinically significant ventricular arrhythmias (that is, sustained ventricular tachycardia, ventricular fibrillation or Torsades de Pointes)
- QTc interval >450 ms for male patients; QTc interval >470 ms for female patients
- History or family history of congenital long QT syndrome
- Cardiac arrhythmias requiring anti-arrhythmic medications (patients with rate controlled atrial fibrillation for >1 month before randomization are eligible)

16. Patient has history of central nervous system (CNS) metastases and/or carcinomatous meningitis from G/GEJ cancer.
17. Patient has known peripheral sensory neuropathy grade >1 unless the absence of deep tendon reflexes is the sole neurological abnormality.
18. Patient has had a major surgical procedure ≤28 days before randomization.
19. Patient without complete recovery from a major surgical procedure ≤14 days before randomization.
20. Patient has psychiatric illness or social situations that would preclude study compliance, per investigator judgment.
21. Patient has another malignancy for which treatment is required, per investigator judgment.
22. Patient has any concurrent disease, infection or comorbid condition that interferes with the ability of the patient to participate in the study, which places the patient at undue risk or complicates the interpretation of data, in the opinion of the investigator.

### Study design and treatment

GLOW is a global, randomized, double-blind, phase 3 trial. Patients were randomly assigned 1:1 to receive intravenous infusion of zolbetuximab 800 mg/m^2^ (cycle 1, day 1) followed by 600 mg/m^2^ (day 1 of subsequent cycles) plus CAPOX (oral capecitabine, 1,000 mg/m^2^, twice daily on days 1–14 of each cycle; intravenous infusion of oxaliplatin, 130 mg/m^2^, day 1 of each cycle) for eight 21-day cycles versus placebo plus CAPOX. Patients continued beyond eight cycles with zolbetuximab or placebo, plus, at the investigator’s discretion, capecitabine, until disease progression, unacceptable toxicity, start of another anti-cancer treatment or other discontinuation criteria were met as specified in the protocol (Supplementary Information).

Randomization was performed by blinded site staff using interactive response technology by block randomization with block sizes of two and was stratified according to region (Asia versus non-Asia), number of organs with metastases (0–2 versus ≥3) and prior gastrectomy (yes versus no). Countries in the Asia subgroup analysis were mainland China, Japan, South Korea, Malaysia, Taiwan (province of China) and Thailand, and countries in the non-Asia subgroup analysis were Argentina, Canada, Croatia, Greece, Ireland, The Netherlands, Portugal, Romania, Spain, Turkey, UK and USA. The randomization list and study drug blinding were maintained by the interactive response technology system. The sponsor, investigators, clinical staff and patients remained blinded to treatment throughout the study. To maintain blinding, zolbetuximab and placebo, which were identical in appearance and form, were provided to investigators or designees by an unblinded pharmacist and administered in identical volumes, routes and schedules.

### Endpoints

The primary endpoint was PFS per RECIST version 1.1 as determined by an IRC. Key secondary endpoints were OS and time to confirmed deterioration in scores for European Organization for Research and Treatment of Cancer global health status and quality of life, physical functioning and abdominal pain and discomfort assessments, which were determined as clinically meaningful to patients; time to confirmed deterioration data are pending the clinically meaningful threshold obtained from the ongoing exit survey study per protocol and will be reported in a future publication. Additional secondary endpoints were ORR and DOR per RECIST version 1.1 as determined by an IRC, safety and tolerability of zolbetuximab, additional patient-reported outcomes and pharmacokinetics and immunogenicity of zolbetuximab; patient-reported outcomes will also be reported in a future publication.

PFS, OS, ORR and DOR were assessed in the ITT population, which consisted of all randomized patients. Safety was assessed in all patients who received at least one dose of any study drug.

### Assessments

Tumor response was assessed by imaging at screening, every 9 weeks for the first 54 weeks of treatment and every 12 weeks thereafter until disease progression or start of another anti-cancer treatment. Survival was assessed at least every 12 weeks during follow-up. Patients completed health-related quality of life assessments, including the EuroQOL EQ-5D-5L and the European Organization for Research and Treatment of Cancer QLQ-C30, QLQ-OG25 plus STO22 and Global Pain at screening, every 3 weeks during study treatment, at study treatment discontinuation and 30 and 90 days after study treatment discontinuation. Adverse events, graded according to the National Cancer Institute Common Terminology Criteria for Adverse Events version 4.03, were evaluated throughout the trial and for 90 days after study treatment discontinuation. Data were collected at study sites where study treatment was administered.

### Statistical analysis

The Kaplan–Meier method was used to estimate the median and distribution of PFS, OS and DOR; stratified log-rank tests were used to assess between-group differences, and the stratified Cox proportional hazard model was used to calculate HRs and associated 95% CIs. The Cochran–Mantel–Haenszel test was used to assess between-group differences in ORR. Pre-specified multiplicity adjustment methods were used to control the overall one-sided type I error rate at 0.025. Efficacy boundaries were calculated based on the information fraction at the time of analysis. The reported 95% CIs describe the precision of the point estimates and may not correspond to the significance of the test. The study aimed to enroll 500 patients. The final analysis of PFS was planned when 300 patients experienced disease progression or death to provide 93.4% power to detect a between-group difference with the assumption of median PFS of 9 months with zolbetuximab plus CAPOX versus 6 months with placebo plus CAPOX (HR = 0.67) at an overall one-sided alpha level of 0.025. An interim analysis of OS was planned at the final PFS analysis, and a final analysis of OS was planned after 386 deaths to provide 80% power to detect a between-group difference with the assumption of median OS of 14.7 months with zolbetuximab plus CAPOX versus 11 months with placebo plus CAPOX (HR = 0.75) at an overall one-sided alpha level of 0.025. An efficacy boundary was calculated for the interim OS based on the information fraction at the time of the interim analysis; a one-sided level of significance of 0.0135 was used with an 82.4% information fraction. To strictly control the type I error rate at an alpha level of 0.025, OS was tested only if the null hypothesis of the final PFS analysis was rejected. Full details of the statistical analysis plan are provided in the protocol (Supplementary Information).

Collected data were entered using the RAVE electronic data collection system. Sample size calculations were performed with East version 6.4 software. Statistical data analyses were performed with SAS version 9.3 or higher software.

### Reporting summary

Further information on research design is available in the Nature Portfolio Reporting Summary linked to this article.

## Online content

Any methods, additional references, Nature Portfolio reporting summaries, source data, extended data, supplementary information, acknowledgements, peer review information; details of author contributions and competing interests; and statements of data and code availability are available at 10.1038/s41591-023-02465-7.

### Supplementary information

Supplementary Information Supplementary Tables 1–6, Study Protocol, Statistical Analyis Plan (SAP) and SAP amendments

Reporting Summary

## Figures and Tables

**Extended Data Table 1:**
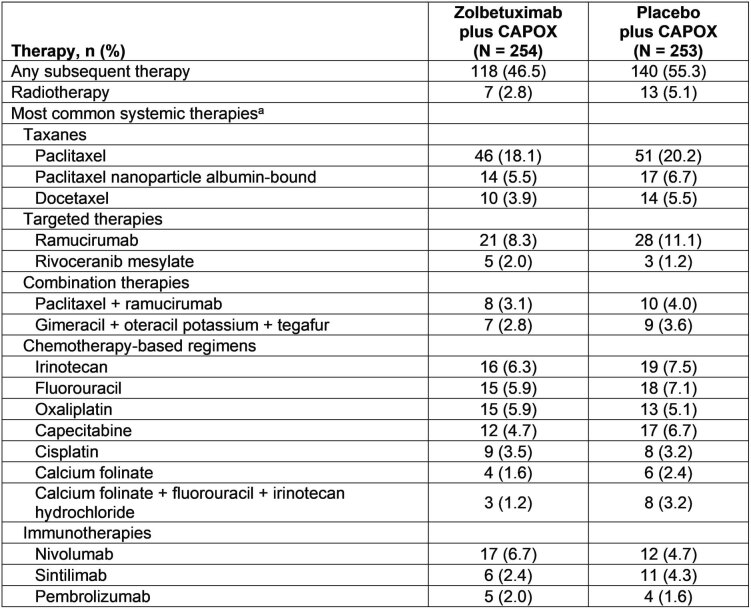
Subsequent anti-cancer therapies in the ITT population

^a^ Systemic therapies administered to ≥2% of patients in either treatment arm are reported.

**Extended Data Table 2:**
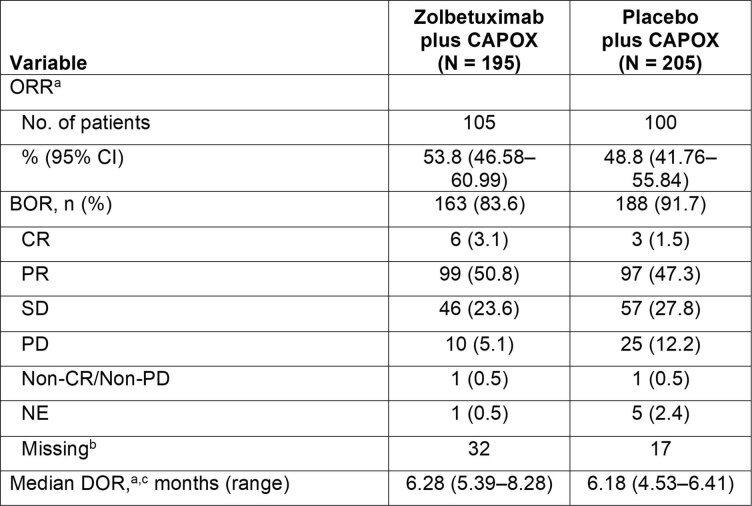
Anti-tumor activity in patients with measurable lesions

^a^ Per RECIST version 1.1 by IRC.

^b^ Patients with missing data had no post-baseline imaging assessment.

^c^ DOR is reported among patients with an objective response.

BOR, best overall response; CR, complete response; ND, no disease; NE, not evaluable; No., number; PD, progressive disease; PR, partial response; SD, stable disease.

**Extended Data Table 3:**
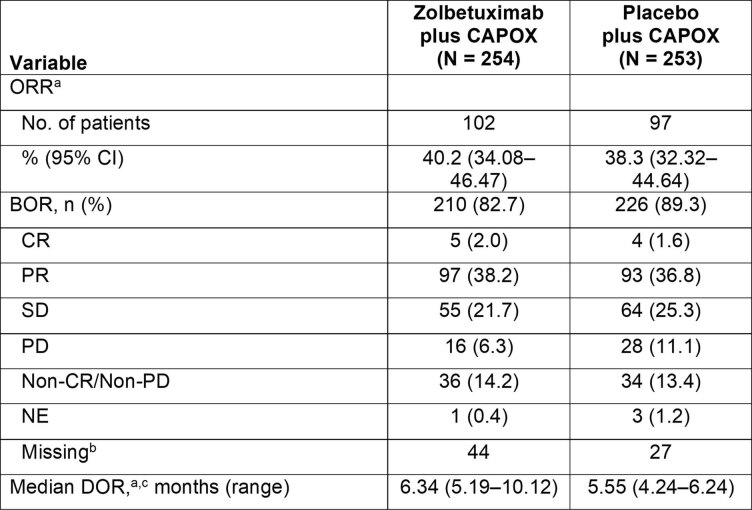
Anti-tumor activity in the ITT population assessed by investigator

^a^ Per RECIST version 1.1.

^b^ Patients with missing data had no post-baseline imaging assessment.

^c^ DOR is reported among patients with an objective response.

BOR, best overall response; CR, complete response; ND, no disease; NE, not evaluable; No., number; PD, progressive disease; PR, partial response; SD, stable disease.

**Extended Data Table 4:**
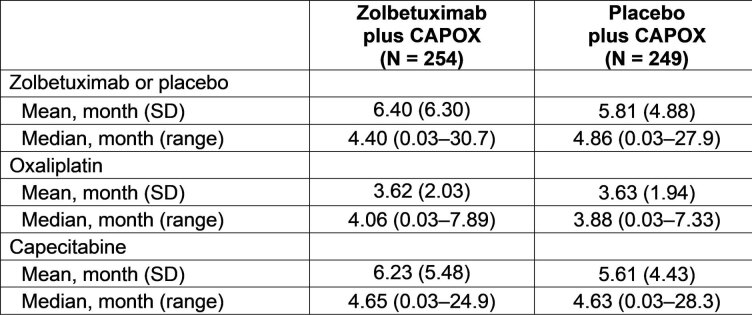
Duration of exposure of each treatment component in the safety analysis set

The duration of each component is defined as date of last dose minus date of first dose plus 1.

**Extended Data Table 5:**
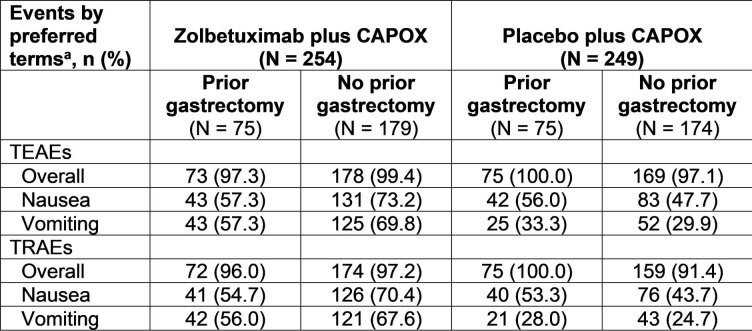
Incidence of nausea and vomiting in patients with or without prior gastrectomy

^a^ Preferred terms were defined according to the Medical Dictionary for Regulatory Activities terminology

version 25.0.

**Extended Data Table 6:**
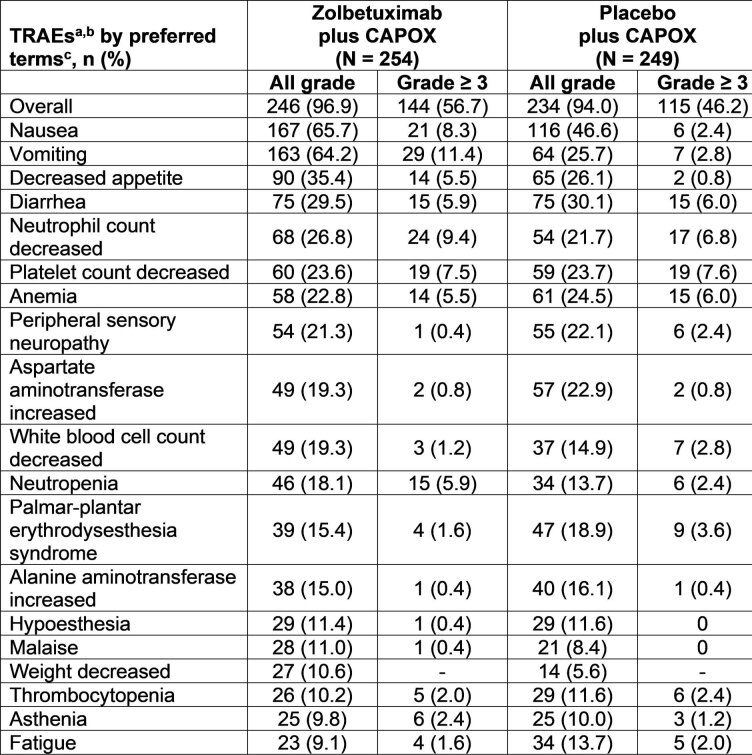
TRAEs in the safety analysis set

^a^ The all-grade TRAEs reported here occurred in ≥10% of patients in either treatment arm.

^b^ Adverse events with a reasonable possibility of relationship as assessed by the investigator or missing relationship are reported here.

^c^ Preferred terms were defined according to the Medical Dictionary for Regulatory Activities terminology version 25.0.

**Extended Data Table 7:**
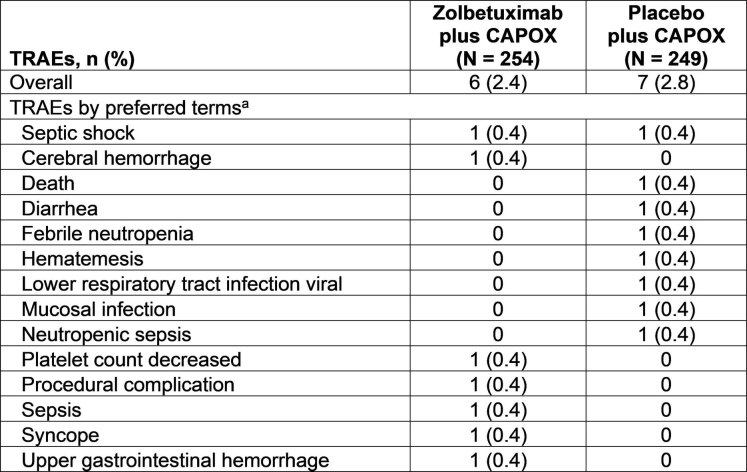
Grade 5 TRAEs leading to death in the safety analysis set

^a^ Preferred terms were defined according to the Medical Dictionary for Regulatory Activities terminology version 25.0.

## Data Availability

Upon request, and subject to certain criteria, conditions and exceptions, Astellas will provide access to anonymized patient-level data from completed Astellas-sponsored phase 1 to phase 4 interventional clinical studies conducted for products and indications that have been approved in any country and also for studies conducted for terminated compounds. Approval must have been granted by the agencies of the main regions: the USA the European Union and Japan. If approval is sought in only one or two regions, approval must have been granted by those agencies. Where available, the following anonymized patient-level data and information are provided for each clinical study: raw dataset, analysis-ready dataset, protocols with any amendments or addenda, annotated case report form, statistical analysis plan, dataset specifications and clinical study report. Additionally, data may be available upon reasonable request. Researchers may request access to anonymized participant-level data, trial-level data and protocols from Astellas-sponsored clinical trials at https://www.clinicalstudydatarequest.com/. For the Astellas criteria on data sharing, see https://clinicalstudydatarequest.com/Study-Sponsors/Study-Sponsors-Astellas.aspx. Patients remain on trial; for this reason, patient-level data are not available for this trial until completion.
