# Supplementary material for: Zolbetuximab plus CAPOX in CLDN18.2-positive gastric or gastroesophageal junction adenocarcinoma: the randomized, phase 3 GLOW trial
Source: Nat Med. 2023 Jul 31;29(8):2133–41. doi: 10.1038/s41591-023-02465-7 (PMC10427418; doi:10.1038/s41591-023-02465-7)
Supplement: Supplementary file 1 — Supplementary Tables 1–6, Study Protocol, Statistical Analyis Plan (SAP) and SAP amendments [file 41591_2023_2465_MOESM1_ESM.pdf]

nature medicine

Article https://doi.org/10.1038/s41591-023-02465-7

Zolbetuximab plus CAPOX in CLDN18.2-positive gastric or gastroesophageal junction adenocarcinoma: the randomized, phase 3 GLOW trial

In the format provided by the authors and unedited

Supplementary Table 1. Participating study sites by country

Country	Sites
Argentina	Clinica Viedma, Centro de Investigacion Pergamino SA - Clinica Pergamino
Canada	Centre Integre de Sante et des Services Sociaux du Bas-Saint, Tom Baker Cancer Centre
China	Sun Yat-sen University - Cancer Center, The First Hospital Bethune of Jilin University, Huazhong University of Science and Technology - Wuhan Union, Zhejiang Cancer Hospital, Tianjin Medical University Cancer Hospital, Xuzhou Central Hospital, Henan Cancer Hospital - Oncology, Xi'an Jiaotong University - School of Medicine - The First Affiliated Hospital, Fujian Provincial Cancer Hospital - Abdominal Oncology, Changzhou First People's Hospital, The First affiliated Hospital of Zhengzhou University, The First Affiliated Hospital of Bengbu Medical College, The Second Affiliated Hospital of Soochow University, Xiangya Hospital of Central South University, The First Affiliated Hospital of Xiamen University, Xin Jiang Tumor Hospital, Hainan General Hospital, First Affiliated Hospital of Fujian Medical University, Cancer Institute & Hospital of Chinese Academy of Medical Science - Internal Medicine, Second Affiliated Hospital of Zhejiang University of College of Medicine, Fujian Provincial

	Cancer Hospital - Abdominal Oncology, Shandong Linyi Tumor Hospital, The People’s Hospital of Sichuan Province Oncology, Tianjin Medical University General Hospital, The Sixth Affiliated Hospital ,Sun Yat-Sen University, The First Affiliated Hospital of University of South China, Affiliated Hospital of Hebei University - Oncology, The People's Hospital of Guangxi Zhuang Automous Region, Wuxi Fourth People's Hospital - Oncology, Beijing Cancer Hospital, China-Japan Friendship Hospital
Croatia	Opca bolnica Varazdin, Klinichki Bolnicki Centar-Zagreb, University Hospital "Sisters of Mercy"
Greece	General Oncology Hospital of Kifissia Agioi Anargiroi, Anticancer Hospital of Thessaloniki "Theagenio," University General Hospital of Herakleio, Papageorgiou General Hospital of Thessaloniki, University General Hospital of Patras
Ireland	St James Hospital
Japan	Kanagawa Cancer Center, Chiba Cancer Center, St. Marianna University Hospital, Kagawa University Hospital, Tochigi Cancer Center, National Cancer Center Hospital East, National Hospital Organization Kyushu Cancer Center, Hyogo Cancer Center, Shikoku

	Cancer Center, National Cancer Center Hospital, The Cancer Institute Hospital of JFCR
Korea, Republic Of	Korea University Anam Hospital, Kyungpook National University Chilgok Hospital, Seoul National University Boramae Medical Center, National Cancer Center, CHA Bundang Medical Cente of CHA University - Oncology, Chonnam National University Hwasun Hospital, Ajou University Hospital - Hematology/Oncology, Chonbuk National University Hospital, Seoul National University Bundang Hospital, Asan Medical Center, Korea University Medical Center Guro Hospital, Gachon University Gil Hospital
Malaysia	Hospital Pulau Pinang, University Malaya Medical Centre, Hospital Kuala Lumpur
Netherlands	Elisabeth Tweesteden ziekenhuis, UMC Groningen
Portugal	Hospital da Luz, Centro Hospitalar do Porto-Hospital Santo Antonio, Centro Hospitalar Lisboa Norte - Hospital Santa Maria, H. Sao Pedro de Vila Real. Centro Hospitalar Tras os Montes, Instituto Portuges de Oncologia de Francisco Gentil, Hospital Braga, Instituto Portuges de Oncologia de Francisco Gentil, Hospital Senhora da Oliveira - Guimaraes
Romania	Institutul Clinic Fundeni, Centrul de Oncologie Sf. Nectarie, Radiotherapy Center CJ radioterapie si

	chimioterapie adulti, Institutul Oncologic Prof. Dr. Ion. Chiricuta, Institutul Regional de Oncologie Iasi, Oncomed SRL
Spain	H.G.U. de Elche, Hospital Universitario La Paz, Hospital Clinico San Carlos, Clinica Universitaria de Navarra, C.H.U. A Coruna, Hospital Clinic de Barcelona, Hospital Universitari i Politecnic La Fe, Hospital Universitario 12 de octubre, Institut Catala d Oncologia- Hospital Duran i Reynals, Hospital Universitari General de Catalunya, Hospital Regional Universitario de Malaga, Hospital Clinico de Valencia, Clinica Universitaria de Navarra
Taiwan, Province of China	Kaohsiung Medical University Hospital, Taichung Veteran General Hospital, Chi Mei Medical Center - Liouying - Hematology and Oncology
Thailand	Chiang Mai University - Faculty of Medicine, King Chulalongkorn Memorial Hospital, Srinagarind Hospital, Songklanagarind Hospital, Ramathibodi Hospital, Thammasat University Hospital, Vajira Hospital, Bumrungrad Hospital Public Company Ltd. Clinical Research C, Siriraj Hospital, Police General Hospital, Chulabhorn Hospital
Turkey	Goztepe Prof Dr Suleyman Yalcin sehir Hastanesi, Ondokuz Mayis University, Necmettin Erbakan University Meram Medical Faculty, Bezmi Alem Vakif University Medical Faculty, Inonu Universitesi Turgut

	Ozal Tip Merkezi, Ege University Medical Faculty, Akdeniz Universitesi, Istanbul Uni. Cerrahpasa Med. Fac.
United Kingdom	Guy's and St Thomas' NHS Trust- St Thomas' Hospital, Bristol Haematology and Oncology Centre, Velindre Hospital, Mount Vernon Cancer Centre
United States	University of Texas Southwestern - Simmons Cancer Center, Pacific Cancer Medical Center Inc, Weill Cornell Medical College, Houston Institute for Clinical Research, Parkland Health & Hospital System, Utah Cancer Specialists

Supplementary Table 2. Patient enrollment by country in the ITT population

Country, n (%)	Zolbetuximab plus CAPOX (N = 254)	Placebo plus CAPOX (N = 253)
Argentina	2 (0.8)	0
Canada	1 (0.4)	1 (0.4)
China, Mainland	76 (29.9)	69 (27.3)
Croatia	2 (0.8)	4 (1.6)
Greece	8 (3.1)	4 (1.6)
Ireland	1 (0.4)	2 (0.8)
Japan	24 (9.4)	27 (10.7)
Korea, Republic of	25 (9.8)	25 (9.9)
Malaysia	10 (3.9)	9 (3.6)
Netherlands	1 (0.4)	5 (2.0)
Portugal	14 (5.5)	12 (4.7)
Romania	15 (5.9)	10 (4.0)
Spain	28 (11.0)	29 (11.5)
Taiwan, Province of China	4 (1.6)	7 (2.8)
Thailand	18 (7.1)	21 (8.3)
Turkey	17 (6.7)	20 (7.9)
United Kingdom	4 (1.6)	6 (2.4)
United States	4 (1.6)	2 (0.8)

CAPOX, capecitabine plus oxaliplatin regimen; ITT, intent-to-treat.

Supplementary Table 3. First occurrence of nausea in the safety analysis set

	Incidence of Nausea (%)			
	Zolbetuximab plus CAPOX (N = 254)		Placebo plus CAPOX (N = 249)	
**Onset interval (days)[a]**	**All grade**	**Grade ≥ 3**	**All grade**	**Grade ≥ 3**
≤ 1 to < 22	57.1	7.5	33.3	1.2
≤ 22 to < 43	6.7	0	8.4	0.4
≤ 43 to < 64	1.3	0.4	1.7	0.9
≤ 64 to < 85	0.9	0	2.3	0
≤ 85 to < 106	1.0	0.5	1.4	0
≤ 106 to < 127	0.5	0.5	1.1	0
≤ 127 to < 148	1.1	0	0	0
≤ 148 to < 169	0.7	0	1.3	0
≤ 169 to < 190	0	0	0	0

Events were counted if they did not occur in any previous onset interval.

[a]The onset day in the onset interval was defined as the date of onset minus the date of first dose plus 1.

CAPOX, capecitabine plus oxaliplatin regimen.

Supplementary Table 4. First occurrence of vomiting in the safety analysis set

	**Incidence of Vomiting (%)**			
	**Zolbetuximab plus CAPOX (N = 254)**		**Placebo plus CAPOX (N = 249)**	
**Onset interval (days)[a]**	**All grade**	**Grade ≥ 3**	**All grade**	**Grade ≥ 3**
≤ 1 to < 22	54.7	9.4	16.9	2.0
≤ 22 to < 43	6.3	0.8	6.0	0.4
≤ 43 to < 64	2.6	0.9	1.3	0.4
≤ 64 to < 85	0.9	0.5	1.8	0
≤ 85 to < 106	0.5	0.5	1.0	0.5
≤ 106 to < 127	0	0.5	1.6	0
≤ 127 to < 148	0	0	1.7	0.6
≤ 148 to < 169	0	0	1.3	0
≤ 169 to < 190	2.3	0	0.7	0

Events were counted if they did not occur in any previous onset interval.

[a]The onset day in the onset interval was defined as the date of onset minus the date of first dose plus 1.

CAPOX, capecitabine plus oxaliplatin regimen.

Supplementary Table 5. All occurrence of nausea in the safety analysis set

	Incidence of Nausea (%)			
	Zolbetuximab plus CAPOX (N = 254)		Placebo plus CAPOX (N = 249)	
**Onset interval (days)[a]**	**All grade**	**Grade ≥ 3**	**All grade**	**Grade ≥ 3**
≤ 1 to < 22	57.1	7.5	33.3	1.2
≤ 22 to < 43	30.7	0.4	21.7	0.4
≤ 43 to < 64	21.9	0.4	15.0	0.9
≤ 64 to < 85	18.9	0	10.8	0
≤ 85 to < 106	12.4	0.5	11.0	0
≤ 106 to < 127	11.7	0.5	9.0	0
≤ 127 to < 148	7.5	0	5.1	0
≤ 148 to < 169	9.3	0	5.8	0
≤ 169 to < 190	6.0	0	3.0	0

[a]The onset day in the onset interval was defined as the date of onset minus the date of first dose plus 1.

CAPOX, capecitabine plus oxaliplatin regimen.

Supplementary Table 6. All occurrence of vomiting in the safety analysis set

	Incidence of Vomiting (%)			
	Zolbetuximab plus CAPOX (N = 254)		Placebo plus CAPOX (N = 249)	
**Onset interval (days)[a]**	**All grade**	**Grade ≥ 3**	**All grade**	**Grade ≥ 3**
≤ 1 to < 22	54.7	9.4	16.9	2.0
≤ 22 to < 43	28.7	1.6	10.0	0.8
≤ 43 to < 64	20.2	1.7	7.3	0.4
≤ 64 to < 85	19.3	0.9	6.3	0
≤ 85 to < 106	9.0	0.5	5.3	0.5
≤ 106 to < 127	8.5	1.1	4.3	0
≤ 127 to < 148	4.6	0.6	4.0	0.6
≤ 148 to < 169	7.9	0	4.5	0
≤ 169 to < 190	7.5	0.8	3.7	0

[a]The onset day in the onset interval was defined as the date of onset minus the date of first dose plus 1.

CAPOX, capecitabine plus oxaliplatin regimen.

GLOW study protocol and statistical analysis plan

This supplement contains the following items:

1. Original protocol, p. 13–141
2. Final protocol, p. 142–288
3. Summary of protocol changes, p. 289–460
4. Original statistical analysis plan, p. 461–505
5. Final statistical analysis plan, p. 506–554
6. Original interim analysis plan, p. 555–601
7. Final interim analysis plan, p. 602–674

A Phase 3, Global, Multi-Center, Double-Blind, Randomized, Efficacy Study of IMAB362 Plus CAPOX Compared with Placebo Plus CAPOX as First-line Treatment of Subjects with Claudin (CLDN) 18.2-Positive, HER2-Negative, Locally Advanced Unresectable or Metastatic Gastric or Gastroesophageal Junction (GEJ) Adenocarcinoma

GLOW

ISN/Protocol 8951-CL-0302

Version 1.0

26 April 2018

IND 129598

EudraCT 2018-000519-26

Sponsor:

Astellas Pharma Global Development, Inc. (APGD)

1 Astellas Way

Northbrook, IL 60062

The information contained in this document is supplied as a background for clinical investigations. This document contains confidential information which is the intellectual property of Astellas. By accepting or reviewing this document, you agree to hold this information in confidence and not copy or disclose it to others or use it for unauthorized purposes except (1) as otherwise agreed to in writing; (2) where required by applicable law; (3) where disclosure is directly related to the care and safety of the research participant; and (4) where disclosure of such information is made to a member of the investigator's team who agrees to hold this information in confidence.

TABLE OF CONTENTS

I. SIGNATURES 9

II. CONTACT DETAILS OF KEY SPONSOR'S PERSONNEL 12

III. LIST OF ABBREVIATIONS AND DEFINITION OF KEY TERMS 13

IV. SYNOPSIS 17

V. FLOW CHART, STUDY SCHEMATICS AND SCHEDULE OF ASSESSMENTS 32

1 INTRODUCTION 43

1.1 Background 44

1.2 Nonclinical and Clinical Data 45

1.2.1 Nonclinical Data 45

1.2.2 Clinical Data 46

1.3 Summary of Key Safety Information for Study Drugs 47

1.4 Efficacy 48

1.4.1 Efficacy Results from Study GM-IMAB-001-02 (MONO) 48

1.4.2 Efficacy Results from Study GM-IMAB-001-03 (FAST) 48

1.4.3 Efficacy of CAPOX 49

1.5 Risk Benefit Assessment 49

2 STUDY OBJECTIVE(S), DESIGN, AND ENDPOINTS 50

2.1 Study Objective(s) 50

2.1.1 Primary Objectives 50

2.1.2 Secondary Objectives 50

2.1.3 Exploratory Objective 51

2.2 Study Design and Dose Rationale 51

2.2.1 Study Design 51

2.2.2 Dose Rationale 54

2.3 Endpoints 54

2.3.1 Primary Endpoints 54

2.3.2 Secondary Endpoints 54

2.3.3 Exploratory Endpoints 55

3 STUDY POPULATION 55

3.1 Selection of Study Population 55

3.2 Inclusion Criteria 55

3.3 Exclusion Criteria 57
4 IDENTIFICATION OF STUDY TREATMENT(S) 59
4.1 IMAB362 (Investigational Product) 59
4.2 Comparative Drug (Placebo) 60
4.3 CAPOX 60
4.4 Other Drug(s) 61
4.4.1 Antiemetic Premedication 61
4.5 Study Drug Handling 61
4.6 Blinding 62
4.6.1 Blinding Method 62
4.6.2 Confirmation of the Indistinguishability of the Study Drugs 62
4.6.3 Retention of the Assignment Schedule and Procedures for Treatment Code Breaking 62
4.6.4 Breaking the Treatment Code for Emergency 62
4.6.5 Breaking the Treatment Code by the Sponsor 63
4.7 Assignment and Allocation 63
5 TREATMENTS AND EVALUATION 64
5.1 Dosing and Administration of Study Drug(s) and Other Medication(s) 64
5.1.1 Dose/Dose Regimen and Administration Period 64
5.1.1.1 IMAB362/Placebo 64
5.1.1.2 Antiemetics 64
5.1.1.3 CAPOX 65
5.1.2 Study Treatment Dose Modifications, Delays And Interruptions 65
5.1.2.1 Increase or Reduction of IMAB362/Placebo 65
5.1.2.2 IMAB362/Placebo Interruption or Permanent Discontinuation 65
5.1.2.3 Guidelines for Infusion-Related Reactions for IMAB362/Placebo 68
5.1.2.4 CAPOX Dose Modification 69
5.1.2.5 CAPOX: Dose Modifications for Hematologic Toxicity 70
5.1.2.6 CAPOX: Dose Modification for Non-Hematologic Toxicity 73
5.1.2.7 Guidelines for Infusion-Related Reactions for Oxaliplatin 74
5.1.2.8 Oxaliplatin-Induced Neurotoxicity and Laryngopharyngeal Dysesthesia 75
5.1.2.9 Allergic Reaction to Oxaliplatin 76
5.1.2.10 Extravasation of Oxaliplatin 77

5.1.3 Treatment Compliance 77
5.1.4 Criteria for Continuation of Treatment 77
5.1.4.1 Discontinuation of CAPOX (both components) and Continuation of IMAB362/Placebo 77
5.1.4.2 Discontinuation of IMAB362/Placebo and Continued of CAPOX (one or both components) 77
5.1.4.3 If Both IMAB362/Placebo and CAPOX (both components) are discontinued 77
5.1.5 Previous and Concomitant Treatment (Medication and Non-Medication Therapy) 78
5.2 Demographics and Baseline Characteristics 79
5.2.1 Demographics 79
5.2.2 Medical History 79
5.2.3 Diagnosis of the Target Disease, Severity, and Duration of Disease 79
5.3 Efficacy Assessments 79
5.4 Safety Assessment 80
5.4.1 Vital Signs 80
5.4.2 Observation Period following IMAB362/Placebo Infusion 80
5.4.3 Laboratory Assessments 80
5.4.4 Physical Examination 82
5.4.5 Electrocardiogram 82
5.4.6 Performance Status 83
5.5 Adverse Events and Other Safety Aspects 83
5.5.1 Definition of Adverse Events 83
5.5.1.1 Abnormal Laboratory Findings 83
5.5.1.2 Potential Cases of Drug-Induced Liver Injury 84
5.5.1.3 Disease Progression and Study Endpoints 84
5.5.2 Definition of Serious Adverse Events 84
5.5.2.1 Always Serious Adverse Events 85
5.5.3 Criteria for Causal Relationship to Study Treatment 85
5.5.4 Criteria for Defining the Severity of an Adverse Event 87
5.5.5 Reporting of Serious Adverse Events 87
5.5.6 Follow-up of Adverse Events 89
5.5.7 Monitoring of Common Serious Adverse Events 89
5.5.8 Adverse Events of Special Interest 89
5.5.9 Special Situations 89

5.5.9.1 Pregnancy 90
5.5.9.2 Medication Error, Overdose and "Off-Label Use" 91
5.5.9.3 Misuse/Abuse 91
5.5.9.4 Occupational Exposure 91
5.5.9.5 Suspected Drug-Drug Interaction 91
5.5.10 Supply of New Information Affecting the Conduct of the Study 92
5.5.11 Urgent Safety Measures 92
5.5.12 Reporting Urgent Safety Measures 92
5.6 Test Drug Concentration 93
5.7 Other Measurements, Assessments or Methods 93
5.7.1 Biomarkers 93
5.7.2 Blood, Serum and Plasma Samples 93
5.7.3 Tumor Tissue Samples 94
5.7.4 Immunogenicity Assessment (Anti-drug Antibody) 94
5.7.5 Optional Samples for Banked PGx Sample Analysis 95
5.7.6 Electronic Clinical Outcome Assessments 95
5.7.6.1 Quality of Life Questionnaire C30 95
5.7.6.2 Oesophago-Gastric Module 25 plus STO22 Belching subscale 95
5.7.6.3 Global Pain 95
5.7.6.4 EuroQOL Five Dimensions Questionnaire 95
5.7.6.5 Health Resource Utilization 95
5.8 Total Amount of Blood 96
6 DISCONTINUATION 96
6.1 Discontinuation of Individual Subject(s) From Study Treatment 96
6.1.1 Lost to Follow Up 97
6.2 Discontinuation of the Site 97
6.3 Discontinuation of the Study 98
7 STATISTICAL METHODOLOGY 98
7.1 Sample Size 98
7.2 Analysis Sets 98
7.2.1 Full Analysis Set 98
7.2.2 Per Protocol Set 99
7.2.3 Safety Analysis Set 99
7.2.4 Pharmacokinetic Analysis Set (PKAS) 99

7.3 Demographics and Baseline Characteristics 99
7.3.1 Subject Disposition 99
7.3.2 Previous and Concomitant Medications 99
7.3.3 Medical History 99
7.4 Analysis of Efficacy 99
7.4.1 Analysis of Primary Endpoint 100
7.4.1.1 Primary Analysis 100
7.4.1.2 Sensitivity Analysis 100
7.4.1.3 Subgroup Analysis 100
7.4.2 Analysis of Secondary Endpoints 101
7.4.2.1 Overall Survival 101
7.4.2.2 Objective Response Rate 101
7.4.2.3 Duration of Response 101
7.4.2.4 Health-Related Quality of Life 102
7.4.3 Analysis of Exploratory Endpoints 102
7.4.3.1 Time to Progression 102
7.4.3.2 Progression Free Survival After Subsequent Therapy (PFS2) 102
7.4.3.3 Disease Control Rate 102
7.4.3.4 Biomarkers 102
7.4.3.5 Health Resource Utilization 102
7.5 Analysis of Safety 103
7.5.1 Adverse Events 103
7.5.2 Laboratory Assessments 103
7.5.3 Vital Signs 103
7.5.4 Routine 12-lead Electrocardiograms 103
7.5.5 Eastern Cooperative Oncology Group Performance Status 103
7.6 Analysis of Pharmacokinetics 103
7.6.2 Immunogenicity 104
7.7 Major Protocol Deviations and Other Analyses 104
7.8 Interim Analysis (and Early Discontinuation of the Clinical Study) 104
7.9 Handling of Missing Data, Outliers, Visit Windows, and Other Information 105
8 OPERATIONAL CONSIDERATIONS 105
8.1 Procedure for Clinical Study Quality Control 105
8.1.1 Data Collection 105

8.1.1.1 Collection of Data Via Electronic Source 105
8.1.1.2 Electronic Clinical Outcomes Assessment 106
8.2 Major Protocol Deviations 106
9 END OF TRIAL 107
10 STUDY ORGANIZATION 107
10.1 Independent Data Monitoring Committee 107
10.2 Other Study Organization 107
11 REFERENCES 108
12 APPENDICES 111
12.1 Ethical, Regulatory, and Study Oversight Considerations 111
12.1.1 Ethical Conduct of the Study 111
12.1.2 Institutional Review Board (IRB)/Independent Ethics Committee (IEC)/Competent Authorities (CA) 111
12.1.3 Protocol Amendment and/or Revision 111
12.1.4 Informed Consent of Subjects 112
12.1.4.1 Subject Information and Consent 112
12.1.4.2 Supply of New and Important Information Influencing the Subject’s Consent and Revision of the Written Information 112
12.1.5 Source Documents 113
12.1.6 Record Retention 113
12.1.7 Subject Confidentiality and Privacy 113
12.1.8 Arrangement for Use of Information and Publication of the Clinical Study 114
12.1.9 Insurance of Subjects and Others (UNIQUE TO JAPAN REGION/STUDIES ENROLLING SUBJECTS IN EU) 114
12.1.10 Signatory Investigator for Clinical Study Report 115
12.2 Procedure for Clinical Study Quality Control 116
12.2.1 Clinical Study Monitoring 116
12.2.2 Direct Access to Source Data/Documents 116
12.2.3 Data Management 116
12.2.4 QUALITY ASSURANCE 116
12.3 Contraception Requirements 117
12.4 Concomitant Medication Restrictions or Requirements 119
12.5 Liver Safety Monitoring and Assessment 121
12.6 Common Serious Adverse Events 124

12.7 Pharmacogenomic (PGx) Analysis With Banked Sample 125

12.8 Eastern Cooperative Oncology Group Performance Status Scale 127

13 COORDINATING INVESTIGATOR'S SIGNATURE 128

14 SPONSOR'S SIGNATURES 129

List of In-text Tables

Table 1 Schedule of Assessments 36
Table 2 Guidelines for IMAB362/Placebo Treatment Modification Due to Non-hematologic Toxicity 66
Table 3 Guidelines for IMAB362/Placebo Treatment Modification Due to Hematologic Toxicity 67
Table 4 Infusion-Related Reactions 69
Table 5 Dose Adjustment Levels for Oxaliplatin and Capecitabine 70
Table 6 CAPOX Dose Modification Due to Hematologic Toxicity 71
Table 7 CAPOX Dose Modification Due to Non-Hematologic Toxicity 74
Table 8 Oxaliplatin Dose Modification for Associated Neurotoxicity 75
Table 9 Comparison of the Symptoms and Treatment of Laryngopharyngeal Dysesthesia and Platinum Hypersensitivity Reactions 76

I. SIGNATURES

1. SPONSOR'S SIGNATURES

Required signatures (e.g. Protocol authors and contributors, etc.) are located in [Section 14 Sponsor's Signatures].

2. COORDINATING INVESTIGATOR'S SIGNATURE

The Coordinating Investigator's signature can be found in [Section 13 Coordinating Investigator's Signature]; located at the end of this document.

3. INVESTIGATOR'S SIGNATURE

A Phase 3, Global, Multi-Center, Double-Blind, Randomized, Efficacy Study of IMAB362 Plus CAPOX Compared with Placebo Plus CAPOX as First-line Treatment of Subjects with Claudin (CLDN)18.2-Positive, HER2-Negative, Locally Advanced Unresectable or Metastatic Gastric or Gastroesophageal Junction (GEJ) Adenocarcinoma

ISN/Protocol 8951-CL-0302

Version 1.0

26 April 2018

I have read all pages of this clinical study protocol for which Astellas is the sponsor. I agree to conduct the study as outlined in the protocol and to comply with all the terms and conditions set out therein. I confirm that I will conduct the study in accordance with International Council for Harmonisation (ICH) Good Clinical Practice (GCP) guidelines and applicable local regulations. I will also ensure that subinvestigator(s) and other relevant members of my staff have access to copies of this protocol and the ICH GCP guidelines to enable them to work in accordance with the provisions of these documents.
**Principal Investigator**:<br>Signature: --<br>Date (DD Mmm YYYY)<br>Printed Name: --<br>*<Insert name and qualification of the Investigator>*<br>Address: --<br>--

II. CONTACT DETAILS OF KEY SPONSOR'S PERSONNEL

**24h-Contact for Serious Adverse Events (SAEs)**<br><br>See [Section 5.5.5 Reporting of Serious Adverse Events] for SAE Fax Number and Email	**Please fax or email the SAE Worksheet to:**<br>Astellas Pharma Global Development, Inc.<br>Global Pharmacovigilance<br><br>**North America Fax number**: 888-396-3750<br>North America Alternate Fax number: 847-317-1241<br><br>**International Fax Number** : +44-800-471-5263<br>**Email: safety-us@astellas.com**<br><br>**Japan investigational sites:**<br>PAREXEL International<br>Global Monitoring Operations<br>Fax: 03-6888-2947
Medical Monitor/Study Physician:	PPD [redacted]<br>Astellas Pharma Global Development, Inc.<br>1 Astellas Way, Northbrook, Illinois 60062<br>PPD [redacted]
Clinical Research Contacts:	PPD [redacted]<br>Astellas Pharma Global Development, Inc.<br>1 Astellas Way, Northbrook, Illinois 60062<br>PPD [redacted]
Clinical Research Contacts Japan:	Corporate Name: Astellas Pharma Inc.<br>Location: 2-5-1, Nihonbashi-Honcho, Chuo-ku, Tokyo<br>PPD [redacted]

III. LIST OF ABBREVIATIONS AND DEFINITION OF KEY TERMS

List of Abbreviations

Abbreviations	Description of abbreviations
ADA	anti-drug antibody
ADCC	antibody-dependent cell-mediated cytotoxicity
AE	adverse event
ALP	alkaline phosphatase
ALT	alanine aminotransferase
ANC	absolute neutrophil count
APEBV	Astellas Pharma Europe B.V.
AST	aspartate aminotransferase
βhCG	beta human chorionic gonadotropin
C1D1	Cycle 1 Day 1
CAPOX	capecitabine and oxaliplatin
CDC	complement-dependent cytotoxicity
CE	Conformité Européene
CI	confidence interval
CLDN	claudin
CMH	Cochran-Mantel-Haenszel
CR	complete response
CRO	contract research organization
CT	computerized tomography
CTCAE	Common Terminology Criteria For Adverse Events
DCR	disease control rate
DOR	duration of response
ECG	electrocardiogram
ECL	electrochemiluminescense
eCOA	Electronic Clinical Outcomes Assessment
ECOG	Eastern Cooperative Oncology Group
eCRF	electronic case report form
EEA	European Economic Area
EORTC	European Organization for Research and Treatment of Cancer
EOX	epirubicin, oxaliplatin and capecitabine
EQ5D-5L	EuroQOL Five Dimensions Questionnaire 5L
EU	European Union
FAS	full analysis set
FIM	first-in-human

Abbreviations	Description of abbreviations
FFPE	formalin-fixed paraffin embedded
GCP	Good Clinical Practices
GE	gastroesophageal
GEJ	gastroesophageal junction
GMP	Good Manufacturing Practices
GP	Global Pain
Hgb	hemoglobin
HER2	human epidermal growth factor receptor 2
HIPAA	Health Insurance Portability and Accountability Act
HIV	human immunodeficiency virus
HRQoL	health-related quality of life
HRU	Health Resource Utilization
HSR	hypersensitivity reactions
IB	Investigator's Brochure
ICF	informed consent form
ICH	international council for harmonisation of technical requirements for registration of pharmaceuticals for human use
IDMC	independent data monitoring committee
IEC	independent ethics committee
IND	investigational new drug
IHC	immunohistochemistry
IMAB	ideal monoclonal antibody
INR	international normalized ratio
IRB	institutional review board
IRC	independent review committee
IRR	infusion-related reaction
IRT	interactive response technology
ISN	international study number
LA-CRF	liver abnormality case report form
LFT	liver function tests
mAbs	monoclonal antibodies
MRI	magnetic resonance imaging
NCCN	National Comprehensive Cancer Network
NCI	National Cancer Institute
NSAID	nonsteroidal anti-inflammatory drugs
ORR	objective response rate
OS	overall survival

**Abbreviations**	**Description of abbreviations**
PD	progressive disease
PFS	progression free survival
PFS2	progression free survival following subsequent anticancer treatment
PGx	pharmacogenomics
PKAS	pharmacokinetic analysis set
PPS	per protocol set
PR	partial response
PT	preferred term
QLQ-C30	Quality of Life Questionnaire - Core Questionnaire
QLQ-OG25	Quality of Life Questionnaire - Oesophago-Gastric Module 25
QTc	QT corrected
RECIST	Response Evaluation Criteria In Solid Tumors
RSI	reference safety information
(S)AE	serious adverse event and/or adverse event
SAE	serious adverse event
SAF	safety analysis set
SAP	statistical analysis plan
SD	stable disease
SOP	standard operating procedure
SPC	summary of product characteristics
SUSAR	suspected unexpected serious adverse reactions
TEAE	treatment-emergent adverse event
TTP	time to progression
ULN	upper limit of normal
VEGF	vascular endothelial growth factor
WOCBP	woman of childbearing potential

Definition of Key Study Terms

**Terms**	**Definition of terms**
Baseline	Assessments of subjects as they enter a study before they receive any treatment.
Endpoint	Variable that pertains to the efficacy or safety evaluations of a study.
Enroll	To register or enter a subject into a clinical study. NOTE: Once a subject has received the study drug or placebo, the clinical study protocol applies to the subject.
Intervention	The drug, device, therapy or process under investigation in a clinical study that is believed to have an effect on outcomes of interest in a study. (e.g., health-related quality of life, efficacy, safety and pharmacoeconomics).
Investigational period	Period of time where major interests of protocol objectives are observed, and where the test drug or comparative drug (sometimes without randomization) is usually given to a subject, and continues until the last assessment after completing administration of the test drug or comparative drug.
Post investigational period	Period of time after the last assessment of the protocol. Follow-up observations for sustained adverse events and/or survival are done in this period.
Randomization	The process of assigning study subjects to treatment or control groups using an element of chance to determine assignments in order to reduce bias.
Screening	A process of active consideration of potential subjects for enrollment in a study.
Screen failure	Potential subject who did not meet 1 or more criteria required for participation in a study.
Screening period	Period of time before entering the investigational period, usually from the time when a subject signs the consent until just before the test drug or comparative drug (sometimes without randomization) is given to a subject.
Study period	Period of time from the first site initiation date to the last site completing the study.
Study Treatment	Includes IMAB362/placebo and both components of CAPOX
Variable	Any entity that varies; any attribute, phenomenon or event that can have different qualitative or quantitative values.

IV. SYNOPSIS

**Date and Version No of Protocol Synopsis:**	26 April 2018, Version 1.0
**Sponsor:**<br>Astellas Pharma Global Development Inc. (APGD)	**Protocol Number:**<br>8951-CL-0302
**Name of Study Drug:**<br>IMAB362	**Phase of Development:**<br>Phase 3

Title of Study:

A Phase 3, Global, Multi-Center, Double-Blind, Randomized, Efficacy Study of IMAB362 Plus CAPOX Compared with Placebo Plus CAPOX as First-line Treatment of Subjects with Claudin (CLDN)18.2-Positive, HER2-Negative, Locally Advanced Unresectable or Metastatic Gastric or Gastroesophageal Junction (GEJ) Adenocarcinoma

Planned Study Period:

From 4Q2018 to 3Q2022

Study Objective(s):

Primary

- To evaluate the efficacy of IMAB362 plus CAPOX compared with placebo plus CAPOX (as first-line treatment) as measured by Progression Free Survival (PFS) in subjects with Claudin (CLDN) 18.2-positive, human epidermal growth factor receptor 2 (HER2)-negative locally advanced unresectable or metastatic gastric and gastroesophageal junction (GEJ) adenocarcinoma

Secondary

- To evaluate efficacy as measured by Overall Survival (OS) as a key secondary objective
- To evaluate efficacy as measured by Objective Response Rate (ORR)
- To evaluate efficacy as measured by Duration of Response (DOR)
- To evaluate safety and tolerability of IMAB362
- To evaluate health related quality of life (HRQoL) using the parameters as measured by European Organization for Research and Treatment of Cancer (EORTC) Quality of Life Questionnaire (QLQ)-C30 and QLQ-OG25 plus STO22 Belching subscale, Global Pain (GP) and the EuroQOL Five Dimensions Questionnaire 5L (EQ5D-5L) questionnaires
- To evaluate the pharmacokinetics of IMAB362
- To evaluate the immunogenicity profile of IMAB362

Exploratory

- To evaluate efficacy as measured by Time to Progression (TTP)
- To evaluate PFS following subsequent anticancer treatment (PFS2)
- To evaluate Disease Control Rate (DCR)
- To evaluate potential genomic and/or other biomarkers that may correlate with treatment outcome to IMAB362 and CAPOX.
- To evaluate Health Resource Utilization (HRU)

Planned Total Number of Study Centers and Location(s):

Approximately 125 centers globally

Study Population:

Subjects with locally advanced unresectable or metastatic gastric or GEJ adenocarcinoma whose tumors are CLDN18.2-positive, HER2-negative and who have not been previously treated for metastatic disease with chemotherapy (first-line).

For the purpose of this study, CLDN18.2-positive is defined as CLDN18.2 expression in ≥ 75% of tumor cells demonstrating moderate to strong membranous staining as determined by central immunohistochemistry (IHC) testing.

Number of Subjects to be Enrolled/Randomized:

Approximately 500 subjects

Study Design Overview:

This global, multicenter, double-blind, 1:1 randomized, phase 3 study will evaluate efficacy of IMAB362 plus CAPOX versus placebo plus CAPOX as first-line treatment in subjects with CLDN18.2-positive, HER2-negative locally advanced unresectable or metastatic gastric and GEJ adenocarcinoma.

PFS as assessed by the Independent Review Committee (IRC) is the primary outcome. Secondary outcomes include OS, ORR, DOR, safety and tolerability, HRQoL, pharmacokinetics and the immunogenicity profile of IMAB362. Exploratory outcomes include TTP, PFS2, DCR, biomarkers, and HRU.

Approximately 500 subjects will be randomized 1:1 into 1 of 2 treatment arms:

- Arm A (IMAB362 in combination with CAPOX chemotherapy)
- Arm B (placebo in combination with CAPOX chemotherapy)

Randomization of subjects will be stratified by the following factors:

- Region (Asia vs Non-Asia)
- Number of Metastatic Sites (0 to 2 vs ≥ 3)
- Prior Gastrectomy (Yes or No)

Screening:

The Screening period is 45 days. Re-screening may be allowed upon discussion with the medical monitor.

Formalin fixed paraffin embedded (FFPE) tumor tissue will be collected for central testing to determine CLDN18.2 and HER2 status. Confirmation of CLDN18.2-positive and HER2-negative status is to be obtained prior to subjects proceeding to any other Screening procedures.

- Archival tumor tissue is preferred.
  - A minimum of 1 FFPE tumor tissue block (preferred) OR a minimum of 15 FFPE unstained slides are required. If slides are submitted, the slides should be freshly cut from the FFPE block within the time frame described in the laboratory manual.
  - If local HER2 results are already available from local testing, a minimum of 12 FFPE unstained slides are required to be submitted to the central laboratory.
- If the specimen is insufficient or unavailable, a biopsy may be performed to obtain tumor sample.
  - Sponsor pre-approval is required when the sole purpose of the biopsy procedure is to assess eligibility for this study.
  - If the required number of slides cannot be provided, the sponsor or designee should be contacted for further guidance.

Treatment Period:

Subjects will be treated with either IMAB362 (Arm A) or placebo (Arm B) on Day 1 of each cycle until the subject meets study treatment discontinuation criteria. For all study treatments, a cycle is defined as 21 days.

Subjects will also receive up to 8 cycles of CAPOX treatment. Oxaliplatin is administered on day 1 of each cycle, whereas capecitabine is taken twice daily on days 1 through 14. After 8 cycles, subjects may continue to receive capecitabine taken twice daily on days 1 through 14 of each cycle at the investigator's discretion until the subject meets study treatment discontinuation criteria.

Radiologic imaging will be evaluated every 9 weeks (± 7 days) counting from cycle 1/day 1 (C1D1) for the first 54 weeks and every 12 weeks (± 7 days) thereafter until subject develops radiological disease progression per Response Evaluation Criteria In Solid Tumors (RECIST 1.1) by IRC or starts other systemic anticancer treatment, whichever occurs earlier.

If a subject discontinues CAPOX (or oxaliplatin only) due to any reason other than disease progression as confirmed by IRC, they may continue on IMAB362/placebo at the discretion of the investigator provided that all of the following have been met:

- the subject completed at least 2 cycles of CAPOX treatment;
- the subject received no other chemotherapy during the study treatment period;
- in the investigator's opinion, the subject continues to derive clinical benefit with acceptable toxicity.

Subjects should continue to follow the Study Treatment Period schedule of assessments.

Safety Assessments:

Safety will be evaluated based on adverse events (AEs), vital signs, electrocardiograms (ECGs), physical exams, Eastern Cooperative Oncology Group (ECOG) performance status and laboratory assessments. Severity of AEs and laboratory abnormalities will be assessed based on National Cancer Institute-Common Terminology Criteria For Adverse Events (NCI-CTCAE).

Radiologic Imaging and Independent Review Committee:

Radiologic imaging will be evaluated at Screening (within 28 days prior to C1D1) and every 9 weeks (±7 days) counting from C1D1 for the first 54 weeks and then every 12 weeks (±7 days) thereafter until subject develops radiological disease progression per RECIST 1.1 by IRC or starts other systemic anticancer treatment, whichever comes earlier.

All measurable disease must be documented at Screening and re-assessed at each subsequent radiologic evaluation. Imaging will include computerized tomography (CT) scans with contrast of the thorax, abdomen, and pelvis. If CT scan with contrast is medically not feasible, magnetic resonance imaging (MRI) may be used for imaging. Bone scans (or focal X-ray) or brain imaging should be performed if metastatic disease in bone or brain is suspected, respectively. The same mode of imaging should be utilized throughout the study unless medical necessity requires a change. All imaging will be sent to the sponsor designated facility within 7 days of scanning for the blinded IRC assessment of radiological tumor response based on RECIST 1.1 [Eisenhauer et al, 2009]. The investigator should make every effort to immediately submit radiologic assessments for IRC review when progressive disease (PD) is suspected.

Study Treatment Discontinuation and Safety Follow-up Visits:

Following discontinuation from IMAB362/placebo, subjects will have an IMAB362/placebo Study Treatment Discontinuation Visit within 7 days after the decision to discontinue, and 30-day and 90-day Safety Follow-up Visits after their last dose of IMAB362/placebo.

Additionally, if CAPOX (both components) is discontinued on a different day than IMAB362/placebo, subjects will also have a Study Treatment Discontinuation Visit within 7 days after the decision to discontinue, and 30-day and 90-day Safety Follow-up Visits after the last dose of CAPOX (both components). The CAPOX 30-day and 90-day Safety Follow-up Visits may be conducted by telephone if the subject is unable to visit the study site and will require contact for AE collection only.

Post-treatment Follow-up Period (for PFS):

If a subject discontinues all study treatments (IMAB362/placebo and both components of CAPOX) prior to disease progression per IRC, the subject will enter the Post-treatment Follow-up Period and continue to undergo scheduled imaging assessments every 9 weeks (±7 days) (or every 12 weeks [±7 days] if subject has been on study over 54 weeks) until radiologic disease progression (i.e., PFS event) per IRC, or until the subject starts any other anticancer treatment, whichever occurs earlier.

If study treatments (IMAB362/placebo and both components of CAPOX) are discontinued due to disease progression (PFS event), the subject will enter the Long-term and Survival Follow-up Period.

Long-term Follow-up Period (for PFS2) and Survival Follow-up (for OS) Period:

Following disease progression on first-line treatment or start of any other anticancer treatment, subjects will be followed in the Long-term and Survival Follow-up Period per institutional guidelines, but not less than every 12 weeks. Subsequent anticancer treatment details, progression status and survival status will be collected until PD following subsequent anticancer therapy (PFS2) is documented, or the subject starts another systemic anticancer treatment, whichever occurs earlier. Radiologic imaging for PFS2 will be done per local standard of care and read locally. Subjects will continue to be followed for survival status (OS) in the Survival Follow-up Period until death (from any cause).

All postprogression details including subsequent anticancer treatment and date and site of progression will be recorded on the electronic case report form (eCRF). Subject contact by phone or other remote method is sufficient during Long-term and Survival Follow-up. Additional follow-up contacts may be required per sponsor request for analysis purposes.

Biomarkers and Other Sampling:

Samples for pharmacokinetics, immunogenicity and biomarkers, as well as FFPE tumor tissue specimens will be collected. Optional pharmacogenomics and post-progression tumor samples may be collected for those subjects who sign a separate informed consent form (ICF).

Electronic Clinical Outcomes Assessments (HRQoL and HRU):

HRQoL and HRU will be assessed during the visit prior to any study treatment(s) administration or physician assessment. Assessments will be collected at Screening, every 3 weeks, at study treatment discontinuation and 30 and 90 days post IMAB362/placebo treatment. HRQoL will be measured by EORTC QLQ-C30, QLQ-OG25 plus STO22 Belching subscale, GP and the EQ5D-5L.

Independent Data Monitoring Committee and Independent Data Analysis Center:

An Independent Data Monitoring Committee (IDMC) will be established and will monitor the ongoing benefit-risk status of study treatment in an unblinded fashion per a pre-defined IDMC charter. The first IDMC meeting will be approximately 6 weeks after the 40th subject enrolled has completed or discontinued cycle 2 (6 weeks) and meetings will be conducted thereafter, as defined in the IDMC charter.

An Independent Data Analysis Center will conduct an interim analysis of OS at the same time as the final PFS analysis, which will occur when approximately 344 PFS events have occurred. This analysis will be utilized by the IDMC to recommend whether the study should be stopped earlier than planned if IMAB362 in combination with CAPOX has a favorable outcome compared with placebo in combination with CAPOX. If the OS interim analysis demonstrates a highly more favorable outcome for IMAB362 in combination with CAPOX, the study may be stopped for success. However, any subject continuing to derive clinical benefit from IMAB362/placebo in combination with CAPOX, as assessed by the investigator, will be allowed to continue treatment.

Inclusion/Exclusion Criteria:

Inclusion Criteria:

Waivers to the inclusion criteria will **NOT** be allowed.

<u>*General Criteria*</u>*:*

1. Institutional Review Board (IRB)/Independent Ethics Committee (IEC) approved written informed consent and privacy language as per national regulations (e.g., Health Insurance Portability and Accountability Act [HIPAA] Authorization for US sites) must be obtained from the subject or legally authorized representative (if applicable) prior to any study-related procedures.
2. Subject is considered an adult (e.g., ≥ 18 years of age in the US) according to local regulation at the time of signing the informed consent.
3. A female subject is eligible to participate if she is not pregnant (negative serum pregnancy test at screening; female subjects with elevated serum beta human chorionic gonadotropin (βhCG) and a demonstrated non-pregnant status through additional testing are eligible) and at least 1 of the following conditions applies:
   - Not a woman of childbearing potential (WOCBP) as defined in [Appendix 12.3 Contraception Requirements]

   OR
   - WOCBP who agrees to follow the contraceptive guidance as defined in [Appendix 12.3 Contraception Requirements] throughout the treatment period and for 6 months after the final study treatment administration
4. Female subject must agree not to breastfeed starting at screening and throughout the study period, and for 6 months after the final study treatment administration.
5. Female subject must not donate ova starting at screening and throughout the study period, and for 6 months after the final study treatment administration.
6. A male subject with female partner(s) of childbearing potential:
   - must agree to use contraception as detailed in [Appendix 12.3 Contraception Requirements] during the treatment period and for 6 months after the final study treatment administration.
7. A male subject must not donate sperm during the treatment period and for 6 months after the final study treatment administration.
8. Male subject with a pregnant or breastfeeding partner(s) must agree to remain abstinent or use a condom for the duration of the pregnancy or time partner is breastfeeding throughout the study period and for 6 months after the final study treatment administration.
9. Subject agrees not to participate in another interventional study while receiving study drug in present study.

Disease Specific Criteria:

10. Subject has histologically confirmed diagnosis of Gastric or GEJ adenocarcinoma.
11. Subject has radiologically confirmed locally advanced unresectable or metastatic disease within 28 days prior to the first dose of study treatment.
12. Subject has measurable disease according to RECIST 1.1 within 28 days prior to the first dose of study treatment. For subjects with only 1 measurable lesion and prior radiotherapy, the lesion must be outside the field of prior radiotherapy or must have documented progression following radiation therapy.
13. Subject's tumor expresses CLDN18.2 in ≥ 75% of tumor cells demonstrating moderate to strong membranous staining as determined by central IHC testing.
14. Subject has a HER2-negative tumor as determined by local or central testing on a gastric or GEJ tumor specimen.

Physical or Laboratory Findings:

15. Subject has ECOG performance status 0 or 1.
16. Subject has predicted life expectancy ≥ 12 weeks in the opinion of the investigator.
17. Subject must meet all of the following criteria based on the centrally analyzed laboratory tests within 14 days prior to the first dose of study treatment. In case of multiple central laboratory data within this period, the most recent data should be used to determine eligibility.
    - Hemoglobin (Hb) ≥ 9 g/dl. NOTE: subject must not have received any growth factor or blood transfusions within 14 days prior to the hematology values obtained at screening. Subjects requiring transfusions to meet eligibility criteria are not eligible.
    - Absolute Neutrophil Count (ANC) ≥ 1.5x10^9/L
    - Platelets ≥ 100x10^9/L
    - Albumin ≥ 2.5 g/dL
    - Total Bilirubin ≤ 1.5 x upper limit of normal (ULN)
    - Aspartate aminotransferase (AST) and alanine aminotransferase (ALT) ≤ 2.5 x ULN without liver metastases (or ≤ 5 x ULN if liver metastases are present)
    - Either serum creatinine ≤ 1.5 x ULN or estimated glomerular filtration rate ≥ 50 mL/min/1.73 m^2

Prothrombin time/international normalized ratio (PT/INR) and partial thromboplastin time (PTT) ≤ 1.5 x ULN (except for subjects receiving anticoagulation therapy)

Exclusion Criteria:

Waivers to the exclusion criteria will **NOT** be allowed.

Subject who meets any of the following exclusion criteria prior to enrollment is not eligible for enrollment:

Prohibited Treatment or Therapies:

1. Subject has received prior systemic chemotherapy for locally advanced unresectable or metastatic gastric or GEJ adenocarcinoma. However, subject may have received either neo-adjuvant or adjuvant chemotherapy as long as it was completed at least 6 months prior to the first dose of study treatment.
2. Subject has received radiotherapy for locally advanced unresectable or metastatic gastric or GEJ adenocarcinoma unless the radiotherapy was completed > 28 days prior to the first dose of study treatment. Subject who received palliative radiotherapy to peripheral bone metastases ≥ 14 days prior to first dose of study treatment and has recovered from all acute toxicities is eligible.

3. Subject has received treatment with herbal medications or other treatments that have known antitumor activity within 28 days prior to first dose of study treatment.
4. Subject has received systemic immunosuppressive therapy, including systemic corticosteroids within 14 days prior to first dose of study treatment. Subject using a physiologic replacement dose of hydrocortisone or its equivalent (defined as up to 30 mg per day of hydrocortisone or up to 10 mg per day of prednisone) is eligible.
5. Subject has received other investigational agents or devices within 28 days prior to first dose of study treatment.

Medical History or Concurrent Disease:

6. Subject has prior severe allergic reaction or intolerance to a monoclonal antibody, including humanized or chimeric antibodies.
7. Subject has known immediate or delayed hypersensitivity, intolerance or contraindication to any component of study treatment.
8. Subject has prior severe allergic reaction or intolerance to any component of CAPOX.
9. Subject has known dihydropyrimidine dehydrogenase (DPD) deficiency.(NOTE: Subjects with low or absent DPD should not be treated with capecitabine due to increased risk for severe, life-threatening, or fatal adverse reactions.)
10. Subject has gastric outlet syndrome or persistent/recurrent vomiting.
11. Subject had recent gastric bleeding and/or is symptomatic with proven gastric ulcers that excludes the subject from participation per investigator judgement.
12. Subject has a known history of a positive test for human immunodeficiency virus (HIV) infection or known active hepatitis B (positive HBs Ag) or C infection. For subjects who are negative for HBs Ag, but HBc Ab positive, an HB DNA test will be performed and if positive, the subject will be excluded. Subjects with positive serology but negative hepatitis C virus (HCV) RNA test results are eligible.
13. Subject has an active autoimmune disease that has required systemic treatment within the past 2 years.
14. Subject has active infection requiring systemic therapy that has not completely resolved within 14 days prior to first dose of study treatment.
15. Subject has significant cardiovascular disease, including:
    - Congestive heart failure (defined as New York Heart Association [NYHA] Class III or IV), myocardial infarction, unstable angina, coronary angioplasty, coronary stenting, coronary artery bypass graft, cerebrovascular accident (CVA), or hypertensive crisis within 6 months prior to administration of first dose of study treatment;
    - History of clinically significant ventricular arrhythmias (i.e., sustained ventricular tachycardia, ventricular fibrillation, or Torsades de Pointes);
    - QTc interval > 450 msec for male subjects; QTc interval > 470 msec for female subjects;
    - Cardiac arrhythmias requiring anti-arrhythmic medications (Subjects with rate controlled atrial fibrillation for > 1 month prior to first dose of study treatment are eligible.)
16. Subject has known central nervous system (CNS) metastases and/or carcinomatous meningitis.
17. Subject has known peripheral sensory neuropathy > grade 1 unless the absence of deep tendon reflexes is the sole neurological abnormality.

18. Subject has had a major surgical procedure and has not completely recovered within 28 days prior to the first dose of study treatment.
19. Subject has psychiatric illness or social situations that would preclude study compliance, per investigator judgement.
20. Subject has another malignancy for which treatment is required per investigator's clinical judgment
21. Subject has any concurrent disease, infection, or co-morbid condition that interferes with the ability of the subject to participate in the study, which places the subject at undue risk or complicates the interpretation of data in the opinion of the investigator.

Investigational Product(s):

**IMAB362/Placebo:**	IMAB362: The investigational product, IMAB362, is a sterile lyophilized powder preparation with the chimeric monoclonal antibody IMAB362 as the active pharmaceutical ingredient.<br>Each vial contains 105 mg of IMAB362 and has to be reconstituted with 5.0 mL sterile water for injection (WFI), to a concentration of 20 mg/mL. Further dilution with sterile 0.9% Sodium Chloride Injection, to a final concentration of 2 mg/mL is required.<br>Placebo: Sites should use 0.9 % Sodium Chloride Injection as placebo.
**Dose(s):**	800 mg/m² loading dose of IMAB362 at C1D1 followed by subsequent doses of 600 mg/m² every 3 weeks.
**Dosing Schedule:**	Subjects will be treated with either IMAB362 (Arm A) or placebo (Arm B) on day 1 of each cycle until the subject meets study treatment discontinuation criteria.<br>IMAB362/placebo to be administered after antiemetic premedication but prior to CAPOX.
**Mode of Administration:**	Intravenous infusion of IMAB362/placebo as a 2-hour infusion.<br>Intravenous infusion may be interrupted or slowed down to manage toxicity. **It is recommended that IMAB362/placebo infusion not exceed 6 hours from start of infusion.**

Other Product(s):

**CAPOX:**	Subjects will receive CAPOX treatment until IRC confirmed disease progression or a total of 8 cycles (each cycle is defined as 3 weeks = 21 days). Oxaliplatin is administered on day 1 of each cycle, whereas capecitabine is taken twice daily on days 1 through 14.<br>After 8 cycles of CAPOX, subjects may continue to receive capecitabine twice daily on days 1 through 14 of each cycle at the investigator's discretion until the subject meets study treatment discontinuation criteria.<br>CAPOX is to be administered after IMAB362/placebo infusion.
**Oxaliplatin:**	130 mg/m^2 intravenous infusion on day 1 of each cycle over 2 hours (or longer per institutional standard of care) for up to 8 cycles.
**Capecitabine**	Administered orally at 1000 mg/m^2 twice daily (bid) (total daily dose is 2000 mg/m^2) on days 1 through 14 of each cycle
**<u>Antiemetic Pre-medications</u>**	Prophylactic antiemetics should be administered according to institutional standard of care, published guidelines and the respective product package insert(s).<br>(NOTE: Subjects receiving IMAB362 do not need to be premedicated for prevention of IRRs; however, subjects should be closely monitored for IRRs to facilitate early identification and management.)<br>• All antiemetic premedication should be given at minimum 30 minutes prior to IMAB362/placebo treatment.<br>• It is recommended that the prophylactic antiemetic regimen include (but is not limited to) the following agents:<br>o NK-1 receptor blockers<br>o 5-HT3 receptor blockers*<br>*To minimize the risk of Torsades de Pointes, avoid use in subjects with congenital long QT syndrome and administer with caution to subjects who have or may develop QTc prolongation.<br>**<u>Corticosteroids:</u>**<br>• The impact of corticosteroids on the potential efficacy of IMAB362 is not known. Therefore, consideration should be given to avoid or minimize the use of corticosteroids as a prophylactic antiemetic, if possible.<br>• For a subject's <u>first dose</u> of IMAB362/placebo, it is recommended that the prophylactic use of corticosteroids <u>be avoided</u>.

Concomitant Medication Restrictions or Requirements:

Prohibited Concomitant Treatment

The following are strictly prohibited:

- Sorivudine or analogs (during capecitabine treatment)
- Systemic immunosuppressive agents:
  - Concurrent systemic immunosuppressive therapy, in particular systemic corticosteroids, should be stopped 14 days prior to first dose of study treatment.
  - Subjects are allowed to use a physiologic replacement dose of hydrocortisone or its equivalent (defined as up to 30 mg per day of hydrocortisone or up to 10 mg per day of prednisone).
- Live vaccines should be avoided during the treatment period in which subject is receiving capecitabine and up to 6 months after final capecitabine dose.
- Other systemic chemotherapy, immunotherapy, radiotherapy, herbal medications or other treatments intended for antitumor activity. Palliative radiotherapy for peripheral bone metastases is allowed.
- Investigational products or therapy other than IMAB362.

Cautionary Concomitant Treatment

Considerations should be given to avoid or minimize the use of the following concomitant medications, if possible, during IMAB362/placebo treatment:

- Systemic corticosteroids, because their impact on the potential efficacy of IMAB362 is not known.
  - Systemic corticosteroids should be avoided or minimized while subject is on study treatment unless required for management of an emergent medical condition (e.g., severe nausea/vomiting or hypersensitivity reaction).
  - For a subject's first dose of IMAB362/placebo, it is recommended that the prophylactic use of corticosteroids be avoided.
  - Inhaled, intranasal and topically applied steroids are allowed.
- Avoid the class of 5-HT3 blockers in subjects with congenital long QT syndrome. Administer these drugs with caution in subjects who have or may develop QTc prolongation.
- Nonsteroidal anti-inflammatory drugs (NSAIDs) because of the potential to cause gastric ulcers and covert bleeding.
  - In such cases where NSAID use is necessary, the use of NSAIDs with lower gastric ulcerogenic potential is preferred and concomitant gastric protection with proton pump inhibitors is recommended.

The following should be avoided or used with caution and closely monitored during CAPOX administration:

- CytochromeP450 (CYP) 2C9 substrates (Subjects taking coumarin-derivative anticoagulants concomitantly with capecitabine should have PT/INR monitored regularly and anticoagulant dose adjusted accordingly).
- Anti-epileptic medications (e.g. phenobarbital, phenytoin and primidone)

Duration of Treatment:

Subjects will receive IMAB362/placebo until IRC confirmed disease progression, toxicity requiring study treatment cessation, start of another anticancer treatment, or other treatment discontinuation criteria are met.

Subjects will also receive up to 8 cycles of CAPOX followed by continued use of capecitabine (based on investigator's judgement) beyond 8 cycles until treatment discontinuation criteria are met.

Study Treatment Discontinuation Criteria:

A subject who enrolled in the study and for whom study treatment (IMAB362/placebo and both components of CAPOX) is permanently discontinued for any reason will be assessed as having met study treatment discontinuation criteria.

As overall survival is the key secondary end-point of the study, all subjects will be followed for survival after meeting study treatment discontinuation criteria unless the subject withdraws consent or is considered lost to follow-up after repeated attempts to contact or if the sponsor discontinues the study.

A subject is free to withdraw from the study treatment and/or study for any reason and at any time without giving reason for doing so and without penalty or prejudice. The investigator is also free to terminate a subject's involvement in the study at any time if the subject's clinical condition warrants it.

The subject will be discontinued from study treatment (IMAB362/placebo and both components of CAPOX) if any of the following occur:

- Investigator determines it is in the subject’s best interest to discontinue study treatment
- Subject develops radiological disease progression per RECIST 1.1 criteria based on assessment by IRC.
  - If the investigator believes that the subject is continuing to derive clinical benefit (asymptomatic and/or without worsening of performance status or overall health) from study treatment, and an increase in tumor burden is not likely to affect vital organ function, the subject may remain on study treatment until an additional radiologic assessment is completed (≤ 9 weeks from previous radiologic assessment).
    - If the additional radiologic assessment by IRC indicates PD per RECIST 1.1, then the subject must be discontinued from study treatment.
    - If the additional radiologic assessment by IRC does not confirm the initial assessment of PD, the subject may continue to receive study treatment.
- Subject develops clinical progression per investigator assessment and radiologic assessment is not medically feasible to confirm radiologic progression due to the subject’s condition.
- Subject starts another systemic chemotherapy, immunotherapy, radiotherapy or other treatment intended for antitumor activity.
- Subject starts other investigational agent or device.
- Subject develops unacceptable toxicity.
- Subject has a delay of study treatment (IMAB362/placebo and both components CAPOX) for > 28 days.
- Any clinical AE, laboratory abnormality, or inter-current illness, in the opinion of the investigator, indicates continued treatment is not in the best interest of the subject
- Female subject becomes pregnant.
- Significant deviation from the protocol or eligibility criteria as determined by the sponsor.
- Subject declines further treatment.
- Subject is lost to follow-up despite reasonable efforts by the investigator to locate the subject.
- Subject is noncompliant with the protocol based on investigator or medical monitor assessment.

Note, if a subject discontinues both components of CAPOX and IMAB362/placebo due to any reason other than IRC confirmed disease progression (and is not receiving any other anticancer therapy), the subject must be followed according to the protocol-specified radiologic assessment schedule until radiological disease progression per RECIST 1.1 criteria is confirmed by IRC assessment.

Study Discontinuation Criteria

All subjects should remain in the study through the Survival Follow-up Period (OS is a key secondary study endpoint). A subject will be discontinued from the Post-treatment, Long-term and Survival Follow-up Periods if any of the following occur:

- Subject specifically withdraws consent for any further contact with him/her or persons previously authorized by the participant to provide this information
- Subject is lost to follow-up despite reasonable efforts by the investigator to locate the subject
- Death (from any cause)
- Study termination by the sponsor

Endpoints for Evaluation:

Primary:

- PFS, defined as the time from the date of randomization until the date of radiological PD (per RECIST 1.1 by IRC) or death from any cause, whichever is earliest

Secondary:

- OS, defined as the time from the date of randomization until the date of death from any cause
- ORR, defined as the proportion of subjects who have a best overall response of complete response (CR) or partial response (PR) as assessed by IRC per RECIST 1.1
- DOR, defined as the time from the date of the first response (CR/PR) until the date of PD as assessed by IRC per RECIST 1.1 or date of death from any cause, whichever is earliest
- Safety and tolerability, as measured by AEs, laboratory test results, vital signs, ECGs and ECOG performance status
- HRQoL, as collected via EORTC QLQ-C30, QLQ-OG25 plus STO22 Belching subscale, GP, and EQ5D-5L questionnaires
- Pharmacokinetics of IMAB362, C_{trough}
- Immunogenicity of IMAB362 as measured by the frequency of antidrug-antibody (ADA) positive subjects

Exploratory:

- TTP, defined as the time from the date of randomization until the date of PD as assessed by IRC per RECIST 1.1.
- PFS2, defined as the time from the date of randomization until the date of PD (per investigator) following subsequent anticancer therapy, death from any cause or start of any other anticancer therapy, whichever is earliest
- DCR, defined as the proportion of subjects who have a best overall response of CR, PR or SD as assessed by IRC per RECIST 1.1
- Potential genomic and/or other exploratory biomarkers that may be related to treatment outcome of IMAB362
- HRU, defined as health resource utilization

Statistical Methods:

Sample Size Justification:

One interim analysis and 1 final analysis are planned for OS, while only 1 final analysis is planned for PFS. The OS interim analysis will occur at the same time of final PFS analysis (after pre-specified number of PFS events) and final OS analysis will be performed after the pre-specified number of OS events are observed. The O'Brien-Fleming boundaries as implemented by Lan-DeMets (1983) alpha spending method (East®) will be used for the OS interim and OS final analyses. All statistical tests of treatment effects will be conducted at the 1-sided 0.025 level of significance unless otherwise specified.

Approximately 500 subjects will be randomized in a 1:1 ratio to receive IMAB362 in combination with CAPOX chemotherapy (Arm A) or placebo in combination with CAPOX chemotherapy (Arm B).

The planned 344 PFS events during the study will provide 96% power to detect a difference in PFS between Arm A (IMAB362+CAPOX) with the assumption of 9 months median PFS time and Arm B (placebo+CAPOX) with the assumption of 6 months median PFS time (hazard ratio = 0.67) at the overall 1-sided 0.025 significance level. Similarly, the planned 386 OS events during the study will provide 80% power to detect a difference in OS between Arm A (IMAB362+CAPOX) with the assumption of 14.7 months median survival time and Arm B (placebo+CAPOX) with the assumption of 11 months median OS time (hazard ratio = 0.75) at the overall 1-sided 0.025 significance level.

Analysis Populations:

- The full analysis set (FAS) will include all subjects who are randomized to 1 of the treatment arms. Subjects will be analyzed according to the treatment arm to which they were randomized to. The FAS will be used for description of baseline characteristics and all efficacy analyses.
- The per-protocol set (PPS) will consist of the subset of the FAS who do not meet PPS exclusion criteria. These criteria are intended to capture relevant nonadherence to the protocol and will be defined in the SAP. The sensitivity analyses for the primary (PFS) and key secondary endpoints (OS) will be performed on the PPS.
- The safety analysis set (SAF) will contain all subjects who received at least 1 dose of any study drug (IMAB362/placebo/CAPOX). The SAF will be used for all safety analyses. Subjects will be analyzed according to the actual treatment they received.
- The pharmacokinetic analysis set (PKAS) will consist of the subset of the SAF for which at least one IMAB362 concentration measurement is available. Additional subjects may be excluded from the PKAS at the discretion of the pharmacokineticist. The PKAS will be used for description of pharmacokinetic data.

Efficacy Analyses:

All efficacy analyses will be performed using FAS. In addition, PPS will be used for the primary and key secondary efficacy analyses.

Primary Efficacy Endpoint:

The primary efficacy endpoint of PFS will be analyzed using the stratified Log Rank Test with stratification factors to be specified in the SAP.

The hypothesis testing on the primary analysis will be performed at an overall 1-sided 0.025 significance level to test the null hypothesis that PFS is not prolonged in Arm A compared to Arm B versus the alternative hypothesis that PFS is prolonged in Arm A compared to Arm B.

Estimates of the treatment effect will be expressed as Hazard Ratio using a stratified Cox model, including 95% Confidence Interval.

The sensitivity analysis for the primary efficacy endpoint will also be performed.

Secondary Endpoints:

The key secondary efficacy endpoint of OS will be analyzed using the stratified Log Rank Test with the same strata used in the analysis of PFS. To maintain the overall Type I error rate at the 0.025 significance level, the hypothesis testing on OS will be performed only if the null hypothesis on the primary analysis is rejected at the overall 1-sided 0.025 significance level. The sensitivity analysis for the key secondary endpoint will be performed on the PPS.

The secondary efficacy endpoint of ORR will be analyzed using the Cochran-Mantel-Haenszel test to control for the same strata used in the analysis of PFS and OS. The secondary efficacy endpoint of DOR will be analyzed similarly to PFS and OS.

The secondary HRQoL endpoints collected via the EORTC QLQ-C30 and QLQ-OG25 plus STO22 Belching subscale, GP and EQ-5D-5L will be analyzed with summary of change from baseline over time through the end of CAPOX treatment and inferential methods. Detailed analysis of HRQoL endpoints will be provided in the statistical analysis plan.

Exploratory Endpoints: TTP and PFS2 will be analyzed in a similar way as PFS. However, in TTP analysis, deaths are not counted as events; rather, deaths are censored. DCR will be analyzed similarly as ORR. The HRU variables will be summarized by treatment arm.

Safety Analyses:

The safety evaluation will be based on AEs, clinical laboratory tests, vital signs, ECG and ECOG status. Descriptive statistics will be used to summarize safety data. All safety data will be summarized by treatment received (SAF).

All summaries of AEs will include only treatment-emergent events unless otherwise stated. AEs will be categorized by SOC and preferred term using MedDRA and will be graded for severity according to the NCI-CTCAE.

Pharmacokinetics:

Descriptive statistics will be used to summarize serum concentrations of IMAB362. The potential relationship between IMAB362 immunogenicity and IMAB362 pharmacokinetics, efficacy, and safety profile will be assessed. Additional model-based analyses may be performed and reported separately.

Biomarkers:

Biomarkers will be summarized graphically or descriptively, and summary statistics may be tabulated. Associations between biomarkers and clinical (e.g., efficacy, safety or pharmacodynamics, or pharmacokinetics) measures may be performed on subjects who have sufficient baseline and on-study treatment measurements to provide interpretable results for specific parameters.

Interim Analyses:

To evaluate whether IMAB362 + CAPOX (Arm A) is beneficial compared to placebo + CAPOX (Arm B) while the study is ongoing, a formal OS interim analysis is planned when the final PFS analysis occurs with the pre-specified number of PFS events. A group sequential design using the O'Brien-Fleming type alpha-spending function [Lan & DeMets, 1983] for efficacy will be utilized to control the overall 1-sided 0.025 significance level (East®) for the OS analyses.

The IDMC may recommend terminating the study for favorable results at the formal efficacy interim analysis using OS. In the case of favorable results, the 1-sided significance level for superiority is 0.0074 for the interim OS analysis and 0.0228 for the final OS analysis. If the 1-sided P value of the interim analysis is less than 0.0074, the IDMC may recommend terminating the study for success. If the study is not stopped after the interim analysis, a final OS analysis will occur after 100% of the planned death events have been observed.

Details for the interim analyses, monitoring subject safety, enrollment rates and event (PFS/death) rates will be contained in the IDMC Charter. Recommendations regarding study conduct will be made by the IDMC based on their assessment of these rates.

V. FLOW CHART, STUDY SCHEMATICS AND SCHEDULE OF ASSESSMENTS

a. Flow Chart

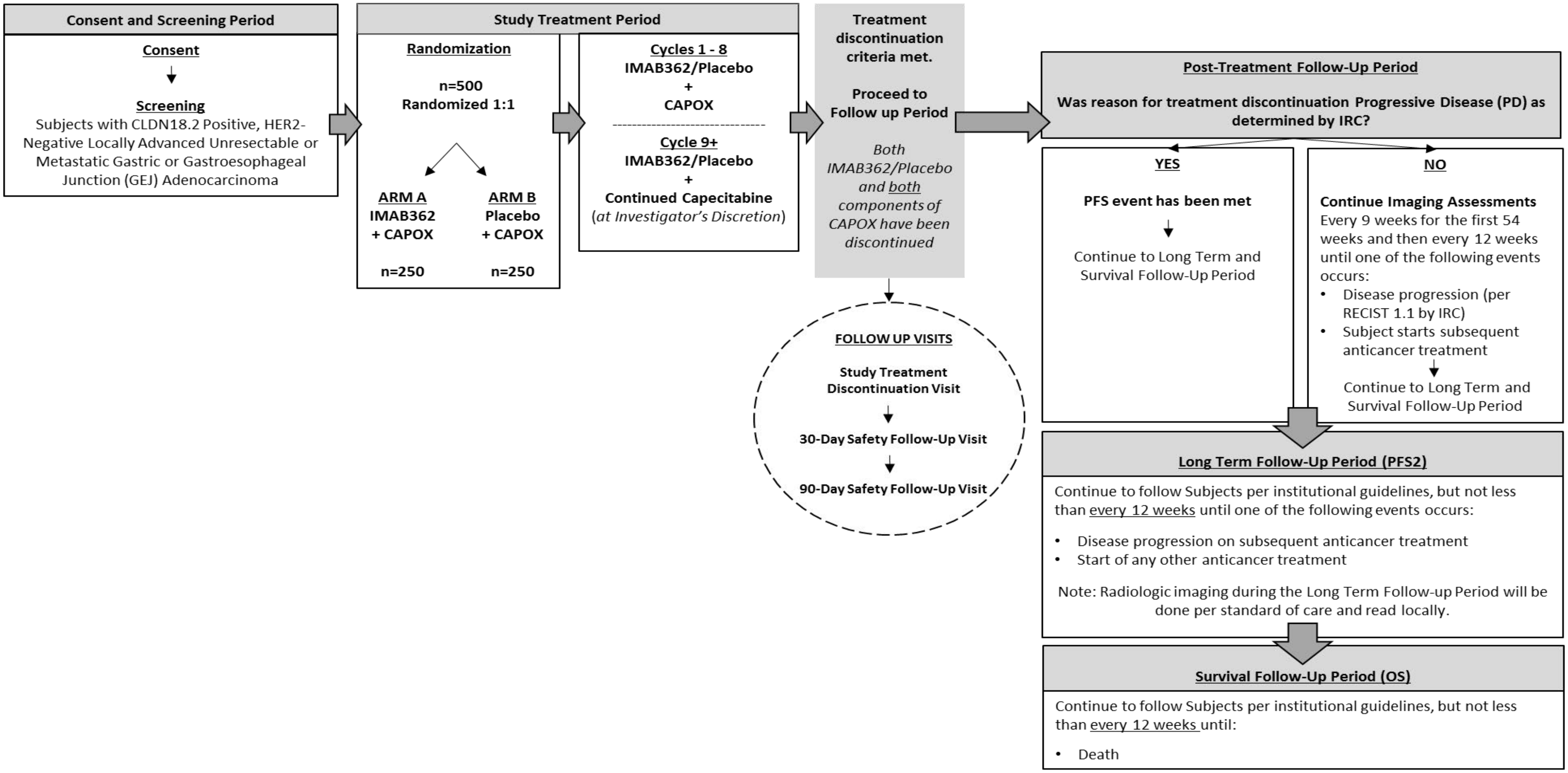

CLDN: claudin; HER2: human epidermal growth factor receptor 2; IRC: independent review committee; OS: overall survival; PFS: progression free survival; PFS2: progression free survival following subsequent anticancer treatment; RECIST: Response Evaluation Criteria In Solid Tumors

b. Study Treatment Period Dosing Schedule (1 Cycle = 21 Days)

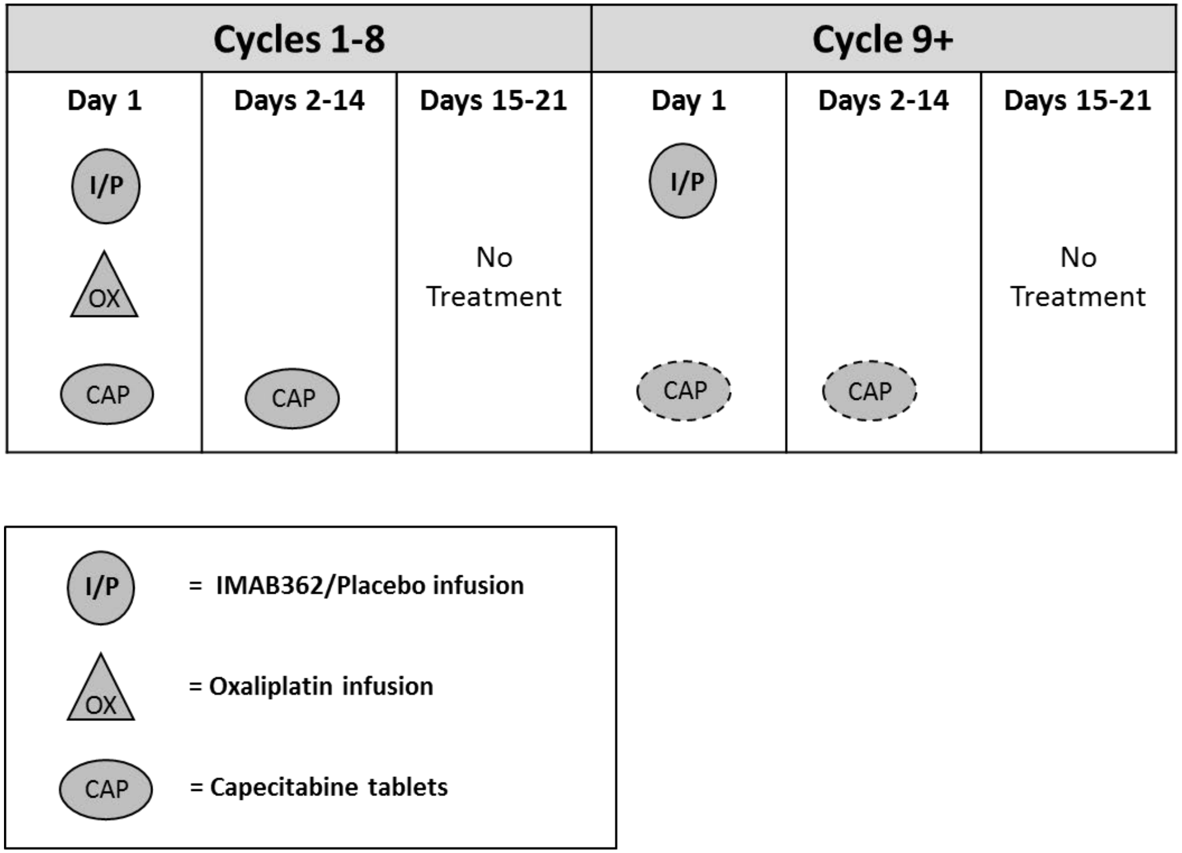

Cycles 9+: Subjects may continue on Capecitabine at Investigator's discretion

Dosing Schematics:

a. Combination IMAB362/Placebo and CAPOX Dosing (Cycle 1 to Cycle 8)

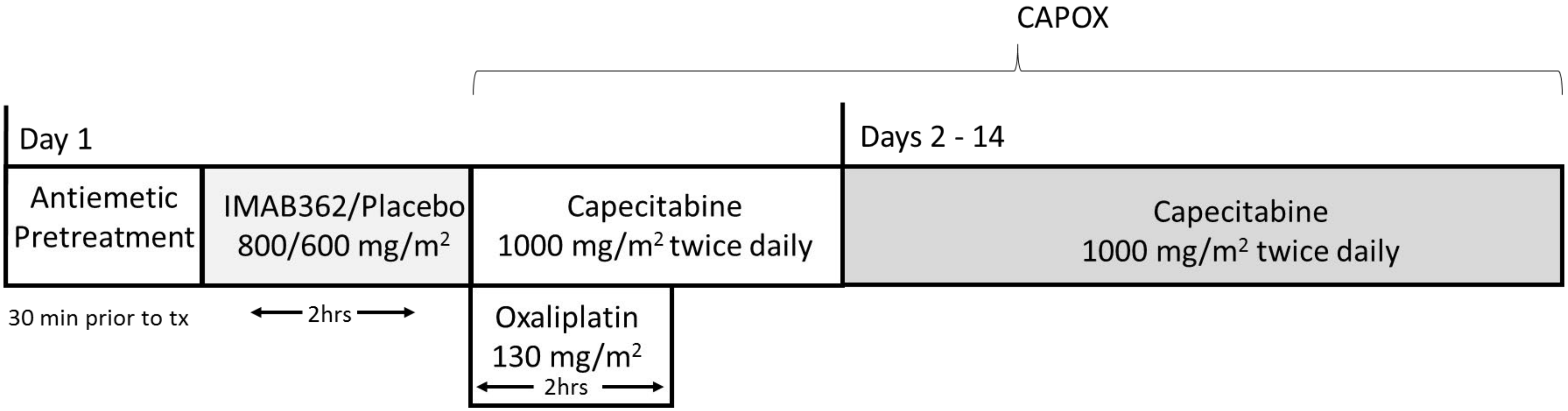

Note: Standard timeframes described above may be modified as per Section 5.1.2, Study Treatment Dose Modifications, Delays and Interruptions

b. IMAB362/Placebo Only Dosing (Cycle 9 and onwards)*

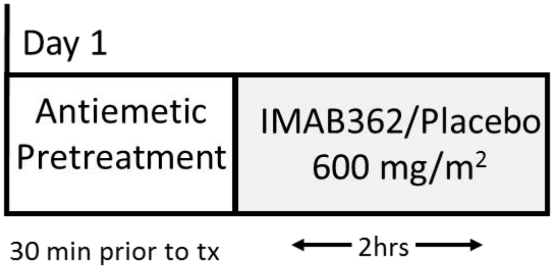

Note: Standard timeframes described above may be modified as per Section 5.1.2, Study Treatment Dose Modifications, Delays and Interruptions
*Cycles 9+: Subjects may continue on Capecitabine at Investigator's discretion

Table 1 Schedule of Assessments

VISIT	Screening[1]	Study Treatment Period (Each Cycle = 21 Days)						Follow-up Period				
		**Cycles 1-8** IMAB362/Placebo + CAPOX			**Cycle 9+** IMAB362/Placebo + Capecitabine (at investigator discretion)			Study Treatment Discontinuation Visit[18]	30-Day Safety Follow-up Visit(s)[19]	90-Day Safety Follow-up Visit(s)[20]	Post-treatment Follow-up Period [21]	Long-term and Survival Follow-up Periods [22]
**Day**		1	2-14	15-21	1	2-14	15-21					
**Visit Window (calendar days)**	-45 to -1	+7*	(no visit)	(no visit)	+7	(no visit)	(no visit)	+7	+7	±7	±7	±14
Informed Consent	X											
CLDN18.2 Tumor Sample[2]	X											
HER2 Tumor Sample[2]	X											
Biopsy (if applicable)[2]	X											
Medical and Disease History	X											
Confirmation of Inclusion/Exclusion Criteria[3]	X	X										
Randomization[4]		X										
**Treatments**												
Antiemetic Pretreatment[5]		X			X							
IMAB362/Placebo[6]		X			X							
Postinfusion Observation Period[7]		X			X							
Oxaliplatin (CAPOX[8])		X										
Capecitabine (CAPOX[8])		X	X		X	X						
**Safety Assessments**												
Physical Examination[9]	X	X			X			X	X			
Weight[9]	X	X			X			X	X			
Vital Signs[10]	X	X			X			X	X			
ECOG Performance Status[9]	X	X			X			X				
12-lead ECG[11]	X	If clinically indicated and/or per local requirements						X	X			
**Image Assessment[12]**	X[1]	Every 9 weeks ±7 days for first 54 weeks and then every 12 weeks ±7 days thereafter										
Table continued on next page												

VISIT	Screening[1]	Study Treatment Period (Each Cycle = 21 Days)						Follow-up Period				
		**Cycles 1-8** IMAB362/Placebo + CAPOX			**Cycle 9+** IMAB362/Placebo + Capecitabine (at investigator discretion)			Study Treatment Discontinuation Visit[18]	30-Day Safety Follow-up Visit(s)[19]	90-Day Safety Follow-up Visit(s)[20]	Post-treatment Follow-up Period [21]	Long-term and Survival Follow-up Periods [22]
**Day**		1	2-14	15-21	1	2-14	15-21					
**Visit Window (calendar days)**	-45 to -1	+7*	(no visit)	(no visit)	+7	(no visit)	(no visit)	+7	+7	±7	±7	±14
**Subject Contact**												X
**Laboratory Tests**												
Biochemistry[13]	X	X			X			X	X			
TSH and T4[13]	X	If clinically indicated						X				
Hematology[13]	X	X			X			X	X			
Coagulation Parameters (PT, PTT and INR)[14]	X	If clinically indicated										
Urinalysis[13]	X	X			X			X	X			
Serum Pregnancy Test[15]	X	If clinically indicated and/or per local requirements										
Urine Pregnancy Test[16]		X			X			X	X			
**Electronic Clinical Outcomes Assessments (eCOA)**												
HRQoL[16]	X	X			X			X	X	X		
Health Resource Utilization (HRU)[17]		X			X			X	X	X		
**Sampling**												
Pharmacokinetics of IMAB362 (Serum)[23]		X			X				X	X		
Antidrug-Antibodies (ADA) for Immunogenicity[24]		X			X				X	X		
Genetic Immune Polymorphisms (Whole Blood)[25]		X										
Exploratory Biomarkers (Serum)[26]		X						X				
Exploratory Biomarkers (Plasma)[26]		X						X				
Whole Blood Sample for PGx (optional)[27]		X										
Post-progression Tumor Sample (optional)[28]								X				
**Concomitant Medication**	X	X			X			X	X			
**AE[29]**	X	X			X			X	X	X		

Footnotes appear on next page

ADA: antidrug antibody; AE: adverse event; βhCG: beta human chorionic gonadotropin; C1D1: Cycle 1 Day 1; CAPOX: capecitabine and oxaliplatin; CLDN: claudin;CT: computerized tomography; eCOA: electronic Clinical Outcomes Assessment; ECG: electrocardiogram; ECOG: European Cooperative Oncology Group; eCRF: electronic case report form; FFPE: formalin fixed paraffin embedded; HER2: human epidermal growth factor receptor 2; HRQoL: health-related quality of life; HRU: Health Resource Utilization; ICF: informed consent form: INR: international normalized ratio; IRC: independent review committee; IRR: infusion-related reaction; IV: intravenous; MRI: magnetic resonance imaging; OS: overall survival; PD: progressive disease; PFS: progression free survival; PFS2: progression free survival following subsequent anticancer treatment; PGx: pharmacogenomics; PT: prothrombin time; PTT: partial thromboplastin time; RECIST: Response Evaluation Criteria In Solid Tumors; SAE: serious adverse event; T4: thyroxine; TSH: thyroid stimulating hormone

* +7 day visit window does not apply to C1D1.

1. Screening: The Screening period is 45 days. Re-screening may be allowed once and upon discussion with the medical monitor. Central laboratory results must be used to confirm eligibility. The screening labs used to determine eligibility should be collected within 14 days prior to C1D1. In situations where laboratory results are outside of the permitted range, the investigator may opt to retest the subject and subsequent within range central lab screening results may be used to confirm eligibility. In case of multiple central laboratory data within the Screening period, the most recent data should be used to confirm eligibility. Subjects requiring transfusions to meet eligibility criteria are not eligible. Radiologic imaging used to confirm eligibility must be conducted within 28 days prior to C1D1.
2. CLDN18.2 and HER2 Testing: FFPE tumor tissue will be collected for central testing to determine CLDN18.2 and HER2 status. Confirmation of CLDN18.2-positive and HER2-negative status should be obtained prior to subjects proceeding to any other Screening procedures. Archival tumor tissue is preferred. A minimum of 1 FFPE tumor tissue block (preferred) OR a minimum of 15 FFPE unstained slides are required. If slides are submitted, the slides should be freshly cut from the FFPE block within the time frame described in the laboratory manual. If local HER2 results are already available from local testing, a minimum of 12 FFPE unstained slides are required to be submitted to the central lab. If the specimen is insufficient or unavailable, a biopsy may be performed to obtain tumor sample. Sponsor pre-approval is required when the sole purpose of the biopsy procedure is to assess eligibility for this study. If the required number of slides cannot be provided, the sponsor or designee should be contacted for further guidance. See [Section 5.7.3 Tumor Tissue Samples].
3. Confirmation of Inclusion/Exclusion Criteria must be completed on C1D1.
4. Randomization: After confirmation of eligibility, the unblinded pharmacist/designee will contact the interactive response technology (IRT) system in order to determine the randomly assigned treatment. Randomization may be performed prior to C1D1; however, the time between the beginning of IMAB362/placebo reconstitution and the beginning of IMAB362/placebo infusion must be within 4 hours at controlled room temperature (15°C to 25°C). If the infusion cannot be initiated within the 4 hours, the infusion bag must be stored at 2°C to 8°C and infusion must be initiated within 24 hours from the beginning of reconstitution.
5. Antiemetic Pretreatment: Prophylactic antiemetics should be given according to institutional standard of care, published guidelines and the respective product package insert(s). All antiemetic premedication should be given at minimum 30 minutes prior to treatment. For further details, see [Section 5.1.1.2 Antiemetics].
6. IMAB362/placebo will be administered as a 2-hour intravenous infusion every 3 weeks starting on C1D1. IMAB362/placebo should be administered prior to CAPOX. It is recommended that IMAB362/placebo infusion not exceed 6 hours from start of infusion. For further details, see [Section 5.1.1.1 IMAB362/Placebo].

Footnotes continued on next page

7. Postinfusion Observation Period: Following the first dose of IMAB362/placebo on C1D1, the subject must be observed for 2 hours post IMAB362/placebo infusion. The postinfusion observation period can be conducted during the CAPOX administration. If AEs are observed during this time, subsequent IMAB362/placebo infusion times should be extended and subjects should continue to be observed for 2 hours post IMAB362/placebo infusion. If the subject does not develop any AEs, the subject should be observed for 1 hour post-infusion for their subsequent IMAB362/placebo infusions. The subject should be instructed to notify site personnel if they develop any AEs during this observation time period. In the event of grade 3 or 4 infusion-related reactions (IRRs) post IMAB362/placebo infusion, samples for cytokine/chemokine and tryptase should be collected. See Observation Period following IMAB362/placebo [Section 5.4.2] for further details.
8. CAPOX is a combination of oxaliplatin intravenous infusion and capecitabine tablets and will be administered starting at C1D1 for up to 8 cycles. See [Section 5.1.1.3].
9. Physical Exam: should include height (at Screening only), weight and ECOG performance status. A full physical exam is required at Screening. The physical exam only needs to be repeated on C1D1 if clinically significant changes from Screening are observed (in the opinion of the investigator). Targeted (symptom driven) physical exams should be conducted every 3 weeks on Day 1 of each cycle. For further details, see [Section 5.4.4 Physical Examination].
10. Vital signs (pulse, blood pressure, temperature) should be taken during every visit at the following time points (see [Section 5.4.1 Vital Signs]):
    - Pre-dose at every visit
    - C1D1 and C2D1: Every 15 (±5) minutes during IMAB362/placebo infusion
    - Subsequent IMAB362/placebo infusions: every 30 (±10) minutes during IMAB362/placebo infusions if the subject did not develop any AEs during the Postinfusion Observation Period of cycle 1.
    - Every 30 (±10) and 60 (±10) minutes post IMAB362/placebo infusion during the Postinfusion Observation Period (for 1 or 2 hours. See footnote 7)
    - Unscheduled if clinically indicated
11. ECGs: a single ECG will be performed at Screening, the IMAB362/placebo Discontinuation Visit, the 30-Day Follow-up Visit, and if clinically indicated or per local requirements. ECGs will be locally assessed. When collected on the same day, ECG should be collected prior to pharmacokinetic samples. For further details, see [Section 5.4.5 Electrocardiogram].
12. Imaging Assessments: Radiologic imaging will be evaluated at Screening (must be conducted within 28 days prior to C1D1) and every 9 weeks (± 7 days) counting from C1D1 for the first 54 weeks and then every 12 weeks (± 7 days) thereafter until subject develops radiological disease progression per RECIST 1.1 by IRC or starts other systemic anticancer treatment, whichever comes earlier. Imaging will include CT scans with contrast of the thorax, abdomen, and pelvis. If CT scan with contrast is medically not feasible, MRI may be used for imaging. Bone scans (or focal X-ray) or brain imaging should be performed if metastatic disease in bone or brain is suspected, respectively. The same mode of imaging should be utilized throughout the study unless medical necessity requires a change. All imaging will be sent to the sponsor designated facility within 7 days for the blinded independent central assessment of radiological tumor response based on RECIST 1.1. The investigator should make every effort to immediately submit radiologic assessments for IRC review when PD is suspected. See [Section 5.3 Efficacy Assessments].

Footnotes continued on next page

13. Laboratory Assessments: See [Section 5.4.3 Laboratory Assessments] for list of laboratory assessments. Laboratory tests must be sent to the central laboratory for analysis. Central laboratory results must be used to confirm eligibility. The screening labs used to determine eligibility should be collected within 14 days prior to C1D1. In situations where central laboratory results are outside of the permitted range, the investigator may opt to retest the subject and subsequent within range screening results may be used to confirm eligibility. In case of multiple laboratory data within the Screening period, the most recent central laboratory data should be used to confirm eligibility. Subjects requiring transfusions to meet eligibility criteria are not eligible. Laboratory tests will be reviewed by the investigator prior to each infusion. In the event that the central laboratory results are not available in time for treatment decisions, local certified laboratory tests may be used. Holidays and weekends should be taken into account when scheduling these blood draws. Additional assessments may be done centrally or locally to monitor AEs or as clinically indicated. Clinical significance of out-of-range laboratory findings is to be determined and documented by the investigator/sub-investigator who is a qualified physician.
14. Coagulation (PT, PTT and INR): Coagulation tests should be done at Screening and during study treatment period if clinically indicated. Central lab results must be used to confirm eligibility. Ongoing evaluation should be continued for subjects who are receiving therapeutic anticoagulation according to local standard of care. See [Section 5.4.3 Laboratory Assessments].
15. Serum Pregnancy Test: Serum pregnancy tests will be collected for female subjects of childbearing potential only. Serum pregnancy tests collected at Screening, during study treatment period and if clinically indicated or per local requirements. (Note: For Screening, subjects with elevated serum βHCG and a demonstrated non-pregnant status through additional testing are eligible.) Central laboratory results must be used to confirm eligibility.
16. Urine Pregnancy Test: Urine pregnancy tests will be collected for female subjects of childbearing potential only. Urine pregnancy tests to be completed during the treatment period every 3 weeks on day 1 of each cycle and at the IMAB362/placebo Study Treatment Discontinuation and 30-Day Safety Follow-up Visits.
17. HRQoL and HRU questionnaires: eCOA questionnaires are to be administered on Day 1 of each cycle before any antiemetic or drug treatment or other scheduled assessments are conducted and before the disease status is discussed with the subject.
18. Study Treatment Discontinuation Visit (End of Study Treatment): The Study Treatment Discontinuation Visit will take place ≤ 7 days following the decision to discontinue study treatment (IMAB362/placebo and CAPOX [both components]). If IMAB362/placebo and CAPOX (both components) are discontinued on a different day, subjects will have separate Study Treatment Discontinuation Visits following each treatment's discontinuation.
19. 30-Day Safety Follow-up Visit: A 30-Day Safety Follow-up Visit should occur 30 days after the last dose of IMAB362/placebo and will include the assessments as shown the in the Schedule of Assessments above. A 30-Day Safety Follow-up Visit should occur 30 days after the last dose of CAPOX (both components) and may be conducted by phone if the subject is unable to visit the site and will require contact for AE collection only.
20. 90-Day Safety Follow-up Visits: A 90-Day Safety Follow-up Visit should occur 90 days after the last dose of IMAB362/placebo and will include the assessments as shown the in the Schedule of Assessments above. A 90-Day Safety Follow-up Visit should occur 90 days after the last dose of CAPOX (both components) and may be conducted by phone if the subject is unable to visit the site and will require contact for SAE collection only.
21. Post-treatment Follow-up: if a subject discontinues all study treatments (IMAB362/placebo and both components of CAPOX) prior to IRC confirmed disease progression, the subject will enter the Post-treatment Follow-up Period and continue to undergo imaging assessments every 9 weeks (±7 days) (or every 12 weeks [±7 days] if subjects has been on study over 54 weeks) until radiologic disease progression (i.e., PFS) or the subject starts subsequent anticancer treatment, whichever occurs earlier. If study treatments (IMAB362/placebo and both components of CAPOX) are discontinued due to PD, the subject will enter the Long-term and Survival Follow-up Period.

Footnotes continued on next page

22. Long-term and Survival Follow-up Period: Following disease progression on first-line treatment or start of subsequent anticancer treatment, subjects will be followed in the Long-term and Survival Follow-up Period per institutional guidelines, but not less than every 12 weeks. Radiologic imaging will be done per standard of care and read locally until PFS2 is documented. Survival Follow-up Period will continue until death (from any cause). All post-progression details including subsequent anticancer treatment and date and site of progression will be recorded on the eCRF. Subject contact by phone or other remote methods is sufficient during Long-term and Survival Follow-up.
23. Pharmacokinetics: Serum samples for IMAB362/placebo will be taken at the below timepoints. The date and time of each blood sample collection will be recorded to the nearest minute.
    - Cycle 1 Day 1: End of IMAB362/placebo infusion
    - Cycle 2 Day 1: Predose
    - Cycle 3 Day 1: End of IMAB362/placebo infusion
    - Predose on Day 1 of Cycles 5, 9, 13 and 17
    - IMAB362/placebo 30-Day Safety Follow-up visit
    - IMAB362/placebo 90-Day Safety Follow-up visit
    - Unscheduled pharmacokinetic blood samples may be taken at any time during the study to evaluate drug exposure following a safety event

    Pharmacokinetic Sampling Window:
    - Predose: within 60 minutes prior to dosing
    - End of Infusion: within 10 minutes after the end of the infusion
24. ADA: Blood samples (Serum) for ADA will be taken at the below timepoints.
    - Cycle 1 Day 1: Predose
    - Cycle 2 Day 1: Predose
    - Predose on Day 1 of Cycles 5, 9, 13 and 17
    - IMAB362/placebo 30-Day Safety Follow-up visit
    - IMAB362/placebo 90-Day Safety Follow-up visit

    ADA Sampling Window: Predose: within 60 minutes prior to dosing
25. Genetic Immune Polymorphism: Whole blood sample taken at C1D1.
26. Exploratory Biomarker (Serum and Plasma) samples should be taken at the below timepoints:
    - Cycle 1 Day 1: Predose
    - Cycle 2 Day 1: Predose
    - Cycle 3 Day 1: Predose
    - Cycle 4 Day 1: Predose
    - Cycle 5 Day 1: Predose
    - Cycle 6 Day 1: Predose
    - Cycle 8 Day 1: Predose
    - IMAB362/placebo Study Treatment Discontinuation Visit
27. Optional PGx: for subjects who signed a separate ICF, an optional whole blood sample for PGx for exploratory biomarker analysis may be collected prior to first study drug administration.

Footnotes continued on next page

28. Optional Post-Progression Tumor Sample: for subjects who signed a separate ICF, an optional post-progression tumor sample for exploratory biomarker analysis may be collected following IRC confirmation of disease progression and prior to initiation of subsequent anticancer therapy.
29. AEs: AEs will be collected from the time of signed informed consent through 30 days following the last dose of study treatment or until initiation of a new anticancer treatment, whichever comes first. SAEs (regardless of causality) will be collected from the time of informed consent through 90 days following the last dose of study treatment or until initiation of a subsequent anticancer treatment, whichever comes first. See [Section 5.5.5 Reporting of Serious Adverse Events].

1 INTRODUCTION

Gastric and gastroesophageal junction (GEJ) cancers are among the malignancies with the highest unmet medical need. Gastric cancer-related mortality is the fourth leading cause of cancer death worldwide, even if its incidence has decreased over the last 50 years in different regions of the world [Cancer Fact Sheet, 2018; Amiri et al, 2011]. On the other hand, the incidence of subjects with GEJ adenocarcinoma has increased in recent decades, coinciding with a shift in histological type and primary tumor location [Waddell et al, 2013; Sahin et al, 2008].

In 2017, an estimated 723,100 people died worldwide from gastric cancer [Lederman, 2017]. The overall 5-year survival rate for gastric and GEJ cancers is averaging 20% in the US and Europe, despite aggressive standard treatments, which are also associated with substantial side effects [Pennathur et al, 2013; Sahin et al, 2008].

There is no single standard, globally accepted first-line reference chemotherapeutic regimen for advanced gastric cancer. In the US and Europe, the current standard of care consists of fluoropyrimidine with platinum-based combination chemotherapy regimens with or without a third agent such as docetaxel or epirubicin [National Comprehensive Cancer Network (NCCN), 2017; Waddell et al, 2013; Pasini et al, 2011]. Subjects in this study will receive CAPOX (a combination of capecitabine and oxaliplatin), which is a globally accepted standard-of-care treatment for subjects with locally advanced unresectable or metastatic gastric or gastroesophageal (GE) cancer. The safety of this combination regimen is well-documented [Kim et al, 2012].

The lack of a major benefit from the various newer generation combination chemotherapy regimens for these cancers has stimulated research into the use of targeted agents such as monoclonal antibodies (mAbs). Two mAbs, trastuzumab and ramucirumab, have received approval for treatment of gastric cancer. Trastuzumab selectively binds the extracellular domain of human epidermal growth factor receptor 2 (HER2), which is overexpressed in approximately 20% to 30% of gastric tumors [Bang et al, 2009], and ramucirumab specifically binds vascular endothelial growth factor (VEGF) receptor 2 and blocks binding of VEGF receptor ligands VEGF-A, VEGF-C and VEGF-D. Trastuzumab is approved for treatment of HER2 overexpressing metastatic gastric or GEJ adenocarcinoma while ramucirumab is approved as a single agent or in combination with paclitaxel, for treatment of advanced gastric or GEJ adenocarcinoma, with disease progression on or after prior fluoropyrimidine-or platinum-containing chemotherapy [CYRAMZA Prescribing Information, 2017; HERCEPTIN Prescribing Information, 2016]. These agents prolonged median overall survival (OS) by 4 or fewer months when given alone or in combination with chemotherapy compared with standard of care cytotoxic chemotherapy [Fuchs et al, 2014; Wilke et al, 2014; Ohtsu et al, 2011; Bang et al, 2010].

Approximately 70% to 80% of patients with metastatic or advanced unresectable gastric and GEJ adenocarcinoma in the first line setting have tumors that are HER2 negative and are not treatable with trastuzumab. These patients have an expected median survival of approximately 1 year [Shah, 2017]. Therefore, a significant unmet medical need exists for

the first-line treatment of patients with HER2 negative locally advanced or metastatic unresectable gastric and GEJ cancers. IMAB362 is being developed with the goal of addressing this unmet medical need.

1.1 Background

IMAB362 is a genetically engineered, highly purified chimeric (mouse/human IgG1) antibody directed against the tight junction molecule Claudin 18.2 (CLDN18.2). The target is a member of the claudin family of more than 20 structurally related proteins that are involved in the formation of tight junctions in epithelia and endothelia [Niimi et al, 2001]. Tight junctions, together with adherens junctions and desmosomes, form the apical junctional complex in epithelial and endothelial cellular sheets. Adherens junctions and desmosomes are responsible for the mechanical adhesion between adjacent cells, whereas tight junctions are essential for the tight sealing of the cellular sheets forming a luminary barrier and controlling the paracellular ion flux.

One hallmark of cancer is that tight junction proteins lose their organization in multimeric structures, promoting loss of cell polarity, cohesion and differentiation [Mori et al, 1999]. Because of this, epitopes of tight junction molecules, which are shielded in the normal epithelia, might become exposed and accessible to antibodies such as IMAB362 after malignant transformation.

CLDN18.2 is a 27.8 kDa protein with 4 membrane-spanning domains and 2 small extracellular loops [Gunzel & Yu, 2013; Sahin et al, 2008]. IMAB362 recognizes the first extracellular domain of CLDN18.2 with high affinity and specificity. IMAB362 does not bind to any other claudin family member including the closely related splice variant 1 of Claudin 18 (CLDN18.1).

CLDN18.2 is a highly cell type specific differentiation antigen that is expressed by differentiated gastric mucosa cells in the pit and base regions of gastric glands. Moreover, CLDN18.2 is not detectable in any other normal cell type of the human body either at transcript level or as protein. This highly selective tissue distribution pattern results in CLDN18.2 expression being strictly confined to a subpopulation of gastric epithelial cells in normal tissue [Sahin et al, 2008].

CLDN18.2 is expressed in a diversity of human cancers and is the dominant isoform in GE and pancreatic cancer [Lee et al, 2011]. The expression of CLDN18.2 is retained upon malignant transformation of gastric epithelia and is present in 81% of primary gastric adenocarcinomas. CLDN18.2 expression is frequently detected in diffuse and in intestinal gastric cancers. The CLDN18.2 protein is also localized in lymph node metastases of gastric cancer adenocarcinomas and in distant metastases into the bile duct, lung and especially into the ovary (so-called Krukenberg tumors). Furthermore, over 42% of esophageal adenocarcinomas and 50% to 70% of pancreatic cancers display significant expression of CLDN18.2 [Woll et al, 2014; Lee et al, 2011; Sanada et al, 2010; Karanjawala et al, 2008; Sahin et al, 2008].

IMAB362 is being developed for the first-line treatment of adult subjects with locally advanced unresectable or metastatic CLDN18.2-positive, HER2-negative gastric or GEJ adenocarcinoma in combination with platinum- and fluoropyrimidine-based chemotherapy. For this study, a subject's tumor must express CLDN18.2 in ≥ 75% of tumor cells demonstrating moderate to strong membranous staining as determined by central immunohistochemistry (IHC) testing.

1.2 Nonclinical and Clinical Data

1.2.1 Nonclinical Data

In vitro studies with CLDN18.2-positive and negative cancer cell lines showed that IMAB362 binds to the extracellular domain 1 of CLDN18.2 on human gastric cancer cell lines with high relative affinity and selectivity. In vitro assays demonstrated that IMAB362 mediated an efficient lysis of CLDN18.2-positive cells through antibody-dependent cell-mediated cytotoxicity (ADCC) and complement-dependent cytotoxicity (CDC).

All IMAB362-mediated effects are strictly CLDN18.2 antigen-specific.

In bio-distribution studies in nude mice with human tumor xenografts, intravenously administered IMAB362 was retained, as well as specifically and strongly enriched in CLDN18.2-positive human xenografts. No or little in vivo binding of IMAB362 to any other mouse tissues including stomach tissue was observed.

Administration of repeated doses of IMAB362 to mice bearing CLDN18.2-positive tumors resulted in retardation of tumor growth kinetics in tumor models.

A series of experiments were conducted with CLDN18.2-expressing cell lines derived from NUGC-4 and KATO-III to investigate the effects of combining these chemotherapy agents with IMAB362. Combinations of chemotherapy agents used in the treatment of gastric and esophageal cancers, including 5-FU, oxaliplatin and epirubicin (e.g., 5-FU/oxaliplatin, 5-FU/oxaliplatin/epirubicin) augmented IMAB362 activity. In vitro pre-sensitization of human gastric cancer cells with chemotherapy resulted in an increase in the amount of cell surface CLDN18.2 and thus improved IMAB362-mediated ADCC and CDC.

In immunocompetent mice, IMAB362 in combination with chemotherapy resulted in a pronounced T cell infiltration into the tumors and significant long-term survival benefit over IMAB362 alone. Most likely this effect was mediated by induction of adaptive T cell immunity, which may have led to a prolonged antitumor effect and immune surveillance.

CLDN18.2 is highly conserved across species, and the epitope of IMAB362 is identical between humans, mice and cynomolgus monkeys. In addition, the binding affinity of IMAB362 to CLDN18.2 orthologs from mice, humans and cynomolgus monkeys was shown to be comparable, providing sufficient evidence that testing in mice and monkeys covers the potential on-target effects and toxicities of IMAB362.

The nonclinical pharmacology studies conducted with IMAB362 provide sufficient experimental evidence that IMAB362 depletes CLDN18.2-positive cells via ADCC and CDC. Cytotoxic drugs were shown to increase CLDN18.2 expression on human cancer cells

and to improve the activity of the major mechanism of action (ADCC and CDC). Hence, the combination of IMAB362 with first-line chemotherapy is being investigated in the clinic.

Safety pharmacology and toxicity of IMAB362 were assessed in mice and cynomolgus monkeys. In mice, the maximum exposure tested was 300 mg/kg weekly over 13 weeks, and in cynomolgus monkeys, the maximum exposure tested was 100 mg/kg weekly over 4 weeks.

In both species, no target organs of toxicity were identified; however, in monkeys, emesis was observed in a non-dose-related manner. The emesis that was observed in monkeys was not severe and spontaneously resolved despite continued dosing. The emetic potential of IMAB362 was confirmed in an investigational study in ferrets. This effect is considered to be related to the binding of IMAB362 to junctional protein, CLDN18.2, in the gastric epithelium. A human tissue cross-reactivity study of IMAB362 showed that the gastric mucosa was the only tissue with strong membrane staining. However, histological assessment of the gastric tissue in monkeys failed to identify any histopathological lesions.

Besides the findings listed previously, no other IMAB362-related adverse effects were observed in any organ, neither clinically, nor macroscopically or histologically upon postmortem analysis.

In summary, the nonclinical data outlined above supports the clinical development of IMAB362 in combination with standard chemotherapy for the treatment of CLDN18.2-positive gastric or GEJ adenocarcinoma.

Please refer to the current IB for most recent nonclinical data.

1.2.2 Clinical Data

IMAB362 has been evaluated in clinical studies as a single agent and in combination with epirubicin, oxaliplatin and capecitabine (EOX) chemotherapy or in combination with immunomodulation therapy (zoledronic acid [ZA] with or without interleukin-2 [IL-2]) for the treatment of adult subjects with CLDN18.2-positive advanced adenocarcinoma of the stomach, esophagus or GEJ.

CLDN18.2 expression was immunohistochemically determined for all subjects enrolled in the clinical studies. Several studies had an enrichment type of design meaning that only subjects above a certain threshold of CLDN18.2 positivity in their tumors were eligible for treatment. Eligibility for enrollment in the GM-IMAB-001-03 (EudraCT No. 2011-005285-38) and GM-IMAB-001-04 (EudraCT No. 2011-005509-64) studies, hereafter referred to as FAST and PILOT, respectively, was determined using the analytically validated and Conformité Européene(CE)-marked diagnostic kit, CLAUDETECT™18.2.

To date, 2 clinical studies have been completed and include GM-IMAB-001 (EudraCT No. 2008-004719-37, referred to as first-in-human [FIM]) and PILOT. Dosing is complete and final data analyses and reporting are ongoing for a third study, GM-IMAB-001-02 (EudraCT No. 2009-017365-36), referred to as MONO. The FAST study is ongoing.

IMAB362 has been granted orphan drug designation for the treatment of stomach cancer by the EMA and FDA.

Clinical data from the studies described above supports the clinical development of IMAB362 in combination with standard chemotherapy for the treatment of CLDN18.2-positive, HER2-negative locally advanced unresectable or metastatic gastric or GEJ adenocarcinomas.

Please refer to the current IB for most recent clinical data.

1.3 Summary of Key Safety Information for Study Drugs

IMAB362 has been administered to 259 subjects across 4 clinical studies:

- FIM study as a single monotherapy dose (up to 1000 mg/m^2) in 15 subjects;
- MONO study as repeated monotherapy doses up to 600 mg/m^2 once every 2 weeks in 54 subjects (maximum exposure was 72 infusions);
- FAST study as repeated doses of IMAB362 in combination with EOX chemotherapy (up to 1000 mg/m^2 once every 3 weeks) in 162 subjects (maximum exposure greater than 40 infusions); and
- PILOT study in combination with immunomodulation therapy (up to 600 mg/m^2 once every 3 weeks) in 28 subjects.

Nausea and vomiting have been confirmed as important identified risks as has hypersensitivity reactions (HSRs), including infusion-related reactions (IRRs). Anemia and neutropenia are considered important potential risks. In clinical studies, adverse reactions with nausea and/or vomiting and HSRs up to National Cancer Institute-Common Terminology Criteria for Adverse Events (NCI-CTCAE) grade 3 were reported. Please refer to the current IB for details regarding these important identified and potential risks.

In the case of IMAB362-induced nausea, vomiting or hypersensitivity including IRRs, the infusion rate of IMAB362 may be reduced or the infusion may be paused or discontinued based on investigator's clinical judgment about severity of toxicity and local standard of care. Subjects receiving IMAB362 should receive prophylactic antiemetic medications, but do not need to be premedicated for prevention of HSRs and IRRs; however, subjects should be closely monitored for IRRs to facilitate early identification and management.

The risks, both identified and potential, associated with IMAB362 are balanced by the anticipated benefits to subjects with adenocarcinomas of the stomach and the esophagus.

Potential IMAB362 toxicities, based on nonclinical studies, and important identified risks along with important potential risks, based on observations from the clinical studies, are described in Section 5.2.3 of the Investigator's Brochure (IB). Expected adverse drug reactions, including reference safety information (RSI) used for expedited health authority reporting are described in Section 5.2.4 of the IB.

Detailed information on the toxicities associated with CAPOX can be found within Section 4.8 of the EU summary of product characteristics (SPC) or local product information for each component.

Potential overlapping toxicities during treatment with IMAB362 in combination with CAPOX include nausea, vomiting, abdominal pain, constipation, diarrhea, fatigue, hypersensitivity reactions (HSR), infusion reactions, weight loss and edema.

1.4 Efficacy

1.4.1 Efficacy Results from Study GM-IMAB-001-02 (MONO)

In the MONO study, efficacy analyses included subjects who received IMAB362 at least once (Full Analysis Set [FAS]) at doses of 300 mg/m^2 (3 subjects) and 600 mg/m^2 (40 subjects). The best overall confirmed response for FAS subjects in the IMAB362 600 mg/m^2 dose group was partial response [PR] in 4 subjects (10.0%) that ranged in duration from 43 through 1037 days (GM-IMAB-001, Listing 13.2.6.4). The best overall confirmed response was stable disease (SD) in 6 subjects (15.0%) and progressive disease (PD) in 28 subjects (70.0%) in the 600 mg/m^2 group (GM-IMAB-001-02, Table 12.3.7.1). No subject achieved complete response (CR). Median progression free survival (PFS) in the FAS was 10 weeks (95% confidence interval [CI]: 8.6, 10.1 weeks) in the 600 mg/m^2 group (GM-IMAB-001-02, Table 12.3.3.1).

1.4.2 Efficacy Results from Study GM-IMAB-001-03 (FAST)

Treatment groups in the FAST study are referred to as EOX, EOX + IMAB600 (EOX plus IMAB362 600 mg/m^2 once every 3 weeks, with a loading dose of 800 mg/m^2 in cycle 1) and EOX + IMAB1000 (EOX plus IMAB362 1000 mg/m^2 once every 3 weeks).

In the FAST study, efficacy analyses of all randomized subjects were included in the intent-to-treat set and included subjects randomized to EOX only (85 subjects), EOX + IMAB600 (79 subjects) and EOX + IMAB1000 (88 subjects). Additional analyses were completed for the FAS, defined as randomized subjects who received at least 1 dose of any study drug (E, Ox, capecitabine or IMAB362). The FAS differed from the intent-to-treat set (all randomized subjects) by 6 subjects all of whom discontinued the study early without death or post-baseline tumor assessment. The reasons for early discontinuation of these subjects included protocol violation (1 subject), physician decision (1 subject), withdrawal by subject (2 subjects) and adverse event (AE) (1 subject was anemic and another had a deep vein thrombosis) (GM-IMAB-001-03, Listing 13.2.1).

<u>PFS Based on Central Independent Review (Kaplan-Meier Model, Intent-to-Treat)</u>

The addition of IMAB362 to EOX led to a statistically significant prolongation of PFS, both for the lower IMAB362 dose (hazard ratio [HR] 0.44, $P < 0.0005$) and the higher IMAB362 dose (HR 0.58, $P = 0.0114$). Median PFS was 32.4 weeks in the EOX + IMAB600 arm and 30.7 weeks in the EOX + IMAB1000 arm vs 23.1 weeks in the EOX arm, representing a median PFS prolongation by 9.3 and 7.6 weeks, respectively [IB, Table 13].

Overall Survival (Kaplan-Meier, Intent-to-Treat)

The addition of IMAB362 600 mg/m^2 led to a statistically significant prolongation in OS (HR 0.52, P < 0.0005). Median OS was 56.7 weeks in the EOX + IMAB600 arm vs 36.3 weeks in the EOX arm, representing an increase in median OS by ~20 weeks. In the EOX + IMAB1000 arm, median OS was 41.7 weeks and hence, numerically longer than in the EOX arm. However, the difference between the groups did not reach statistical significance. The difference in OS between the 2 IMAB362 doses was statistically significant (P = 0.0361) (GM-IMAB-001-03, Table 12.3.1.1.5.3).

No major imbalances were seen between the treatment groups in the subsequent use of any anticancer therapy (EOX: 38.1%; EOX + IMAB600: 37.7%; EOX + IMAB1000: 32.9%) and any chemotherapy (EOX: 34.5%; EOX + IMAB600: 36.4%; EOX + IMAB1000: 28.2%) (GM-IMAB-001-03, Tables 12.2.1.1.5.1 and 12.2.1.5 [Safety-Evaluable Set]).

Best Objective Tumor Response by Independent Review Committee

Based on confirmed responses, the ORR was 38.0% in the EOX + IMAB600 arm, 29.5% in the EOX + IMAB1000 arm and 24.7% in the EOX arm [IB, Table 17]. This included 10.1% of subjects with a CR in the EOX + IMAB600 arm, 4.5% in the EOX + IMAB1000 arm and 3.5% in the EOX arm [IB, Table 12.3.3.1.3].

1.4.3 Efficacy of CAPOX

The results from Study GM-IMAB-001-03 (FAST) using EOX support the use of CAPOX in this study, as CAPOX is similar to EOX except that CAPOX does not include epirubicin. Subjects in this study will receive CAPOX, which is an accepted standard-of-care treatment for subjects with locally advanced unresectable metastatic gastric or GE cancer [NCCN, 2017].

1.5 Risk Benefit Assessment

IMAB362 is an investigational agent in the treatment of CLDN18.2-positive, HER2-negative locally advanced unresectable or metastatic gastric or GEJ adenocarcinoma in combination with standard of care oxaliplatin and fluoropyrimidine-based combination chemotherapy as first-line treatment.

Based on clinical efficacy and safety data from the MONO study, preliminary data from the FAST study and supportive preclinical pharmacological studies, there is potential of achieving clinically relevant benefit in the later line setting as a single agent or in combination with oxaliplatin and fluoropyrimidine-based chemotherapy in a first-line setting.

Important identified risks of IMAB362 are:

- Nausea
- Vomiting
- HSRs (including IRRs)

Important potential risks of IMAB362 include:

- Neutropenia
- Anemia

Based on currently available clinical data, IMAB362 was tolerated from a safety perspective and most observed AEs have been considered manageable. Non-clinical toxicity data were supportive of the clinical findings. The risks associated with IMAB362 are well managed with dose modifications and pretreatment with antiemetics. Subjects receiving IMAB362 do not need to be premedicated for prevention of HSRs and IRRs; however, subjects should be closely monitored for IRRs to facilitate early identification and management.

Overall, the potential benefits of IMAB362 in combination with CAPOX outweigh the risks and the available nonclinical and clinical data support further clinical development for subjects with CL DN18.2-positive, HER2 negative locally advanced unresectable or metastatic gastric or GEJ adenocarcinoma who meet protocol eligibility criteria.

As described in [Section 10.1], an Independent Data Monitoring Committee (IDMC) will be responsible for reviewing the unblinded data from the study to ensure the safety of the subjects.

2 STUDY OBJECTIVE(S), DESIGN, AND ENDPOINTS

2.1 Study Objective(s)

2.1.1 Primary Objectives

The primary objective is to evaluate the efficacy of IMAB362 plus CAPOX compared with placebo plus CAPOX (as first-line treatment) as measured by Progression Free Survival (PFS) in subjects with Claudin (CLDN) 18.2-positive, human epidermal growth factor receptor 2 (HER2)-negative locally advanced unresectable or metastatic gastric and gastroesophageal junction (GEJ) adenocarcinoma

2.1.2 Secondary Objectives

The secondary objectives are:

- To evaluate efficacy as measured by Overall Survival (OS) as a key secondary objective
- To evaluate efficacy as measured by Objective Response Rate (ORR)
- To evaluate efficacy as measured by Duration of Response (DOR)
- To evaluate safety and tolerability of IMAB362
- To evaluate health related quality of life (HRQoL) using the parameters as measured by European Organization for Research and Treatment of Cancer (EORTC) Quality of Life Questionnaire (QLQ)-C30 and QLQ-OG25 plus STO22 Belching subscale, Global Pain (GP) and the EuroQOL Five Dimensions Questionnaire 5L (EQ5D-5L) questionnaires
- To evaluate the pharmacokinetics of IMAB362
- To evaluate the immunogenicity profile of IMAB362

2.1.3 Exploratory Objective

The exploratory objectives are:

- To evaluate efficacy as measured by Time to Progression (TTP)
- To evaluate PFS following subsequent anticancer treatment (PFS2)
- To evaluate Disease Control Rate (DCR)
- To evaluate potential genomic and/or other biomarkers that may correlate with treatment outcome to IMAB362 and CAPOX.
- To evaluate Health Resource Utilization (HRU)

2.2 Study Design and Dose Rationale

2.2.1 Study Design

This global, multicenter, double-blind, 1:1 randomized, phase 3 study will evaluate efficacy of IMAB362 plus CAPOX versus placebo plus CAPOX as first-line treatment in subjects with CLDN18.2-positive, HER2-negative locally advanced unresectable or metastatic gastric and GEJ adenocarcinoma.

PFS as assessed by the Independent Review Committee (IRC) is the primary outcome. Secondary outcomes include OS, ORR, DOR, safety and tolerability, HRQoL, pharmacokinetics and the immunogenicity profile of IMAB362. Exploratory outcomes include TTP, PFS2, DCR, biomarkers, and HRU.

Approximately 500 subjects will be randomized 1:1 into 1 of 2 treatment arms:

- Arm A (IMAB362 in combination with CAPOX chemotherapy)
- Arm B (placebo in combination with CAPOX chemotherapy)

Randomization of subjects will be stratified by the following factors:

- Region (Asia vs Non-Asia)
- Number of Metastatic Sites (0 to 2 vs ≥ 3)
- Prior Gastrectomy (Yes or No)

Screening:

The Screening period is 45 days. Re-screening may be allowed upon discussion with the medical monitor.

Formalin fixed paraffin embedded (FFPE) tumor tissue will be collected for central testing to determine CLDN18.2 and HER2 status. Confirmation of CLDN18.2-positive and HER2-negative status is to be obtained prior to subjects proceeding to any other Screening procedures.

Archival tumor tissue is preferred.

- A minimum of 1 FFPE tumor tissue block (preferred) OR a minimum of 15 FFPE unstained slides are required. If slides are submitted, the slides should be freshly cut from the FFPE block within the time frame described in the laboratory manual.
- If local HER2 results are already available from local testing, a minimum of 12 FFPE unstained slides are required to be submitted to the central laboratory.

- If the specimen is insufficient or unavailable, a biopsy may be performed to obtain tumor sample.
  - Sponsor pre-approval is required when the sole purpose of the biopsy procedure is to assess eligibility for this study.
  - If the required number of slides cannot be provided, the sponsor or designee should be contacted for further guidance.

Re-Screening:

Subjects who have failed screening are allowed to be re-screened one time after consultation with the medical monitor. A new subject number will be assigned. Subjects have to re-consent to the study and all screening procedures must be repeated, with the exception of the CLDN18.2 and HER2 testing as well as the radiologic imaging procedure to confirm eligibility if the scan is within 28 days prior to the first dose of study treatment.

Laboratory values re-tested within the original 45-day screening period are not considered re-screening and no new subject number will be assigned.

Treatment Period:

Subjects will be treated with either IMAB362 (Arm A) or placebo (Arm B) on day 1 of each cycle until the subject meets study treatment discontinuation criteria. For all study treatments, a cycle is defined as 21 days.

Subjects will also receive up to 8 cycles of CAPOX treatment. Oxaliplatin is administered on day 1 of each cycle, whereas capecitabine is taken twice daily on days 1 through 14. After 8 cycles, subjects may continue to receive capecitabine taken twice daily on days 1 through 14 of each cycle at the investigator's discretion until the subject meets study treatment discontinuation criteria.

Radiologic imaging will be evaluated every 9 weeks (± 7 days) counting from cycle 1/day 1 (C1D1) for the first 54 weeks and every 12 weeks (± 7 days) thereafter until subject develops radiological disease progression per Response Evaluation Criteria In Solid Tumors (RECIST) 1.1 by IRC or starts other systemic anticancer treatment, whichever occurs earlier.

Study Treatment Discontinuation and Safety Follow-up Visits:

Following discontinuation from IMAB362/placebo, subjects will have an IMAB362/placebo Study Treatment Discontinuation Visit within 7 days after the decision to discontinue, and 30-day and 90-day Safety Follow-up Visits after their last dose of IMAB362/placebo.

Additionally, if CAPOX (both components) is discontinued on a different day than IMAB362/placebo, subjects will also have a Study Treatment Discontinuation Visit within 7 days after the decision to discontinue, and 30-day and 90-day Safety Follow-up Visits after the last dose of CAPOX (both components). The CAPOX 30-day and 90-day Safety Follow-up Visits may be conducted by telephone if the subject is unable to visit the study site and will require contact for AE collection only.

Post-treatment Follow-up Period (for PFS):

If a subject discontinues all study treatments (IMAB362/placebo and both components of CAPOX) prior to disease progression per IRC, the subject will enter the Post-treatment Follow-up Period and continue to undergo scheduled imaging assessments every 9 weeks (±7 days) (or every 12 weeks [±7 days] if subject has been on study over 54 weeks) until radiologic disease progression (i.e., PFS event) per IRC, or until the subject starts any other anticancer treatment, whichever occurs earlier.

If study treatments (IMAB362/placebo and both components of CAPOX) are discontinued due to disease progression (PFS event), the subject will enter the Long-term and Survival Follow-up Period.

Long-term Follow-up Period (for PFS2) and Survival Follow-up (for OS) Period:

Following disease progression on first-line treatment or start of any other anticancer treatment, subjects will be followed in the Long-term and Survival Follow-up Period per institutional guidelines, but not less than every 12 weeks. Subsequent anticancer treatment details, progression status and survival status will be collected until PD following subsequent anticancer therapy (PFS2) is documented, or the subject starts another systemic anticancer treatment, whichever occurs earlier. Radiologic imaging for PFS2 will be done per local standard of care and read locally. Subjects will continue to be followed for survival status (OS) in the Survival Follow-up Period until death (from any cause).

All postprogression details including subsequent anticancer treatment and date and site of progression will be recorded on the electronic case report form (eCRF). Subject contact by phone or other remote method is sufficient during Long-term and Survival Follow-up. Additional follow-up contacts may be required per sponsor request for analysis purposes.

Independent Data Monitoring Committee and Independent Data Analysis Center:

An IDMC will be established and will monitor the ongoing benefit-risk status of study treatment in an unblinded fashion per a pre-defined IDMC charter. The first IDMC meeting will be approximately 6 weeks after the 40th subject enrolled has completed or discontinued cycle 2 (6 weeks) and meetings will be conducted thereafter, as defined in the IDMC charter.

An Independent Data Analysis Center will conduct an interim analysis of OS at the same time as the final PFS analysis, which will occur when approximately 344 PFS events have occurred. This analysis will be utilized by the IDMC to recommend whether the study should be stopped earlier than planned if IMAB362 in combination with CAPOX has a favorable outcome compared with placebo in combination with CAPOX. If the OS interim analysis demonstrates a highly more favorable outcome for IMAB362 in combination with CAPOX, the study may be stopped for success. However, any subject continuing to derive clinical benefit from IMAB362/placebo in combination with CAPOX, as assessed by the investigator, will be allowed to continue treatment.

2.2.2 Dose Rationale

The dose of IMAB362 in this study is an 800 mg/m^2 loading dose (C1D1) followed by 600 mg/m^2 every 3 weeks in combination with CAPOX. This dose and schedule of IMAB362 (800/600 mg/m^2 every 3 weeks) were chosen based on the observed data from the GM-IMAB-001-03 (FAST) study. In the FAST study, addition of IMAB362 800/600 mg/m^2 every 3 weeks to EOX (Arm 2) demonstrated a statistically significant and clinically meaningful improvement on PFS and OS in subjects with CLDN18.2-positive advanced gastric/GEJ cancer compared to EOX (Arm 1). The mean serum trough concentration of IMAB362 was maintained above the targeted value of 50 µg/mL (based on half maximal effective concentration [EC_{50}] of in vitro ADCC and CDC activities) following 800/600 mg/m^2 every 3 weeks administration. A higher IMAB362 dose (1000 mg/m^2 every 3 weeks) in combination with EOX was added 18 months later (with the allocation ratio of 1:1:7) as Arm 3 in the FAST study to evaluate safety and efficacy at the higher dose. However, this arm had less treatment benefit compared to Arm 2. Although there were numerical differences observed in some demographics and baseline characteristics, the sample size was not sufficient to evaluate and confirm the impact of such differences; therefore, the reason for underperformance in Arm 3 remains inconclusive.

There is no single standard, globally accepted first-line reference chemotherapeutic regimen for advanced gastric cancer. A fluoropyrimidine (capecitabine or 5-FU) in combination with platinum agent (cisplatin or oxaliplatin) is an accepted standard of care in both Western and Asian countries [NCCN, 2017; Ohtsu et al, 2011; Ajani et al, 2010; Vita et al, 2005; Kim et al, 1993]. Both classes of agents are considered to be interchangeable according to NCCN and European Society for Medical Oncology treatment guidelines. Subjects in this study will receive CAPOX (a combination of capecitabine and oxaliplatin), which is a globally accepted standard-of-care treatment for subjects with locally advanced unresectable or metastatic gastric or GE cancer. The safety of this combination regimen is well-documented.

2.3 Endpoints

2.3.1 Primary Endpoints

The primary endpoint is PFS, which is defined as the time from the date of randomization until the date of radiological PD (per RECIST 1.1 by IRC) or death from any cause, whichever is earliest.

2.3.2 Secondary Endpoints

The secondary endpoints are:

- OS, defined as the time from the date of randomization until the date of death from any cause
- ORR, defined as the proportion of subjects who have a best overall response (BOR) of CR or PR as assessed by IRC per RECIST 1.1
- DOR, defined as the time from the date of the first response (CR/PR) until the date of PD as assessed by IRC per RECIST 1.1 or date of death from any cause, whichever is earliest

- Safety and tolerability, as measured by AEs, laboratory test results, vital signs, electrocardiograms (ECGs) and Eastern Cooperative Oncology Group (ECOG) performance status
- HRQoL, as collected via EORTC QLQ-C30, QLQ-OG25 plus STO22 Belching subscale, GP and EQ5D-5L questionnaires
- Pharmacokinetics of IMAB362, C_{trough}
- Immunogenicity of IMAB362 as measured by the frequency of anti-drug antibody (ADA) positive subjects.

2.3.3 Exploratory Endpoints

The exploratory endpoints are:

- TTP, defined as the time from the date of randomization until the date of PD as assessed by IRC per RECIST 1.1.
- PFS2, defined as the time from the date of randomization until the date of PD (per investigator) following subsequent anti-cancer therapy, death from any cause or start of any other anti-cancer therapy, whichever is earliest.
- DCR, defined as the proportion of subjects who have a BOR of CR, PR or SD as assessed by IRC per RECIST 1.1.
- Potential genomic and/or other exploratory biomarkers that may be related to treatment outcome of IMAB362
- Health Resource Utilization (HRU)

3 STUDY POPULATION

3.1 Selection of Study Population

Subjects with locally advanced unresectable or metastatic gastric or GEJ adenocarcinoma whose tumors are CLDN18.2 positive, HER2-negative and who have not been previously treated for metastatic disease with chemotherapy (1st line).

For the purpose of this study, CLDN18.2-positive is defined as CLDN18.2 expression in ≥ 75% of tumor cells demonstrating moderate to strong membranous staining as determined by central IHC testing.

3.2 Inclusion Criteria

Waivers to the inclusion criteria will **NOT** be allowed.

General Criteria:

1. Institutional Review Board (IRB)/Independent Ethics Committee (IEC) approved written informed consent and privacy language as per national regulations (e.g., Health Insurance Portability and Accountability Act [HIPAA] Authorization for US sites) must be obtained from the subject or legally authorized representative (if applicable) prior to any study-related procedures.

2. Subject is considered an adult (e.g., ≥ 18 years of age in the US) according to local regulation at the time of signing the informed consent.
3. A female subject is eligible to participate if she is not pregnant (negative serum pregnancy test at screening; female subjects with elevated serum beta human chorionic gonadotropin (βhCG) and a demonstrated non-pregnant status through additional testing are eligible) and at least 1 of the following conditions applies:
    - Not a woman of childbearing potential (WOCBP) as defined in [Appendix 12.3 Contraception Requirements]

    OR

    - WOCBP who agrees to follow the contraceptive guidance as defined in [Appendix 12.3 Contraception Requirements] throughout the treatment period and for 6 months after the final study treatment administration
4. Female subject must agree not to breastfeed starting at screening and throughout the study period, and for 6 months after the final study treatment administration.
5. Female subject must not donate ova starting at screening and throughout the study period, and for 6 months after the final study treatment administration.
6. A male subject with female partner(s) of childbearing potential must agree to use contraception as detailed in [Appendix 12.3 Contraception Requirements] during the treatment period and for 6 months after the final study treatment administration.
7. A male subject must not donate sperm during the treatment period and for 6 months after the final study treatment administration.
8. Male subject with a pregnant or breastfeeding partner(s) must agree to remain abstinent or use a condom for the duration of the pregnancy or time partner is breastfeeding throughout the study period and for 6 months after the final study treatment administration.
9. Subject agrees not to participate in another interventional study while receiving study drug in present study.

Disease Specific Criteria:

10. Subject has histologically confirmed diagnosis of Gastric or GEJ adenocarcinoma.
11. Subject has radiologically confirmed locally advanced unresectable or metastatic disease within 28 days prior to the first dose of study treatment.
12. Subject has measurable disease according to RECIST 1.1 within 28 days prior to the first dose of study treatment. For subjects with only 1 measurable lesion and prior radiotherapy, the lesion must be outside the field of prior radiotherapy or must have documented progression following radiation therapy.
13. Subject's tumor expresses CLDN18.2 in ≥ 75% of tumor cells demonstrating moderate to strong membranous staining as determined by central IHC testing.

14. Subject has a HER2-negative tumor as determined by local or central testing on a gastric or GEJ tumor specimen.

Physical or Laboratory Findings:

15. Subject has ECOG performance status 0 or 1.
16. Subject has predicted life expectancy ≥ 12 weeks in the opinion of the investigator.
17. Subject must meet all of the following criteria based on the centrally analyzed laboratory tests within 14 days prior to the first dose of study treatment. In case of multiple central laboratory data within this period, the most recent data should be used to determine eligibility.
    - Hemoglobin (Hb) ≥ 9 g/dl. NOTE: subject must not have received any growth factor or blood transfusions within 14 days prior to the hematology values obtained at screening. Subjects requiring transfusions to meet eligibility criteria are not eligible.
    - Absolute Neutrophil Count (ANC) ≥ $1.5x10^9$/L
    - Platelets ≥ $100x10^9$/L
    - Albumin ≥ 2.5 g/dL
    - Total Bilirubin ≤ 1.5 x upper limit of normal (ULN)
    - Aspartate aminotransferase (AST) and alanine aminotransferase (ALT) ≤ 2.5 x ULN without liver metastases (or ≤ 5 x ULN if liver metastases are present)
    - Either serum creatinine ≤ 1.5 x ULN or estimated glomerular filtration rate ≥ 50 mL/min/1.73 m^2
    - Prothrombin time/international normalized ratio (PT/INR) and partial thromboplastin time (PTT) ≤ 1.5 x ULN (except for subjects receiving anticoagulation therapy)

3.3 Exclusion Criteria

Waivers to the exclusion criteria will **NOT** be allowed.

Subject who meets any of the following exclusion criteria prior to enrollment is not eligible for enrollment:

Prohibited Treatment or Therapies:

1. Subject has received prior systemic chemotherapy for locally advanced unresectable or metastatic gastric or GEJ adenocarcinoma. However, subject may have received either neo-adjuvant or adjuvant chemotherapy as long as it was completed at least 6 months prior to the first dose of study treatment.
2. Subject has received radiotherapy for locally advanced unresectable or metastatic gastric or GEJ adenocarcinoma unless the radiotherapy was completed > 28 days prior to the first dose of study treatment. Subject who received palliative radiotherapy to peripheral bone metastases ≥ 14 days prior to first dose of study treatment and has recovered from all acute toxicities is eligible.

3. Subject has received treatment with herbal medications or other treatments that have known antitumor activity within 28 days prior to first dose of study treatment.
4. Subject has received systemic immunosuppressive therapy, including systemic corticosteroids within 14 days prior to first dose of study treatment. Subject using a physiologic replacement dose of hydrocortisone or its equivalent (defined as up to 30 mg per day of hydrocortisone or up to 10 mg per day of prednisone) is eligible.
5. Subject has received other investigational agents or devices within 28 days prior to first dose of study treatment.

Medical History or Concurrent Disease:

6. Subject has prior severe allergic reaction or intolerance to a monoclonal antibody, including humanized or chimeric antibodies.
7. Subject has known immediate or delayed hypersensitivity, intolerance or contraindication to any component of study treatment.
8. Subject has prior severe allergic reaction or intolerance to any component of CAPOX.
9. Subject has known dihydropyrimidine dehydrogenase (DPD) deficiency. (NOTE: Subjects with low or absent DPD should not be treated with capecitabine due to increased risk for severe, life-threatening, or fatal adverse reactions.)
10. Subject has gastric outlet syndrome or persistent/recurrent vomiting.
11. Subject had recent gastric bleeding and/or is symptomatic with proven gastric ulcers that excludes the subject from participation per investigator judgement.
12. Subject has a known history of a positive test for human immunodeficiency virus (HIV) infection or known active hepatitis B (positive HBs Ag) or C infection. For subjects who are negative for HBs Ag, but HBc Ab positive, an HB DNA test will be performed and if positive, the subject will be excluded. Subjects with positive serology but negative hepatitis C virus (HCV) RNA test results are eligible.
13. Subject has an active autoimmune disease that has required systemic treatment within the past 2 years.
14. Subject has active infection requiring systemic therapy that has not completely resolved within 14 days prior to first dose of study treatment.
15. Subject has significant cardiovascular disease, including:
    - Congestive heart failure (defined as New York Heart Association [NYHA] Class III or IV), myocardial infarction, unstable angina, coronary angioplasty, coronary stenting, coronary artery bypass graft, cerebrovascular accident (CVA), or hypertensive crisis within 6 months prior to administration of first dose of study treatment;
    - History of clinically significant ventricular arrhythmias (i.e., sustained ventricular tachycardia, ventricular fibrillation, or Torsades de Pointes);
    - QTc interval > 450 msec for male subjects; QTc interval > 470 msec for female subjects;

- Cardiac arrhythmias requiring anti-arrhythmic medications (Subjects with rate controlled atrial fibrillation for > 1 month prior to first dose of study treatment are eligible.)

16. Subject has known central nervous system (CNS) metastases and/or carcinomatous meningitis.
17. Subject has known peripheral sensory neuropathy > grade 1 unless the absence of deep tendon reflexes is the sole neurological abnormality.
18. Subject has had a major surgical procedure and has not completely recovered within 28 days prior to the first dose of study treatment.
19. Subject has psychiatric illness or social situations that would preclude study compliance, per investigator judgement.
20. Subject has another malignancy for which treatment is required per investigator's clinical judgment.
21. Subject has any concurrent disease, infection, or co-morbid condition that interferes with the ability of the subject to participate in the study, which places the subject at undue risk or complicates the interpretation of data in the opinion of the investigator.

4 IDENTIFICATION OF STUDY TREATMENT(S)

4.1 IMAB362 (Investigational Product)

The investigational product, IMAB362, is a sterile lyophilized powder with the chimeric (mouse/human IgG1) monoclonal antibody IMAB362 as the active pharmaceutical ingredient.

The investigational product is supplied by Astellas in single-use glass vials containing 105 mg of IMAB362. All excipients are animal component free and of compendial grade (Pharm. Eur. current version). No preservatives are contained, since the vial is designed for single use.

The investigational product should be stored at refrigerated conditions (2°C to 8°C; 36°F to 46°F). Temperature should be controlled and monitored. Details of investigational product receipt, labeling, storage and preparation are provided in the Pharmacy Manual.

The investigational product has to be reconstituted with 5.0 mL water for injection to a concentration of 20 mg/mL. Further dilution in an IV bag with sterile 0.9% sodium chloride to a final concentration of 2 mg/mL is required. The time between the beginning of reconstitution and the beginning of infusion must be within 4 hours at controlled room temperature (15°C to 25°C). If the infusion cannot be initiated within the 4 hours, the infusion bag must be stored at 2°C to 8°C and infusion must be initiated within 24 hours from the beginning of reconstitution.

The IMAB362 used in this study will be prepared, packaged, and labeled under the responsibility of qualified staff at Astellas Pharma Global Development, Inc. (APGD), Astellas US Technologies, Inc. or sponsor's designee in accordance with APGD or sponsor's

designee Standard Operating Procedures (SOPs), Good Manufacturing Practices (GMP) guidelines, International Council for Harmonisation (ICH) Good Clinical Practice (GCP) guidelines, and applicable local laws/regulations.

Each vial and carton will bear a label conforming to regulatory guidelines, GMP and local laws and regulations that identifies the contents as investigational drug.

As required, a qualified person of Astellas Pharma Europe B.V. (APEBV) or sponsor’s designee will perform the final release of the medication according to the requirements of the EU Directive 2003/94/EC annex 13.

4.2 Comparative Drug (Placebo)

Placebo will not be manufactured or provided by the sponsor. Sites should use their own commercial supply of 0.9% Sodium Chloride Injection as placebo.

For manufacturing, formulation, storage, handling and preparation details please refer to the package insert, SPC or local product information supplied by the manufacturer.

4.3 CAPOX

Capecitabine and Oxaliplatin (CAPOX) are administered in combination with IMAB362/placebo. CAPOX products should be given according to institutional standards, published guidelines, the respective product package insert(s) or dosed according to this protocol.

CAPOX treatment will be supplied by the responsible site pharmacy of each investigational site or by the sponsor at the sponsor’s discretion. Generic drug may be used where approved by the respective regulatory authority. CAPOX treatment will be prepared and/or dispensed by the responsible site pharmacy of each investigational site.

For manufacturing, formulation, storage, handling and preparation details please refer to the package insert, SPC or local product information supplied by the manufacturer.

If CAPOX is supplied by the sponsor, CAPOX used in this study will be packaged and labeled under the responsibility of qualified staff at APGD/Astellas US Technologies, Inc. (AUST) sponsor’s designee in accordance with APGD-AUST or sponsor’s designee SOPs, GMP guidelines, ICH GCP guidelines, and applicable local laws/regulations.

Each product will bear a label conforming to regulatory guidelines, GMP and local laws and regulations that identifies the contents as investigational drug.

As required, a qualified person of APEBV or sponsor’s designee will perform the final release of the medication according to the requirements of the EU Directive 2003/94/EC annex 13.

4.4 Other Drug(s)

4.4.1 Antiemetic Premedication

Prophylactic antiemetics will not be provided by the sponsor but rather will be sourced by the Sites via commercial supply.

For manufacturing, formulation, storage, handling and preparation details please refer to the package insert, SPC or local product information supplies by the manufacturer.

4.5 Study Drug Handling

Current ICH GCP Guidelines require the investigator to ensure that study treatment and other drug deliveries from the sponsor are received by the investigator or designee and that:

- Such deliveries are recorded,
- Study drug is handled and stored according to labeled storage conditions,
- Study drug with appropriate expiry/retest and is only dispensed to study subjects in accordance with the protocol, and
- Any unused study drug is returned to the sponsor, unless prior approval is received from the sponsor allowing local standard procedures for the alternative disposition of unused study drug.

Study drug inventory and accountability records will be kept by the investigator, head of study site (***SPECIFIC TO SITES IN JAPAN***) or designee. Study drug accountability throughout the study must be documented and reconciled. The following guidelines are therefore pertinent:

- The investigator agrees not to supply study drugs to any persons except the eligible subjects in this study in accordance with the protocol.
- The investigator or designee will keep the study drugs in a pharmacy or other locked and secure storage facility under controlled storage conditions, accessible only to those authorized by the investigator to dispense these study drugs.
- A study drug inventory will be maintained by the investigator or designee. The inventory will include details of material received and a clear record of when they were dispensed and to which subject.
- At the conclusion or termination of this study, the investigator or designee agrees to conduct a final drug supply inventory and to record the results of this inventory on the Drug Accountability Record. It must be possible to reconcile delivery records with those of used and/or returned study drug. Any discrepancies must be accounted for and documented. Appropriate forms of deliveries and returns must be signed by the site staff delegated this responsibility.
- The site staff must return unused study drug to the sponsor or designee at the end of the study or upon expiration unless otherwise approved by the sponsor.

4.6 Blinding

4.6.1 Blinding Method

The clinical study will be conducted as subject- and investigator-blinded. Subjects will be randomized to receive IMAB362 or placebo in a blinded fashion such that neither the investigator, sponsor’s study management team, clinical staff, nor subject will know which agent is being administered. All pharmacy-related tasks must be conducted by an unblinded pharmacist/designee. The unblinded pharmacist/designee must not share any unblinded information with the blinded site personnel

Upon the need for unblinding (e.g., real-time assessment of the safety data), the chair of the IDMC can ask for the release of the treatment assignment for 1 or more subjects and will identify the sponsor staff who will receive the information.

4.6.2 Confirmation of the Indistinguishability of the Study Drugs

The appearance of the diluted IMAB362 solution for infusion is identical to commercial saline solution (placebo). In order to maintain the blind, the subjects randomized to the placebo treatment arm (Arm B) will receive placebo in a volume and route corresponding to the appropriate IMAB362 dose (Arm A). The unblinded pharmacist/designee will provide the investigator or designee with blinded study drugs to dose the subjects. Refer to the pharmacy manual for detailed information.

4.6.3 Retention of the Assignment Schedule and Procedures for Treatment Code Breaking

The randomization list and study drug blind will be maintained by the Interactive Response Technology (IRT) system.

4.6.4 Breaking the Treatment Code for Emergency

The treatment code for each randomized subject will be provided by the IRT in the event of a medical emergency requiring knowledge of the treatment assigned to the subject. The IRT will be programmed with blind-breaking instructions that may only be requested by the investigator or subinvestigators designated to have access to perform blind-break. No subjects or other study personnel, other than the unblinded pharmacist or designee, will be made aware of the treatment given to any subject unless a medical emergency necessitates such disclosure. In case of a medical emergency, the investigator has the sole responsibility for determining if unblinding of subject’s treatment assignment is warranted. Subject safety must always be the first consideration in making such determination. If the investigator decides that unblinding is warranted, the investigator should make every effort to contact the sponsor prior to unblinding a subject’s treatment assignment unless this could delay emergency treatment for the subject. Any unblinding by the investigators must be reported immediately to the sponsor and must include an explanation of why the study drug was unblinded.

The investigator must have confirmed functionality to access code-break through the IRT system and must have a designated back up (e.g. redundant processes) to support emergency unblinding requirements.

Prior to randomization, subjects should be provided with information that includes the site emergency contact number and back-up contact number in case of a medical emergency. Any unblinding by the investigational staff must be reported immediately to the sponsor and include an explanation of why the study drug was unblinded. If unblinding is associated with a SAE the investigator is to follow the instructions in [Section 5.5.5 Reporting of SAE].

Care should be taken to limit knowledge of the randomization arm, in case this could affect the blinding of other subjects or future study assessment for the subject.

The time, date, subject number and reason for obtaining any of these codes, and therefore breaking the blind, must be documented in the study file.

If possible, the sponsor should be contacted prior to unblinding of the study drug.

4.6.5 Breaking the Treatment Code by the Sponsor

The sponsor may break the treatment code for subjects who experience a Suspected Unexpected Serious Adverse Reaction (SUSAR), in order to determine if the individual case or a group of cases requires expedited regulatory reporting. Individual Emergency Codes will be provided to the limited staff who are responsible to break the codes for all SUSAR cases for reporting purposes.

4.7 Assignment and Allocation

Subject randomization will be performed via IRT and treatment assigned in a 1:1 ratio to IMAB362 or placebo. Prior to the initiation of the study treatment, the unblinded pharmacist/designee will contact the IRT system in order to determine the randomly assigned treatment. The unblinded pharmacist/designee will dispense the treatment according to the IRT system’s assignment. Specific procedures for randomization through the IRT are contained in the IRT manual.

Randomization will be stratified by:

- Region (Asia vs Non-Asia)
- Number of Metastatic Sites (0 to 2 vs ≥ 3)
- Prior Gastrectomy (Yes or No)

5 TREATMENTS AND EVALUATION

5.1 Dosing and Administration of Study Drug(s) and Other Medication(s)

5.1.1 Dose/Dose Regimen and Administration Period

5.1.1.1 IMAB362/Placebo

Subjects will be administered IMAB362/placebo as a 2-hour intravenous infusion on day 1 of each cycle starting with a loading dose of 800 mg/m² of IMAB362 at C1D1 followed by subsequent doses of 600 mg/m² every 3 weeks. IMAB362/placebo should be administered prior to CAPOX.

Please also refer to the dosing schematics for details [Section V].

It is recommended that IMAB362/placebo infusion not exceed 6 hours from start of infusion.

5.1.1.2 Antiemetics

Prophylactic antiemetics should be administered according to institutional standard of care, published guidelines and the respective product package insert(s).

(NOTE: Subjects receiving IMAB362 do not need to be premedicated for prevention of IRRs; however, subjects should be closely monitored for IRRs to facilitate early identification and management.)

- All antiemetic premedication should be given at minimum 30 minutes prior to IMAB362/placebo treatment.
- It is recommended that the prophylactic antiemetic regimen include (but is not limited to) the following agents:
  - NK-1 receptor blockers
  - 5-HT3 receptor blockers*

*To minimize the risk of Torsades de Pointes, avoid use in subjects with congenital long QT syndrome and administer with caution to subjects who have or may develop QTc prolongation.

Corticosteroids:

- The impact of corticosteroids on the potential efficacy of IMAB362 is not known. Therefore, consideration should be given to avoid or minimize the use of corticosteroids as a prophylactic antiemetic, if possible.
- For a subject's first dose of IMAB362/placebo, it is recommended that the prophylactic use of corticosteroids be avoided.

5.1.1.3 CAPOX

Subjects will receive CAPOX treatment until IRC confirmed disease progression or a total of 8 cycles (each cycle is defined as 3 weeks = 21 days). Oxaliplatin is administered on day 1 of each cycle, whereas capecitabine is taken twice daily on days 1 through 14.

After 8 cycles of CAPOX, subjects may continue to receive capecitabine twice daily on days 1 through 14 of each cycle at the investigator's discretion until the subject meets study treatment discontinuation criteria.

CAPOX is to be administered after IMAB362/placebo infusion.

- Oxaliplatin: 130 mg/m2 intravenous infusion on day 1 of each cycle over 2 hours (or longer per institutional standard of care) for up to 8 cycles.
- Capecitabine: Administered orally at 1000 mg/m2 twice daily (bid) (total daily dose is 2000 mg/m2) on days 1 through 14 of each cycle. Capecitabine can be continued beyond 8 cycles based on investigator's judgment.
  - When possible, the first dose of Capecitabine on day 1 should be administered on site under the supervision of the site staff.
  - In case the first dose of Capecitabine on day 1 of a cycle is administered too late in the day for the second dose to be administered on the same day, the last dose of that cycle should be taken in the morning of day 15.
  - Capecitabine should be taken within 30 minutes after a meal.

5.1.2 Study Treatment Dose Modifications, Delays And Interruptions

5.1.2.1 Increase or Reduction of IMAB362/Placebo

Dose increase or dose reduction for IMAB362/placebo is not allowed. Body surface area should only be recalculated if there is a weight change of at least 10% since the last dose.

5.1.2.2 IMAB362/Placebo Interruption or Permanent Discontinuation

There is a +7 day allowable window for dosing IMAB362/placebo. If IMAB362/placebo treatment is delayed more than 7 days then it should be administered as soon as the reason for delay has resolved, which will then become day 1 of the next cycle. If IMAB362/placebo treatment is delayed, CAPOX/Capecitabine administration should also be delayed at the same time and can be restarted when IMAB362/placebo administration has been restarted, unless there are other reasons warranting further delay of CAPOX/Capecitabine at the investigator's discretion (refer to Section 5.1.2.4).

Permanently discontinue IMAB362/placebo treatment if delayed beyond 28 days from the last administered dose due to toxicity of IMAB362/placebo.

Note: Intravenous infusion of IMAB362/placebo should be administered as a 2-hour infusion. It is recommended that IMAB362/placebo infusion not exceed 6 hours from start of infusion.

Guidelines for IMAB362/placebo treatment modification due to non-hematologic and hematologic toxicities are described below in Table 2 and Table 3, respectively.

Table 2 Guidelines for IMAB362/Placebo Treatment Modification Due to Non-hematologic Toxicity

Toxicity	Grade 1	Grade 2	Grade 3	Grade 4
**Infusion-related reaction (IRR)**<br>See Table 4 for further guidance	Continue Infusion	Interrupt infusion. Infusion may be resumed at a reduced rate when toxicity has improved to grade ≤ 1.	Stop the infusion immediately.<br>Institute appropriate medical management immediately based on the type of reaction.<br>Permanently Discontinue IMAB362/placebo	
**Nausea**	Continue Infusion		Interrupt infusion. Hold IMAB362/placebo treatment until toxicity improved to grade ≤ 1.<br>If the investigator determines that the toxicity is not related to IMAB362 and the toxicity improves to grade ≤ 2, then infusion may be restarted at the investigator's discretion.	Not applicable
**Vomiting**	Continue Infusion		Interrupt infusion. Hold IMAB362/placebo treatment until toxicity improved to grade ≤ 1.	Permanently Discontinue IMAB362/placebo
**Other Non-hematologic toxicity**	Continue Infusion		Interrupt infusion. Hold IMAB362/placebo treatment until toxicity improved to grade ≤ 1.<br>If the investigator determines that the toxicity is not related to IMAB362 and the toxicity improves to grade ≤ 2, then infusion may be restarted at the investigator's discretion.	Permanently Discontinue IMAB362/placebo

Table 3 Guidelines for IMAB362/Placebo Treatment Modification Due to Hematologic Toxicity

Toxicity	Grade 1	Grade 2	Grade 3	Grade 4
**Neutropenia†**	ANC < LLN to 1500/mm³; < LLN to 1.5 x 10⁹/L	ANC < 1500 to 1000/mm³; < 1.5 to 1.0 x 10⁹/L	ANC < 1000 to 500/mm³; < 1.0 to 0.5 x 10⁹/L	ANC < 500/mm³; < 0.5 x 10⁹/L
Action	Continue treatment		Hold treatment and recheck blood counts weekly until ANC resolves to ≥ 1.5 x 10⁹/L (≤ grade 1) before restarting treatment. Discontinue IMAB362/placebo if ANC remains < 1.5 x 10⁹/L (≥ grade 2) after a > 28-day treatment delay.	
**Febrile Neutropenia†**	Not Applicable		ANC < 1000/mm³ with a single temperature of > 38.3°C (101°F) or a sustained temperature of ≥ 38°C (100.4°F) for more than 1 hour.	Life-threatening consequences; urgent intervention indicated
Action			Follow standard treatment guidelines. Hold treatment and recheck blood counts weekly until ANC recovers to ≥ 1.5 x 10⁹/L (grade ≤ 1) and fever has resolved. Discontinue IMAB362/placebo if ANC remains < 1.5 x 10⁹/L (≥ grade 2) after a > 28-day treatment delay.	
**Thrombocytopenia**	PLT < LLN to 75,000/mm³; < LLN to 75.0 x 10⁹/L	PLT < 75,000 to 50,000/mm³; < 75.0 to 50.0 x 10⁹/L	PLT < 50,000 to 25,000/mm³; < 50.0 to 25.0 x 10⁹/L	PLT < 25,000/mm³; < 25.0 x 10⁹/L
Action	Continue treatment		Withhold treatment and recheck blood counts weekly until platelets recover to > 75× 10⁹/L (grade ≤ 1) before restarting treatment. Discontinue IMAB362/placebo if platelets remain < 75 x 10⁹/L (Grade ≥ 2) after a > 28-day treatment delay.	
**Anemia**	Hgb < LLN to 10.0 g/dL; < LLN to 6.2 mmol/L; < LLN to 100 g/L	Hgb < 10.0 to 8.0 g/dL; < 6.2 to 4.9 mmol/L; < 100 to 80 g/L	Hgb < 8.0 g/dL; < 4.9 mmol/L; < 80 g/L; transfusion indicated	Life-threatening consequences; urgent intervention indicated

Table continued on next page

Toxicity	Grade 1	Grade 2	Grade 3	Grade 4
Action	Continue treatment		Withhold treatment.<br>Follow standard treatment guidelines. Transfuse if indicated.<br>Recheck blood counts weekly until Hgb recovers to > 8.0 g/dL (≤ Grade 2) before restarting treatment.<br>Discontinue IMAB362/ placebo if Hgb remains < 8.0 g/dL (≥ Grade 3) after a > 28-day treatment delay.	Withhold treatment.<br>Follow standard treatment guidelines including urgent intervention and blood transfusions as required.<br>The subject may be discontinued after discussion with Medical Monitor.

ANC: absolute neutrophil count; PLT: platelet count; Hgb: hemoglobin
† At the investigator's discretion growth factors may be used according to standard practice guidelines.

5.1.2.3 Guidelines for Infusion-Related Reactions for IMAB362/Placebo

Subjects should be closely monitored for IRRs to facilitate early identification and management.

The management of such toxicities should be based on investigator utilizing institutional standard of care, published guidelines and the general guidelines provided in Table 4 below.

A subject with an infusion reaction should be evaluated specifically for the symptoms and signs that are highly suggestive of anaphylaxis (urticaria, repetitive cough, wheeze and throat tightness/change in voice). A careful examination of the skin is advised in order to detect urticaria, which often appears first in the neck, trunk, abdomen and axillae.

Not all anaphylactic reactions manifest as anaphylactic shock. Because anaphylaxis can recur and worsen with re-exposure, permanently discontinue IMAB362/placebo for any subject having a reaction with features (even if mild) that are highly suggestive of anaphylaxis.

Note: Intravenous infusion of IMAB362/placebo should be administered as a 2-hour infusion. It is recommended that IMAB362/placebo infusion not exceed 6 hours from start of infusion.

Table 4 Infusion-Related Reactions

**Infusion-Related Reactions**	
**CTCAE Grade**	**Management**
Grade 1 standard infusion reactions	Continue infusion and closely monitor the subject.
Grade 2 standard infusion reaction	Interrupt.<br>Medical management as per type of reaction. Resume infusion once toxicity Grade ≤ 1 and reduce the infusion rate for the remaining infusion.<br>For the next infusion:<br>• Increase total infusion time (reduce infusion rate).<br>• Pre-medicate as appropriate.*<br>• Closely monitor the subject for symptoms and signs of an infusion reaction.
Grade 3 or 4 standard infusion reactions or any reaction with features of anaphylaxis	Stop the infusion immediately.<br>Institute appropriate medical management immediately based on the type of reaction.<br>Permanently Discontinue IMAB362/placebo<br>*Once the subject has been stabilized, collect blood for a cytokine/chemokine panel.*<br>*If the reaction is suggestive of anaphylaxis, collect blood for serum total tryptase level (levels typically peak within 3 hours after the onset of symptoms).*

CTCAE: Common Terminology Criteria For Adverse Events

* At the investigators discretion, anti-histamines may be used as pre-medication for the next infusion. Systemic corticosteroids should be avoided or minimized while subject is on study treatment unless required for management of an emergent medical condition (e.g., severe nausea/vomiting or hypersensitivity reaction).

5.1.2.4 CAPOX Dose Modification

The CAPOX dose modifications described below are based on [XELOX CCO Formulary, August 2017].

If CAPOX/Capecitabine treatment is delayed or interrupted, CAPOX/Capecitabine can be administered as soon as the reason for delay or interruption is resolved. The delay/interruption in treatment should be regarded as lost treatment days and missed doses should not be replaced; the planned treatment schedule should be maintained.

Dosing and dose modification should be based on investigator utilizing institutional standard of care, approved package insert, SPC or local product information supplied by the manufacturer for each agent (oxaliplatin, capecitabine) and general guidelines provided below.

The first dose of CAPOX should not be modified. After the assessment of tolerability, dose adjustments should be performed according to the criteria in Table 5 based on maximum hematologic or non-hematologic toxicity data from the previous cycle as shown in Table 6 and Table 7, respectively. Dose reduction criteria for oxaliplatin-related neurotoxicity are presented in Table 8. Each drug may be dose reduced independently based on the specific types of toxicities observed. No more than 2 dose reductions will be allowed per drug per

subject (see Table 5). Dose re-escalation is not permitted. If further dose reduction is required beyond the criteria in Table 5, that component of CAPOX should be discontinued.

In subjects experiencing toxicity requiring a delay or discontinuation of CAPOX, subject should continue to receive IMAB362/placebo as clinically appropriate. If CAPOX is interrupted, subject should be evaluated weekly (at a minimum) until the toxicity has improved sufficiently at which time treatment can be restarted as described in the tables below (as applicable). Unresolved toxicity > 28 days associated with CAPOX will result in the subject discontinuing CAPOX (both components).

Table 5 Dose Adjustment Levels for Oxaliplatin and Capecitabine

**Drug**		**Oxaliplatin**	**Capecitabine**
**Initial Dose**		130 mg/m^2	1000 mg/m^2 bid
**Dose Reduction**	**Level 1**	100 mg/m^2	750 mg/m^2 bid
	**Level 2**	75 mg/m^2	500 mg/m^2 bid

For the first 8 cycles, if a subject has capecitabine discontinued or interrupted, oxaliplatin should be discontinued or interrupted until capecitabine is resumed. For additional information, refer to the approved package insert, SPC or local product information supplied by the manufacturer for each agent (oxaliplatin, capecitabine).

In case Capecitabine administration is delayed or interrupted, the missed doses should not be taken later during the cycle. Capecitabine administration is to be stopped at day 14 (or the morning of day 15 as applicable) of each cycle.

5.1.2.5 CAPOX: Dose Modifications for Hematologic Toxicity

The CAPOX dose modifications for hematologic toxicity are presented in Table 6. Dose modifications should be maintained until recovery from hematologic toxicity. CAPOX should be permanently stopped in subjects not recovering from hematologic toxicity in 28 days.

Table 6 CAPOX Dose Modification Due to Hematologic Toxicity

Toxicity	Grade 1	Grade 2	Grade 3	Grade 4
**Neutropenia†**	ANC < LLN to 1500/mm^3; < LLN to 1.5 x 10^9/L	ANC < 1500 to 1000/mm^3; < 1.5 to 1.0 x 10^9/L	ANC < 1000 to 500/mm^3; < 1.0 to 0.5 x 10^9/L	ANC < 500/mm^3; < 0.5 x 10^9/L
Action	Continue treatment.	Oxaliplatin: Continue Treatment<br><br>Capecitabine: 1st, 2nd and 3rd events: Interrupt until event resolves to grade ≤ 1.<br><br>Fourth event: Discontinue treatment permanently	Hold treatment and recheck blood counts weekly until ANC resolves to ≥ 1.5 x 10^9/L before restarting treatment.<br>Discontinue CAPOX if ANC remains < 1.5 x 10^9/L after a > 28-day treatment delay.	
Dose Modification (for next treatment)	Maintain dose level.	Oxaliplatin: Maintain dose of oxaliplatin<br><br>Capecitabine: First event: Maintain dose level<br><br>Second event: Reduce capecitabine 1 level.†<br><br>Third event: Reduce capecitabine 2 levels	Oxaliplatin Dose adjustment next cycle: Reduce 1 dose level<br><br>Capecitabine Dose adjustment next cycle:<br><br>First event: Reduce capecitabine 1 level<br><br>Second event: Reduce capecitabine 2 levels.†<br><br>Third event: Discontinue capecitabine	Discontinue both. If investigator deems it to be in the subject's best interest to continue, resume next dose of oxaliplatin reduced 1 level and capecitabine reduced 2 dose levels
**Febrile Neutropenia†**			ANC < 1000/mm^3 with a single temperature of > 38.3°C (101°F) or a sustained temperature of ≥ 38°C (100.4°F) for more than 1 hour.	Life-threatening consequences; urgent intervention indicated

Table continued on next page

Toxicity	Grade 1	Grade 2	Grade 3	Grade 4
Action	Maintain dose level	Oxaliplatin: Continue Treatment<br><br>Capecitabine: 1st, 2nd and 3rd events: Interrupt until event resolves to grade ≤ 1.<br><br>Fourth event: Discontinue treatment permanently	Follow standard treatment guidelines	Proceed to next treatment when fever has resolved and ANC recovers to ≥ 1.5 x 10^9/L within 28 days
Dose Modification (at next treatment)	Maintain dose level	Oxaliplatin: Maintain dose of oxaliplatin<br><br>Capecitabine: First event: Maintain dose level<br><br>Second event: Reduce capecitabine 1 level.†<br><br>Third event: Reduce capecitabine 2 levels	Oxaliplatin Dose adjustment next cycle: Reduce 1 dose level<br><br>Capecitabine First event: Reduce capecitabine 1 level<br><br>Second event: Reduce capecitabine 2 levels.†<br><br>Third event: Discontinue capecitabine	Discontinue both. If physician deems it to be in the patient's best interest to continue, interrupt until resolved to grade 0-1 and resume next dose of oxaliplatin reduced 1 level and capecitabine by reducing 2 dose levels
**Thrombocytopenia**	PLT < LLN to 75,000/mm^3; < LLN to 75.0 x 10^9/L	PLT < 75,000 to 50,000/mm^3; < 75.0 to 50.0 x 10^9/L	PLT < 50,000 to 25,000/mm^3; < 50.0 to 25.0 x 10^9/L	PLT < 25,000/mm^3; < 25.0 x 10^9/L

Table continued on next page

Toxicity	Grade 1	Grade 2	Grade 3	Grade 4
Action	Continue treatment.	Oxaliplatin: Continue Treatment<br><br>Capecitabine: 1st, 2nd and 3rd events: Interrupt until event resolves to grade ≤ 1.<br><br>Fourth event: Discontinue treatment permanently	Withhold treatment and recheck blood counts weekly until platelets recover to > 75× 10^9/L before restarting treatment. Discontinue CAPOX if PLT remains < 75 x 10^9/L after a > 28-day delay.	
Dose Modification (for next treatment)	Maintain dose level.	Oxaliplatin: Maintain dose of oxaliplatin<br><br>Capecitabine: First event: Maintain dose level<br><br>Second event: Reduce capecitabine 1 level.<br><br>Third event: Reduce capecitabine 2 levels	Oxaliplatin Dose adjustment next cycle: Reduce 1 dose level<br><br>Capecitabine First event: Reduce capecitabine 1 level<br><br>Second event: Reduce capecitabine 2 levels.<br><br>Third event: Discontinue capecitabine	Discontinue both. If physician deems it to be in the patient's best interest to continue, interrupt until resolved to grade 0-1 and resume next dose of oxaliplatin reduced 1 level and capecitabine by reducing 2 dose levels

ANC: absolute neutrophil count; CAPOX: Capecitabine and Oxaliplatin; LLN: lower limit of normal; PLT: platelet count
† At the investigator's discretion growth factors may be used according to standard practice guidelines.

5.1.2.6 CAPOX: Dose Modification for Non-Hematologic Toxicity

CAPOX dose modifications for non-hematologic toxicity should be based on the most severe toxicity experienced during the last treatment (Table 7). Retreatment should be delayed until recovery of all non-hematologic toxicity to ≤ Grade 2 with the exception of increased bilirubin or ALT, which must recover to Grade 1 or baseline, whichever was higher. The maximum permitted treatment delay is 28 days for recovery of non-hematologic toxicity. If after a > 28-day treatment delay, the subject has not recovered sufficiently to meet retreatment criteria, CAPOX should be discontinued.

Table 7 CAPOX Dose Modification Due to Non-Hematologic Toxicity

Toxicity	Grade 1	Grade 2	Grade 3	Grade 4
**Diarrhea**				
Action	Continue treatment	Start medical management for diarrhea. Continue treatment.	Start medical management for diarrhea. Withhold all CAPOX treatment. Restart treatment after diarrhea recovers to ≤ Grade 2	
Dose Modification (for next treatment)	None	First event: None<br>Second event: Reduce capecitabine 1 dose level.	First event: Reduce oxaliplatin 1 level and capecitabine 1 level.<br>Second event: Reduce oxaliplatin 1 dose level and capecitabine 2 levels.	Reduce oxaliplatin 1 dose level and capecitabine 2 levels.
**Other Non-Hematologic Toxicities**†‡				
Action	Continue treatment	Oxaliplatin: Continue Treatment<br>Capecitabine:<br>1st, 2nd and 3rd events: Interrupt until event resolves to grade ≤ 1<br><br>Fourth event: Discontinue treatment permanently	Withhold all CAPOX treatment until toxicity improves to ≤ Grade 2.	
Dose Modification (for next treatment)	None	Oxaliplatin: Maintain dose of oxaliplatin<br><br>Capecitabine: First event: Maintain dose level<br><br>Second event: Reduce capecitabine 1 level.†<br><br>Third event: Reduce capecitabine 2 levels	Oxaliplatin Dose adjustment next cycle: Reduce 1 dose level<br><br>Capecitabine First event: Reduce capecitabine 1 level<br><br>Second event: Reduce capecitabine 2 levels.†<br><br>Third event: Discontinue capecitabine	Discontinue both If physician deems it to be in the patient's best interest to continue, interrupt until resolved to grade 0-1 and resume next dose of oxaliplatin reduced 1 level and capecitabine by reducing 2 dose levels

CAPOX: Capecitabine and Oxaliplatin

† For skin toxicity, reduce capecitabine only, not oxaliplatin.

‡ Exceptions: alopecia, fatigue, anorexia, nausea/vomiting (if can be controlled by antiemetics) and constipation (if can be controlled with laxatives, stool softeners, etc.).

5.1.2.7 Guidelines for Infusion-Related Reactions for Oxaliplatin

Subjects should be closely monitored for IRRs to facilitate early identification and management. The management of such toxicities should be based on investigator utilizing institutional standard of care.

5.1.2.8 Oxaliplatin-Induced Neurotoxicity and Laryngopharyngeal Dysesthesia

Oxaliplatin is known to be associated with peripheral neuropathy, including paresthesia and dysesthesia of the hands, feet and perioral region. Subjects treated with oxaliplatin in this study should be advised to avoid cold drinks and exposure to cold water or air, especially within 3 to 5 days of receiving oxaliplatin. Dose modifications for oxaliplatin related to neurotoxicity are presented in Table 8.

Cases of posterior reversible encephalopathy syndrome (PRES) have been reported in subjects receiving Oxaliplatin combination chemotherapy. Discontinue Oxaliplatin if PRES is suspected. Confirm PRES diagnosis by brain imaging, preferably by magnetic resonance imaging (MRI).

Table 8 Oxaliplatin Dose Modification for Associated Neurotoxicity

<table>
<tr><th>Toxicity/Duration</th><th>Grade 1</th><th>Grade 2</th><th>Grade 3</th><th>Grade 4</th></tr>
<tr><td>Paresthesia or dysesthesia</td><td>Paresthesia or dysesthesia‡ that does not interfere with function</td><td>Paresthesia or dysesthesia‡, interfering with function, but not activities of daily living</td><td>Paresthesia or dysesthesia‡ with pain or with functional impairment that also interferes with activities of daily living</td><td>Persistent paresthesia or dysesthesia that is disabling or life-threatening</td></tr>
<tr><td>1 to 7 Days</td><td rowspan="3">No dose reduction</td><td rowspan="2">No dose reduction</td><td>First Event: Reduce oxaliplatin by 1 dose level at next treatment. Second Event: Reduce oxaliplatin by a second dose level at next treatment.</td><td rowspan="3">Discontinue oxaliplatin</td></tr>
<tr><td>> 7 Days</td><td>Discontinue oxaliplatin</td></tr>
<tr><td>Persistent between treatments†</td><td>Reduce oxaliplatin by 1 dose level at next treatment</td><td>Discontinue oxaliplatin</td></tr>
<tr><td>Acute laryngopharyngeal dysesthesia‡ (during or after the 2-hour infusion)</td><td colspan="4">Discontinue current infusion.
At next treatment, increase duration of infusion to 6 hours; may also pretreat with benzodiazepines</td></tr>
</table>

NA: not applicable
† Not resolved by the beginning of the next treatment.
‡ May be cold induced.

Oxaliplatin has also been associated with laryngopharyngeal dysesthesia, which is an unusual loss of sensation of breathing (acute respiratory distress) without any objective evidence of respiratory distress (hypoxia, laryngospasm or bronchospasm). Laryngopharyngeal dysesthesia may be induced or exacerbated upon exposure to cold.

Subjects developing laryngopharyngeal dysesthesia should have their oxygen saturation evaluated via a pulse oximeter. If results are normal, reassurance should be provided, a benzodiazepine or other anxiolytic agent should be considered, and the subject should remain for observation in the clinic until the episode has resolved. After resolution, the oxaliplatin infusion may then be continued at one-third the rate.

Because laryngopharyngeal dysesthesia may be associated with the rate of oxaliplatin infusion, subsequent infusions of oxaliplatin should be prolonged from a normal 2-hour infusion to a 6-hour infusion.

Subjects receiving oxaliplatin should avoid consuming cold drinks or ice chips on day 1 of each cycle, as this may exacerbate oral or throat dysesthesia, as well as laryngopharyngeal dysesthesia. Administration of prophylactic medication such as Mg^{2+}/Ca^{2+} infusions or others is at the discretion of the investigator.

The symptoms and treatments of laryngopharyngeal dysesthesia and platinum HSRs are compared in Table 9.

Table 9 Comparison of the Symptoms and Treatment of Laryngopharyngeal Dysesthesia and Platinum Hypersensitivity Reactions

Clinical Symptoms	Laryngopharyngeal Dysesthesia	Platinum Hypersensitivity
Dyspnea	Present	Present
Bronchospasm	Absent	Present
Laryngospasm	Absent	Present
Anxiety	Present	Present
O_2 saturation	Normal	Decreased
Difficulty swallowing	Present (loss of sensation)	Absent
Pruritus	Absent	Present
Urticaria/rash	Absent	Present
Cold-induced symptoms	Yes	No
Blood pressure	Normal or increased	Normal or decreased
Treatment	Reassurance, anxiolytics, observation in a controlled clinical setting until symptoms abate or at the physician’s discretion	Oxygen, steroids, epinephrine, bronchodilators; fluids and vasopressors, if appropriate

5.1.2.9 Allergic Reaction to Oxaliplatin

Subjects developing grade 1 or 2 allergic reaction to oxaliplatin should receive premedication according to institutional practice prior to further administration of oxaliplatin. Appropriate premedication should also be given if grade 1 to 2 allergic reaction persists into the next cycle. Oxaliplatin should be discontinued in subjects developing grade 3 to 4 allergic reactions.

Oxaliplatin should be interrupted pending further investigation in subjects experiencing respiratory symptoms indicative of pulmonary fibrosis such as nonproductive cough, dyspnea, crackles, rales, hypoxia, tachypnea or radiological pulmonary infiltrates. Oxaliplatin should be permanently discontinued in subjects with confirmed interstitial pulmonary fibrosis.

5.1.2.10 Extravasation of Oxaliplatin

Necrosis has been seen in conjunction with extravasation of oxaliplatin. Subjects with suspected extravasation should have their infusion stopped and the drug administered at another site. Extravasation should be treated according to institutional guidelines.

5.1.3 Treatment Compliance

The dose and schedule of IMAB362/placebo and CAPOX administered to each subject will be recorded on the appropriate eCRF at every cycle. Reasons for dose delay or omission will also be recorded. This information will be used to assess compliance with the treatment. Subjects will be provided with Capecitabine tablets for self-administration and are asked to return any unused tablets and empty packaging at the next scheduled visit for accountability.

5.1.4 Criteria for Continuation of Treatment

IMAB362 may be made available after conclusion of the study to subjects who are still receiving and benefitting from study treatment until study defined treatment discontinuation criterion is met in countries where the drug does not have marketing approval nor is commercially available.

5.1.4.1 Discontinuation of CAPOX (both components) and Continuation of IMAB362/Placebo

If a subject discontinues CAPOX (or either component of CAPOX) due to any reason other than disease progression as confirmed by IRC, they may continue on IMAB362/placebo at the discretion of the investigator provided that all of the following have been met:

- the subject completed at least 2 cycles of CAPOX treatment;
- the subject received no other chemotherapy during the study treatment period; and
- in the investigator’s opinion the subject continues to derive clinical benefit with acceptable toxicity.

Subjects should continue to follow the Study Treatment Period schedule of assessments.

5.1.4.2 Discontinuation of IMAB362/Placebo and Continued of CAPOX (one or both components)

If IMAB362/placebo is permanently discontinued first for reasons other than PD as confirmed by IRC and no other anti-cancer treatment is started, subjects may continue to receive CAPOX/Capecitabine until treatment discontinuation criteria are met. Subjects should continue to follow the Study Treatment Period schedule of assessments.

5.1.4.3 If Both IMAB362/Placebo and CAPOX (both components) are discontinued

If both IMAB362/placebo and CAPOX (both components) are permanently discontinued for reasons other than PD as confirmed by IRC, the subject should enter the Post-Treatment Follow-Up Period and continue to undergo imaging assessments per Schedule of Assessments.

5.1.5 Previous and Concomitant Treatment (Medication and Non-Medication Therapy)

All medications and concomitant treatments administered from 28 days prior to day 1 through the 90-day safety follow-up visit must be recorded in the eCRF. Documentation will include the medication name, indication, route and dates of administration.

Prohibited Concomitant Treatment

The following are strictly prohibited:

- Sorivudine or analogs (during capecitabine treatment)
- Systemic immunosuppressive agents:
  - Concurrent systemic immunosuppressive therapy, in particular systemic corticosteroids, should be stopped 14 days prior to first dose of study treatment.
  - Subjects are allowed to use a physiologic replacement dose of hydrocortisone or its equivalent (defined as up to 30 mg per day of hydrocortisone or up to 10 mg per day of prednisone).
- Live vaccines should be avoided during the treatment period in which subject is receiving capecitabine and up to 6 months after final capecitabine dose.
- Other systemic chemotherapy, immunotherapy, radiotherapy, herbal medications or other treatments intended for antitumor activity. Palliative radiotherapy for peripheral bone metastases is allowed.
- Investigational products or therapy other than IMAB362.

Cautionary Concomitant Treatment

Considerations should be given to avoid or minimize the use of the following concomitant medications, if possible, during IMAB362/placebo treatment:

- Systemic corticosteroids, because their impact on the potential efficacy of IMAB362 is not known.
  - Systemic corticosteroids should be avoided or minimized while subject is on study treatment unless required for management of an emergent medical condition (e.g., severe nausea/vomiting or hypersensitivity reaction).
  - For a subject's first dose of IMAB362/placebo, it is recommended that the prophylactic use of corticosteroids be avoided.
  - Inhaled, intranasal and topically applied steroids are allowed.
- Avoid the class of 5-HT3 blockers in subjects with congenital long QT syndrome. Administer these drugs with caution in subjects who have or may develop QTc prolongation.
- Nonsteroidal anti-inflammatory drugs (NSAIDs) because of the potential to cause gastric ulcers and covert bleeding.
  - In such cases where NSAID use is necessary, the use of NSAIDs with lower gastric ulcerogenic potential is preferred and concomitant gastric protection with proton pump inhibitors is recommended.

The following should be avoided or used with caution and closely monitored during CAPOX administration:

- Cytochrome P450 2C9 substrates (Subjects taking coumarin-derivative anticoagulants concomitantly with capecitabine should have PT/INR monitored regularly and anticoagulant dose adjusted accordingly).
- Anti-epileptic medications (e.g., phenobarbital, phenytoin and primidone)

5.2 Demographics and Baseline Characteristics

5.2.1 Demographics

Demographic information will be collected for all subjects and will include initials (where permitted), age, date of birth (where permitted), sex, race (where permitted) and ethnicity (where permitted).

5.2.2 Medical History

Medical history includes all significant medical conditions per the judgment of the investigator that have resolved prior to informed consent or are ongoing at the time of consent. Details that will be collected include the onset date and recovery date and CTCAE grade, if applicable for ongoing conditions.

5.2.3 Diagnosis of the Target Disease, Severity, and Duration of Disease

A complete medical history of the target disease will be recorded during Screening. This will include the subject's medical condition, date of initial diagnosis, tumor location, and other disease specific information as designated in the eCRF.

5.3 Efficacy Assessments

Radiologic imaging will be evaluated at Screening (within 28 days prior to C1D1) and every 9 weeks (±7 days) counting from C1D1 for the first 54 weeks and then every 12 weeks (±7 days) thereafter until subject develops radiological disease progression per RECIST 1.1 by IRC or starts other systemic anticancer treatment, whichever comes earlier.

All measurable disease must be documented at Screening and re-assessed at each subsequent radiologic evaluation. Imaging will include computerized tomography (CT) scans with contrast of the thorax, abdomen, and pelvis. If CT scan with contrast is medically not feasible, MRI may be used for imaging. Bone scans (or focal X-ray) or brain imaging should be performed if metastatic disease in bone or brain is suspected, respectively. The same mode of imaging should be utilized throughout the study unless medical necessity requires a change. All imaging will be sent to the sponsor designated facility within 7 days of scanning for the blinded IRC assessment of radiological tumor response based on RECIST 1.1 [Eisenhauer et al, 2009]. The investigator should make every effort to immediately submit radiologic assessments for IRC review when PD is suspected.

5.4 Safety Assessment

5.4.1 Vital Signs

Vital signs, including systolic and diastolic blood pressures (mmHg) and radial pulse rate (beats/minute) and temperature, will be obtained and recorded at the times specified in the Schedule of Assessments [Table 1]. All vital sign measurements will be obtained in a consistent manner (sitting or supine) throughout their study participation. Height and weight will be measured using normal institutional standards.

If clinically significant vital sign changes from baseline (pretreatment) are noted, the changes will be documented as AEs on the AE page of the eCRF. Clinical significance will be defined as a variation in vital signs, which has medical relevance as deemed by the investigator that could result in an alteration in medical care. For clinically significant vital sign changes, the investigator will continue to monitor the subject until the parameter returns to Grade ≤ 1, or to the baseline (pretreatment) value, or until the investigator determines that follow up is no longer medically necessary.

5.4.2 Observation Period following IMAB362/Placebo Infusion

Following the first dose of IMAB362/placebo on C1D1, the subject must be observed for 2 hours post IMAB362/placebo infusion. The post-infusion observation period can be conducted during the CAPOX administration. If AEs are observed during this time, subsequent IMAB362/placebo infusion times should be extended and subjects should continue to be observed for 2 hours post IMAB362/placebo infusion. If the subject does not develop any AEs, the subject should be observed for 1 hour post-infusion for their subsequent IMAB362/placebo infusions. The subject should be instructed to notify site personnel if they develop any AEs during this post-infusion observation time period.

In the event of a Grade 3 or 4 IRR, additional samples should be collected as follows:

- Once the subject has been stabilized, blood for cytokine/chemokine panel should be collected for shipment to central laboratory.
- If the reaction is suggestive of anaphylaxis, blood for serum total tryptase level (levels typically peak within 3 hours after the onset of symptoms) should collected for shipment to the central laboratory.

5.4.3 Laboratory Assessments

Below is a table of the laboratory tests that will be performed during the conduct of the study.

Laboratory tests will be performed according to the Schedule of Assessments [Table 1] and must be sent to the central laboratory for analysis.

Central laboratory results must be used to confirm eligibility. The screening labs used to determine eligibility should be collected within 14 days prior to C1D1. In situations where laboratory results are outside of the permitted range, the investigator may opt to retest the subject and subsequent within range screening central lab results may be used to confirm eligibility. In case of multiple central laboratory data within the Screening period, the most

recent data should be used to confirm eligibility. Subjects requiring transfusions to meet eligibility criteria are not eligible.

Laboratory tests will be reviewed by the investigator prior to each infusion. In the event that the central laboratory results are not available in time for treatment decisions, local certified laboratory tests may be used.

Holidays and weekends should be taken into account when scheduling these blood draws.

Additional assessments may be done centrally or locally to monitor AEs or as clinically indicated. Clinical significance of out-of-range laboratory findings is to be determined and documented by the investigator/sub-investigator who is a qualified physician.

**Panel/Assessment**	**Parameters to be Analyzed**
Hematology	Hematocrit (Hct)<br>Hemoglobin (Hgb)<br>Red Blood Cell Count (RBC)<br>White Blood Cell Count (WBC)<br>WBC differential<br>Absolute Neutrophil Count (ANC)<br>Platelets<br>Mean Corpuscular Volume (MCV)<br>Mean Corpuscular Hemoglobin (MCH)<br>Mean Corpuscular Hemoglobin Concentration (MCHC)
Biochemistry	Albumin<br>Blood Urea Nitrogen (BUN)<br>Calcium<br>Bicarbonate<br>Chloride<br>Creatinine<br>Glucose<br>Magnesium<br>Phosphate<br>Potassium<br>Sodium<br>Total Bilirubin<br>Total Protein<br>Alanine Aminotransferase (ALT)<br>Alkaline Phosphatase (ALP)<br>Aspartate Aminotransferase (AST)
Table continued on next page	

**Panel/Assessment**	**Parameters to be Analyzed**
Urinalysis	Color<br>Clarity/turbidity<br>pH<br>Specific gravity<br>Glucose<br>Ketones<br>Nitrites<br>Leukocyte esterase<br>Bilirubin<br>Urobilirubin<br>Blood<br>Protein<br>RBCs<br>WBCs
Coagulation	Prothrombin time (PT) (sec)<br>Partial Thromboplastin Time (PTT)<br>International normalized ratio (INR)
Thyroid Function Test	Thyroid stimulating hormone (TSH)<br>T4 (thyroxine)
Serum Pregnancy Test	Human chorionic gonadotropin (HCG)

5.4.4 Physical Examination

Physical examinations will be conducted at visits as outlined in the Schedule of Assessments [Table 1]. Each physical exam should include height (at Screening only), weight and ECOG performance status. A full physical exam is required at Screening. The physical exam only needs to be repeated on C1D1 if clinically significant changes from Screening are observed (in the opinion of the investigator). Targeted (symptom driven) physical exams should be conducted every 3 weeks on day 1 of each cycle. If clinically significant worsening of findings from baseline is noted at any study visit, the changes will be documented as AEs on the AE eCRF. Clinical significance is defined as any variation in physical findings, which has medical relevance that could result in an alteration in medical care. The investigator will continue to monitor the subject until the parameter returns to grade ≤ 1, or to the baseline condition, or until the investigator determines that follow up is no longer medically necessary.

5.4.5 Electrocardiogram

A single 12-lead ECG will be performed at the time points outlined in the Schedule of Assessments [Table 1]. Prior to performing ECG, subjects should rest in supine position for 10 minutes. When collected on the same day, ECG should be collected prior to pharmacokinetic samples. Additional ECG may be performed based on medical history and investigator medical judgment. ECGs will be assessed locally. Clinically significant findings or changes from baseline should be recorded as an AE.

5.4.6 Performance Status

The ECOG Scale [Oken, 1982] will be used to assess performance status. Refer to [Appendix 12.8].

5.5 Adverse Events and Other Safety Aspects

AE collection will begin from time of informed consent and continue through the 30 days following the last dose of IMAB362/placebo and CAPOX (bothcomponents), or until initiation of a subsequent anticancer therapy.

Serious adverse events (SAEs), regardless of causality will be collected from the time of informed consent through 90 days following the last dose of IMAB362/placebo and CAPOX (both components) or until initiation of a subsequent anti-cancer treatment, whichever comes first.

AEs will be documented at each clinic visit, but can be collected at any time. Any AE that meets the definition of an SAE will also be reported on a separate form to the sponsor.

5.5.1 Definition of Adverse Events

An AE is any untoward medical occurrence in a subject administered a study drug, and which does not necessarily have to have a causal relationship with this treatment. An AE can therefore be any unfavorable and unintended sign (including an abnormal laboratory finding), symptom, or disease (new or exacerbated) temporally associated with the use of a medicinal product whether or not considered related to the medicinal product.

In order to identify any events that may be associated with study procedures and could lead to a change in the conduct of the study, Astellas collects AEs even if the subject has not received study drug treatment. AE collection begins after the signing of the informed consent and will be collected until 30 days after the last dose of study drug or the subject is determined to be a screen failure.

Care will be taken not to introduce bias when detecting AEs and/or SAEs. Open-ended and non-leading verbal questioning of the subject is the preferred method to inquire about AE occurrences.

5.5.1.1 Abnormal Laboratory Findings

Any abnormal laboratory test result (e.g., hematology, clinical chemistry or urinalysis) or other safety assessment (e.g., ECGs, radiology scans, vital signs measurements, physical examination), including those that worsen from baseline, that is considered to be clinically significant in the medical and scientific judgment of the investigator and not related to underlying disease, is to be reported as an (S)AE.

Any clinically significant abnormal laboratory finding or other abnormal safety assessment which is associated with the underlying disease does not require reporting as an (S)AE, unless judged by the investigator to be more severe than expected for the subject’s condition.

Repeating an abnormal laboratory test or other safety assessment, in the absence of any of the above criteria, does not constitute an AE. Any abnormal test result that is determined to be an error does not require reporting as an AE.

5.5.1.2 Potential Cases of Drug-Induced Liver Injury

Refer to [Appendix 12.4 Liver Safety Monitoring and Assessment] for detailed instructions on drug induced liver injury. Abnormal values in AST and/or ALT concurrent or with abnormal elevations in total bilirubin that meet the criteria outlined in [Appendix 12.4 Liver Safety Monitoring and Assessment] in the absence of other causes of liver injury, are considered potential cases of drug-induced liver injury (potential Hy's Law cases) and are always to be considered important medical events and reported per [Section 5.5.5 Reporting of Serious Adverse Events].

5.5.1.3 Disease Progression and Study Endpoints

Under this protocol, the following event(s) will not be considered as an (S)AE:

- Disease Progression: events including defined study endpoints that are clearly consistent with the expected pattern of progression of the underlying disease are not to be recorded as AEs unless resulting in death. These data will be captured as efficacy assessment data as outlined in [Section 5.3 Efficacy Assessments]. If there is any uncertainty as to whether an event is due to anticipated disease progression and/or if there is evidence suggesting a causal relationship between the study treatment and the event, it should be reported as an (S)AE.
- Disease progression can be considered as the worsening of a subject's condition attributable to Gastric or GEJ cancer. It may be an increase in the severity of the disease under study and/or increases in the symptoms of the disease. The development of new, or progression of existing metastases to the primary cancer under study should be considered as disease progression not an AE. Events which are unequivocally due to disease progression should not be reported as an AE during the study.

5.5.2 Definition of Serious Adverse Events

An AE is considered "serious" if, in the view of either the investigator or sponsor, it results in any of the following outcomes:

- Results in death
- Results in life-threatening event (an AE is considered "life-threatening" if, in the view of either the investigator or sponsor, its occurrence places the subject at immediate risk of death. It does not include an AE that, had it occurred in a more severe form, might have caused death)
- Results in persistent or significant disability/incapacity or substantial disruption of the ability to conduct normal life functions
- Results in congenital anomaly, or birth defect

- Requires inpatient hospitalization (except for planned procedures as allowed per study) or leads to prolongation of hospitalization (except if prolongation of planned hospitalization is not caused by an AE). Hospitalization for treatment/observation/ examination caused by AE is to be considered as serious.
- Other medically important events (defined in paragraph below)

Medical and scientific judgment should be exercised in deciding whether expedited reporting is appropriate in other situations, such as important medical events that may not be immediately life-threatening or result in death or hospitalization but may jeopardize the subject or may require intervention to prevent one of the other outcomes listed in the definition above. These events, including those that may result in disability/incapacity, usually are considered serious. Examples of such events are intensive treatment in an emergency room or at home for allergic bronchospasm; blood dyscrasias or convulsions that do not result in hospitalization; or development of drug dependency or drug abuse.

5.5.2.1 Always Serious Adverse Events

The sponsor has a list of events that they classify as “always serious” events. If an AE is reported that is considered by the sponsor to be an SAE per this classification as “always serious”, additional information on the event (e.g. investigator confirmation of seriousness, causality) will be requested.

Progression of Gastric or GEJ cancer, including signs and symptoms of progression, should not be reported as an SAE unless it results in death within 90 days of the last dose of study treatment. For progression-related death reported as an SAE, there should be available immediate cause of death reported as the event term. “Death due to disease progression” should be recorded as the AE term only when the cause of death cannot be otherwise determined.

5.5.3 Criteria for Causal Relationship to Study Treatment

A medically qualified investigator is obligated to assess the relationship between each study treatment and each occurrence of each (S)AE. This medically qualified investigator will use medical judgment as well as the RSI [Section 1.3] to determine the relationship. The causality assessment is 1 of the criteria used when determining regulatory reporting requirements. The medically qualified investigator is requested to provide an explanation for the causality assessment for each (S)AE and must document in the medical notes that he/she has reviewed the (S)AE and has provided an assessment of causality.

Following a review of the relevant data, the causal relationship between the study treatment and each (S)AE will be assessed by answering ‘**yes**’ or ‘**no**’ to the question “**Do you consider that there is a reasonable possibility that the event may have been caused by the study treatment**”.

When making an assessment of causality, the following factors are to be considered when deciding if there is evidence and/or arguments to suggest there is a ‘reasonable possibility’

that an (S)AE may have been caused by the study treatment (rather than a relationship cannot be ruled out) or if there is evidence to reasonably deny a causal relationship:

- Plausible temporal relationship between exposure to the study treatment and (S)AE onset and/or resolution. Has the subject actually received the study treatment? Did the (S)AE occur in a reasonable temporal relationship to the administration of the study treatment?
- Plausibility; i.e., could the event have been caused by the study treatment? Consider biologic and/or pharmacologic mechanism, half-life, literature evidence, drug class, preclinical and clinical study data, etc.
- Dechallenge/Dose reduction/Rechallenge:
  - Did the (S)AE resolve or improve after stopping or reducing the dose of the suspect drug? Also consider the impact of treatment for the event when evaluating a dechallenge experience.
  - Did the (S)AE reoccur if the suspected drug was reintroduced after having been stopped?
- Laboratory or other test results; a specific lab investigation supports the assessment of the relationship between the (S)AE and the study treatment (e.g., based on values pre-, during and post-treatment)
- Available alternative explanations independent of study treatment exposure; such as other concomitant drugs, past medical history, concurrent or underlying disease, risk factors including medical and family history, season, location, etc. and strength of the alternative explanation

There may be situations in which an SAE has occurred and the investigator has minimal information to include in the initial report to the sponsor. However, it is very important that the medically qualified investigator always make an assessment of causality for every event before the initial transmission of the SAE data to the sponsor. With limited or insufficient information about the event to make an informed medical judgment and in absence of any indication or evidence to establish a causal relationship, a causality assessment of ‘no’ is to be considered. In such instance, the investigator is expected to obtain additional information regarding the event as soon as possible and to re-evaluate the causality upon receipt of additional information. The medically qualified investigator may revise his/her assessment of causality in light of new information regarding the SAE and shall send an SAE follow-up report and update the eCRF with the new information and updated causality assessment.

5.5.4 Criteria for Defining the Severity of an Adverse Event

AEs, including abnormal clinical laboratory values, will be graded using the NCI-CTCAE guidelines. The items that are not stipulated in the NCI-CTCAE will be assessed according to the criteria below and entered into the eCRF.

Grade	Assessment Standard
1-Mild	Asymptomatic or mild symptoms, clinical or diagnostic observations noted; intervention not indicated.
2-Moderate	Local or noninvasive intervention indicated.
3-Severe	Medically significant but not immediately life threatening, hospitalization or prolonged hospitalization.
4-Life Threatening	Life threatening consequences, urgent intervention indicated
5-Death	Death related to AE

5.5.5 Reporting of Serious Adverse Events

The collection of AEs and the expedited reporting of SAEs will start following receipt of the signed informed consent and will continue until 90 days (30 days for AEs) after the last dose of study treatment or the subject is determined to be a screen failure.

In the case of a SAE, the investigator must contact the sponsor by fax or email immediately (within 24 hours of awareness).

The investigator must complete and submit an SAE worksheet containing all information that is required by local and/or regional regulations to the sponsor by email or fax immediately (within 24 hours of awareness).

The SAE worksheet must be signed by a medically qualified investigator (as identified on Delegation of Authority Log). Signature confirms accuracy and completeness of the SAE data as well as the investigator causality assessment including the explanation for the causality assessment.

If the SAE is associated with emergency unblinding as outlined in [Section 4.6.4 Breaking the Blind in Emergency] this is to be recorded on the SAE worksheet. Within the SAE worksheet, the investigator is to include when unblinding took place in association with the SAE.

JAPAN SITES ONLY**:** In the case of a SAE, the investigator or sub-investigator must report to the head of the study site and must contact the sponsor by email or fax immediately (within 24 hours of awareness). The investigator should complete and submit JUTOKUNA YUUGAIJISHOU HOUKOKUSHO containing all information that is required by the Regulatory Authorities to the sponsor by email or fax immediately (within 24 hours of awareness) and to the head of the hospital.

For contact details, see [Section II Contact Details of Key Sponsor's Personnel]. Fax or email the SAE and Special Situations Worksheet to:

Astellas Pharma Global Development, Inc.
Pharmacovigilance
North American Fax: 888-396-3750
(North America Alternate Fax: 847-317-1241)
International Fax: +44-800-471-5263
Email: safety-us@astellas.com

***UNIQUE TO JAPAN REGION**:* JUTOKUNA YUUGAIJISHOU HOUKOKUSHO the SAE and Special Situations Worksheet to:

PAREXEL International
Fax number: 03-6888-1686

If there are any questions, or if clarification is needed regarding the SAE, please contact the sponsor's medical monitor/Study Physician or his/her designee [Section II Contact Details of Key Sponsor's Personnel].

Follow-up information for the event should be sent promptly (within 7 days of the initial notification (***UNIQUE TO JAPAN REGION*****:** within 2 days for the initial notification).

Full details of the SAE or Special Situation should be recorded on the medical records, SAE or Special Situation Worksheet and on the (e)CRF.

The following minimum information is required:

- International Study Number (ISN)/Study number,
- Subject number, sex and age,
- The date of report,
- A description of the SAE (event, seriousness criteria),
- Causal relationship to the study treatment (including reason), and
- The drug provided (if any). Note: blinded regimen is also an option.

The sponsor or sponsor's designee will medically evaluate the SAE and determine if the report meets the requirements for expedited reporting based on seriousness, causality, and expectedness of the events (e.g., SUSAR reporting) according to current local/regional regulatory requirements in participating countries. The sponsor or sponsor's designee will submit expedited safety reports (e.g., Investigational New Drug [IND] Safety Reports, SUSAR, Council for International Organizations of Medical Sciences I Form) to Competent Authorities and concerned Ethics Committee per current local regulations, and will inform the investigators of such regulatory reports as required. Investigators must submit safety reports as required by their IRB/local IEC within timelines set by regional regulations (e.g., EMA, FDA) where required. Documentation of the submission to and receipt by the IRB/local IEC of expedited safety reports should be retained by the study site.

The sponsor will notify all investigators responsible for ongoing clinical studies with the study treatment of all SUSARs which require submission per local requirements IRB/local IEC/ head of the study site (as applicable).

The heads of the study sites (if applicable) and investigators should provide written documentation of IRB/IEC notification for each report to the sponsor.

5.5.6 Follow-up of Adverse Events

All AEs occurring during or after the subject has discontinued the study are to be followed up until resolved or judged to be no longer clinically significant, or until they become chronic to the extent that they can be fully characterized by the investigator.

If after the protocol defined AE collection period [see Section 5.5.1 Definition of Adverse Event], an AE progresses to an SAE, or the investigator learns of any (S)AE including death, where he/she considers there is reasonable possibility it is related to the study treatment or study participation, the investigator must promptly notify the sponsor.

5.5.7 Monitoring of Common Serious Adverse Events

Common SAEs are SAEs commonly anticipated to occur in the study population independent of drug exposure. SAEs classified as “common” are provided in [Appendix 12.6 Common Serious Adverse Events] for reference. The list does NOT change the investigator’s reporting obligations, nor his obligations to perform a causality assessment, or prevent the need to report an AE meeting the definition of an SAE as detailed above. The purpose of this list is to alert the investigator that some events reported as SAEs may not require expedited reporting to the regulatory authorities based on the classification of “common SAEs” as specified in [Appendix 12.6 Common Serious Adverse Events]. The sponsor will monitor these events throughout the course of the study for any change in frequency. Any changes to this list will be communicated to the participating investigational sites. Investigators must report individual occurrences of these events as stated in [Section 5.5.5 Reporting of Serious Adverse Events].

5.5.8 Adverse Events of Special Interest

In case of IMAB362 induced nausea, vomiting or hypersensitivity/IRR, infusion rate of IMAB362/placebo may be reduced or infusion paused or discontinued based on investigator’s clinical judgment about severity of toxicity and local standard of care. See [Section 5.1.2 Study Treatment Dose Modifications, Delays and Interruption].

If the AEs of interest are classified as serious, they are to be collected via the SAE worksheet and reported within 24 hours as described in [Section 5.5.5 Reporting of Serious Adverse Events].

5.5.9 Special Situations

Certain Special Situations observed in association with the study treatment(s), such as incorrect administration (e.g., wrong dose of study treatment, comparator or background therapy) are collected in the eCRF, as Protocol Deviations per [Section 8.2 Major Protocol

Deviations] or may require special reporting, as described below. These Special Situations are not considered AEs, but do require to be communicated to Astellas as per the timelines defined below.

If a Special Situation is associated with, or results in, an AE, the AE is to be assessed separately from the Special Situation and captured as an AE in the eCRF. If the AE meets the definition of a SAE, the SAE is to be reported as described in [Section 5.5.5 Reporting of Serious Adverse Events] and the details of the associated Special Situation are to be included in the clinical description on the Special Situation worksheet.

The Special Situations are:

- Pregnancy
- Medication error, Overdose and "Off label use"
- Misuse/abuse
- Occupational exposure
- (Suspicion of) Transmission of infectious agent
- Suspected Drug-Drug interaction

5.5.9.1 Pregnancy

If a female subject or partner of a male subject becomes pregnant during the study dosing period or within 6 months from the discontinuation of dosing, the investigator is to report the information to the sponsor according to the timelines in [Section 5.5.5 Reporting of Serious Adverse Events] using the Pregnancy Reporting Form and in the eCRF.

The expected date of delivery or expected date of the end of the pregnancy, last menstruation, estimated conception date, pregnancy result and neonatal data etc., should be included in this information.

While pregnancy itself is not considered to be an AE or SAE, any pregnancy complication or termination (including elective termination) of a pregnancy is to be reported for a female study subject as an AE in the eCRF or SAE per [Section 5.5.5 Reporting of Serious Adverse Events]. For (S)AEs experienced by a female partner of a male subject, (S)AEs are to be reported via the Pregnancy Reporting Form.

Additional information regarding the outcome of a pregnancy when also categorized as an SAE is mentioned below:

- "Spontaneous abortion" includes miscarriage, abortion and missed abortion.
- Death of a newborn or infant within 1 month after birth is to be reported as an SAE regardless of its relationship with the study drug.
- If an infant dies more than 1 month after the birth, is to be reported if a relationship between the death and intrauterine exposure to the study drug is judged as "possible" by the investigator.
- Congenital anomaly (including anomaly in miscarried fetus)

Unless a congenital anomaly is identified prior to spontaneous abortion or miscarriage, the embryo or fetus should be assessed for congenital defects by visual examination. (S)AEs experienced by the newborn/infant should be reported via the Pregnancy Reporting Form. Generally, follow up will be no longer than 6 to 8 weeks following the estimated delivery date.

5.5.9.2 Medication Error, Overdose and "Off-Label Use"

If a Medication Error, Overdose or "Off-label Use" (i.e., use outside of what is stated in the protocol) is suspected, refer to [Section 8.2 Major Protocol Deviations]. Any associated (S)AEs are to be reported in the eCRF. If the AE meets the definition of a SAE, the SAE is also to be reported as described in [Section 5.5.5 Reporting of Serious Adverse Events] together with the details of the medication error, overdose and/or "Off-Label Use".

There is no antidote for overdose of the study drug. In the event of suspected IMAB362 overdose, the subject should receive supportive care and monitoring (including, but not limited to, inpatient hospitalization). The medical monitor should be contacted as applicable.

In the event of suspected CAPOX overdose, refer to the approved package insert, SPC or local product information supplied by the manufacturer for each agent.

5.5.9.3 Misuse/Abuse

If misuse or abuse of the study drug(s) is suspected, the investigator must forward the Special Situation worksheet to the sponsor/delegated contract research organization (CRO) by fax or email immediately (within 24 hours of awareness). Any associated (S)AEs are to be reported in the eCRF. If the AE meets the definition of a SAE, the SAE is also to be reported as described in [Section 5.5.5 Reporting of Serious Adverse Events] together with details of the misuse or abuse of the study drug(s).

5.5.9.4 Occupational Exposure

If occupational exposure (e.g., inadvertent exposure to the study drug(s) of site staff whilst preparing it for administration to the subject) to the study drug(s) occurs, the investigator must forward the Special Situation worksheet to the sponsor/delegated CRO by fax or email immediately (within 24 hours of awareness). Any associated (S)AEs occurring to the individual associated with or resulting from the Special Situation are to be reported on the Special Situations worksheet.

5.5.9.5 Suspected Drug-Drug Interaction

If a suspected drug-drug interaction associated with the study drug(s) is suspected, the investigator must forward the Special Situation worksheet to the sponsor/delegated CRO by fax or email immediately (within 24 hours of awareness). Any associated (S)AEs are to be reported in the eCRF. If the AE meets the definition of a SAE, the SAE is also to be reported as described in [Section 5.5.5 Reporting of Serious Adverse Events] together with details of the suspected drug-drug interaction.

5.5.10 Supply of New Information Affecting the Conduct of the Study

When new information becomes available necessary for conducting the clinical study properly, the sponsor will inform all investigators involved in the clinical study as well as the regulatory authorities. Investigators should inform the IRB/IEC of such information when needed.

The investigator will also inform the subjects, who will be required to sign an updated informed consent form (ICF) in order to continue in the clinical study.

UNIQUE TO JAPAN REGION:

1. When information is obtained regarding serious and unexpected adverse drug reactions (or other) that are specified in Article 273 of the Act on Securing Quality, Efficacy and Safety of Pharmaceuticals, Medical Devices, Regenerative and Cellular Therapy Products, Gene Therapy Products, and Cosmetics, in compliance with Article 80-2 Paragraph 6 of the Pharmaceutical Affairs Law, the sponsor should inform all the investigators involved in the clinical study, the head of the study site, and the regulatory authorities of such information. The head of the study site who receives such information will decide whether the clinical study should be continued after hearing the opinions of the IRB. The investigator will supply the new information to the subjects, in compliance with [Appendix 12.1.4.2 Supply of New and Important Information Influencing the Subject’s Consent and Revision of the Written Information].
2. In addition to the above item (1), when the head of the study site receives the revisions of the IB, protocol or written information, information on the matters covering the quality of the study drug, efficacy and safety, information necessary for conducting the clinical study properly, or documents to be examined by the IRB these documents should be sent to the IRB.

5.5.11 Urgent Safety Measures

An Urgent Safety Measure (USM) is an intervention, which is not defined by the protocol and can be put in place with immediate effect without needing to gain prior approval by the sponsor, relevant Competent Authorities, IRB/IEC, where applicable, in order to protect study participants from any immediate hazard to their health and/or safety. Either the investigator or the sponsor can initiate a USM. The cause of a USM can be safety, product or procedure related.

5.5.12 Reporting Urgent Safety Measures

In the event of a potential USM, the investigator must contact the Astellas Study Physician and/or – unique for JP region – Astellas team member (within 24 hrs of awareness). Full details of the potential USM are to be recorded in the subject’s medical records. The sponsor may request additional information related to the event to support their evaluation.

If the event is confirmed to be an USM the sponsor will take appropriate action to ensure the safety and welfare of the patients. These actions may include but are not limited to a change in study procedures or study treatment, halting further enrollment in the study, or stopping

the study in its entirety. The sponsor or sponsor's designee will notify CA and cEC within the timelines required per current local regulations, and will inform the investigators as required. When required, investigators must notify their IRB/IEC within timelines set by regional regulations.

5.6 Test Drug Concentration

Serum concentrations of IMAB362 will be measured. Samples will be collected as outlined in the Schedule of Assessments. Blood sampling, processing, storage and shipment instructions will be provided in the Laboratory Manual. Samples will be shipped to and analyzed by a sponsor-designated analytical laboratory using validated analytical methods. Please refer to the Laboratory Manual for more detailed information.

IMAB362 in serum will be quantified using a ligand-binding assay with electrochemiluminescense (ECL) detection. Samples remaining after pharmacokinetic assessments may be used for additional biomarker analysis described in [Section 5.7.1 Biomarkers].

5.7 Other Measurements, Assessments or Methods

5.7.1 Biomarkers

Tumor tissue and blood/serum/plasma samples described in [Sections 5.7.2 Blood/Serum/ Plasma Samples and 5.7.3 Tumor Tissue Samples] may be used for research purposes to identify genomic and/or other biomarkers that may be associated with clinical outcome or dynamic changes associated with IMAB362 treatment (in terms of dose, safety, tolerability and efficacy). Since the identification of exploratory biomarkers that correlate with the efficacy or safety of IMAB362 treatment may continue to evolve as new findings becomes available, additional analyses related to IMAB362 activity on tumor signaling pathways or clinical outcomes may be conducted. Tumor tissue and blood/serum samples remaining after the specified biomarker assessments (e.g., aliquots of tumor cell RNA or DNA) may be used for re-testing, additional analyses as defined above or developing, and validating assays related to prediction of response or dynamic changes associated with IMAB362 treatment. The tumor tissue and blood/serum/plasma samples (e.g., aliquots of tumor cell RNA or DNA, peripheral blood mononuclear cells) will be stored at the study sponsors' facility or a contract laboratory facility for up to 15 years after database closure, at which time the samples will be destroyed. The procedures for the collection, handling and shipping of laboratory samples being submitted to the central laboratory will be specified in a laboratory manual.

5.7.2 Blood, Serum and Plasma Samples

Blood, serum and plasma samples will be collected according to the Schedule of Assessments for exploratory biomarker measurements. Blood, serum and plasma samples may be analyzed for biomarkers including but not limited to chemokines, cytokines, CDC activation, circulating DNA soluble factors and genetic markers.

5.7.3 Tumor Tissue Samples

FFPE tumor tissue samples will be obtained for all subjects and sent for central IHC testing to evaluate for CLDN18.2, and HER2 status if a previously documented HER2 test result is not available. Archival tumor tissue is preferred, but if the specimen is insufficient or unavailable, a biopsy may be performed to obtain a tumor sample. Sponsor preapproval is required when a biopsy procedure is needed for the sole purpose of determining study eligibility. Optional post-progression tumor tissue sample for exploratory biomarker analysis may be collected following IRC confirmation of disease progression and prior to initiation of subsequent anti-cancer therapy for subjects who sign a separate ICF. Tumor specimens may be analyzed for exploratory biomarkers including but not limited to CLDN18.2 expression, immune cells, genetic markers and gene/protein expression.

The investigator, in consultation with other specialists, as needed (e.g., radiology staff) will assess the risk associated with obtaining a tumor tissue sample and determine if the subject is an appropriate candidate for the procedure. Biopsies should be obtained in accordance with institutional policies/guidelines to minimize risk. Procedures requiring general anesthesia should not be performed to obtain a tumor tissue sample; however, if a surgical procedure under general anesthesia is performed for a clinical indication, excess tumor tissue may be used for research purposes with the consent of the subject.

Tumor Tissue Requirements

Visit	Tumor Tissue Requirement
Screening	A minimum of 1 FFPE tumor tissue block (preferred) OR a minimum of 15 FFPE unstained slides are required.<br>*If local HER2 results are available, a minimum of 12 slides are required along with the pathology report/documented test results. If local HER2 results are unavailable, follow guidance above.
Post-Progression (optional)	A minimum of 1 FFPE tumor tissue block (preferred) OR a minimum of 15 FFPE unstained slides are required.

FFPE: formalin-fixed paraffin embedded; HER2: human epidermal growth factor receptor 2

5.7.4 Immunogenicity Assessment (Anti-drug Antibody)

Serum samples to assess the formation of anti-drug antibodies (ADAs) against IMAB362 will be collected as outlined in the Schedule of Assessments [Table 1]. Blood sampling, processing, storage and shipment instructions will be provided in the Laboratory Manual. Samples will be shipped to and analyzed by a sponsor designated analytical laboratory using validated analytical methods. Please refer to the Laboratory Manual for more detailed information.

ADAs against IMAB362 in serum will be detected using a ligand-binding assay with ECL detection. A tiered approach will be followed for screening, confirming and titering the samples. Samples remaining after immunogenicity assessments may be used for additional biomarker analysis as described in [Section 5.7.1 Biomarkers].

5.7.5 Optional Samples for Banked PGx Sample Analysis

For subjects who signed a separate ICF, an optional whole blood sample for pharmacogenomics (PGx) will be collected at C1D1 prior to first study drug administration. PGx research may be conducted in the future to analyze or determine genes of relevance to clinical response, pharmacokinetics and toxicity/safety issues. A sample of whole blood for possible retrospective PGx analysis will be collected and processed. Blood sampling, processing, storage and shipment instructions will be provided in the Laboratory Manual. Samples will be shipped to a sponsor-designated analytical storage laboratory. Please refer to the Laboratory Manual for more detailed information.

See [Appendix 12.7 Pharmacogenomic Analysis With Banked Sample] for further details on the banking procedures.

5.7.6 Electronic Clinical Outcome Assessments

Subjects will be asked to complete HRQoL and HRU questionnaires as specified in the schedule of assessments. The electronic Clinical Outcomes Assessment (eCOA) questionnaires should be administered prior to the start of any study treatment, study procedure, physician assessment or disease status is discussed with the subject. Assessments will be collected at Screening, every 3 weeks, at study treatment discontinuation and 30 and 90 days post IMAB362/placebo treatment. HRQoL will be measured by EORTC QLQ-C30, QLQ-OG25 plus STO22 Belching subscale, GP and the EQ5D-5L.

5.7.6.1 Quality of Life Questionnaire C30

The EORTC-QLQ-C30 is a cancer-specific instrument consisting of 5 functional domain scales: physical, role, emotional, social and cognitive.

5.7.6.2 Oesophago-Gastric Module 25 plus STO22 Belching subscale

The EORTC-QLQ-OG25 instrument evaluates Gastric and GEJ cancer-specific symptoms such as stomach discomfort, difficulties eating and swallowing and indigestion.
Two questions from the QLQ-STO22 module related to belching will be used.

5.7.6.3 Global Pain

The GP instrument is a single assessment of overall pain.

5.7.6.4 EuroQOL Five Dimensions Questionnaire

The EQ-5D-5L is a standardized instrument for use as a measure of health outcome consisting of 6 items that cover 5 main domains (mobility, self-care, usual activities, pain/discomfort, and anxiety/depression) and a general visual analog scale for health status.

5.7.6.5 Health Resource Utilization

The HRU questionnaire is used to assess the number of office visits, hospital stays, and other healthcare resource utilization that occur outside of the clinical study.

5.8 Total Amount of Blood

The total amount of blood collected for study assessments for each subject will vary depending on how long they stay on treatment.

At any time during the study, if any laboratory abnormalities are found for a subject or for disease assessment, additional blood may be drawn for monitoring.

Additional blood beyond standard monitoring that will be drawn for this study will include draws for eligibility assessment, hematology, chemistry, and coagulation at specific study defined time points, pharmacokinetics, and biomarker sampling.

The maximum amount of blood collected is approximately 88 mL in cycle 1.

6 DISCONTINUATION

6.1 Discontinuation of Individual Subject(s) From Study Treatment

A subject who enrolled in the study and for whom study treatment (IMAB362/placebo and both components of CAPOX) is permanently discontinued for any reason will be assessed as having met study treatment discontinuation criteria.

As overall survival is the key secondary end-point of the study, all subjects will be followed for survival after meeting study treatment discontinuation criteria unless the subject withdraws consent or is considered lost to follow-up after repeated attempts to contact or if the sponsor discontinues the study. The reason for discontinuation from study treatment must be documented in the subject's medical records.

The subject will be discontinued from study treatment (IMAB362/placebo and both components of CAPOX) if any of the following occur:

- Investigator determines it is in the subject's best interest to discontinue study treatment
- Subject develops radiological disease progression per RECIST 1.1 criteria based on assessment by IRC.
    - If the investigator believes that the subject is continuing to derive clinical benefit (asymptomatic and/or without worsening of performance status or overall health) from study treatment, and an increase in tumor burden is not likely to affect vital organ function, the subject may remain on study treatment until an additional radiologic assessment is completed (≤ 9 weeks from previous radiologic assessment).
        - If the additional radiologic assessment by IRC indicates PD per RECIST 1.1, then the subject must be discontinued from study treatment.
        - If the additional radiologic assessment by IRC does not confirm the initial assessment of PD, the subject may continue to receive study treatment.
- Subject develops clinical progression per investigator assessment and radiologic assessment is not medically feasible to confirm radiologic progression due to the subject's condition.

- Subject starts another systemic chemotherapy, immunotherapy, radiotherapy or other treatment intended for antitumor activity.
- Subject starts other investigational agent or device.
- Subject develops unacceptable toxicity.
- Subject has a delay of study treatment (IMAB362/placebo and both components CAPOX) for > 28 days.
- Any clinical AE, laboratory abnormality, or inter-current illness, in the opinion of the investigator, indicates continued treatment is not in the best interest of the subject
- Female subject becomes pregnant.
- Significant deviation from the protocol or eligibility criteria as determined by the sponsor.
- Subject declines further treatment.
- Subject is lost to follow-up despite reasonable efforts by the investigator to locate the subject.
- Subject is noncompliant with the protocol based on investigator or medical monitor assessment.

Note, if a subject discontinues both components of CAPOX and IMAB362/placebo due to any reason other than IRC confirmed disease progression (and is not receiving any other anticancer therapy), the subject must be followed according to the protocol-specified radiologic assessment schedule until radiological disease progression per RECIST 1.1 criteria is confirmed by IRC assessment.

Study Discontinuation Criteria

All subjects should remain in the study through the Survival Follow-up Period (OS is a key secondary study endpoint). A subject will be discontinued from the Post-treatment, Long-term and Survival Follow-up Periods if any of the following occur:

- Subject specifically withdraws consent for any further contact with him/her or persons previously authorized by the participant to provide this information
- Subject is lost to follow-up despite reasonable efforts by the investigator to locate the subject
- Death (from any cause)
- Study termination by the sponsor

6.1.1 Lost to Follow Up

Every reasonable effort is to be made to contact any subject lost to follow-up during the course of the study to complete study-related assessments, record outstanding data, and retrieve study drug.

6.2 Discontinuation of the Site

If an investigator intends to discontinue participation in the study, the investigator must immediately inform the sponsor and, ***SPECIFIC TO SITES IN JAPAN***, the head of the study site must also be informed immediately.

6.3 Discontinuation of the Study

The sponsor may terminate this study prematurely, either in its entirety or at any study site, for reasonable cause provided that written notice is submitted in advance of the intended termination. Advance notice is not required if the study is stopped due to safety concerns. If the sponsor terminates the study for safety reasons, the sponsor will immediately notify the investigator and subsequently provide written instructions for study termination.

7 STATISTICAL METHODOLOGY

A statistical analysis plan (SAP) will be written to provide details of the analysis, along with specifications for tables, listings and figures to be produced The final SAP will be approved prior to database hardlock and unblinding the subject treatment assignment. Changes that affected the statistical analyses from the planned analyses in the SAP will be documented in the Clinical Study Report (CSR).

In general, continuous data will be summarized with descriptive statistics (number of subjects, mean, standard deviation, minimum, median and maximum) for continuous variables, and frequency and percentage for categorical variables.

Baseline will be defined as the last observation prior to first dose, unless otherwise specified.

7.1 Sample Size

Approximately 500 subjects will be randomized in a 1:1 ratio to receive IMAB362 in combination with CAPOX chemotherapy (Arm A) or placebo in combination with CAPOX chemotherapy (Arm B). The planned 344 PFS events during the study will provide 96% power to detect a difference in PFS between Arm A (IMAB362+CAPOX) with the assumption of 9 months median PFS time and Arm B (placebo+CAPOX) with the assumption of 6 months median PFS time (hazard ratio = 0.67) at the overall 1-sided 0.025 significance level. Similarly, the planned 386 OS events during the study will provide 80% power to detect a difference in OS between Arm A (IMAB362+CAPOX) with the assumption of 14.7 months median survival time and Arm B (placebo+CAPOX) with the assumption of 11 months median OS time (hazard ratio = 0.75) at the overall 1-sided 0.025 significance level.

7.2 Analysis Sets

The allocation of subjects to analysis sets will be determined prior to database hard-lock. For each treatment group, the number and percentage of subjects will be characterized for all randomized subjects and by each analysis set.

7.2.1 Full Analysis Set

The full analysis set (FAS) will consist of all subjects who are randomized to 1 of the treatment arms. Subjects would be analyzed according to the treatment they were randomized to. The FAS would be used for description of baseline characteristics and all efficacy analyses.

7.2.2 Per Protocol Set

The per protocol set (PPS) will consist of the subset of the FAS who do not meet criteria for PPS exclusion. These criteria are to capture relevant non-adherence to the protocol and will be defined in the SAP or Classification Specifications. In addition, subjects in the PPS are required to have both baseline imaging and at least 1 postbaseline imaging scan. The sensitivity analyses for the primary and select secondary endpoints will be performed on the PPS.

7.2.3 Safety Analysis Set

The safety analysis set (SAF) will consist of all subjects who received at least 1 dose of any study drug (IMAB362/placebo/CAPOX). The SAF will be used for all safety analyses. Subjects would be analyzed according to the actual treatment they received.

7.2.4 Pharmacokinetic Analysis Set (PKAS)

The pharmacokinetic analysis set (PKAS) consists of the subset of the SAF for which at least 1 concentration data is available. Additional subjects may be excluded from the PKAS at the discretion of the pharmacokineticist. The PKAS would be used for description of pharmacokinetic data.

7.3 Demographics and Baseline Characteristics

Demographics and other baseline characteristics will be summarized by treatment group and overall for FAS, PPS and SAF.

7.3.1 Subject Disposition

The number and percentage of subjects who discontinued treatment and reasons for treatment discontinuation will be presented for FAS by treatment group and overall. Similar tables for subjects who do not have a PFS event and subjects who do not have observed death will also be presented. All disposition details and dates of first and last evaluations for each subject will be listed.

7.3.2 Previous and Concomitant Medications

All previous and concomitant medications will be presented in a listing. The frequency of concomitant medications (prescription, over-the-counter, and nutritional supplements) will be summarized. Any component of CAPOX is not considered concomitant medications.

7.3.3 Medical History

Medical history for each subject will be presented in a listing and summarized.

7.4 Analysis of Efficacy

Efficacy analysis will be conducted on the FAS and, for select endpoints, PPS. The interpretation of results from statistical tests will be primarily based on the FAS. The PPS will be used to assess the robustness of the results from the statistical tests based on the FAS. All randomized subjects will be analyzed according to the treatment to which they are randomized.

7.4.1 Analysis of Primary Endpoint

7.4.1.1 Primary Analysis

The primary endpoint is PFS assessed by the blinded IRC. For each subject, PFS is defined as the time from the date of randomization until the date of radiological disease progression (i.e., PFS) assessed by IRC, or until death due to any cause, whichever is earlier. If a subject has neither progressed nor died, the subject will be censored at the date of last radiological assessment. Subjects who receive new anticancer therapy before radiological progression will be censored at the date of the last radiological assessment before the new anticancer therapy started. If progression or death occurs after missing 2 or more scheduled radiological assessments, the subject will be censored at the date of last radiological assessment or at the date of randomization if no post-baseline radiological assessment is available.

The primary analysis will be performed when approximately 344 PFS events have been observed.

The distribution of PFS will be estimated for each treatment arm using Kaplan-Meier methodology and compared between Arm A and Arm B using log-rank test stratified by:

- Region (Asia vs Non-Asia)
- Number of Metastatic Sites (0 to 2 vs ≥ 3)
- Prior Gastrectomy (Yes or No)

The hypothesis testing on the primary analysis will be performed at an overall 1-sided 0.025 significance level to test the null hypothesis that PFS is not prolonged in Arm A compared to Arm B versus the alternative hypothesis that PFS is prolonged in Arm A compared to Arm B.

In addition, stratified Cox proportional hazard model will be used to estimate the hazard ratio and the corresponding 95% CI.

The primary analysis will be performed using the FAS.

7.4.1.2 Sensitivity Analysis

The analysis described in [Section 7.4.1.1 Primary Analysis] will be conducted in the PPS. Additional sensitivity analyses will be described in the SAP.

7.4.1.3 Subgroup Analysis

The analysis described in [Section 7.4.1.1 Primary Analysis] will be conducted in the subgroups using the FAS. Subgroups defined by baseline factors will be described in the SAP.

Forest plot will be presented to illustrate the strength of treatment effects across subgroups.

7.4.2 Analysis of Secondary Endpoints

7.4.2.1 Overall Survival

A key secondary endpoint OS is defined as the time from the date of randomization until the documented date of death from any cause. All events of death will be included, regardless of whether the event occurred while the subject is still taking study drug or after the subject discontinue study drug. Subjects who are still alive at the time of analysis will be censored at the last day known to be alive.

The distribution of OS will be estimated for each treatment arm using Kaplan-Meier methodology and compared between Arm A and Arm B using the log-rank test stratified by the same stratification factors used for PFS analysis. To maintain the overall Type I error rate at the 0.025 significance level, the hypothesis testing on OS will be performed only if the null hypothesis on the primary analysis is rejected at the overall 1-sided 0.025 significance level. In addition, stratified Cox proportional hazard model will be used to estimate the hazard ratio and the corresponding 95% CI. The sensitivity analysis for the key secondary endpoint will be performed on the PPS.

7.4.2.2 Objective Response Rate

Best overall response (BOR) is determined once all tumor response data for the subject is available. Subjects will be classified by BOR on study as outlined in RECIST 1.1 criteria. For BOR of SD, SD must be documented as present at least once after study entry and at least 8 weeks after first dose.

The ORR is defined as the proportion of subjects with a BOR of CR or PR based on IRC per RECIST V1.1.

The comparison of ORR between Arm A and Arm B will be performed using stratified Cochran-Mantel-Haenszel (CMH) test with the same stratification factor used for the PFS analysis. In addition, ORR for each arm will be estimated and corresponding 95% CI will be constructed.

In addition, percent of subjects with CR will be summarized.

7.4.2.3 Duration of Response

DOR is defined as the time from the date of the first response of CR or PR (whichever is first recorded) as assessed by IRC to the date of radiological progression or death, whichever is earlier. If a subject has not progressed, the subject will be censored at the date of last radiological assessment or at the date of first CR/PR if no post-baseline radiological assessment is available. Other censoring used for the PFS analysis will apply to DOR too.

The distribution of DOR will be estimated for each treatment arm using Kaplan-Meier methodology and compared between Arm A and Arm B using the log-rank test stratified by the same stratification factors used for the PFS analysis. In addition, stratified Cox proportional hazard model will be used to estimate the hazard ratio and the corresponding 95% CI.

7.4.2.4 Health-Related Quality of Life

HRQoL endpoints will be summarized by descriptive statistics with respect to change from baseline for the FAS for each treatment arm. Completion rate for each questionnaire will be summarized by time point. Additional analyses will be described in the SAP.

7.4.3 Analysis of Exploratory Endpoints

7.4.3.1 Time to Progression

TTP is defined as the time from the date of randomization until the date of PD (per RECIST 1.1 by IRC). TTP does not include deaths as event. For deaths before the first documented PD by IRC, subjects will be censored at the time of last radiological assessment. Kaplan-Meier and log-rank methods will be applied to TTP endpoint.

7.4.3.2 Progression Free Survival After Subsequent Therapy (PFS2)

PFS2 is defined as the time from the date of randomization until the date of PD (per investigator) following subsequent anti-cancer therapy, death from any cause or start of any other anti-cancer therapy, whichever is earliest. In cases where PFS2 cannot be reliably determined, discontinuation of subsequent anti-cancer treatment may be used as the event date. Otherwise, subjects will be censored. Subjects who are alive and for whom a PFS2 event date has not been observed should be censored at the last time known to be alive and without second objective disease progression.

The distribution of PFS2 will be estimated for each treatment arm using Kaplan-Meier methodology and compared between Arm A and Arm B using stratified log-rank test with the same stratification factors used for the PFS analysis. In addition, stratified Cox proportional hazard model will be used to estimate the hazard ratio and the corresponding 95% CI.

7.4.3.3 Disease Control Rate

The DCR is defined as the proportion of subjects with a BOR of CR, PR or SD based on RECIST 1.1 by IRC.

The comparison of DCR between Arm A and Arm B will be performed using CMH test with the same stratification factor used for PFS analysis. In addition, DCR for each arm will be estimated and corresponding 95% CI will be constructed.

7.4.3.4 Biomarkers

Biomarkers may be summarized graphically or descriptively, and summary statistics may be tabulated. Associations between biomarkers and clinical (e.g., efficacy, safety or pharmacodynamics, pharmacokinetics) measures may be performed on subjects who have sufficient baseline and on-study measurements to provide interpretable results for specific parameters. Additional post-hoc statistical analyses may be outlined in the SAP.

7.4.3.5 Health Resource Utilization

HRU variables will be summarized for each treatment arm.

7.5 Analysis of Safety

All treated subjects will be analyzed according to the treatment they received.

7.5.1 Adverse Events

AEs will be coded using the Medical Dictionary for Regulatory Activities (MedDRA) and graded using NCI-CTCAE.

Treatment-emergent adverse event (TEAE) is defined as an AE observed after starting administration of the study treatment and within 30 days after the last dose of study treatment.

A study drug-related TEAE is defined as any TEAE with a causal relationship of YES by the investigator.

AEs of special interest described in [Section 5.5.8 Adverse Events of Special Interest] will be summarized.

The number and percentage of AEs, SAEs, AEs leading to interruption/discontinuation, AEs leading to death and AEs related to study drug will be summarized by SOC, preferred term (PT) for SAF. The number and percentage of AEs by toxicity grade will also be summarized. All AEs will be listed.

7.5.2 Laboratory Assessments

For quantitative laboratory measurements descriptive statistics will be used to summarize results and change from baseline for subjects in the SAF by treatment group and time point.

Shifts from baseline to the worst grade based on NCI-CTCAE in laboratory tests will also be tabulated.

7.5.3 Vital Signs

Descriptive statistics will be used to summarize vital sign results and changes from baseline for subjects in the SAF by treatment group and time point.

7.5.4 Routine 12-lead Electrocardiograms

The 12-lead ECG results will be summarized by treatment group and time point.

7.5.5 Eastern Cooperative Oncology Group Performance Status

Summary statistics (number and percent of subjects) for each category of the ECOG performance status at each assessment will be provided. The change from baseline to final visit or early termination will also be summarized. Negative change scores indicate an improvement. Positive scores indicate a decline in performance.

7.6 Analysis of Pharmacokinetics

Descriptive statistics will include the number of subjects (n), mean, standard deviation, minimum, median, maximum, coefficient of variation (CV), geometric mean and geometric CV.

7.6.1 Serum Concentrations

Serum concentrations of IMAB362 will be listed and summarized using descriptive statistics by scheduled time point. Box and whisker plots of trough concentrations against cycle and dosing day will be provided.

7.6.2 Immunogenicity

Immunogenicity of IMAB362 will be summarized using the frequency of ADA positive subjects. The potential relationship between IMAB362 immunogenicity and IMAB362 pharmacokinetics, efficacy, safety profile in subjects will be assessed.

Additional model-based analyses may be performed and reported separately for IMAB362 pharmacokinetics.

7.7 Major Protocol Deviations and Other Analyses

Major protocol deviations as defined in [Section 8.2 Major Protocol Deviations] will be summarized for all randomized subjects by treatment group and total as well as by site. A data listing will be provided by site and subject.

The major protocol deviation criteria will be uniquely identified in the summary table and listing.

7.8 Interim Analysis (and Early Discontinuation of the Clinical Study)

To evaluate whether IMAB362 + CAPOX (Arm A) is beneficial compared to placebo + CAPOX (Arm B) while the study is ongoing, a formal OS interim analysis is planned when the final PFS analysis occurs with the pre-specified number of PFS events. A group sequential design using the O’Brien-Fleming type alpha-spending function [Lan & DeMets, 1983] for efficacy will be utilized to control the overall 1-sided 0.025 significance level (East®) for the OS analyses.

The IDMC may recommend terminating the study for favorable results at the formal efficacy interim analysis using OS. In the case of favorable results, the 1-sided significance level for superiority is 0.0074 for the interim OS analysis and 0.0228 for the final OS analysis. If the 1-sided P value of the interim analysis is less than 0.0074, the IDMC may recommend terminating the study for success. If the study is not stopped after the interim analysis, a final OS analysis will occur after 100% of the planned death events have been observed.

The interim analysis will be conducted by an Independent Data Analysis Center for IDMC. In addition, safety data reviews during the study will be conducted by the IDMC on a periodic basis. For example, the IDMC will have its first safety data review 6 weeks after the 40th subject has been randomized and on study drug for 2 cycles (6 weeks) and meetings will be conducted regularly thereafter, as determined by the IDMC.

The full procedures for IDMC safety review will be described in a separate IDMC Charter.

7.9 Handling of Missing Data, Outliers, Visit Windows, and Other Information

Imputation for missing data, if applicable, will be addressed in the SAP.

8 OPERATIONAL CONSIDERATIONS

8.1 Procedure for Clinical Study Quality Control

8.1.1 Data Collection

The investigator or site designee will enter data collected using an electronic data capture system. In the interest of collecting data in the most efficient manner, the investigator or site designee should record data (including laboratory values, if applicable) in the eCRF within 5 days after the subject visit.

The investigator or site designee is responsible to ensure that all data in the eCRFs and queries are accurate and complete and that all entries are verifiable with source documents. These documents should be appropriately maintained by the site.

The monitor should verify the data in the eCRFs with source documents and confirm that there are no inconsistencies between them.

Laboratory tests are performed at a central laboratory. Central Laboratory data will be transferred electronically to the sponsor or designee at predefined intervals during the study. The central laboratory will provide the sponsor or designee with a complete and clean copy of the data.

Imaging results are read by a central imaging laboratory. Central imaging data will be transferred electronically to the sponsor or designee at predefined intervals during the study. The central imaging laboratory will provide the sponsor or designee with a complete and clean copy of the data.

For Screen failures the demographic data, reason for failing, informed consent, inclusion and exclusion criteria and AEs will be collected in the eCRF.

8.1.1.1 Collection of Data Via Electronic Source

All procedures conducted under the protocol must be documented. For screen failures, the minimum demographic data (sex, birth date, race and informed consent date), outcome of eligibility assessment (inclusion and exclusion criteria), reason for screen failure and AEs details must be documented.

The investigator or designee will be responsible for eCRF completion and that all data and queries are accurate, complete and are verifiable with the source. The source should be appropriately maintained by the clinical unit.

Electronic data sources and any supporting documents should be available for review/retrieval by the sponsor/designee at any given time.

8.1.1.2 Electronic Clinical Outcomes Assessment

eCOA assessments will be performed according to the Schedule of Assessments [Table 1]. Subject HRQoL and HRU questionnaires will be completed by the subject on an electronic tablet device during site visits. The subject questionnaire responses captured on the electronic device will be transferred to the eCOA vendor's central portal (web portal). The investigator or site designee should review the questionnaire data on the web portal for correct completion while the subject is at the site. The questionnaire data will be transferred electronically to sponsor or designee at predefined intervals during the study. Sponsor/CRO staff also have access to the vendor web portal for continuous review of the data and access to reports. The vendor will provide sponsor or designee with a complete and clean copy of the data.

For further details please refer to the eCOA guidelines/manual (e.g., ERT Site Guide to eCOA Product).

8.2 Major Protocol Deviations

A major protocol deviation is generally an unplanned excursion from the protocol that is not implemented or intended as a systematic change. The investigator is responsible for ensuring the study is conducted in accordance with the procedures and evaluations described in this protocol and must protect the rights, safety, and welfare of subjects. The investigator should not implement any deviation from, or changes of, the protocol, unless it is necessary to eliminate an immediate hazard to study subjects.

A protocol waiver is a documented prospective approval of a request from an investigator to deviate from the protocol. Protocol waivers are strictly prohibited.

The major protocol deviation criteria are as follows:

PD1 - Entered into the study even though they did not satisfy entry criteria,

PD2 - Developed withdrawal criteria during the study and was not withdrawn,

PD3 - Received wrong treatment or incorrect dose,

PD4 - Received excluded concomitant treatment.

When a major deviation from the protocol is identified for an individual subject, the investigator or designee must ensure the sponsor is notified. The sponsor will follow up with the investigator, as applicable, to assess the deviation and the possible impact to the safety of the subject to determine subject continuation in the study.

If a major deviation impacts the safety of a subject, the investigator must contact the sponsor immediately.

The investigator will also assure that deviations meeting IRB/IEC and applicable regulatory authorities' criteria are documented and communicated appropriately. All documentation and communications to the IRB/IEC and applicable regulatory authorities will be provided to the sponsor and maintained within the trial master file.

9 END OF TRIAL

The end of the study is defined as the last visit or scheduled procedure shown in the Schedule of Assessments [Table 1] for the last study participant in the study.

Study completion is defined as the conclusion of data collection for the defined study endpoints. The study may be closed within a participating country per local regulations once the study has completed and if all subjects enrolled in the country are no longer receiving study treatment. In addition, the sponsor may prematurely terminate the study for reasonable cause at any time.

10 STUDY ORGANIZATION

10.1 Independent Data Monitoring Committee

An IDMC will evaluate the unblinded safety data of subjects enrolled on a periodic basis during this study. IDMC members will be clinicians with expertise in gastric cancer studies and are not investigators participating in this study or Astellas employees. A separate charter will outline the activities of this committee.

10.2 Other Study Organization

SPECIFIC TO SITES IN JAPAN: The Japan site contact list is kept as a separate attachment to the protocol.

11 REFERENCES

Ajani JA, Rodriguez W, Bodoky G, Moiseyenko V, Lichinitser M, Gorbunova V, et al. Multicenter phase III comparison of cisplatin/S-1 with cisplatin/infusional fluorouracil in advanced gastric or gastroesophageal adenocarcinoma study: the FLAGS trial. J Clin Oncol. 2010 Mar;28(9):1547-53.

Amiri M, Janssen F, Kunst AE. The decline in stomach cancer mortality: exploration of future trends in seven European countries. Eur J Epidermiol. 2011;26:23-8.

Bang Y, Chung H, Xu J, Lordick F, Sawaki A, Al-Sakaff N, et al. Pathological features of advanced gastric cancer (GC): Relationship to human epidermal growth factor receptor 2 (HER2) positivity in the global screening programme of the ToGA trial. J Clin Oncol. 2009;27(155):4556.

Bang YJ, Van Cutsem E, Feyereislova A, Chung HC, Shen L, Sawaki A. Trastuzumab in combination with chemotherapy versus chemotherapy alone for treatment of HER2-positive advanced gastric or gastro-oesophageal junction cancer (ToGA): a phase 3, open-label, randomised controlled trial. Lancet 2010;376:687-97.

Cancer Fact Sheet. World Health Organization; February 2018.

CYRAMZA Prescribing Information. Indianapolis, IN: Eli Lilly and Company; 2017.

Eisenhauer EA, Therasse P, Bogaerts J, et al. New response evaluation criteria in solid tumours: revised RECIST guideline (version 1.1). Eur J Cancer 2009;45:228-247.

Fuchs CS, Tomasek J, Yong CJ, Dumitru F, Passalacqua R, Goswami C, et al. Ramucirumab monotherapy for previously treated advanced gastric or gastro-oesophageal junction adenocarcinoma (REGARD): an international, randomised, multicentre, placebo-controlled, phase 3 trial. Lancet. 2014;383:31-39.

Gunzel D, Yu SL. Claudins of the modulation of tight junction permeability. Physiol. Rev. 2013;93(2): 525-69.

HERCEPTIN (trastuzumab) [package insert]. South San Francisco, CA: Genentech, Inc.; 2016.

Karanjawala ZE, Illei PB, Ashfaq R, Infante JR, Murphy K, Pandey A, et al. New markers of pancreatic cancer identified through differential gene expression analyses: claudin 18 and annexin A8. Am J Surg Pathol. 2008;32:188-196.

Kim GM, Jeung HC, Rha SY, Kim HS, Jung I, Nam BH, et al. A randomized phase II trial of S-1-oxaliplatin versus capecitabine-oxaliplatin in advanced gastric cancer. Eur J Cancer. 2012 Mar;48(4):518-26.

Kim NK, Park YS, Heo DS, Suh C, Kim SY, Park KC, et al. A phase III randomized study of 5-fluorouracil and cisplatin versus 5-fluorouracil, doxorubicin and mitomycin C versus 5-fluorouracil alone in the treatment of advanced gastric cancer. Cancer. 1993 Jun;71(12):3813–18.

Lan KKG and De Mets DL. Discrete sequential boundaries for clinical trials. Biometrika. 1983;70:659-663.

Lederman L. Gastric Cancer: local and global burden. Supplement: the current and future management of gastric cancer. Am J Manag Care. 2017:2-5.

Lee JH, Kim KS, Kim TJ, Hong SP, Song SY, Chung JB, et al. Immunohistochemical analysis of claudin expression in pancreatic cystic tumors. Oncol Rep. 2011;25:971-978.

Mori M, Sawada N, Kokai Y, Satoh M. Role of tight junctions in the occurrence of cancer invasion and metastasis. Med Electron Microsc. 1999;32:193-198.

National Comprehensive Cancer Network Clinical Practice Guidelines in Oncology (NCCN Guidelines): Gastric cancer. National Comprehensive Cancer Network (NCCN). 2017; version 1.2017.

Niimi T, Nagashima K, Ward JM, Minoo P, Zimonjic DB, Popescu NC, et al. claudin-18, a novel downstream target gene for the T/EBP/NKX2.1 homeodomain transcription factor, encodes lung- and stomach-specific isoforms through alternative splicing. Mol Cell Biol. 2001;21(21):7380-7390.

Ohtsu A, Shah MA, Van Cutsem E, Rha SY, Sawaki A, Park SR, et al. Bevacizumab in combination with chemotherapy as first-line therapy in advanced gastric cancer: a randomized, double-blind, placebo-controlled phase III study. J Clin Oncol. 2011;29:3968-3976.

Pasini F, Fraccon AP, De Manzoni G. The role of chemotherapy in metastatic gastric cancer. Cancer Res. 2011;31:3543-3554.

Pennathur A, Gibson MK, Jobe BA, Luketich JD. Oesophageal carcinoma. Lancet. 2013;381:400-12.

Sahin U, Koslowski M, Dhaene K, Usener D, Brandenburg G, Seitz G, et al. Claudin-18 splice variant 2 is a pan-cancer target suitable for therapeutic antibody development. Clin Cancer Res. 2008;14(23):7624-34.

Sanada Y, Hirose Y, Osada S, Tanaka Y, Takahashi T, Yamaguchi K, et al. Immunohistochemical study of claudin 18 involvement in intestinal differentiation during the progression of intraductal papillary mucinous neoplasm. Anticancer Res. 2010;30:2995-3003.

Shah MA, Bang YJ, Lordick F, Alsina M, Chen M, Hack SP, et al. Effect of Fluorouracil, Leucovorin, and Oxaliplatin With or Without Onartuzumab in HER2-Negative, MET-Positive Gastroesophageal Adenocarcinoma - The METGastric Randomized Clinical Trial. JAMA Oncol. 2017;3(5):620-7.

Vita FDe, Orditura M, Matano E, Bianco R, Carlomagno C, Infusino S, et al. A phase II study of biweekly oxaliplatin plus infusional 5-fluorouracil and folinic acid (FOLFOX-4) as first-line treatment of advanced gastric cancer patients. Br J Cancer. 2005;92(9):1644-49.

Waddell T, Chau I, Cunningham D, Gonzalez D, Okines AF, Wotherspoon A, et al. Epirubicin, oxaliplatin, and capecitabine with or without panitumumab for patients with previously untreated advanced oesophagogastric cancer (REAL3): a randomized, open-label phase 3 trial. Lancet Oncol. 2013;14(6):481-9.

Wilke H, Muro K, Van Cutsem E, Oh SC, Bodoky G, Shimada Y, et al. Ramucirumab plus paclitaxel versus placebo plus paclitaxel in patients with previously treated advanced gastric or gastro-oesophageal junction adenocarcinoma (RAINBOW): a double-blind, randomised phase 3 trial. Lancet Oncol. 2014;15:1224-35.

Woll S, Schlitter AM, Dhaene K, Roller M, Esposito I, Sahin U, et al. Claudin 18.2 is a target for IMAB362 antibody in pancreatic neoplasms. Int. J. Cancer. 2014;134:731-39.

XELOX CCO Formulary, August 2017.

12 APPENDICES

12.1 Ethical, Regulatory, and Study Oversight Considerations

12.1.1 Ethical Conduct of the Study

The study will be conducted in accordance with the protocol, ICH guidelines, applicable regulations and guidelines governing clinical study conduct and the ethical principles that have their origin in the Declaration of Helsinki.

12.1.2 Institutional Review Board (IRB)/Independent Ethics Committee (IEC)/Competent Authorities (CA)

GCP requires that the clinical protocol, any protocol amendments, the IB, the informed consent and all other forms of subject information related to the study (e.g., advertisements used to recruit subjects) and any other necessary documents be reviewed by an IEC/IRB. The IEC/IRB will review the ethical, scientific and medical appropriateness of the study before it is conducted. IEC/IRB approval of the protocol, informed consent and subject information and/or advertising, as relevant, will be obtained prior to the authorization of drug shipment to a study site.

Any substantial amendments to the protocol will require IRB/IEC approval before to implementation , except for changes necessary to eliminate an immediate hazard to subjects.

The investigator will be responsible for the following:

- Providing written summaries of the status of the study to the IRB/IEC annually or more frequently in accordance with the requirements, policies, and procedures established by the IRB/IEC
- Notifying the IRB/IEC of SAEs or other significant safety findings as required by IRB/IEC procedures
- Providing oversight of the conduct of the study at the site and adherence to requirements of 21 CFR, ICH guidelines, the IRB/IEC, European regulation 536/2014 for clinical studies (if applicable), and all other applicable local regulations

12.1.3 Protocol Amendment and/or Revision

Any changes to the study that arise after approval of the protocol must be documented as protocol amendments: substantial amendments and/or non-substantial amendments. Depending on the nature of the amendment, either IRB/IEC, Competent Authority approval or notification may be required. The changes will become effective only after the approval of the sponsor, the investigator, the regulatory authority, and the IRB/IEC (if applicable).

Amendments to this protocol must be signed by the sponsor and the investigator. Written verification of IRB/IEC approval will be obtained before any amendment is implemented. Modifications to the protocol that are administrative in nature do not require IRB/IEC approval, but will be submitted to the IRB/IEC for their information, if required by local regulations.

If there are changes to the informed consent, written verification of IRB/IEC approval must be forwarded to the sponsor. An approved copy of the new informed consent must also be forwarded to the sponsor.

12.1.4 Informed Consent of Subjects

12.1.4.1 Subject Information and Consent

The investigator or his/her representative will explain the nature of the study to the subject or his/her guardian or legal representative (if applicable), and answer all questions regarding this study. Prior to any study-related screening procedures being performed on the subject, the informed consent statement will be reviewed and signed (***UNIQUE TO JAPAN REGION****:* place a personal seal) and dated by the subject or his/her guardian or legal representative, the person who administered the informed consent and any other signatories according to local requirements. A copy of the signed (***UNIQUE TO JAPAN REGION:*** or sealed) ICF will be given to the subject and the original will be placed in the subject's medical record. An entry must also be made in the subject's dated source documents to confirm that informed consent was obtained prior to any study-related procedures and that the subject received a signed copy.

The signed consent forms will be retained by the investigator and made available (for review only) to the study monitor and auditor regulatory authorities and other applicable individuals upon request.

12.1.4.2 Supply of New and Important Information Influencing the Subject's Consent and Revision of the Written Information

1. The investigator or his/her representative will immediately inform the subject orally whenever new information becomes available that may be relevant to the subject's consent or may influence the subject's willingness to continue to participate in the study (e.g., report of serious drug adverse drug reaction). The communication must be documented in the subject's medical records and whether the subject is willing to remain in the study or not must be confirmed and documented.
2. The investigator must update their ICF and submit it for approval to the IRB/IEC. The investigator or his/her representative must obtain written informed consent from the subject on all updated ICFs throughout their participation in the study. The investigator or his/her designee must re-consent subjects with the updated ICF even if relevant information was provided orally. The investigator or his/her representative who obtained the written informed consent and the subject should sign and date the ICF (***UNIQUE TO JAPAN REGION:*** place a personal seal). A copy of the signed (***UNIQUE TO JAPAN REGION:*** or sealed) ICF will be given to the subject and the original will be placed in the subject's medical record. An entry must be made in the subject's records documenting the re-consent process.

12.1.5 Source Documents

Source data must be available at the site to document the existence of the study subjects and to substantiate the integrity of study data collected. Source data must include the original documents relating to the study, as well as the medical treatment and medical history of the subject.

The investigator is responsible for ensuring the source data are attributable, legible, contemporaneous, original, accurate and complete whether the data are hand-written on paper or entered electronically. If source data are created (first entered), modified, maintained, achieved, retrieved or transmitted electronically via computerized systems (and/or other kind of electric devices) as part of regulated clinical study activities, such systems must be compliant with all applicable laws and regulations governing use of electronic records and/or electronic signatures. Such systems may include, but are not limited to, electronic medical/health records, protocol related assessments, AE tracking, and/or drug accountability.

Paper records from electronic systems used in place of electronic format must be certified copies. A certified copy must be an exact copy and must have all the same attributes and information as the original. Certified copies must include signature and date of the individual completing the certification. Certified copies must be a complete and chronological set of study records (including notes, attachments, and audit trail information (if applicable). All printed records must be kept in the subject file and available for archive.

12.1.6 Record Retention

The investigator will archive all study data (e.g., subject identification code list, source data, eCRFs and investigator's file) and relevant correspondence. These documents are to be kept on file for the appropriate term determined by local regulation (for US sites, two years after approval of the NDA or discontinuation of the IND). The sponsor will notify the site/investigator if the NDA/MAA/J-NDA is approved or if the IND/IMPD/CHIKEN TODOKE is discontinued. The investigator agrees to obtain the sponsor's agreement prior to disposal, moving, or transferring of any study-related records. The sponsor will archive and retain all documents pertaining to the study according to local regulations.

Data generated by the methods described in the protocol will be recorded in the subjects' medical records and/or study progress notes.

12.1.7 Subject Confidentiality and Privacy

Individual subject medical information obtained as a result of this study is considered confidential and disclosure to third parties is prohibited unless otherwise the subject provides written consent or approval. Additional medical information may be given only after approval of the subject to the investigator or to other appropriate medical personnel responsible for the subject's well-being.

The sponsor shall not disclose any confidential information on subjects obtained during the performance of their duties in the clinical study without justifiable reasons.

Even though any individuals involved in the study, including the study monitors and auditors, may get to know matters related to subject's privacy due to direct access to source documents, or from other sources, they may not leak the content to third parties.

The sponsor affirms the subject's right to protection against invasion of privacy. Only a subject identification number will identify subject data retrieved by the sponsor. However, the sponsor requires the investigator to permit the sponsor, sponsor's representative(s), the IRB/IEC and when necessary, representatives of the regulatory health authorities to review and/or to copy any medical records relevant to the study.

The sponsor agrees to comply and process personal data in accordance with all applicable privacy laws and regulations, including, without limitation, the Personal Information Protection Law in Japan and Privacy laws in the US. If the services will involve the collection or processing of personal data (as defined by applicable data protection legislation) within the European Economic Area (EEA), then sponsor shall serve as the controller of such data, as defined by the European Union (EU) Data Protection Directive, and Investigator and/or third party shall act only under the instructions of the sponsor in regard to personal data If sponsor is not based in the EEA, sponsor must appoint a third party to act as its local data protection representative or arrange for a co-controller established in the EU for data protection purposes in order to comply with the Directive.

12.1.8 Arrangement for Use of Information and Publication of the Clinical Study

Information concerning the study drug, patent applications, processes, unpublished scientific data, the IB and other pertinent information is confidential and remains the property of the sponsor. Details should be disclosed only to the persons involved in the approval or conduct of the study. The investigator may use this information for the purpose of the study only. It is understood by the investigator that the sponsor will use the information obtained during the clinical study in connection with the development of the drug and therefore may disclose it as required to other clinical investigators or to regulatory agencies. In order to allow for the use of the information derived from this clinical study, the investigator understands that he/she has an obligation to provide the sponsor with all data obtained during the study.

Publication of the study results is discussed in the clinical study agreement.

12.1.9 Insurance of Subjects and Others (UNIQUE TO JAPAN REGION/STUDIES ENROLLING SUBJECTS IN EU)

If a subject suffers any study-related injury, the sponsor will compensate appropriately according to the severity and duration of the damage. However, if it was caused intentionally or was due to gross negligence by the study site, the sponsor will consult with the study site about handling the injury, based on the agreed study contract. Compensation for the study-related injury is provided by the following procedures:

1. If a subject incurs an injury as a result of participation in the clinical study, the study site should provide medical treatment and other necessary measures. The sponsor should be notified of the injury.

2. When the subject claims compensation from the study site for the above study-related injury, or such compensation may be claimed, the study site should immediately communicate the fact to the sponsor. Both parties should work together towards compensation settlement.
3. The sponsor shall pay compensation or indemnification and bear expenses necessary for the settlement as provided in the clinical contract.
4. The sponsor shall make an arranging for insurance and take measures necessary to ensure the compensation or indemnification mentioned above.

The sponsor has covered this study by means of an insurance of the study according to national requirements. The name and address of the relevant insurance company, the certificate of insurance, the policy number and the sum insured are provided in the investigator's file.

12.1.10 Signatory Investigator for Clinical Study Report

ICH E3 guidelines recommend and EU Directive 2001/83/EC requires that a final study report, which forms part of a marketing authorization application, be signed by the representative for the coordinating investigator(s) or the principal investigator(s). The representative for the coordinating investigator (s) or the principal investigator(s) will have the responsibility to review the final study results to confirm to the best of his/her knowledge it accurately describes the conduct and results of the study. The representative for coordinating investigator(s) or the principal investigator(s) will be selected from the participating investigators by the sponsor prior to database lock.

12.2 Procedure for Clinical Study Quality Control

12.2.1 Clinical Study Monitoring

The sponsor or delegated CRO is responsible for monitoring the clinical study to ensure that subject's human rights, safety, and well-being are protected, that the study is properly conducted in adherence to the current protocol and GCP, and study data reported by the investigator/sub-investigator are accurate and complete and that they are verifiable with study-related records such as source documents. The sponsor is responsible for assigning study monitor(s) to this study for proper monitoring. They will monitor the study in accordance with planned monitoring procedures.

12.2.2 Direct Access to Source Data/Documents

The investigator and the study site must accept monitoring and auditing by the sponsor or delegated CRO as well as inspections from the IRB/IEC and relevant regulatory authorities. In these instances, they must provide all study-related records, including source documents when they are requested by the sponsor monitors and auditors, the IRB/IEC or regulatory authorities. The confidentiality of the subject's identities shall be well protected consistent with local and national regulations when the source documents are subject to direct access.

12.2.3 Data Management

Data Management will be coordinated by the Data Science of the sponsor in accordance with the SOPs for data management. All study-specific processes and definitions will be documented by Data Management. eCRF completion will be described in the eCRF instructions. Coding of medical terms and medications will be performed using MedDRA and World Health Organization (WHO) Drug Dictionary, respectively.

12.2.4 QUALITY ASSURANCE

The sponsor is implementing and maintaining quality assurance and quality control systems with written SOPs to ensure that studies are conducted and data are generated, documented, recorded, and reported in compliance with the protocol, GCP, and applicable regulatory requirement(s). Where applicable, the quality assurance and quality control systems and written SOPs of the CRO will be applied.

The sponsor or sponsor's designee may arrange to audit the clinical study at any or all investigational sites and facilities. The audit may include on-site review of regulatory documents, case report forms, and source documents. Direct access to these documents will be required by the auditors.

12.3 Contraception Requirements

WOCBP participants who choose complete abstinence must continue to have pregnancy tests, as specified in Schedule of Assessments.

WOMEN OF CHILDBEARING POTENTIAL DEFINITIONS AND METHODS OF CONTRACEPTION DEFINITIONS (WOCBP)

A woman is considered fertile following menarche and until becoming postmenopausal unless permanently sterile.

Women in the following categories are not considered WOCBP

- Premenarchal
- Premenopausal female with 1 of the following:
  - Documented hysterectomy
  - Documented bilateral salpingectomy
  - Documented bilateral oophorectomy
- Postmenopausal

Documentation of any of these categories can come from the site personnel's review of the female subject's medical records, medical examination, or medical history interview.

A postmenopausal state is defined as at least 12 months after last regular menstrual bleeding without an alternative medical cause.

- In case the last regular menstrual bleeding cannot be clearly determined, confirmation with repeated FSH measurements of at least > 40 IU/L (or higher per local institutional guidelines), is required.

Females on HRT and whose menopausal status is in doubt will be required to use one of the non-estrogen hormonal highly effective contraception methods if they wish to continue their HRT during the study. Otherwise, they must discontinue HRT to allow confirmation of postmenopausal status by repeated FSH measurements before study enrollment.

CONTRACEPTION GUIDANCE FOR FEMALE PARTICIPANTS OF CHILD BEARING POTENTIAL

One of the highly effective methods of contraception listed below is required at the time of informed consent and until the end of relevant systemic exposure, defined as 6 months after the final study drug administration[a].

Highly Effective Contraceptive Methods (Failure rate of <1% per year when used consistently and correctly)[b]

Combined (estrogen- and progestogen-containing) hormonal contraception associated with inhibition of ovulation

- oral
- intravaginal
- transdermal

Progestogen-only hormonal contraception associated with inhibition of ovulation

- oral
- injectable
- implantable

Hormonal methods of contraception containing a combination of estrogen and progesterone, vaginal ring, injectables, implants and intrauterine hormone-releasing system (IUS)

- intrauterine device (IUD)
- bilateral tubal occlusion

Vasectomized partner *(A vasectomized partner is a highly effective contraception method provided that the partner is the sole male sexual partner of the WOCBP and the absence of sperm has been confirmed. If not, an additional highly effective method of contraception should be used.)*

Sexual abstinence (*Sexual abstinence is considered a highly effective method only if defined as refraining from heterosexual intercourse during the entire period of risk associated with the study drug. The reliability of sexual abstinence needs to be evaluated in relation to the duration of the study and the preferred and usual lifestyle of the participant. It is not necessary to use any other method of contraception when complete abstinence is elected.)*

[a] Local laws and regulations may require use of alternative and/or additional contraception methods.

[b] Typical use failure rates may differ from those when used consistently and correctly. Use should be consistent with local regulations regarding the use of contraceptive methods for participants participating in clinical studies.

CONTRACEPTION GUIDANCE FOR MALE PARTICIPANTS WITH PARTNER(S) OF CHILD BEARING POTENTIAL.

Male participants with female partners of childbearing potential are eligible to participate if they agree to the following during treatment and until the end of relevant systemic exposure defined as 6 months after final drug administration.

- Inform any and all partner(s) of their participation in a clinical drug study and the need to comply with contraception instructions as directed by the investigator.
- Male participants are required to use a condom during treatment and until end of relevant systemic exposure defined as 6 months after final drug administration.
- Female partners of male participants who have not undergone a vasectomy with the absence of sperm confirmed or a bilateral orchiectomy should consider use of effective methods of contraception until the end of relevant systemic exposure, defined as 6 months after final drug administration.

12.4 Concomitant Medication Restrictions or Requirements

Prohibited Concomitant Treatment

The following are strictly prohibited:

- Sorivudine or analogs (during capecitabine treatment)
- Systemic immunosuppressive agents:
  - Concurrent systemic immunosuppressive therapy, in particular systemic corticosteroids, should be stopped 14 days prior to first dose of study treatment.
  - Subjects are allowed to use a physiologic replacement dose of hydrocortisone or its equivalent (defined as up to 30 mg per day of hydrocortisone or up to 10 mg per day of prednisone).
- Live vaccines should be avoided during the treatment period in which subject is receiving capecitabine and up to 6 months after final capecitabine dose.
- Other systemic chemotherapy, immunotherapy, radiotherapy, herbal medications or other treatments intended for antitumor activity. Palliative radiotherapy for peripheral bone metastases is allowed.
- Investigational products or therapy other than IMAB362.

Cautionary Concomitant Treatment

Considerations should be given to avoid or minimize the use of the following concomitant medications, if possible, during IMAB362/placebo treatment:

- Systemic corticosteroids, because their impact on the potential efficacy of IMAB362 is not known.
  - Systemic corticosteroids should be avoided or minimized while subject is on study treatment unless required for management of an emergent medical condition (e.g., severe nausea/vomiting or hypersensitivity reaction).
  - For a subject's first dose of IMAB362/placebo, it is recommended that the prophylactic use of corticosteroids be avoided.
  - Inhaled, intranasal and topically applied steroids are allowed.
- Avoid the class of 5-HT3 blockers in subjects with congenital long QT syndrome. Administer these drugs with caution in subjects who have or may develop QTc prolongation.
- Nonsteroidal anti-inflammatory drugs (NSAIDs) because of the potential to cause gastric ulcers and covert bleeding.
  - In such cases where NSAID use is necessary, the use of NSAIDs with lower gastric ulcerogenic potential is preferred and concomitant gastric protection with proton pump inhibitors is recommended.

The following should be avoided or used with caution and closely monitored during CAPOX administration:

- Cytochrome P450 (CYP) 2C9 substrates (Subjects taking coumarin-derivative anticoagulants concomitantly with capecitabine should have PT/INR monitored regularly and anticoagulant dose adjusted accordingly).
- Anti-epileptic medications (e.g. phenobarbital, phenytoin and primidone)

12.5 Liver Safety Monitoring and Assessment

Any subject enrolled in a clinical study with active drug therapy and reveals an increase of serum aminotransferases (AT) to > 3 x ULN (to > 5 x ULN in subjects with liver metastases) or bilirubin > 2 x ULN should undergo detailed testing for liver enzymes (including at least ALT, AST, alkaline phosphatase [ALP] and total bilirubin). Testing should be repeated within 72 hours of notification of the test results. For studies for which a central laboratory is used, alerts will be generated by the central laboratory regarding moderate and severe liver abnormality to inform the investigator, study monitor and study team. Subjects should be asked if they have any symptoms suggestive of hepatobiliary dysfunction.

Definition of Liver Abnormalities

Confirmed abnormalities will be characterized as moderate and severe where ULN:

	**ALT or AST**		**Total Bilirubin**
**Moderate**	> 3 x ULN (in subjects without liver metastases), > 5 x ULN (in subjects with liver metastases)	or	> 2 x ULN
**Severe***	> 3 x ULN	and	> 2 x ULN

In addition, the subject should be considered to have severe hepatic abnormalities for any of the following:

- ALT or AST > 8 x ULN.
- ALT or AST > 5 x ULN for more than 2 weeks (in the absence of liver metastases).
- ALT or AST > 3 x ULN and International Normalized Ratio (INR) > 1.5 (If INR testing is applicable/evaluated).
- ALT or AST > 3 x ULN with the appearance of fatigue, nausea, vomiting, right upper quadrant pain or tenderness, fever, rash and/or eosinophilia (> 5%).

The investigator may determine that abnormal liver function results, other than as described above, may qualify as moderate or severe abnormalities and require additional monitoring and follow-up.

Follow-up Procedures

Confirmed moderate and severe abnormalities in hepatic functions should be thoroughly characterized by obtaining appropriate expert consultations, detailed pertinent history, physical examination and laboratory tests. The site staff is to complete the liver abnormality case report form (LA-CRF). Subjects with confirmed abnormal liver function testing should be followed as described below.

Confirmed moderately abnormal LFTs should be repeated 2 to 3 times weekly then weekly or less if abnormalities stabilize or the study treatment has been discontinued and the subject is asymptomatic.

Severe hepatic liver function abnormalities as defined above, in the absence of another etiology may be considered an important medical event and may be reported as a SAE. The sponsor

should be contacted and informed of all subjects for whom severe hepatic liver function abnormalities possibly attributable to study treatment are observed.

To further assess abnormal hepatic laboratory findings, the investigator is expected to:

- Obtain a more detailed history of symptoms and prior or concurrent diseases. Symptoms and new-onset diseases is to be recorded as “AEs” in the (e)CRF. Illnesses and conditions such as hypotensive events, and decompensated cardiac disease that may lead to secondary liver abnormalities should be noted. Nonalcoholic steatohepatitis is seen in obese hyperlipoproteinemic, and/or diabetic subjects and may be associated with fluctuating AT levels. The investigator should ensure that the medical history form captures any illness that predates study enrollment that may be relevant in assessing hepatic function.
- Obtain a history of concomitant drug use (including nonprescription medication, complementary and alternative medications), alcohol use, recreational drug use and special diets. Medications, including dose, is to be entered in the (e)CRF. Information on alcohol, other substance use and diet should be entered on the LA-CRF or an appropriate document.
- Obtain a history of exposure to environmental chemical agents.
- Based on the subject’s history, other testing may be appropriate including:
    - Acute viral hepatitis (A, B, C, D, E or other infectious agents),
    - Ultrasound or other imaging to assess biliary tract disease,
    - Other laboratory tests including INR, direct bilirubin.
- Consider gastroenterology or hepatology consultations.
- Submit results for any additional testing and possible etiology on the LA-CRF or an appropriate document.

Study Treatment Discontinuation

In the absence of an explanation for increased LFT’s, such as viral hepatitis, preexisting or acute liver disease, presence of liver metastases or exposure to other agents associated with liver injury, the subject may be discontinued from study treatment. The investigator may determine that it is not in the subject’s best interest to continue study treatment. Discontinuation of study treatment should be considered if:

- ALT or AST > 8 x ULN.
- ALT or AST > 5 x ULN for more than 2 weeks (in subjects without liver metasteses).
- ALT or AST > 3 x ULN and total bilirubin > 2 x ULN or INR > 1.5 (If INR testing is applicable/evaluated).
- ALT or AST > 5 x ULN and (total bilirubin > 2 x ULN in subjects with liver metasteses).
- ALT or AST > 3 x ULN with the appearance of fatigue, nausea, vomiting, right upper quadrant pain or tenderness, fever, rash and/or eosinophilia (> 5%).

In addition, if close monitoring for a subject with moderate or severe hepatic laboratory tests is not possible, study treatment should be discontinued.

*Hy's Law Definition—The 2 "requirements" for Hy's Law are:

1. Evidence that a drug can cause hepatocellular-type injury, generally shown by a higher rate than control of people with 3 x and greater transaminase elevations over ULN (2 x elevations are too common in treated and untreated subjects to be discriminating).
2. Cases of increased bilirubin (to at least 2 x ULN) in people with concurrent transaminase elevation to at least 3 x ULN (but it is almost invariably higher) and no evidence of intra-or extra-hepatic bilirubin obstruction (elevated ALP) or Gilbert's syndrome. [Temple, 2006]

<u>FDA Guidance for Industry, "Drug-Induced Liver Injury: Premarketing Clinical Evaluation" 2009:</u>

1. The drug causes hepatocellular injury, generally shown by a higher incidence of 3-fold or greater elevations above the ULN of ALT or AST than the (non-hepatotoxic) control drug or placebo.
2. Among study subjects showing such AT elevations, often with ATs much greater than 3 x ULN, 1 or more also show elevation of serum total bilirubin to > 2 x ULN, without initial findings of cholestasis (elevated serum ALP).
3. No other reason can be found to explain the combination of increased AT and total bilirubin, such as viral hepatitis A, B or C; preexisting or acute liver disease; or another drug capable of causing the observed injury.

<u>References</u>

Temple R. Hy's law: Predicting Serious Hepatotoxicity. Pharmacoepidemiol Drug Saf. 2006 April;15(Suppl 4):241-3.

Guidance for Industry titled "Drug-Induced Liver Injury: Premarketing Clinical Evaluation" issued by FDA on July 2009.

12.6 Common Serious Adverse Events

The following is a list of SAEs that the sponsor considers to be associated with the disease state being studied. The list does NOT change your reporting obligations or prevent the need to report an AE meeting the definition of an SAE as detailed in [Section 5.5.2 Definition of Serious Adverse Events]. The purpose of this list is to alert the investigator that some events reported as SAEs may not require expedited reporting to the regulatory authorities based on the classification of "common SAEs". The investigator is required to follow the requirements detailed in [Section 5.5.5 Reporting of Serious Adverse Events].

For IND safety reporting, single occurrences of the following events may be excluded from expedited reporting to the FDA. If aggregate analysis of these events indicates they occur more frequently with study treatment, an expedited IND safety report may be submitted to the FDA.

AEs most likely related to Gastric or GEJ adenocarcinoma:

- Gastric reflux
- Abdominal pain
- Abdominal distention
- Dysphagia
- Loss of appetite

12.7 Pharmacogenomic (PGx) Analysis With Banked Sample

INTRODUCTION

The PGx research aims to provide information regarding how naturally occurring differences in a subject's gene and/or expression of genes based on genetic variation may impact what treatment options are best suited for the subject. Through investigation of PGx by technologies such as genotyping, gene sequencing, statistical genetics and Genome-Wide Association Studies, the relationship between gene profiles and a drug's kinetics, efficacy or toxicity may be better understood. As many diseases may be influenced by 1 or more genetic variations, PGx research may identify which genes are involved in determining the way a subject may or may not respond to a drug.

OBJECTIVES

The PGx research that may be conducted in the future with acquired blood samples is exploratory. The objective of this research will be to analyze or determine genes of relevance to clinical response, pharmacokinetics and toxicity/safety issues.

By analyzing genetic variations, it may be possible to predict an individual subject's response to treatment in terms of efficacy and/or toxicity.

SUBJECT PARTICIPATION

Subjects who have consented to participate in this study may participate in this PGx sub-study. Subjects must provide written consent prior to providing any blood samples that may be used at a later time for PGx analysis.

SAMPLE COLLECTION AND STORAGE

Subjects who consent to participate in this sub-study will provide one tube of whole blood of approximately 4–6 mL per Astellas' instructions. Each sample will be identified by the unique subject number. Samples will be shipped to a designated banking CRO as directed by Astellas.

PGx ANALYSIS

Details on the potential PGx analysis cannot be established yet. Astellas may initiate the PGx analysis if evidence suggests that genetic variants may be influencing the drug's kinetics, efficacy and/or safety.

DISPOSAL OF PGx SAMPLES/DATA

All PGx samples collected will be stored for a period of up to 15 years following study database hard-lock. If there is no requirement for analysis, the whole blood sample will be destroyed after the planned storage period. The subject has the right to withdraw consent at any time. When a subject's withdraw notification is received, the PGx sample will be destroyed. The results of any PGx analysis conducted on a sample prior to its withdrawal will be retained at Astellas indefinitely unless otherwise specified by local regulation.

INFORMATION DISCLOSURE TO THE SUBJECTS

Exploratory PGx analysis may be conducted following the conclusion of the clinical study, if applicable. The results of the PGx analysis will not be provided to any investigators or subjects, nor can the results be requested at a later date. Any information that is obtained from the PGx analysis will be the property of Astellas.

12.8 Eastern Cooperative Oncology Group Performance Status Scale

GRADE	ECOG PERFORMANCE STATUS
0	Fully active, able to carry on all pre-disease performance without restriction
1	Restricted in physically strenuous activity but ambulatory and able to carry out work of a light or sedentary nature (e.g., light house work, office work)
2	Ambulatory and capable of all self-care but unable to carry out any work activities; up and about more than 50% of waking hours
3	Capable of only limited self-care; confined to bed or chair more than 50% of waking hours
4	Completely disabled; cannot carry on any self-care; totally confined to bed or chair
5	Dead

Reference: Oken MM, Creech RH, Tormey DC, Horton J, Davis TE, McFadden ET, et al. Toxicity and response criteria of the Eastern Cooperative Oncology Group. Am J Clin Oncol. 1982;5(6):649-55.

13 COORDINATING INVESTIGATOR'S SIGNATURE

A Phase 3, Global, Multi-Center, Double-Blind, Randomized, Efficacy Study of IMAB362 Plus CAPOX Compared with Placebo Plus CAPOX as First-line Treatment of Subjects with Claudin (CLDN)18.2-Positive, HER2-Negative, Locally Advanced Unresectable or Metastatic Gastric or Gastroesophageal Junction (GEJ) Adenocarcinoma

ISN/Protocol 8951-CL-0302

Version 1.0

26 April 2018

I have read all pages of this clinical study protocol for which Astellas is the sponsor. I agree that it contains all the information required to conduct this study.<br>**Coordinating Investigator:**
Signature: -- ----------------------------<br>*<Insert name, department/affiliation, name of institution>* Date (DD Mmm YYYY)<br><br>Printed Name: --<br><br>Address: --<br>--

14 SPONSOR’S SIGNATURES

A Phase 3, Global, Multi-Center, Double-Blind, Randomized, Efficacy Study of Zolbetuximab (IMAB362) Plus CAPOX Compared with Placebo Plus CAPOX as First-line Treatment of Subjects with Claudin (CLDN) 18.2-Positive, HER2-Negative, Locally Advanced Unresectable or Metastatic Gastric or Gastroesophageal Junction (GEJ) Adenocarcinoma

GLOW

ISN/Protocol 8951-CL-0302

Version 5.0

Incorporating Substantial Amendment 4 [see Section 13]

18 Oct 2021

IND 129598

EudraCT 2018-000519-26

Sponsor:

Astellas Pharma Global Development, Inc. (APGD)

1 Astellas Way

Northbrook, IL 60062

Protocol History

Version 1.0 [26 Apr 2018]
Version 2.0 [29 Jun 2018] Incorporating Substantial Amendment 1
Version 3.0 [06 Aug 2019] Incorporating Substantial Amendment 2
Version 3.1 [06 Jan 2021] Incorporating Nonsubstantial Amendment 1
Version 4.0 [29 Aug 2019] Incorporating Country-specific Substantial Amendment 3 for China
Version 4.1 [11 Jan 2021] Incorporating Country-specific Nonsubstantial Amendment 2 for China

---

The information contained in this document is supplied as a background for clinical investigations. This document contains confidential information which is the intellectual property of Astellas. By accepting or reviewing this document, you agree to hold this information in confidence and not copy or disclose it to others or use it for unauthorized purposes except (1) as otherwise agreed to in writing; (2) where required by applicable law; (3) where disclosure is directly related to the care and safety of the research participant; and (4) where disclosure of such information is made to a member of the investigator's team who agrees to hold this information in confidence.

TABLE OF CONTENTS

I. SIGNATURES 9

II. CONTACT DETAILS OF KEY SPONSOR'S PERSONNEL 12

III. LIST OF ABBREVIATIONS AND DEFINITION OF KEY TERMS 13

IV. SYNOPSIS 17

V. FLOW CHART, STUDY SCHEMATICS AND SCHEDULE OF ASSESSMENTS 32

1 INTRODUCTION 43

1.1 Background 44

1.2 Nonclinical and Clinical Data 45

1.2.1 Nonclinical Data 45

1.2.2 Clinical Data 46

1.3 Summary of Key Safety Information for Study Drugs 47

1.4 Efficacy 48

1.4.1 Efficacy Results from Study GM-IMAB-001-02 (MONO) 48

1.4.2 Efficacy Results from Study GM-IMAB-001-03 (FAST) 48

1.4.3 Efficacy of CAPOX 49

1.5 Risk Benefit Assessment 49

2 STUDY OBJECTIVE(S), DESIGN AND ENDPOINTS 50

2.1 Study Objective(s) 50

2.1.1 Primary Objectives 50

2.1.2 Secondary Objectives 50

2.1.3 Exploratory Objectives 51

2.2 Study Design and Dose Rationale 51

2.2.1 Study Design 51

2.2.2 Dose Rationale 54

2.3 Endpoints 55

2.3.1 Primary Endpoints 55

2.3.2 Secondary Endpoints 55

2.3.3 Exploratory Endpoints 55

3 STUDY POPULATION 56

3.1 Selection of Study Population 56

3.2 Inclusion Criteria 56

3.3 Exclusion Criteria 58
4 **IDENTIFICATION OF STUDY TREATMENT(S) 60**
4.1 Zolbetuximab (Investigational Product) 60
4.2 0.9% Sodium Chloride Injection 60
4.3 Comparative Drug (Placebo) 61
4.4 CAPOX (Backbone Treatment) 61
4.5 Other Drug(s) 61
4.5.1 Antiemetic Premedication 61
4.6 Study Drug Handling 62
4.7 Blinding 62
4.7.1 Blinding Method 62
4.7.2 Confirmation of the Indistinguishability of the Study Drugs 63
4.7.3 Retention of the Assignment Schedule and Procedures for Treatment Code Breaking 63
4.7.4 Breaking the Treatment Code for Emergency 63
4.7.5 Breaking the Treatment Code by the Sponsor 64
4.8 Assignment and Allocation 64
5 **TREATMENTS AND EVALUATION 64**
5.1 Dosing and Administration of Study Drug(s) and Other Medication(s) 64
5.1.1 Dose/Dose Regimen and Administration Period 64
5.1.1.1 Zolbetuximab/Placebo 64
5.1.1.2 Antiemetics 64
5.1.1.3 CAPOX 65
5.1.2 Study Treatment Dose Modifications, Delays And Interruptions 66
5.1.2.1 Increase or Reduction of zolbetuximab/Placebo 66
5.1.2.2 Zolbetuximab/Placebo Interruption or Permanent Discontinuation 66
5.1.2.3 Guidelines for Infusion-related Reactions for Zolbetuximab/Placebo 69
5.1.2.4 CAPOX Dose Modification 70
5.1.2.5 CAPOX: Dose Modifications for Hematologic Toxicity 71
5.1.2.6 CAPOX: Dose Modification for Non-hematologic Toxicity 74
5.1.2.7 Guidelines for Infusion-related Reactions for Oxaliplatin 76
5.1.2.8 Oxaliplatin-induced Neurotoxicity 76
5.1.2.9 Oxaliplatin-induced Laryngopharyngeal Dysesthesia 77
5.1.2.10 Allergic Reaction to Oxaliplatin 78

5.1.2.11 Extravasation of Oxaliplatin 79
5.1.3 Treatment Compliance 79
5.1.4 Criteria for Continuation of Treatment 79
5.1.4.1 Discontinuation of CAPOX (both components) and Continuation of Zolbetuximab/Placebo 79
5.1.4.2 Discontinuation of Zolbetuximab/Placebo and Continued of CAPOX (one or both components) 79
5.1.4.3 If Both Zolbetuximab/Placebo and CAPOX (both components) are discontinued 79
5.1.5 Previous and Concomitant Treatment (Medication and Non-Medication Therapy) 80
5.2 Demographics and Baseline Characteristics 81
5.2.1 Demographics 81
5.2.2 Medical History 81
5.2.3 Diagnosis of the Target Disease, Severity, and Duration of Disease 81
5.3 Efficacy Assessments 81
5.4 Safety Assessment 82
5.4.1 Vital Signs 82
5.4.2 Observation Period following Zolbetuximab/Placebo Infusion 82
5.4.3 Laboratory Assessments 83
5.4.4 Physical Examination 85
5.4.5 Electrocardiogram 85
5.4.6 Performance Status 85
5.5 Adverse Events and Other Safety Aspects 85
5.5.1 Definition of Adverse Events 86
5.5.1.1 Abnormal Laboratory Findings 86
5.5.1.2 Potential Cases of Drug-Induced Liver Injury 86
5.5.1.3 Disease Progression and Study Endpoints 87
5.5.2 Definition of Serious Adverse Events 87
5.5.2.1 Important Medical Events 88
5.5.3 Criteria for Causal Relationship to Study Treatment 88
5.5.4 Criteria for Defining the Severity of an Adverse Event 89
5.5.5 Reporting of Serious Adverse Events 89
5.5.6 Follow-up of Adverse Events 91
5.5.7 Monitoring of Common Serious Adverse Events 91
5.5.8 Adverse Events of Special Interest 92

5.5.9 Special Situations 92
5.5.9.1 Pregnancy 92
5.5.9.2 Medication Error, Overdose and “Off-Label Use” 93
5.5.9.3 Misuse/Abuse 93
5.5.9.4 Occupational Exposure 94
5.5.9.5 Suspected Drug-Drug Interaction 94
5.5.10 Supply of New Information Affecting the Conduct of the Study 94
5.5.11 Urgent Safety Measures 95
5.5.12 Reporting Urgent Safety Measures 95
5.6 Test Drug Concentration 95
5.7 Other Measurements, Assessments or Methods 95
5.7.1 Biomarkers 95
5.7.2 Blood, Serum and Plasma Samples 96
5.7.3 Tumor Tissue Samples 96
5.7.4 Immunogenicity Assessment (Anti-drug Antibody) 97
5.7.5 Optional Samples for Banked Pharmacogenomics Sample Analysis 97
5.7.6 Electronic Clinical Outcome Assessments 97
5.7.6.1 Quality of Life Questionnaire C30 98
5.7.6.2 Oesophago-Gastric Module 25 plus STO22 Belching Subscale 98
5.7.6.3 Global Pain 98
5.7.6.4 EuroQOL Five Dimensions Questionnaire 98
5.7.6.5 Health Resource Utilization 98
5.8 Total Amount of Blood 98
6 DISCONTINUATION 99
6.1 Discontinuation of Individual Subject(s) From Study Treatment 99
6.1.1 Lost to Follow Up 100
6.2 Discontinuation of the Site 101
6.3 Discontinuation of the Study 101
7 STATISTICAL METHODOLOGY 101
7.1 Sample Size 101
7.2 Analysis Sets 102
7.2.1 Full Analysis Set 102
7.2.2 Safety Analysis Set 102
7.2.3 Pharmacokinetic Analysis Set (PKAS) 102

7.3 Demographics and Baseline Characteristics 102
7.3.1 Subject Disposition 102
7.3.2 Previous and Concomitant Medications 102
7.3.3 Medical History 102
7.4 Analysis of Efficacy 103
7.4.1 Analysis of Primary Endpoint 103
7.4.1.1 Primary Analysis 103
7.4.1.2 Sensitivity Analysis 103
7.4.1.3 Subgroup Analysis 103
7.4.2 Analysis of Secondary Endpoints 104
7.4.2.1 Overall Survival 104
7.4.2.2 PF, OG25-Pain Plus STO22, and GHS/QoL Scores 104
7.4.2.3 Objective Response Rate 105
7.4.2.4 Duration of Response 105
7.4.2.5 Health-Related Quality of Life 106
7.4.3 Analysis of Exploratory Endpoints 106
7.4.3.1 Time to Progression 106
7.4.3.2 Progression Free Survival After Subsequent Therapy (PFS2) 106
7.4.3.3 Disease Control Rate 106
7.4.3.4 Biomarkers 106
7.4.3.5 Health Resource Utilization 106
7.5 Analysis of Safety 107
7.5.1 Adverse Events 107
7.5.2 Laboratory Assessments 107
7.5.3 Vital Signs 107
7.5.4 Routine 12-lead Electrocardiograms 107
7.5.5 Eastern Cooperative Oncology Group Performance Status 107
7.6 Analysis of Pharmacokinetics 108
7.6.1 Serum Concentrations 108
7.6.2 Immunogenicity 108
7.7 Major Protocol Deviations and Other Analyses 108
7.8 Interim Analysis (and Early Discontinuation of the Clinical Study) 108
7.9 Handling of Missing Data, Outliers, Visit Windows, and Other Information 109
8 OPERATIONAL CONSIDERATIONS 109

8.1 Procedure for Clinical Study Quality Control 109
8.1.1 Data Collection 109
8.1.1.1 Collection of Data Via Electronic Source 109
8.1.1.2 Electronic Clinical Outcomes Assessment 110
8.2 Major Protocol Deviations 110
9 END OF TRIAL 111
10 STUDY ORGANIZATION 111
10.1 Independent Data Monitoring Committee 111
10.2 Other Study Organization 111
11 REFERENCES 112
12 APPENDICES 115
12.1 Ethical, Regulatory, and Study Oversight Considerations 115
12.1.1 Ethical Conduct of the Study 115
12.1.2 Institutional Review Board (IRB)/Independent Ethics Committee (IEC)/Competent Authorities (CA) 115
12.1.3 Protocol Amendment and/or Revision 115
12.1.4 Informed Consent of Subjects 116
12.1.4.1 Subject Information and Consent 116
12.1.4.2 Supply of New and Important Information Influencing the Subject’s Consent and Revision of the Written Information 116
12.1.5 Source Documents 117
12.1.6 Record Retention 117
12.1.7 Subject Confidentiality and Privacy 117
12.1.8 Arrangement for Use of Information and Publication of the Clinical Study 118
12.1.9 Insurance of Subjects and Others (UNIQUE TO JAPAN REGION/STUDIES ENROLLING SUBJECTS IN EU) 118
12.1.10 Signatory Investigator for Clinical Study Report 119
12.2 Procedure for Clinical Study Quality Control 120
12.2.1 Clinical Study Monitoring 120
12.2.2 Direct Access to Source Data/Documents 120
12.2.3 Data Management 120
12.2.4 QUALITY ASSURANCE 120
12.3 Contraception Requirements 121
12.4 Concomitant Medication Restrictions or Requirements 123

12.5 Liver Safety Monitoring and Assessment 125
12.6 Common Serious Adverse Events 128
12.7 Pharmacogenomic Analysis With Banked Sample 129
12.8 Eastern Cooperative Oncology Group Performance Status Scale 131
12.9 Clinical Study Continuity 132
13 ATTACHMENT 1: SUBSTANTIAL AMENDMENT 4 148
14 COORDINATING INVESTIGATOR'S SIGNATURE 153
15 SPONSOR'S SIGNATURES 154

List of In-text Tables

Table 1 Schedule of Assessments 36
Table 2 Guidelines for Zolbetuximab/Placebo Treatment Modification Due to Non-hematologic Toxicity 67
Table 3 Guidelines for Zolbetuximab/Placebo Treatment Modification Due to Hematologic Toxicity 68
Table 4 Infusion-related Reactions 70
Table 5 Recommended Dose Adjustment Levels for Oxaliplatin and Capecitabine 71
Table 6 CAPOX Dose Modification Due to Hematologic Toxicity 72
Table 7 CAPOX Dose Modification Due to Non-hematologic Toxicity 75
Table 8 Oxaliplatin Dose Modification for Associated Neurotoxicity 77
Table 9 Comparison of the Symptoms and Treatment of Laryngopharyngeal Dysesthesia and Platinum Hypersensitivity Reactions 78
Table 10 Alternative Schedule of Assessments in Response to a Crisis 134

I. SIGNATURES

1. SPONSOR'S SIGNATURES

Required signatures (e.g., Protocol authors and contributors, etc.) are located in [Section 15 Sponsor's Signatures].

2. COORDINATING INVESTIGATOR'S SIGNATURE

The Coordinating Investigator's signature can be found in [Section 14 Coordinating Investigator's Signature]; located at the end of this document.

3. INVESTIGATOR'S SIGNATURE

A Phase 3, Global, Multi-Center, Double-Blind, Randomized, Efficacy Study of Zolbetuximab (IMAB362) Plus CAPOX Compared with Placebo Plus CAPOX as First-line Treatment of Subjects with Claudin (CLDN)18.2-Positive, HER2-Negative, Locally Advanced Unresectable or Metastatic Gastric or Gastroesophageal Junction (GEJ) Adenocarcinoma

ISN/Protocol 8951-CL-0302

Version 5.0 Incorporating Substantial Amendment 4

18 Oct 2021

I have read all pages of this clinical study protocol for which Astellas is the sponsor. I agree to conduct the study as outlined in the protocol and to comply with all the terms and conditions set out therein. I confirm that I will conduct the study in accordance with International Council for Harmonisation (ICH) Good Clinical Practice (GCP) guidelines and applicable local regulations. I will also ensure that subinvestigator(s) and other relevant members of my staff have access to copies of this protocol and the ICH GCP guidelines to enable them to work in accordance with the provisions of these documents.
**Principal Investigator**:<br><br>Signature: ---<br>Date (DD Mmm YYYY)<br><br>Printed Name: ---<br>*<Insert name and qualification of the Investigator>*<br><br>Address: --<br>--

II. CONTACT DETAILS OF KEY SPONSOR'S PERSONNEL

**24h-Contact for Serious Adverse Events (SAEs)**<br><br>See [Section 5.5.5 Reporting of Serious Adverse Events] for SAE Fax Number and Email	**Please fax or email the SAE Worksheet to:**<br>Astellas Pharma Global Development, Inc.<br>Global Pharmacovigilance<br><br>**North America Fax number**: +1-888-396-3750<br>North America Alternate Fax number: +1-847-317-1241<br><br>**International Fax Number**: +44-800-471-5263<br>**Email: safety-us@astellas.com**<br><br>**Japan investigational sites:**<br>PAREXEL International<br>Global Monitoring Operations<br>Fax: 03-6888-5798
Medical Monitor/Study Physician:	PPD [redacted]<br>Astellas Pharma Global Development, Inc.<br>1 Astellas Way, Northbrook, Illinois 60062<br>PPD [redacted]
Clinical Research Contacts:	PPD [redacted]<br>Astellas Pharma Global Development, Inc.<br>1 Astellas Way, Northbrook, Illinois 60062<br>PPD [redacted]
Clinical Research Contacts Japan:	Corporate Name: Astellas Pharma Inc.<br>Location: 2-5-1, Nihonbashi-Honcho, Chuo-ku, Tokyo<br>PPD [redacted]

III. LIST OF ABBREVIATIONS AND DEFINITION OF KEY TERMS

List of Abbreviations

Abbreviations	Description of abbreviations
5-FU	fluorouracil
ADA	anti-drug antibody
ADCC	antibody-dependent cell-mediated cytotoxicity
AE	adverse event
ALP	alkaline phosphatase
ALT	alanine aminotransferase
ANC	absolute neutrophil count
APEBV	Astellas Pharma Europe B.V.
AST	aspartate aminotransferase
βhCG	beta human chorionic gonadotropin
C1D1	Cycle 1 Day 1
CA	Competent Authorities
CAPOX	capecitabine and oxaliplatin
CDC	complement-dependent cytotoxicity
CE	Conformité Europèene
CI	confidence interval
CLDN	claudin
CMH	Cochran-Mantel-Haenszel
CR	complete response
CRO	contract research organization
CT	computerized tomography
CTCAE	Common Terminology Criteria For Adverse Events
DCR	disease control rate
DOR	duration of response
DPD	dihydropyrimidine dehydrogenase
ECG	electrocardiogram
ECL	electrochemiluminescense
eCOA	Electronic Clinical Outcomes Assessment
ECOG	Eastern Cooperative Oncology Group
eCRF	electronic case report form
EEA	European Economic Area
EORTC	European Organization for Research and Treatment of Cancer
EOX	epirubicin, oxaliplatin and capecitabine
EQ5D-5L	EuroQOL Five Dimensions Questionnaire 5L

Abbreviations	Description of abbreviations
EU	European Union
FAS	full analysis set
FIM	first-in-human
FFPE	formalin-fixed paraffin embedded
GCP	Good Clinical Practices
GE	gastroesophageal
GEJ	gastroesophageal junction
GHS/QoL	Global Health Status/Quality of Life
GMP	Good Manufacturing Practices
GP	Global Pain
Hgb	hemoglobin
HEOR	health economics and outcomes research
HER2	human epidermal growth factor receptor 2
HIPAA	Health Insurance Portability and Accountability Act
HIV	human immunodeficiency virus
HRQoL	health-related quality of life
HRU	Health Resource Utilization
HSR	hypersensitivity reactions
IB	Investigator's Brochure
ICF	informed consent form
ICH	international council for harmonisation of technical requirements for registration of pharmaceuticals for human use
IDMC	independent data monitoring committee
IEC	independent ethics committee
IND	investigational new drug
IHC	immunohistochemistry
IMAB	ideal monoclonal antibody
INR	international normalized ratio
IRB	institutional review board
IRC	independent review committee
IRR	infusion-related reaction
IRT	interactive response technology
ISN	international study number
LA-CRF	liver abnormality case report form
LFT	liver function tests
mAbs	monoclonal antibodies
MRI	magnetic resonance imaging

Abbreviations	Description of abbreviations
NCCN	National Comprehensive Cancer Network
NCI	National Cancer Institute
NSAID	nonsteroidal anti-inflammatory drugs
OG25-Pain	oesophago-gastric questionnaire (OG25) on abdominal pain and discomfort
ORR	objective response rate
OS	overall survival
PD	progressive disease
PF	physical function
PFS	progression free survival
PFS2	progression free survival following subsequent anticancer treatment
PGx	pharmacogenomics
PKAS	pharmacokinetic analysis set
PR	partial response
PRES	posterior reversible encephalopathy syndrome
PT	preferred term
QLQ-C30	Quality of Life Questionnaire - Core Questionnaire
QLQ-OG25	Quality of Life Questionnaire - Oesophago-Gastric Module 25
QTc	QT corrected
RECIST	Response Evaluation Criteria In Solid Tumors
RSI	reference safety information
(S)AE	serious adverse event and/or adverse event
SAE	serious adverse event
SAF	safety analysis set
SAP	statistical analysis plan
SD	stable disease
SOP	standard operating procedure
SPC	summary of product characteristics
SUSAR	suspected unexpected serious adverse reactions
TEAE	treatment-emergent adverse event
TTCD	time to confirmed deterioration
TTP	time to progression
ULN	upper limit of normal
VEGF	vascular endothelial growth factor
WOCBP	woman of childbearing potential

Definition of Key Study Terms

**Terms**	**Definition of terms**
Baseline	Assessments of subjects as they enter a study before they receive any treatment.
Endpoint	Variable that pertains to the efficacy or safety evaluations of a study.
Enroll	To register or enter a subject into a clinical study. NOTE: Once a subject has received the study drug or placebo, the clinical study protocol applies to the subject.
Intervention	The drug, device, therapy or process under investigation in a clinical study that is believed to have an effect on outcomes of interest in a study. (e.g., health-related quality of life, efficacy, safety and pharmacoeconomics).
Investigational period	Period of time where major interests of protocol objectives are observed, and where the test drug or comparative drug (sometimes without randomization) is usually given to a subject, and continues until the last assessment after completing administration of the test drug or comparative drug.
Post investigational period	Period of time after the last assessment of the protocol. Follow-up observations for sustained adverse events and/or survival are done in this period.
Randomization	The process of assigning study subjects to treatment or control groups using an element of chance to determine assignments in order to reduce bias.
Screening	A process of active consideration of potential subjects for enrollment in a study.
Screen failure	Potential subject who did not meet 1 or more criteria required for participation in a study.
Screening period	Period of time before entering the investigational period, usually from the time when a subject signs the consent until just before the test drug or comparative drug (sometimes without randomization) is given to a subject.
Study period	Period of time from the first site initiation date to the last site completing the study.
Study Treatment	Includes zolbetuximab/placebo and both components of CAPOX
Variable	Any entity that varies; any attribute, phenomenon or event that can have different qualitative or quantitative values.

IV. SYNOPSIS

**Date and Version No of Protocol Synopsis:**	18 Oct 2021, Version 5.0
**Sponsor:**<br>Astellas Pharma Global Development Inc. (APGD)	**Protocol Number:**<br>8951-CL-0302
**Name of Study Drug:**<br>Zolbetuximab (IMAB362)	**Phase of Development:**<br>Phase 3

Title of Study:

A Phase 3, Global, Multi-Center, Double-Blind, Randomized, Efficacy Study of Zolbetuximab (IMAB362) Plus CAPOX Compared with Placebo Plus CAPOX as First-line Treatment of Subjects with Claudin (CLDN)18.2-Positive, HER2-Negative, Locally Advanced Unresectable or Metastatic Gastric or Gastroesophageal Junction (GEJ) Adenocarcinoma

Planned Study Period:

From 4Q2018 to 3Q2023

Study Objective(s):

Primary

- To evaluate the efficacy of zolbetuximab plus capecitabine and oxaliplatin (CAPOX) compared with placebo plus CAPOX (as first-line treatment) as measured by Progression Free Survival (PFS) in subjects with Claudin (CLDN) 18.2-positive, human epidermal growth factor receptor 2 (HER2)-negative locally advanced unresectable or metastatic gastric and gastroesophageal junction (GEJ) adenocarcinoma

Secondary

- To evaluate efficacy as measured by Overall Survival (OS) as a key secondary objective
- To evaluate the physical function (PF), OG25-Pain and GHS/QoL scores as measured by European Organization for Research and Treatment of Cancer (EORTC) as a key secondary objective
- To evaluate efficacy as measured by Objective Response Rate (ORR)
- To evaluate efficacy as measured by Duration of Response (DOR)
- To evaluate safety and tolerability of zolbetuximab
- To further evaluate other health related quality of life (HRQoL) using additional parameters as measured by EORTC QLQ-C30 and QLQ-OG25 plus STO22 Belching subscale, Global Pain (GP) and the EuroQOL Five Dimensions Questionnaire 5L (EQ5D-5L) questionnaires
- To evaluate the pharmacokinetics of zolbetuximab
- To evaluate the immunogenicity profile of zolbetuximab

Exploratory

- To evaluate efficacy as measured by Time to Progression (TTP)
- To evaluate PFS following subsequent anticancer treatment (PFS2)
- To evaluate Disease Control Rate (DCR)
- To evaluate potential genomic and/or other biomarkers that may correlate with treatment outcome to zolbetuximab and CAPOX.
- To evaluate Health Resource Utilization (HRU)

Planned Total Number of Study Centers and Location(s):

Approximately 175 centers globally

**Study Population:**<br>Subjects with locally advanced unresectable or metastatic gastric or GEJ adenocarcinoma whose tumors are CLDN18.2-positive, HER2-negative and who have not been previously treated for metastatic disease with chemotherapy (first-line).<br>For the purpose of this study, CLDN18.2-positive is defined as CLDN18.2 expression in ≥ 75% of tumor cells demonstrating moderate to strong membranous staining as determined by central immunohistochemistry (IHC) testing.
**Number of Subjects to be Enrolled/Randomized:**<br>Approximately 500 subjects
**Study Design Overview:**<br>This global, multicenter, double-blind, 1:1 randomized, phase 3 study will evaluate efficacy of zolbetuximab plus CAPOX versus placebo plus CAPOX as first-line treatment in subjects with CLDN18.2-positive, HER2-negative locally advanced unresectable or metastatic gastric and GEJ adenocarcinoma.<br>PFS as assessed by the Independent Review Committee (IRC) is the primary outcome. Secondary outcomes include OS, ORR, DOR, safety and tolerability, HRQoL, pharmacokinetics and the immunogenicity profile of zolbetuximab. Exploratory outcomes include TTP, PFS2, DCR, biomarkers, and HRU.<br>Approximately 500 subjects will be randomized 1:1 into 1 of 2 treatment arms:<br>• Arm A (zolbetuximab in combination with CAPOX chemotherapy)<br>• Arm B (placebo in combination with CAPOX chemotherapy)<br>Randomization of subjects will be stratified by the following factors:<br>• Region (Asia vs Non-Asia)<br>• Number of Organs with Metastatic Sites (0 to 2 vs ≥ 3)<br>• Prior Gastrectomy (Yes or No)<br>*Screening:*<br>The Screening period is 45 days from full main informed consent form (ICF) signature. Retesting of lab values is allowed within the 45-day Screening period. Re-screening outside the 45-day window under a new subject number may be allowed once and upon discussion with the Medical Monitor. Computerized tomography (CT) scans and magnetic resonance imaging (MRI) conducted as part of a subject's routine clinical management (i.e., standard of care) obtained before signing the ICF may be utilized for screening or baseline purposes, provided the procedures met the protocol-specified criteria and were performed within the Screening period.<br>Formalin fixed paraffin embedded (FFPE) tumor tissue will be collected for central testing to determine CLDN18.2 and HER2 status.<br>An optional partial screening ICF may be available to allow central testing of tissue for CLDN18.2 and HER2 only.<br>• Archival tumor tissue is preferred.<br>o A minimum of 1 FFPE tumor tissue block (preferred) OR a minimum* of 15 FFPE unstained slides are required as allowed per local policy. If slides are submitted, the slides should be freshly cut from the FFPE block within the time frame described in the laboratory manual.

- If local HER2 results are already available from local testing, a minimum* of 12 FFPE unstained slides are required to be submitted to the central laboratory as allowed per local policy.

*If the required minimum number of slides is not able to be submitted, sponsor notification and approval is required.

- If the specimen is insufficient or unavailable, a biopsy may be performed to obtain tumor sample.
  - Sponsor pre-approval is required when the sole purpose of the biopsy procedure is to assess eligibility for this study.
  - If the required number of slides cannot be provided, the sponsor or designee should be contacted for further guidance.

Treatment Period:

Subjects will be treated with either zolbetuximab (Arm A) or placebo (Arm B) on day 1 of each cycle until the subject meets study treatment discontinuation criteria. For all study treatments, a cycle is defined as approximately 21 days.

Subjects will also receive up to 8 treatments of CAPOX treatment. Oxaliplatin is administered on day 1 of each cycle, whereas capecitabine is taken twice daily on days 1 through 14. After a maximum of 8 treatments of oxaliplatin, subjects may continue to receive capecitabine taken twice daily on days 1 through 14 of each cycle at the investigator's discretion until the subject meets study treatment discontinuation criteria. (NOTE: An ECG is required to be performed and assessed locally prior to every oxaliplatin infusion [before any antiemetic treatment] and following completion of every oxaliplatin infusion. ECG should be performed up to 48 hours *prior* to and up to 6 hours *following* every oxaliplatin infusion. Oxaliplatin administration and electrolyte levels should be managed according to investigator judgment for subjects with grade 1 or 2 hypokalemia, hypomagnesemia and/or hypocalcemia.)

Radiologic imaging will be evaluated every 9 weeks (± 7 days) counting from cycle 1/day 1 (C1D1) for the first 54 weeks and every 12 weeks (± 7 days) thereafter until subject develops radiological disease progression per Response Evaluation Criteria In Solid Tumors (RECIST) 1.1 by IRC or starts other systemic anticancer treatment, whichever occurs earlier.

If a subject discontinues CAPOX (or oxaliplatin only) due to any reason other than disease progression as confirmed by IRC, they may continue on zolbetuximab/placebo at the discretion of the investigator provided that all of the following have been met:

- the subject completed at least 2 cycles of CAPOX treatment;
- the subject will not receive another systemic chemotherapy, immunotherapy, radiotherapy or other treatment intended for antitumor activity; and
- in the investigator's opinion, the subject continues to derive clinical benefit with acceptable toxicity.

Subjects should continue to follow the Study Treatment Period Schedule of Assessments [Table 1].

Safety Assessments:

Safety will be evaluated based on adverse events (AEs), vital signs, ECGs, physical exams, Eastern Cooperative Oncology Group (ECOG) performance status and laboratory assessments. Severity of AEs and laboratory abnormalities will be assessed based on National Cancer Institute-Common Terminology Criteria For Adverse Events (NCI-CTCAE) v4.03.

Radiologic Imaging and Independent Review Committee:

Radiologic imaging will be evaluated at Screening (within 28 days prior to randomization) and every 9 weeks (±7 days) counting from C1D1 for the first 54 weeks and then every 12 weeks (±7 days) thereafter until subject develops radiological disease progression per RECIST 1.1 by IRC or starts other systemic anticancer treatment, whichever comes earlier.

All radiologically evaluable disease (measurable and/or non-measurable), per local assessment, must be documented at Screening and re-assessed at each subsequent radiologic evaluation. Imaging will include CT scans with contrast of the thorax, abdomen, and pelvis. If CT scan with contrast is medically not feasible, magnetic resonance imaging (MRI) may be used for imaging. Bone scans (or focal X-ray) or brain imaging should be performed if metastatic disease in bone or brain is suspected, respectively. The same mode of imaging should be utilized throughout the study unless medical necessity requires a change. For randomized subjects, screening imaging should be sent to the central imaging vendor no later than at the time of submission of the first on-treatment imaging. All imaging acquired post randomization will be sent to the central imaging vendor within 7 days of scanning for the blinded IRC assessment of radiological tumor response based on RECIST 1.1 [Eisenhauer et al, 2009]. The investigator should make every effort to immediately submit radiologic assessments for IRC review when progressive disease (PD) is suspected.

Study Treatment Discontinuation and Safety Follow-up Visits:

Following discontinuation from zolbetuximab/placebo, subjects will have a zolbetuximab/placebo Study Treatment Discontinuation Visit within 7 days after the decision to discontinue, and 30-day and 90-day Safety Follow-up Visits after their last dose of zolbetuximab/placebo.

Additionally, if CAPOX (both components) is discontinued on a different day than zolbetuximab/placebo, subjects will also have a Study Treatment Discontinuation Visit within 7 days after the decision to discontinue, and 30-day and 90-day Safety Follow-up Visits after the last dose of CAPOX (both components). The CAPOX 30-day and 90-day Safety Follow-up Visits may be conducted by telephone if the subject is unable to visit the study site and will require contact for AE/SAE collection only.

Post-treatment Follow-up Period (for PFS):

If a subject discontinues all study treatments (zolbetuximab/placebo and both components of CAPOX) prior to IRC-confirmed radiological disease progression, the subject will enter the Post-treatment Follow-up Period and continue to undergo scheduled imaging assessments every 9 weeks (±7 days) (or every 12 weeks [±7 days] if subject has been on study over 54 weeks) until radiologic disease progression (i.e., PFS event) per IRC, or until the subject starts any other anticancer treatment, whichever occurs earlier.

If study treatments (zolbetuximab/placebo and both components of CAPOX) are discontinued due to disease progression (PFS event), the subject will enter the Long-term and Survival Follow-up Period.

Long-term Follow-up Period (for PFS2) and Survival Follow-up (for OS) Period:

Following disease progression on first-line treatment or start of any other anticancer treatment, subjects will be followed in the Long-term and Survival Follow-up Period per institutional guidelines, but not less than every 12 weeks. Subsequent anticancer treatment details, progression status and survival status will be collected until PD following subsequent anticancer therapy (PFS2) is documented, or the subject starts another systemic anticancer treatment, whichever occurs earlier. Radiologic imaging for PFS2 will be done per local standard of care and read locally. Subjects will continue to be followed for survival status (OS) in the Survival Follow-up Period until death (from any cause).

All post-progression details including subsequent anticancer treatment and date and site of progression will be recorded on the electronic case report form (eCRF). Subject contact by phone or other remote method is sufficient during Long-term and Survival Follow-up. Additional follow-up contacts may be required per sponsor request for analysis purposes.

Biomarkers and Other Sampling:

Samples for pharmacokinetics, immunogenicity and biomarkers, as well as FFPE tumor tissue specimens will be collected. Optional pharmacogenomics and post-progression tumor samples may be collected for those subjects who sign a separate informed consent form (ICF).

Electronic Clinical Outcomes Assessments (HRQoL and HRU):

HRQoL and HRU should be assessed during the visit (or up to 48 hours) before any antiemetic or drug treatment and before the disease status is discussed with the subject. Assessments will be collected at Screening (except for HRU), every 3 weeks, at study treatment discontinuation and 30 and 90 days post zolbetuximab/placebo treatment. HRQoL will be measured by EORTC QLQ-C30, QLQ-OG25 plus STO22 Belching subscale, GP and the EQ5D-5L.

Independent Data Monitoring Committee and Independent Data Analysis Center:

An Independent Data Monitoring Committee (IDMC) will be established and will monitor the ongoing benefit-risk status of study treatment in an unblinded fashion per a pre-defined IDMC charter. The first IDMC meeting will be approximately 6 weeks after the 40th subject enrolled has completed or discontinued cycle 2 (6 weeks) and meetings will be conducted thereafter, as defined in the IDMC charter.

An Independent Data Analysis Center will conduct an interim analysis of OS at the same time as the final PFS analysis, which will occur when approximately 300 PFS events have occurred. This analysis will be utilized by the IDMC to recommend whether the study should be stopped earlier than planned if zolbetuximab in combination with CAPOX has a favorable outcome compared with placebo in combination with CAPOX. If the OS interim analysis demonstrates a highly more favorable outcome for zolbetuximab in combination with CAPOX, the study may be stopped for success. However, any subject continuing to derive clinical benefit from zolbetuximab/placebo in combination with CAPOX, as assessed by the investigator, will be allowed to continue treatment.

Inclusion/Exclusion Criteria:

Inclusion Criteria:

Waivers to the inclusion criteria will **NOT** be allowed.

General Criteria:

1. Institutional Review Board (IRB)/Independent Ethics Committee (IEC) approved written informed consent and privacy language as per national regulations (e.g., Health Insurance Portability and Accountability Act [HIPAA] Authorization for US sites) must be obtained from the subject or legally authorized representative (if applicable) prior to any study-related procedures.
2. Subject is considered an adult (e.g., ≥ 18 years of age in the US) according to local regulation at the time of signing the informed consent.
3. A female subject is eligible to participate if she is not pregnant (negative serum pregnancy test at screening; female subjects with elevated serum beta human chorionic gonadotropin (βhCG) and a demonstrated non-pregnant status through additional testing are eligible) and at least 1 of the following conditions applies:
    - Not a woman of childbearing potential (WOCBP) as defined in [Appendix 12.3 Contraception Requirements]

    OR

- WOCBP who agrees to follow the contraceptive guidance as defined in [Appendix 12.3 Contraception Requirements] throughout the treatment period and for 9 months after the final administration of oxaliplatin and 6 months after the final administration of all other study drugs.

4. Female subject must agree not to breastfeed starting at screening and throughout the study period, and for 6 months after the final study treatment administration.
5. Female subject must not donate ova starting at screening and throughout the study period, and for 9 months after the final administration of oxaliplatin and 6 months after the final administration of all other study drugs.
6. A male subject with female partner(s) of childbearing potential:
   - must agree to use contraception as detailed in [Appendix 12.3 Contraception Requirements] during the treatment period and for 6 months after the final study treatment administration.
7. A male subject must not donate sperm during the treatment period and for 6 months after the final study treatment administration.
8. Male subject with a pregnant or breastfeeding partner(s) must agree to remain abstinent or use a condom for the duration of the pregnancy or time partner is breastfeeding throughout the study period and for 6 months after the final study treatment administration.
9. Subject agrees not to participate in another interventional study while receiving study drug in present study.

Disease Specific Criteria:

10. Subject has histologically confirmed diagnosis of Gastric or GEJ adenocarcinoma.
11. Subject has radiologically confirmed locally advanced unresectable or metastatic disease within 28 days prior to randomization.
12. Subject has radiologically evaluable disease (measurable and/or non-measurable) according to RECIST 1.1, per local assessment, ≤ 28 days prior to randomization. For subjects with only 1 evaluable lesion and prior radiotherapy ≤ 3 months before randomization, the lesion must either be outside the field of prior radiotherapy or have documented progression following radiation therapy.
13. Subject's tumor expresses CLDN18.2 in ≥ 75% of tumor cells demonstrating moderate to strong membranous staining as determined by central IHC testing.
14. Subject has a HER2-negative tumor as determined by local or central testing on a gastric or GEJ tumor specimen.

Physical or Laboratory Findings:

15. Subject has ECOG performance status 0 or 1.
16. Subject has predicted life expectancy ≥ 12 weeks in the opinion of the investigator.
17. Subject must meet all of the following criteria based on the centrally or locally analyzed laboratory tests collected within 14 days prior to randomization. In the case of multiple sample collections within this period, the most recent sample collection with available results should be used to determine eligibility.
    a. Hemoglobin (Hgb) ≥ 9 g/dL. Subjects requiring transfusions are eligible if they have a post-transfusion Hgb ≥ 9 g/dL.
    b. Absolute Neutrophil Count (ANC) ≥ 1.5×10^9/L
    c. Platelets ≥ 100×10^9/L
    d. Albumin ≥ 2.5 g/dL
    e. Total bilirubin ≤ 1.5 x upper limit of normal (ULN) without liver metastases (or < 3.0 x ULN if liver metastases are present)

f. Aspartate aminotransferase (AST) and alanine aminotransferase (ALT) ≤ 2.5 x ULN without liver metastases (or ≤ 5 x ULN if liver metastases are present)
g. Estimated creatinine clearance ≥ 30 mL/min
h. Prothrombin time/international normalized ratio (PT/INR) and partial thromboplastin time (PTT) ≤ 1.5 x ULN (except for subjects receiving anticoagulation therapy)

Exclusion Criteria:

Waivers to the exclusion criteria will **NOT** be allowed.

Subject who meets any of the following exclusion criteria prior to enrollment is not eligible for enrollment:

Prohibited Treatment or Therapies:

1. Subject has received prior systemic chemotherapy for locally advanced unresectable or metastatic gastric or GEJ adenocarcinoma. However, subject may have received either neo-adjuvant or adjuvant chemotherapy, immunotherapy or other systemic anticancer therapies as long as it was completed at least 6 months prior to randomization.
2. Subject has received radiotherapy for locally advanced unresectable or metastatic gastric or GEJ adenocarcinoma ≤ 14 days prior to randomization and has not recovered from any related toxicity.
3. Subject has received treatment with herbal medications or other treatments that have known antitumor activity within 28 days prior to randomization.
4. Subject has received systemic immunosuppressive therapy, including systemic corticosteroids within 14 days prior to randomization. Subject using a physiologic replacement dose of hydrocortisone or its equivalent (defined as up to 30 mg per day of hydrocortisone or up to 10 mg per day of prednisone), receiving a single dose of systemic corticosteroids, or receiving systemic corticosteroids as premedication for radiologic imaging contrast use is eligible.
5. Subject has received other investigational agents or devices within 28 days prior to randomization.

Medical History or Concurrent Disease:

6. Subject has prior severe allergic reaction or intolerance to known ingredients of zolbetuximab or other monoclonal antibodies, including humanized or chimeric antibodies.
7. Subject has known immediate or delayed hypersensitivity, intolerance or contraindication to any component of study treatment.
8. Subject has prior severe allergic reaction or intolerance to any component of CAPOX.
9. Subject has known dihydropyrimidine dehydrogenase (DPD) deficiency. (NOTE: Screening for DPD deficiency should be conducted per local requirements.)
10. Subject has a complete gastric outlet syndrome or a partial gastric outlet syndrome with persistent/recurrent vomiting.
11. Per investigator judgment, subject has significant gastric bleeding and/or untreated gastric ulcers that exclude the subject from participation.
12. Subject has a known history of a positive test for human immunodeficiency virus (HIV) infection or known active hepatitis B (positive HBs Ag) or C infection. NOTE: Screening for these infections should be conducted per local requirements.
    a. For subjects who are negative for HBs Ag, but HBc Ab positive, an HB DNA test will be performed and if positive, the subject will be excluded.
    b. Subjects with positive hepatitis C virus (HCV) serology but negative HCV RNA test are eligible.
    c. Subjects treated for HCV with undetectable viral load results are eligible.

13. Subject has an active autoimmune disease that has required systemic treatment within the past 3 months prior to randomization.
14. Subject has active infection requiring systemic therapy that has not completely resolved within 7 days prior to randomization.
15. Subject has significant cardiovascular disease, including any of the following:
    a. Congestive heart failure (defined as New York Heart Association [NYHA] Class III or IV), myocardial infarction, unstable angina, coronary angioplasty, coronary stenting, coronary artery bypass graft, cerebrovascular accident (CVA), or hypertensive crisis within 6 months prior to randomization;
    b. History of clinically significant ventricular arrhythmias (i.e., sustained ventricular tachycardia, ventricular fibrillation, or Torsades de Pointes);
    c. QTc interval > 450 msec for male subjects; QTc interval > 470 msec for female subjects;
    d. History or family history of congenital long QT syndrome
    e. Cardiac arrhythmias requiring anti-arrhythmic medications (Subjects with rate controlled atrial fibrillation for > 1 month prior to randomization are eligible.)
16. Subject has a history of central nervous system (CNS) metastases and/or carcinomatous meningitis from gastric/GEJ cancer.
17. Subject has known peripheral sensory neuropathy > grade 1 unless the absence of deep tendon reflexes is the sole neurological abnormality.
18. Subject has had a major surgical procedure ≤ 28 days prior to randomization.
    a. Subject is without complete recovery from a major surgical procedure ≤ 14 days prior to randomization.
19. Subject has psychiatric illness or social situations that would preclude study compliance, per investigator judgment.
20. Subject has another malignancy for which treatment is required per investigator's clinical judgment
21. Subject has any concurrent disease, infection, or co-morbid condition that interferes with the ability of the subject to participate in the study, which places the subject at undue risk or complicates the interpretation of data in the opinion of the investigator.

Investigational Product(s):

**Zolbetuximab/ Placebo:**	Zolbetuximab: The investigational product, zolbetuximab, is a sterile lyophilized powder preparation with the chimeric monoclonal antibody zolbetuximab as the active pharmaceutical ingredient.<br>Each vial contains 105 mg of zolbetuximab and has to be reconstituted with 5.0 mL sterile water for injection (WFI), to a concentration of 20 mg/mL. Further dilution with sterile 0.9% Sodium Chloride Injection, to a final concentration of 2 mg/mL is required.<br>Placebo: Placebo will not be manufactured or provided by the sponsor. 0.9% Sodium Chloride Injection will be used for placebo treatment arm as a placebo infusion solution.
**Dose(s):**	800 mg/m² loading dose of zolbetuximab at C1D1 followed by subsequent doses of 600 mg/m² every 3 weeks.
**Dosing Schedule:**	Subjects will be treated with either zolbetuximab (Arm A) or placebo (Arm B) on day 1 of each cycle until the subject meets study treatment discontinuation criteria.<br>Zolbetuximab/placebo should be administered after antiemetic premedication but prior to CAPOX.
**Mode of Administration:**	Intravenous infusion of zolbetuximab/placebo as a minimum 2-hour infusion.<br>Intravenous infusion may be slowed or interrupted to manage toxicity. **Please refer to Pharmacy Manual and Infusion Guidelines for more detailed information.**

Other Product(s):

**0.9% Sodium Chloride Injection**	0.9% Sodium Chloride Injection will be used for infusion solution in this study for both zolbetuximab arm and placebo arm. Details of preparation of infusion solution are provided in the Pharmacy Manual and Infusion Guidelines<br>***SPECIFIC TO JAPAN:*** In this study, 0.9% Sodium Chloride Injection is not considered an 'investigational product' as defined in J-GCP.
**CAPOX:**	Subjects will receive CAPOX treatment until IRC confirmed disease progression or a total of 8 treatments over 8 or more cycles (each cycle is defined as approximately 3 weeks = approximately 21 days). Oxaliplatin is administered on day 1 of each cycle, whereas capecitabine is taken twice daily on days 1 through 14.<br>After 8 treatments of CAPOX, subjects may continue to receive capecitabine twice daily on days 1 through 14 of each cycle at the investigator's discretion until the subject meets study treatment discontinuation criteria.<br>CAPOX should be administered after zolbetuximab/placebo infusion.
**Oxaliplatin:**	130 mg/m^2 intravenous infusion on day 1 of each cycle over 2 hours (or longer per institutional standard of care) for a maximum of 8 treatments.<br>(NOTE: ECG is required to be performed and assessed locally prior to every oxaliplatin infusion [before any antiemetic treatment] and following completion of every oxaliplatin infusion. ECG should be performed up to 48 hours *prior* to and up to 6 hours *following* every oxaliplatin infusion. Oxaliplatin administration and electrolyte levels should be managed according to investigator judgment for subjects with grade 1 or 2 hypokalemia, hypomagnesemia, and/or hypocalcemia).
**Capecitabine**	Administered orally at 1000 mg/m^2 twice daily (bid) (total daily dose is 2000 mg/m^2) on days 1 through 14 of each cycle
**<u>Antiemetic Pre-medications</u>**	Antiemetic premedication (prophylactic antiemetics) should be administered prior to each study treatment.<br>(NOTE: Subjects receiving zolbetuximab do not need to be premedicated for prevention of infusion-related reactions [IRRs]; however, subjects should be closely monitored for IRRs to facilitate early identification and management.)<br>• Antiemetic premedication<br>o IV antiemetic premedication should be initiated prior to treatment, or<br>o Oral antiemetic premedication should be initiated at a minimum of 30 minutes prior to zolbetuximab/placebo treatment.<br>• It is recommended that the prophylactic antiemetic regimen include (but is not limited to) the following agents:<br>o NK-1 receptor blockers<br>o 5-HT3 receptor blockers*<br>*To minimize the risk of Torsades de Pointes, administer 5-HT3 receptor blockers with caution to subjects who have or may develop QTc prolongation.<br>Antiemetic premedication should be administered according to institutional standard of care, published guidelines and the respective product package insert(s).<br>**<u>Corticosteroids:</u>**<br>• The impact of corticosteroids on the potential efficacy of zolbetuximab is not known. Therefore, consideration should be given to avoid or minimize the use of corticosteroids as a prophylactic antiemetic, if possible.<br>• For a subject's <u>first dose</u> of zolbetuximab/placebo, it is recommended that the prophylactic use of corticosteroids <u>be avoided</u>.

Concomitant Medication Restrictions or Requirements:

<u>Prohibited</u> Concomitant Treatment

The following are <u>strictly prohibited</u>:

- Sorivudine or analogs (during capecitabine treatment)

- Concurrent nonsteroid systemic immunosuppressive agents (for systemic corticosteroids see cautionary concomitant treatment below).
- Live vaccines should be avoided during the treatment period in which subject is receiving oxaliplatin or capecitabine and up to 6 months after final oxaliplatin or capecitabine dose. In cases where a live vaccine is needed for COVID-19 prevention and allowed per local regulations, please contact the Medical Monitor for discussion.
- Other systemic chemotherapy, immunotherapy, radiotherapy, herbal medications or other treatments intended for antitumor activity.
    - Palliative radiotherapy for peripheral bone metastases is allowed.
    - For gastric bleeding, palliative radiotherapy is prohibited, but esophagogastroduodenoscopy with epinephrine injection is allowed.
- Investigational products or therapy other than zolbetuximab.

Cautionary Concomitant Treatment

Considerations should be given to avoid or minimize the use of the following concomitant medications, if possible, during zolbetuximab/placebo treatment:

- Systemic corticosteroids, because their impact on the potential efficacy of zolbetuximab is not known.
    - Systemic corticosteroids should be avoided or minimized while subject is on study treatment unless required for management of an emergent medical condition (e.g., severe nausea/vomiting or hypersensitivity reaction).
    - For a subject's first dose of zolbetuximab/placebo, it is recommended that the prophylactic use of corticosteroids be avoided.
    - Inhaled, intranasal, ophthalmic, otic and topically applied steroids are allowed.
    - Subjects are allowed to use a physiologic replacement dose of hydrocortisone or its equivalent (defined as up to 30 mg/day of hydrocortisone or up to 10 mg/day of prednisone), receive a single dose of systemic corticosteroids or receive systemic corticosteroids as pre-medication for radiologic imaging contrast use.
- Administer 5-HT3 blockers with caution in subjects who have or may develop QTc prolongation.
- Nonsteroidal anti-inflammatory drugs (NSAIDs) because of the potential to cause gastric ulcers and covert bleeding.
    - In such cases where NSAID use is necessary, the use of NSAIDs with lower gastric ulcerogenic potential is preferred and concomitant gastric protection with proton pump inhibitors is recommended.

The following should be avoided or used with caution and closely monitored during capecitabine administration:

- CytochromeP450 (CYP) 2C9 substrates (Subjects taking coumarin-derivative anticoagulants concomitantly with capecitabine should have PT/INR monitored regularly and anticoagulant dose adjusted accordingly).
- Anti-epileptic medications (e.g. phenobarbital, phenytoin and primidone)

The following should be avoided or used with caution and closely monitored during oxaliplatin administration:

- Medications known to prolong the QT or QTc interval (refer to https://www.crediblemeds.org for a list of these medications)

Prohibited and cautionary concomitant treatments are described in Appendix 12.4.

**Duration of Treatment:**<br>Subjects will receive zolbetuximab/placebo until IRC confirmed disease progression, toxicity requiring study treatment cessation, start of another anticancer treatment, or other treatment discontinuation criteria are met.<br>Subjects will also receive up to 8 treatments of CAPOX followed by continued use of capecitabine (based on investigator's judgment) beyond 8 treatments until treatment discontinuation criteria are met.
**Study Treatment Discontinuation Criteria:**<br>A subject who enrolled in the study and for whom study treatment (zolbetuximab/placebo and both components of CAPOX) is permanently discontinued for any reason will be assessed as having met study treatment discontinuation criteria.<br>As overall survival is the key secondary end-point of the study, all subjects will be followed for survival after meeting study treatment discontinuation criteria unless the subject withdraws consent or is considered lost to follow-up after repeated attempts to contact or if the sponsor discontinues the study.<br>A subject is free to withdraw from the study treatment and/or study for any reason and at any time without giving reason for doing so and without penalty or prejudice. The investigator is also free to terminate a subject's involvement in the study at any time if the subject's clinical condition warrants it.<br>The subject will be discontinued from study treatment (zolbetuximab/placebo and both components of CAPOX) if any of the following occur:<br>• Investigator determines it is in the subject's best interest to discontinue study treatment<br>• Subject develops radiological disease progression per RECIST 1.1 criteria based on assessment by IRC.<br>o If the investigator believes that the subject is continuing to derive clinical benefit (asymptomatic and/or without worsening of performance status or overall health) from study treatment, and an increase in tumor burden is not likely to affect vital organ function, the subject may remain on study treatment until an additional radiologic assessment is completed (≤ 9 weeks from previous radiologic assessment).<br>▪ If the additional radiologic assessment by IRC indicates PD per RECIST 1.1, then the subject must be discontinued from study treatment.<br>▪ If the additional radiologic assessment by IRC does not confirm the initial assessment of PD, the subject may continue to receive study treatment.<br>• Subject develops clinical progression per investigator assessment and radiologic assessment is not medically feasible to confirm radiologic progression due to the subject's condition.<br>• Subject starts another systemic chemotherapy, immunotherapy, radiotherapy or other treatment intended for antitumor activity.

- Subject starts another investigational agent or device.
- Subject develops unacceptable toxicity.
- Subject has a delay of zolbetuximab/placebo and both components of CAPOX for > 28 days from when the next zolbetuximab/placebo and both components of CAPOX treatment was scheduled to be begin (> 49 days from when the last dose of zolbetuximab/placebo and both components of CAPOX treatment began).
- Any clinical AE, laboratory abnormality, or inter-current illness, in the opinion of the investigator, indicates continued treatment is not in the best interest of the subject
- Female subject becomes pregnant.
- Significant deviation from the protocol or eligibility criteria as determined by the sponsor.
- Subject declines further treatment.
- Subject is lost to follow-up despite reasonable efforts by the investigator to locate the subject.
- Subject is noncompliant with the protocol based on investigator or Medical Monitor assessment.

Note, if a subject discontinues both components of CAPOX and zolbetuximab/placebo due to any reason other than IRC confirmed disease progression (and is not receiving any other anticancer therapy), the subject must be followed according to the protocol-specified radiologic assessment schedule until radiological disease progression per RECIST 1.1 criteria is confirmed by IRC assessment.

Study Discontinuation Criteria

All subjects should remain in the study through the Survival Follow-up Period (OS is a key secondary study endpoint). A subject will be discontinued from the Post-treatment, Long-term and Survival Follow-up Periods if any of the following occur:

- Subject specifically withdraws consent for any further contact with him/her or persons previously authorized by the participant to provide this information
- Subject is lost to follow-up despite reasonable efforts by the investigator to locate the subject
- Death (from any cause)
- Study termination by the sponsor

Endpoints for Evaluation:

Primary:

- PFS, defined as the time from the date of randomization until the date of radiological PD (by IRC per RECIST 1.1) or death from any cause, whichever is earliest

Secondary:

- OS, defined as the time from the date of randomization until the date of death from any cause
- Time to confirmed deterioration (TTCD) using the PF, OG25-Pain and GHS/QoL scores as measured by EORTC QLQ-C30 and QLQ-OG25 plus STO22 Belching subscale. TTCD is defined as time to first confirmed deterioration, i.e., time from randomization to first clinically meaningful deterioration that is confirmed at the next scheduled visit.
- ORR, defined as the proportion of subjects who have a best overall response of complete response (CR) or partial response (PR) as assessed by IRC per RECIST 1.1
- DOR, defined as the time from the date of the first response (CR/PR) until the date of PD as assessed by IRC per RECIST 1.1 or date of death from any cause, whichever is earliest
- Safety and tolerability, as measured by AEs, laboratory test results, vital signs, ECGs and ECOG performance status

- HRQoL, using the additional parameters as measured by EORTC QLQ-C30, QLQ-OG25 plus STO22 Belching subscale, GP, and EQ5D-5L questionnaires
- Pharmacokinetics of zolbetuximab, C_{trough}
- Immunogenicity of zolbetuximab as measured by the frequency of antidrug-antibody (ADA) positive subjects

Exploratory:

- TTP, defined as the time from the date of randomization until the date of PD as assessed by IRC per RECIST 1.1.
- PFS2, defined as the time from the date of randomization until the date of PD (per investigator) following subsequent anticancer therapy, death from any cause or start of any other anticancer therapy, whichever is earliest
- DCR, defined as the proportion of subjects who have a best overall response of CR, PR or SD as assessed by IRC per RECIST 1.1
- Potential genomic and/or other exploratory biomarkers that may be related to treatment outcome of zolbetuximab
- HRU

Statistical Methods:

Sample Size Justification:

One interim analysis and 1 final analysis are planned for OS, while only 1 final analysis is planned for PFS. The OS interim analysis will occur at the same time of final PFS analysis (after pre-specified number of PFS events) and final OS analysis will be performed after the pre-specified number of OS events are observed. The O'Brien-Fleming boundaries as implemented by Lan-DeMets (1983) alpha spending method (East®) will be used for the OS interim and OS final analyses. All statistical tests of treatment effects will be conducted at the 1-sided 0.025 level of significance unless otherwise specified.

Approximately 500 subjects will be randomized in a 1:1 ratio to receive zolbetuximab in combination with CAPOX chemotherapy (Arm A) or placebo in combination with CAPOX chemotherapy (Arm B).

The planned 300 PFS events during the study will provide 93.4% power to detect a difference in PFS between Arm A (zolbetuximab+CAPOX) with the assumption of 9 months median PFS time and Arm B (placebo+CAPOX) with the assumption of 6 months median PFS time (hazard ratio = 0.67) at the overall 1-sided 0.025 significance level. Similarly, the planned 386 OS events during the study will provide 80% power to detect a difference in OS between Arm A (zolbetuximab+CAPOX) with the assumption of 14.7 months median survival time and Arm B (placebo+CAPOX) with the assumption of 11 months median OS time (hazard ratio = 0.75) at the overall 1-sided 0.025 significance level.

Analysis Populations:

- The full analysis set (FAS) will include all subjects who are randomized to 1 of the treatment arms. Subjects will be analyzed according to the treatment arm to which they were randomized to. The FAS will be used for description of baseline characteristics and all efficacy analyses.
- The safety analysis set (SAF) will contain all subjects who received at least 1 dose of any study drug (zolbetuximab/placebo/CAPOX). The SAF will be used for all safety analyses. Subjects will be analyzed according to the actual treatment they received.
- The pharmacokinetic analysis set (PKAS) will consist of the subset of the SAF for which at least one zolbetuximab concentration measurement is available. Additional subjects may be

excluded from the PKAS at the discretion of the pharmacokineticist. The PKAS will be used for description of pharmacokinetic data.
**Efficacy Analyses:**<br>All efficacy analyses will be performed using FAS.
**Primary Efficacy Endpoint:**<br>The primary efficacy endpoint of PFS will be analyzed using the stratified Log Rank Test with stratification factors to be specified in the SAP.<br>The hypothesis testing on the primary analysis will be performed at an overall 1-sided 0.025 significance level to test the null hypothesis that PFS is not prolonged in Arm A compared to Arm B versus the alternative hypothesis that PFS is prolonged in Arm A compared to Arm B.<br>Estimates of the treatment effect will be expressed as Hazard Ratio using a stratified Cox model, including 95% Confidence Interval.<br>The sensitivity analysis for the primary efficacy endpoint will also be performed.<br>**Secondary Endpoints:**<br>The key secondary efficacy endpoint of OS will be analyzed using the stratified Log Rank Test with the same strata used in the analysis of PFS. To maintain the overall Type I error rate at the 0.025 significance level, the hypothesis testing on OS will be performed only if the null hypothesis on the primary analysis is rejected at the overall 1-sided 0.025 significance level. The key secondary TTCD endpoints of PF, OG25 Pain, and GHS/QoL will be analyzed using the same method as OS and PFS.<br>The secondary efficacy endpoint of ORR will be analyzed using the Cochran-Mantel-Haenszel test to control for the same strata used in the analysis of PFS and OS. The secondary efficacy endpoint of DOR will be analyzed similarly to PFS and OS.<br>The secondary HRQoL endpoints collected via the EORTC QLQ-C30 and QLQ-OG25 plus STO22 Belching subscale, GP and EQ-5D-5L will be analyzed with summary of change from baseline over time through the end of CAPOX treatment and inferential methods. Detailed analysis of HRQoL endpoints will be provided in the statistical analysis plan.
**Exploratory Endpoints:**<br>TTP and PFS2 will be analyzed in a similar way as PFS. However, in TTP analysis, deaths are not counted as events; rather, deaths are censored. DCR will be analyzed similarly as ORR. The HRU variables will be summarized by treatment arm.<br>**Safety Analyses:**<br>The safety evaluation will be based on AEs, clinical laboratory tests, vital signs, ECG and ECOG status. Descriptive statistics will be used to summarize safety data. All safety data will be summarized by treatment received (SAF).<br>All summaries of AEs will include only treatment-emergent events unless otherwise stated. AEs will be categorized by SOC and preferred term using MedDRA and will be graded for severity according to the NCI-CTCAE v4.03.<br>**Pharmacokinetics:**<br>Descriptive statistics will be used to summarize serum concentrations of zolbetuximab. The potential relationship between zolbetuximab immunogenicity and zolbetuximab pharmacokinetics, efficacy, and safety profile may be assessed. Additional model-based analyses may be performed and reported separately.

Biomarkers:

Biomarkers will be summarized graphically or descriptively, and summary statistics may be tabulated. Associations between biomarkers and clinical (e.g., efficacy, safety or pharmacodynamics, or pharmacokinetics) measures may be performed on subjects who have sufficient baseline and on-study treatment measurements to provide interpretable results for specific parameters.

Interim Analyses:

To evaluate whether zolbetuximab + CAPOX (Arm A) is beneficial compared to placebo + CAPOX (Arm B) while the study is ongoing, a formal OS interim analysis is planned when the final PFS analysis occurs with the pre-specified number of PFS events. A group sequential design using the O'Brien-Fleming type alpha-spending function [Lan & DeMets, 1983] for efficacy will be utilized to control the overall 1-sided 0.025 significance level (East®) for the OS analyses.

The IDMC may recommend terminating the study for favorable results at the formal efficacy interim analysis using OS. In the case of favorable results, the 1-sided significance level for superiority is 0.0074, assuming about 70% of the target number of OS events is obtained, for the interim OS analysis and 0.0228 for the final OS analysis. (Note: The OS significance level will be adjusted depending on the number of OS event at the time of interim analysis). If the 1-sided P value of the interim analysis is less than the significance level (and PFS is also significant at 1-sided 0.025 alpha), the IDMC may recommend terminating the study for success. If the study is not stopped after the interim analysis, a final OS analysis will occur after 100% of the planned death events have been observed.

Details for the interim analyses, monitoring subject safety, enrollment rates and event (PFS/death) rates will be contained in the IDMC Charter. Recommendations regarding study conduct will be made by the IDMC based on their assessment of these rates.

V. FLOW CHART, STUDY SCHEMATICS AND SCHEDULE OF ASSESSMENTS

a. Flow Chart

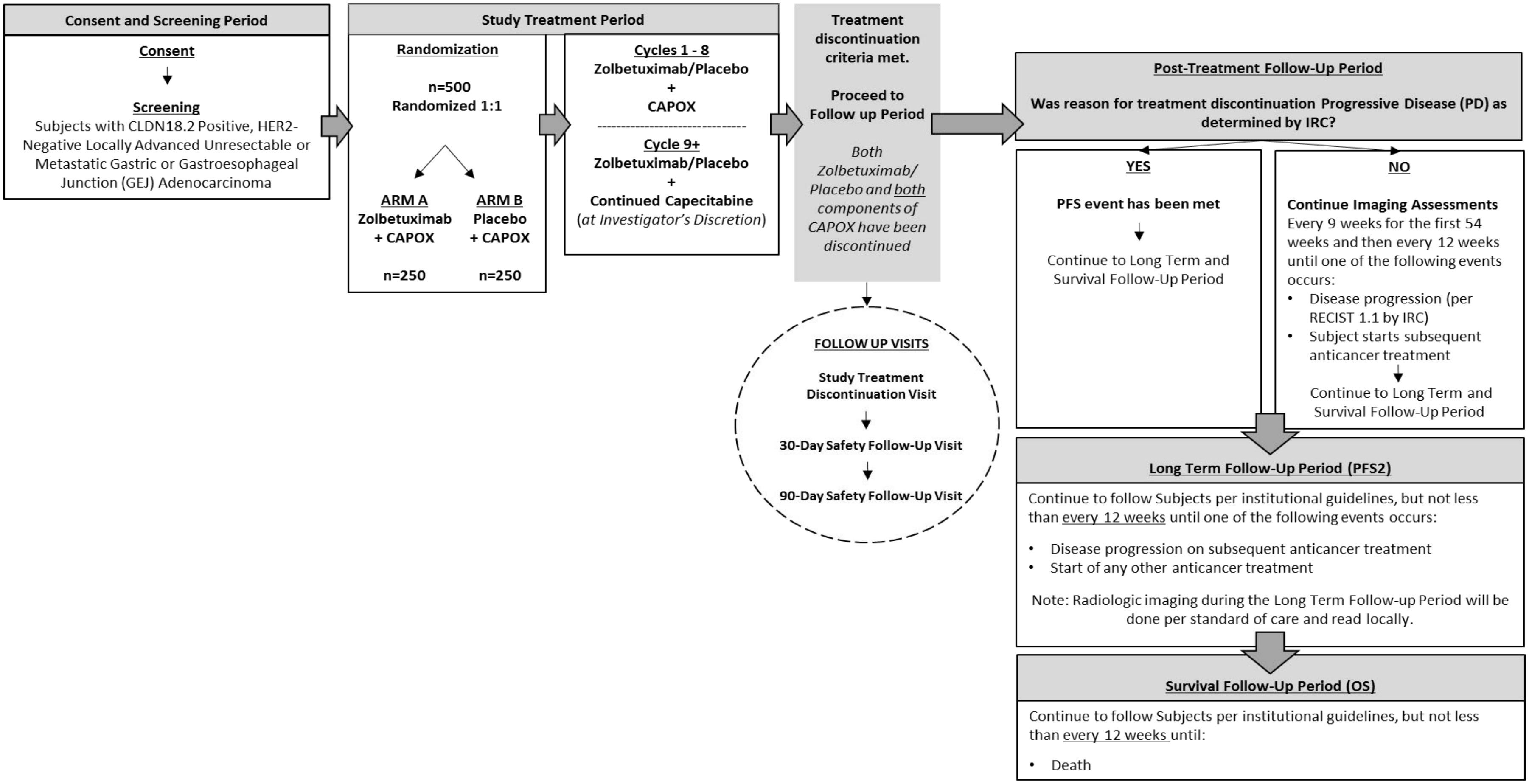

CLDN: claudin; HER2: human epidermal growth factor receptor 2; IRC: independent review committee; OS: overall survival; PFS: progression free survival; PFS2: progression free survival following subsequent anticancer treatment; RECIST: Response Evaluation Criteria In Solid Tumors

b. Study Treatment Period Dosing Schedule (1 Cycle = approximately 21 Days)

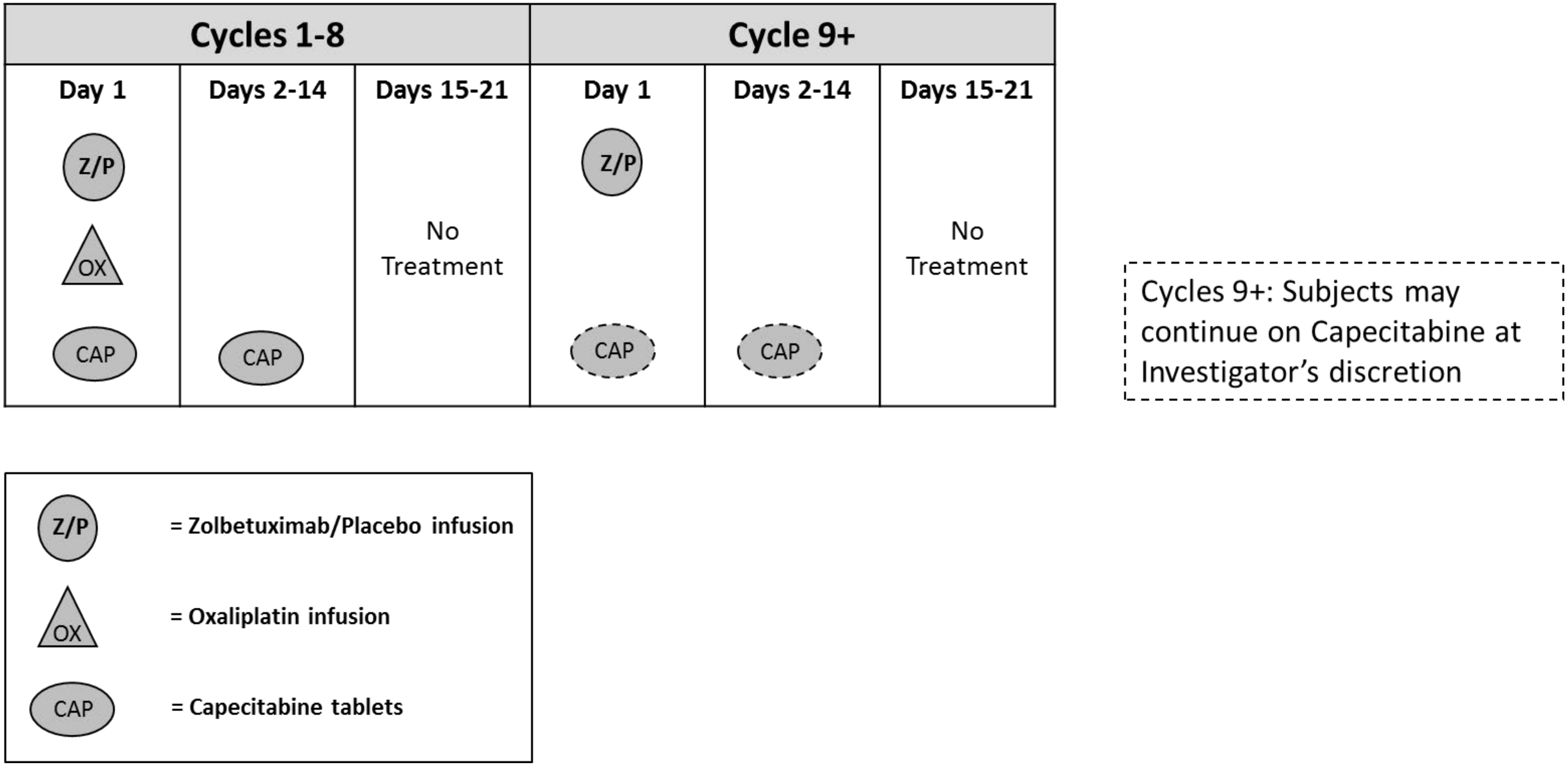

Dosing Schematics:

a. Combination Zolbetuximab/Placebo and CAPOX Dosing (Cycle 1 to Cycle 8)

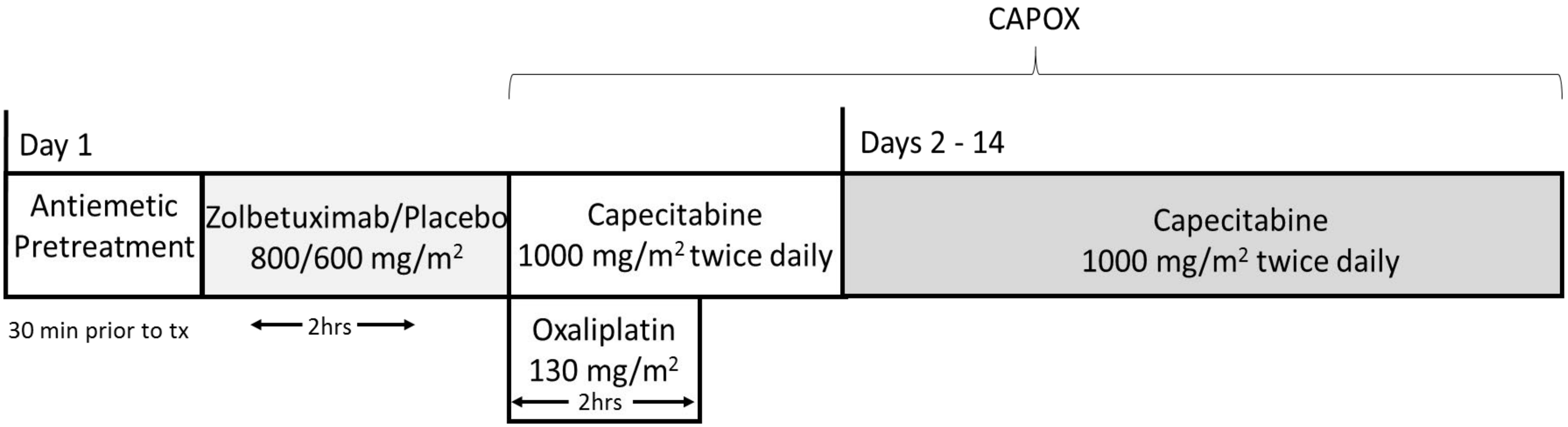

Note: Standard timeframes described above may be modified as per Section 5.1.2, Study Treatment Dose Modifications, Delays and Interruptions

tx: treatment.

b. Zolbetuximab/Placebo Only Dosing (Cycle 9 and onwards)*

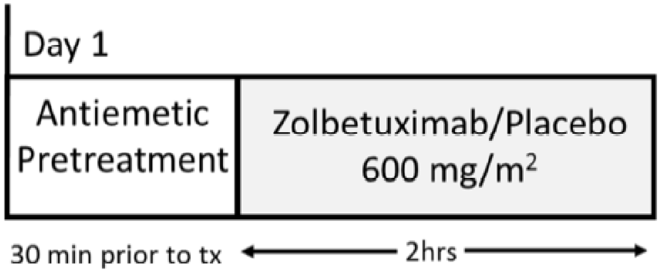

Note: Standard timeframes described above may be modified as per Section 5.1.2, Study Treatment Dose Modifications, Delays and Interruptions
*Cycles 9+: Subjects may continue on Capecitabine at Investigator's discretion

Table 1 Schedule of Assessments

VISIT†	Screening[1]	Study Treatment Period (Each Cycle = approximately 21 Days)						Follow-up Period				
		**Cycles 1-8** Zolbetuximab/Placebo + CAPOX			**Cycle 9+** Zolbetuximab/Placebo + Capecitabine (at investigator discretion)			Study Treatment Discontinuation Visit[18]	30-Day Safety Follow-up Visit(s)[19]	90-Day Safety Follow-up Visit(s)[20]	Post-treatment Follow-up Period [21]	Long-term and Survival Follow-up Periods [22]
**Day**		1	2-14	15-21	1	2-14	15-21					
**Visit Window (calendar days)**	-45 to -1	+7*	(no visit)	(no visit)	+7	(no visit)	(no visit)	+7	±7	±7	±7	±14
Informed Consent	X											
CLDN18.2 Tumor Sample[2]	X											
HER2 Tumor Sample[2]	X											
Biopsy (if applicable)[2]	X											
Medical and Disease History	X											
Confirmation of Inclusion/Exclusion Criteria[3]	X											
Randomization[4]		X										
**Treatments**												
Antiemetic Pretreatment[5]		X			X							
Zolbetuximab/Placebo[6]		X			X							
Post-infusion Observation Period[7]		X			X							
Oxaliplatin (CAPOX[8])		X										
Capecitabine (CAPOX[8])		X	X		X	X						
**Safety Assessments**												
Physical Examination[9]	X	X			X			X	X			
Weight[9]	X	X			X			X	X			
Vital Signs[10]	X	X			X			X	X			
ECOG Performance Status[9]	X	X			X			X				
12-lead ECG[11]	X	X	If clinically indicated and/or per local requirements					X	X			
**Image Assessment[12]**	X[1]	Every 9 weeks ±7 days from C1D1 for the first 54 weeks and then every 12 weeks ±7 days thereafter										
Table continued on next page												

VISIT†	Screening[1]	Study Treatment Period (Each Cycle = approximately 21 Days)						Follow-up Period				
		**Cycles 1-8** Zolbetuximab/Placebo + CAPOX			**Cycle 9+** Zolbetuximab/Placebo + Capecitabine (at investigator discretion)			Study Treatment Discontinuation Visit[18]	30-Day Safety Follow-up Visit(s)[19]	90-Day Safety Follow-up Visit(s)[20]	Post-treatment Follow-up Period [21]	Long-term and Survival Follow-up Periods [22]
**Day**		1	2-14	15-21	1	2-14	15-21					
**Visit Window (calendar days)**	-45 to -1	+7*	(no visit)	(no visit)	+7	(no visit)	(no visit)	+7	±7	±7	±7	±14
**Subject Contact**												X
**Laboratory Tests**												
Biochemistry[13]	X	X			X			X	X			
TSH and Free T4[13]	X	If clinically indicated						X				
Hematology[13]	X	X			X			X	X			
Coagulation Parameters (PT, PTT and INR)[14]	X	If clinically indicated										
Urinalysis[13]	X	X			X			X	X			
Serum Pregnancy Test[15]	X	If clinically indicated and/or per local requirements										
Urine Pregnancy Test[16]		X			X			X	X			
DPD testing per local requirements	X											
Cytokine/Chemokine and/or Tryptase		If clinically indicated										
**Electronic Clinical Outcomes Assessments (eCOA)**												
HRQoL[17]	X	X			X			X	X	X		
Health Resource Utilization (HRU)[17]		X			X			X	X	X		
**Sampling**												
Pharmacokinetics of Zolbetuximab (Serum)[23]		X			X				X	X		
Antidrug-Antibodies (ADA) for Immunogenicity[24]		X			X				X	X		
Genetic Immune Polymorphisms (Whole Blood)[25]		X										
Exploratory Biomarkers (Serum)[26]		X						X				
Exploratory Biomarkers (Plasma)[26]		X						X				
Whole Blood Sample for PGx (optional)[27]		X										
Post-progression Tumor Sample (optional)[28]								X				
**Concomitant Medication[29]**	X	X			X			X	X	X		
**AE/SAE[30]**	X	X			X			X	X	X		

Footnotes appear on next page

ADA: antidrug antibody; AE: adverse event; βhCG: beta human chorionic gonadotropin; C1D1: Cycle 1 Day 1; CAPOX: capecitabine and oxaliplatin; CLDN: claudin; CT: computerized tomography; DPD: dihydropyrimidine dehydrogenase; ECG: electrocardiogram; eCOA: electronic Clinical Outcomes Assessment; ECOG: Eastern Cooperative Oncology Group; eCRF: electronic case report form; FFPE: formalin fixed paraffin embedded; HER2: human epidermal growth factor receptor 2; HRQoL: health-related quality of life; HRU: Health Resource Utilization; ICF: informed consent form: INR: international normalized ratio; IRC: independent review committee; IRR: infusion-related reaction; IRT: interactive response technology; IV: intravenous; MRI: magnetic resonance imaging; OS: overall survival; PD: progressive disease; PFS: progression free survival; PFS2: progression free survival following subsequent anticancer treatment; PGx: pharmacogenomics; PT: prothrombin time; PTT: partial thromboplastin time; RECIST: Response Evaluation Criteria In Solid Tumors; SAE: serious adverse event; T4: thyroxine; TSH: thyroid stimulating hormone

†Local and/or regional protocols or precautions for COVID-19 management should be followed as applicable.

*+7 calendar day visit window does not apply to C1D1.

1. Screening: The Screening period is 45 days from full main ICF signature. Retesting of lab values is allowed within the 45-day Screening period. Re-screening outside the 45-day window under a new subject number may be allowed once and upon discussion with the Medical Monitor. CT scans and MRIs conducted as part of a subject's routine clinical management (i.e., standard of care) obtained before signing the ICF may be utilized for screening or baseline purposes, provided the procedures met the protocol-specified criteria and were performed within the Screening period.
    - Optional partial screening: A partial screening ICF may be available for central testing of tissue for CLDN18.2 and HER2 only.
    - Laboratory testing:
        - Eligibility can be determined based on central and/or local laboratory testing; **however**:
            - The most recent laboratory tests with available results must be used to confirm the subject's eligibility.
            - Central labs must be collected and submitted to the central laboratory during the Screening period.
            - If retesting of lab values is necessary to confirm eligibility, local labs can be used without requiring additional sample collection for central laboratory submission.
        - The screening labs used to determine eligibility should be collected within 14 days prior to randomization.
    - Radiologic imaging used to confirm eligibility must be conducted within 28 days prior to randomization.
2. CLDN18.2 and HER2 Testing: FFPE tumor tissue will be collected for central testing to determine CLDN18.2 and HER2 status. Archival tumor tissue from the primary tumor (gastric or GEJ) is preferred. If primary tumor tissue is not available, tumor tissue from a metastatic site (excluding bone metastasis) may be used. A minimum of 1 FFPE tumor tissue block (preferred) OR a minimum of 15 FFPE unstained slides are required as allowed per local policy. If slides are submitted, the slides should be freshly cut from the FFPE block within the time frame described in the laboratory manual. If local HER2 results are already available from local testing, a minimum of 12 FFPE unstained slides are required to be submitted to the central lab as allowed per local policy. If the specimen is insufficient or unavailable, a biopsy may be performed to obtain primary tumor tissue (preferred) or tumor tissue from metastatic site (excluding bone metastasis). Sponsor pre-approval is required when the sole purpose of the biopsy procedure is to assess eligibility for this study. If the required number of slides cannot be provided, the sponsor or designee should be contacted for further guidance. See [Section 5.7.3 Tumor Tissue Samples].
3. Confirmation of Inclusion/Exclusion Criteria must be completed prior to randomization.
4. Randomization: After confirmation of eligibility, the blinded site user will perform the randomization IRT transaction. The unblinded pharmacist/designee will be notified by the IRT system about the randomly assigned treatment. Randomization may be performed prior to C1D1. If C1D1 cannot be performed within 5 calendar days from Randomization, please contact the Medical Monitor for discussion. Details of infusion preparation and storage requirements are defined in the Pharmacy Manual and Infusion Guidelines.

Footnotes continued on next page

5. Antiemetic Pretreatment: Antiemetic premedication (prophylactic antiemetics) should be administered prior to each study treatment. IV antiemetic premedication should be initiated prior to treatment, or oral antiemetic premedication should be initiated at a minimum of 30 minutes prior to treatment. Antiemetic premedication should be given according to institutional standard of care, published guidelines and the respective product package insert(s). For further details, see [Section 5.1.1.2 Antiemetics]. .
6. Zolbetuximab/placebo will be administered as a minimum 2-hour intravenous infusion every 3 weeks starting on C1D1. Please refer to Pharmacy Manual and Infusion Guidelines for more detailed information. Zolbetuximab/placebo should be administered prior to CAPOX. For further details, see [Section 5.1.1.1 Zolbetuximab/Placebo].
7. Post-infusion Observation Period: Following the first dose of zolbetuximab/placebo on C1D1, the subject must be observed for 2 hours post zolbetuximab/placebo infusion. The post-infusion observation period can be conducted during the CAPOX administration. If any ≥ grade 2 AEs are observed during infusion or during the post-infusion observation period, subsequent zolbetuximab/placebo infusion times should be extended and subjects should continue to be observed for 2 hours post zolbetuximab/placebo infusion. If the subject does not develop any grade ≥ 2 AEs, the subject should be observed for 1 hour post-infusion for their subsequent zolbetuximab/placebo infusions. The subject should be instructed to notify site personnel if they develop any AEs during this observation time period. In the event of an IRR with features of anaphylaxis (regardless of grade) or grade 3 or 4 IRR, blood samples for cytokine/chemokine panel and serum total tryptase level (levels typically peak within 3 hours after the onset of symptoms) should be collected once the subject has stabilized, for shipment to the central laboratory. See Observation Period following zolbetuximab/placebo infusion [Section 5.4.2] for further details.
8. CAPOX is a combination of oxaliplatin intravenous infusion and capecitabine tablets and will be administered starting at C1D1 for up to 8 treatments. See [Section 5.1.1.3].
9. Physical Exam: should include height (at Screening only), weight and ECOG performance status. A full physical exam is required at Screening. The physical exam only needs to be repeated on C1D1 if clinically significant changes from screening are observed (in the opinion of the investigator). For all cycles, the physical examination, weight and ECOG performance status assessments can be completed up to 48 hours prior to zolbetuximab/placebo administration. Targeted (symptom driven) physical exams should be conducted every 3 weeks on day 1 of each cycle. For further details, see [Section 5.4.4 Physical Examination]
10. Vital signs (pulse, blood pressure, temperature) should be taken during every visit at the following time points (see [Section 5.4.1 Vital Signs]):
    - Predose at every visit
    - C1D1: Every 30 (±10) minutes during zolbetuximab/placebo infusion
      - If the subject did not develop any ≥ grade 2 AEs during the C1D1 zolbetuximab/placebo infusion or Post-infusion Observation Period, the site may do the following for subsequent zolbetuximab/placebo infusions:
        - The post-infusion observation period can be 1 hour for subsequent visits after C1D1
        - The vital signs can be assessed every 60 minutes for subsequent visits after C1D1
    - Every 60 (±10) minutes post zolbetuximab/placebo infusion during the Post-infusion Observation Period (for 1 or 2 hours. See footnote 7)
    - Unscheduled if clinically indicated
11. ECGs: ECGs will be locally assessed. When collected on the same day, ECG should be collected prior to pharmacokinetic samples. For further details, see [Section 5.4.5 Electrocardiogram]. A single ECG will be performed at the following time points:

Footnotes continued on next page

- Screening
- Up to 48 hours prior to every oxaliplatin infusion (before any antiemetic treatment administration)
- Up to 6 hours following completion of every oxaliplatin infusion
- Zolbetuximab/placebo Discontinuation Visit
- Zolbetuximab/placebo 30-day Follow-up Visit
- If clinically indicated or per local requirements

12. Imaging Assessments: Radiologic imaging will be evaluated at Screening (must be conducted within 28 days prior to randomization) and every 9 weeks (± 7 days) counting from C1D1 for the first 54 weeks and then every 12 weeks (± 7 days) thereafter until subject develops radiological disease progression per RECIST 1.1 by IRC or starts other systemic anticancer treatment, whichever comes earlier. Imaging schedule should be maintained regardless of treatment delay. Imaging will include CT scans with contrast of the thorax, abdomen, and pelvis. If CT scan with contrast is medically not feasible, MRI may be used for imaging. Bone scans (or focal X-ray) or brain imaging should be performed if metastatic disease in bone or brain is suspected, respectively. The same mode of imaging should be utilized throughout the study unless medical necessity requires a change. For randomized subjects, screening imaging should be sent to the central imaging vendor no later than at the time of submission of the first on-treatment imaging. All imaging acquired post randomization will be sent to the central imaging vendor within 7 days of scanning for the blinded independent central assessment of radiological tumor response based on RECIST 1.1. The investigator should make every effort to immediately submit radiologic assessments for IRC review when PD is suspected. See [Section 5.3 Efficacy Assessments]. Refer to Imaging Acquisition Guidelines for further detail on scan modality and contrast options.
13. Laboratory Assessments: See [Section 5.4.3 Laboratory Assessments] for list of laboratory assessments. Laboratory tests must be sent to the central laboratory for analysis. For screening/eligibility laboratory assessments, see footnote number 1.
    - Laboratory test results (central or local) will be reviewed by the investigator prior to any study treatment. Clinical significance of out-of-range laboratory findings is to be determined and documented by the investigator/sub-investigator who is a qualified physician.
    - Local laboratory results may be used for treatment decisions; however, central laboratory samples must also be drawn per protocol and sent to the central laboratory.
    - Central and local labs may be collected up to 48 hours prior to study treatment.
    - Holidays and weekends should be taken into account when scheduling these sample collections.
    - Additional assessments may be done centrally or locally to monitor AEs or as clinically indicated.
14. Coagulation (PT, PTT and INR): Coagulation tests should be done at Screening and during study treatment period if clinically indicated. Local or central lab results may be used to confirm eligibility. Ongoing evaluation should be continued for subjects who are receiving therapeutic anticoagulation according to local standard of care. See [Section 5.4.3 Laboratory Assessments].
15. Serum Pregnancy Test: Serum pregnancy tests will be collected for female subjects of childbearing potential only. Serum pregnancy tests collected at Screening, during study treatment period and if clinically indicated or per local requirements. Serum pregnancy test can be completed up to 48 hours prior to zolbetuximab/placebo administration. (Note: For Screening, subjects with elevated serum βHCG and a demonstrated non-pregnant status through additional testing are eligible.) Local or central laboratory results may be used to confirm eligibility.
16. Urine Pregnancy Test: Urine pregnancy tests will be collected for female subjects of childbearing potential only. Local urine pregnancy tests to be performed during the treatment period every 3 weeks on day 1 of each cycle and at the zolbetuximab/placebo Study Treatment Discontinuation and 30-day Safety Follow-up Visits. Urine pregnancy test can be completed up to 48 hours prior to zolbetuximab/placebo administration. Additional urine pregnancy testing for up to 9 months after the final study treatment administration may be conducted based on local requirements.

Footnotes continued on next page

17. HRQoL and HRU questionnaires: eCOA questionnaires should be completed by the subject at Screening (except for HRU), on day 1 of each cycle (or up to 48 hours) before any antiemetic or drug treatment and before the disease status is discussed with the subject, using the electronic tablet device provided. When completion by the subject is not possible, the questionnaires may be administered to the subject by site personnel using the electronic tablet device. For subjects with low literacy, situations where translations are unavailable or other circumstances preventing the screening questionnaires to be completed, please contact the sponsor for further guidance.
18. Study Treatment Discontinuation Visit (End of Study Treatment): The Study Treatment Discontinuation Visit will take place ≤ 7 days following the decision to discontinue study treatment (zolbetuximab/placebo and CAPOX [both components]). If zolbetuximab/placebo and CAPOX (both components) are discontinued on a different day, subjects will have separate Study Treatment Discontinuation Visits following each treatment's discontinuation. Laboratory tests must be sent to the central laboratory for analysis. HRQoL and HRU questionnaires are not required at CAPOX treatment discontinuation visit. A combined visit can be completed if zolbetuximab/placebo are discontinued on the same day.
19. 30-day Safety Follow-up Visit: A 30-day Safety Follow-up Visit should occur 30 days after the last dose of zolbetuximab/placebo and will include the assessments as shown in the Schedule of Assessments above. A 30-day Safety Follow-up Visit should occur 30 days after the last dose of CAPOX (both components) and may be conducted by phone if the subject is unable to visit the site and will require contact for AE/SAE collection only. HRQoL and HRU questionnaires are not required at CAPOX 30-day safety follow up visit. A combined visit can be completed if zolbetuximab/placebo and both components of CAPOX are discontinued on the same day and HRQoL and HRU questionnaires should be completed for a combined visit.
20. 90-day Safety Follow-up Visits: A 90-day Safety Follow-up Visit should occur 90 days after the last dose of zolbetuximab/placebo and will include the assessments as shown in the Schedule of Assessments above. A 90-day Safety Follow-up Visit should occur 90 days after the last dose of CAPOX (both components) and may be conducted by phone if the subject is unable to visit the site and will require contact for AE/SAE collection only. HRQoL and HRU questionnaires are not required at CAPOX 90-day safety follow up visit. A combined visit can be completed if zolbetuximab/placebo and both components of CAPOX are discontinued on the same day and HRQoL and HRU questionnaires should be completed for a combined visit.
21. Post-treatment Follow-up: if a subject discontinues all study treatments (zolbetuximab/placebo and both components of CAPOX) prior to IRC-confirmed radiological disease progression, the subject will enter the Post-treatment Follow-up Period and continue to undergo imaging assessments every 9 weeks (±7 days) (or every 12 weeks [±7 days] if subject has been on study over 54 weeks) until radiologic disease progression (i.e., PFS) or the subject starts subsequent anticancer treatment, whichever occurs earlier. If study treatments (zolbetuximab/placebo and both components of CAPOX) are discontinued due to PD, the subject will enter the Long-term and Survival Follow-up Period.
22. Long-term and Survival Follow-up Period: Following disease progression on first-line treatment or start of subsequent anticancer treatment, subjects will be followed in the Long-term and Survival Follow-up Period per institutional guidelines, but at least every 12 weeks. Radiologic imaging will be done per standard of care and read locally until PFS2 is documented. Survival Follow-up Period will continue until death (from any cause). All post-progression details including subsequent anticancer treatment and date and site of progression will be recorded on the eCRF. Subject contact by phone or other remote methods is sufficient during Long-term and Survival Follow-up.
23. Pharmacokinetics: Serum samples for zolbetuximab/placebo will be taken at the below time points and sent to the central laboratory. The date and time of each blood sample collection will be recorded to the nearest minute.
    - Cycle 1 Day 1: End of zolbetuximab/placebo infusion
    - Cycle 2 Day 1: Predose
    - Cycle 3 Day 1: End of zolbetuximab/placebo infusion
    - Predose on Day 1 of Cycles 5, 9, 13 and 17
    - Zolbetuximab/placebo 30-Day Safety Follow-up visit
    - Zolbetuximab/placebo 90-Day Safety Follow-up visit
    - Unscheduled pharmacokinetic blood samples may be taken at any time during the study to evaluate drug exposure following a safety event

Footnotes continued on next page

Pharmacokinetic Sampling Window:
- Predose: within 60 minutes prior to dosing
- End of Infusion: within 10 minutes after the end of the infusion

24. ADA: Blood samples (Serum) for ADA will be taken at the below time points and sent to the central laboratory.
    - Cycle 1 Day 1: Predose
    - Cycle 2 Day 1: Predose
    - Predose on Day 1 of Cycles 5, 9, 13 and 17
    - Zolbetuximab/placebo 30-Day Safety Follow-up visit
    - Zolbetuximab/placebo 90-Day Safety Follow-up visit

    ADA Sampling Window: Predose: within 60 minutes prior to dosing
25. Genetic Immune Polymorphism: To be collected per local policy. Whole blood sample taken at C1D1 will be sent to the central laboratory. Samples may be collected up to 48 hours prior to study treatment.
26. Exploratory Biomarker (Serum and Plasma): To be collected per local policy. Samples should be taken at the below time points and sent to the central laboratory:
    - Cycle 1 Day 1: Predose
    - Cycle 2 Day 1: Predose
    - Cycle 3 Day 1: Predose
    - Cycle 4 Day 1: Predose
    - Cycle 5 Day 1: Predose
    - Cycle 6 Day 1: Predose
    - Cycle 8 Day 1: Predose
    - Zolbetuximab/placebo Study Treatment Discontinuation Visit

    Exploratory Biomarker samples may be collected up to 48 hours prior to study treatment
27. Optional PGx: For subjects who signed a separate ICF, an optional whole blood sample for PGx for exploratory biomarker analysis may be collected up to 48 hours prior to first study drug administration. Sample collection is optional and only collected as allowed per local policy.
28. Optional Post-Progression Tumor Sample: For subjects who signed a separate ICF, an optional post-progression tumor sample for exploratory biomarker analysis may be collected following IRC confirmation of disease progression and prior to initiation of subsequent anticancer therapy. Sample collection is optional and only collected as allowed per local policy.
29. Concomitant medications will be collected from the time of full main informed consent through 90 days following the last dose of study treatment.
30. AEs/SAEs: AEs and SAEs (regardless of causality) will be collected from the time of full main informed consent through 90 days following the last dose of study treatment. See [Section 5.5.5 Reporting of Serious Adverse Events].

1 INTRODUCTION

Gastric and gastroesophageal junction (GEJ) cancers are among the malignancies with the highest unmet medical need. Gastric cancer-related mortality is the fourth leading cause of cancer death worldwide, even if its incidence has decreased over the last 50 years in different regions of the world [Cancer Fact Sheet, 2018; Amiri et al, 2011]. On the other hand, the incidence of subjects with GEJ adenocarcinoma has increased in recent decades, coinciding with a shift in histological type and primary tumor location [Waddell et al, 2013; Sahin et al, 2008].

In 2017, an estimated 723100 people died worldwide from gastric cancer [Lederman, 2017]. The overall 5-year survival rate for gastric and GEJ cancers is averaging 20% in the US and Europe, despite aggressive standard treatments, which are also associated with substantial side effects [Pennathur et al, 2013; Sahin et al, 2008].

There is no single standard, globally accepted first-line reference chemotherapeutic regimen for advanced gastric cancer. In the US and Europe, the current standard of care cytotoxic chemotherapy regimen consists of fluoropyrimidine with platinum-based combination chemotherapy regimens with or without a third agent such as docetaxel or epirubicin [National Comprehensive Cancer Network (NCCN), 2017; Waddell et al, 2013; Pasini et al, 2011]. Subjects in this study will receive CAPOX (a combination of capecitabine and oxaliplatin), which is a globally accepted standard-of-care treatment for subjects with locally advanced unresectable or metastatic gastric or gastroesophageal (GE) cancer. The safety of this combination regimen is well-documented [Kim et al, 2012].

The lack of a major benefit from the various newer generation combination chemotherapy regimens for these cancers has stimulated research into the use of targeted agents such as monoclonal antibodies (mAbs). Two mAbs, trastuzumab and ramucirumab, have received approval for treatment of gastric cancer. Trastuzumab selectively binds the extracellular domain of human epidermal growth factor receptor 2 (HER2), which is overexpressed in approximately 20% to 30% of gastric tumors [Bang et al, 2009], and ramucirumab specifically binds vascular endothelial growth factor (VEGF) receptor 2 and blocks binding of VEGF receptor ligands VEGF-A, VEGF-C and VEGF-D. Trastuzumab is approved for treatment of HER2 overexpressing metastatic gastric or GEJ adenocarcinoma while ramucirumab is approved as a single agent or in combination with paclitaxel, for treatment of advanced gastric or GEJ adenocarcinoma, with disease progression on or after prior fluoropyrimidine-or platinum-containing chemotherapy [CYRAMZA Prescribing Information, 2017; HERCEPTIN Prescribing Information, 2016]. These agents prolonged median overall survival (OS) by 4 or fewer months when given alone or in combination with chemotherapy compared with standard of care cytotoxic chemotherapy [Fuchs et al, 2014; Wilke et al, 2014; Ohtsu et al, 2011; Bang et al, 2010].

Approximately 70% to 80% of patients with metastatic or advanced unresectable gastric and GEJ adenocarcinoma in the first line setting have tumors that are HER2 negative and are not treatable with trastuzumab. These patients have an expected median survival of approximately 1 year [Shah et al, 2017]. Therefore, a significant unmet medical need exists

for the first-line treatment of patients with HER2 negative locally advanced or metastatic unresectable gastric and GEJ cancers. Zolbetuximab is being developed with the goal of addressing this unmet medical need.

1.1 Background

Zolbetuximab is a genetically engineered, highly purified chimeric (mouse/human IgG1) antibody directed against the tight junction molecule Claudin 18.2 (CLDN18.2). The target is a member of the claudin family of more than 20 structurally related proteins that are involved in the formation of tight junctions in epithelia and endothelia [Niimi et al, 2001]. Tight junctions, together with adherens junctions and desmosomes, form the apical junctional complex in epithelial and endothelial cellular sheets. Adherens junctions and desmosomes are responsible for the mechanical adhesion between adjacent cells, whereas tight junctions are essential for the tight sealing of the cellular sheets forming a luminary barrier and controlling the paracellular ion flux.

CLDN18.2 is a 27.8 kDa protein with 4 membrane-spanning domains and 2 small extracellular loops [Gunzel & Yu, 2013; Sahin et al, 2008]. Zolbetuximab recognizes the first extracellular domain of CLDN18.2 with high affinity and specificity. Zolbetuximab does not bind to any other claudin family member including the closely related splice variant 1 of Claudin 18 (CLDN18.1).

CLDN18.2 is a highly cell type specific differentiation antigen that is expressed by differentiated gastric mucosa cells in the pit and base regions of gastric glands. Moreover, CLDN18.2 is not detectable in any other normal cell type of the human body either at transcript level or as protein. This highly selective tissue distribution pattern results in CLDN18.2 expression being strictly confined to a subpopulation of gastric epithelial cells in normal tissue [Sahin et al, 2008].

CLDN18.2 is expressed in a diversity of human cancers and is the dominant isoform in GE and pancreatic cancer [Lee et al, 2011]. The expression of CLDN18.2 is retained upon malignant transformation of gastric epithelia and is present in 81% of primary gastric adenocarcinomas. CLDN18.2 expression is frequently detected in diffuse and in intestinal gastric cancers. The CLDN18.2 protein is also localized in lymph node metastases of gastric cancer adenocarcinomas and in distant metastases into the bile duct, lung and especially into the ovary (so-called Krukenberg tumors). Furthermore, over 42% of esophageal adenocarcinomas and 50% to 70% of pancreatic cancers display significant expression of CLDN18.2 [Woll et al, 2014; Lee et al, 2011; Sanada et al, 2010; Karanjawala et al, 2008; Sahin et al, 2008].

Zolbetuximab is being developed for the first-line treatment of adult subjects with locally advanced unresectable or metastatic CLDN18.2-positive, HER2-negative gastric or GEJ adenocarcinoma in combination with platinum- and fluoropyrimidine-based chemotherapy. For this study, a subject’s tumor must express CLDN18.2 in ≥ 75% of tumor cells demonstrating moderate to strong membranous staining as determined by central immunohistochemistry (IHC) testing.

1.2 Nonclinical and Clinical Data

1.2.1 Nonclinical Data

In vitro studies with CLDN18.2-positive and negative cancer cell lines showed that zolbetuximab binds to the extracellular domain 1 of CLDN18.2 on human gastric cancer cell lines with high relative affinity and selectivity. In vitro assays demonstrated that zolbetuximab mediated an efficient lysis of CLDN18.2-positive cells through antibody-dependent cell-mediated cytotoxicity (ADCC) and complement-dependent cytotoxicity (CDC).

All zolbetuximab-mediated effects are strictly CLDN18.2 antigen-specific.

In bio-distribution studies in nude mice with human tumor xenografts, intravenously administered zolbetuximab was retained, as well as specifically and strongly enriched in CLDN18.2-positive human xenografts. No or little in vivo binding of zolbetuximab to any other mouse tissues including stomach tissue was observed.

Administration of repeated doses of zolbetuximab to mice bearing CLDN18.2-positive tumors resulted in retardation of tumor growth kinetics in tumor models.

A series of experiments were conducted with CLDN18.2-expressing cell lines derived from NUGC-4 and KATO-III to investigate the effects of combining these chemotherapy agents with zolbetuximab. Combinations of chemotherapy agents used in the treatment of gastric and esophageal cancers, including 5-FU, oxaliplatin and epirubicin (e.g., 5-FU/oxaliplatin, 5-FU/oxaliplatin/epirubicin) augmented zolbetuximab activity. In vitro pre-sensitization of human gastric cancer cells with chemotherapy resulted in an increase in the amount of cell surface CLDN18.2 and thus improved zolbetuximab-mediated ADCC and CDC.

In immunocompetent mice, zolbetuximab in combination with chemotherapy resulted in a pronounced T cell infiltration into the tumors and significant long-term survival benefit over zolbetuximab alone. Most likely this effect was mediated by induction of adaptive T cell immunity, which may have led to a prolonged antitumor effect and immune surveillance.

CLDN18.2 is highly conserved across species, and the epitope of zolbetuximab is identical between humans, mice and cynomolgus monkeys. In addition, the binding affinity of zolbetuximab to CLDN18.2 orthologs from mice, humans and cynomolgus monkeys was shown to be comparable, providing sufficient evidence that testing in mice and monkeys covers the potential on-target effects and toxicities of zolbetuximab.

The nonclinical pharmacology studies conducted with zolbetuximab provide sufficient experimental evidence that zolbetuximab depletes CLDN18.2-positive cells via ADCC and CDC. Cytotoxic drugs were shown to increase CLDN18.2 expression on human cancer cells and to improve the activity of the major mechanism of action (ADCC and CDC). Hence, the combination of zolbetuximab with first-line chemotherapy is being investigated in the clinic.

Safety pharmacology and toxicity of zolbetuximab were assessed in mice and cynomolgus monkeys. In mice, the maximum exposure tested was 300 mg/kg weekly over 13 weeks, and in cynomolgus monkeys, the maximum exposure tested was 100 mg/kg weekly over 4 weeks.

In both species, no target organs of toxicity were identified; however, in monkeys, emesis was observed in a non-dose-related manner. The emesis that was observed in monkeys was not severe and spontaneously resolved despite continued dosing. The emetic potential of zolbetuximab was confirmed in an investigational study in ferrets. This effect is considered to be related to the binding of zolbetuximab to junctional protein, CLDN18.2, in the gastric epithelium. A human tissue cross-reactivity study of zolbetuximab showed that the gastric mucosa was the only tissue with strong membrane staining. However, histological assessment of the gastric tissue in monkeys failed to identify any histopathological lesions.

Besides the findings listed previously, no other zolbetuximab-related adverse effects were observed in any organ, neither clinically, nor macroscopically or histologically upon postmortem analysis.

In summary, the nonclinical data outlined above supports the clinical development of zolbetuximab in combination with standard chemotherapy for the treatment of CLDN18.2-positive gastric or GEJ adenocarcinoma.

Please refer to the current IB for most recent nonclinical data.

1.2.2 Clinical Data

Zolbetuximab has been evaluated in clinical studies as a single agent and in combination with epirubicin, oxaliplatin and capecitabine (EOX) chemotherapy or in combination with immunomodulation therapy (zoledronic acid [ZA] with or without interleukin-2 [IL-2]) for the treatment of adult subjects with CLDN18.2-positive advanced adenocarcinoma of the stomach, esophagus or GEJ.

CLDN18.2 expression was immunohistochemically determined for all subjects enrolled in the clinical studies. Several studies had an enrichment type of design meaning that only subjects above a certain threshold of CLDN18.2 positivity in their tumors were eligible for treatment. Eligibility for enrollment in the GM-IMAB-001-03 (EudraCT No. 2011-005285-38) and GM-IMAB-001-04 (EudraCT No. 2011-005509-64) studies, hereafter referred to as FAST and PILOT, respectively, was determined using the analytically validated and Conformité Européene (CE)-marked diagnostic kit, CLAUDETECT™18.2.

To date, 4 clinical studies have been completed and include GM-IMAB-001 (EudraCT No. 2008-004719-37, referred to as first-in-human [FIM]), PILOT, FAST and GM-IMAB-001-02 (EudraCT No. 2009-017365-36) referred to as MONO.

Zolbetuximab has been granted orphan drug designation for the treatment of stomach cancer by the EMA and FDA.

Clinical data from the studies described above supports the clinical development of zolbetuximab in combination with standard chemotherapy for the treatment of CLDN18.2-positive, HER2-negative locally advanced unresectable or metastatic gastric or GEJ adenocarcinomas.

Please refer to the current IB for most recent clinical data.

1.3 Summary of Key Safety Information for Study Drugs

As of 06 May 2019, zolbetuximab has been administered to 306 subjects including 259 subjects in the following 4 completed clinical studies:

- FIM study as a single monotherapy dose (up to 1000 mg/m^2) in 15 subjects;
- MONO study as repeated monotherapy doses up to 600 mg/m^2 once every 2 weeks in 54 subjects (maximum exposure was 72 infusions);
- FAST study as repeated doses of zolbetuximab in combination with EOX chemotherapy (up to 1000 mg/m^2 once every 3 weeks) in 162 subjects (maximum exposure greater than 40 infusions); and
- PILOT study in combination with immunomodulation therapy (up to 600 mg/m^2 once every 3 weeks) in 28 subjects.

And 2 ongoing open-label clinical studies:

- 8951-CL-0103 (ILUSTRO) as monotherapy or in combination with mFOLFOX6 in 32 subjects
- 8951-CL-0104 as monotherapy in 15 Japanese subjects

Current phase 1/2 study status and enrollment are available in the zolbetuximab Investigator's Brochure (IB) (see end-of-text Table 4.1 in the IB).

Nausea and vomiting have been confirmed as important identified risks as has hypersensitivity reactions (HSRs), including infusion-related reactions (IRRs). Anemia and neutropenia are considered important potential risks. These important identified risks along with important potential risks, based on observations from the clinical studies, are described in Section 5.2 of the IB. Expected adverse drug reactions, including reference safety information (RSI) used for expedited health authority reporting are described in Appendix 1 of the IB.

One patient from an ongoing Phase 2 study (monotherapy arm) experienced grade 4 acute coronary syndrome, grade 4 cardiac arrest, and grade 4 posterior reversible encephalopathy syndrome (PRES), and grade 3 pulmonary embolism, 22 days after the first infusion of zolbetuximab monotherapy. The patient had a medical history significant for uncontrolled hypertension, asthenia and anemia. The patient recovered from these events, which were deemed serious, and a possible causal relationship to zolbetuximab could not be excluded. Refer to the IB for additional details.

In clinical studies, adverse reactions with nausea and/or vomiting and HSRs up to National Cancer Institute-Common Terminology Criteria for Adverse Events (NCI-CTCAE) grade 3 were reported. Subjects receiving zolbetuximab should receive prophylactic antiemetic medications, but do not need to be premedicated for prevention of HSRs and IRRs; however, subjects should be closely monitored for IRRs to facilitate early identification and management. In the case of zolbetuximab-induced nausea, vomiting or hypersensitivity including IRRs, the infusion rate of zolbetuximab may be reduced or the infusion may be

paused or discontinued based on investigator's clinical judgment about severity of toxicity and local standard of care.

Detailed information on the toxicities associated with CAPOX can be found within Section 4.8 of the EU summary of product characteristics (SPC) or local product information for each component.

Potential overlapping toxicities during treatment with zolbetuximab in combination with CAPOX include nausea, vomiting, abdominal pain, constipation, diarrhea, fatigue, HSR, infusion reactions, weight loss and edema.

1.4 Efficacy

1.4.1 Efficacy Results from Study GM-IMAB-001-02 (MONO)

In the MONO study, efficacy analyses included subjects who received zolbetuximab at least once (Full Analysis Set [FAS]) at doses of 300 mg/m^2 (3 subjects) and 600 mg/m^2 (40 subjects). The best overall confirmed response for FAS subjects in the zolbetuximab 600 mg/m^2 dose group was partial response [PR] in 4 subjects (10.0%) that ranged in duration from 43 through 1037 days (GM-IMAB-001, Listing 13.2.6.4). The best overall confirmed response was stable disease (SD) in 6 subjects (15.0%) and progressive disease (PD) in 28 subjects (70.0%) in the 600 mg/m^2 group (GM-IMAB-001-02, Table 12.3.7.1). No subject achieved complete response (CR). Median progression free survival (PFS) in the FAS was 10 weeks (95% confidence interval [CI]: 8.6, 10.1 weeks) in the 600 mg/m^2 group (GM-IMAB-001-02, Table 12.3.3.1).

1.4.2 Efficacy Results from Study GM-IMAB-001-03 (FAST)

Treatment groups in the FAST study are referred to as EOX, EOX + IMAB600 (EOX plus zolbetuximab 600 mg/m^2 once every 3 weeks, with a loading dose of 800 mg/m^2 in cycle 1) and EOX + IMAB1000 (EOX plus zolbetuximab 1000 mg/m^2 once every 3 weeks).

In the FAST study, efficacy analyses of all randomized subjects were included in the intent-to-treat set and included subjects randomized to EOX only (85 subjects), EOX + IMAB600 (79 subjects) and EOX + IMAB1000 (88 subjects). Additional analyses were completed for the FAS, defined as randomized subjects who received at least 1 dose of any study drug (E, Ox, capecitabine or zolbetuximab). The FAS differed from the intent-to-treat set (all randomized subjects) by 6 subjects all of whom discontinued the study early without death or post-baseline tumor assessment. The reasons for early discontinuation of these subjects included protocol violation (1 subject), physician decision (1 subject), withdrawal by subject (2 subjects) and adverse event (AE) (1 subject was anemic and another had a deep vein thrombosis) (GM-IMAB-001-03, Listing 13.2.1).

<u>PFS Based on Central Independent Review (Kaplan-Meier Model, Intent-to-Treat)</u>

The addition of zolbetuximab to EOX led to a statistically significant prolongation of PFS, both for the lower zolbetuximab dose (hazard ratio [HR] 0.45, $P < 0.0005$) and the higher zolbetuximab dose (HR 0.57, $P = 0.0114$). Median PFS was 7.5 months in the EOX +

IMAB600 arm and 7.1 months in the EOX + IMAB1000 arm vs 5.3 months in the EOX arm, representing a median PFS prolongation by 2.2 and 1.8 months, respectively [IB, Table 17].

Overall Survival (Kaplan-Meier, Intent-to-Treat)

The addition of zolbetuximab 600 mg/m^2 led to a statistically significant prolongation in OS (HR 0.52, P < 0.0005). Median OS was 13 months in the EOX + IMAB600 arm vs 8.3 months in the EOX arm, representing an increase in median OS by 4.7 months. In the EOX + IMAB1000 arm, median OS was 9.6 months and hence, numerically longer than in the EOX arm [IB, Table 18]. However, the difference between the groups did not reach statistical significance. The difference in OS between the 2 zolbetuximab doses was statistically significant (P = 0.0406) (GM-IMAB-001-03, Table 12.3.2.1.5.3).

No major imbalances were seen between the treatment groups in the subsequent use of any anticancer therapy (EOX: 38.1%; EOX + IMAB600: 40.3%; EOX + IMAB1000: 34.1%) and any chemotherapy (EOX: 34.5%; EOX + IMAB600: 39.0%; EOX + IMAB1000: 29.4%) (GM-IMAB-001-03, Tables 12.2.1.1.5.1 and 12.2.1.5 [Safety-evaluable Set]).

Best Objective Tumor Response by Independent Review Committee

Based on confirmed responses, the ORR was 38.0% in the EOX + IMAB600 arm, 29.5% in the EOX + IMAB1000 arm and 24.7% in the EOX arm [IB, Table 21]. This included 10.1% of subjects with a CR in the EOX + IMAB600 arm, 4.5% in the EOX + IMAB1000 arm and 3.5% in the EOX arm (GM-IMAB-001-03, Table 12.3.3.1.3).

1.4.3 Efficacy of CAPOX

The results from Study GM-IMAB-001-03 (FAST) using EOX support the use of CAPOX in this study, as CAPOX is similar to EOX except that CAPOX does not include epirubicin. Subjects in this study will receive CAPOX, which is an accepted standard-of-care treatment for subjects with locally advanced unresectable metastatic gastric or GE cancer [NCCN, 2017].

1.5 Risk Benefit Assessment

Zolbetuximab is an investigational agent for the treatment of CLDN18.2-positive, HER2-negative locally advanced unresectable or metastatic gastric or GEJ adenocarcinoma in combination with standard of care oxaliplatin and fluoropyrimidine-based combination chemotherapy as first-line treatment.

Based on clinical efficacy and safety data from the MONO study, preliminary data from the FAST study and supportive preclinical pharmacological studies, there is potential of achieving clinically relevant benefit in combination with oxaliplatin and fluoropyrimidine-based chemotherapy in a first-line setting and as a single agent in the later line setting.

Based on currently available clinical data, zolbetuximab was tolerated from a safety perspective and most observed AEs have been considered manageable. Nonclinical toxicity data were supportive of the clinical findings.

Important identified risks of zolbetuximab are:

- Nausea
- Vomiting
- HSRs (including IRRs)

Important potential risks of zolbetuximab include:

- Neutropenia
- Anemia

Management of the important identified risks associated with zolbetuximab includes antiemetic pretreatment, reduction of the initial zolbetuximab infusion rate, and pausing or discontinuing the infusion based on patient tolerability and investigator's clinical judgment about severity of toxicity and local standard of care. Subjects receiving zolbetuximab do not need to be premedicated for prevention of HSRs and IRRs; however, subjects should be closely monitored for IRRs to facilitate early identification and management.

Overall, the risks, both identified and potential, associated with zolbetuximab in combination with CAPOX are balanced by the anticipated benefits to subjects with CLDN18.2-positive, HER2 negative locally advanced unresectable or metastatic gastric or GEJ adenocarcinoma.

As described in [Section 10.1], an Independent Data Monitoring Committee (IDMC) will be responsible for reviewing the unblinded data from the study to ensure the safety of the subjects.

2 STUDY OBJECTIVE(S), DESIGN AND ENDPOINTS

2.1 Study Objective(s)

2.1.1 Primary Objectives

The primary objective is to evaluate the efficacy of zolbetuximab plus CAPOX compared with placebo plus CAPOX (as first-line treatment) as measured by Progression Free Survival (PFS) in subjects with Claudin (CLDN) 18.2-positive, human epidermal growth factor receptor 2 (HER2)-negative locally advanced unresectable or metastatic gastric and gastroesophageal junction (GEJ) adenocarcinoma.

2.1.2 Secondary Objectives

The secondary objectives are:

- To evaluate efficacy as measured by Overall Survival (OS) as a key secondary objective
- To evaluate the physical function (PF), OG25-Pain and GHS/QoL scores as measured by European Organization for Research and Treatment of Cancer (EORTC) as a key secondary objective
- To evaluate efficacy as measured by Objective Response Rate (ORR)
- To evaluate efficacy as measured by Duration of Response (DOR)
- To evaluate safety and tolerability of zolbetuximab

- To further evaluate other health related quality of life (HRQoL) using additional parameters as measured by EORTC QLQ-C30 and QLQ-OG25 plus STO22 Belching subscale, Global Pain (GP) and the EuroQOL Five Dimensions Questionnaire 5L (EQ5D-5L) questionnaires
- To evaluate the pharmacokinetics of zolbetuximab
- To evaluate the immunogenicity profile of zolbetuximab

2.1.3 Exploratory Objectives

The exploratory objectives are:

- To evaluate efficacy as measured by Time to Progression (TTP)
- To evaluate PFS following subsequent anticancer treatment (PFS2)
- To evaluate Disease Control Rate (DCR)
- To evaluate potential genomic and/or other biomarkers that may correlate with treatment outcome to zolbetuximab and CAPOX.
- To evaluate Health Resource Utilization (HRU)

2.2 Study Design and Dose Rationale

2.2.1 Study Design

This global, multicenter, double-blind, 1:1 randomized, phase 3 study will evaluate efficacy of zolbetuximab plus CAPOX versus placebo plus CAPOX as first-line treatment in subjects with CLDN18.2-positive, HER2-negative locally advanced unresectable or metastatic gastric and GEJ adenocarcinoma.

PFS as assessed by the Independent Review Committee (IRC) is the primary outcome. Secondary outcomes include OS, ORR, DOR, safety and tolerability, HRQoL, pharmacokinetics and the immunogenicity profile of zolbetuximab. Exploratory outcomes include TTP, PFS2, DCR, biomarkers, and HRU.

Approximately 500 subjects will be randomized 1:1 into 1 of 2 treatment arms:

- Arm A (zolbetuximab in combination with CAPOX chemotherapy)
- Arm B (placebo in combination with CAPOX chemotherapy)

Randomization of subjects will be stratified by the following factors:

- Region (Asia vs Non-Asia)
- Number of Organs with Metastatic Sites (0 to 2 vs ≥ 3)
- Prior Gastrectomy (Yes or No)

Screening:

The Screening period is 45 days from full main informed consent form (ICF) signature. Retesting of lab values is allowed within the 45-day Screening period. Re-screening outside the 45-day window under a new subject number may be allowed once and upon discussion with the Medical Monitor (see Re-Screening below). Computerized tomography (CT) scans and magnetic resonance imaging (MRI) conducted as part of a subject's routine clinical

management (i.e., standard of care) obtained before signing the ICF may be utilized for screening or baseline purposes, provided the procedures met the protocol-specified criteria and were performed within the Screening period.

An optional partial screening ICF may be available to allow central testing of tissue for CLDN18.2 and HER2 only.

Formalin fixed paraffin embedded (FFPE) tumor tissue will be collected for central testing to determine CLDN18.2 and HER2 status.

Archival tumor tissue is preferred.

- A minimum of 1 FFPE tumor tissue block (preferred) OR a minimum* of 15 FFPE unstained slides are required as allowed per local policy. If slides are submitted, the slides should be freshly cut from the FFPE block within the time frame described in the laboratory manual.
- If local HER2 results are already available from local testing, a minimum* of 12 FFPE unstained slides are required to be submitted to the central laboratory as allowed per local policy.

  *If the required minimum number of slides is not able to be submitted, sponsor notification and approval is required.
- If the specimen is insufficient or unavailable, a biopsy may be performed to obtain tumor sample.
  - Sponsor pre-approval is required when the sole purpose of the biopsy procedure is to assess eligibility for this study.
  - If the required number of slides cannot be provided, the sponsor or designee should be contacted for further guidance.

Re-Screening:

Subjects who have failed screening are allowed to be re-screened one time after consultation with the Medical Monitor. Upon re-screening, a new subject number will be assigned. Subjects have to re-consent to the study and all screening procedures must be repeated, with the exception of the CLDN18.2 and HER2 testing as well as the radiologic imaging procedure to confirm eligibility if the scan is within 28 days prior to randomization.

Laboratory values re-tested within the original 45-day Screening period are not considered re-screening and no new subject number will be assigned.

Treatment Period:

Subjects will be treated with either zolbetuximab (Arm A) or placebo (Arm B) on day 1 of each cycle until the subject meets study treatment discontinuation criteria. For all study treatments, a cycle is defined as approximately 21 days.

Subjects will also receive up to 8 treatments of CAPOX treatment. Oxaliplatin is administered on day 1 of each cycle, whereas capecitabine is taken twice daily on days 1 through 14. After a maximum of 8 treatments of oxaliplatin, subjects may continue to receive capecitabine taken twice daily on days 1 through 14 of each cycle at the

investigator's discretion until the subject meets study treatment discontinuation criteria. (NOTE: An ECG is required to be performed and assessed locally prior to every oxaliplatin infusion [before any antiemetic treatment] and following completion of every oxaliplatin infusion. ECG should be performed up to 48 hours *prior* to and up to 6 hours *following* every oxaliplatin infusion. Oxaliplatin administration and electrolyte levels should be managed according to investigator judgment for subjects with grade 1 or 2 hypokalemia, hypomagnesemia, and/or hypocalcemia.)

Radiologic imaging will be evaluated every 9 weeks (± 7 days) counting from cycle 1/day 1 (C1D1) for the first 54 weeks and every 12 weeks (± 7 days) thereafter until subject develops radiological disease progression per Response Evaluation Criteria In Solid Tumors (RECIST) 1.1 by IRC or starts other systemic anticancer treatment, whichever occurs earlier.

Study Treatment Discontinuation and Safety Follow-up Visits:

Following discontinuation from zolbetuximab/placebo, subjects will have a zolbetuximab/placebo Study Treatment Discontinuation Visit within 7 days after the decision to discontinue, and 30-day and 90-day Safety Follow-up Visits after their last dose of zolbetuximab/placebo.

Additionally, if CAPOX (both components) is discontinued on a different day than zolbetuximab/placebo, subjects will also have a Study Treatment Discontinuation Visit within 7 days after the decision to discontinue, and 30-day and 90-day Safety Follow-up Visits after the last dose of CAPOX (both components). The CAPOX 30-day and 90-day Safety Follow-up Visits may be conducted by telephone if the subject is unable to visit the study site and will require contact for AE/SAE collection only.

Post-treatment Follow-up Period (for PFS):

If a subject discontinues all study treatments (zolbetuximab/placebo and both components of CAPOX) prior to IRC-confirmed radiological disease progression, the subject will enter the Post-treatment Follow-up Period and continue to undergo scheduled imaging assessments every 9 weeks (±7 days) (or every 12 weeks [±7 days] if subject has been on study over 54 weeks) until radiologic disease progression (i.e., PFS event) per IRC, or until the subject starts any other anticancer treatment, whichever occurs earlier.

If study treatments (zolbetuximab/placebo and both components of CAPOX) are discontinued due to disease progression (PFS event), the subject will enter the Long-term and Survival Follow-up Period.

Long-term Follow-up Period (for PFS2) and Survival Follow-up (for OS) Period:

Following disease progression on first-line treatment or start of any other anticancer treatment, subjects will be followed in the Long-term and Survival Follow-up Period per institutional guidelines, but not less than every 12 weeks. Subsequent anticancer treatment details, progression status and survival status will be collected until PD following subsequent anticancer therapy (PFS2) is documented, or the subject starts another systemic anticancer

treatment, whichever occurs earlier. Radiologic imaging for PFS2 will be done per local standard of care and read locally. Subjects will continue to be followed for survival status (OS) in the Survival Follow-up Period until death (from any cause).

All post-progression details including subsequent anticancer treatment and date and site of progression will be recorded on the electronic case report form (eCRF). Subject contact by phone or other remote method is sufficient during Long-term and Survival Follow-up. Additional follow-up contacts may be required per sponsor request for analysis purposes.

Independent Data Monitoring Committee and Independent Data Analysis Center:

An IDMC will be established and will monitor the ongoing benefit-risk status of study treatment in an unblinded fashion per a pre-defined IDMC charter. The first IDMC meeting will be approximately 6 weeks after the 40th subject enrolled has completed or discontinued cycle 2 (6 weeks) and meetings will be conducted thereafter, as defined in the IDMC charter.

An Independent Data Analysis Center will conduct an interim analysis of OS at the same time as the final PFS analysis, which will occur when approximately 300 PFS events have occurred. This analysis will be utilized by the IDMC to recommend whether the study should be stopped earlier than planned if zolbetuximab in combination with CAPOX has a favorable outcome compared with placebo in combination with CAPOX. If the OS interim analysis demonstrates a highly more favorable outcome for zolbetuximab in combination with CAPOX, the study may be stopped for success. However, any subject continuing to derive clinical benefit from zolbetuximab/placebo in combination with CAPOX, as assessed by the investigator, will be allowed to continue treatment.

2.2.2 Dose Rationale

The dose of zolbetuximab in this study is an 800 mg/m^2 loading dose (C1D1) followed by 600 mg/m^2 every 3 weeks in combination with CAPOX. This dose and schedule of zolbetuximab (800/600 mg/m^2 every 3 weeks) were chosen based on the observed data from the GM-IMAB-001-03 (FAST) study. In the FAST study, addition of zolbetuximab 800/600 mg/m^2 every 3 weeks to EOX (Arm 2) demonstrated a statistically significant and clinically meaningful improvement on PFS and OS in subjects with CLDN18.2-positive advanced gastric/GEJ cancer compared to EOX (Arm 1). The mean serum trough concentration of zolbetuximab was maintained above the targeted value of 50 μg/mL (based on half maximal effective concentration [EC_{50}] of in vitro ADCC and CDC activities) following 800/600 mg/m^2 every 3 weeks administration. A higher zolbetuximab dose (1000 mg/m^2 every 3 weeks) in combination with EOX was added 18 months later (with the allocation ratio of 1:1:7) as Arm 3 in the FAST study to evaluate safety and efficacy at the higher dose. However, this arm had less treatment benefit compared to Arm 2. Although there were numerical differences observed in some demographics and baseline characteristics, the sample size was not sufficient to evaluate and confirm the impact of such differences; therefore, the reason for underperformance in Arm 3 remains inconclusive.

There is no single standard, globally accepted first-line reference chemotherapeutic regimen for advanced gastric cancer. A fluoropyrimidine (capecitabine or 5-FU) in combination with

platinum agent (cisplatin or oxaliplatin) is an accepted standard of care cytotoxic chemotherapy regimen in both Western and Asian countries [NCCN, 2017; Ohtsu et al, 2011; Ajani et al, 2010; Vita et al, 2005; Kim et al, 1993]. Both classes of agents are considered to be interchangeable according to NCCN and European Society for Medical Oncology treatment guidelines. Subjects in this study will receive CAPOX (a combination of capecitabine and oxaliplatin), which is a globally accepted standard-of-care treatment for subjects with locally advanced unresectable or metastatic gastric or GEJ cancer. The safety of this combination regimen is well-documented.

2.3 Endpoints

2.3.1 Primary Endpoints

The primary endpoint is PFS, which is defined as the time from the date of randomization until the date of radiological PD (per RECIST 1.1 by IRC) or death from any cause, whichever is earliest.

2.3.2 Secondary Endpoints

The secondary endpoints are:

- OS, defined as the time from the date of randomization until the date of death from any cause
- Time to confirmed deterioration (TTCD) using the PF, OG25-Pain and GHS/QoL scores as measured by EORTC QLQ-C30 and QLQ-OG25 plus STO22 Belching subscale. TTCD is defined as time to first confirmed deterioration, i.e., time from randomization to first clinically meaningful deterioration that is confirmed at the next scheduled visit.
- ORR, defined as the proportion of subjects who have a best overall response (BOR) of CR or PR as assessed by IRC per RECIST 1.1
- DOR, defined as the time from the date of the first response (CR/PR) until the date of PD as assessed by IRC per RECIST 1.1 or date of death from any cause, whichever is earliest
- Safety and tolerability, as measured by AEs, laboratory test results, vital signs, ECGs and Eastern Cooperative Oncology Group (ECOG) performance status
- HRQoL using the additional parameters as measured by EORTC QLQ-C30, QLQ-OG25 plus STO22 Belching subscale, GP and EQ5D-5L questionnaires
- Pharmacokinetics of zolbetuximab, C_{trough}
- Immunogenicity of zolbetuximab as measured by the frequency of anti-drug antibody (ADA) positive subjects.

2.3.3 Exploratory Endpoints

The exploratory endpoints are:

- TTP, defined as the time from the date of randomization until the date of PD as assessed by IRC per RECIST 1.1.

- PFS2, defined as the time from the date of randomization until the date of PD (per investigator) following subsequent anticancer therapy, death from any cause or start of any other anticancer therapy, whichever is earliest.
- DCR, defined as the proportion of subjects who have a BOR of CR, PR or SD as assessed by IRC per RECIST 1.1.
- Potential genomic and/or other exploratory biomarkers that may be related to treatment outcome of zolbetuximab
- Health Resource Utilization (HRU)

3 STUDY POPULATION

3.1 Selection of Study Population

Subjects with locally advanced unresectable or metastatic gastric or GEJ adenocarcinoma whose tumors are CLDN18.2 positive, HER2-negative and who have not been previously treated for metastatic disease with chemotherapy (1st line).

For the purpose of this study, CLDN18.2-positive is defined as CLDN18.2 expression in ≥ 75% of tumor cells demonstrating moderate to strong membranous staining as determined by central IHC testing.

3.2 Inclusion Criteria

Waivers to the inclusion criteria will **NOT** be allowed.

General Criteria:

1. Institutional Review Board (IRB)/Independent Ethics Committee (IEC) approved written informed consent and privacy language as per national regulations (e.g., Health Insurance Portability and Accountability Act [HIPAA] Authorization for US sites) must be obtained from the subject or legally authorized representative (if applicable) prior to any study-related procedures.

2. Subject is considered an adult (e.g., ≥ 18 years of age in the US) according to local regulation at the time of signing the informed consent.

3. A female subject is eligible to participate if she is not pregnant (negative serum pregnancy test at screening; female subjects with elevated serum beta human chorionic gonadotropin (βhCG) and a demonstrated non-pregnant status through additional testing are eligible) and at least 1 of the following conditions applies:

   - Not a woman of childbearing potential (WOCBP) as defined in [Appendix 12.3 Contraception Requirements]

   OR

   - WOCBP who agrees to follow the contraceptive guidance as defined in [Appendix 12.3 Contraception Requirements] throughout the treatment period and for 9 months after the final administration of oxaliplatin and 6 months after the final administration of all other study drugs.

4. Female subject must agree not to breastfeed starting at screening and throughout the study period, and for 6 months after the final study treatment administration.
5. Female subject must not donate ova starting at screening and throughout the study period, and for 9 months after the final administration of oxaliplatin and 6 months after the final administration of all other study drugs.
6. A male subject with female partner(s) of childbearing potential must agree to use contraception as detailed in [Appendix 12.3 Contraception Requirements] during the treatment period and for 6 months after the final study treatment administration.
7. A male subject must not donate sperm during the treatment period and for 6 months after the final study treatment administration.
8. Male subject with a pregnant or breastfeeding partner(s) must agree to remain abstinent or use a condom for the duration of the pregnancy or time partner is breastfeeding throughout the study period and for 6 months after the final study treatment administration.
9. Subject agrees not to participate in another interventional study while receiving study drug in present study.

Disease Specific Criteria:

10. Subject has histologically confirmed diagnosis of Gastric or GEJ adenocarcinoma.
11. Subject has radiologically confirmed locally advanced unresectable or metastatic disease within 28 days prior to randomization.
12. Subject has radiologically evaluable disease (measurable and/or non-measurable) according to RECIST 1.1, per local assessment, ≤ 28 days prior to randomization. For subjects with only 1 evaluable lesion and prior radiotherapy ≤ 3 months before randomization, the lesion must either be outside the field of prior radiotherapy or have documented progression following radiation therapy.
13. Subject's tumor expresses CLDN18.2 in ≥ 75% of tumor cells demonstrating moderate to strong membranous staining as determined by central IHC testing.
14. Subject has a HER2-negative tumor as determined by local or central testing on a gastric or GEJ tumor specimen.

Physical or Laboratory Findings:

15. Subject has ECOG performance status 0 or 1.
16. Subject has predicted life expectancy ≥ 12 weeks in the opinion of the investigator.
17. Subject must meet all of the following criteria based on the centrally or locally analyzed laboratory tests collected within 14 days prior to randomization. In the case of multiple sample collections within this period, the most recent sample collection with available results should be used to determine eligibility.

a. Hemoglobin (Hgb) ≥ 9 g/dL. Subjects requiring transfusions are eligible if they have a post-transfusion Hgb ≥ 9 g/dL.
b. Absolute Neutrophil Count (ANC) ≥ $1.5x10^9$/L
c. Platelets ≥ $100x10^9$/L
d. Albumin ≥ 2.5 g/dL
e. Total bilirubin ≤ 1.5 x upper limit of normal (ULN) without liver metastases (or < 3.0 x ULN if liver metastases are present)
f. Aspartate aminotransferase (AST) and alanine aminotransferase (ALT) ≤ 2.5 x ULN without liver metastases (or ≤ 5 x ULN if liver metastases are present)
g. Estimated creatinine clearance ≥ 30 mL/min
h. Prothrombin time/international normalized ratio (PT/INR) and partial thromboplastin time (PTT) ≤ 1.5 x ULN (except for subjects receiving anticoagulation therapy)

3.3 Exclusion Criteria

Waivers to the exclusion criteria will **NOT** be allowed.

Subject who meets any of the following exclusion criteria prior to enrollment is not eligible for enrollment:

Prohibited Treatment or Therapies:

1. Subject has received prior systemic chemotherapy for locally advanced unresectable or metastatic gastric or GEJ adenocarcinoma. However, subject may have received either neo-adjuvant or adjuvant chemotherapy, immunotherapy or other systemic anticancer therapies as long as it was completed at least 6 months prior to randomization.
2. Subject has received radiotherapy for locally advanced unresectable or metastatic gastric or GEJ adenocarcinoma ≤ 14 days prior to randomization and has not recovered from any related toxicity.
3. Subject has received treatment with herbal medications or other treatments that have known antitumor activity within 28 days prior to randomization.
4. Subject has received systemic immunosuppressive therapy, including systemic corticosteroids within 14 days prior to randomization. Subject using a physiologic replacement dose of hydrocortisone or its equivalent (defined as up to 30 mg per day of hydrocortisone or up to 10 mg per day of prednisone), receiving a single dose of systemic corticosteroids, or receiving systemic corticosteroids as premedication for radiologic imaging contrast use is eligible.
5. Subject has received other investigational agents or devices within 28 days prior to randomization.

Medical History or Concurrent Disease:

6. Subject has prior severe allergic reaction or intolerance to known ingredients of zolbetuximab or other monoclonal antibodies, including humanized or chimeric antibodies.

7. Subject has known immediate or delayed hypersensitivity, intolerance or contraindication to any component of study treatment.
8. Subject has prior severe allergic reaction or intolerance to any component of CAPOX.
9. Subject has known dihydropyrimidine dehydrogenase (DPD) deficiency. (NOTE: Screening for DPD deficiency should be conducted per local requirements.)
10. Subject has a complete gastric outlet syndrome or a partial gastric outlet syndrome with persistent/recurrent vomiting.
11. Per investigator judgment, subject has significant gastric bleeding and/or untreated gastric ulcers that exclude the subject from participation.
12. Subject has a known history of a positive test for human immunodeficiency virus (HIV) infection or known active hepatitis B (positive HBs Ag) or C infection. NOTE: Screening for these infections should be conducted per local requirements.
    a. For subjects who are negative for HBs Ag, but HBc Ab positive, an HB DNA test will be performed, and if positive, the subject will be excluded.
    b. Subjects with positive hepatitis C virus (HCV) serology but negative HCV RNA test are eligible.
    c. Subjects treated for HCV with undetectable viral load results are eligible.
13. Subject has an active autoimmune disease that has required systemic treatment within the past 3 months prior to randomization.
14. Subject has active infection requiring systemic therapy that has not completely resolved within 7 days prior to randomization.
15. Subject has significant cardiovascular disease, including any of the following:
    a. Congestive heart failure (defined as New York Heart Association [NYHA] Class III or IV), myocardial infarction, unstable angina, coronary angioplasty, coronary stenting, coronary artery bypass graft, cerebrovascular accident (CVA), or hypertensive crisis within 6 months prior to randomization;
    b. History of clinically significant ventricular arrhythmias (i.e., sustained ventricular tachycardia, ventricular fibrillation, or Torsades de Pointes);
    c. QTc interval > 450 msec for male subjects; QTc interval > 470 msec for female subjects;
    d. History or family history of congenital long QT syndrome
    e. Cardiac arrhythmias requiring anti-arrhythmic medications (Subjects with rate controlled atrial fibrillation for > 1 month prior to randomization are eligible.)
16. Subject has history of central nervous system (CNS) metastases and/or carcinomatous meningitis from gastric/GEJ cancer.
17. Subject has known peripheral sensory neuropathy > grade 1 unless the absence of deep tendon reflexes is the sole neurological abnormality.
18. Subject has had a major surgical procedure ≤ 28 days prior to randomization.

   a. Subject without complete recovery from a major surgical procedure ≤ 14 days prior to randomization.

19. Subject has psychiatric illness or social situations that would preclude study compliance, per investigator judgment.
20. Subject has another malignancy for which treatment is required per investigator’s clinical judgment.
21. Subject has any concurrent disease, infection, or co-morbid condition that interferes with the ability of the subject to participate in the study, which places the subject at undue risk or complicates the interpretation of data in the opinion of the investigator.

4 IDENTIFICATION OF STUDY TREATMENT(S)

4.1 Zolbetuximab (Investigational Product)

The investigational product, zolbetuximab, is a sterile lyophilized powder with the chimeric (mouse/human IgG1) monoclonal antibody zolbetuximab as the active pharmaceutical ingredient.

The investigational product is supplied by Astellas in single-use glass vials containing 105 mg of zolbetuximab. All excipients are animal component free and of compendial grade (Pharm. Eur. current version). No preservatives are contained, since the vial is designed for single use.

The investigational product should be stored at refrigerated conditions (2°C to 8°C; 36°F to 46°F). Temperature should be controlled and monitored. Details of investigational product receipt, labeling, storage and preparation are provided in the Pharmacy Manual and Infusion Guidelines.

The zolbetuximab used in this study will be prepared, packaged, and labeled under the responsibility of qualified staff at Astellas Pharma Global Development, Inc. (APGD), Astellas US Technologies, Inc. or sponsor’s designee in accordance with APGD or sponsor’s designee Standard Operating Procedures (SOPs), Good Manufacturing Practices (GMP) guidelines, International Council for Harmonisation (ICH) Good Clinical Practice (GCP) guidelines, and applicable local laws/regulations.

Each vial and carton will bear a label conforming to regulatory guidelines, GMP and local laws and regulations that identifies the contents as investigational drug.

As required, a qualified person of Astellas Pharma Europe B.V. (APEBV) or sponsor’s designee will perform the final release of the medication according to the requirements of the EU Directive 2003/94/EC annex 13.

4.2 0.9% Sodium Chloride Injection

0.9% Sodium Chloride Injection will be used for infusion solution preparation in this study for both zolbetuximab arm and placebo arm. 0.9% Sodium Chloride Injection will not be manufactured or provided to sites by the sponsor. Sites should use their own commercially

obtained supply of 0.9% Sodium Chloride Injection. Details of preparation of infusion solution are provided in the Pharmacy Manual and Infusion Guidelines. ***SPECIFIC TO JAPAN:*** In this study, 0.9% Sodium Chloride Injection is not considered an 'investigational product' as defined in J-GCP.

For manufacturing, formulation, storage, handling and preparation details please refer to the package insert, SPC or local product information supplied by the manufacturer.

4.3 Comparative Drug (Placebo)

Placebo will not be manufactured or provided by the sponsor. Sites should use their own commercial supply of 0.9% Sodium Chloride Injection as placebo infusion solution. Details of preparation of placebo infusion solution are provided in the Pharmacy Manual and Infusion Guidelines.

4.4 CAPOX (Backbone Treatment)

Capecitabine and Oxaliplatin (CAPOX) are administered in combination with zolbetuximab/placebo. CAPOX products should be given according to institutional standards, published guidelines, the respective product package insert(s) or dosed according to this protocol.

CAPOX treatment will be supplied by the responsible site pharmacy of each investigational site or by the sponsor at the sponsor's discretion. Generic drug may be used where approved by the respective regulatory authority. CAPOX treatment will be prepared and/or dispensed by the responsible site pharmacy of each investigational site.

For manufacturing, formulation, storage, handling and preparation details please refer to the package insert, SPC or local product information supplied by the manufacturer.

If CAPOX is supplied by the sponsor, CAPOX used in this study will be packaged and labeled under the responsibility of qualified staff at APGD/Astellas US Technologies, Inc. (AUST) sponsor's designee in accordance with APGD-AUST or sponsor's designee SOPs, GMP guidelines, ICH GCP guidelines, and applicable local laws/regulations.

Each product will bear a label conforming to regulatory guidelines, GMP and local laws and regulations that identifies the contents as investigational drug.

As required, a qualified person of APEBV or sponsor's designee will perform the final release of the medication according to the requirements of the EU Directive 2003/94/EC annex 13.

4.5 Other Drug(s)

4.5.1 Antiemetic Premedication

Prophylactic antiemetics will not be provided by the sponsor but rather will be sourced by the Sites via commercial supply.

For manufacturing, formulation, storage, handling and preparation details please refer to the package insert, SPC or local product information supplies by the manufacturer.

4.6 Study Drug Handling

Current ICH GCP Guidelines require the investigator to ensure that study treatment and other drug deliveries from the sponsor are received by the investigator or designee and that:

- Such deliveries are recorded,
- Study drug is handled and stored according to labeled storage conditions,
- Study drug with appropriate expiry/retest and is only dispensed to study subjects in accordance with the protocol, and
- Any unused study drug is returned to the sponsor, unless prior approval is received from the sponsor allowing local standard procedures for the alternative disposition of unused study drug.

Study drug inventory and accountability records will be kept by the investigator, head of study site (***SPECIFIC TO SITES IN JAPAN***) or designee. Study drug accountability throughout the study must be documented and reconciled. The following guidelines are therefore pertinent:

- The investigator agrees not to supply study drugs to any persons except the eligible subjects in this study in accordance with the protocol.
- The investigator or designee will keep the study drugs in a pharmacy or other locked and secure storage facility under controlled storage conditions, accessible only to those authorized by the investigator to dispense these study drugs.
- A study drug inventory will be maintained by the investigator or designee. The inventory will include details of material received and a clear record of when they were dispensed and to which subject.
- At the conclusion or termination of this study, the investigator or designee agrees to conduct a final drug supply inventory and to record the results of this inventory on the Drug Accountability Record. It must be possible to reconcile delivery records with those of used and/or returned study drug. Any discrepancies must be accounted for and documented. Appropriate forms of deliveries and returns must be signed by the site staff delegated this responsibility.
- The site staff must return unused study drug to the sponsor or designee at the end of the study or upon expiration unless otherwise approved by the sponsor.

4.7 Blinding

4.7.1 Blinding Method

The clinical study will be conducted as subject- and investigator-blinded. Subjects will be randomized to receive zolbetuximab or placebo in a blinded fashion such that neither the investigator, sponsor's study management team, clinical staff, nor subject will know which agent is being administered. All pharmacy-related tasks must be conducted by an unblinded pharmacist/designee. The unblinded pharmacist/designee must not share any unblinded information with the blinded site personnel

Upon the need for unblinding (e.g., real-time assessment of the safety data), the chair of the IDMC can ask for the release of the treatment assignment for 1 or more subjects and will identify the sponsor staff who will receive the information.

4.7.2 Confirmation of the Indistinguishability of the Study Drugs

The appearance of the diluted zolbetuximab solution for infusion is identical to commercial saline solution (placebo). In order to maintain the blind, the subjects randomized to the placebo treatment arm (Arm B) will receive placebo in a volume and route corresponding to the appropriate zolbetuximab dose (Arm A). The unblinded pharmacist/designee will provide the investigator or designee with blinded study drugs to dose the subjects. Refer to the Pharmacy Manual and Infusion Guidelines for detailed information.

4.7.3 Retention of the Assignment Schedule and Procedures for Treatment Code Breaking

The randomization list and study drug blind will be maintained by the Interactive Response Technology (IRT) system.

4.7.4 Breaking the Treatment Code for Emergency

The treatment code for each randomized subject will be provided by the IRT in the event of a medical emergency requiring knowledge of the treatment assigned to the subject. The IRT will be programmed with blind-breaking instructions that may only be requested by the investigator or subinvestigators designated to have access to perform blind-break. No subjects or other study personnel, other than the unblinded pharmacist or designee, will be made aware of the treatment given to any subject unless a medical emergency necessitates such disclosure. In case of a medical emergency, the investigator has the sole responsibility for determining if unblinding of subject's treatment assignment is warranted. Subject safety must always be the first consideration in making such determination. If the investigator decides that unblinding is warranted, the investigator should make every effort to contact the sponsor prior to unblinding a subject's treatment assignment unless this could delay emergency treatment for the subject. Any unblinding by the investigators must be reported immediately to the sponsor and must include an explanation of why the study drug was unblinded.

The investigator must have confirmed functionality to access code-break through the IRT system and must have a designated back up (e.g. redundant processes) to support emergency unblinding requirements.

Prior to randomization, subjects should be provided with information that includes the site emergency contact number and back-up contact number in case of a medical emergency. Any unblinding by the investigational staff must be reported immediately to the sponsor and include an explanation of why the study drug was unblinded. If unblinding is associated with a SAE the investigator is to follow the instructions in [Section 5.5.5 Reporting of SAE].

Care should be taken to limit knowledge of the randomization arm, in case this could affect the blinding of other subjects or future study assessment for the subject.

The time, date, subject number and reason for obtaining any of these codes, and therefore breaking the blind, must be documented in the study file.

If possible, the sponsor should be contacted prior to unblinding of the study drug.

4.7.5 Breaking the Treatment Code by the Sponsor

The sponsor may break the treatment code for subjects who experience a Suspected Unexpected Serious Adverse Reaction (SUSAR), in order to determine if the individual case or a group of cases requires expedited regulatory reporting. Individual Emergency Codes will be provided to the limited staff who are responsible to break the codes for all SUSAR cases for reporting purposes.

4.8 Assignment and Allocation

Subject randomization will be performed by blinded site user via IRT and treatment assigned in a 1:1 ratio to zolbetuximab or placebo. Prior to the initiation of the study treatment, the unblinded pharmacist/designee will be notified by the IRT system about the randomly assigned treatment. The unblinded pharmacist/designee will dispense the treatment according to the IRT system's assignment. Specific procedures for randomization through the IRT are contained in the IRT manual.

Randomization will be stratified by:

- Region (Asia vs Non-Asia)
- Number of Organs with Metastatic Sites (0 to 2 vs ≥ 3)
- Prior Gastrectomy (Yes or No)

5 TREATMENTS AND EVALUATION

5.1 Dosing and Administration of Study Drug(s) and Other Medication(s)

5.1.1 Dose/Dose Regimen and Administration Period

5.1.1.1 Zolbetuximab/Placebo

Subjects will be administered zolbetuximab/placebo as a minimum 2-hour intravenous infusion on day 1 of each cycle. **Guidance for slowing the initial infusion to minimize toxicity can be found in the Pharmacy Manual and Infusion Guidelines.** Subjects will be administered with a loading dose of 800 mg/m² of zolbetuximab at C1D1 followed by subsequent doses of 600 mg/m² every 3 weeks. Zolbetuximab/placebo should be administered prior to CAPOX. Flow rate data will be collected in the eCRF.

Please also refer to the dosing schematics for details [Section V].

Intravenous infusion may be interrupted or slowed down to manage toxicity. Please refer to Pharmacy Manual and Infusion Guidelines for more detailed information.

5.1.1.2 Antiemetics

Antiemetic premedication (prophylactic antiemetics) should be administered prior to each study treatment.

(NOTE: Subjects receiving zolbetuximab do not need to be premedicated for prevention of IRRs; however, subjects should be closely monitored for IRRs to facilitate early identification and management.)

- Antiemetic premedication
  - IV antiemetic premedication should be initiated prior to treatment, or
  - Oral antiemetic premedication should be initiated at a minimum of 30 minutes prior to treatment.
- It is recommended that the prophylactic antiemetic regimen include (but is not limited to) the following agents:
  - NK-1 receptor blockers
  - 5-HT3 receptor blockers*

  *To minimize the risk of Torsades de Pointes, administer 5-HT3 receptor blockers with caution to subjects who have or may develop QTc prolongation.

Antiemetic premedication should be administered according to institutional standard of care, published guidelines and the respective product package insert(s).

Corticosteroids:

- The impact of corticosteroids on the potential efficacy of zolbetuximab is not known. Therefore, consideration should be given to avoid or minimize the use of corticosteroids as a prophylactic antiemetic, if possible.
- For a subject's first dose of zolbetuximab/placebo, it is recommended that the prophylactic use of corticosteroids be avoided.

5.1.1.3 CAPOX

Subjects will receive CAPOX treatment until IRC confirmed disease progression or a total of 8 treatments over 8 or more cycles (each cycle is defined as approximately 3 weeks = approximately 21 days). Oxaliplatin is administered on day 1 of each cycle, whereas capecitabine is taken twice daily on days 1 through 14.

After 8 treatments of CAPOX, subjects may continue to receive capecitabine twice daily on days 1 through 14 of each cycle at the investigator's discretion until the subject meets study treatment discontinuation criteria.

CAPOX should be administered after zolbetuximab/placebo infusion.

- Oxaliplatin: 130 mg/m^2 intravenous infusion on day 1 of each cycle over 2 hours (or longer per institutional standard of care) for a maximum of 8 treatments. (NOTE: ECG is required to be performed and assessed locally prior to every oxaliplatin infusion [before any antiemetic treatment] and following completion of every oxaliplatin infusion. ECG should be performed up to 48 hours *prior* to and up to 6 hours *following* every oxaliplatin infusion. Oxaliplatin administration and electrolyte levels should be managed according to investigator judgment for subjects with grade 1 or 2 hypokalemia, hypomagnesemia, and/or hypocalcemia.)

- Capecitabine: Administered orally at 1000 mg/m^2 twice daily (bid) (total daily dose is 2000 mg/m^2) on days 1 through 14 of each cycle. Capecitabine can be continued beyond 8 treatments based on investigator's judgment.
    - When possible, the first dose of Capecitabine on day 1 should be administered on site under the supervision of the site staff.
    - In case the first dose of Capecitabine on day 1 of a cycle is administered too late in the day for the second dose to be administered on the same day, the last dose of that cycle should be taken in the morning of day 15.
    - Capecitabine should be taken within 30 minutes after a meal.

5.1.2 Study Treatment Dose Modifications, Delays And Interruptions

5.1.2.1 Increase or Reduction of zolbetuximab/Placebo

Dose increase or dose reduction for zolbetuximab/placebo is not allowed. Body surface area should be recalculated at minimum if there is a weight change of at least 10% since the last dose calculation.

5.1.2.2 Zolbetuximab/Placebo Interruption or Permanent Discontinuation

There is a +7 calendar day allowable window for dosing zolbetuximab/placebo. If zolbetuximab/placebo treatment is delayed more than 7 calendar days then it should be administered as soon as the reason for delay has resolved, which will then become day 1 of the next cycle. The timing of subsequent doses should be scheduled using the date of the last dose administration. If zolbetuximab/placebo treatment is delayed, CAPOX/Capecitabine administration should also be delayed at the same time and can be restarted when zolbetuximab/placebo administration has been restarted, unless there are other reasons warranting further delay of CAPOX/Capecitabine at the investigator's discretion (refer to Section 5.1.2.4). Refer to the Pharmacy Manual and Infusion Guidelines for more detailed information on incomplete dosing.

A delay of zolbetuximab/placebo treatment of > 28 days from when the next zolbetuximab/placebo treatment was scheduled to begin (> 49 days from when the last dose of zolbetuximab/placebo began) to be administered due to unresolved toxicity associated with zolbetuximab/placebo will result in the subject discontinuing zolbetuximab/placebo.

Radiologic imaging is to be scheduled every 9 weeks (±7 days) counting from C1D1 for the first 54 weeks and then every 12 weeks (±7 days) thereafter; the schedule should be maintained regardless of treatment delay.

Note: Intravenous infusion of zolbetuximab/placebo should be administered as a minimum 2-hour infusion. Intravenous infusion may be interrupted or slowed down to manage toxicity. Please refer to Pharmacy Manual and Infusion Guidelines for more detailed information.

Guidelines for zolbetuximab/placebo treatment modification due to non-hematologic and hematologic toxicities, regardless of investigator assessment of relationship to zolbetuximab/placebo, are described below in Table 2 and Table 3, respectively.

One case of PRES has been reported in subjects receiving zolbetuximab. **Discontinue zolbetuximab/placebo if PRES is suspected.** Confirm PRES diagnosis by brain imaging, preferably by magnetic resonance imaging (MRI).

Table 2 Guidelines for Zolbetuximab/Placebo Treatment Modification Due to Non-hematologic Toxicity

<table>
<tr><th>Toxicity</th><th>Grade 1</th><th>Grade 2</th><th>Grade 3</th><th>Grade 4</th></tr>
<tr><td>Infusion-related reaction (IRR) other than nausea, vomiting or abdominal pain.
See Table 4 for further IRR management guidance</td><td>Continue Infusion</td><td>Interrupt infusion. Infusion may be resumed at a reduced rate when toxicity has improved to grade ≤ 1.</td><td colspan="2">Stop the infusion immediately.
Institute appropriate medical management immediately based on the type of reaction.
Permanently Discontinue zolbetuximab/placebo</td></tr>
<tr><td>Nausea</td><td colspan="2">Continue Infusion</td><td>Interrupt infusion. Hold zolbetuximab/placebo treatment until toxicity has improved to grade ≤ 1, then restart the infusion at a lower rate.
If the investigator determines that the toxicity is not related to zolbetuximab and the toxicity has improved to grade ≤ 2, then infusion may be restarted at the investigator's discretion at a lower rate.</td><td>Not applicable*</td></tr>
<tr><td>Vomiting</td><td>Continue Infusion</td><td>Continue infusion; however, if infusion was held due to grade 3 vomiting, hold infusion until vomiting has improved to ≤ grade 1.</td><td>Interrupt infusion. Hold zolbetuximab/placebo treatment until toxicity has improved to grade ≤ 1 and then restart infusion at a lower rate.</td><td>Permanently Discontinue zolbetuximab/ placebo</td></tr>
<tr><td>Other Non-hematologic toxicity</td><td colspan="2">Continue Infusion</td><td>Interrupt infusion. Hold zolbetuximab/placebo treatment until toxicity has improved to grade ≤ 1.
If the investigator determines that the toxicity is not related to zolbetuximab and the toxicity has improved to grade ≤ 2, then infusion may be restarted at the investigator's discretion.#</td><td>Permanently Discontinue zolbetuximab/ placebo</td></tr>
<tr><td>PRES</td><td colspan="4">Discontinue zolbetuximab/placebo if PRES is suspected.</td></tr>
</table>

Footnotes appear on next page

* Grade 4 nausea is not defined in CTCAE v4.03. If investigator assesses nausea as grade 4, manage per local standard of care.

# For subjects with a pulmonary embolism, treatment can continue without resolving to grade 2 or less.

Table 3 Guidelines for Zolbetuximab/Placebo Treatment Modification Due to Hematologic Toxicity

Toxicity	Grade 1	Grade 2	Grade 3	Grade 4
**Neutropenia†**	ANC < LLN to 1500/mm^3; < LLN to 1.5 x 10^9/L	ANC < 1500 to 1000/mm^3; < 1.5 to 1.0 x 10^9/L	ANC < 1000 to 500/mm^3; < 1.0 to 0.5 x 10^9/L	ANC < 500/mm^3; < 0.5 x 10^9/L
Action	Continue treatment	Continue treatment; however, if treatment was held due to ≥ grade 3 neutropenia, hold treatment until ANC improves to ≤ grade 1.	Hold treatment and recheck blood counts weekly until ANC resolves to ≥ 1.5 x 10^9/L (≤ grade 1) before restarting treatment. Discontinue zolbetuximab/placebo if ANC remains < 1.5 x 10^9/L (≥ grade 2) after a > 28-day treatment delay from when the next study treatment was scheduled to be administered.	
**Febrile Neutropenia†**	Not Applicable		ANC < 1000/mm^3 with a single temperature of > 38.3°C (101°F) or a sustained temperature of ≥ 38°C (100.4°F) for more than 1 hour.	Life-threatening consequences; urgent intervention indicated
Action			Follow standard/local management guidelines.<br>Hold treatment and recheck blood counts weekly until ANC recovers to ≥ 1.5 x 10^9/L (grade ≤ 1) and fever has resolved. Discontinue zolbetuximab/placebo if ANC remains < 1.5 x 10^9/L (≥ grade 2) after a > 28-day treatment delay from when the next study treatment was scheduled to be administered.	
**Thrombocytopenia**	PLT < LLN to 75,000/mm^3; < LLN to 75.0 x 10^9/L	PLT < 75,000 to 50,000/mm^3; < 75.0 to 50.0 x 10^9/L	PLT < 50,000 to 25,000/mm^3; < 50.0 to 25.0 x 10^9/L	PLT < 25,000/mm^3; < 25.0 x 10^9/L
Action	Continue treatment	Continue treatment; however, if treatment was held due to grade 3 or higher thrombocytopenia, hold treatment until thrombocytopenia improves to ≤ grade 1.	Withhold treatment and recheck blood counts weekly until platelets recover to > 75× 10^9/L (grade ≤ 1) before restarting treatment.<br>Discontinue zolbetuximab/placebo if platelets remain < 75 x 10^9/L (grade ≥ 2) after a > 28-day treatment delay from when the next study treatment was scheduled to be administered.	

Table continued on next page

**Anemia†**	Hgb < LLN to 10.0 g/dL; < LLN to 6.2 mmol/L; < LLN to 100 g/L	Hgb < 10.0 to 8.0 g/dL; < 6.2 to 4.9 mmol/L; < 100 to 80 g/L	Hgb < 8.0 g/dL; < 4.9 mmol/L; < 80 g/L; transfusion indicated	Life-threatening consequences; urgent intervention indicated
Action	Continue treatment		Withhold treatment. Follow standard treatment guidelines. Transfuse if indicated. Recheck blood counts weekly until Hgb recovers to > 8.0 g/dL (≤ grade 2) before restarting treatment. Discontinue zolbetuximab/ placebo if Hgb remains < 8.0 g/dL (≥ grade 3) after a > 28-day treatment delay from when the next study treatment was scheduled to be administered.	Withhold treatment. Follow standard treatment guidelines including urgent intervention and blood transfusions as required. The subject may be discontinued after discussion with the Medical Monitor.

ANC: absolute neutrophil count; PLT: platelet count; Hgb: hemoglobin
† At the investigator's discretion growth factors may be used according to standard practice guidelines.

5.1.2.3 Guidelines for Infusion-related Reactions for Zolbetuximab/Placebo

Subjects should be closely monitored for IRRs to facilitate early identification and management.

The management of such toxicities should be based on investigator utilizing institutional standard of care, published guidelines and the general guidelines provided in Table 4 below.

A subject with an infusion reaction should be evaluated specifically for the symptoms and signs that are highly suggestive of anaphylaxis (urticaria, repetitive cough, wheeze and throat tightness/change in voice). A careful examination of the skin is advised in order to detect urticaria, which often appears first in the neck, trunk, abdomen and axillae.

Not all anaphylactic reactions manifest as anaphylactic shock. Because anaphylaxis can recur and worsen with re-exposure, permanently discontinue zolbetuximab/placebo for any subject having a reaction with features (even if mild) that are highly suggestive of anaphylaxis.

Note: Intravenous infusion of zolbetuximab/placebo should be administered as a minimum 2-hour infusion. Intravenous infusion may be interrupted or slowed down to manage toxicity. Please refer to Pharmacy Manual and Infusion Guidelines for more detailed information.

Table 4 Infusion-related Reactions

**Infusion-Related Reactions**	
Refer to Table 2 for management of infusion related reactions of nausea, vomiting or abdominal pain	
**CTCAE v4.03 Grade**	**Management**
Grade 1 standard infusion reactions ***other than nausea, vomiting or abdominal pain***#	Continue infusion and closely monitor the subject.
Grade 2 standard infusion reaction ***other than nausea, vomiting or abdominal pain***#	Interrupt.<br>Medical management as per type of reaction. Resume infusion once toxicity grade ≤ 1 and reduce the infusion rate for the remaining infusion.<br>For the next infusion:<br>• Increase total infusion time (reduce infusion rate).<br>• Pre-medicate as appropriate.*<br>• Closely monitor the subject for symptoms and signs of an infusion reaction.
Any infusion reaction with features of anaphylaxis<br>**OR**<br>Grade 3 or 4 standard infusion reactions ***other than nausea, vomiting or abdominal pain***#	Stop the infusion immediately.<br>Institute appropriate medical management immediately based on the type of reaction.<br>Permanently Discontinue zolbetuximab/placebo<br>*Once the subject has been stabilized, collect blood for cytokine/chemokine panel and serum total tryptase level (levels typically peak within 3 hours after the onset of symptoms) and send to the central laboratory.*

CTCAE v4.03: Common Terminology Criteria For Adverse Events

* At the investigators discretion, antihistamines may be used as premedication for the next infusion. Systemic corticosteroids should be avoided or minimized while subject is on study treatment unless required for management of an emergent medical condition (e.g., severe nausea/vomiting or hypersensitivity reaction).

# For grade 3 or 4 IRR of nausea, vomiting or abdominal pain, collect blood for cytokine/chemokine panel and serum total tryptase level and send to the central laboratory.

5.1.2.4 CAPOX Dose Modification

The CAPOX dose modifications described below are based on [XELOX CCO Formulary, August 2017].

There is a + 7 calendar day allowable window for CAPOX dosing. If CAPOX treatment is delayed more than 7 calendar days then it should be administered at the next scheduled zolbetuximab/placebo treatment visit, i.e. day 1 of the next cycle. The timing of subsequent doses should be scheduled using the date of the last dose administration.

If Capecitabine treatment is delayed or interrupted, Capecitabine can be administered as soon as the reason for delay or interruption is resolved. The delay/interruption in treatment should be regarded as lost treatment days and missed doses should not be replaced; the planned treatment schedule should be maintained.

Dosing and dose modification should be based on investigator utilizing institutional standard of care, approved package insert, SPC or local product information supplied

by the manufacturer for each agent (oxaliplatin, capecitabine) and general guidelines provided below.

The first dose of CAPOX should not be modified. After the assessment of tolerability, dose adjustments should be performed based on investigator judgment utilizing institutional standard of care, approved package insert, SPC, or local product information and/or the recommended criteria in Table 5 based on maximum hematologic or non-hematologic toxicity data from the previous cycle as shown in Table 6 and Table 7, respectively. Dose reduction criteria for oxaliplatin-related neurotoxicity are presented in Table 8. Each drug may be dose reduced independently based on the specific types of toxicities observed. It is recommended that no more than 2 dose reductions per drug per subject occur (see Table 5). Dose re-escalation is not recommended after treatment-related AEs. Dose re-escalation of capecitabine is permitted after the subject has completed 8 cycles or 8 doses of CAPOX and/or has permanently discontinued oxaliplatin if the investigator chooses to continue capecitabine. If further dose reduction is required beyond the criteria in Table 5, that component of CAPOX should be discontinued.

In subjects experiencing toxicity requiring a delay or discontinuation of CAPOX, subject should continue to receive zolbetuximab/placebo as clinically appropriate. If CAPOX is interrupted, subject should be evaluated weekly (at a minimum) until the toxicity has improved sufficiently at which time treatment can be restarted as described in the tables below (as applicable). A delay of CAPOX treatment for > 28 days from when the next CAPOX treatment was scheduled to begin (> 49 days from when the last CAPOX dose began) due to unresolved toxicity associated with CAPOX will result in the subject discontinuing CAPOX (both components).

Table 5 Recommended Dose Adjustment Levels for Oxaliplatin and Capecitabine

**Drug**		**Oxaliplatin**	**Capecitabine**
**Initial Dose**		130 mg/m^2	1000 mg/m^2 bid
**Dose Reduction**	**Level 1**	100 mg/m^2	750 mg/m^2 bid
	**Level 2**	75 mg/m^2	500 mg/m^2 bid

For the first 8 treatments, if a subject has capecitabine discontinued or interrupted, oxaliplatin should be discontinued or interrupted until capecitabine is resumed. For additional information, refer to the approved package insert, SPC or local product information supplied by the manufacturer for each agent (oxaliplatin, capecitabine).

In case Capecitabine administration is delayed or interrupted, the missed doses should not be taken later during the cycle. Capecitabine administration is to be stopped at day 14 (or the morning of day 15 as applicable) of each cycle.

5.1.2.5 CAPOX: Dose Modifications for Hematologic Toxicity

The CAPOX dose modifications for hematologic toxicity are presented in Table 6. Dose modifications should be maintained until recovery from hematologic toxicity. A delay of CAPOX treatment for > 28 days from when the next CAPOX treatment was scheduled to

begin (> 49 days from when the last CAPOX dose began) due to hematologic toxicity associated with CAPOX will result in the subject discontinuing CAPOX (both components).

Dosing and dose modification should be based on investigator utilizing institutional standard of care, approved package insert, SPC or local product information supplied by the manufacturer for each agent (oxaliplatin, capecitabine) and general guidelines provided below.

Table 6 CAPOX Dose Modification Due to Hematologic Toxicity

**Toxicity**	**Grade 1**	**Grade 2**	**Grade 3**	**Grade 4**
**Neutropenia†**	ANC < LLN to 1500/mm^3; < LLN to 1.5 x 10^9/L	ANC < 1500 to 1000/mm^3; < 1.5 to 1.0 x 10^9/L	ANC < 1000 to 500/mm^3; < 1.0 to 0.5 x 10^9/L	ANC < 500/mm^3; < 0.5 x 10^9/L
Action	Continue treatment.	CAPOX:<br>1st, 2nd and 3rd events: Interrupt until event resolves to grade ≤ 1.<br><br>Fourth event: Discontinue treatment permanently	Hold treatment and recheck blood counts weekly until ANC resolves to ≥ 1.5 x 10^9/L before restarting treatment.<br>Discontinue CAPOX if ANC remains < 1.5 x 10^9/L after a > 28-day treatment delay from when the next study treatment was scheduled to be administered.	
Dose Modification (for next treatment)	Maintain dose level.	Oxaliplatin: Maintain dose of oxaliplatin<br><br>Capecitabine: First event: Maintain dose level<br><br>Second event: Reduce capecitabine 1 level.†<br><br>Third event: Reduce capecitabine 2 levels	Oxaliplatin Dose adjustment next cycle: Reduce 1 dose level<br><br>Capecitabine Dose adjustment next cycle:<br><br>First event: Reduce capecitabine 1 level<br><br>Second event: Reduce capecitabine 2 levels.†<br><br>Third event: Discontinue CAPOX	Discontinue both. If investigator deems it to be in the subject's best interest to continue, resume next dose of oxaliplatin reduced 1 level and capecitabine reduced 2 dose levels
**Febrile Neutropenia†**			ANC < 1000/mm^3 with a single temperature of > 38.3°C (101°F) or a sustained temperature of ≥ 38°C (100.4°F) for more than 1 hour.	Life-threatening consequences; urgent intervention indicated

Table continued on next page

**Toxicity**	**Grade 1**	**Grade 2**	**Grade 3**	**Grade 4**
Action	Maintain dose level	CAPOX:<br>1st, 2nd and 3rd events: Interrupt until event resolves to grade ≤ 1.<br><br>Fourth event: Discontinue treatment permanently	Follow standard treatment guidelines	Proceed to next treatment when fever has resolved and ANC recovers to ≥ 1.5 x 10^9/L within 28 days from when the next study treatment was scheduled to be administered.
Dose Modification (at next treatment)	Maintain dose level	Oxaliplatin: Maintain dose of oxaliplatin<br><br>Capecitabine: First event: Maintain dose level<br><br>Second event: Reduce capecitabine 1 level.†<br><br>Third event: Reduce capecitabine 2 levels	Oxaliplatin Dose adjustment next cycle: Reduce 1 dose level<br><br>Capecitabine First event: Reduce capecitabine 1 level<br><br>Second event: Reduce capecitabine 2 levels.†<br><br>Third event: Discontinue CAPOX	Discontinue both. If physician deems it to be in the patient's best interest to continue, interrupt until resolved to grade 0-1 and resume next dose of oxaliplatin reduced 1 level and capecitabine by reducing 2 dose levels
**Thrombocytopenia**	PLT < LLN to 75,000/mm^3; < LLN to 75.0 x 10^9/L	PLT < 75,000 to 50,000/mm^3; < 75.0 to 50.0 x 10^9/L	PLT < 50,000 to 25,000/mm^3; < 50.0 to 25.0 x 10^9/L	PLT < 25,000/mm^3; < 25.0 x 10^9/L
Action	Continue treatment.	CAPOX:<br>1st, 2nd and 3rd events: Interrupt until event resolves to grade ≤ 1.<br><br>Fourth event: Discontinue treatment permanently	Withhold treatment and recheck blood counts weekly* until platelets recover to > 75× 10^9/L before restarting treatment. Discontinue CAPOX if PLT remains < 75 x 10^9/L after a > 28-day delay from when the next study treatment was scheduled to be administered.	
Table continued on next page				

Toxicity	Grade 1	Grade 2	Grade 3	Grade 4
Dose Modification (for next treatment)	Maintain dose level.	Oxaliplatin: Maintain dose of oxaliplatin<br><br>Capecitabine: First event: Maintain dose level<br><br>Second event: Reduce capecitabine 1 level.<br><br>Third event: Reduce capecitabine 2 levels	Oxaliplatin Dose adjustment next cycle: Reduce 1 dose level<br><br>Capecitabine First event: Reduce capecitabine 1 level<br><br>Second event: Reduce capecitabine 2 levels.<br><br>Third event: Discontinue CAPOX	Discontinue both. If physician deems it to be in the patient's best interest to continue, interrupt until resolved to grade 0-1 and resume next dose of oxaliplatin reduced 1 level and capecitabine by reducing 2 dose levels

ANC: absolute neutrophil count; CAPOX: Capecitabine and Oxaliplatin; LLN: lower limit of normal; PLT: platelet count
† At the investigator's discretion growth factors may be used according to standard practice guidelines.
* Weekly recheck with ±3 day window for thrombocytopenia

5.1.2.6 CAPOX: Dose Modification for Non-hematologic Toxicity

CAPOX dose modifications for non-hematologic toxicity should be based on the most severe toxicity experienced during the last treatment (Table 7). Retreatment should be delayed until recovery of all non-hematologic toxicity to ≤ grade 2 with the exception of increased bilirubin or ALT, which must recover to grade 1 or baseline, whichever was higher. The maximum permitted treatment delay is 28 days from when the next study treatment was scheduled to begin (49 days from when the last dose of study treatment began) for recovery of non-hematologic toxicity. If the subject has not recovered sufficiently to meet retreatment criteria within that timeframe, CAPOX should be discontinued.

ECG is required to be performed and assessed locally prior to every oxaliplatin infusion (before any antiemetic treatment) and following completion of every oxaliplatin infusion. ECG should be performed up to 48 hours *prior* to and up to 6 hours *following* every oxaliplatin infusion. Ensure that potassium, magnesium, and calcium levels are grade 2 or less prior to oxaliplatin infusion. Oxaliplatin may be administered in the setting of grade 1 or grade 2 hypokalemia, hypomagnesemia, and/or hypocalcemia, based on investigator judgment.

During or following study treatment, additional ECG monitoring should be initiated per local standard of care for subjects who experience syncope, presyncope, palpitations and/or bradycardia.

- If the QTc interval is > 450 msec, medically manage per local standard of care, including correction of hypokalemia, hypomagnesemia, and/or hypocalcemia.
- If the QTc interval is > 500 msec, medically manage per local standard of care, withhold capecitabine and oxaliplatin treatment, ensure appropriate (continuous) ECG monitoring,

and obtain cardiology consultation. If the QTc interval resolves to ≤ 450 msec, the subject may resume treatment at 1 reduced dose level.

Dosing and dose modification should be based on investigator utilizing institutional standard of care, approved package insert, SPC or local product information supplied by the manufacturer for each agent (oxaliplatin, capecitabine) and general guidelines provided below.

Table 7 CAPOX Dose Modification Due to Non-hematologic Toxicity

<table>
<tr><th>Toxicity</th><th>Grade 1</th><th>Grade 2</th><th>Grade 3</th><th>Grade 4</th></tr>
<tr><td colspan="5">Diarrhea</td></tr>
<tr><td>Action</td><td>Continue treatment</td><td>Start medical management for diarrhea. Continue treatment.</td><td colspan="2">Start medical management for diarrhea. Withhold all CAPOX treatment. Restart treatment after diarrhea recovers to ≤ grade 2</td></tr>
<tr><td>Dose Modification (for next treatment)</td><td>None</td><td>First event:
None
Second event:
Reduce capecitabine 1 dose level.</td><td>First event:
Reduce oxaliplatin 1 level and capecitabine 1 level.
Second event:
Reduce oxaliplatin 1 dose level and capecitabine 2 levels.</td><td>Reduce oxaliplatin 1 dose level and capecitabine 2 levels.</td></tr>
<tr><td colspan="5">Other Non-hematologic Toxicities†‡</td></tr>
<tr><td>Action</td><td>Continue treatment</td><td>CAPOX:
1st, 2nd and 3rd events: Interrupt until event resolves to grade ≤ 1

Fourth event:
Discontinue treatment permanently</td><td colspan="2">Withhold all CAPOX treatment until toxicity improves to ≤ grade 2.</td></tr>
<tr><td>Dose Modification (for next treatment)</td><td>None</td><td>Oxaliplatin:
Maintain dose of oxaliplatin

Capecitabine:
First event:
Maintain dose level

Second event:
Reduce capecitabine 1 level.†

Third event:
Reduce capecitabine 2 levels</td><td>Oxaliplatin Dose adjustment next cycle: Reduce 1 dose level

Capecitabine
First event:
Reduce capecitabine 1 level

Second event:
Reduce capecitabine 2 levels.†

Third event:
Discontinue CAPOX</td><td>Discontinue both If physician deems it to be in the patient's best interest to continue, interrupt until resolved to grade 0-1 and resume next dose of oxaliplatin reduced 1 level and capecitabine by reducing 2 dose levels</td></tr>
</table>

CAPOX: Capecitabine and Oxaliplatin

† For skin toxicity, reduce capecitabine only, not oxaliplatin.

‡ Exceptions: alopecia, fatigue, anorexia, nausea/vomiting (if can be controlled by antiemetics) and constipation (if can be controlled with laxatives, stool softeners, etc.).

5.1.2.7 Guidelines for Infusion-related Reactions for Oxaliplatin

Subjects should be closely monitored for IRRs to facilitate early identification and management. The management of such toxicities should be based on investigator utilizing institutional standard of care.

5.1.2.8 Oxaliplatin-induced Neurotoxicity

Oxaliplatin is known to be associated with peripheral neuropathy, including paresthesia and dysesthesia of the hands, feet and perioral region. Subjects treated with oxaliplatin in this study should be advised to avoid cold drinks and exposure to cold water or air, especially within 3 to 5 days of receiving oxaliplatin. Dose modifications for oxaliplatin related to neurotoxicity are presented in Table 8.

Cases of PRES have been reported in subjects receiving Oxaliplatin combination chemotherapy. Discontinue Oxaliplatin if PRES is suspected. Confirm PRES diagnosis by brain imaging, preferably by MRI.

Table 8 Oxaliplatin Dose Modification for Associated Neurotoxicity

<table>
<tr><th>Toxicity/Duration</th><th>Grade 1</th><th>Grade 2</th><th>Grade 3</th><th>Grade 4</th></tr>
<tr><td>Paresthesia or dysesthesia</td><td>Paresthesia or dysesthesia‡ that does not interfere with function</td><td>Paresthesia or dysesthesia‡, interfering with function, but not activities of daily living</td><td>Paresthesia or dysesthesia‡ with pain or with functional impairment that also interferes with activities of daily living</td><td>Persistent paresthesia or dysesthesia that is disabling or life-threatening</td></tr>
<tr><td>1 to 7 Days</td><td rowspan="3">No dose reduction</td><td rowspan="2">No dose reduction</td><td>First Event: Reduce oxaliplatin by 1 dose level at next treatment. Second Event: Reduce oxaliplatin by a second dose level at next treatment.</td><td rowspan="3">Discontinue oxaliplatin</td></tr>
<tr><td>> 7 Days</td><td>For Grade 3 neurotoxicity > 7 days/persistent between treatments, discuss oxaliplatin discontinuation with Medical Monitor.</td></tr>
<tr><td>Persistent between treatments†</td><td>Reduce oxaliplatin by 1 dose level at next treatment</td><td>For Grade 3 neurotoxicity > 7 days/persistent between treatments, discuss oxaliplatin discontinuation with Medical Monitor</td></tr>
<tr><td>Acute laryngopharyngeal dysesthesia‡
(during or after the 2-hour infusion)</td><td colspan="4">Discontinue current infusion.
At next treatment, consider pretreatment with benzodiazepines and increasing duration of infusion as clinically indicated per investigator discretion.</td></tr>
</table>

NA: not applicable

† Not resolved by the beginning of the next treatment.

‡ May be cold induced.

5.1.2.9 Oxaliplatin-induced Laryngopharyngeal Dysesthesia

Oxaliplatin has also been associated with laryngopharyngeal dysesthesia, which is an unusual loss of sensation of breathing (acute respiratory distress) without any objective evidence of respiratory distress (hypoxia, laryngospasm or bronchospasm). Laryngopharyngeal dysesthesia may be induced or exacerbated upon exposure to cold.

Subjects developing laryngopharyngeal dysesthesia should have their oxygen saturation evaluated via a pulse oximeter. If results are normal, reassurance should be provided, a benzodiazepine or other anxiolytic agent should be considered, and the subject should remain for observation in the clinic until the episode has resolved. After resolution, the oxaliplatin infusion may then be continued at one-third the rate.

Because laryngopharyngeal dysesthesia may be associated with the rate of oxaliplatin infusion, subsequent infusions of oxaliplatin should be prolonged from a normal 2-hour infusion to a 6-hour infusion.

Subjects receiving oxaliplatin should avoid consuming cold drinks or ice chips on day 1 of each cycle, as this may exacerbate oral or throat dysesthesia, as well as laryngopharyngeal dysesthesia. Administration of prophylactic medication such as Mg^{2+}/Ca^{2+} infusions or others is at the discretion of the investigator.

The symptoms and treatments of laryngopharyngeal dysesthesia and platinum HSRs are compared in Table 9.

Table 9 Comparison of the Symptoms and Treatment of Laryngopharyngeal Dysesthesia and Platinum Hypersensitivity Reactions

Clinical Symptoms	Laryngopharyngeal Dysesthesia	Platinum Hypersensitivity
Dyspnea	Present	Present
Bronchospasm	Absent	Present
Laryngospasm	Absent	Present
Anxiety	Present	Present
O_2 saturation	Normal	Decreased
Difficulty swallowing	Present (loss of sensation)	Absent
Pruritus	Absent	Present
Urticaria/rash	Absent	Present
Cold-induced symptoms	Yes	No
Blood pressure	Normal or increased	Normal or decreased
Treatment	Reassurance, anxiolytics, observation in a controlled clinical setting until symptoms abate or at the physician’s discretion	Oxygen, steroids, epinephrine, bronchodilators; fluids and vasopressors, if appropriate

5.1.2.10 Allergic Reaction to Oxaliplatin

Subjects developing grade 1 or 2 allergic reaction to oxaliplatin should receive premedication according to institutional practice prior to further administration of oxaliplatin. Appropriate premedication should also be given if grade 1 to 2 allergic reaction persists into the next cycle. Oxaliplatin should be discontinued in subjects developing grade 3 to 4 allergic reactions.

Oxaliplatin should be interrupted pending further investigation in subjects experiencing respiratory symptoms indicative of pulmonary fibrosis such as nonproductive cough, dyspnea, crackles, rales, hypoxia, tachypnea or radiological pulmonary infiltrates. Oxaliplatin should be permanently discontinued in subjects with confirmed interstitial pulmonary fibrosis.

5.1.2.11 Extravasation of Oxaliplatin

Necrosis has been seen in conjunction with extravasation of oxaliplatin. Subjects with suspected extravasation should have their infusion stopped and the drug administered at another site. Extravasation should be treated according to institutional guidelines.

5.1.3 Treatment Compliance

The dose and schedule of zolbetuximab/placebo and CAPOX administered to each subject will be recorded on the appropriate eCRF at every cycle. Reasons for dose delay or omission will also be recorded. This information will be used to assess compliance with the treatment. Subjects will be provided with Capecitabine tablets for self-administration and are asked to return any unused tablets and empty packaging at the next scheduled visit for accountability.

5.1.4 Criteria for Continuation of Treatment

Zolbetuximab may be made available after conclusion of the study to subjects who are still receiving and benefitting from study treatment until a study-defined treatment discontinuation criterion is met.

5.1.4.1 Discontinuation of CAPOX (both components) and Continuation of Zolbetuximab/Placebo

If a subject discontinues CAPOX (or either component of CAPOX) due to any reason other than disease progression as confirmed by IRC, they may continue on zolbetuximab/placebo at the discretion of the investigator provided that all of the following have been met:

- the subject completed at least 2 treatments of CAPOX treatment;
- the subject will not receive another systemic chemotherapy, immunotherapy, radiotherapy or other treatment intended for antitumor activity; and
- in the investigator’s opinion the subject continues to derive clinical benefit with acceptable toxicity.

Subjects should continue to follow the Study Treatment Period Schedule of Assessments [Table 1].

5.1.4.2 Discontinuation of Zolbetuximab/Placebo and Continued of CAPOX (one or both components)

If zolbetuximab/placebo is permanently discontinued first for reasons other than PD as confirmed by IRC and no other anticancer treatment is started, subjects may continue to receive CAPOX/Capecitabine until treatment discontinuation criteria are met. Subjects should continue to follow the Study Treatment Period Schedule of Assessments [Table 1].

5.1.4.3 If Both Zolbetuximab/Placebo and CAPOX (both components) are discontinued

If both zolbetuximab/placebo and CAPOX (both components) are permanently discontinued for reasons other than IRC-confirmed radiological PD, the subject should enter the Post-Treatment Follow-up Period and continue to undergo imaging assessments per Schedule of Assessments [Table 1].

5.1.5 Previous and Concomitant Treatment (Medication and Non-Medication Therapy)

All medications and concomitant treatments administered from the time of full main informed consent through the 90-day safety follow-up visit must be recorded in the eCRF. Documentation will include the medication name, indication, route and dates of administration.

Prohibited Concomitant Treatment

The following are strictly prohibited:

- Sorivudine or analogs (during capecitabine treatment)
- Concurrent non-steroid systemic immunosuppressive agents (for systemic corticosteroids, see cautionary concomitant treatment below).
- Live vaccines should be avoided during the treatment period in which subject is receiving oxaliplatin or capecitabine and up to 6 months after final oxaliplatin or capecitabine dose. In cases where a live vaccine is needed for COVID-19 prevention and allowed per local regulations, please contact the Medical Monitor for discussion.
- Other systemic chemotherapy, immunotherapy, radiotherapy, herbal medications or other treatments intended for antitumor activity.
  - Palliative radiotherapy for peripheral bone metastases is allowed.
  - For gastric bleeding, palliative radiotherapy is prohibited, but esophagogastroduodenoscopy with epinephrine injection is allowed.
- Investigational products or therapy other than zolbetuximab.

Cautionary Concomitant Treatment

Considerations should be given to avoid or minimize the use of the following concomitant medications, if possible, during zolbetuximab/placebo treatment:

- Systemic corticosteroids, because their impact on the potential efficacy of zolbetuximab is not known.
  - Systemic corticosteroids should be avoided or minimized while subject is on study treatment unless required for management of an emergent medical condition (e.g., severe nausea/vomiting or hypersensitivity reaction).
  - For a subject's first dose of zolbetuximab/placebo, it is recommended that the prophylactic use of corticosteroids be avoided.
  - Inhaled, intranasal, ophthalmic, otic and topically applied steroids are allowed.
  - Subjects are allowed to use a physiologic replacement dose of hydrocortisone or its equivalent (defined as up to 30 mg/day of hydrocortisone or up to 10 mg/day of prednisone), receive a single dose of systemic corticosteroids or receive systemic corticosteroids as pre-medication for radiologic imaging contrast use.
- Administer 5-HT3 blockers with caution in subjects who have or may develop QTc prolongation.
- Nonsteroidal anti-inflammatory drugs (NSAIDs) because of the potential to cause gastric ulcers and covert bleeding.

  - In such cases where NSAID use is necessary, the use of NSAIDs with lower gastric ulcerogenic potential is preferred and concomitant gastric protection with proton pump inhibitors is recommended.

The following should be avoided or used with caution and closely monitored during capecitabine administration:

- Cytochrome P450 2C9 substrates (Subjects taking coumarin-derivative anticoagulants concomitantly with capecitabine should have PT/INR monitored regularly and anticoagulant dose adjusted accordingly).
- Anti-epileptic medications (e.g., phenobarbital, phenytoin and primidone)

The following should be avoided or used with caution and closely monitored during oxaliplatin administration:

- Medications known to prolong the QT or QTc interval (refer to https://www.crediblemeds.org for a list of these medications

Prohibited and cautionary treatments are described in Appendix 12.4.

5.2 Demographics and Baseline Characteristics

5.2.1 Demographics

Demographic information will be collected for all subjects and will include initials (where permitted), age, date of birth (where permitted), sex, race (where permitted) and ethnicity (where permitted).

5.2.2 Medical History

Medical history includes all significant medical conditions per the judgment of the investigator that have resolved prior to informed consent or are ongoing at the time of full main consent. Details that will be collected include the onset date and recovery date and CTCAE v4.03 grade, if applicable for ongoing conditions.

5.2.3 Diagnosis of the Target Disease, Severity, and Duration of Disease

A complete medical history of the target disease will be recorded during Screening. This will include the subject's medical condition, date of initial diagnosis, tumor location, and other disease specific information as designated in the eCRF.

5.3 Efficacy Assessments

Radiologic imaging will be evaluated at Screening (within 28 days prior to randomization) and every 9 weeks (±7 days) counting from C1D1 for the first 54 weeks and then every 12 weeks (±7 days) thereafter until subject develops radiological disease progression per RECIST 1.1 by IRC or starts other systemic anticancer treatment, whichever comes earlier.

All radiologically-evaluable disease (measurable and/or non-measurable), per local assessment, must be documented at Screening and re-assessed at each subsequent radiologic evaluation. Imaging will include CT scans with contrast of the thorax, abdomen, and pelvis. If CT scan with contrast is medically not feasible, MRI may be used for imaging. Bone scans

(or focal X-ray) or brain imaging should be performed if metastatic disease in bone or brain is suspected, respectively. The same mode of imaging should be utilized throughout the study unless medical necessity requires a change. For randomized subjects, screening imaging should be sent to the central imaging vendor no later than at the time of submission of the first on-treatment imaging. All imaging acquired post randomization will be sent to the central imaging vendor within 7 days of scanning for the blinded IRC assessment of radiological tumor response based on RECIST 1.1 [Eisenhauer et al, 2009]. The investigator should make every effort to immediately submit radiologic assessments for IRC review when PD is suspected. Refer to Imaging Acquisition Guidelines for further detail on scan modality and contrast options.

5.4 Safety Assessment

5.4.1 Vital Signs

Vital signs, including systolic and diastolic blood pressures (mmHg) and radial pulse rate (beats/minute) and temperature, will be obtained and recorded at the times specified in the Schedule of Assessments [Table 1]. All vital sign measurements will be obtained in a consistent manner (sitting or supine) throughout their study participation. Height and weight will be measured using normal institutional standards.

If clinically significant vital sign changes from baseline (pretreatment) are noted, the changes will be documented as AEs on the AE page of the eCRF. Clinical significance will be defined as a variation in vital signs, which has medical relevance as deemed by the investigator that could result in an alteration in medical care. For clinically significant vital sign changes, the investigator will continue to monitor the subject until the parameter returns to grade ≤ 1, or to the baseline (pretreatment) value, or until the investigator determines that follow up is no longer medically necessary.

5.4.2 Observation Period following Zolbetuximab/Placebo Infusion

Following the first dose of zolbetuximab/placebo on C1D1, the subject must be observed for 2 hours post zolbetuximab/placebo infusion. The post-infusion observation period can be conducted during the CAPOX administration. If ≥ grade 2 AEs are observed during infusion or during the post-infusion observation period, subsequent zolbetuximab/placebo infusion times should be extended and subjects should continue to be observed for 2 hours post zolbetuximab/placebo infusion. If the subject does not develop any ≥ grade 2 AEs, the subject should be observed for 1 hour post-infusion for their subsequent zolbetuximab/placebo infusions. The subject should be instructed to notify site personnel if they develop any AEs during this post-infusion observation time period.

In the event of an IRR with features of anaphylaxis (regardless of grade) or grade 3 or 4 IRR, blood samples for cytokine/chemokine panel and serum total tryptase level (levels typically peak within 3 hours after the onset of symptoms) should be collected once the subject has stabilized, for shipment to the central laboratory.

5.4.3 Laboratory Assessments

Below is a table of the laboratory tests that will be performed during the conduct of the study.

Laboratory tests will be performed according to the Schedule of Assessments [Table 1] and must be sent to the central laboratory for analysis.

- Eligibility can be determined based on central and/or local laboratory testing:
    - The most recent laboratory data must be used to confirm the subject's eligibility. In the case of multiple sample collections within 14 days prior to randomization, the most recent sample collection with available results should be used to determine eligibility.
    - Central labs must be collected and submitted to the central laboratory during the Screening period.
    - If retesting of lab values is necessary to confirm eligibility, local labs can be used without requiring additional sample collection for central laboratory submission.
    - The screening labs used to determine eligibility should be collected within 14 days prior to randomization.
- Laboratory test results (central or local) will be reviewed by the investigator prior to any study treatment. Clinical significance of out-of-range laboratory findings is to be determined and documented by the investigator/sub-investigator who is a qualified physician.
- Local laboratory results may be used for treatment decisions; however, central laboratory samples must also be drawn per protocol and sent to the central laboratory.
- Central and local labs may be collected up to 48 hours prior to study treatment.
- Holidays and weekends should be taken into account when scheduling these sample collections.
- Additional assessments may be done centrally or locally to monitor AEs or as clinically indicated.

**Panel/Assessment**	**Parameters to be Analyzed**
Hematology	Hematocrit (Hct)<br>Hemoglobin (Hgb)<br>Red Blood Cell Count (RBC)<br>White Blood Cell Count (WBC)<br>WBC differential<br>Absolute Neutrophil Count (ANC)<br>Platelets<br>Mean Corpuscular Volume (MCV)<br>Mean Corpuscular Hemoglobin (MCH)<br>Mean Corpuscular Hemoglobin Concentration (MCHC)
Table continued on next page	

**Panel/Assessment**	**Parameters to be Analyzed**
Biochemistry	Albumin<br>Blood Urea Nitrogen (BUN)<br>Calcium<br>Bicarbonate<br>Chloride<br>Creatinine<br>Glucose<br>Magnesium<br>Phosphate<br>Potassium<br>Sodium<br>Total Bilirubin<br>Total Protein<br>Alanine Aminotransferase (ALT)<br>Alkaline Phosphatase (ALP)<br>Aspartate Aminotransferase (AST)<br>Creatinine Clearance
Grade 3 or 4 Infusion-related Reactions (IRR)	Cytokine/Chemokine Panel†<br>Serum total tryptase†
Any reaction with features of anaphylaxis	Cytokine/Chemokine Panel†<br>Serum total tryptase†
Dihydropyrimidine dehydrogenase (DPD) deficiency screening†	DPD deficiency alleles
Urinalysis	Color<br>Clarity/turbidity<br>pH<br>Specific gravity<br>Glucose<br>Ketones<br>Nitrites<br>Leukocyte esterase<br>Bilirubin<br>Urobilinogen<br>Blood<br>Protein<br>RBCs<br>WBCs
Coagulation	Prothrombin time (PT) (sec)<br>Partial Thromboplastin Time (PTT)<br>International normalized ratio (INR)
Table continued on next page	

Panel/Assessment	Parameters to be Analyzed
Thyroid Function Test	Thyroid stimulating hormone (TSH)<br>Free T4 (thyroxine)
Urine Pregnancy Test	Human chorionic gonadotropin (HCG)
Serum Pregnancy Test	Human chorionic gonadotropin (HCG)

† As applicable

5.4.4 Physical Examination

Physical examinations will be conducted at visits as outlined in the Schedule of Assessments [Table 1]. Each physical exam should include height (at Screening only), weight and ECOG performance status. A full physical exam is required at Screening. The physical exam only needs to be repeated on C1D1 if clinically significant changes from Screening are observed (in the opinion of the investigator). For all cycles, the physical examination, weight and ECOG performance status assessments can be completed up to 48 hours prior to zolbetuximab/placebo administration. Targeted (symptom driven) physical exams should be conducted every 3 weeks on day 1 of each cycle. If clinically significant worsening of findings from baseline is noted at any study visit, the changes will be documented as AEs on the AE eCRF. Clinical significance is defined as any variation in physical findings, which has medical relevance that could result in an alteration in medical care. The investigator will continue to monitor the subject until the parameter returns to grade ≤ 1, or to the baseline condition, or until the investigator determines that follow up is no longer medically necessary.

5.4.5 Electrocardiogram

A single 12-lead ECG will be performed at the time points outlined in the Schedule of Assessments [Table 1]. Prior to performing ECG, subjects should rest in supine position for 10 minutes. When collected on the same day, ECG should be collected prior to pharmacokinetic samples. Additional ECG may be performed based on medical history and investigator medical judgment. ECGs will be assessed locally. Clinically significant findings should be recorded as an AE.

5.4.6 Performance Status

The ECOG Scale [Oken et al, 1982] will be used to assess performance status. Refer to [Appendix 12.8].

5.5 Adverse Events and Other Safety Aspects

AE collection will begin from time of full main informed consent and continue through the 90 days following the last dose of zolbetuximab/placebo and CAPOX (both components).

Serious adverse events (SAEs), regardless of causality will be collected from the time of full main informed consent through 90 days following the last dose of zolbetuximab/placebo and CAPOX (both components).

AEs will be documented at each clinic visit, but can be collected at any time. Any AE that meets the definition of an SAE will also be reported on a separate form to the sponsor.

5.5.1 Definition of Adverse Events

An AE is any untoward medical occurrence in a subject administered a study drug, and which does not necessarily have to have a causal relationship with this treatment. An AE can therefore be any unfavorable and unintended sign (including an abnormal laboratory finding), symptom, or disease (new or exacerbated) temporally associated with the use of a medicinal product whether or not considered related to the medicinal product.

In order to identify any events that may be associated with study procedures and could lead to a change in the conduct of the study, Astellas collects AEs even if the subject has not received study drug treatment. AE collection begins after the signing of the full main informed consent and will be collected until 90 days after the last dose of study drug or the subject is determined to be a screen failure.

Care will be taken not to introduce bias when detecting AEs and/or SAEs. Open-ended and non-leading verbal questioning of the subject is the preferred method to inquire about AE occurrences.

5.5.1.1 Abnormal Laboratory Findings

Any abnormal laboratory test result (e.g., hematology, clinical chemistry or urinalysis) or other safety assessment (e.g., ECGs, radiology scans, vital signs measurements, physical examination), including those that worsen from baseline, that is considered to be clinically significant in the medical and scientific judgment of the investigator and not related to underlying disease, is to be reported as an (S)AE.

Any clinically significant abnormal laboratory finding or other abnormal safety assessment which is associated with the underlying disease does not require reporting as an (S)AE, unless judged by the investigator to be more severe than expected for the subject's condition.

Repeating an abnormal laboratory test or other safety assessment, in the absence of any of the above criteria, does not constitute an AE. Any abnormal test result that is determined to be an error does not require reporting as an AE.

5.5.1.2 Potential Cases of Drug-Induced Liver Injury

Refer to [Appendix 12.5 Liver Safety Monitoring and Assessment] for detailed instructions on drug induced liver injury. Abnormal values in AST and/or ALT concurrent or with abnormal elevations in total bilirubin that meet the criteria outlined in [Appendix 12.5 Liver Safety Monitoring and Assessment] in the absence of other causes of liver injury, are considered potential cases of drug-induced liver injury (potential Hy's Law cases) and are always to be considered important medical events and reported per [Section 5.5.5 Reporting of Serious Adverse Events].

5.5.1.3 Disease Progression and Study Endpoints

Under this protocol, the following event(s) will not be considered as an (S)AE:

- Disease Progression: events including defined study endpoints that are clearly consistent with the expected pattern of progression of the underlying disease are not to be recorded as AEs unless resulting in death. These data will be captured as efficacy assessment data as outlined in [Section 5.3 Efficacy Assessments]. If there is any uncertainty as to whether an event is due to anticipated disease progression and/or if there is evidence suggesting a causal relationship between the study treatment and the event, it should be reported as an (S)AE. All deaths up to 90 days after the last dose of study drug must be reported as an SAE, even if attributed to disease progression.
- Disease progression can be considered as the worsening of a subject's condition attributable to Gastric or GEJ cancer. It may be an increase in the severity of the disease under study and/or increases in the symptoms of the disease. The development of new, or progression of existing metastases to the primary cancer under study should be considered as disease progression not an AE. Events which are unequivocally due to disease progression should not be reported as an AE during the study.

5.5.2 Definition of Serious Adverse Events

An AE is considered "serious" if, in the view of either the investigator or sponsor, it results in any of the following outcomes:

- Results in death
- Results in life-threatening event (an AE is considered "life-threatening" if, in the view of either the investigator or sponsor, its occurrence places the subject at immediate risk of death. It does not include an AE that, had it occurred in a more severe form, might have caused death)
- Results in persistent or significant disability/incapacity or substantial disruption of the ability to conduct normal life functions
- Results in congenital anomaly, or birth defect
- Requires inpatient hospitalization (except for planned procedures as allowed per study) or leads to prolongation of hospitalization (except if prolongation of planned hospitalization is not caused by an AE). Hospitalization for treatment/observation/ examination caused by AE is to be considered as serious.
- Other medically important events (defined in paragraph below)

Medical and scientific judgment should be exercised in deciding whether expedited reporting is appropriate in other situations, such as important medical events that may not be immediately life-threatening or result in death or hospitalization but may jeopardize the subject or may require intervention to prevent one of the other outcomes listed in the definition above. These events, including those that may result in disability/incapacity, usually are considered serious. Examples of such events are intensive treatment in an emergency room or at home for allergic bronchospasm; blood dyscrasias or convulsions that do not result in hospitalization; or development of drug dependency or drug abuse.

5.5.2.1 Important Medical Events

If an AE occurs that the sponsor determines to be an Important Medical Event, additional information on the event (e.g., investigator confirmation of seriousness, causality) will be requested.

Progression of Gastric or GEJ cancer, including signs and symptoms of progression, should not be reported as an SAE unless it results in death within 90 days of the last dose of study treatment. For progression-related death reported as an SAE, there should be available immediate cause of death reported as the event term. "Death due to disease progression" should be recorded as the AE term only when the cause of death cannot be otherwise determined.

5.5.3 Criteria for Causal Relationship to Study Treatment

A medically qualified investigator is obligated to assess the relationship between each study treatment and each occurrence of each (S)AE. This medically qualified investigator will use medical judgment as well as the RSI [Section 1.3] to determine the relationship. The causality assessment is 1 of the criteria used when determining regulatory reporting requirements. The medically qualified investigator is requested to provide an explanation for the causality assessment for each (S)AE and must document in the medical notes that he/she has reviewed the (S)AE and has provided an assessment of causality.

Following a review of the relevant data, the causal relationship between the study treatment and each (S)AE will be assessed by answering '**yes**' or '**no**' to the question "**Do you consider that there is a reasonable possibility that the event may have been caused by the study treatment**".

When making an assessment of causality, the following factors are to be considered when deciding if there is evidence and/or arguments to suggest there is a 'reasonable possibility' that an (S)AE may have been caused by the study treatment (rather than a relationship cannot be ruled out) or if there is evidence to reasonably deny a causal relationship:

- Plausible temporal relationship between exposure to the study treatment and (S)AE onset and/or resolution. Has the subject actually received the study treatment? Did the (S)AE occur in a reasonable temporal relationship to the administration of the study treatment?
- Plausibility; i.e., could the event have been caused by the study treatment? Consider biologic and/or pharmacologic mechanism, half-life, literature evidence, drug class, preclinical and clinical study data, etc.
- Dechallenge/Dose reduction/Rechallenge:
    - Did the (S)AE resolve or improve after stopping or reducing the dose of the suspect drug? Also consider the impact of treatment for the event when evaluating a dechallenge experience.
    - Did the (S)AE reoccur if the suspected drug was reintroduced after having been stopped?

- Laboratory or other test results; a specific lab investigation supports the assessment of the relationship between the (S)AE and the study treatment (e.g., based on values pre-, during and post-treatment)
- Available alternative explanations independent of study treatment exposure; such as other concomitant drugs, past medical history, concurrent or underlying disease, risk factors including medical and family history, season, location, etc. and strength of the alternative explanation

There may be situations in which an SAE has occurred and the investigator has minimal information to include in the initial report to the sponsor. However, it is very important that the medically qualified investigator always make an assessment of causality for every event before the initial transmission of the SAE data to the sponsor. With limited or insufficient information about the event to make an informed medical judgment and in absence of any indication or evidence to establish a causal relationship, a causality assessment of ‘no’ is to be considered. In such instance, the investigator is expected to obtain additional information regarding the event as soon as possible and to re-evaluate the causality upon receipt of additional information. The medically qualified investigator may revise his/her assessment of causality in light of new information regarding the SAE and shall send an SAE follow-up report and update the eCRF with the new information and updated causality assessment.

5.5.4 Criteria for Defining the Severity of an Adverse Event

AEs, including abnormal clinical laboratory values, will be graded using the NCI-CTCAE v4.03 guidelines. The items that are not stipulated in the NCI-CTCAE v4.03 will be assessed according to the criteria below and entered into the eCRF.

Grade	Assessment Standard
1-Mild	Asymptomatic or mild symptoms, clinical or diagnostic observations noted; intervention not indicated.
2-Moderate	Local or noninvasive intervention indicated.
3-Severe	Medically significant but not immediately life threatening, hospitalization or prolonged hospitalization.
4-Life Threatening	Life threatening consequences, urgent intervention indicated
5-Death	Death related to AE

5.5.5 Reporting of Serious Adverse Events

The collection of AEs and the expedited reporting of SAEs will start following receipt of the signed full main informed consent and will continue until 90 days after the last dose of study treatment or the subject is determined to be a screen failure.

In the case of a SAE, the investigator must contact the sponsor by fax or email immediately (within 24 hours of awareness).

The investigator must complete and submit an SAE worksheet containing all information that is required by local and/or regional regulations to the sponsor by email or fax immediately (within 24 hours of awareness).

The SAE worksheet must be signed by a medically qualified investigator (as identified on Delegation of Authority Log). Signature confirms accuracy and completeness of the SAE data as well as the investigator causality assessment including the explanation for the causality assessment.

If the SAE is associated with emergency unblinding as outlined in [Section 4.7.4 Breaking the Blind in Emergency**]** this is to be recorded on the SAE worksheet. Within the SAE worksheet, the investigator is to include when unblinding took place in association with the SAE.

JAPAN SITES ONLY**:** In the case of a SAE, the investigator or sub-investigator must report to the head of the study site and must contact the sponsor by email or fax immediately (within 24 hours of awareness). The investigator should complete and submit JUTOKUNA YUUGAIJISHOU HOUKOKUSHO containing all information that is required by the Regulatory Authorities to the sponsor by email or fax immediately (within 24 hours of awareness) and to the head of the hospital.

For contact details, see [Section II Contact Details of Key Sponsor's Personnel]. Fax or email the SAE and Special Situations Worksheet to:

Astellas Pharma Global Development, Inc.
Pharmacovigilance
North American Fax: +1-888-396-3750
(North America Alternate Fax: +1-847-317-1241)
International Fax: +44-800-471-5263
Email: safety-us@astellas.com

UNIQUE TO JAPAN REGION*:* JUTOKUNA YUUGAIJISHOU HOUKOKUSHO the SAE and Special Situations Worksheet to:

PAREXEL International
Fax number: 03-6888-5798

If there are any questions, or if clarification is needed regarding the SAE, please contact the sponsor's Medical Monitor/Study Physician or his/her designee [Section II Contact Details of Key Sponsor’s Personnel].

Follow-up information for the event should be sent promptly (within 7 days of the initial notification (***UNIQUE TO JAPAN REGION*****:** within 2 days for the initial notification).

Full details of the SAE or Special Situation should be recorded on the medical records, SAE or Special Situation Worksheet and on the (e)CRF.

The following minimum information is required:

- International Study Number (ISN)/Study number,
- Subject number, sex and age,
- The date of report,
- A description of the SAE (event, seriousness criteria),

- Causal relationship to the study treatment (including reason), and
- The drug provided (if any). Note: blinded regimen is also an option.

The sponsor or sponsor's designee will medically evaluate the SAE and determine if the report meets the requirements for expedited reporting based on seriousness, causality, and expectedness of the events (e.g., SUSAR reporting) according to current local/regional regulatory requirements in participating countries. The sponsor or sponsor's designee will submit expedited safety reports (e.g., Investigational New Drug [IND] Safety Reports, SUSAR, Council for International Organizations of Medical Sciences I Form) to Competent Authorities and concerned Ethics Committee per current local regulations, and will inform the investigators of such regulatory reports as required. Investigators must submit safety reports as required by their IRB/local IEC within timelines set by regional regulations (e.g., EMA, FDA) where required. Documentation of the submission to and receipt by the IRB/local IEC of expedited safety reports should be retained by the study site.

The sponsor will notify all investigators responsible for ongoing clinical studies with the study treatment of all SUSARs which require submission per local requirements IRB/local IEC/ head of the study site (as applicable).

The heads of the study sites (if applicable) and investigators should provide written documentation of IRB/IEC notification for each report to the sponsor.

5.5.6 Follow-up of Adverse Events

All AEs occurring during or after the subject has discontinued the study are to be followed up until resolved or judged to be no longer clinically significant, or until they become chronic to the extent that they can be fully characterized by the investigator.

If after the protocol defined AE collection period [see Section 5.5.1 Definition of Adverse Event], an AE progresses to an SAE, or the investigator learns of any (S)AE including death, where he/she considers there is reasonable possibility it is related to the study treatment or study participation, the investigator must promptly notify the sponsor.

5.5.7 Monitoring of Common Serious Adverse Events

Common SAEs are SAEs commonly anticipated to occur in the study population independent of drug exposure. SAEs classified as "common" are provided in [Appendix 12.6 Common Serious Adverse Events] for reference. The list does NOT change the investigator's reporting obligations, nor his obligations to perform a causality assessment, or prevent the need to report an AE meeting the definition of an SAE as detailed above. The purpose of this list is to alert the investigator that some events reported as SAEs may not require expedited reporting to the regulatory authorities based on the classification of "common SAEs" as specified in [Appendix 12.6 Common Serious Adverse Events]. The sponsor will monitor these events throughout the course of the study for any change in frequency. Any changes to this list will be communicated to the participating investigational sites. Investigators must report individual occurrences of these events as stated in [Section 5.5.5 Reporting of Serious Adverse Events].

5.5.8 Adverse Events of Special Interest

In case of zolbetuximab induced nausea, vomiting or hypersensitivity/IRR, infusion rate of zolbetuximab/placebo may be reduced or infusion paused or discontinued based on investigator's clinical judgment about severity of toxicity and local standard of care.
See [Section 5.1.2 Study Treatment Dose Modifications, Delays and Interruptions] and the Pharmacy Manual and Infusion Guidelines.

If the AEs of interest are classified as serious, they are to be collected via the SAE worksheet and reported within 24 hours as described in [Section 5.5.5 Reporting of Serious Adverse Events].

5.5.9 Special Situations

Certain Special Situations observed in association with the study treatment(s), such as incorrect administration (e.g., wrong dose of study treatment, comparator or background therapy) are collected in the eCRF, as Protocol Deviations per [Section 8.2 Major Protocol Deviations] or may require special reporting, as described below. These Special Situations are not considered AEs, but do require to be communicated to Astellas as per the timelines defined below.

If a Special Situation is associated with, or results in, an AE, the AE is to be assessed separately from the Special Situation and captured as an AE in the eCRF. If the AE meets the definition of a SAE, the SAE is to be reported as described in [Section 5.5.5 Reporting of Serious Adverse Events] and the details of the associated Special Situation are to be included in the clinical description on the Special Situation worksheet.

The Special Situations are:

- Pregnancy
- Medication error, Overdose and "Off label use"
- Misuse/abuse
- Occupational exposure
- (Suspicion of) Transmission of infectious agent
- Suspected Drug-Drug interaction

5.5.9.1 Pregnancy

If a female subject becomes pregnant during the study dosing period or within 9 months after the final administration of oxaliplatin and 6 months after the final administration of all other study drugs, or if a partner of a male subject becomes pregnant during the study dosing period or within 6 months from the discontinuation of dosing, the investigator is to report the information to the sponsor according to the timelines in [Section 5.5.5 Reporting of Serious Adverse Events] using the Pregnancy Reporting Form and in the eCRF.

The expected date of delivery or expected date of the end of the pregnancy, last menstruation, estimated conception date, pregnancy result and neonatal data etc., should be included in this information.

While pregnancy itself is not considered to be an AE or SAE, any pregnancy complication or termination (including elective termination) of a pregnancy is to be reported for a female study subject as an AE in the eCRF or SAE per [Section 5.5.5 Reporting of Serious Adverse Events]. For (S)AEs experienced by a female partner of a male subject, (S)AEs are to be reported via the Pregnancy Reporting Form.

Additional information regarding the outcome of a pregnancy when also categorized as an SAE is mentioned below:

- "Spontaneous abortion" includes miscarriage, abortion and missed abortion.
- Death of a newborn or infant within 1 month after birth is to be reported as an SAE regardless of its relationship with the study drug.
- If an infant dies more than 1 month after the birth, is to be reported if a relationship between the death and intrauterine exposure to the study drug is judged as "possible" by the investigator.
- Congenital anomaly (including anomaly in miscarried fetus)

Unless a congenital anomaly is identified prior to spontaneous abortion or miscarriage, the embryo or fetus should be assessed for congenital defects by visual examination. (S)AEs experienced by the newborn/infant should be reported via the Pregnancy Reporting Form. Generally, follow up will be no longer than 6 to 8 weeks following the estimated delivery date.

5.5.9.2 Medication Error, Overdose and "Off-Label Use"

If a Medication Error, Overdose or "Off-label Use" (i.e., use outside of what is stated in the protocol) is suspected, refer to [Section 8.2 Major Protocol Deviations]. Any associated (S)AEs are to be reported in the eCRF. If the AE meets the definition of a SAE, the SAE is also to be reported as described in [Section 5.5.5 Reporting of Serious Adverse Events] together with the details of the medication error, overdose and/or "Off-Label Use".

There is no antidote for overdose of the study drug. In the event of suspected zolbetuximab overdose, the subject should receive supportive care and monitoring (including, but not limited to, inpatient hospitalization). The Medical Monitor should be contacted as applicable.

In the event of suspected CAPOX overdose, refer to the approved package insert, SPC or local product information supplied by the manufacturer for each agent.

5.5.9.3 Misuse/Abuse

If misuse or abuse of the study drug(s) is suspected, the investigator must forward the Special Situation worksheet to the sponsor/delegated contract research organization (CRO) by fax or email immediately (within 24 hours of awareness). Any associated (S)AEs are to be reported in the eCRF. If the AE meets the definition of a SAE, the SAE is also to be reported as described in [Section 5.5.5 Reporting of Serious Adverse Events] together with details of the misuse or abuse of the study drug(s).

5.5.9.4 Occupational Exposure

If occupational exposure (e.g., inadvertent exposure to the study drug(s) of site staff whilst preparing it for administration to the subject) to the study drug(s) occurs, the investigator must forward the Special Situation worksheet to the sponsor/delegated CRO by fax or email immediately (within 24 hours of awareness). Any associated (S)AEs occurring to the individual associated with or resulting from the Special Situation are to be reported on the Special Situations worksheet.

5.5.9.5 Suspected Drug-Drug Interaction

If a suspected drug-drug interaction associated with the study drug(s) is suspected, the investigator must forward the Special Situation worksheet to the sponsor/delegated CRO by fax or email immediately (within 24 hours of awareness). Any associated (S)AEs are to be reported in the eCRF. If the AE meets the definition of a SAE, the SAE is also to be reported as described in [Section 5.5.5 Reporting of Serious Adverse Events] together with details of the suspected drug-drug interaction.

5.5.10 Supply of New Information Affecting the Conduct of the Study

When new information becomes available necessary for conducting the clinical study properly, the sponsor will inform all investigators involved in the clinical study as well as the regulatory authorities. Investigators should inform the IRB/IEC of such information when needed.

The investigator will also inform the subjects, who will be required to sign an updated ICF in order to continue in the clinical study.

UNIQUE TO JAPAN REGION:

1. When information is obtained regarding serious and unexpected adverse drug reactions (or other) that are specified in Article 273 of the Act on Securing Quality, Efficacy and Safety of Pharmaceuticals, Medical Devices, Regenerative and Cellular Therapy Products, Gene Therapy Products, and Cosmetics, in compliance with Article 80-2 Paragraph 6 of the Pharmaceutical Affairs Law, the sponsor should inform all the investigators involved in the clinical study, the head of the study site, and the regulatory authorities of such information. The head of the study site who receives such information will decide whether the clinical study should be continued after hearing the opinions of the IRB. The investigator will supply the new information to the subjects, in compliance with [Appendix 12.1.4.2 Supply of New and Important Information Influencing the Subject’s Consent and Revision of the Written Information].
2. In addition to the above item (1), when the head of the study site receives the revisions of the IB, protocol or written information, information on the matters covering the quality of the study drug, efficacy and safety, information necessary for conducting the clinical study properly, or documents to be examined by the IRB these documents should be sent to the IRB.

5.5.11 Urgent Safety Measures

An Urgent Safety Measure (USM) is an intervention, which is not defined by the protocol and can be put in place with immediate effect without needing to gain prior approval by the sponsor, relevant Competent Authorities, IRB/IEC, where applicable, in order to protect study participants from any immediate hazard to their health and/or safety. Either the investigator or the sponsor can initiate a USM. The cause of a USM can be safety, product or procedure related.

5.5.12 Reporting Urgent Safety Measures

In the event of a potential USM, the investigator must contact the Astellas Study Physician and/or – unique for JP region – Astellas team member (within 24 hrs of awareness). Full details of the potential USM are to be recorded in the subject's medical records. The sponsor may request additional information related to the event to support their evaluation.

If the event is confirmed to be an USM the sponsor will take appropriate action to ensure the safety and welfare of the patients. These actions may include but are not limited to a change in study procedures or study treatment, halting further enrollment in the study, or stopping the study in its entirety. The sponsor or sponsor's designee will notify CA and cEC within the timelines required per current local regulations, and will inform the investigators as required. When required, investigators must notify their IRB/IEC within timelines set by regional regulations.

5.6 Test Drug Concentration

Serum concentrations of zolbetuximab will be measured. Samples will be collected as outlined in the Schedule of Assessments [Table 1]. Blood sampling, processing, storage and shipment instructions will be provided in the Laboratory Manual. Samples will be shipped to and analyzed by a sponsor-designated analytical laboratory using validated analytical methods. Please refer to the Laboratory Manual for more detailed information.

Samples remaining after pharmacokinetic assessments may be used for additional biomarker analysis described in [Section 5.7.1 Biomarkers].

5.7 Other Measurements, Assessments or Methods

5.7.1 Biomarkers

Tumor tissue and blood/serum/plasma samples described in [Sections 5.7.2 Blood/Serum/ Plasma Samples and 5.7.3 Tumor Tissue Samples] may be used for research purposes as allowed per local policy to identify genomic and/or other biomarkers that may be associated with clinical outcome or dynamic changes associated with zolbetuximab treatment (in terms of dose, safety, tolerability and efficacy). Since the identification of exploratory biomarkers that correlate with the efficacy or safety of zolbetuximab treatment may continue to evolve as new findings becomes available, additional analyses related to zolbetuximab activity on tumor signaling pathways or clinical outcomes may be conducted as allowed per local policy. Tumor tissue and blood/serum samples remaining after the specified biomarker assessments (e.g., aliquots of tumor cell RNA or DNA) may be used for re-testing, additional analyses as

defined above or developing, and validating assays related to prediction of response or dynamic changes associated with zolbetuximab treatment. The tumor tissue and blood/serum/plasma samples (e.g., aliquots of tumor cell RNA or DNA, peripheral blood mononuclear cells) will be stored at the study sponsors' facility or a contract laboratory facility for up to 15 years after database closure, at which time the samples will be destroyed. The procedures for the collection, handling and shipping of laboratory samples being submitted to the central laboratory will be specified in a laboratory manual.

5.7.2 Blood, Serum and Plasma Samples

Blood, serum and plasma samples will be collected as allowed per local policy according to the Schedule of Assessments [Table 1] for exploratory biomarker measurements. Blood, serum and plasma samples may be analyzed for biomarkers including but not limited to chemokines, cytokines, CDC activation, circulating DNA soluble factors and genetic markers.

5.7.3 Tumor Tissue Samples

FFPE tumor tissue samples will be obtained for all subjects and sent for central IHC testing to evaluate for CLDN18.2, and HER2 status if a previously documented HER2 test result is not available. Tissue from the primary site is preferred, however, if a metastatic site is used (excluding bone metastasis), the sample should be gastric or GEJ in origin. Archival tumor tissue is preferred, but if the specimen is insufficient or unavailable, a biopsy may be performed to obtain a primary tumor tissue or tumor tissue from metastatic site (excluding bone metastasis). Sponsor preapproval is required when a biopsy procedure is needed for the sole purpose of determining study eligibility. Optional post-progression tumor tissue sample for exploratory biomarker analysis may be collected as allowed per local policy following IRC confirmation of disease progression and prior to initiation of subsequent anticancer therapy for subjects who sign a separate ICF. Tumor specimens may be analyzed for exploratory biomarkers including but not limited to CLDN18.2 expression, immune cells, genetic markers and gene/protein expression, as allowed per local policy.

The investigator, in consultation with other specialists, as needed (e.g., radiology staff) will assess the risk associated with obtaining a tumor tissue sample and determine if the subject is an appropriate candidate for the procedure. Biopsies should be obtained in accordance with institutional policies/guidelines to minimize risk. Procedures requiring general anesthesia should not be performed to obtain a tumor tissue sample; however, if a surgical procedure under general anesthesia is performed for a clinical indication, excess tumor tissue may be used for research purposes with the consent of the subject.

Tumor Tissue Requirements

Visit	Tumor Tissue Requirement
Screening	A minimum of 1 FFPE tumor tissue block (preferred) OR a minimum* of 15 FFPE unstained slides are required as allowed per local policy.<br>If local HER2 results are available, a minimum* of 12 slides are required along with the pathology report/documented test results. If local HER2 results are unavailable, follow guidance above.
Post-Progression (optional)	A minimum of 1 FFPE tumor tissue block (preferred) OR a minimum* of 15 FFPE unstained slides are required as allowed per local policy.

FFPE: formalin-fixed paraffin embedded; HER2: human epidermal growth factor receptor 2

*If the required minimum number of slides is not able to be submitted, sponsor notification and approval is required.

5.7.4 Immunogenicity Assessment (Anti-drug Antibody)

Serum samples to assess the formation of anti-drug antibodies (ADAs) against zolbetuximab will be collected as outlined in the Schedule of Assessments [Table 1]. Blood sampling, processing, storage and shipment instructions will be provided in the Laboratory Manual. Samples will be shipped to and analyzed by a sponsor designated analytical laboratory using validated analytical methods. Please refer to the Laboratory Manual for more detailed information.

Samples remaining after immunogenicity assessments may be used for additional biomarker analysis as allowed per local policy and as described in [Section 5.7.1 Biomarkers].

5.7.5 Optional Samples for Banked Pharmacogenomics Sample Analysis

For subjects who signed a separate ICF, an optional whole blood sample for pharmacogenomics (PGx) will be collected at C1D1 prior to first study drug administration, as allowed per local policy. PGx research may be conducted in the future to analyze or determine genes of relevance to clinical response, pharmacokinetics and toxicity/safety issues. A sample of whole blood for possible retrospective PGx analysis will be collected and processed as allowed per local policy. Blood sampling, processing, storage and shipment instructions will be provided in the Laboratory Manual. Samples will be shipped to a sponsor-designated analytical storage laboratory. Please refer to the Laboratory Manual for more detailed information.

See [Appendix 12.7 Pharmacogenomic Analysis With Banked Sample] for further details on the banking procedures.

5.7.6 Electronic Clinical Outcome Assessments

Subjects will be asked to complete HRQoL and HRU questionnaires as specified in the Schedule of Assessments [Table 1]. The electronic Clinical Outcomes Assessment (eCOA) questionnaires should be completed by the subject at Screening (except for HRU), day 1 of each cycle (or up to 48 hours) before any antiemetic or drug treatment and before the disease status is discussed with the subject using the electronic device provided. When completion by the subject is not possible, the questionnaires may be administered to the subject by site personnel using the electronic tablet device. For subjects with low literacy or situations where

required translation is not available, please contact the sponsor for further guidance. Assessments will also be collected at study treatment discontinuation and 30 and 90 days post zolbetuximab/placebo treatment. HRQoL and HRU questionnaires are not required at CAPOX dosing visits (if different from zolbetuximab/placebo dosing visit), CAPOX treatment discontinuation, 30 day safety follow-up and 90 day safety follow-up visits. A combined visit can be completed if zolbetuximab/placebo and both components of CAPOX are discontinued on the same day and HRQoL and HRU questionnaires will be required at those combined visits. HRQoL will be measured by EORTC QLQ-C30, QLQ-OG25 plus STO22 Belching subscale, GP and the EQ5D-5L.

5.7.6.1 Quality of Life Questionnaire C30

The EORTC-QLQ-C30 is a 30-item cancer-specific instrument consisting of 5 functional scales (physical, role, emotional, social and cognitive), 9 symptom scales/items (fatigue, nausea/vomiting, general pain, dyspnea, insomnia, appetite loss, constipation, diarrhea, and financial difficulties) and a global health status scale. For functional scales, higher scores indicate better functioning, while for symptom scales/items, higher scores indicate worse symptoms.

5.7.6.2 Oesophago-Gastric Module 25 plus STO22 Belching Subscale

The EORTC QLQ-OG25 questionnaire is a 25-item instrument that evaluates gastric and GEJ cancer-specific symptoms. This module consists of 6 scales: dysphagia (3 items), eating restrictions (4 items), reflux (2 items), odynophagia (2 items), pain and discomfort (2 items) and anxiety (2 items), as well as 10 single items: eating in front of others, dry mouth, trouble with taste, body image, trouble swallowing saliva, choked when swallowing, trouble with coughing, trouble talking, weight loss and hair loss. For symptom scales/items, higher scores indicate worse symptoms. To ensure relevant symptoms are adequately covered, 2 items from the EORTC-QLQ-STO22 instrument related to belching and bile or acid coming in mouth will be asked following the EORTC QLQ-OG25 questionnaire.

5.7.6.3 Global Pain

The GP instrument is a single assessment of overall pain.

5.7.6.4 EuroQOL Five Dimensions Questionnaire

The EQ-5D-5L is a standardized instrument for use as a measure of health outcome consisting of 6 items that cover 5 main domains (mobility, self-care, usual activities, pain/discomfort, and anxiety/depression) and a general visual analog scale for health status.

5.7.6.5 Health Resource Utilization

The HRU questionnaire is used to assess the number of office visits, hospital stays, and other healthcare resource utilization that occur outside of the clinical study.

5.8 Total Amount of Blood

The total amount of blood collected for study assessments for each subject will vary depending on how long they stay on treatment.

At any time during the study, if any laboratory abnormalities are found for a subject or for disease assessment, additional blood may be drawn for monitoring.

Additional blood beyond standard monitoring that will be drawn for this study will include draws for eligibility assessment, hematology, chemistry, and coagulation at specific study-defined time points, pharmacokinetics, and biomarker sampling.

The maximum amount of blood collected is approximately 40 mL in cycle 1, and less in later cycles.

6 DISCONTINUATION

6.1 Discontinuation of Individual Subject(s) From Study Treatment

A subject who enrolled in the study and for whom study treatment (zolbetuximab/placebo and both components of CAPOX) is permanently discontinued for any reason will be assessed as having met study treatment discontinuation criteria.

As overall survival is the key secondary end-point of the study, all subjects will be followed for survival after meeting study treatment discontinuation criteria unless the subject withdraws consent or is considered lost to follow-up after repeated attempts to contact or if the sponsor discontinues the study. The reason for discontinuation from study treatment must be documented in the subject's medical records.

A subject is free to withdraw from the study treatment and/or study for any reason and at any time without giving reason for doing so and without penalty or prejudice. The investigator is also free to terminate a subject's involvement in the study at any time if the subject's clinical condition warrants it. The subject will be discontinued from study treatment (zolbetuximab/placebo and both components of CAPOX) if any of the following occur:

- Investigator determines it is in the subject's best interest to discontinue study treatment
- Subject develops radiological disease progression per RECIST 1.1 criteria based on assessment by IRC.
  - If the investigator believes that the subject is continuing to derive clinical benefit (asymptomatic and/or without worsening of performance status or overall health) from study treatment, and an increase in tumor burden is not likely to affect vital organ function, the subject may remain on study treatment until an additional radiologic assessment is completed (≤ 9 weeks from previous radiologic assessment).
    - If the additional radiologic assessment by IRC indicates PD per RECIST 1.1, then the subject must be discontinued from study treatment.
    - If the additional radiologic assessment by IRC does not confirm the initial assessment of PD, the subject may continue to receive study treatment.
- Subject develops clinical progression per investigator assessment and radiologic assessment is not medically feasible to confirm radiologic progression due to the subject's condition.

- Subject starts another systemic chemotherapy, immunotherapy, radiotherapy or other treatment intended for antitumor activity.
- Subject starts another investigational agent or device.
- Subject develops unacceptable toxicity.
- Subject has a delay of zolbetuximab/placebo and both components of CAPOX treatment for > 28 days from when the next zolbetuximab/placebo and both components of CAPOX treatment was scheduled to begin (> 49 days from when the last dose of zolbetuximab/placebo and both components of CAPOX treatment began).
- Any clinical AE, laboratory abnormality, or inter-current illness, in the opinion of the investigator, indicates continued treatment is not in the best interest of the subject
- Female subject becomes pregnant.
- Significant deviation from the protocol or eligibility criteria as determined by the sponsor.
- Subject declines further treatment.
- Subject is lost to follow-up despite reasonable efforts by the investigator to locate the subject.
- Subject is noncompliant with the protocol based on investigator or Medical Monitor assessment.

Note, if a subject discontinues both components of CAPOX and zolbetuximab/placebo due to any reason other than IRC confirmed disease progression (and is not receiving any other anticancer therapy), the subject must be followed according to the protocol-specified radiologic assessment schedule until radiological disease progression per RECIST 1.1 criteria is confirmed by IRC assessment.

Study Discontinuation Criteria

All subjects should remain in the study through the Survival Follow-up Period (OS is a key secondary study endpoint). A subject will be discontinued from the Post-treatment, Long-term and Survival Follow-up Periods if any of the following occur:

- Subject specifically withdraws consent for any further contact with him/her or persons previously authorized by the participant to provide this information
- Subject is lost to follow-up despite reasonable efforts by the investigator to locate the subject
- Death (from any cause)
- Study termination by the sponsor

6.1.1 Lost to Follow Up

Every reasonable effort is to be made to contact any subject lost to follow-up during the course of the study to complete study-related assessments, record outstanding data, and retrieve study drug.

6.2 Discontinuation of the Site

If an investigator intends to discontinue participation in the study, the investigator must immediately inform the sponsor and, ***SPECIFIC TO SITES IN JAPAN***, the head of the study site must also be informed immediately.

6.3 Discontinuation of the Study

If the study is prematurely terminated or suspended the sponsor or designee shall promptly inform the investigators, the IECs/IRBs, the regulatory authorities, and any contract research organization(s) used in the study of the reason for termination or suspension, as specified by the applicable regulatory requirements.

The study may also be terminated if it is not possible for the sponsor to make a necessary adjustment to the maximum insurance sum as required by local law/regulation.

In case of premature study termination, zolbetuximab may be made available to subjects who are still receiving and benefitting from study treatment until a study-defined treatment discontinuation criterion is met.

7 STATISTICAL METHODOLOGY

A statistical analysis plan (SAP) will be written to provide details of the analysis, along with specifications for tables, listings and figures to be produced The final SAP will be approved prior to database hardlock and unblinding the subject treatment assignment. Changes that affected the statistical analyses from the planned analyses in the SAP will be documented in the Clinical Study Report (CSR).

In general, continuous data will be summarized with descriptive statistics (number of subjects, mean, standard deviation, minimum, median and maximum) for continuous variables, and frequency and percentage for categorical variables.

Baseline will be defined as the last observation prior to first dose, unless otherwise specified.

7.1 Sample Size

Approximately 500 subjects will be randomized in a 1:1 ratio to receive zolbetuximab in combination with CAPOX chemotherapy (Arm A) or placebo in combination with CAPOX chemotherapy (Arm B). The planned 300 PFS events during the study will provide 93.4% power to detect a difference in PFS between Arm A (zolbetuximab+CAPOX) with the assumption of 9 months median PFS time and Arm B (placebo+CAPOX) with the assumption of 6 months median PFS time (hazard ratio = 0.67) at the overall 1-sided 0.025 significance level. Similarly, the planned 386 OS events during the study will provide 80% power to detect a difference in OS between Arm A (zolbetuximab+CAPOX) with the assumption of 14.7 months median survival time and Arm B (placebo+CAPOX) with the assumption of 11 months median OS time (hazard ratio = 0.75) at the overall 1-sided 0.025 significance level.

7.2 Analysis Sets

The allocation of subjects to analysis sets will be determined prior to database hard-lock. For each treatment group, the number and percentage of subjects will be characterized for all randomized subjects and by each analysis set.

7.2.1 Full Analysis Set

The full analysis set (FAS) will consist of all subjects who are randomized to 1 of the treatment arms. Subjects would be analyzed according to the treatment they were randomized to. The FAS would be used for description of baseline characteristics and all efficacy analyses.

7.2.2 Safety Analysis Set

The safety analysis set (SAF) will consist of all subjects who received at least 1 dose of any study drug (zolbetuximab/placebo/CAPOX). The SAF will be used for all safety analyses. Subjects would be analyzed according to the actual treatment they received.

7.2.3 Pharmacokinetic Analysis Set (PKAS)

The pharmacokinetic analysis set (PKAS) consists of the subset of the SAF for which at least 1 concentration data is available. Additional subjects may be excluded from the PKAS at the discretion of the pharmacokineticist. The PKAS would be used for description of pharmacokinetic data.

7.3 Demographics and Baseline Characteristics

Demographics and other baseline characteristics will be summarized by treatment group and overall for FAS and SAF.

7.3.1 Subject Disposition

The number and percentage of subjects who discontinued treatment and reasons for treatment discontinuation will be presented for FAS by treatment group and overall. Similar tables for subjects who do not have a PFS event and subjects who do not have observed death will also be presented. All disposition details and dates of first and last evaluations for each subject will be listed.

7.3.2 Previous and Concomitant Medications

All previous and concomitant medications will be presented in a listing. The frequency of concomitant medications (prescription, over-the-counter, and nutritional supplements) will be summarized. Any component of CAPOX is not considered concomitant medications.

7.3.3 Medical History

Medical history for each subject will be presented in a listing and summarized.

7.4 Analysis of Efficacy

Efficacy analysis will be conducted on the FAS. The interpretation of results from statistical tests will be primarily based on the FAS. All randomized subjects will be analyzed according to the treatment to which they are randomized.

7.4.1 Analysis of Primary Endpoint

7.4.1.1 Primary Analysis

The primary endpoint is PFS assessed by the blinded IRC. For each subject, PFS is defined as the time from the date of randomization until the date of radiological disease progression (i.e., PFS) assessed by IRC, or until death due to any cause, whichever is earlier. If a subject has neither progressed nor died, the subject will be censored at the date of last radiological assessment. Subjects who receive new anticancer therapy before radiological progression will be censored at the date of the last radiological assessment before the new anticancer therapy started. If progression or death occurs after missing 2 or more scheduled radiological assessments, the subject will be censored at the date of last radiological assessment or at the date of randomization if no post-baseline radiological assessment is available.

The primary analysis will be performed when approximately 300 PFS events have been observed.

The distribution of PFS will be estimated for each treatment arm using Kaplan-Meier methodology and compared between Arm A and Arm B using log-rank test stratified by:

- Region (Asia vs Non-Asia)
- Number of Organs with Metastatic Sites (0 to 2 vs ≥ 3)
- Prior Gastrectomy (Yes or No)

The hypothesis testing on the primary analysis will be performed at an overall 1-sided 0.025 significance level to test the null hypothesis that PFS is not prolonged in Arm A compared to Arm B versus the alternative hypothesis that PFS is prolonged in Arm A compared to Arm B.

In addition, stratified Cox proportional hazard model will be used to estimate the hazard ratio and the corresponding 95% CI.

The primary analysis will be performed using the FAS.

7.4.1.2 Sensitivity Analysis

Sensitivity analyses with different censoring rules will be described in the SAP.

7.4.1.3 Subgroup Analysis

The analysis described in [Section 7.4.1.1 Primary Analysis] will be conducted in the subgroups using the FAS. Subgroups defined by baseline factors will be described in the SAP.

Forest plot will be presented to illustrate the strength of treatment effects across subgroups.

7.4.2 Analysis of Secondary Endpoints

7.4.2.1 Overall Survival

A key secondary endpoint OS is defined as the time from the date of randomization until the documented date of death from any cause. All events of death will be included, regardless of whether the event occurred while the subject is still taking study drug or after the subject discontinue study drug. Subjects who are still alive at the time of analysis will be censored at the last day known to be alive.

The distribution of OS will be estimated for each treatment arm using Kaplan-Meier methodology and compared between Arm A and Arm B using the log-rank test stratified by the same stratification factors used for PFS analysis. To maintain the overall Type I error rate at the 0.025 significance level, the hypothesis testing on OS will be performed only if the null hypothesis on the primary analysis is rejected at the overall 1-sided 0.025 significance level. In addition, stratified Cox proportional hazard model will be used to estimate the hazard ratio and the corresponding 95% CI.

7.4.2.2 PF, OG25-Pain Plus STO22, and GHS/QoL Scores

A key secondary endpoint, TTCD, is defined for the following 3 HRQoL domains: physical functioning (PF) and Global Health Status/Quality of Life (GHS/QoL) as collected in the EORTC QLQ-C30, and abdominal pain and discomfort (OG25-Pain plus STO22) as collected in the EORTC QLQ-OG25 plus STO22). TTCD is defined as the time from the date of randomization until the date of first clinically meaningful deterioration that is confirmed at a next scheduled assessment or followed by drop-out resulting in missing data.

Clinically meaningful deterioration will be defined if a subject’s change from baseline exceeds a pre-specified threshold denoting a clinically meaningful change. While clinically meaningful thresholds have been derived for EORTC QLQ-30 for some domains (e.g., 10 for GHS/QoL in Osoba 1998), an appropriate threshold for this population and for all domains of interest will be derived using the study’s data. A separate analysis plan will be developed where methods for the estimation of the clinically meaningful threshold will be defined in detail and signed off before database lock. The execution of these analysis and derivation of the threshold value to be used in the TTCD analysis will also be performed before database lock, and the resulting value will be inserted in the clinical SAP in a last amendment again before the study is unblinded.

Subjects who did not experience a confirmed deterioration will be censored at the last eCOA assessment. Subjects who have no baseline or post-baseline assessment, or are not able to show deterioration due to their baseline value will be censored at randomization. For example, if a subject has a baseline GHS/QoL value of 5 and the clinically meaningful threshold proves to be 8, then this subject will be censored at baseline. Death or disease progression will not be considered as an event.

Similarly to PFS and OS endpoints, the distribution of TTCD will be estimated for each treatment arm using Kaplan-Meier methodology and compared between Arm A and Arm B using the log-rank test stratified by the same stratification factors used for PFS and OS

analysis. To maintain the overall Type I error rate at the 0.025 significance level, the hypothesis testing on TTCD will be performed only if the null hypothesis on the OS is rejected at the overall 1-sided 0.025 significance level. Specifically, if the null hypothesis on the OS is rejected at the overall 1-sided 0.025 significance level, then the TTCD will be tested using the gatekeeping procedure with the following order:

1. Non-inferiority testing for TTCD in PF at 0.025 significance level
2. Non-inferiority testing for TTCD in OG25-Pain plus STO22 at 0.025 significance level
3. Non-inferiority testing for TTCD in GHS/QoL at 0.025 significance level
4. Superiority testing for TTCD in PF at 0.025 significance level
5. Superiority testing for TTCD in OG25-Pain plus STO22 at 0.025 significance level
6. Superiority testing for TTCD in GHS/QoL at 0.025 significance level

A stratified Cox proportional hazard model will be used to estimate the hazard ratio and the corresponding 95% CI for the three time-to-event eCOA endpoints..

7.4.2.3 Objective Response Rate

Best overall response (BOR) is determined once all tumor response data for the subject is available. Subjects will be classified by BOR on study as outlined in RECIST 1.1 criteria. For BOR of SD, SD must be documented as present at least once after study entry and at least 8 weeks after first dose.

The ORR is defined as the proportion of subjects with a BOR of CR or PR based on IRC per RECIST V1.1.

The comparison of ORR between Arm A and Arm B will be performed using stratified Cochran-Mantel-Haenszel (CMH) test with the same stratification factor used for the PFS analysis. In addition, ORR for each arm will be estimated and corresponding 95% CI will be constructed.

In addition, percent of subjects with CR will be summarized.

7.4.2.4 Duration of Response

DOR is defined as the time from the date of the first response of CR or PR (whichever is first recorded) as assessed by IRC to the date of radiological progression or death, whichever is earlier. If a subject has not progressed, the subject will be censored at the date of last radiological assessment or at the date of first CR/PR if no post-baseline radiological assessment is available. Other censoring used for the PFS analysis will apply to DOR too.

The distribution of DOR will be estimated for each treatment arm using Kaplan-Meier methodology and compared between Arm A and Arm B using the log-rank test stratified by the same stratification factors used for the PFS analysis. In addition, stratified Cox proportional hazard model will be used to estimate the hazard ratio and the corresponding 95% CI.

7.4.2.5 Health-Related Quality of Life

HRQoL endpoints will be summarized by descriptive statistics with respect to change from baseline for the FAS for each treatment arm. Completion rate for each questionnaire will be summarized by time point. Additional analyses will be described in the SAP.

7.4.3 Analysis of Exploratory Endpoints

7.4.3.1 Time to Progression

TTP is defined as the time from the date of randomization until the date of PD (per RECIST 1.1 by IRC). TTP does not include deaths as event. For deaths before the first documented PD by IRC, subjects will be censored at the time of last radiological assessment. Kaplan-Meier and log-rank methods will be applied to TTP endpoint.

7.4.3.2 Progression Free Survival After Subsequent Therapy (PFS2)

PFS2 is defined as the time from the date of randomization until the date of PD (per investigator) following subsequent anticancer therapy, death from any cause or start of any other anticancer therapy, whichever is earliest. In cases where PFS2 cannot be reliably determined, discontinuation of subsequent anticancer treatment may be used as the event date. Otherwise, subjects will be censored. Subjects who are alive and for whom a PFS2 event date has not been observed should be censored at the last time known to be alive and without second objective disease progression.

The distribution of PFS2 will be estimated for each treatment arm using Kaplan-Meier methodology and compared between Arm A and Arm B using stratified log-rank test with the same stratification factors used for the PFS analysis. In addition, stratified Cox proportional hazard model will be used to estimate the hazard ratio and the corresponding 95% CI.

7.4.3.3 Disease Control Rate

The DCR is defined as the proportion of subjects with a BOR of CR, PR or SD based on RECIST 1.1 by IRC.

The comparison of DCR between Arm A and Arm B will be performed using CMH test with the same stratification factor used for PFS analysis. In addition, DCR for each arm will be estimated and corresponding 95% CI will be constructed.

7.4.3.4 Biomarkers

Biomarkers may be summarized graphically or descriptively, and summary statistics may be tabulated. Associations between biomarkers and clinical (e.g., efficacy, safety or pharmacodynamics, pharmacokinetics) measures may be performed on subjects who have sufficient baseline and on-study measurements to provide interpretable results for specific parameters. Additional post-hoc statistical analyses may be outlined in the SAP.

7.4.3.5 Health Resource Utilization

HRU variables will be summarized for each treatment arm.

7.5 Analysis of Safety

All treated subjects will be analyzed according to the treatment they received.

7.5.1 Adverse Events

AEs will be coded using the Medical Dictionary for Regulatory Activities (MedDRA) and graded using NCI-CTCAE v4.03.

Treatment-emergent adverse event (TEAE) is defined as an AE observed after starting administration of the study treatment and within 30 days after the last dose of study treatment. Late SAE and/or AE is defined as (S)AE that is collected after 30 days post last dose of study drug.

A study drug-related TEAE is defined as any TEAE with a causal relationship of YES by the investigator.

AEs of special interest described in [Section 5.5.8 Adverse Events of Special Interest] will be summarized.

The number and percentage of AEs, SAEs, AEs leading to interruption/discontinuation, AEs leading to death and AEs related to study drug will be summarized by SOC, preferred term (PT) for SAF. The number and percentage of AEs by toxicity grade will also be summarized. Late (S)AEs will be summarized. All AEs will be listed.

7.5.2 Laboratory Assessments

For quantitative laboratory measurements descriptive statistics will be used to summarize results and change from baseline for subjects in the SAF by treatment group and time point.

Shifts from baseline to the worst grade based on NCI-CTCAE v4.03 in laboratory tests will also be tabulated.

7.5.3 Vital Signs

Descriptive statistics will be used to summarize vital sign results and changes from baseline for subjects in the SAF by treatment group and time point.

7.5.4 Routine 12-lead Electrocardiograms

The 12-lead ECG results will be summarized by treatment group and time point.

7.5.5 Eastern Cooperative Oncology Group Performance Status

Summary statistics (number and percent of subjects) for each category of the ECOG performance status at each assessment will be provided. The change from baseline to final visit or early termination will also be summarized. Negative change scores indicate an improvement. Positive scores indicate a decline in performance.

7.6 Analysis of Pharmacokinetics

Descriptive statistics will include the number of subjects (n), mean, standard deviation, minimum, median, maximum, coefficient of variation (CV), geometric mean and geometric CV.

7.6.1 Serum Concentrations

Serum concentrations of zolbetuximab will be listed and summarized using descriptive statistics by scheduled time point. Box and whisker plots of trough concentrations against cycle and dosing day will be provided.

7.6.2 Immunogenicity

Immunogenicity of zolbetuximab will be summarized using the frequency of ADA positive subjects. The potential relationship between zolbetuximab immunogenicity and zolbetuximab pharmacokinetics, efficacy, safety profile in subjects may be assessed.

Additional model-based analyses may be performed and reported separately for zolbetuximab pharmacokinetics.

7.7 Major Protocol Deviations and Other Analyses

Major protocol deviations as defined in [Section 8.2 Major Protocol Deviations] will be summarized for all randomized subjects by treatment group and total as well as by site. A data listing will be provided by site and subject.

The major protocol deviation criteria will be uniquely identified in the summary table and listing.

7.8 Interim Analysis (and Early Discontinuation of the Clinical Study)

To evaluate whether zolbetuximab + CAPOX (Arm A) is beneficial compared to placebo + CAPOX (Arm B) while the study is ongoing, a formal OS interim analysis is planned when the final PFS analysis occurs with the pre-specified number of PFS events. A group sequential design using the O'Brien-Fleming type alpha-spending function [Lan & DeMets, 1983] for efficacy will be utilized to control the overall 1-sided 0.025 significance level (East®) for the OS analyses.

The IDMC may recommend terminating the study for favorable results at the formal efficacy interim analysis using OS. In the case of favorable results, the 1-sided significance level for superiority is 0.0074, assuming about 70% of the target number of OS events is obtained, for the interim OS analysis and 0.0228 for the final OS analysis (Note: The OS significance level will be adjusted depending on the number of OS events at the time of interim analysis). If the 1-sided P value of the interim analysis is less than the significance level (and PFS is also significant at 1-sided 0.025 alpha), the IDMC may recommend terminating the study for success. If the study is not stopped after the interim analysis, a final OS analysis will occur after 100% of the planned death events have been observed.

The interim OS analysis will be conducted by an Independent Data Analysis Center for IDMC. In addition, safety data reviews during the study will be conducted by the IDMC on a periodic basis. For example, the IDMC will have its first safety data review 6 weeks after the 40th subject has been randomized and on study drug for 2 cycles (6 weeks) and meetings will be conducted regularly thereafter, as determined by the IDMC.

The full procedures for IDMC safety review will be described in a separate IDMC Charter.

The analysis for the eCOA endpoints will be performed as the final analysis once OS is significant either at the interim OR or at final OS anlaysis hence there is no interim analysis planned for eCOA endpoints.

7.9 Handling of Missing Data, Outliers, Visit Windows, and Other Information

Imputation for missing data, if applicable, will be addressed in the SAP.

8 OPERATIONAL CONSIDERATIONS

8.1 Procedure for Clinical Study Quality Control

8.1.1 Data Collection

The investigator or site designee will enter data collected using an electronic data capture system. In the interest of collecting data in the most efficient manner, the investigator or site designee should record data (including laboratory values, if applicable) in the eCRF within 5 days after the subject visit.

The investigator must maintain accurate documentation (source data) that supports the information entered in the eCRF. These documents should be appropriately maintained by the site.

Local laboratory results may be used for treatment decisions; however, central laboratory samples must also be drawn per protocol and sent to the central laboratory unless otherwise approved by sponsor. Central Laboratory data will be transferred electronically to the sponsor or designee at predefined intervals during the study. The central laboratory will provide the sponsor or designee with a complete and clean copy of the data.

Imaging results are read by a central imaging laboratory. Central imaging data will be transferred electronically to the sponsor or designee at predefined intervals during the study. The central imaging laboratory will provide the sponsor or designee with a complete and clean copy of the data.

For Screen failures the demographic data, reason for failing, informed consent, inclusion and exclusion criteria and AEs will be collected in the eCRF.

8.1.1.1 Collection of Data Via Electronic Source

All procedures conducted under the protocol must be documented. For screen failures, the minimum demographic data (sex, birth date, race and informed consent date), outcome of

eligibility assessment (inclusion and exclusion criteria), reason for screen failure and AEs details must be documented.

The investigator or designee will be responsible for eCRF completion and for ensuring the source data are attributable, legible, contemporaneous, original, accurate and complete whether the data are handwritten on paper or entered electronically. If source data are created (first entered), modified, maintained, archived, retrieved or transmitted electronically via computerized systems (and/or other kind of electronic devices) as part of regulated study activities, such systems must be compliant with all applicable laws and regulations governing use of electronic records and/or electronic signatures. Such systems may include, but are not limited to, electronic medical/health records, protocol-related assessments, AE tracking, electronic clinical outcome assessment and/or drug accountability.

Electronic data sources and any supporting documents should be available for review/retrieval by the sponsor/designee at any given time.

Study monitors will perform ongoing source data review to confirm that the safety and rights of participants are being protected; and that the study is being conducted in accordance with the currently approved protocol and any other study agreements, ICH GCP, and all applicable regulatory requirements.

8.1.1.2 Electronic Clinical Outcomes Assessment

eCOA assessments will be performed according to the Schedule of Assessments [Table 1]. Subject HRQoL and HRU questionnaires will be completed by the subject or, if not possible, administered to the subject by site personnel on an electronic tablet device for subject visits. The subject questionnaire responses captured on the electronic device will be transferred to the eCOA vendor’s central portal (web portal). The investigator or site designee should review the questionnaire data on the web portal for correct completion while the subject is at the site. The questionnaire data will be transferred electronically to sponsor or designee at predefined intervals during the study. Sponsor/CRO staff also have access to the vendor web portal for continuous review of the data and access to reports. The vendor will provide sponsor or designee with a complete and clean copy of the data.

For further details please refer to the eCOA guidelines/manual (e.g., ERT Site Guide to eCOA Product).

8.2 Major Protocol Deviations

A major protocol deviation is generally an unplanned excursion from the protocol that is not implemented or intended as a systematic change. All deviations from the protocol are to be recorded. A protocol waiver is a documented prospective approval of a request from an investigator to deviate from the protocol. Protocol waivers are strictly prohibited.

The investigator is responsible for ensuring the study is conducted in accordance with the procedures and evaluations described in this protocol and must protect the rights, safety, and welfare of subjects. The investigator should not implement any deviation from, or changes of, the protocol, unless it is necessary to eliminate an immediate hazard to study subjects.

When a major deviation from the protocol is identified for an individual subject, the investigator or designee must ensure the sponsor is notified. The sponsor will follow up with the investigator, as applicable, to assess the deviation and the possible impact to the safety of the subject to determine subject continuation in the study.

Major protocol deviation criteria will be summarized at the end of the study.

The investigator will also assure that deviations meeting IRB/IEC and applicable regulatory authorities' criteria are documented and communicated appropriately. All documentation and communications to the IRB/IEC and applicable regulatory authorities will be provided to the sponsor and maintained within the trial master file.

9 END OF TRIAL

The end of the study is defined as the last visit or scheduled procedure shown in the Schedule of Assessments [Table 1] for the last study participant in the study.

Study completion is defined as the conclusion of data collection for the defined study endpoints. The study may be closed within a participating country per local regulations once the study has completed and if all subjects enrolled in the country are no longer receiving study treatment. In addition, the sponsor may prematurely terminate the study for reasonable cause at any time.

10 STUDY ORGANIZATION

10.1 Independent Data Monitoring Committee

An IDMC will evaluate the unblinded safety data of subjects enrolled on a periodic basis during this study. IDMC members will be clinicians with expertise in gastric cancer studies and are not investigators participating in this study or Astellas employees. A separate charter will outline the activities of this committee.

10.2 Other Study Organization

SPECIFIC TO SITES IN JAPAN: The Japan site contact list is kept as a separate attachment to the protocol.

11 REFERENCES

Ajani JA, Rodriguez W, Bodoky G, Moiseyenko V, Lichinitser M, Gorbunova V, et al. Multicenter phase III comparison of cisplatin/S-1 with cisplatin/infusional fluorouracil in advanced gastric or gastroesophageal adenocarcinoma study: the FLAGS trial. J Clin Oncol. 2010 Mar;28(9):1547-53.

Amiri M, Janssen F, Kunst AE. The decline in stomach cancer mortality: exploration of future trends in seven European countries. Eur J Epidermiol. 2011;26:23-8.

Bang Y, Chung H, Xu J, Lordick F, Sawaki A, Al-Sakaff N, et al. Pathological features of advanced gastric cancer (GC): Relationship to human epidermal growth factor receptor 2 (HER2) positivity in the global screening programme of the ToGA trial. J Clin Oncol. 2009;27(155):4556.

Bang YJ, Van Cutsem E, Feyereislova A, Chung HC, Shen L, Sawaki A. Trastuzumab in combination with chemotherapy versus chemotherapy alone for treatment of HER2-positive advanced gastric or gastro-oesophageal junction cancer (ToGA): a phase 3, open-label, randomised controlled trial. Lancet 2010;376:687-97.

Cancer Fact Sheet. World Health Organization; February 2018.

CYRAMZA Prescribing Information. Indianapolis, IN: Eli Lilly and Company; 2017.

Eisenhauer EA, Therasse P, Bogaerts J, et al. New response evaluation criteria in solid tumours: revised RECIST guideline (version 1.1). Eur J Cancer 2009;45:228-247.

Fuchs CS, Tomasek J, Yong CJ, Dumitru F, Passalacqua R, Goswami C, et al. Ramucirumab monotherapy for previously treated advanced gastric or gastro-oesophageal junction adenocarcinoma (REGARD): an international, randomised, multicentre, placebo-controlled, phase 3 trial. Lancet. 2014;383:31-39.

Gunzel D, Yu SL. Claudins of the modulation of tight junction permeability. Physiol. Rev. 2013;93(2): 525-69.

HERCEPTIN (trastuzumab) [package insert]. South San Francisco, CA: Genentech, Inc.; 2016.

Karanjawala ZE, Illei PB, Ashfaq R, Infante JR, Murphy K, Pandey A, et al. New markers of pancreatic cancer identified through differential gene expression analyses: claudin 18 and annexin A8. Am J Surg Pathol. 2008;32:188-196.

Kim GM, Jeung HC, Rha SY, Kim HS, Jung I, Nam BH, et al. A randomized phase II trial of S-1-oxaliplatin versus capecitabine-oxaliplatin in advanced gastric cancer. Eur J Cancer. 2012 Mar;48(4):518-26.

Kim NK, Park YS, Heo DS, Suh C, Kim SY, Park KC, et al. A phase III randomized study of 5-fluorouracil and cisplatin versus 5-fluorouracil, doxorubicin and mitomycin C versus 5-fluorouracil alone in the treatment of advanced gastric cancer. Cancer. 1993 Jun;71(12):3813–18.

Lan KKG and De Mets DL. Discrete sequential boundaries for clinical trials. Biometrika. 1983;70:659-663.

Lederman L. Gastric Cancer: local and global burden. Supplement: the current and future management of gastric cancer. Am J Manag Care. 2017:2-5.

Lee JH, Kim KS, Kim TJ, Hong SP, Song SY, Chung JB, et al. Immunohistochemical analysis of claudin expression in pancreatic cystic tumors. Oncol Rep. 2011;25:971-978.

National Comprehensive Cancer Network Clinical Practice Guidelines in Oncology (NCCN Guidelines): Gastric cancer. National Comprehensive Cancer Network (NCCN). 2017; version 1.2017.

Niimi T, Nagashima K, Ward JM, Minoo P, Zimonjic DB, Popescu NC, et al. claudin-18, a novel downstream target gene for the T/EBP/NKX2.1 homeodomain transcription factor, encodes lung- and stomach-specific isoforms through alternative splicing. Mol Cell Biol. 2001;21(21):7380-7390.

Ohtsu A, Shah MA, Van Cutsem E, Rha SY, Sawaki A, Park SR, et al. Bevacizumab in combination with chemotherapy as first-line therapy in advanced gastric cancer: a randomized, double-blind, placebo-controlled phase III study. J Clin Oncol. 2011;29:3968-3976.

Oken MM, Creech RH, Tormey DC, Horton J, Davis TE, McFadden ET, et al. Toxicity and response criteria of the Eastern Cooperative Oncology Group. Am J Clin Oncol. 1982;5(6):649-55.

Osoba D, Rodrigues G, Myles J, Zee B, Pater J. Interpreting the significance of changes in health-related quality-of-life scores. J Clin Oncol.1998;16(1):139-44.

Pasini F, Fraccon AP, De Manzoni G. The role of chemotherapy in metastatic gastric cancer. Cancer Res. 2011;31:3543-3554.

Pennathur A, Gibson MK, Jobe BA, Luketich JD. Oesophageal carcinoma. Lancet. 2013;381:400-12.

Sahin U, Koslowski M, Dhaene K, Usener D, Brandenburg G, Seitz G, et al. Claudin-18 splice variant 2 is a pan-cancer target suitable for therapeutic antibody development. Clin Cancer Res. 2008;14(23):7624-34.

Sanada Y, Hirose Y, Osada S, Tanaka Y, Takahashi T, Yamaguchi K, et al. Immunohistochemical study of claudin 18 involvement in intestinal differentiation during the progression of intraductal papillary mucinous neoplasm. Anticancer Res. 2010;30:2995-3003.

Shah MA, Bang YJ, Lordick F, Alsina M, Chen M, Hack SP, et al. Effect of Fluorouracil, Leucovorin, and Oxaliplatin With or Without Onartuzumab in HER2-Negative, MET-Positive Gastroesophageal Adenocarcinoma - The METGastric Randomized Clinical Trial. JAMA Oncol. 2017;3(5):620-7.

Vita FDe, Orditura M, Matano E, Bianco R, Carlomagno C, Infusino S, et al. A phase II study of biweekly oxaliplatin plus infusional 5-fluorouracil and folinic acid (FOLFOX-4) as first-line treatment of advanced gastric cancer patients. Br J Cancer. 2005;92(9):1644-49.

Waddell T, Chau I, Cunningham D, Gonzalez D, Okines AF, Wotherspoon A, et al. Epirubicin, oxaliplatin, and capecitabine with or without panitumumab for patients with previously untreated advanced oesophagogastric cancer (REAL3): a randomized, open-label phase 3 trial. Lancet Oncol. 2013;14(6):481-9.

Wilke H, Muro K, Van Cutsem E, Oh SC, Bodoky G, Shimada Y, et al. Ramucirumab plus paclitaxel versus placebo plus paclitaxel in patients with previously treated advanced gastric or gastro-oesophageal junction adenocarcinoma (RAINBOW): a double-blind, randomised phase 3 trial. Lancet Oncol. 2014;15:1224-35.

Woll S, Schlitter AM, Dhaene K, Roller M, Esposito I, Sahin U, et al. Claudin 18.2 is a target for IMAB362 antibody in pancreatic neoplasms. Int. J. Cancer. 2014;134:731-39.

XELOX CCO Formulary, August 2017.

12 APPENDICES

12.1 Ethical, Regulatory, and Study Oversight Considerations

12.1.1 Ethical Conduct of the Study

The study will be conducted in accordance with the protocol, ICH guidelines, all applicable regulations and guidelines governing clinical study conduct and the ethical principles that have their origin in the Declaration of Helsinki.

12.1.2 Institutional Review Board (IRB)/Independent Ethics Committee (IEC)/Competent Authorities (CA)

GCP requires that the clinical protocol, any protocol amendments, the IB, the informed consent and all other forms of subject information related to the study (e.g., advertisements used to recruit subjects) and any other necessary documents be reviewed by an IEC/IRB. The IEC/IRB will review the ethical, scientific and medical appropriateness of the study before it is conducted. IEC/IRB approval of the protocol, informed consent and subject information and/or advertising, as relevant, will be obtained prior to the authorization of drug shipment to a study site.

Any substantial amendments to the protocol will require IRB/IEC approval before to implementation, except for changes necessary to eliminate an immediate hazard to subjects.

The investigator will be responsible for the following:

- Providing written summaries of the status of the study to the IRB/IEC annually or more frequently in accordance with the requirements, policies, and procedures established by the IRB/IEC
- Notifying the IRB/IEC of SAEs or other significant safety findings as required by IRB/IEC procedures
- Providing oversight of the conduct of the study at the site and adherence to requirements of 21 CFR, ICH guidelines, the IRB/IEC, European regulation 536/2014 for clinical studies (if applicable), and all other applicable local regulations

12.1.3 Protocol Amendment and/or Revision

Any changes to the study that arise after approval of the protocol must be documented as protocol amendments: substantial amendments and/or non-substantial amendments. Depending on the nature of the amendment, either IRB/IEC, Competent Authority approval or notification may be required. The changes will become effective only after the approval of the sponsor, the investigator, the regulatory authority, and the IRB/IEC (if applicable).

Amendments to this protocol must be signed by the sponsor and the investigator. Written verification of IRB/IEC approval will be obtained before any amendment is implemented. Modifications to the protocol that are administrative in nature do not require IRB/IEC approval, but will be submitted to the IRB/IEC for their information, if required by local regulations.

If there are changes to the informed consent, written verification of IRB/IEC approval must be forwarded to the sponsor. An approved copy of the new informed consent must also be forwarded to the sponsor.

12.1.4 Informed Consent of Subjects

12.1.4.1 Subject Information and Consent

The investigator or his/her representative will explain the nature of the study to the subject or his/her guardian or legal representative (if applicable), and answer all questions regarding this study.

An optional partial screening ICF may be available to allow central testing of tissue for CLDN18.2 and HER2 only.

Prior to any study-related screening procedures being performed on the subject, the informed consent statement will be reviewed and signed (***UNIQUE TO JAPAN REGION****:* place a personal seal) and dated by the subject or his/her guardian or legal representative, the person who administered the informed consent and any other signatories according to local requirements. A copy of the signed (***UNIQUE TO JAPAN REGION:*** or sealed) ICF will be given to the subject and the original will be placed in the subject’s medical record. An entry must also be made in the subject’s dated source documents to confirm that informed consent was obtained prior to any study-related procedures and that the subject received a signed copy. The signed consent forms will be retained by the investigator and made available (for review only) to the study monitor and auditor regulatory authorities and other applicable individuals upon request.

12.1.4.2 Supply of New and Important Information Influencing the Subject’s Consent and Revision of the Written Information

1. The investigator or his/her representative will immediately inform the subject orally whenever new information becomes available that may be relevant to the subject’s consent or may influence the subject’s willingness to continue to participate in the study (e.g., report of serious drug adverse drug reaction). The communication must be documented in the subject’s medical records and whether the subject is willing to remain in the study or not must be confirmed and documented.
2. The investigator must update their ICF and submit it for approval to the IRB/IEC. The investigator or his/her representative must obtain written informed consent from the subject on all updated ICFs throughout their participation in the study. The investigator or his/her designee must re-consent subjects with the updated ICF even if relevant information was provided orally. The investigator or his/her representative who obtained the written informed consent and the subject should sign and date the ICF (***UNIQUE TO JAPAN REGION:*** place a personal seal). A copy of the signed (***UNIQUE TO JAPAN REGION:*** or sealed) ICF will be given to the subject and the original will be placed in the subject’s medical record. An entry must be made in the subject’s records documenting the re-consent process.

12.1.5 Source Documents

Source data must be available at the site to document the existence of the study subjects and to substantiate the integrity of study data collected. Source data must include the original documents relating to the study, as well as the medical treatment and medical history of the subject.

The investigator is responsible for ensuring the source data are attributable, legible, contemporaneous, original, accurate and complete whether the data are hand-written on paper or entered electronically. If source data are created (first entered), modified, maintained, achieved, retrieved or transmitted electronically via computerized systems (and/or other kind of electric devices) as part of regulated clinical study activities, such systems must be compliant with all applicable laws and regulations governing use of electronic records and/or electronic signatures. Such systems may include, but are not limited to, electronic medical/health records, protocol related assessments, AE tracking, and/or drug accountability.

Paper records from electronic systems used in place of electronic format must be certified copies. A certified copy must be an exact copy and must have all the same attributes and information as the original. Certified copies must include signature and date of the individual completing the certification. Certified copies must be a complete and chronological set of study records (including notes, attachments, and audit trail information (if applicable). All printed records must be kept in the subject file and available for archive.

12.1.6 Record Retention

The investigator will archive all study data (e.g., subject identification code list, source data, eCRFs and investigator's file) and relevant correspondence. These documents are to be kept on file for the appropriate term determined by local regulation (for US sites, two years after approval of the NDA or discontinuation of the IND). The sponsor will notify the site/investigator if the NDA/MAA/J-NDA is approved or if the IND/IMPD/CHIKEN TODOKE is discontinued. The investigator agrees to obtain the sponsor's agreement prior to disposal, moving, or transferring of any study-related records. The sponsor will archive and retain all documents pertaining to the study according to local regulations.

Data generated by the methods described in the protocol will be recorded in the subjects' medical records and/or study progress notes.

12.1.7 Subject Confidentiality and Privacy

Individual subject medical information obtained as a result of this study is considered confidential and disclosure to third parties is prohibited unless otherwise the subject provides written consent or approval. Additional medical information may be given only after approval of the subject to the investigator or to other appropriate medical personnel responsible for the subject's well-being.

The sponsor shall not disclose any confidential information on subjects obtained during the performance of their duties in the clinical study without justifiable reasons.

Even though any individuals involved in the study, including the study monitors and auditors, may get to know matters related to subject's privacy due to direct access to source documents, or from other sources, they may not leak the content to third parties.

The sponsor affirms the subject's right to protection against invasion of privacy. Only a subject identification number will identify subject data retrieved by the sponsor. However, the sponsor requires the investigator to permit the sponsor, sponsor's representative(s), the IRB/IEC and when necessary, representatives of the regulatory health authorities to review and/or to copy any medical records relevant to the study.

The sponsor agrees to comply and process personal data in accordance with all applicable privacy laws and regulations, including, without limitation, the Personal Information Protection Law in Japan and Privacy laws in the US. If the services will involve the collection or processing of personal data (as defined by applicable data protection legislation) within the European Economic Area (EEA), then sponsor shall serve as the controller of such data, as defined by the European Union (EU) Data Protection Directive, and Investigator and/or third party shall act only under the instructions of the sponsor in regard to personal data If sponsor is not based in the EEA, sponsor must appoint a third party to act as its local data protection representative or arrange for a co-controller established in the EU for data protection purposes in order to comply with the Directive.

12.1.8 Arrangement for Use of Information and Publication of the Clinical Study

Information concerning the study drug, patent applications, processes, unpublished scientific data, the IB and other pertinent information is confidential and remains the property of the sponsor. Details should be disclosed only to the persons involved in the approval or conduct of the study. The investigator may use this information for the purpose of the study only. It is understood by the investigator that the sponsor will use the information obtained during the clinical study in connection with the development of the drug and therefore may disclose it as required to other clinical investigators or to regulatory agencies. In order to allow for the use of the information derived from this clinical study, the investigator understands that he/she has an obligation to provide the sponsor with all data obtained during the study.

Publication of the study results is discussed in the clinical study agreement.

12.1.9 Insurance of Subjects and Others (UNIQUE TO JAPAN REGION/STUDIES ENROLLING SUBJECTS IN EU)

If a subject suffers any study-related injury, the sponsor will compensate appropriately according to the severity and duration of the damage. However, if it was caused intentionally or was due to gross negligence by the study site, the sponsor will consult with the study site about handling the injury, based on the agreed study contract. Compensation for the study-related injury is provided by the following procedures:

1. If a subject incurs an injury as a result of participation in the clinical study, the study site should provide medical treatment and other necessary measures. The sponsor should be notified of the injury.

2. When the subject claims compensation from the study site for the above study-related injury, or such compensation may be claimed, the study site should immediately communicate the fact to the sponsor. Both parties should work together towards compensation settlement.
3. The sponsor shall pay compensation or indemnification and bear expenses necessary for the settlement as provided in the clinical contract.
4. The sponsor shall make an arranging for insurance and take measures necessary to ensure the compensation or indemnification mentioned above.

The sponsor has covered this study by means of an insurance of the study according to national requirements. The name and address of the relevant insurance company, the certificate of insurance, the policy number and the sum insured are provided in the investigator's file.

12.1.10 Signatory Investigator for Clinical Study Report

ICH E3 guidelines recommend and EU Directive 2001/83/EC requires that a final study report, which forms part of a marketing authorization application, be signed by the representative for the coordinating investigator(s) or the principal investigator(s). The representative for the coordinating investigator (s) or the principal investigator(s) will have the responsibility to review the final study results to confirm to the best of his/her knowledge it accurately describes the conduct and results of the study. The representative for coordinating investigator(s) or the principal investigator(s) will be selected from the participating investigators by the sponsor prior to database lock.

12.2 Procedure for Clinical Study Quality Control

12.2.1 Clinical Study Monitoring

The sponsor or delegated CRO is responsible for monitoring the clinical study to ensure that subject's human rights, safety, and well-being are protected, that the study is properly conducted in adherence to the current protocol and GCP, and study data reported by the investigator/sub-investigator are accurate and complete and that they are verifiable with study-related records such as source documents. The sponsor is responsible for assigning study monitor(s) to this study for proper monitoring. They will monitor the study in accordance with planned monitoring procedures.

12.2.2 Direct Access to Source Data/Documents

The investigator and the study site must accept monitoring and auditing by the sponsor or delegated CRO as well as inspections from the IRB/IEC and relevant regulatory authorities. In these instances, they must provide all study-related records, including source documents when they are requested by the sponsor monitors and auditors, the IRB/IEC or regulatory authorities. The confidentiality of the subject's identities shall be well protected consistent with local and national regulations when the source documents are subject to direct access.

Remote source data review will be used when needed. It will focus on the quality control of critical data such as primary efficacy data and important safety data. Important secondary efficacy data will be monitored simultaneously, provided this does not result in a need to access additional documents and therefore increase the burden for site staff. The sponsor will determine the extent and nature of remote source data review that is needed for any exceptional situations and will carefully weigh it against the extra burden that introduction of any alternative measures would put on site staff and facilities.

12.2.3 Data Management

Data Management will be coordinated by the Data Science of the sponsor in accordance with the SOPs for data management. All study-specific processes and definitions will be documented by Data Management. eCRF completion will be described in the eCRF instructions. Coding of medical terms and medications will be performed using MedDRA and World Health Organization (WHO) Drug Dictionary, respectively.

12.2.4 QUALITY ASSURANCE

The sponsor is implementing and maintaining quality assurance and quality control systems with written SOPs to ensure that studies are conducted and data are generated, documented, recorded, and reported in compliance with the protocol, GCP, and applicable regulatory requirement(s). Where applicable, the quality assurance and quality control systems and written SOPs of the CRO will be applied.

The sponsor or sponsor's designee may arrange to audit the clinical study at any or all investigational sites and facilities. The audit may include on-site review of regulatory documents, case report forms, and source documents. Direct access to these documents will be required by the auditors.

12.3 Contraception Requirements

WOCBP participants who choose complete abstinence must continue to have pregnancy tests, as specified in Schedule of Assessments [Table 1].

WOMEN OF CHILDBEARING POTENTIAL DEFINITIONS AND METHODS OF CONTRACEPTION DEFINITIONS (WOCBP)

A woman is considered fertile following menarche and until becoming postmenopausal unless permanently sterile.

Women in the following categories are not considered WOCBP

- Premenarchal
- Premenopausal female with 1 of the following:
  - Documented hysterectomy
  - Documented bilateral salpingectomy
  - Documented bilateral oophorectomy
- Postmenopausal

Documentation of any of these categories can come from the site personnel's review of the female subject's medical records, medical examination, or medical history interview.

A postmenopausal state is defined as at least 12 months after last regular menstrual bleeding without an alternative medical cause.

- In case the last regular menstrual bleeding cannot be clearly determined, confirmation with repeated FSH measurements of at least > 40 IU/L (or higher per local institutional guidelines), is required.

Females on HRT and whose menopausal status is in doubt will be required to use one of the non-estrogen hormonal highly effective contraception methods if they wish to continue their HRT during the study. Otherwise, they must discontinue HRT to allow confirmation of postmenopausal status by repeated FSH measurements before study enrollment.

CONTRACEPTION GUIDANCE FOR FEMALE PARTICIPANTS OF CHILD BEARING POTENTIAL

One of the highly effective methods of contraception listed below is required at the time of informed consent and until the end of relevant systemic exposure, defined as 9 months after the final administration of oxaliplatin and 6 months after the final administration of all other study drugs[a].

Highly Effective Contraceptive Methods (Failure rate of <1% per year when used consistently and correctly)[b]

Combined (estrogen- and progestogen-containing) hormonal contraception associated with inhibition of ovulation

- oral
- intravaginal

- transdermal

Progestogen-only hormonal contraception associated with inhibition of ovulation

- oral
- injectable
- implantable

Hormonal methods of contraception containing a combination of estrogen and progesterone, vaginal ring, injectables, implants and intrauterine hormone-releasing system (IUS)

- intrauterine device (IUD)
- bilateral tubal occlusion

Vasectomized partner *(A vasectomized partner is a highly effective contraception method provided that the partner is the sole male sexual partner of the WOCBP and the absence of sperm has been confirmed. If not, an additional highly effective method of contraception should be used.)*

Sexual abstinence (*Sexual abstinence is considered a highly effective method only if defined as refraining from heterosexual intercourse during the entire period of risk associated with the study drug. The reliability of sexual abstinence needs to be evaluated in relation to the duration of the study and the preferred and usual lifestyle of the participant. It is not necessary to use any other method of contraception when complete abstinence is elected.)*

[a] Local laws and regulations may require use of alternative and/or additional contraception methods.

[b] Typical use failure rates may differ from those when used consistently and correctly. Use should be consistent with local regulations regarding the use of contraceptive methods for participants participating in clinical studies.

CONTRACEPTION GUIDANCE FOR MALE PARTICIPANTS WITH PARTNER(S) OF CHILD BEARING POTENTIAL.

Male participants with female partners of childbearing potential are eligible to participate if they agree to the following during treatment and until the end of relevant systemic exposure defined as 6 months after final drug administration.

- Inform any and all partner(s) of their participation in a clinical drug study and the need to comply with contraception instructions as directed by the investigator.
- Male participants are required to use a condom during treatment and until end of relevant systemic exposure defined as 6 months after final drug administration.
- Female partners of male participants who have not undergone a vasectomy with the absence of sperm confirmed or a bilateral orchiectomy should consider use of effective methods of contraception until the end of relevant systemic exposure, defined as 6 months after final drug administration.

12.4 Concomitant Medication Restrictions or Requirements

Prohibited Concomitant Treatment

The following are strictly prohibited:

- Sorivudine or analogs (during capecitabine treatment)
- Concurrent non-steroid systemic immunosuppressive agents (for systemic corticosteroids, see cautionary concomitant treatment below).
- Live vaccines should be avoided during the treatment period in which subject is receiving oxaliplatin or capecitabine and up to 6 months after final oxaliplatin or capecitabine dose. In cases where a live vaccine is needed for COVID-19 prevention and allowed per local regulations, please contact the Medical Monitor for discussion.
- Other systemic chemotherapy, immunotherapy, radiotherapy, herbal medications or other treatments intended for antitumor activity.
  - Palliative radiotherapy for peripheral bone metastases is allowed.
  - For gastric bleeding, palliative radiotherapy is prohibited, but esophagogastroduodenoscopy with epinephrine injection is allowed.
- Investigational products or therapy other than zolbetuximab.

Cautionary Concomitant Treatment

Considerations should be given to avoid or minimize the use of the following concomitant medications, if possible, during zolbetuximab/placebo treatment:

- Systemic corticosteroids, because their impact on the potential efficacy of zolbetuximab is not known.
  - Systemic corticosteroids should be avoided or minimized while subject is on study treatment unless required for management of an emergent medical condition (e.g., severe nausea/vomiting or hypersensitivity reaction).
  - For a subject's first dose of zolbetuximab/placebo, it is recommended that the prophylactic use of corticosteroids be avoided.
  - Inhaled, intranasal, ophthalmic, otic and topically applied steroids are allowed.
  - Subjects are allowed to use a physiologic replacement dose of hydrocortisone or its equivalent (defined as up to 30 mg/day of hydrocortisone or up to 10 mg/day of prednisone), receive a single dose of systemic corticosteroids or receive systemic corticosteroids as pre-medication for radiologic imaging contrast use.
- Administer 5-HT3 blockers with caution in subjects who have or may develop QTc prolongation.
- Nonsteroidal anti-inflammatory drugs (NSAIDs) because of the potential to cause gastric ulcers and covert bleeding.
  - In such cases where NSAID use is necessary, the use of NSAIDs with lower gastric ulcerogenic potential is preferred and concomitant gastric protection with proton pump inhibitors is recommended.

The following should be avoided or used with caution and closely monitored during capecitabine administration:

- Cytochrome P450 (CYP) 2C9 substrates (Subjects taking coumarin-derivative anticoagulants concomitantly with capecitabine should have PT/INR monitored regularly and anticoagulant dose adjusted accordingly).
- Anti-epileptic medications (e.g. phenobarbital, phenytoin and primidone)

The following should be avoided or used with caution and closely monitored during oxaliplatin administration:

- Medications known to prolong the QT or QTc interval (refer to https://www.crediblemeds.org for a list of these medications)

12.5 Liver Safety Monitoring and Assessment

Any subject enrolled in a clinical study with active drug therapy and reveals an increase of serum aminotransferases (AT) to > 3 x ULN (to > 5 x ULN in subjects with liver metastases) or bilirubin > 2 x ULN should undergo detailed testing for liver enzymes (including at least ALT, AST, alkaline phosphatase [ALP] and total bilirubin). Testing should be repeated within 72 hours of notification of the test results. For studies for which a central laboratory is used, alerts will be generated by the central laboratory regarding moderate and severe liver abnormality to inform the investigator, study monitor and study team. Subjects should be asked if they have any symptoms suggestive of hepatobiliary dysfunction.

Definition of Liver Abnormalities

Confirmed abnormalities will be characterized as moderate and severe where ULN:

	**ALT or AST**		**Total Bilirubin**
**Moderate**	> 3 x ULN (in subjects without liver metastases), > 5 x ULN (in subjects with liver metastases)	or	> 2 x ULN
**Severe***	> 3 x ULN	and	> 2 x ULN

In addition, the subject should be considered to have severe hepatic abnormalities for any of the following:

- ALT or AST > 8 x ULN.
- ALT or AST > 5 x ULN for more than 2 weeks (in the absence of liver metastases).
- ALT or AST > 3 x ULN and International Normalized Ratio (INR) > 1.5 (If INR testing is applicable/evaluated).
- ALT or AST > 3 x ULN with the appearance of fatigue, nausea, vomiting, right upper quadrant pain or tenderness, fever, rash and/or eosinophilia (> 5%).

The investigator may determine that abnormal liver function results, other than as described above, may qualify as moderate or severe abnormalities and require additional monitoring and follow-up.

Follow-up Procedures

Confirmed moderate and severe abnormalities in hepatic functions should be thoroughly characterized by obtaining appropriate expert consultations, detailed pertinent history, physical examination and laboratory tests. The site staff is to complete the liver abnormality case report form (LA-CRF). Subjects with confirmed abnormal liver function testing should be followed as described below.

Confirmed moderately abnormal LFTs should be repeated 2 to 3 times weekly then weekly or less if abnormalities stabilize or the study treatment has been discontinued and the subject is asymptomatic.

Severe hepatic liver function abnormalities as defined above, in the absence of another etiology may be considered an important medical event and may be reported as a SAE. The sponsor

should be contacted and informed of all subjects for whom severe hepatic liver function abnormalities possibly attributable to study treatment are observed.

To further assess abnormal hepatic laboratory findings, the investigator is expected to:

- Obtain a more detailed history of symptoms and prior or concurrent diseases. Symptoms and new-onset diseases is to be recorded as "AEs" in the (e)CRF. Illnesses and conditions such as hypotensive events, and decompensated cardiac disease that may lead to secondary liver abnormalities should be noted. Nonalcoholic steatohepatitis is seen in obese hyperlipoproteinemic, and/or diabetic subjects and may be associated with fluctuating AT levels. The investigator should ensure that the medical history form captures any illness that predates study enrollment that may be relevant in assessing hepatic function.
- Obtain a history of concomitant drug use (including nonprescription medication, complementary and alternative medications), alcohol use, recreational drug use and special diets. Medications, including dose, is to be entered in the (e)CRF. Information on alcohol, other substance use and diet should be entered on the LA-CRF or an appropriate document.
- Obtain a history of exposure to environmental chemical agents.
- Based on the subject's history, other testing may be appropriate including:
    - Acute viral hepatitis (A, B, C, D, E or other infectious agents),
    - Ultrasound or other imaging to assess biliary tract disease,
    - Other laboratory tests including INR, direct bilirubin.
- Consider gastroenterology or hepatology consultations.
- Submit results for any additional testing and possible etiology on the LA-CRF or an appropriate document.

Study Treatment Discontinuation

In the absence of an explanation for increased LFT's, such as viral hepatitis, preexisting or acute liver disease, presence of liver metastases or exposure to other agents associated with liver injury, the subject may be discontinued from study treatment. The investigator may determine that it is not in the subject's best interest to continue study treatment. Discontinuation of study treatment should be considered if:

- ALT or AST > 8 x ULN.
- ALT or AST > 5 x ULN for more than 2 weeks (in subjects without liver metastases).
- ALT or AST > 3 x ULN and total bilirubin > 2 x ULN or INR > 1.5 (If INR testing is applicable/evaluated).
- ALT or AST > 5 x ULN and (total bilirubin > 2 x ULN in subjects with liver metastases).
- ALT or AST > 3 x ULN with the appearance of fatigue, nausea, vomiting, right upper quadrant pain or tenderness, fever, rash and/or eosinophilia (> 5%).

In addition, if close monitoring for a subject with moderate or severe hepatic laboratory tests is not possible, study treatment should be discontinued.

*Hy's Law Definition—The 2 "requirements" for Hy's Law are:

1. Evidence that a drug can cause hepatocellular-type injury, generally shown by a higher rate than control of people with 3 x and greater transaminase elevations over ULN (2 x elevations are too common in treated and untreated subjects to be discriminating).
2. Cases of increased bilirubin (to at least 2 x ULN) in people with concurrent transaminase elevation to at least 3 x ULN (but it is almost invariably higher) and no evidence of intra-or extra-hepatic bilirubin obstruction (elevated ALP) or Gilbert's syndrome. [Temple, 2006]

FDA Guidance for Industry, "Drug-Induced Liver Injury: Premarketing Clinical Evaluation" 2009:

1. The drug causes hepatocellular injury, generally shown by a higher incidence of 3-fold or greater elevations above the ULN of ALT or AST than the (non-hepatotoxic) control drug or placebo.
2. Among study subjects showing such AT elevations, often with ATs much greater than 3 x ULN, 1 or more also show elevation of serum total bilirubin to > 2 x ULN, without initial findings of cholestasis (elevated serum ALP).
3. No other reason can be found to explain the combination of increased AT and total bilirubin, such as viral hepatitis A, B or C; preexisting or acute liver disease; or another drug capable of causing the observed injury.

References

Temple R. Hy's law: Predicting Serious Hepatotoxicity. Pharmacoepidemiol Drug Saf. 2006 April;15(Suppl 4):241-3.

Guidance for Industry titled "Drug-Induced Liver Injury: Premarketing Clinical Evaluation" issued by FDA on July 2009.

12.6 Common Serious Adverse Events

The following is a list of SAEs that the sponsor considers to be associated with the disease state being studied. The list does NOT change your reporting obligations or prevent the need to report an AE meeting the definition of an SAE as detailed in [Section 5.5.2 Definition of Serious Adverse Events]. The purpose of this list is to alert the investigator that some events reported as SAEs may not require expedited reporting to the regulatory authorities based on the classification of “common SAEs”. The investigator is required to follow the requirements detailed in [Section 5.5.5 Reporting of Serious Adverse Events].

For IND safety reporting, single occurrences of the following events may be excluded from expedited reporting to the FDA. If aggregate analysis of these events indicates they occur more frequently with study treatment, an expedited IND safety report may be submitted to the FDA.

AEs most likely related to Gastric or GEJ adenocarcinoma:

- Gastric reflux
- Abdominal pain
- Abdominal distention
- Dysphagia
- Loss of appetite

12.7 Pharmacogenomic Analysis With Banked Sample

INTRODUCTION

The pharmacogenomic (PGx) research aims to provide information regarding how naturally occurring differences in a subject's gene and/or expression of genes based on genetic variation may impact what treatment options are best suited for the subject. Through investigation of PGx by technologies such as genotyping, gene sequencing, statistical genetics and Genome-Wide Association Studies, the relationship between gene profiles and a drug's kinetics, efficacy or toxicity may be better understood. As many diseases may be influenced by 1 or more genetic variations, PGx research may identify which genes are involved in determining the way a subject may or may not respond to a drug. Samples for PGx are optional and will only be collected as allowed per local policy.

OBJECTIVES

The PGx research that may be conducted in the future with acquired blood samples is exploratory. The objective of this research will be to analyze or determine genes of relevance to clinical response, pharmacokinetics and toxicity/safety issues.

By analyzing genetic variations, it may be possible to predict an individual subject's response to treatment in terms of efficacy and/or toxicity.

SUBJECT PARTICIPATION

Subjects who have consented to participate in this study may participate in this PGx sub-study, if applicable per local policy. Subjects must provide written consent prior to providing any blood samples that may be used at a later time for PGx analysis.

SAMPLE COLLECTION AND STORAGE

Subjects who consent to participate in this sub-study will provide one tube of whole blood of approximately 4–6 mL per Astellas' instructions. Each sample will be identified by the unique subject number. Samples will be shipped to a designated banking CRO as directed by Astellas.

PGx ANALYSIS

Details on the potential PGx analysis cannot be established yet. Astellas may initiate the PGx analysis if evidence suggests that genetic variants may be influencing the drug's kinetics, efficacy and/or safety.

DISPOSAL OF PGx SAMPLES/DATA

All PGx samples collected will be stored for a period of up to 15 years following study database hard-lock. If there is no requirement for analysis, the whole blood sample will be destroyed after the planned storage period. The subject has the right to withdraw consent at any time. When a subject's withdraw notification is received, the PGx sample will be destroyed. The results of any PGx analysis conducted on a sample prior to its withdrawal will be retained at Astellas indefinitely unless otherwise specified by local regulation.

INFORMATION DISCLOSURE TO THE SUBJECTS

Exploratory PGx analysis may be conducted following the conclusion of the clinical study, if applicable. The results of the PGx analysis will not be provided to any investigators or subjects, nor can the results be requested at a later date. Any information that is obtained from the PGx analysis will be the property of Astellas.

12.8 Eastern Cooperative Oncology Group Performance Status Scale

GRADE	ECOG PERFORMANCE STATUS
0	Fully active, able to carry on all pre-disease performance without restriction
1	Restricted in physically strenuous activity but ambulatory and able to carry out work of a light or sedentary nature (e.g., light house work, office work)
2	Ambulatory and capable of all self-care but unable to carry out any work activities; up and about more than 50% of waking hours
3	Capable of only limited self-care; confined to bed or chair more than 50% of waking hours
4	Completely disabled; cannot carry on any self-care; totally confined to bed or chair
5	Dead

Reference: Oken MM, Creech RH, Tormey DC, Horton J, Davis TE, McFadden ET, et al. Toxicity and response criteria of the Eastern Cooperative Oncology Group. Am J Clin Oncol. 1982;5(6):649-55.

12.9 Clinical Study Continuity

INTRODUCTION

The purpose of this appendix is to provide acceptable alternate methods to assess safety and efficacy parameters, as appropriate, in the event the clinical study is interrupted at the country, state, site or participant level during any crisis (e.g., natural disaster, pandemic).

BENEFIT-RISK RATIONALE

Maintaining the safety of clinical study participants and delivering continuity of care in the clinical study setting is paramount during any crisis. The site is expected to follow the protocol and associated Schedule of Assessments [Table 1] unless the site PI determines the need to implement the alternate measures. The PI should notify Astellas and/or their CRA when these alternate measures are needed.

The approach outlined within this appendix defines which assessments are required to maintain a favorable benefit/risk to the participant, to maintain overall study integrity and to provide acceptable alternate methods to complete the study required assessments and procedures if study activities are unable to be performed as described in Table 1 due to a crisis.

INFORMED CONSENT

Participants who need to follow any or all of the alternate measures outlined in this Appendix will be required to provide informed consent, which explicitly informs them of the nature of, and rationale for these changes, and gain their agreement to continue participation in the study prior to the implementation of any of these changes. In the event the urgency of implementing the alternate measures does not allow for the participant to provide written consent prior to implementation, the PI or designee will obtain oral agreement from the subject followed by written documentation as soon as is feasible. A separate addendum to the study informed consent will be provided to document the participant's consent of the changes.

PARTICIPANT PROCEDURES ASSESSMENT

Sites with participants who are currently enrolled into this clinical study may consider implementing the alternate methods outlined below if one or more of the following conditions are met due to the crisis:

- Regional or local travel has been restricted, inclusive of mandatory shelter in place measures, which makes participant travel to/from the study site nearly impossible
- Site facilities have been closed for clinical study conduct
- Site has been restricted to treating patients with conditions outside of the scope of the study
- Site personnel have temporarily relocated the conduct of the study to a location that place a burden on the participant with respect to time and travel

- Participant(s) have temporarily relocated from the current study site to an alternate study site to avoid placing a burden on the participant with respect to travel
- Participant(s) have temporarily relocated from their home location and the new distances from the site would cause undue burden with respect to time and travel
- Participant has risk factors for which traveling to the site poses an additional risk to the participant’s health and safety

Adherence to the original protocol as reflected in the Schedule of Assessments [Table 1] is expected, where plausible, in the case of a crisis. The alternate measures as noted in [Table 10] below are only permissible in the event of a crisis, and after discussing the need with the Astellas Medical Monitor to implement the alternate measures. This is to allow for continuity of receiving investigational medicinal product (IMP) and maintaining critical safety and efficacy assessments for patients participating in the study at a time of crisis.

If one or more of the alternate measures noted below is implemented for a participant, the site should document in the participant's source document the justification for implementing the alternate measure and the actual alternate measures that were implemented, along with the corresponding time point(s).

Table 10 Alternative Schedule of Assessments in Response to a Crisis

Critical Assessment†	Alternate Approach(es)	Critical Timepoint†										
		Cycles 1-8			Cycle 9+			Study Treatment Discontinuation Visit[18]	30-Day Safety Follow-up Visit(s)[19]	90-Day Safety Follow-up Visit(s)[20]	Post-treatment Follow-up Period [21]	Long-term and Survival Follow-up Periods [22]
**Cycle Day**		**1**	**2-14**	**15-21**	**1**	**2-14**	**15-21**					
**Treatments**												
Antiemetic Pretreatment[5]	Oral antiemetics can be administered at home as per SoC	X			X							
Zolbetuximab/Placebo[6]	Window of -2 days acceptable; at least every other cycle must be conducted at the study site for zolbetuximab/placebo and oxaliplatin administration and capecitabine dispensing	X			X							
Post-infusion Observation Period[7]	Decrease of initial observation period to 1 hour and subsequent observation period to 30 min is acceptable if there are no AEs of ≥ grade 2	X			X							

Table continued on next page

Critical Assessment†		Alternate Approach(es)	Critical Timepoint†										
			Cycles 1-8			Cycle 9+			Study Treatment Discontinuation Visit[18]	30-Day Safety Follow-up Visit(s)[19]	90-Day Safety Follow-up Visit(s)[20]	Post-treatment Follow-up Period [21]	Long-term and Survival Follow-up Periods [22]
**Cycle Day**			**1**	**2-14**	**15-21**	**1**	**2-14**	**15-21**					
Oxaliplatin	CAPOX[8]	If previous cycle Day 1 zolbetuximab/placebo administration was conducted at the study site, the following cycle can be administered (oxaliplatin) and dispensed (capecitabine) locally per SoC only (not zolbetuximab/placebo administration) by oncology qualified personnel, and if dosing records can be obtained from the treating facility. The next cycle then must be administered at the study site for zolbetuximab/placebo administration. At least every other cycle must be conducted at the study site for zolbetuximab/placebo and oxaliplatin administration and capecitabine dispensing.	X										
Capecitabine			X	X		X	X						

Table continued on next page

Critical Assessment†	Alternate Approach(es)	Critical Timepoint†										
		Cycles 1-8			Cycle 9+			Study Treatment Discontinuation Visit[18]	30-Day Safety Follow-up Visit(s)[19]	90-Day Safety Follow-up Visit(s)[20]	Post-treatment Follow-up Period [21]	Long-term and Survival Follow-up Periods [22]
**Cycle Day**		1	2-14	15-21	1	2-14	15-21					
**Safety Assessments**												
Physical Examination[9]	Targeted exam is allowed after C1D1. Physical exam not completed at Study Treatment Discontinuation Visit and 30-Day Safety Follow-up Visit acceptable if no active AE.	X			X							
Weight[9]	If there are no associated active AEs, it is acceptable if weight is not done at Study Treatment Discontinuation Visit and 30-Day Safety Follow-up Visit.	X			X							
Vital Signs[10]	If a cycle is administered at a local facility for SoC regimen administration, SoC can be applied. Missed assessments at Study Treatment Discontinuation Visit and 30-Day Safety Follow-up visit acceptable if there are no associated active AEs. Vital sign frequency during post observation period can be decreased.	X			X							
ECOG Performance Status[9]	Not required at Study Treatment Discontinuation visit; ECOG Performance Status may be assessed and captured via phone contact.	X			X							

Table continued on next page

**Critical Assessment†**	**Alternate Approach(es)**	**Critical Timepoint†**										
		**Cycles 1-8**			**Cycle 9+**			**Study Treatment Discontinuation Visit[18]**	**30-Day Safety Follow-up Visit(s)[19]**	**90-Day Safety Follow-up Visit(s)[20]**	**Post-treatment Follow-up Period [21]**	**Long-term and Survival Follow-up Periods [22]**
**Cycle Day**		**1**	**2-14**	**15-21**	**1**	**2-14**	**15-21**					
12-lead ECG[11]	ECGs allowed up to 4 days prior to treatment visits after C1D1 and can be done locally but must be reviewed prior to dosing; if dosing visits are conducted at local facility, SoC ECG monitoring would be acceptable; if capecitabine only is dispensed and ECG is not able to be performed, this is acceptable; Study Treatment Discontinuation Visit and 30-Day Safety Follow-up Visit required only if clinically indicated.	X			If clinically indicated and/or per local requirements							
**Image Assessment[12]**	At select visits, efficacy assessment using radiological examinations are required. Independent central reading of locally obtained scans can be facilitated by sharing of Image Acquisition Guidelines from the study site to local site, if applicable. Imaging assessment can be done locally but must be available for submission to central imaging vendor. Investigational site will be requested to re-read the scan performed at local site. If investigational site read is not an option, the investigator should discuss the case with the local institution radiologist. The local site imaging report is required.	Every 9 weeks ±7 days from C1D1 for the first 54 weeks and then every 12 weeks ±7 days thereafter										

Table continued on next page

**Critical Assessment†**	**Alternate Approach(es)**	**Critical Timepoint†**										
		**Cycles 1-8**			**Cycle 9+**			**Study Treatment Discontinuation Visit[18]**	**30-Day Safety Follow-up Visit(s)[19]**	**90-Day Safety Follow-up Visit(s)[20]**	**Post-treatment Follow-up Period [21]**	**Long-term and Survival Follow-up Periods [22]**
**Cycle Day**		**1**	**2-14**	**15-21**	**1**	**2-14**	**15-21**					
**Subject Contact**	Long Term Survival and Safety follow-up visits can be conducted via phone.											X
**Laboratory Tests**												
Biochemistry[13]	Sample may be collected up to 4 days prior to treatment visit; collection of samples at local facility acceptable if results can be made available to investigative site.	X			X			X	X			
TSH and Free T4[13]	If this testing is unable to be performed, this is acceptable	If clinically indicated										
Hematology[13]	Sample may be collected up to 4 days prior to treatment visit; collection of samples at local facility acceptable if results can be made available to investigative site.	X			X			X	X			
Coagulation Parameters (PT, PTT and INR)[14]	None as protocol allows SoC if clinically indicated. If the subject is not on a concomitant medication that affects these parameters, it is acceptable if these are not done.	If clinically indicated										
Urinalysis[13]	Sample may be collected up to 4 days prior to treatment visit; collection at local facility also allowed.	X			X			X	X			
Serum Pregnancy Test[15]	Collection at local facility also allowed.	If clinically indicated and/or per local requirements										

Table continued on next page

Critical Assessment†	Alternate Approach(es)	Critical Timepoint†										
		Cycles 1-8			Cycle 9+			Study Treatment Discontinuation Visit[18]	30-Day Safety Follow-up Visit(s)[19]	90-Day Safety Follow-up Visit(s)[20]	Post-treatment Follow-up Period [21]	Long-term and Survival Follow-up Periods [22]
**Cycle Day**		1	2-14	15-21	1	2-14	15-21					
Urine Pregnancy Test[16]	Sample may be collected up to 4 days prior to treatment visit; collection at local facility also allowed if results can be made available to investigative site.	X			X			X	X			
**Sampling**												
Pharmacokinetics of Zolbetuximab (Serum)[23]	Samples at predose/End of Infusion will be collected if subject receives treatment at investigative site. If central lab cannot receive samples, samples can be stored at sites in -70°C freezer until shipping is accepted again. Follow-up samples will be collected if subject visits the investigative site.	X			X				X	X		
Antidrug-Antibodies (ADA) for Immunogenicity[24]	If subject is dosed or visits investigative site, ADA samples should be collected. Samples cannot be collected at local facility. Sample collection prioritized if clinically indicated. If central lab cannot receive samples, samples can be stored at sites in -70°C freezer until shipping is accepted again.	X			X				X	X		

Table continued on next page

Critical Assessment†	Alternate Approach(es)	Critical Timepoint†										
		**Cycles 1-8**			**Cycle 9+**			**Study Treatment Discontinuation Visit[18]**	**30-Day Safety Follow-up Visit(s)[19]**	**90-Day Safety Follow-up Visit(s)[20]**	**Post-treatment Follow-up Period [21]**	**Long-term and Survival Follow-up Periods [22]**
**Cycle Day**		**1**	**2-14**	**15-21**	**1**	**2-14**	**15-21**					
Genetic Immune Polymorphisms (Whole Blood)[25]	In general, biomarker samples are collected on zolbetuximab treatment days and do not require a unique visit to the study site. If samples can be collected but central labs cannot receive samples, samples can be stored at sites until shipping is accepted again.	X										
Exploratory Biomarkers (Serum)[26]	In general, biomarker samples are collected on zolbetuximab treatment days and do not require a unique visit to the study site. If samples can be collected but central labs cannot receive samples, samples can be stored at sites until shipping is accepted again.	X						X				
Exploratory Biomarkers (Plasma)[26]	In general, biomarker samples are collected on zolbetuximab treatment days and do not require a unique visit to the study site. If samples can be collected but central labs cannot receive samples, samples can be stored at sites until shipping is accepted again.	X						X				

Table continued on next page

**Critical Assessment†**	**Alternate Approach(es)**	**Critical Timepoint†**										
		**Cycles 1-8**			**Cycle 9+**			**Study Treatment Discontinuation Visit[18]**	**30-Day Safety Follow-up Visit(s)[19]**	**90-Day Safety Follow-up Visit(s)[20]**	**Post-treatment Follow-up Period [21]**	**Long-term and Survival Follow-up Periods [22]**
**Cycle Day**		**1**	**2-14**	**15-21**	**1**	**2-14**	**15-21**					
Whole Blood Sample for PGx (optional)[27]	In general, biomarker samples are collected on zolbetuximab treatment days and do not require a unique visit to the study site. If samples can be collected but central labs cannot receive samples, samples can be stored at sites until shipping is accepted again.	X										
Post-progression Tumor Sample (optional)[28]	In general, biomarker samples are collected on zolbetuximab treatment days and do not require a unique visit to the study site. If samples can be collected but central labs cannot receive samples, samples can be stored at sites until shipping is accepted again.							X				
**Concomitant Medication[29]**	Remote/Virtual/Telemedicine "Visits" allowed for non-dosing visits. Please refer to protocol schedule of assessments.	X			X			X	X	X		
**AE/SAE[30]**	Remote/Virtual/Telemedicine "Visits" allowed for non-dosing visits. Please refer to protocol schedule of assessments.	X			X			X	X	X		

Footnotes appear on next page

ADA: antidrug antibody; AE: adverse event; βhCG: beta human chorionic gonadotropin; C1D1: Cycle 1 Day 1; CAPOX: capecitabine and oxaliplatin; CLDN: claudin; CT: computerized tomography; DPD: dihydropyrimidine dehydrogenase; eCOA: electronic Clinical Outcomes Assessment; ECG: electrocardiogram; ECOG: Eastern Cooperative Oncology Group; eCRF: electronic case report form; FFPE: formalin fixed paraffin embedded; HER2: human epidermal growth factor receptor 2; HRQoL: health-related quality of life; HRU: Health Resource Utilization; ICF: informed consent form: INR: international normalized ratio; IRC: independent review committee; IRR: infusion-related reaction; IRT: interactive response technology; IV: intravenous; MRI: magnetic resonance imaging; OS: overall survival; PD: progressive disease; PFS: progression free survival; PFS2: progression free survival following subsequent anticancer treatment; PGx: pharmacogenomics; PT: prothrombin time; PTT: partial thromboplastin time; RECIST: Response Evaluation Criteria In Solid Tumors; SAE: serious adverse event; SoC: standard of care; T4: thyroxine; TSH: thyroid stimulating hormone

†In case subjects are unable to be evaluated in person on Day 1 of each cycle, the site will contact the subject by phone for a safety assessment at the time the next visit would be due.

*+7 calendar day visit window does not apply to C1D1.

1. Screening: The Screening period is 45 days from full main ICF signature. Retesting of lab values is allowed within the 45-day Screening period. Re-screening outside the 45-day window under a new subject number may be allowed once and upon discussion with the Medical Monitor. CT scans and MRIs conducted as part of a subject's routine clinical management (i.e., standard of care) obtained before signing the ICF may be utilized for screening or baseline purposes, provided the procedures met the protocol-specified criteria and were performed within the Screening period.
   - Optional partial screening: A partial screening ICF may be available for central testing of tissue for CLDN18.2 and HER2 only.
   - Laboratory testing:
     - Eligibility can be determined based on central and/or local laboratory testing; **however**:
       - The most recent laboratory tests with available results must be used to confirm the subject's eligibility.
       - Central labs must be collected and submitted to the central laboratory during the Screening period.
       - If retesting of lab values is necessary to confirm eligibility, local labs can be used without requiring additional sample collection for central laboratory submission.
     - The screening labs used to determine eligibility should be collected within 14 days prior to randomization.
   - Radiologic imaging used to confirm eligibility must be conducted within 28 days prior to randomization.
2. CLDN18.2 and HER2 Testing: FFPE tumor tissue will be collected for central testing to determine CLDN18.2 and HER2 status. Archival tumor tissue from the primary tumor (gastric or GEJ) is preferred. If primary tumor tissue is not available, tumor tissue from a metastatic site (excluding bone metastasis) may be used. A minimum of 1 FFPE tumor tissue block (preferred) OR a minimum of 15 FFPE unstained slides are required as allowed per local policy. If slides are submitted, the slides should be freshly cut from the FFPE block within the time frame described in the laboratory manual. If local HER2 results are already available from local testing, a minimum of 12 FFPE unstained slides are required to be submitted to the central lab as allowed per local policy. If the specimen is insufficient or unavailable, a biopsy may be performed to obtain primary tumor tissue (preferred) or tumor tissue from metastatic site (excluding bone metastasis). Sponsor pre-approval is required when the sole purpose of the biopsy procedure is to assess eligibility for this study. If the required number of slides cannot be provided, the sponsor or designee should be contacted for further guidance. See [Section 5.7.3 Tumor Tissue Samples].
3. Confirmation of Inclusion/Exclusion Criteria must be completed prior to randomization.
4. Randomization: After confirmation of eligibility, the blinded site user will perform the randomization IRT transaction. The unblinded pharmacist/designee will be notified by the IRT system about the randomly assigned treatment. Randomization may be performed prior to C1D1. If C1D1 cannot be performed within 5 calendar days from Randomization please contact the Medical Monitor for discussion. Details of infusion preparation and storage requirements are defined in the Pharmacy Manual and Infusion Guidelines.

Footnotes continued on next page

5. Antiemetic Pretreatment: Antiemetic premedication (prophylactic antiemetics) should be administered prior to each study treatment. IV antiemetic premedication should be initiated prior to treatment, or oral antiemetic premedication should be initiated at a minimum of 30 minutes prior to treatment. Antiemetic premedication should be given according to institutional standard of care, published guidelines and the respective product package insert(s). For further details, see [Section 5.1.1.2 Antiemetics].
6. Zolbetuximab/placebo will be administered as a minimum 2-hour intravenous infusion every 3 weeks starting on C1D1. Please refer to Pharmacy Manual and Infusion Guidelines for more detailed information. Zolbetuximab/placebo should be administered prior to CAPOX. For further details, see [Section 5.1.1.1 Zolbetuximab/Placebo].
7. Post-infusion Observation Period: Following the first dose of zolbetuximab/placebo on C1D1, the subject must be observed for 2 hours post-zolbetuximab/placebo infusion. The post-infusion observation period can be conducted during the CAPOX administration. If any ≥ grade 2 AEs are observed during infusion or during the post-infusion observation period, subsequent zolbetuximab/placebo infusion times should be extended and subjects should continue to be observed for 2 hours post zolbetuximab/placebo infusion. If the subject does not develop any grade ≥ 2 AEs, the subject should be observed for 1 hour post-infusion for their subsequent zolbetuximab/placebo infusions. The subject should be instructed to notify site personnel if they develop any AEs during this observation time period. In the event of an IRR with features of anaphylaxis (regardless of grade) or grade 3 or 4 IRR, blood samples for cytokine/chemokine panel and serum total tryptase level (levels typically peak within 3 hours after the onset of symptoms) should be collected once the subject has stabilized, for shipment to the central laboratory. See Observation Period following zolbetuximab/placebo infusion [Section 5.4.2] for further details.
8. CAPOX is a combination of oxaliplatin intravenous infusion and capecitabine tablets and will be administered starting at C1D1 for up to 8 treatments. See [Section 5.1.1.3].
9. Physical Exam: should include height (at Screening only), weight and ECOG performance status. A full physical exam is required at Screening. The physical exam only needs to be repeated on C1D1 if clinically significant changes from screening are observed (in the opinion of the investigator). For all cycles, the physical examination, weight and ECOG performance status assessments can be completed up to 48 hours prior to zolbetuximab/placebo administration. Targeted (symptom driven) physical exams should be conducted every 3 weeks on day 1 of each cycle. For further details, see [Section 5.4.4 Physical Examination]
10. Vital signs (pulse, blood pressure, temperature) should be taken during every visit at the following time points (see [Section 5.4.1 Vital Signs]):
    - Predose at every visit
    - C1D1: Every 30 (±10) minutes during zolbetuximab/placebo infusion
    - If the subject did not develop any ≥ grade 2 AEs during the C1D1 zolbetuximab/placebo infusion or Post-infusion Observation Period, the site may do the following for subsequent zolbetuximab/placebo infusions:
        - The post-infusion observation period can be 1 hour for subsequent visits after C1D1
        - The vital signs can be assessed every 60 minutes for subsequent visits after C1D1
    - Every 60 (±10) minutes post zolbetuximab/placebo infusion during the Post-infusion Observation Period (for 1 or 2 hours. See footnote 7)
    - Unscheduled if clinically indicated
11. ECGs: ECGs will be locally assessed. When collected on the same day, ECG should be collected prior to pharmacokinetic samples. For further details, see [Section 5.4.5 Electrocardiogram]. A single ECG will be performed at the following time points:
    - Screening
    - Up to 48 hours prior to every oxaliplatin infusion (before any antiemetic treatment administration)
    - Up to 6 hours following completion of every oxaliplatin infusion
    - Zolbetuximab/placebo Discontinuation Visit
    - Zolbetuximab/placebo 30-day Follow-up Visit
    - If clinically indicated or per local requirements

Footnotes continued on next page

12. Imaging Assessments: Radiologic imaging will be evaluated at Screening (must be conducted within 28 days prior to randomization) and every 9 weeks (± 7 days) counting from C1D1 for the first 54 weeks and then every 12 weeks (± 7 days) thereafter until subject develops radiological disease progression per RECIST 1.1 by IRC or starts other systemic anticancer treatment, whichever comes earlier. Imaging schedule should be maintained regardless of treatment delay. Imaging will include CT scans with contrast of the thorax, abdomen, and pelvis. If CT scan with contrast is medically not feasible, MRI may be used for imaging. Bone scans (or focal X-ray) or brain imaging should be performed if metastatic disease in bone or brain is suspected, respectively. The same mode of imaging should be utilized throughout the study unless medical necessity requires a change. For randomized subjects, screening imaging should be sent to the central imaging vendor no later than at the time of submission of the first on-treatment imaging. All imaging acquired post randomization will be sent to the central imaging vendor within 7 days of scanning for the blinded independent central assessment of radiological tumor response based on RECIST 1.1. The investigator should make every effort to immediately submit radiologic assessments for IRC review when PD is suspected. See [Section 5.3 Efficacy Assessments]. Refer to Imaging Acquisition Guidelines for further detail on scan modality and contrast options.
13. Laboratory Assessments: See [Section 5.4.3 Laboratory Assessments] for list of laboratory assessments. Laboratory tests must be sent to the central laboratory for analysis. For screening/eligibility laboratory assessments, see footnote number 1.
    - Laboratory test results (central or local) will be reviewed by the investigator prior to any study treatment. Clinical significance of out-of-range laboratory findings is to be determined and documented by the investigator/sub-investigator who is a qualified physician.
    - Local laboratory results may be used for treatment decisions; however, central laboratory samples must also be drawn per protocol and sent to the central laboratory.
    - Central and local labs may be collected up to 48 hours prior to study treatment.
    - Holidays and weekends should be taken into account when scheduling these blood draws.
    - Additional assessments may be done centrally or locally to monitor AEs or as clinically indicated.
14. Coagulation (PT, PTT and INR): Coagulation tests should be done at Screening and during study treatment period if clinically indicated. Local or central lab results may be used to confirm eligibility. Ongoing evaluation should be continued for subjects who are receiving therapeutic anticoagulation according to local standard of care. See [Section 5.4.3 Laboratory Assessments].
15. Serum Pregnancy Test: Serum pregnancy tests will be collected for female subjects of childbearing potential only. Serum pregnancy tests collected at Screening, during study treatment period and if clinically indicated or per local requirements. Serum pregnancy test can be completed up to 48 hours prior to zolbetuximab/placebo administration. (Note: For Screening, subjects with elevated serum βHCG and a demonstrated non-pregnant status through additional testing are eligible.) Local or central laboratory results may be used to confirm eligibility.
16. Urine Pregnancy Test: Urine pregnancy tests will be collected for female subjects of childbearing potential only. Local urine pregnancy tests to be performed during the treatment period every 3 weeks on day 1 of each cycle and at the zolbetuximab/placebo Study Treatment Discontinuation and 30-day Safety Follow-up Visits. Urine pregnancy test can be completed up to 48 hours prior to zolbetuximab/placebo administration. Additional urine pregnancy testing for up to 9 months after the final study treatment administration may be conducted based on local requirements.
17. HRQoL and HRU questionnaires: eCOA questionnaires should be completed by the subject at Screening (except for HRU), on day 1 of each cycle (or up to 48 hours) before any antiemetic or drug treatment and before the disease status is discussed with the subject using the electronic tablet device provided. When completion by the subject is not possible, the questionnaires may be administered to the subject by site personnel using the electronic tablet device. For subjects with low literacy, situations where translations are unavailable or other circumstances preventing the screening questionnaires to be completed, please contact the sponsor for further guidance.

Footnotes continued on next page

18. Study Treatment Discontinuation Visit (End of Study Treatment): The Study Treatment Discontinuation Visit will take place ≤ 7 days following the decision to discontinue study treatment (zolbetuximab/placebo and CAPOX [both components]). If zolbetuximab/placebo and CAPOX (both components) are discontinued on a different day, subjects will have separate Study Treatment Discontinuation Visits following each treatment's discontinuation. Laboratory tests must be sent to the central laboratory for analysis. HRQoL and HRU questionnaires are not required at CAPOX treatment discontinuation visit. A combined visit can be completed if zolbetuximab/placebo are discontinued on the same day. If necessary during a crisis, this visit can be completed remotely for safety assessments and the required laboratory samples should be collected as soon as the crisis permits.
19. 30-day Safety Follow-up Visit: A 30-day Safety Follow-up Visit should occur 30 days after the last dose of zolbetuximab/placebo and will include the assessments as shown in the Schedule of Assessments above. A 30-day Safety Follow-up Visit should occur 30 days after the last dose of CAPOX (both components) and may be conducted by phone if the subject is unable to visit the site and will require contact for AE/SAE collection only. HRQoL and HRU questionnaires are not required at CAPOX 30-day safety follow up visit. A combined visit can be completed if zolbetuximab/placebo and both components of CAPOX are discontinued on the same day and HRQoL and HRU questionnaires should be completed for a combined visit. If necessary during a crisis, this visit can be completed remotely for safety assessments and the required laboratory samples should be collected as soon as the crisis permits.
20. 90-day Safety Follow-up Visits: A 90-day Safety Follow-up Visit should occur 90 days after the last dose of zolbetuximab/placebo and will include the assessments as shown in the Schedule of Assessments above. A 90-day Safety Follow-up Visit should occur 90 days after the last dose of CAPOX (both components) and may be conducted by phone if the subject is unable to visit the site and will require contact for AE/SAE collection only. HRQoL and HRU questionnaires are not required at CAPOX 90-day safety follow up visit. A combined visit can be completed if zolbetuximab/placebo and both components of CAPOX are discontinued on the same day and HRQoL and HRU questionnaires should be completed for a combined visit.
21. Post-treatment Follow-up: if a subject discontinues all study treatments (zolbetuximab/placebo and both components of CAPOX) prior to IRC-confirmed radiological disease progression, the subject will enter the Post-treatment Follow-up Period and continue to undergo imaging assessments every 9 weeks (±7 days) (or every 12 weeks [±7 days] if subjects has been on study over 54 weeks) until radiologic disease progression (i.e., PFS) or the subject starts subsequent anticancer treatment, whichever occurs earlier. If study treatments (zolbetuximab/placebo and both components of CAPOX) are discontinued due to PD, the subject will enter the Long-term and Survival Follow-up Period.
22. Long-term and Survival Follow-up Period: Following disease progression on first-line treatment or start of subsequent anticancer treatment, subjects will be followed in the Long-term and Survival Follow-up Period per institutional guidelines, but not less than every 12 weeks. Radiologic imaging will be done per standard of care and read locally until PFS2 is documented. Survival Follow-up Period will continue until death (from any cause). All post-progression details including subsequent anticancer treatment and date and site of progression will be recorded on the eCRF. Subject contact by phone or other remote methods is sufficient during Long-term and Survival Follow-up.
23. Pharmacokinetics: Serum samples for zolbetuximab/placebo will be taken at the below time points and sent to the central laboratory. The date and time of each blood sample collection will be recorded to the nearest minute.
    - Cycle 1 Day 1: End of zolbetuximab/placebo infusion
    - Cycle 2 Day 1: Predose
    - Cycle 3 Day 1: End of zolbetuximab/placebo infusion
    - Predose on Day 1 of Cycles 5, 9, 13 and 17
    - Zolbetuximab/placebo 30-Day Safety Follow-up visit
    - Zolbetuximab/placebo 90-Day Safety Follow-up visit
    - Unscheduled pharmacokinetic blood samples may be taken at any time during the study to evaluate drug exposure following a safety event

Footnotes continued on next page

Pharmacokinetic Sampling Window:

- Predose: within 60 minutes prior to dosing
- End of Infusion: within 10 minutes after the end of the infusion

24. ADA: Blood samples (Serum) for ADA will be taken at the below time points and sent to the central laboratory.
    - Cycle 1 Day 1: Predose
    - Cycle 2 Day 1: Predose
    - Predose on Day 1 of Cycles 5, 9, 13 and 17
    - Zolbetuximab/placebo 30-Day Safety Follow-up visit
    - Zolbetuximab/placebo 90-Day Safety Follow-up visit

    ADA Sampling Window: Predose: within 60 minutes prior to dosing
25. Genetic Immune Polymorphism: To be collected per local policy. Whole blood sample taken at C1D1 will be sent to the central laboratory. Samples may be collected up to 48 hours prior to study treatment.
26. Exploratory Biomarker (Serum and Plasma): To be collected per local policy. Samples should be taken at the below time points and sent to the central laboratory:
    - Cycle 1 Day 1: Predose
    - Cycle 2 Day 1: Predose
    - Cycle 3 Day 1: Predose
    - Cycle 4 Day 1: Predose
    - Cycle 5 Day 1: Predose
    - Cycle 6 Day 1: Predose
    - Cycle 8 Day 1: Predose
    - Zolbetuximab/placebo Study Treatment Discontinuation Visit

    Exploratory Biomarker samples may be collected up to 48 hours prior to study treatment
27. Optional PGx: For subjects who signed a separate ICF, an optional whole blood sample for PGx for exploratory biomarker analysis may be collected up to 48 hours prior to first study drug administration. Sample collection is optional and only collected as allowed per local policy.
28. Optional Post-Progression Tumor Sample: For subjects who signed a separate ICF, an optional post-progression tumor sample for exploratory biomarker analysis may be collected following IRC confirmation of disease progression and prior to initiation of subsequent anticancer therapy. Sample collection is optional and only collected as allowed per local policy.
29. Concomitant medications will be collected from the time of full main informed consent through 90 days following the last dose of study treatment.
30. AEs/SAEs: AEs and SAEs (regardless of causality) will be collected from the time of full main informed consent through 90 days following the last dose of study treatment. See [Section 5.5.5 Reporting of Serious Adverse Events].

IMP SUPPLY

If any of the conditions outlined above in the Participants Procedures Assessment are met, one or all of the following mitigating strategies will be employed, as needed, to ensure continuity of IMP supply to the participants:

- Increase stock of IMP on site to reduce number of shipments required, if site space will allow, as cold storage space is needed.

DATA COLLECTION REQUIREMENTS

Additional data may be collected in order to indicate how participation in the study may have been affected by a crisis and to accommodate data collection resulting from alternate measures implemented to manage the conduct of the study and participant safety.

- Critical assessments for safety and efficacy based on study endpoints to be identified as missing or altered (performed virtually, at alternative locations, out of window, or other modifications) due to the crisis.

13 ATTACHMENT 1: SUBSTANTIAL AMENDMENT 4

Protocol Amendment Summary of Changes

Protocol 8951-CL-0301 A Phase 3, Global, Multi-Center, Double-Blind, Randomized, Efficacy Study of Zolbetuximab (IMAB362) Plus mFOLFOX6 Compared with Placebo Plus mFOLFOX6 as First-line Treatment of Subjects with Claudin (CLDN)18.2-Positive, HER2-Negative, Locally Advanced Unresectable or Metastatic Gastric or Gastroesophageal Junction (GEJ) Adenocarcinoma

Amendment 4 [Substantial] 18 Oct 2021

This amendment is considered to be substantial based on the criteria set forth in Article 10(a) of Directive 2001/20/EC of the European Parliament and the Council of the European Union

Overall Rationale for the Amendment:

Reduction in the number of PFS events required for primary analysis as well as addition of key secondary endpoints for health-related quality of life questionnaires.

Summary of Changes

Table 1 Substantial Changes

Section Number	Description of Change	Brief Rationale
IV, 2.1.2, 2.3.2, 5.7.6.1, 5.7.6.2, 7.4.2.2	Addition of health economics and outcomes research (HEOR) related key secondary endpoints, including physical function, Pain, and Global Health Score.	A key secondary endpoint for QOL measures has been added after FDA interaction in order to more specifically address the effect of zolbetuximab in gastric/GEJ cancer, which impacts the risk/benefit assessment.
IV, 2.2.1, 7.1, 7.4.1.1	The number of PFS events required for the interim analysis of overall survival is reduced from 344 to 300.	The number of required PFS events has been adjusted based on the enrollment and event accrual rates to maintain the timing of Primary Analysis with adequate power which is > 93%.
IV, 5.1.5, 12.4	Clarify that a subject receiving oxaliplatin should not receive live vaccines.	Administration of live or live attenuated vaccines in patients immunocompromised by chemotherapeutic agents may result in serious or fatal infections.
Table continued on next page		

Section Number	Description of Change	Brief Rationale
IV, 7.2.2, 7.3, 7.4, 7.4.1.2, 7.4.2.1	The Per Protocol Set (PPS) has been removed from the protocol.	The PPS is defined as the subjects who do not meet predetermined study entry and treatment criteria as well as those with lack of imaging assessment.<br>The data from subjects meeting these criteria are unlikely to allow adequate assessment of potential impact on treatment benefit, possibly resulting in risk of bias. Therefore, the robustness of treatment benefit in Primary Endpoint will instead be assessed through sensitivity analyses applying different censoring rules.

Table 2 Nonsubstantial Changes

Section Number	Description of Change	Brief Rationale
II	Contact details for the clinical research contact and global clinical research contact are updated.	Contact details of sponsor personnel are updated based on changes to study personnel.
IV	Correct planned study period to end 2Q2023	To reflect the length of the planned study period
IV, V (Table 1 [footnote 1]), 2.2.1, 12.9 (Table 10 [footnote 1])	Specify that CT scans and MRIs conducted as a part of a subject's routine clinical management obtained prior to signing the informed consent form may be utilized for screening or baseline purposes.<br>ECGs removed from the change in text.	Additional instructions are provided for clarity.
IV, V (Table 1 [footnote 5]), 5.1.1.2, 12.9 (Table 10 [footnote 5])	Text revised to clarify prophylactic antiemetic management.	To clarify the timing of oral and IV antiemetics.
IV, V (Table 1 [footnotes 15 and 16]), 3.2 (inclusion criteria #3 and #5), 5.5.9.1, 12.3, 12.9 (Table 10 [footnotes 15 and 16])	Contraceptive guidance is updated to clarify that requirements are applicable for 9 months after the final administration of oxaliplatin and 6 months after the final administration of all other study drugs.	To align with the FDA label for oxaliplatin use in ensuring adequate contraception and pregnancy testing.
Table continued on next page		

Section Number	Description of Change	Brief Rationale
IV, 3.2 (inclusion criterion #17), 5.4.3	This inclusion criterion is updated to clarify that when multiple blood draws are done, the most sample collection draw with available results should be used for determination of eligibility.	This revision is made for clarification.
IV, 3.3 (exclusion criterion #1)	Subjects are not prohibited from participation if they received prior immunotherapy or other systemic anticancer therapies as long as it was completed at least 6 months prior to randomization.	Clarified the types of immunological therapies that are not prohibited.
V (Table 1 [general footnote]), 12.9 (Table 10 [general footnote])	Footnote added to clarify that in case subjects are unable to be evaluated in person on day 1 of each cycle, the site will contact the subject by phone for a safety assessment at the time the next visit would be due.	This revision is made for clarification.
V (Table 1 [footnotes 9, 15, 16]), 5.4.4, 12.9 (Table 10 [footnotes 9, 15, 16])	Clarify that the urine and serum pregnancy tests, physical examination, weight and ECOG performance status assessments can be completed up to 48 hours prior to zolbetuximab/placebo administration.	Provide a window for pre-treatment testing to decrease subject burden and align with standard subject flow for pre-treatment testing.
V (Table 1 [footnote 10]), 12.9 (Table 10 [footnote 10])	The vital sign observation period for subsequent zolbetuximab/placebo infusions is updated to include a reduction of observation time from every 30 minutes to every 60 minutes and a reduction of intervals for assessing vital signs in some situations.	This revision is made for clarification and to decrease the burden on subjects.
1, 2.2.2	Text is added to clarify that the standard of care when discussing fluoropyrimidine with platinum-based combination chemotherapy regimens is the standard of care cytotoxic chemotherapy regimen.	This revision is made for clarification.
1.1	Remove paragraph referencing normal epithelia.	Based on a further interpretation of the data, there was insufficient evidence to support this statement.
Table continued on next page		

Section Number	Description of Change	Brief Rationale
5.1.2.4	Text added to indicate that dose re-escalation of capecitabine is permitted after the subject has completed 8 cycles or 8 doses of CAPOX if the investigator chooses to continue capecitabine.	To clarify that once oxaliplatin therapy is completed and if capecitabine is continued the dose may be increased as a monotherapy.
5.1.2.8 (Table 8)	Dose modifications for oxaliplatin related to neurotoxicity is updated to describe that for Grade 3 neurotoxicity > 7 days/persistent between treatments, discuss oxaliplatin discontinuation with Medical Monitor.	To allow discussion with Medical Monitor to determine if the subject's oxaliplatin treatment should be discontinued or continued.
5.4.5	Text revised to clarify that only clinically significant ECG findings, and not changes from baseline in ECG findings, should be recorded as an AE.	Clarification of language as not all changes from baseline are an AE.
5.5.2.1	Always Serious Adverse Events are now referred to as Important Medical Events and if an AE occurs that the sponsor determines to be an Important Medical Event, additional information on the event (e.g., investigator confirmation of seriousness, causality) will be requested.	Astellas has changed from "Always Serious Adverse Events" categorization to "Important Medical Events" to align with internal processes for medically important events.
6.3	Text is updated to clarify procedures if the study is prematurely terminated or suspended.	This revision is made for clarification.
7.8	Text is added to clarify that the analysis for the eCOA endpoints will be performed as the final analysis once OS is significant either at the interim OR or at final OS analysis; hence, there is no interim analysis planned for eCOA endpoints.	This revision is made for clarification.
8.1.1, 8.1.1.1	Text is added to provide details for the investigator's role in maintaining accurate source data.	Investigator's responsibilities are clarified.
Table continued on next page		

Section Number	Description of Change	Brief Rationale
8.2	Text updated to remove major protocol deviation criteria but note that major protocol deviations will be summarized at the end of the study.	This change is made to help clarify site and sponsor protocol deviation reporting requirements.
12.2.2	Text is added to describe how remote source data review will be used when needed.	This revision is made to address a process change related to source document review requirements.
12.9 (Table 10 [general footnotes])	Footnote added to clarify that:<br>• additional non-protocol-specific testing may be required per local regulations, and<br>• local and/or regional protocols or precautions for COVID-19 management should be followed as applicable	This revision is made for clarification.
Throughout	Minor administrative-type changes, e.g., typos, format, numbering, consistency throughout the protocol.	To provide clarifications to the protocol and to ensure complete understanding of study procedures.

14 COORDINATING INVESTIGATOR'S SIGNATURE

A Phase 3, Global, Multi-Center, Double-Blind, Randomized, Efficacy Study of Zolbetuximab (IMAB362) Plus CAPOX Compared with Placebo Plus CAPOX as First-line Treatment of Subjects with Claudin (CLDN)18.2-Positive, HER2-Negative, Locally Advanced Unresectable or Metastatic Gastric or Gastroesophageal Junction (GEJ) Adenocarcinoma

ISN/Protocol 8951-CL-0302

Version 5.0 Incorporating Substantial Amendment 4

18 Oct 2021

I have read all pages of this clinical study protocol for which Astellas is the sponsor. I agree that it contains all the information required to conduct this study.

Coordinating Investigator:

Signature: ______________________________ ______________________
<Insert name, department/affiliation, name of institution> Date (DD Mmm YYYY)

Printed Name: ______________________________

Address: ______________________________

____

15 SPONSOR’S SIGNATURES

Astellas Signatories

(Electronic signatures are attached at the end of the document)

PPD

13 PROTOCOL AMENDMENT SUMMARY OF CHANGES

Protocol 8951-CL-0302 A Phase 3, Global, Multi-Center, Double-Blind, Randomized, Efficacy Study of Zolbetuximab (IMAB362) Plus CAPOX Compared with Placebo Plus CAPOX as First-line Treatment of Subjects with Claudin (CLDN)18.2-Positive, HER2-Negative, Locally Advanced Unresectable or Metastatic Gastric or Gastroesophageal Junction (GEJ) Adenocarcinoma

Country-Specific Amendment 5 [Substantial] 18 Oct 2021

This amendment is considered to be substantial based on the criteria set forth in Article 10(a) of Directive 2001/20/EC of the European Parliament and the Council of the European Union.

Overall Rationale for the Amendment:

Reduction in the number of PFS events required for primary analysis as well as addition of key secondary endpoints for health-related quality of life questionnaires.

Summary of Changes Table:

**SUBSTANTIAL CHANGES**		
**Section Number**	**Description of Change**	**Brief Rationale**
IV, 2.1.2, 2.3.2, 5.7.3.1, 5.7.3.2, 7.4.2.2	Addition of health economics and outcomes research (HEOR) related key secondary endpoints, including physical function, Pain, and Global Health Score.	A key secondary endpoint for QOL measures has been added after FDA interaction in order to more specifically address the effect of zolbetuximab in gastric/GEJ cancer, which impacts the risk/benefit assessment.
IV, 5.1.5, 12.4	Clarify that a subject receiving oxaliplatin should not receive live vaccines.	Administration of live or live attenuated vaccines in patients immunocompromised by chemotherapeutic agents may result in serious or fatal infections.
IV, 2.2.1, 7.1, 7.4.1.1	The number of PFS events required for the interim analysis of overall survival is reduced from 344 to 300.	The number of required PFS events has been adjusted based on the enrollment and event accrual rates to maintain the timing of Primary Analysis with adequate power which is > 93%.
Table continued on next page		

**SUBSTANTIAL CHANGES**		
**Section Number**	**Description of Change**	**Brief Rationale**
IV, 7.2.2, 7.3, 7.4, 7.4.1.2, 7.4.2.1	The Per Protocol Set (PPS) has been removed from the protocol.	The PPS is defined as the subjects who do not meet predetermined study entry and treatment criteria as well as those with lack of imaging assessment.<br>The data from subjects meeting these criteria are unlikely to allow adequate assessment of potential impact on treatment benefit, possibly resulting in risk of bias. Therefore, the robustness of treatment benefit in Primary Endpoint will instead be assessed through sensitivity analyses applying different censoring rules.

**NONSUBSTANTIAL CHANGES**		
**Section Number**	**Description of Change**	**Brief Rationale**
II	Contact details for the clinical research contact and global clinical research contact are updated.	Contact details of sponsor personnel are updated based on changes to study personnel.
IV	Correct planned study period to end 2Q2023	To reflect the length of the planned study period
IV, V (Table 1 [footnote 1]), 2.2.1, 12.8 (Table 10 [footnote 1]).	Specify that CT scans and MRIs conducted as a part of a subject’s routine clinical management obtained prior to signing the informed consent form may be utilized for screening or baseline purposes.	Additional instructions are provided for clarity.
IV, V (Table 1 [footnote 5]), 5.1.1.2, 12.8 (Table 10 [footnote 5])	Text revised to clarify prophylactic antiemetic management.	To clarify the timing of oral and IV antiemetics.
Table continued on next page		

NONSUBSTANTIAL CHANGES		
**Section Number**	**Description of Change**	**Brief Rationale**
IV, V (Table 1 [footnotes 15 and16]), 3.2 (inclusion criteria #3 and #5), 5.5.9.1, 12.3, 12.8 (Table 10 [footnotes 15 and 16])	Contraceptive guidance is updated to clarify that requirements are applicable for 9 months after the final administration of oxaliplatin and 6 months after the final administration of all other study drugs.	To align with the FDA label for oxaliplatin use in ensuring adequate contraception and pregnancy testing
IV, 3.2 (inclusion criterion #17), 5.4.3	This inclusion criterion is updated to clarify that when multiple sample collections are done, the most recent sample collection with available results should be used for determination of eligibility.	This revision is made for clarification.
IV, 3.3 (exclusion criterion #1)	Subjects are not prohibited from participation if they received prior immunotherapy or other systemic anti-cancer therapies as long as it was completed at least 6 months prior to randomization.	Clarified the types of immunological therapies that are not prohibited.
V (Table 1 [general footnote]), 12.8 (Table 10 [general footnote])	Footnote added to clarify that in case subjects are unable to be evaluated in person on day 1 of each cycle, the site will contact the subject by phone for a safety assessment at the time the next visit would be due.	This revision is made for clarification.
V (Table 1 [footnotes 9, 15, 16]), 5.4.4, 12.8 (Table 10 [footnotes 9, 15, 16])	Clarify that the urine and serum pregnancy tests, physical examination, weight and ECOG performance status assessments can be completed up to 48 hours prior to zolbetuximab/placebo administration.	To provide a window for pre-treatment testing to decrease subject burden and align with standard subject flow for pre-treatment testing.
Table continued on next page		

**NONSUBSTANTIAL CHANGES**		
**Section Number**	**Description of Change**	**Brief Rationale**
V (Table 1 [footnote 10]), 12.8 (Table 10 [footnote 10])	The vital sign observation period for subsequent zolbetuximab/placebo infusions is updated to include a reduction of observation time from every 30 minutes to every 60 minutes and a reduction of intervals for assessing vital signs in some situations.	This revision is made for clarification and to decrease the burden on subjects.
1, 2.2.2	Text is added to clarify that the standard of care when discussing fluoropyrimidine with platinum-based combination chemotherapy regimens is the standard of care cytotoxic chemotherapy regimen.	This revision is made for clarification.
1.1	Remove paragraph referencing normal epithelia.	Based on a further interpretation of the data, there was insufficient evidence to support this statement.
5.1.2.4	Text added to indicate that dose re-escalation of capecitabine is permitted after the subject has completed 8 cycles or 8 doses of CAPOX if the investigator chooses to continue capecitabine.	To clarify that once oxaliplatin therapy is completed and if capecitabine is continued the dose may be increased as a monotherapy.
5.1.2.8 (Table 8)	Dose modifications for oxaliplatin related to neurotoxicity is updated to describe that for Grade 3 neurotoxicity > 7 days/persistent between treatments, discuss oxaliplatin discontinuation with Medical Monitor.	To allow discussion with Medical Monitor to determine if the subject’s oxaliplatin treatment should be discontinued or continued.
5.4.5	Text revised to clarify that only clinically significant ECG findings, and not changes from baseline in ECG findings, should be recorded as an AE.	Clarification of language as not all changes from baseline are an AE.
Table continued on next page		

NONSUBSTANTIAL CHANGES		
**Section Number**	**Description of Change**	**Brief Rationale**
5.5.2.1	Always Serious Adverse Events are now referred to as Important Medical Events and if an AE occurs that the sponsor determines to be an Important Medical Event, additional information on the event (e.g., investigator confirmation of seriousness, causality) will be requested.	Astellas has changed from "Always Serious Adverse Events" categorization to "Important Medical Events" to align with internal processes for medically important events.
6.3	Text is updated to clarify procedures if the study is prematurely terminated or suspended.	This revision is made for clarification.
7.8	Text is added to clarify that the analysis for the eCOA endpoints will be performed as the final analysis once OS is significant either at the interim OR or at final OS analysis; hence, there is no interim analysis planned for eCOA endpoints.	This revision is made for clarification.
8.1.1, 8.1.1.1	Text is added to provide details for the investigator's role in maintaining accurate source data.	Investigator's responsibilities are clarified.
8.2	Text updated to remove major protocol deviation criteria but note that major protocol deviations will be summarized at the end of the study.	This change is made to help clarify site and sponsor protocol deviation reporting requirements
12.2.2	Text is added to describe how remote source data review and/or verification will be used when needed.	This revision is made to address a process change related to source document review requirements.
Table continued on next page		

**NONSUBSTANTIAL CHANGES**		
**Section Number**	**Description of Change**	**Brief Rationale**
12.8 (Table 10 [general footnotes])	Footnote added to clarify that:<br>• additional non-protocol-specific testing may be required per local regulations, and<br>• local and/or regional protocols or precautions for COVID-19 management should be followed as applicable	This revision is made for clarification.
Throughout	Minor administrative-type changes, e.g., format, numbering, consistency, are made throughout the protocol.	To provide clarifications to the protocol and to ensure complete understanding of study procedures.

14 COORDINATING INVESTIGATOR'S SIGNATURE

A Phase 3, Global, Multi-Center, Double-Blind, Randomized, Efficacy Study of Zolbetuximab (IMAB362) Plus CAPOX Compared with Placebo Plus CAPOX as First-line Treatment of Subjects with Claudin (CLDN)18.2-Positive, HER2-Negative, Locally Advanced Unresectable or Metastatic Gastric or Gastroesophageal Junction (GEJ) Adenocarcinoma

ISN/Protocol 8951-CL-0302

Version 6.0 [CN] Incorporating Country-specific Substantial Amendment 5 for China

18 Oct 2021

I have read all pages of this clinical study protocol for which Astellas is the sponsor. I agree that it contains all the information required to conduct this study.<br>**Coordinating Investigator:**
Signature: ____________________ ____________<br>*<Insert name, department/affiliation, name of institution>* Date (DD Mmm YYYY)<br><br>Printed Name: ____________________<br><br>Address: ____________________<br>____________________

15 SPONSOR'S SIGNATURES

Astellas Signatories

(Electronic signatures are attached at the end of the document)

PPD	Development Medical Science - Oncology
PPD	Biostatistics

13 ATTACHMENT 1: COUNTRY-SPECIFIC NONSUBSTANTIAL AMENDMENT 2 FOR CHINA

I. The purpose of this amendment is:

**Non-Substantial Changes**
**1. Adjust Dosing Sequence Language**
DESCRIPTION OF CHANGE:
Relaxed requirement around dosing sequence to say that zolbetuximab/placebo should be administered before chemotherapy.
RATIONALE:
There is no significant impact to subject safety anticipated with sequence of dosing administration.
**2. Update to Dose Modification Tables**
DESCRIPTION OF CHANGE:
Clarify that dose modification must be applied regardless of relationship to the drug.
RATIONALE:
Language added to assist in understanding of application of dose modifications irrespective of causality.
**3. Clarifications to Cytokine/Chemokines/Tryptase Collection**
DESCRIPTION OF CHANGE:
Added clarification that cytokines/chemokines/tryptase need to be collected centrally.
RATIONALE:
To assist in understanding of required lab study procedures when infusion-related reaction criteria is met.
**4. Changed Lab Collection Window Before Cycle 1 Day 1**
DESCRIPTION OF CHANGE:
Changed lab collection on Cycle 1 Day 1 48-hour window to start at Cycle 1 Day 1.
RATIONALE:
This change is made to define a clinically reasonable window for pre-dosing lab collection.

**5. Update Adverse Event and Conmed Collection to Begin at Full Informed Consent Form**
DESCRIPTION OF CHANGE:
Defined adverse event (AE) collection to begin at the time of full informed consent form (ICF).
RATIONALE:
Clarification for AE and Conmed collection to begin at time of full ICF signature as opposed to Partial Screening ICF signature.
**6. Updated Electronic Clinical Outcomes Assessment Visit Language**
DESCRIPTION OF CHANGE:
Clarified that electronic clinical outcomes assessments (eCOAs) only need to be collected for zolbetuximab/placebo visits if CAPOX is discontinued at a different timepoint.
RATIONALE:
To clarify study eCOA assessments are only collected on zolbetuximab/placebo treatment visits, end of treatment, and 30 and 90 Day Safety Follow-up Visits.
**7. Updated Window for the 30-Day Safety Follow-up Visit**
DESCRIPTION OF CHANGE:
Updated window for 30-Day Safety Follow-up Visit from + 7 days to ± 7 days.
RATIONALE:
To establish a larger visit window for the 30 Day Safety Follow-up Visit and align with the visit window for the 90 Day Safety Follow-up Visit.
**8. Adjusted Restrictions Around CAPOX Dose Re-escalation**
DESCRIPTION OF CHANGE:
Adjusted restrictions around CAPOX dose re-escalation after dose adjustment due to drug-related AEs.
RATIONALE:
To indicate CAPOX dose re-escalation is not recommended if the AE was related to CAPOX.

**9. Clarified Discontinuation Criteria and Dosing Guidance**
DESCRIPTION OF CHANGE:
Clarified discontinuation criteria and dosing guidance for zolbetuximab/placebo and chemotherapy if greater than 28 days from next planned dose passes.
RATIONALE:
To assist in understanding of study requirement for discontinuation related to dose interruption duration.
**10. Clarified the Maximum Number of Oxaliplatin Doses Allowed**
DESCRIPTION OF CHANGE:
Added language to make the maximum number of oxaliplatin doses allowed more clear.
RATIONALE:
To emphasize the maximum number of oxaliplatin treatments per oxaliplatin SmPC.
**11. Added Infusion Rate Adjustment to Table 2**
DESCRIPTION OF CHANGE:
Added instruction to reduce the infusion rate in the guidance for nausea/vomiting management.
RATIONALE:
Additional AE management guidance for common AEs observed during study drug infusion.
**12. Clarified Zolbetuximab Infusion Timing**
DESCRIPTION OF CHANGE:
Clarified timing of zolbetuximab infusion.
RATIONALE:
Removed maximum infusion time frame to align with revised zolbetuximab stability requirements in Investigator’s Brochure version 5.0.

**13. Added Appendix 12.8**
DESCRIPTION OF CHANGE:
Added Appendix 12.8 to detail accepted alternate approaches during times when the standard procedures cannot be followed.
RATIONALE:
To outline procedures to be prioritized and alternate methods of assessing safety and efficacy in the event of disruptions to trial operations at study sites.
**14. Adjusted Vaccine Restrictions**
DESCRIPTION OF CHANGE:
Added language regarding potential COVID-19 vaccination.
RATIONALE:
Language added to provide guidance for cases where live vaccine is needed for COVID-19 prevention.
**15. Adjusted Oxaliplatin Dose Modification for Associated Neurotoxicity**
DESCRIPTION OF CHANGE
Adjusted dose modification after acute laryngopharyngeal dysesthesia.
RATIONALE
For clarity on AE management.
**16. Adjusted Sponsor Preapproval Requirements for Biopsies**
DESCRIPTION OF CHANGE
Adjusted language to indicate that Sponsor preapproval is requested for biopsies performed for the sole purpose of determining study eligibility.
RATIONALE
To align Section 5.7.1 and the Synopsis.

**17. Adjusted Language Around Placebo Infusion**
DESCRIPTION OF CHANGE
Provided additional language around the sodium chloride injection for use as placebo.
RATIONALE
Clarify use of 0.9% sodium chloride injection to be used for both treatment arms.
**18. Minor Administrative-type Changes**
DESCRIPTION OF CHANGE:
Include minor administrative-type changes (e.g., typos, format, numbering and consistency throughout the protocol).
RATIONALE:
To provide clarifications to the protocol and to ensure complete understanding of study procedures.

II. Amendment Summary of Changes:

**IV Synopsis, Study Design Overview**<br>*2.2.1 Study Design*
WAS:
<u>*Screening:*</u><br>The Screening period is 45 days from informed consent form (ICF) signature.<br><br>AND<br><br>An optional partial screening ICF is available to allow central testing of tissue for CLDN18.2 only.
IS AMENDED TO:
<u>*Screening:*</u><br>The Screening period is 45 days from **full main** informed consent form (ICF) signature.<br><br>AND<br><br>An optional partial screening ICF ~~is~~ **may be** available to allow central testing of tissue for CLDN18.2 only.

IV Synopsis, Study Design Overview
2.2.1 Study Design

WAS:

Treatment Period:
Subjects will also receive up to 8 treatments of CAPOX treatment. Oxaliplatin is administered on day 1 of each cycle, whereas capecitabine is taken twice daily on days 1 through 14. After 8 treatments, subjects may continue to receive capecitabine taken twice daily on days 1 through 14 of each cycle at the investigator’s discretion until the subject meets study treatment discontinuation criteria.

IS AMENDED TO:

Treatment Period:
Subjects will also receive up to 8 treatments of CAPOX treatment. Oxaliplatin is administered on day 1 of each cycle, whereas capecitabine is taken twice daily on days 1 through 14. After **a maximum of** 8 treatments **of oxaliplatin**, subjects may continue to receive capecitabine taken twice daily on days 1 through 14 of each cycle at the investigator’s discretion until the subject meets study treatment discontinuation criteria.

IV Synopsis, Investigational Product(s), Zolbetuximab/Placebo

WAS:

Placebo: Sites should use 0.9% Sodium Chloride Injection as placebo.

IS AMENDED TO:

Placebo: ~~Sites should use 0.9% Sodium Chloride Injection as placebo.~~ **0.9% Sodium Chloride Injection will be used for placebo treatment arm as a placebo infusion solution.**

IV Synopsis, Investigational Product(s), Dosing Schedule

WAS:

Zolbetuximab/placebo to be administered after antiemetic premedication but prior to CAPOX.

IS AMENDED TO:

Zolbetuximab/placebo ~~to~~ **should** be administered after antiemetic premedication but prior to CAPOX.

**IV Synopsis, Investigational Product(s), Mode of Administration**
WAS:
Intravenous infusion of zolbetuximab/placebo as a minimum 2-hour infusion. It is recommended that zolbetuximab/placebo infusion not exceed 6 hours from start of infusion.<br>Intravenous infusion may be slowed or interrupted to manage toxicity. Please refer to Pharmacy Manual and Infusion Guidelines for more detailed information, if zolbetuximab/placebo infusion cannot be completed.
IS AMENDED TO:
Intravenous infusion of zolbetuximab/placebo as a minimum 2-hour infusion. ~~It is recommended that zolbetuximab/placebo infusion not exceed 6 hours from start of infusion.~~<br>Intravenous infusion may be slowed or interrupted to manage toxicity. Please refer to Pharmacy Manual and Infusion Guidelines for more detailed information~~, if zolbetuximab/placebo infusion cannot be completed~~.

**IV Synopsis, Other Product(s), Oxaliplatin**
WAS:
CAPOX is to be administered after zolbetuximab/placebo infusion.
IS AMENDED TO:
CAPOX ~~is to~~ **should** be administered after zolbetuximab/placebo infusion.

**IV Synopsis, Other Product(s), Oxaliplatin**
WAS:
130 mg/m^2 intravenous infusion on day 1 of each cycle over 2 hours (or longer per institutional standard of care) for up to 8 treatments.
IS AMENDED TO:
130 mg/m^2 intravenous infusion on day 1 of each cycle over 2 hours (or longer per institutional standard of care) for ~~up to~~ **a maximum of** 8 treatments.

**IV Synopsis, Other Product(s), Antiemetic Pre-medications**
WAS:
• All antiemetic premedication should be given at minimum 30 minutes prior to zolbetuximab/placebo treatment.

IS AMENDED TO:
• All antiemetic premedication should be ~~given~~ **initiated** at **a** minimum **of** 30 minutes prior to zolbetuximab/placebo treatment.

**IV Synopsis, Concomitant Medication Restrictions or Requirements, Prohibited Concomitant Treatment**
WAS:
• Live vaccines should be avoided during the treatment period in which subject is receiving capecitabine and up to 6 months after final capecitabine dose.
IS AMENDED TO:
• Live vaccines should be avoided during the treatment period in which subject is receiving capecitabine and up to 6 months after final capecitabine dose. **In cases where a live vaccine is needed for COVID-19 prevention, please contact the Medical Monitor for discussion.**

**IV Synopsis, Concomitant Medication Restrictions or Requirements, Cautionary Concomitant Treatment**
WAS:
The following should be avoided or used with caution and closely monitored during CAPOX administration:<br>• CytochromeP450 (CYP) 2C9 substrates (Subjects taking coumarin-derivative anticoagulants concomitantly with capecitabine should have PT/INR monitored regularly and anticoagulant dose adjusted accordingly).<br>• Anti-epileptic medications (e.g. phenobarbital, phenytoin and primidone)<br>• Medications known to prolong the QT or QTc interval (refer to https://www.crediblemeds.org for a list of these medications)
IS AMENDED TO:
The following should be avoided or used with caution and closely monitored during ~~CAPOX~~ **capecitabine** administration:<br>• CytochromeP450 (CYP) 2C9 substrates (Subjects taking coumarin-derivative anticoagulants concomitantly with capecitabine should have PT/INR monitored regularly and anticoagulant dose adjusted accordingly).<br>• Anti-epileptic medications (e.g. phenobarbital, phenytoin and primidone)<br>**The following should be avoided or used with caution and closely monitored during oxaliplatin administration:**<br>• Medications known to prolong the QT or QTc interval (refer to https://www.crediblemeds.org for a list of these medications)

**IV Synopsis, Study Treatment Discontinuation Criteria**
WAS:
• Subject has a delay of zolbetuximab/placebo and both components CAPOX for > 28 days from when the next zolbetuximab/placebo and both components CAPOX treatment was scheduled to be administered.
IS AMENDED TO:
• Subject has a delay of zolbetuximab/placebo and both components **of** CAPOX for > 28 days from when the next zolbetuximab/placebo and both components **of** CAPOX treatment was scheduled to be ~~administered~~ **begin (> 49 days from when the last dose of zolbetuximab/placebo and both components of CAPOX treatment began).**

V Schedule of Assessments

WAS:

VISIT	Screening[1]	Study Treatment Period (Each Cycle = approximately 21 Days)						Follow-up Period				
		**Cycles 1-8** Zolbetuximab/Placebo + CAPOX			**Cycle 9+** Zolbetuximab/Placebo + Capecitabine (at investigator discretion)			Study Treatment Discontinuation Visit[18]	30-Day Safety Follow-up Visit(s)[19]	90-Day Safety Follow-up Visit(s)[20]	Post-treatment Follow-up Period[21]	Long-term and Survival Follow-up Periods[22]
**Day**		1	2-14	15-21	1	2-14	15-21					
**Visit Window (calendar days)**	-45 to -1	+7*	(no visit)	(no visit)	+7	(no visit)	(no visit)	+7	+7	±7	±7	±14
Informed Consent	X											
CLDN18.2 Tumor Sample[2]	X											
Biopsy (if applicable)[2]	X											
Medical and Disease History	X											
Confirmation of Inclusion/Exclusion Criteria[3]	X											
Randomization[4]		X										
**Treatments**												
Antiemetic Pretreatment[5]		X			X							
Zolbetuximab/Placebo[6]		X			X							
Post-infusion Observation Period[7]		X			X							
Oxaliplatin (CAPOX[8])		X										
Capecitabine (CAPOX[8])		X	X		X	X						
**Safety Assessments**												
Physical Examination[9]	X	X			X			X	X			
Weight[9]	X	X			X			X	X			
Vital Signs[10]	X	X			X			X	X			
ECOG Performance Status[9]	X	X			X			X				
12-lead ECG[11]	X	X	If clinically indicated and/or per local requirements					X	X			
**Image Assessment[12]**	X[1]	Every 9 weeks ±7 days from C1D1 for the first 54 weeks and then every 12 weeks ±7 days thereafter										
Table continued on next page												

**Subject Contact**												X
**Laboratory Tests**												
Biochemistry[13]	X	X			X			X	X			
TSH and Free T4[13]	X	If clinically indicated						X				
Hematology[13]	X	X			X			X	X			
Coagulation Parameters (PT, PTT and INR)[14]	X	If clinically indicated										
Urinalysis[13]	X	X			X			X	X			
Serum Pregnancy Test[15]	X	If clinically indicated and/or per local requirements										
Urine Pregnancy Test[16]		X			X			X	X			
DPD testing per local requirements	X											
Cytokine/Chemokine and/or Tryptase		If clinically indicated										
**Electronic Clinical Outcomes Assessments (eCOA)**												
HRQoL[17]	X	X			X			X	X	X		
Health Resource Utilization (HRU)[17]		X			X			X	X	X		
**Sampling**												
Pharmacokinetics of Zolbetuximab (Serum)[23]		X			X				X	X		
Antidrug-Antibodies (ADA) for Immunogenicity[24]		X			X				X	X		
**Concomitant Medication[25]**	X	X			X			X	X	X		
**AE/SAE[26]**	X	X			X			X	X	X		

ADA: antidrug antibody; AE: adverse event; βhCG: beta human chorionic gonadotropin; C1D1: Cycle 1 Day 1; CAPOX: capecitabine and oxaliplatin; CLDN: claudin; CT: computerized tomography; DPD: dihydropyrimidine dehydrogenase; eCOA: electronic Clinical Outcomes Assessment; ECG: electrocardiogram;
ECOG: Eastern Cooperative Oncology Group; eCRF: electronic case report form; FFPE: formalin fixed paraffin embedded; HRQoL: health-related quality of life; HRU: Health Resource Utilization; ICF: informed consent form: INR: international normalized ratio; IRC: independent review committee; IRR: infusion-related reaction; IRT: interactive response technology; IV: intravenous; MRI: magnetic resonance imaging; OS: overall survival; PD: progressive disease; PFS: progression free survival; PFS2: progression free survival following subsequent anticancer treatment; PGx: pharmacogenomics; PT: prothrombin time; PTT: partial thromboplastin time; RECIST: Response Evaluation Criteria In Solid Tumors; SAE: serious adverse event; T4: thyroxine; TSH: thyroid stimulating hormone

* +7 calendar day visit window does not apply to C1D1.

1. Screening: The Screening period is 45 days from ICF signature. Retesting of lab values is allowed within the 45-day Screening period. Re-screening outside the 45-day window under a new subject number may be allowed once and upon discussion with the medical monitor.
    - Optional partial screening: A partial screening ICF is available for central testing of tissue for CLDN18.2 only.
    - Laboratory testing:
        - Eligibility can be determined based on central and/or local laboratory testing; **however**:
            - The most recent laboratory data must be used to confirm the subject's eligibility.
            - Central labs must be collected and submitted to the central laboratory during the Screening period.
            - If retesting of lab values is necessary to confirm eligibility, local labs can be used without requiring additional sample collection for central laboratory submission.

- The screening labs used to determine eligibility should be collected within 14 days prior to randomization.
- Radiologic imaging used to confirm eligibility must be conducted within 28 days prior to randomization.

2. CLDN18.2 Testing: FFPE tumor tissue will be collected for central testing to determine CLDN18.2 status. Archival tumor tissue from the primary tumor (gastric or GEJ) is preferred. If primary tumor tissue is not available, tumor tissue from a metastatic site (excluding bone metastasis) may be used. Five FFPE unstained slides are required as allowed per local policy. The slides should be freshly cut from the FFPE block within the time frame described in the laboratory manual. If the specimen is insufficient or unavailable, a biopsy may be performed to obtain primary tumor tissue (preferred) or tumor tissue from metastatic site (excluding bone metastasis). Sponsor pre-approval is required when the sole purpose of the biopsy procedure is to assess eligibility for this study. See [Section 5.7.1 Tumor Tissue Samples].
3. Confirmation of Inclusion/Exclusion Criteria must be completed prior to randomization.
4. Randomization: After confirmation of eligibility, the blinded site user will perform the randomization IRT transaction. The unblinded pharmacist/designee will be notified by the IRT system about the randomly assigned treatment. Randomization may be performed prior to C1D1. Details of infusion preparation and storage requirements are defined in the Pharmacy Manual and Infusion Guidelines.
5. Antiemetic Pretreatment: Prophylactic antiemetics should be given according to institutional standard of care, published guidelines and the respective product package insert(s). All antiemetic premedication should be given at minimum 30 minutes prior to treatment. For further details, see [Section 5.1.1.2 Antiemetics].

Footnotes continued on next page

6. Zolbetuximab/placebo will be administered as a minimum 2-hour intravenous infusion every 3 weeks starting on C1D1. It is recommended that zolbetuximab/placebo infusion not exceed 6 hours from start of infusion. Please refer to Pharmacy Manual and Infusion Guidelines for more detailed information, if zolbetuximab/placebo cannot be completed. Zolbetuximab/placebo should be administered prior to CAPOX. For further details, see [Section 5.1.1.1 Zolbetuximab/Placebo].
7. Post-infusion Observation Period: Following the first dose of zolbetuximab/placebo on C1D1, the subject must be observed for 2 hours post zolbetuximab/placebo infusion. The post-infusion observation period can be conducted during the CAPOX administration. If any ≥ grade 2 AEs are observed during infusion or during the post-infusion observation period, subsequent zolbetuximab/placebo infusion times should be extended and subjects should continue to be observed for 2 hours post zolbetuximab/placebo infusion. If the subject does not develop any grade ≥ 2 AEs, the subject should be observed for 1 hour post-infusion for their subsequent zolbetuximab/placebo infusions. The subject should be instructed to notify site personnel if they develop any AEs during this observation time period. In the event of an IRR with features of anaphylaxis (regardless of grade) or grade 3 or 4 IRR, blood samples for cytokine/chemokine panel and serum total tryptase level (levels typically peak within 3 hours after the onset of symptoms) should be collected once the subject has stabilized, for shipment to the central laboratory. See Observation Period following zolbetuximab/placebo [Section 5.4.2] for further details.
8. CAPOX is a combination of oxaliplatin intravenous infusion and capecitabine tablets and will be administered starting at C1D1 for up to 8 treatments. See [Section 5.1.1.3].
9. Physical Exam: should include height (at Screening only), weight and ECOG performance status. A full physical exam is required at Screening. The physical exam only needs to be repeated on C1D1 if clinically significant changes from screening are observed (in the opinion of the investigator). Targeted (symptom driven) physical exams should be conducted every 3 weeks on day 1 of each cycle. For further details, see [Section 5.4.4 Physical Examination]
10. Vital signs (pulse, blood pressure, temperature) should be taken during every visit at the following time points (see [Section 5.4.1 Vital Signs]):
    - Predose at every visit
    - C1D1: Every 30 (±10) minutes during zolbetuximab/placebo infusion
    - Subsequent zolbetuximab/placebo infusions: every 60 (±10) minutes during zolbetuximab/placebo infusions if the subject did not develop any ≥ grade 2 AEs during the C1D1 zolbetuximab/placebo infusion or Post-infusion Observation Period.
    - Every 60 (±10) minutes post zolbetuximab/placebo infusion during the Post-infusion Observation Period (for 1 or 2 hours. See footnote 7)

- Unscheduled if clinically indicated

11. ECGs: ECGs will be locally assessed. When collected on the same day, ECG should be collected prior to pharmacokinetic samples. For further details, see [Section 5.4.5 Electrocardiogram]. A single ECG will be performed at the following time points:
    - Screening
    - Up to 48 hours prior to every oxaliplatin infusion (before any antiemetic treatment administration)
    - Up to 6 hours following completion of every oxaliplatin infusion
    - Zolbetuximab/placebo Discontinuation Visit
    - Zolbetuximab/placebo 30-day Follow-up Visit
    - If clinically indicated or per local requirements

Footnotes continued on next page

12. Imaging Assessments: Radiologic imaging will be evaluated at Screening (must be conducted within 28 days prior to randomization) and every 9 weeks (± 7 days) counting from C1D1 for the first 54 weeks and then every 12 weeks (± 7 days) thereafter until subject develops radiological disease progression per RECIST 1.1 by IRC or starts other systemic anticancer treatment, whichever comes earlier. Imaging schedule should be maintained regardless of treatment delay. Imaging will include CT scans with contrast of the thorax, abdomen, and pelvis. If CT scan with contrast is medically not feasible, MRI may be used for imaging. Bone scans (or focal X-ray) or brain imaging should be performed if metastatic disease in bone or brain is suspected, respectively. The same mode of imaging should be utilized throughout the study unless medical necessity requires a change. For randomized subjects, screening imaging should be sent to the central imaging vendor no later than at the time of submission of the first on-treatment imaging. All imaging acquired post randomization will be sent to the central imaging vendor within 7 days of scanning for the blinded independent central assessment of radiological tumor response based on RECIST 1.1. The investigator should make every effort to immediately submit radiologic assessments for IRC review when PD is suspected. See [Section 5.3 Efficacy Assessments]. Refer to Imaging Acquisition Guidelines for further detail on scan modality and contrast options.
13. Laboratory Assessments: See [Section 5.4.3 Laboratory Assessments] for list of laboratory assessments. Laboratory tests must be sent to the central laboratory for analysis. For screening/eligibility laboratory assessments, see footnote number 1.
    - Laboratory test results (central or local) will be reviewed by the investigator prior to any study treatment. Clinical significance of out-of-range laboratory findings is to be determined and documented by the investigator/sub-investigator who is a qualified physician.
    - Local laboratory results may be used for treatment decisions; however, central laboratory samples should also be drawn per protocol and sent to the central laboratory unless otherwise approved by the sponsor.
    - From cycle 2 onwards labs may be collected up to 48 hours prior to study treatment.
    - Holidays and weekends should be taken into account when scheduling these blood draws.
    - Additional assessments may be done centrally or locally to monitor AEs or as clinically indicated.
14. Coagulation (PT, PTT and INR): Coagulation tests should be done at Screening and during study treatment period if clinically indicated. Local or central lab results may be used to confirm eligibility. Ongoing evaluation should be continued for subjects who are receiving therapeutic anticoagulation according to local standard of care. See [Section 5.4.3 Laboratory Assessments].

15. Serum Pregnancy Test: Serum pregnancy tests will be collected for female subjects of childbearing potential only. Serum pregnancy tests collected at Screening, during study treatment period and if clinically indicated or per local requirements. (Note: For Screening, subjects with elevated serum βHCG and a demonstrated non-pregnant status through additional testing are eligible.)
16. Urine Pregnancy Test: Urine pregnancy tests will be collected for female subjects of childbearing potential only. Local urine pregnancy tests to be performed during the treatment period every 3 weeks on day 1 of each cycle and at the zolbetuximab/placebo Study Treatment Discontinuation and 30-day Safety Follow-up Visits. Additional urine pregnancy testing for 6 months after the final study treatment administration may be conducted based on local requirements.
17. HRQoL and HRU questionnaires: eCOA questionnaires should be administered at Screening (except for HRU), on day 1 of each cycle (or up to 48 hours) before any antiemetic or drug treatment and before the disease status is discussed with the subject. For subjects with low literacy or situations where required translation is not available, please contact the sponsor for further guidance.
18. Study Treatment Discontinuation Visit (End of Study Treatment): The Study Treatment Discontinuation Visit will take place ≤ 7 days following the decision to discontinue study treatment (zolbetuximab/placebo and CAPOX [both components]). If zolbetuximab/placebo and CAPOX (both components) are discontinued on a different day, subjects will have separate Study Treatment Discontinuation Visits following each treatment's discontinuation. Laboratory tests must be sent to the central laboratory for analysis.

Footnotes continued on next page

19. 30-day Safety Follow-up Visit: A 30-day Safety Follow-up Visit should occur 30 days after the last dose of zolbetuximab/placebo and will include the assessments as shown the in the Schedule of Assessments above. A 30-day Safety Follow-up Visit should occur 30 days after the last dose of CAPOX (both components) and may be conducted by phone if the subject is unable to visit the site and will require contact for AE/SAE collection only.
20. 90-day Safety Follow-up Visits: A 90-day Safety Follow-up Visit should occur 90 days after the last dose of zolbetuximab/placebo and will include the assessments as shown the in the Schedule of Assessments above. A 90-day Safety Follow-up Visit should occur 90 days after the last dose of CAPOX (both components) and may be conducted by phone if the subject is unable to visit the site and will require contact for AE/SAE collection only.
21. Post-treatment Follow-up: if a subject discontinues all study treatments (zolbetuximab/placebo and both components of CAPOX) prior to IRC-confirmed radiological disease progression, the subject will enter the Post-treatment Follow-up Period and continue to undergo imaging assessments every 9 weeks (±7 days) (or every 12 weeks [±7 days] if subjects has been on study over 54 weeks) until radiologic disease progression (i.e., PFS) or the subject starts subsequent anticancer treatment, whichever occurs earlier. If study treatments (zolbetuximab/placebo and both components of CAPOX) are discontinued due to PD, the subject will enter the Long-term and Survival Follow-up Period.
22. Long-term and Survival Follow-up Period: Following disease progression on first-line treatment or start of subsequent anticancer treatment, subjects will be followed in the Long-term and Survival Follow-up Period per institutional guidelines, but not less than every 12 weeks. Radiologic imaging will be done per standard of care and read locally until PFS2 is documented. Survival Follow-up Period will continue until death (from any cause). All post-progression details including subsequent anticancer treatment and date and site of progression will be recorded on the eCRF. Subject contact by phone or other remote methods is sufficient during Long-term and Survival Follow-up.
23. Pharmacokinetics: Serum samples for zolbetuximab/placebo will be taken at the below time points and sent to the central laboratory. The date and time of each blood sample collection will be recorded to the nearest minute.
    - Cycle 1 Day 1: End of zolbetuximab/placebo infusion
    - Cycle 2 Day 1: Predose
    - Cycle 3 Day 1: End of zolbetuximab/placebo infusion
    - Predose on Day 1 of Cycles 5, 9, 13 and 17
    - Zolbetuximab/placebo 30-Day Safety Follow-up visit

- Zolbetuximab/placebo 90-Day Safety Follow-up visit
- Unscheduled pharmacokinetic blood samples may be taken at any time during the study to evaluate drug exposure following a safety event

Pharmacokinetic Sampling Window:
- Predose: within 60 minutes prior to dosing
- End of Infusion: within 10 minutes after the end of the infusion

24. ADA: Blood samples (Serum) for ADA will be taken at the below time points and sent to the central laboratory.
    - Cycle 1 Day 1: Predose
    - Cycle 2 Day 1: Predose
    - Predose on Day 1 of Cycles 5, 9, 13 and 17
    - Zolbetuximab/placebo 30-Day Safety Follow-up visit
    - Zolbetuximab/placebo 90-Day Safety Follow-up visit

    ADA Sampling Window: Predose: within 60 minutes prior to dosing
25. Concomitant medications will be collected from the time of informed consent through 90 days following the last dose of study treatment.
26. AEs/SAEs: AEs and SAEs (regardless of causality) will be collected from the time of informed consent through 90 days following the last dose of study treatment. See [Section 5.5.5 Reporting of Serious Adverse Events].

IS AMENDED TO:

VISIT	Screening[1]	Study Treatment Period (Each Cycle = approximately 21 Days)						Follow-up Period				
		**Cycles 1-8** Zolbetuximab/Placebo + CAPOX			**Cycle 9+** Zolbetuximab/Placebo + Capecitabine (at investigator discretion)			Study Treatment Discontinuation Visit[18]	30-Day Safety Follow-up Visit(s)[19]	90-Day Safety Follow-up Visit(s)[20]	Post-treatment Follow-up Period[21]	Long-term and Survival Follow-up Periods[22]
**Day**		1	2-14	15-21	1	2-14	15-21					
**Visit Window (calendar days)**	-45 to -1	+7*	(no visit)	(no visit)	+7	(no visit)	(no visit)	+7	~~+~~±7	±7	±7	±14
Informed Consent	X											
CLDN18.2 Tumor Sample[2]	X											
Biopsy (if applicable)[2]	X											
Medical and Disease History	X											
Confirmation of Inclusion/Exclusion Criteria[3]	X											

Randomization[4]		X										
**Treatments**												
Antiemetic Pretreatment[5]		X			X							
Zolbetuximab/Placebo[6]		X			X							
Post-infusion Observation Period[7]		X			X							
Oxaliplatin (CAPOX[8])		X										
Capecitabine (CAPOX[8])		X	X		X	X						
**Safety Assessments**												
Physical Examination[9]	X	X			X			X	X			
Weight[9]	X	X			X			X	X			
Vital Signs[10]	X	X			X			X	X			
ECOG Performance Status[9]	X	X			X			X				
12-lead ECG[11]	X	X	If clinically indicated and/or per local requirements					X	X			
**Image Assessment[12]**	X[1]	Every 9 weeks ±7 days from C1D1 for the first 54 weeks and then every 12 weeks ±7 days thereafter										
Table continued on next page												
**Subject Contact**												X
**Laboratory Tests**												
Biochemistry[13]	X	X			X			X	X			
TSH and Free T4[13]	X	If clinically indicated						X				
Hematology[13]	X	X			X			X	X			
Coagulation Parameters (PT, PTT and INR)[14]	X	If clinically indicated										
Urinalysis[13]	X	X			X			X	X			
Serum Pregnancy Test[15]	X	If clinically indicated and/or per local requirements										
Urine Pregnancy Test[16]		X			X			X	X			
DPD testing per local requirements	X											
Cytokine/Chemokine and/or Tryptase		If clinically indicated										
**Electronic Clinical Outcomes Assessments (eCOA)**												
HRQoL[17]	X	X			X			X	X	X		
Health Resource Utilization (HRU)[17]		X			X			X	X	X		
**Sampling**												
Pharmacokinetics of Zolbetuximab (Serum)[23]		X			X				X	X		
Antidrug-Antibodies (ADA) for Immunogenicity[24]		X			X				X	X		
**Concomitant Medication[25]**	X	X			X			X	X	X		
**AE/SAE[26]**	X	X			X			X	X	X		

ADA: antidrug antibody; AE: adverse event; βhCG: beta human chorionic gonadotropin; C1D1: Cycle 1 Day 1; CAPOX: capecitabine and oxaliplatin; CLDN: claudin; CT: computerized tomography; DPD: dihydropyrimidine dehydrogenase; eCOA: electronic Clinical Outcomes Assessment; ECG: electrocardiogram; ECOG: Eastern Cooperative Oncology Group; eCRF: electronic case report form; FFPE: formalin fixed paraffin embedded; HRQoL: health-related quality of life; HRU: Health Resource Utilization; ICF: informed consent form: INR: international normalized ratio; IRC: independent review committee; IRR: infusion-related reaction; IRT: interactive

response technology; IV: intravenous; MRI: magnetic resonance imaging; OS: overall survival; PD: progressive disease; PFS: progression free survival; PFS2: progression free survival following subsequent anticancer treatment; PGx: pharmacogenomics; PT: prothrombin time; PTT: partial thromboplastin time; RECIST: Response Evaluation Criteria In Solid Tumors; SAE: serious adverse event; T4: thyroxine; TSH: thyroid stimulating hormone

* +7 calendar day visit window does not apply to C1D1.

1. Screening: The Screening period is 45 days from **full main** ICF signature. Retesting of lab values is allowed within the 45-day Screening period. Re-screening outside the 45-day window under a new subject number may be allowed once and upon discussion with the medical monitor.
   - Optional partial screening: A partial screening ICF ~~is~~ **may be** available for central testing of tissue for CLDN18.2 only.
   - Laboratory testing:
     - Eligibility can be determined based on central and/or local laboratory testing; **however**:
       - The most recent laboratory data must be used to confirm the subject's eligibility.
       - Central labs must be collected and submitted to the central laboratory during the Screening period.
       - If retesting of lab values is necessary to confirm eligibility, local labs can be used without requiring additional sample collection for central laboratory submission.
     - The screening labs used to determine eligibility should be collected within 14 days prior to randomization.
   - Radiologic imaging used to confirm eligibility must be conducted within 28 days prior to randomization.
2. CLDN18.2 Testing: FFPE tumor tissue will be collected for central testing to determine CLDN18.2 status. Archival tumor tissue from the primary tumor (gastric or GEJ) is preferred. If primary tumor tissue is not available, tumor tissue from a metastatic site (excluding bone metastasis) may be used. Five FFPE unstained slides are required as allowed per local policy. The slides should be freshly cut from the FFPE block within the time frame described in the laboratory manual. If the specimen is insufficient or unavailable, a biopsy may be performed to obtain primary tumor tissue (preferred) or tumor tissue from metastatic site (excluding bone metastasis). Sponsor pre-approval is required when the sole purpose of the biopsy procedure is to assess eligibility for this study. See [Section 5.7.1 Tumor Tissue Samples].
3. Confirmation of Inclusion/Exclusion Criteria must be completed prior to randomization.
4. Randomization: After confirmation of eligibility, the blinded site user will perform the randomization IRT transaction. The unblinded pharmacist/designee will be notified by the IRT system about the randomly assigned treatment. Randomization may be performed prior to C1D1. **If C1D1 cannot be performed within 5 calendar days from Randomization, please contact the Medical Monitor for discussion.** Details of infusion preparation and storage requirements are defined in the Pharmacy Manual and Infusion Guidelines.
5. Antiemetic Pretreatment: Prophylactic antiemetics should be given according to institutional standard of care, published guidelines and the respective product package insert(s). All antiemetic premedication should be ~~given~~ **initiated** at **a** minimum **of** 30 minutes prior to treatment. For further details, see [Section 5.1.1.2 Antiemetics].

Footnotes continued on next page

6. Zolbetuximab/placebo will be administered as a minimum 2-hour intravenous infusion every 3 weeks starting on C1D1. It is recommended that zolbetuximab/placebo infusion not exceed 6 hours from start of infusion. Please refer to Pharmacy Manual and Infusion Guidelines for more detailed information, if zolbetuximab/placebo infusion cannot be completed. Zolbetuximab/placebo should be administered prior to CAPOX. For further details, see [Section 5.1.1.1 Zolbetuximab/Placebo].
7. Post-infusion Observation Period: Following the first dose of zolbetuximab/placebo on C1D1, the subject must be observed for 2 hours post zolbetuximab/placebo infusion. The post-infusion observation period can be conducted during the CAPOX administration. If any ≥ grade 2 AEs are observed during infusion or during the post-infusion observation period, subsequent zolbetuximab/placebo infusion times should be extended and subjects should continue to be observed for 2 hours post zolbetuximab/placebo infusion. If the subject does not develop any grade ≥ 2 AEs, the subject should be observed for 1 hour post-infusion for their subsequent zolbetuximab/placebo infusions. The subject should be instructed to notify site personnel if they develop any AEs during this observation time period. In the event of an IRR with features of anaphylaxis (regardless of grade) or grade 3 or 4 IRR, blood samples for cytokine/chemokine panel and serum total tryptase level (levels typically peak within 3 hours after the onset of symptoms) should be collected once the subject has stabilized, for shipment to the central laboratory. See Observation Period following zolbetuximab/placebo **infusion** [Section 5.4.2] for further details.
8. CAPOX is a combination of oxaliplatin intravenous infusion and capecitabine tablets and will be administered starting at C1D1 for up to 8 treatments. See [Section 5.1.1.3].
9. Physical Exam: should include height (at Screening only), weight and ECOG performance status. A full physical exam is required at Screening. The physical exam only needs to be repeated on C1D1 if clinically significant changes from screening are observed (in the opinion of the investigator). Targeted (symptom driven) physical exams should be conducted every 3 weeks on day 1 of each cycle. For further details, see [Section 5.4.4 Physical Examination]
10. Vital signs (pulse, blood pressure, temperature) should be taken during every visit at the following time points (see [Section 5.4.1 Vital Signs]):
    - Predose at every visit
    - C1D1: Every 30 (±10) minutes during zolbetuximab/placebo infusion
    - Subsequent zolbetuximab/placebo infusions: every 60 (±10) minutes during zolbetuximab/placebo infusions if the subject did not develop any ≥ grade 2 AEs during the C1D1 zolbetuximab/placebo infusion or Post-infusion Observation Period.
    - Every 60 (±10) minutes post zolbetuximab/placebo infusion during the Post-infusion Observation Period (for 1 or 2 hours. See footnote 7)
    - Unscheduled if clinically indicated
11. ECGs: ECGs will be locally assessed. When collected on the same day, ECG should be collected prior to pharmacokinetic samples. For further details, see [Section 5.4.5 Electrocardiogram]. A single ECG will be performed at the following time points:
    - Screening
    - Up to 48 hours prior to every oxaliplatin infusion (before any antiemetic treatment administration)
    - Up to 6 hours following completion of every oxaliplatin infusion
    - Zolbetuximab/placebo Discontinuation Visit
    - Zolbetuximab/placebo 30-day Follow-up Visit
    - If clinically indicated or per local requirements

Footnotes continued on next page

12. Imaging Assessments: Radiologic imaging will be evaluated at Screening (must be conducted within 28 days prior to randomization) and every 9 weeks (± 7 days) counting from C1D1 for the first 54 weeks and then every 12 weeks (± 7 days) thereafter until subject develops radiological disease progression per RECIST 1.1 by IRC or starts other

systemic anticancer treatment, whichever comes earlier. Imaging schedule should be maintained regardless of treatment delay. Imaging will include CT scans with contrast of the thorax, abdomen, and pelvis. If CT scan with contrast is medically not feasible, MRI may be used for imaging. Bone scans (or focal X-ray) or brain imaging should be performed if metastatic disease in bone or brain is suspected, respectively. The same mode of imaging should be utilized throughout the study unless medical necessity requires a change. For randomized subjects, screening imaging should be sent to the central imaging vendor no later than at the time of submission of the first on-treatment imaging. All imaging acquired post randomization will be sent to the central imaging vendor within 7 days of scanning for the blinded independent central assessment of radiological tumor response based on RECIST 1.1. The investigator should make every effort to immediately submit radiologic assessments for IRC review when PD is suspected. See [Section 5.3 Efficacy Assessments]. Refer to Imaging Acquisition Guidelines for further detail on scan modality and contrast options.

13. Laboratory Assessments: See [Section 5.4.3 Laboratory Assessments] for list of laboratory assessments. Laboratory tests must be sent to the central laboratory for analysis. For screening/eligibility laboratory assessments, see footnote number 1.
    - Laboratory test results (central or local) will be reviewed by the investigator prior to any study treatment. Clinical significance of out-of-range laboratory findings is to be determined and documented by the investigator/sub-investigator who is a qualified physician.
    - Local laboratory results may be used for treatment decisions; however, central laboratory samples should also be drawn per protocol and sent to the central laboratory unless otherwise approved by the sponsor.
    - ~~From cycle 2 onwards l~~**L**abs may be collected up to 48 hours prior to study treatment.
    - Holidays and weekends should be taken into account when scheduling these blood draws.
    - Additional assessments may be done centrally or locally to monitor AEs or as clinically indicated.
14. Coagulation (PT, PTT and INR): Coagulation tests should be done at Screening and during study treatment period if clinically indicated. Local or central lab results may be used to confirm eligibility. Ongoing evaluation should be continued for subjects who are receiving therapeutic anticoagulation according to local standard of care. See [Section 5.4.3 Laboratory Assessments].
15. Serum Pregnancy Test: Serum pregnancy tests will be collected for female subjects of childbearing potential only. Serum pregnancy tests collected at Screening, during study treatment period and if clinically indicated or per local requirements. (Note: For Screening, subjects with elevated serum βHCG and a demonstrated non-pregnant status through additional testing are eligible.)
16. Urine Pregnancy Test: Urine pregnancy tests will be collected for female subjects of childbearing potential only. Local urine pregnancy tests to be performed during the treatment period every 3 weeks on day 1 of each cycle and at the zolbetuximab/placebo Study Treatment Discontinuation and 30-day Safety Follow-up Visits. Additional urine pregnancy testing for 6 months after the final study treatment administration may be conducted based on local requirements.
17. HRQoL and HRU questionnaires: eCOA questionnaires should be ~~administered~~ **completed by the subject** at Screening (except for HRU), on day 1 of each cycle (or up to 48 hours) before any antiemetic or drug treatment and before the disease status is discussed with the subject**, using the electronic tablet device provided. When completion by the subject is not possible, the questionnaires may be administered to the subject by site personnel using the electronic tablet device.** For subjects with low literacy or situations where required translation is not available, please contact the sponsor for further guidance.
18. Study Treatment Discontinuation Visit (End of Study Treatment): The Study Treatment Discontinuation Visit will take place ≤ 7 days following the decision to discontinue study treatment (zolbetuximab/placebo and CAPOX [both components]). If zolbetuximab/placebo and CAPOX (both components) are discontinued on a different day, subjects will have separate Study Treatment Discontinuation Visits following each treatment's discontinuation. Laboratory tests must be sent to the central laboratory for analysis. **HRQoL and HRU questionnaires are not required at CAPOX treatment discontinuation visit. A combined visit can be completed if zolbetuximab/placebo are discontinued on the same day.**

Footnotes continued on next page

19. 30-day Safety Follow-up Visit: A 30-day Safety Follow-up Visit should occur 30 days after the last dose of zolbetuximab/placebo and will include the assessments as shown ~~the~~ in the Schedule of Assessments above. A 30-day Safety Follow-up Visit should occur 30 days after the last dose of CAPOX (both components) and may be conducted by phone if the subject is unable to visit the site and will require contact for AE/SAE collection only. **HRQoL and HRU questionnaires are not required at CAPOX 30-day safety follow up visit. A combined visit can be completed if zolbetuximab/placebo and both components of CAPOX are discontinued on the same day and HRQoL and HRU questionnaires should be completed for a combined visit.**
20. 90-day Safety Follow-up Visits: A 90-day Safety Follow-up Visit should occur 90 days after the last dose of zolbetuximab/placebo and will include the assessments as shown ~~the~~ in the Schedule of Assessments above. A 90-day Safety Follow-up Visit should occur 90 days after the last dose of CAPOX (both components) and may be conducted by phone if the subject is unable to visit the site and will require contact for AE/SAE collection only. **HRQoL and HRU questionnaires are not required at CAPOX 90-day safety follow up visit. A combined visit can be completed if zolbetuximab/placebo and both components of CAPOX are discontinued on the same day and HRQoL and HRU questionnaires should be completed for a combined visit.**
21. Post-treatment Follow-up: if a subject discontinues all study treatments (zolbetuximab/placebo and both components of CAPOX) prior to IRC-confirmed radiological disease progression, the subject will enter the Post-treatment Follow-up Period and continue to undergo imaging assessments every 9 weeks (±7 days) (or every 12 weeks [±7 days] if subject~~s~~ has been on study over 54 weeks) until radiologic disease progression (i.e., PFS) or the subject starts subsequent anticancer treatment, whichever occurs earlier. If study treatments (zolbetuximab/placebo and both components of CAPOX) are discontinued due to PD, the subject will enter the Long-term and Survival Follow-up Period.
22. Long-term and Survival Follow-up Period: Following disease progression on first-line treatment or start of subsequent anticancer treatment, subjects will be followed in the Long-term and Survival Follow-up Period per institutional guidelines, but not less than every 12 weeks. Radiologic imaging will be done per standard of care and read locally until PFS2 is documented. Survival Follow-up Period will continue until death (from any cause). All post-progression details including subsequent anticancer treatment and date and site of progression will be recorded on the eCRF. Subject contact by phone or other remote methods is sufficient during Long-term and Survival Follow-up.
23. Pharmacokinetics: Serum samples for zolbetuximab/placebo will be taken at the below time points and sent to the central laboratory. The date and time of each blood sample collection will be recorded to the nearest minute.
    - Cycle 1 Day 1: End of zolbetuximab/placebo infusion
    - Cycle 2 Day 1: Predose
    - Cycle 3 Day 1: End of zolbetuximab/placebo infusion
    - Predose on Day 1 of Cycles 5, 9, 13 and 17
    - Zolbetuximab/placebo 30-Day Safety Follow-up visit
    - Zolbetuximab/placebo 90-Day Safety Follow-up visit
    - Unscheduled pharmacokinetic blood samples may be taken at any time during the study to evaluate drug exposure following a safety event

    Pharmacokinetic Sampling Window:
    - Predose: within 60 minutes prior to dosing
    - End of Infusion: within 10 minutes after the end of the infusion
24. ADA: Blood samples (Serum) for ADA will be taken at the below time points and sent to the central laboratory.
    - Cycle 1 Day 1: Predose
    - Cycle 2 Day 1: Predose
    - Predose on Day 1 of Cycles 5, 9, 13 and 17
    - Zolbetuximab/placebo 30-Day Safety Follow-up visit

- Zolbetuximab/placebo 90-Day Safety Follow-up visit

ADA Sampling Window: Predose: within 60 minutes prior to dosing

25. Concomitant medications will be collected from the time of **full main** informed consent through 90 days following the last dose of study treatment.
26. AEs/SAEs: AEs and SAEs (regardless of causality) will be collected from the time of **full main** informed consent through 90 days following the last dose of study treatment. See [Section 5.5.5 Reporting of Serious Adverse Events].

**1 Introduction**
WAS:
Approximately 70% to 80% of patients with metastatic or advanced unresectable gastric and GEJ adenocarcinoma in the first line setting have tumors that are HER2 negative and are not treatable with trastuzumab. These patients have an expected median survival of approximately 1 year [Shah, 2017]. Therefore, a significant unmet medical need exists for the first-line treatment of patients with HER2 negative locally advanced or metastatic unresectable gastric and GEJ cancers. Zolbetuximab is being developed with the goal of addressing this unmet medical need.
IS AMENDED TO:
Approximately 70% to 80% of patients with metastatic or advanced unresectable gastric and GEJ adenocarcinoma in the first line setting have tumors that are HER2 negative and are not treatable with trastuzumab. These patients have an expected median survival of approximately 1 year [Shah **et al**, 2017]. Therefore, a significant unmet medical need exists for the first-line treatment of patients with HER2 negative locally advanced or metastatic unresectable gastric and GEJ cancers. Zolbetuximab is being developed with the goal of addressing this unmet medical need.

**1 Introduction**<br>*1.2.2 Clinical Data*
WAS:
To date, 3 clinical studies have been completed and include GM-IMAB-001 (EudraCT No. 2008 004719-37, referred to as first-in-human [FIM]), PILOT, and GM IMAB 001 02 (EudraCT No. 2009-017365-36) referred to as MONO. Dosing is complete and final data analyses and reporting are ongoing for the FAST study.
IS AMENDED TO:
To date, ~~3~~ **4** clinical studies have been completed and include GM-IMAB-001 (EudraCT No. 2008 004719-37, referred to as first-in-human [FIM]), PILOT, **FAST** and GM IMAB 001 02 (EudraCT No. 2009-017365-36) referred to as MONO. ~~Dosing is complete and final data analyses and reporting are ongoing for the FAST study.~~

**4 Identification of Study Treatment(s)**<br>*4.2 Comparative Drug (Placebo)*
WAS:
Placebo will not be manufactured by the sponsor. Sites should use their own commercial supply of 0.9% Sodium Chloride Injection as placebo. Details of preparation of placebo are provided in the Pharmacy Manual and Infusion Guidelines.

IS AMENDED TO:
**0.9% Sodium Chloride Injection will be used for infusion solution preparation in this study for both zolbetuximab arm and placebo arm.**<br><br>Placebo will not be manufactured by the sponsor. ~~Sites should use their own commercial supply of 0.9% Sodium Chloride Injection as placebo.~~ Details of preparation of placebo are provided in the Pharmacy Manual and Infusion Guidelines.

**5 Treatments and Evaluation**<br>*5.1.1.1 Zolbetuximab/Placebo*
WAS:
Subjects will be administered zolbetuximab/placebo as a minimum 2-hour intravenous infusion on day 1 of each cycle. Guidance for slowing the initial infusion to minimize toxicity can be found in the Pharmacy Manual and Infusion Guidelines. Subjects will be administered with a loading dose of 800 mg/m² of zolbetuximab at C1D1 followed by subsequent doses of 600 mg/m² every 3 weeks. Zolbetuximab/placebo must be administered prior to CAPOX. Flow rate data will be collected in the eCRF.<br><br>Please also refer to the dosing schematics for details [Section V].<br><br>It is recommended that zolbetuximab/placebo infusion not exceed 6 hours from start of infusion. Please refer to Pharmacy Manual and Infusion Guidelines for more detailed information, if zolbetuximab/placebo infusion cannot be completed.
IS AMENDED TO:
Subjects will be administered zolbetuximab/placebo as a minimum 2-hour intravenous infusion on day 1 of each cycle. Guidance for slowing the initial infusion to minimize toxicity can be found in the Pharmacy Manual and Infusion Guidelines. Subjects will be administered with a loading dose of 800 mg/m² of zolbetuximab at C1D1 followed by subsequent doses of 600 mg/m² every 3 weeks. Zolbetuximab/placebo ~~must~~ **should** be administered prior to CAPOX. Flow rate data will be collected in the eCRF.<br><br>Please also refer to the dosing schematics for details [Section V].<br><br>~~It is recommended that zolbetuximab/placebo infusion not exceed 6 hours from start of infusion.~~ **Intravenous infusion may be interrupted or slowed down to manage toxicity.** Please refer to Pharmacy Manual and Infusion Guidelines for more detailed information~~, if zolbetuximab/placebo infusion cannot be completed~~.

**5 Treatments and Evaluation**<br>*5.1.1.2 Antiemetics*
WAS:
• All antiemetic premedication should be given at minimum 30 minutes prior to zolbetuximab/placebo treatment.

IS AMENDED TO:

- All antiemetic premedication should be ~~given~~ **initiated** at **a** minimum **of** 30 minutes prior to zolbetuximab/placebo treatment.

5 Treatments and Evaluation
5.1.1.3 CAPOX

WAS:

CAPOX is to be administered after zolbetuximab/placebo infusion.

- Oxaliplatin: 130 mg/m2 intravenous infusion on day 1 of each cycle over 2 hours (or longer per institutional standard of care) for up to 8 treatments.

IS AMENDED TO:

CAPOX ~~is to~~ **should** be administered after zolbetuximab/placebo infusion.

- Oxaliplatin: 130 mg/m2 intravenous infusion on day 1 of each cycle over 2 hours (or longer per institutional standard of care) for ~~up to~~ **a maximum of** 8 treatments.

5 Treatments and Evaluation
5.1.2.2 Zolbetuximab/Placebo Interruption or Permanent Discontinuation

WAS:

A delay of zolbetuximab/placebo treatment of > 28 days from when the next zolbetuximab/placebo treatment was scheduled to be administered due to unresolved toxicity associated with zolbetuximab/placebo will result in the subject discontinuing zolbetuximab/placebo.

Radiologic imaging is to be scheduled every 9 weeks (±7 days) counting from C1D1 for the first 54 weeks and then every 12 weeks (±7 days) thereafter; the schedule should be maintained regardless of treatment delay.

Note: Intravenous infusion of zolbetuximab/placebo should be administered as a minimum 2 hour infusion. It is recommended that zolbetuximab/placebo infusion not exceed 6 hours from start of infusion. Please refer to Pharmacy Manual and Infusion Guidelines for more detailed information, if zolbetuximab/placebo infusion cannot be completed.

Guidelines for zolbetuximab/placebo treatment modification due to non-hematologic and hematologic toxicities are described below in Table 2 and Table 3, respectively.

AND

Table 2 Guidelines for Zolbetuximab/Placebo Treatment Modification Due to Non-hematologic Toxicity

Toxicity	Grade 1	Grade 2	Grade 3	Grade 4
**Infusion-related reaction (IRR)** ***other than nausea, vomiting or abdominal pain.*** See Table 4 for further IRR management guidance	Continue Infusion	Interrupt infusion. Infusion may be resumed at a reduced rate when toxicity has improved to grade ≤ 1.	Stop the infusion immediately. Institute appropriate medical management immediately based on the type of reaction. Permanently Discontinue zolbetuximab/placebo	
**Nausea**	Continue Infusion		Interrupt infusion. Hold zolbetuximab/placebo treatment until toxicity improved to grade ≤ 1. If the investigator determines that the toxicity is not related to zolbetuximab and the toxicity improves to grade ≤ 2, then infusion may be restarted at the investigator's discretion.	Not applicable*
**Vomiting**	Continue Infusion	Continue infusion; however, if infusion was held due to grade 3 vomiting, hold infusion until vomiting improves to ≤ grade 1.	Interrupt infusion. Hold zolbetuximab/placebo treatment until toxicity improved to grade ≤ 1.	Permanently Discontinue zolbetuximab/ placebo
**Other Non-hematologic toxicity**	Continue Infusion		Interrupt infusion. Hold zolbetuximab/placebo treatment until toxicity improved to grade ≤ 1. If the investigator determines that the toxicity is not related to zolbetuximab and the toxicity improves to grade ≤ 2, then infusion may be restarted at the investigator's discretion.	Permanently Discontinue zolbetuximab/ placebo
**PRES**	Discontinue zolbetuximab/placebo if PRES is suspected.			

* Grade 4 nausea is not defined in CTCAE v4.03. If investigator assesses nausea as grade 4, manage per local standard of care

IS AMENDED TO:

A delay of zolbetuximab/placebo treatment of > 28 days from when the next zolbetuximab/placebo treatment was scheduled **to begin (> 49 days from when the last dose of zolbetuximab/placebo began)** to be administered due to unresolved toxicity associated with zolbetuximab/placebo will result in the subject discontinuing zolbetuximab/placebo.

Radiologic imaging is to be scheduled every 9 weeks (±7 days) counting from C1D1 for the first 54 weeks and then every 12 weeks (±7 days) thereafter; the schedule should be maintained regardless of treatment delay.

Note: Intravenous infusion of zolbetuximab/placebo should be administered as a minimum 2 hour infusion. ~~It is recommended that zolbetuximab/placebo infusion not exceed 6 hours from start of infusion.~~ **Intravenous infusion may be interrupted or slowed down to manage toxicity.** Please refer to Pharmacy Manual and Infusion Guidelines for more detailed information~~, if zolbetuximab/placebo infusion cannot be completed~~.

Guidelines for zolbetuximab/placebo treatment modification due to non-hematologic and hematologic toxicities**, regardless of investigator assessment of relationship to zolbetuximab/placebo,** are described below in Table 2 and Table 3, respectively.

AND

Table 2 Guidelines for Zolbetuximab/Placebo Treatment Modification Due to Non-hematologic Toxicity

Toxicity	Grade 1	Grade 2	Grade 3	Grade 4
**Infusion-related reaction (IRR)** ***other than nausea, vomiting or abdominal pain.*** See Table 4 for further IRR management guidance	Continue Infusion	Interrupt infusion. Infusion may be resumed at a reduced rate when toxicity has improved to grade ≤ 1.	Stop the infusion immediately. Institute appropriate medical management immediately based on the type of reaction. Permanently Discontinue zolbetuximab/placebo	
**Nausea**	Continue Infusion		Interrupt infusion. Hold zolbetuximab/placebo treatment until toxicity **has** improved to grade ≤ 1**, then restart the infusion at a lower rate.** If the investigator determines that the toxicity is not related to zolbetuximab and the toxicity **has** improve~~s~~d to grade ≤ 2, then infusion may be restarted at the	Not applicable*

			investigator's discretion **at a lower rate.**	
**Vomiting**	Continue Infusion	Continue infusion; however, if infusion was held due to grade 3 vomiting, hold infusion until vomiting **has** improvesd to ≤ grade 1.	Interrupt infusion. Hold zolbetuximab/placebo treatment until toxicity **has** improved to grade ≤ 1 **and then restart infusion at a lower rate.**	Permanently Discontinue zolbetuximab/ placebo
**Other Non-hematologic toxicity**	Continue Infusion		Interrupt infusion. Hold zolbetuximab/placebo treatment until toxicity **has** improved to grade ≤ 1.<br>If the investigator determines that the toxicity is not related to zolbetuximab and the toxicity **has** improvesd to grade ≤ 2, then infusion may be restarted at the investigator's discretion.#	Permanently Discontinue zolbetuximab/ placebo
**PRES**	Discontinue zolbetuximab/placebo if PRES is suspected.			

* Grade 4 nausea is not defined in CTCAE v4.03. If investigator assesses nausea as grade 4, manage per local standard of care.
# For subjects with a pulmonary embolism, treatment can continue without resolving to grade 2 or less.

5 Treatments and Evaluation

5.1.2.3 Guidelines for Infusion-related Reactions for Zolbetuximab/Placebo

WAS:

Note: Intravenous infusion of zolbetuximab/placebo should be administered as a minimum 2 hour infusion. It is recommended that zolbetuximab/placebo infusion not exceed 6 hours from start of infusion. Please refer to Pharmacy Manual and Infusion Guidelines for more detailed information, if zolbetuximab/placebo infusion cannot be completed.

Table 4 Infusion-related Reactions

**Infusion-Related Reactions**	
Refer to Table 2 for management of infusion related reactions of nausea, vomiting or abdominal pain	
**CTCAE v4.03 Grade**	**Management**
Grade 1 standard infusion reactions ***other than nausea, vomiting or abdominal pain#***	Continue infusion and closely monitor the subject.
Grade 2 standard infusion reaction ***other than nausea, vomiting or abdominal pain#***	Interrupt.

	Medical management as per type of reaction. Resume infusion once toxicity grade ≤ 1 and reduce the infusion rate for the remaining infusion.<br>For the next infusion:<br>• Increase total infusion time (reduce infusion rate).<br>• Pre-medicate as appropriate.*<br>• Closely monitor the subject for symptoms and signs of an infusion reaction.
Any infusion reaction with features of anaphylaxis<br>**OR**<br>Grade 3 or 4 standard infusion reactions ***other than nausea, vomiting or abdominal pain***#	Stop the infusion immediately.<br>Institute appropriate medical management immediately based on the type of reaction.<br>Permanently Discontinue zolbetuximab/placebo<br>*Once the subject has been stabilized, collect blood for cytokine/chemokine panel and serum total tryptase level (levels typically peak within 3 hours after the onset of symptoms*

CTCAE v4.03: Common Terminology Criteria For Adverse Events
* At the investigators discretion, antihistamines may be used as premedication for the next infusion. Systemic corticosteroids should be avoided or minimized while subject is on study treatment unless required for management of an emergent medical condition (e.g., severe nausea/vomiting or hypersensitivity reaction).
# For grade 3 or 4 IRR of nausea, vomiting or abdominal pain, collect blood for cytokine/chemokine panel and serum total tryptase level.

IS AMENDED TO:

Note: Intravenous infusion of zolbetuximab/placebo should be administered as a minimum 2 hour infusion. ~~It is recommended that zolbetuximab/placebo infusion not exceed 6 hours from start of infusion.~~ **Intravenous infusion may be interrupted or slowed down to manage toxicity.** Please refer to Pharmacy Manual and Infusion Guidelines for more detailed information~~, if zolbetuximab/placebo infusion cannot be completed~~.

Table 4 Infusion-related Reactions

**Infusion-Related Reactions**	
Refer to Table 2 for management of infusion related reactions of nausea, vomiting or abdominal pain	
**CTCAE v4.03 Grade**	**Management**
Grade 1 standard infusion reactions ***other than nausea, vomiting or abdominal pain***#	Continue infusion and closely monitor the subject.
Grade 2 standard infusion reaction ***other than nausea, vomiting or abdominal pain***#	Interrupt.<br>Medical management as per type of reaction. Resume infusion once toxicity grade ≤ 1 and reduce the infusion rate for the remaining infusion.<br>For the next infusion:<br>• Increase total infusion time (reduce infusion rate).<br>• Pre-medicate as appropriate.*<br>• Closely monitor the subject for symptoms and signs of an infusion reaction.

Any infusion reaction with features of anaphylaxis<br>**OR**<br>Grade 3 or 4 standard infusion reactions ***other than nausea, vomiting or abdominal pain***[#]	Stop the infusion immediately.<br>Institute appropriate medical management immediately based on the type of reaction.<br>Permanently Discontinue zolbetuximab/placebo<br>*Once the subject has been stabilized, collect blood for cytokine/chemokine panel and serum total tryptase level (levels typically peak within 3 hours after the onset of symptoms)* ***and send to the central laboratory.***

CTCAE v4.03: Common Terminology Criteria For Adverse Events
* At the investigators discretion, antihistamines may be used as premedication for the next infusion. Systemic corticosteroids should be avoided or minimized while subject is on study treatment unless required for management of an emergent medical condition (e.g., severe nausea/vomiting or hypersensitivity reaction).
# For grade 3 or 4 IRR of nausea, vomiting or abdominal pain, collect blood for cytokine/chemokine panel and serum total tryptase level **and send to the central laboratory**.

5 Treatments and Evaluation
5.1.2.4 CAPOX Dose Modification

WAS:

The first dose of CAPOX should not be modified. After the assessment of tolerability, dose adjustments should be performed based on investigator judgement utilizing institutional standard of care, approved package insert, SPC, or local product information and/or the recommended criteria in Table 5 based on maximum hematologic or non-hematologic toxicity data from the previous cycle as shown in Table 6 and Table 7, respectively. Dose reduction criteria for oxaliplatin-related neurotoxicity are presented in Table 8. Each drug may be dose reduced independently based on the specific types of toxicities observed. No more than 2 dose reductions will be allowed per drug per subject (see Table 5). Dose re-escalation is not permitted. If further dose reduction is required beyond the criteria in Table 5, that component of CAPOX should be discontinued.

In subjects experiencing toxicity requiring a delay or discontinuation of CAPOX, subject should continue to receive zolbetuximab/placebo as clinically appropriate. If CAPOX is interrupted, subject should be evaluated weekly (at a minimum) until the toxicity has improved sufficiently at which time treatment can be restarted as described in the tables below (as applicable). A delay of CAPOX treatment for > 28 days from when the next CAPOX treatment was scheduled to be administered due to unresolved toxicity associated with CAPOX will result in the subject discontinuing CAPOX (both components).

IS AMENDED TO:

The first dose of CAPOX should not be modified. After the assessment of tolerability, dose adjustments should be performed based on investigator judgement utilizing institutional standard of care, approved package insert, SPC, or local product information and/or the recommended criteria in Table 5 based on maximum hematologic or non-hematologic toxicity data from the previous cycle as shown in Table 6 and Table 7, respectively. Dose reduction criteria for oxaliplatin-related neurotoxicity are presented in Table 8. Each drug may be dose reduced independently based on the specific types of toxicities observed. **It is recommended that** ~~N~~**n**o more than 2 dose reductions ~~will be~~

~~allowed~~ per drug per subject **occur** (see Table 5). Dose re-escalation is not ~~permitted~~ **recommended after treatment-related AEs.** If further dose reduction is required beyond the criteria in Table 5, that component of CAPOX should be discontinued.<br><br>In subjects experiencing toxicity requiring a delay or discontinuation of CAPOX, subject should continue to receive zolbetuximab/placebo as clinically appropriate. If CAPOX is interrupted, subject should be evaluated weekly (at a minimum) until the toxicity has improved sufficiently at which time treatment can be restarted as described in the tables below (as applicable). A delay of CAPOX treatment for > 28 days from when the next CAPOX treatment was scheduled to **begin (> 49 days from when the last CAPOX dose began)** ~~be administered~~ due to unresolved toxicity associated with CAPOX will result in the subject discontinuing CAPOX (both components).

**5 Treatments and Evaluation**<br>*5.1.2.5 CAPOX: Dose Modifications for Hematologic Toxicity*
WAS:
The CAPOX dose modifications for hematologic toxicity are presented in Table 6. Dose modifications should be maintained until recovery from hematologic toxicity. A delay of CAPOX treatment for > 28 days from when the next CAPOX treatment was scheduled to be administered due to hematologic toxicity associated with CAPOX will result in the subject discontinuing CAPOX (both components).
IS AMENDED TO:
The CAPOX dose modifications for hematologic toxicity are presented in Table 6. Dose modifications should be maintained until recovery from hematologic toxicity. A delay of CAPOX treatment for > 28 days from when the next CAPOX treatment was scheduled to **begin (> 49 days from when the last CAPOX dose began)** ~~be administered~~ due to hematologic toxicity associated with CAPOX will result in the subject discontinuing CAPOX (both components).

**5 Treatments and Evaluation**<br>*5.1.2.6 CAPOX: Dose Modification for Non-hematologic Toxicity*
WAS:
CAPOX dose modifications for non-hematologic toxicity should be based on the most severe toxicity experienced during the last treatment (Table 7). Retreatment should be delayed until recovery of all non-hematologic toxicity to ≤ grade 2 with the exception of increased bilirubin or ALT, which must recover to grade 1 or baseline, whichever was higher. The maximum permitted treatment delay is 28 days from when the next study treatment was scheduled to be administered for recovery of non-hematologic toxicity. If the subject has not recovered sufficiently to meet retreatment criteria within that timeframe, CAPOX should be discontinued.
IS AMENDED TO:

CAPOX dose modifications for non-hematologic toxicity should be based on the most severe toxicity experienced during the last treatment (Table 7). Retreatment should be delayed until recovery of all non-hematologic toxicity to ≤ grade 2 with the exception of increased bilirubin or ALT, which must recover to grade 1 or baseline, whichever was higher. The maximum permitted treatment delay is 28 days from ~~when~~ the next study treatment was scheduled to **begin (49 days from when the last dose of study treatment began)** ~~be administered~~ for recovery of non-hematologic toxicity. If the subject has not recovered sufficiently to meet retreatment criteria within that timeframe, CAPOX should be discontinued.

5 Treatments and Evaluation
5.1.2.8 Oxaliplatin-induced Neurotoxicity

WAS:

Table 8 Oxaliplatin Dose Modification for Associated Neurotoxicity

Toxicity/Duration	Grade 1	Grade 2	Grade 3	Grade 4
**Paresthesia or dysesthesia**	Paresthesia or dysesthesia‡ that does not interfere with function	Paresthesia or dysesthesia‡, interfering with function, but not activities of daily living	Paresthesia or dysesthesia‡ with pain or with functional impairment that also interferes with activities of daily living	Persistent paresthesia or dysesthesia that is disabling or life-threatening
**1 to 7 Days**	No dose reduction	No dose reduction	First Event: Reduce oxaliplatin by 1 dose level at next treatment. Second Event: Reduce oxaliplatin by a second dose level at next treatment.	Discontinue oxaliplatin
**> 7 Days**			Discontinue oxaliplatin	
**Persistent between treatments†**		Reduce oxaliplatin by 1 dose level at next treatment	Discontinue oxaliplatin	
**Acute laryngopharyngeal dysesthesia‡** (during or after the 2-hour infusion)	Discontinue current infusion. At next treatment, increase duration of infusion to 6 hours; may also pretreat with benzodiazepines.			

IS AMENDED TO:

Table 8 Oxaliplatin Dose Modification for Associated Neurotoxicity

Toxicity/Duration	Grade 1	Grade 2	Grade 3	Grade 4
**Paresthesia or dysesthesia**	Paresthesia or dysesthesia‡	Paresthesia or dysesthesia‡,	Paresthesia or dysesthesia‡	Persistent paresthesia or

<table>
<tr><td></td><td>that does not interfere with function</td><td>interfering with function, but not activities of daily living</td><td>with pain or with functional impairment that also interferes with activities of daily living</td><td>dysesthesia that is disabling or life-threatening</td></tr>
<tr><td>1 to 7 Days</td><td rowspan="3">No dose reduction</td><td rowspan="2">No dose reduction</td><td>First Event: Reduce oxaliplatin by 1 dose level at next treatment. Second Event: Reduce oxaliplatin by a second dose level at next treatment.</td><td rowspan="3">Discontinue oxaliplatin</td></tr>
<tr><td>> 7 Days</td><td>Discontinue oxaliplatin</td></tr>
<tr><td>Persistent between treatments†</td><td>Reduce oxaliplatin by 1 dose level at next treatment</td><td>Discontinue oxaliplatin</td></tr>
<tr><td>Acute laryngopharyngeal dysesthesia‡
(during or after the 2-hour infusion)</td><td colspan="4">Discontinue current infusion.
At next treatment, consider pretreatment with benzodiazepines and <s>increase</s> increasing duration of infusion <s>to 6 hours; may also pretreat with benzodiazepines</s> as clinically indicated per investigator discretion.</td></tr>
</table>

5 Treatments and Evaluation

5.1.5 Previous and Concomitant Treatment (Medication and Non-Medication Therapy)

WAS:

All medications and concomitant treatments administered from the time of informed consent through the 90-day safety follow-up visit must be recorded in the eCRF. Documentation will include the medication name, indication, route and dates of administration.

AND

- Live vaccines should be avoided during the treatment period in which subject is receiving capecitabine and up to 6 months after final capecitabine dose.

AND

The following should be avoided or used with caution and closely monitored during CAPOX administration:

• Cytochrome P450 2C9 substrates (Subjects taking coumarin-derivative anticoagulants concomitantly with capecitabine should have PT/INR monitored regularly and anticoagulant dose adjusted accordingly).<br>• Anti-epileptic medications (e.g., phenobarbital, phenytoin and primidone)<br>• Medications known to prolong the QT or QTc interval (refer to https://www.crediblemeds.org for a list of these medications
IS AMENDED TO:
All medications and concomitant treatments administered from the time of **full main** informed consent through the 90-day safety follow-up visit must be recorded in the eCRF. Documentation will include the medication name, indication, route and dates of administration.<br><br>AND<br><br>• Live vaccines should be avoided during the treatment period in which subject is receiving capecitabine and up to 6 months after final capecitabine dose. **In cases where a live vaccine is needed for COVID-19 prevention, please contact the Medical Monitor for discussion.**<br><br>AND<br><br>The following should be avoided or used with caution and closely monitored during ~~CAPOX~~ **capecitabine** administration:<br>• Cytochrome P450 2C9 substrates (Subjects taking coumarin-derivative anticoagulants concomitantly with capecitabine should have PT/INR monitored regularly and anticoagulant dose adjusted accordingly).<br>• Anti-epileptic medications (e.g., phenobarbital, phenytoin and primidone)<br>**The following should be avoided or used with caution and closely monitored during oxaliplatin administration:**<br>• Medications known to prolong the QT or QTc interval (refer to https://www.crediblemeds.org for a list of these medications

**5 Treatments and Evaluation**<br>*5.2.2 Medical History*
WAS:
Medical history includes all significant medical conditions per the judgement of the investigator that have resolved prior to informed consent or are ongoing at the time of consent.

IS AMENDED TO:
Medical history includes all significant medical conditions per the judgement of the investigator that have resolved prior to informed consent or are ongoing at the time of **full main** consent.

**5 Treatments and Evaluation**<br>*5.4.3 Laboratory Assessments*
WAS:
• From cycle 2 onwards central and local labs may be collected up to 48 hours prior to study treatment.
IS AMENDED TO:
• ~~From cycle 2 onwards l~~**L**abs may be collected up to 48 hours prior to study treatment.

**5 Treatments and Evaluation**<br>*5.4.6 Performance Status*
WAS:
The ECOG Scale [Oken, 1982] will be used to assess performance status. Refer to [Appendix 12.7].
IS AMENDED TO:
The ECOG Scale [Oken **et al**, 1982] will be used to assess performance status. Refer to [Appendix 12.7].

**5 Treatments and Evaluation**<br>*5.5 Adverse Events and Other Safety Aspects*
WAS:
AE collection will begin from time of informed consent and continue through the 90 days following the last dose of zolbetuximab/placebo and CAPOX (both components).<br><br>Serious adverse events (SAEs), regardless of causality will be collected from the time of informed consent through 90 days following the last dose of zolbetuximab/placebo and CAPOX (both components).
IS AMENDED TO:
AE collection will begin from time of **full main** informed consent and continue through the 90 days following the last dose of zolbetuximab/placebo and CAPOX (both components).

Serious adverse events (SAEs), regardless of causality will be collected from the time of **full main** informed consent through 90 days following the last dose of zolbetuximab/placebo and CAPOX (both components).

**5 Treatments and Evaluation**<br>*5.5.1 Definition of Adverse Events*
WAS:
In order to identify any events that may be associated with study procedures and could lead to a change in the conduct of the study, Astellas collects AEs even if the subject has not received study drug treatment. AE collection begins after the signing of the informed consent and will be collected until 90 days after the last dose of study drug or the subject is determined to be a screen failure.
IS AMENDED TO:
In order to identify any events that may be associated with study procedures and could lead to a change in the conduct of the study, Astellas collects AEs even if the subject has not received study drug treatment. AE collection begins after the signing of the **full main** informed consent and will be collected until 90 days after the last dose of study drug or the subject is determined to be a screen failure.

**5 Treatments and Evaluation**<br>*5.5.5 Reporting of Serious Adverse Events*
WAS:
The collection of AEs and the expedited reporting of SAEs will start following receipt of the signed informed consent and will continue until 90 days after the last dose of study treatment or the subject is determined to be a screen failure.
IS AMENDED TO:
The collection of AEs and the expedited reporting of SAEs will start following receipt of the signed **full main** informed consent and will continue until 90 days after the last dose of study treatment or the subject is determined to be a screen failure.

**5 Treatments and Evaluation**<br>*5.5.8 Adverse Events of Special Interest*
WAS:
In case of zolbetuximab induced nausea, vomiting or hypersensitivity/IRR, infusion rate of zolbetuximab/placebo may be reduced or infusion paused or discontinued based on investigator’s clinical judgement about severity of toxicity and local standard of care. See [Section 5.1.2 Study Treatment Dose Modifications, Delays and Interruption].

IS AMENDED TO:
In case of zolbetuximab induced nausea, vomiting or hypersensitivity/IRR, infusion rate of zolbetuximab/placebo may be reduced or infusion paused or discontinued based on investigator's clinical judgement about severity of toxicity and local standard of care. See [Section 5.1.2 Study Treatment Dose Modifications, Delays and Interruptions] **and the Pharmacy Manual and Infusion Guidelines.**

**5 Treatments and Evaluation**<br>*5.7.1 Tumor Tissue Samples*
WAS:
FFPE tumor tissue samples will be obtained for all subjects and sent for central IHC testing to evaluate for CLDN18.2 status. Tissue from the primary site is preferred; however, if a metastatic site is used (excluding bone metastasis), the sample should be gastric or GEJ in origin. Archival tumor tissue is preferred, but if the specimen is insufficient or unavailable, a biopsy may be performed to obtain a primary tumor tissue or tumor tissue from metastatic site (excluding bone metastasis). Sponsor preapproval is required when a biopsy procedure is needed for the sole purpose of determining study eligibility.
IS AMENDED TO:
FFPE tumor tissue samples will be obtained for all subjects and sent for central IHC testing to evaluate for CLDN18.2 status. Tissue from the primary site is preferred; however, if a metastatic site is used (excluding bone metastasis), the sample should be gastric or GEJ in origin. Archival tumor tissue is preferred, but if the specimen is insufficient or unavailable, a biopsy may be performed to obtain a primary tumor tissue or tumor tissue from metastatic site (excluding bone metastasis). Sponsor preapproval is ~~required~~ **requested** when a biopsy procedure is needed for the sole purpose of determining study eligibility.

**5 Treatments and Evaluation**<br>*5.7.3 Electronic Clinical Outcome Assessments*
WAS:
Subjects will be asked to complete HRQoL and HRU questionnaires as specified in the Schedule of Assessments [Table 1]. The electronic Clinical Outcomes Assessment (eCOA) questionnaires should be administered during the visit (or up to 48 hours) before any antiemetic or drug treatment and before the disease status is discussed with the subject. For subjects with low literacy or situations where required translation is not available, please contact the sponsor for further guidance. Assessments will be collected at Screening (except for HRU), every 3 weeks, at study treatment discontinuation and 30 and 90 days post zolbetuximab/placebo treatment. HRQoL will be measured by EORTC QLQ-C30, QLQ OG25 plus STO22 Belching subscale, GP and the EQ5D-5L.
IS AMENDED TO:
Subjects will be asked to complete HRQoL and HRU questionnaires as specified in the Schedule of Assessments [Table 1]. The electronic Clinical Outcomes Assessment (eCOA) questionnaires should be ~~administered during the visit~~ **completed by the subject**

**at Screening (except for HRU), day 1 of each cycle** (or up to 48 hours) before any antiemetic or drug treatment and before the disease status is discussed with the subject **using the electronic device provided. When completion by the subject is not possible, the questionnaires may be administered to the subject by site personnel using the electronic tablet device**. For subjects with low literacy or situations where required translation is not available, please contact the sponsor for further guidance. Assessments will **also** be collected at ~~Screening (except for HRU), every 3 weeks, at~~ study treatment discontinuation and 30 and 90 days post zolbetuximab/placebo treatment. **HRQoL and HRU questionnaires are not required at CAPOX dosing visits (if different from zolbetuximab/placebo dosing visit), CAPOX treatment discontinuation, 30 day safety follow-up and 90 day safety follow-up visits. A combined visit can be completed if zolbetuximab/placebo and both components of CAPOX are discontinued on the same day and HRQoL and HRU questionnaires will be required at those combined visits.** HRQoL will be measured by EORTC QLQ-C30, QLQ OG25 plus STO22 Belching subscale, GP and the EQ5D-5L.

**6 Discontinuation**<br>*6.1 Discontinuation of Individual Subject(s) From Study Treatment*
WAS:
• Subject has a delay of zolbetuximab/placebo and both components CAPOX treatment for > 28 days from when the next zolbetuximab/placebo and both components CAPOX treatment was scheduled to be administered.
IS AMENDED TO:
• Subject has a delay of zolbetuximab/placebo and both components **of** CAPOX treatment for > 28 days from when the next zolbetuximab/placebo and both components **of** CAPOX treatment was scheduled **to begin (> 49 days from when the last dose of zolbetuximab/placebo and both components of CAPOX treatment began)** ~~to be administered~~.

**8 Operational Considerations**<br>*8.1.1.2 Electronic Clinical Outcomes Assessment*
WAS:
eCOA assessments will be performed according to the Schedule of Assessments [Table 1]. Subject HRQoL and HRU questionnaires will be completed by the subject on an electronic tablet device during site visits.
IS AMENDED TO:
eCOA assessments will be performed according to the Schedule of Assessments [Table 1]. Subject HRQoL and HRU questionnaires will be completed by the subject **or, if not possible, administered to the subject by site personnel** on an electronic tablet device ~~during site~~ **for subject** visits.

11 References

WAS:

Ohtsu A, Shah MA, Van Cutsem E, Rha SY, Sawaki A, Park SR, et al. Bevacizumab in combination with chemotherapy as first-line therapy in advanced gastric cancer: a randomized, double-blind, placebo-controlled phase III study. J Clin Oncol. 2011;29:3968-3976.

Pasini F, Fraccon AP, De Manzoni G. The role of chemotherapy in metastatic gastric cancer. Cancer Res. 2011;31:3543-3554.

IS AMENDED TO:

Ohtsu A, Shah MA, Van Cutsem E, Rha SY, Sawaki A, Park SR, et al. Bevacizumab in combination with chemotherapy as first-line therapy in advanced gastric cancer: a randomized, double-blind, placebo-controlled phase III study. J Clin Oncol. 2011;29:3968-3976.

Oken MM, Creech RH, Tormey DC, Horton J, Davis TE, McFadden ET, et al. Toxicity and response criteria of the Eastern Cooperative Oncology Group. Am J Clin Oncol. 1982;5(6):649-55.

Pasini F, Fraccon AP, De Manzoni G. The role of chemotherapy in metastatic gastric cancer. Cancer Res. 2011;31:3543-3554.

Appendix 12.1 Ethical, Regulatory, and Study Oversight Considerations
12.1.4.1 Subject Information and Consent

WAS:

An optional partial screening ICF is available to allow central testing of tissue for CLDN18.2 and HER2 only.

IS AMENDED TO:

An optional partial screening ICF ~~is~~ **may be** available to allow central testing of tissue for CLDN18.2 and HER2 only.

Appendix 12.4 Concomitant Medication Restrictions or Requirements
Prohibited Concomitant Treatment

WAS:

- Live vaccines should be avoided during the treatment period in which subject is receiving capecitabine and up to 6 months after final capecitabine dose.

AND

The following should be avoided or used with caution and closely monitored during CAPOX administration:

- Cytochrome P450 2C9 substrates (Subjects taking coumarin-derivative anticoagulants concomitantly with capecitabine should have PT/INR monitored regularly and anticoagulant dose adjusted accordingly).
- Anti-epileptic medications (e.g., phenobarbital, phenytoin and primidone)

Medications known to prolong the QT or QTc interval (refer to https://www.crediblemeds.org for a list of these medications

IS AMENDED TO:

- Live vaccines should be avoided during the treatment period in which subject is receiving capecitabine and up to 6 months after final capecitabine dose. **In cases where a live vaccine is needed for COVID-19 prevention, please contact the Medical Monitor for discussion.**

AND

The following should be avoided or used with caution and closely monitored during ~~CAPOX~~ **capecitabine** administration:

- Cytochrome P450 2C9 substrates (Subjects taking coumarin-derivative anticoagulants concomitantly with capecitabine should have PT/INR monitored regularly and anticoagulant dose adjusted accordingly).
- Anti-epileptic medications (e.g., phenobarbital, phenytoin and primidone)

The following should be avoided or used with caution and closely monitored during oxaliplatin administration:

- Medications known to prolong the QT or QTc interval (refer to https://www.crediblemeds.org for a list of these medications

Appendix 12.8 Clinical Study Continuity

ADDED:

INTRODUCTION

The purpose of this appendix is to provide acceptable alternate methods to assess safety and efficacy parameters, as appropriate, in the event the clinical study is interrupted at the country, state, site or participant level during any crisis (e.g., natural disaster, pandemic).

BENEFIT-RISK RATIONALE

Maintaining the safety of clinical study participants and delivering continuity of care in the clinical study setting is paramount during any crisis. The site is expected to follow the protocol and associated Schedule of Assessments [Table 1] unless the site PI determines the need to implement the alternate measures. The PI should notify Astellas and/or their CRA when these alternate measures are needed.

The approach outlined within this appendix defines which assessments are required to maintain a favorable benefit/risk to the participant, to maintain overall study integrity and to provide acceptable alternate methods to complete the study required assessments and procedures if study activities are unable to be performed as described in Table 1 due to a crisis.

INFORMED CONSENT

Participants who need to follow any or all of the alternate measures outlined in this Appendix will be required to provide informed consent which explicitly informs them of the nature of, and rationale for these changes, and gain their agreement to continue participation in the study prior to the implementation of any of these changes. In the event the urgency of implementing the alternate measures does not allow for the participant to provide written consent prior to implementation, the PI or designee will obtain oral agreement from the subject followed by written documentation as soon as is feasible. A separate addendum to the study informed consent will be provided to document the participant's consent of the changes.

PARTICIPANT PROCEDURES ASSESSMENT

Sites with participants who are currently enrolled into this clinical study may consider implementing the alternate methods outlined below if one or more of the following conditions are met due to the crisis:

- **Regional or local travel has been restricted, inclusive of mandatory shelter in place measures, which makes participant travel to/from the study site nearly impossible**

- **Site facilities have been closed for clinical study conduct**
- **Site has been restricted to treating patients with conditions outside of the scope of the study**
- **Site personnel have temporarily relocated the conduct of the study to a location that place a burden on the participant with respect to time and travel**
- **Participant(s) have temporarily relocated from the current study site to an alternate study site to avoid placing a burden on the participant with respect to travel**
- **Participant(s) have temporarily relocated from their home location and the new distances from the site would cause undue burden with respect to time and travel**
- **Participant has risk factors for which traveling to the site poses an additional risk to the participant's health and safety**

Adherence to the original protocol as reflected in the Schedule of Assessments [Table 1] is expected, where plausible, in the case of a crisis. The alternate measures as noted in [Table 10] below are only permissible in the event of a crisis, and after discussing the need with the Astellas Medical Monitor to implement the alternate measures. This is to allow for continuity of receiving investigational medicinal product (IMP) and maintaining critical safety and efficacy assessments for patients participating in the study at a time of crisis.

If one or more of the alternate measures noted below is implemented for a participant, the site should document in the participant's source document the justification for implementing the alternate measure and the actual alternate measures that were implemented, along with the corresponding time point(s).

Table 10 Alternative Schedule of Assessments in Response to a Crisis

Critical Assessment	Alternate Approach(es)	Critical Timepoint										
		Cycles 1-8			Cycle 9+			Study Treatment Discontinuation Visit[18]	30-Day Safety Follow-up Visit(s)[19]	90-Day Safety Follow-up Visit(s)[20]	Post-treatment Follow-up Period [21]	Long-term and Survival Follow-up Periods [22]
**Cycle Day**		1	2-14	15-21	1	2-14	15-21					
**Treatments**												
**Antiemetic Pretreatment[5]**	Oral antiemetics can be administered at home as per SoC	X			X							
**Zolbetuximab/Placebo[6]**	Window of -2 days acceptable	X			X							
**Post-infusion Observation Period[7]**	Decrease of initial observation period to 1 hour and subsequent observation period to 30 min is acceptable if there are no AEs of ≥ grade 2	X			X							
Table continued on next page												
**Oxaliplatin** } **CAPOX[8]**	If previous cycle Day 1 zolbetuximab/placebo administration was conducted at the study site, the following cycle can be administered (oxaliplatin) and dispensed (capecitabine) locally per SoC only (not zolbetuximab/placebo administration) by oncology qualified personnel, and if dosing records can be obtained from the treating facility. The next cycle then must be administered at the study site	X										

**Capecitabine**	**for zolbetuximab/placebo administration. At least every other cycle must be conducted at the study site for zolbetuximab/placebo and oxaliplatin administration and capecitabine dispensing.**	**X**	**X**		**X**	**X**						
Table continued on next page												
**Safety Assessments**												
**Physical Examination[9]**	**Targeted exam is allowed after C1D1. Physical exam not completed at Study Treatment Discontinuation Visit and 30-Day Safety Follow-up Visit acceptable if no active AE.**	**X**			**X**							
**Weight[9]**	**If there are no associated active AEs, it is acceptable if weight is not done at Study Treatment Discontinuation Visit and 30-Day Safety Follow-up Visit.**	**X**			**X**							
**Vital Signs[10]**	**If a cycle is administered at a local facility for SoC regimen administration, SoC can be applied. Missed assessments at Study Treatment Discontinuation Visit and 30-Day Safety Follow-up visit acceptable if there are no associated active AEs. Vital sign frequency during post observation period can be decreased.**	**X**			**X**							
**ECOG Performance Status[9]**	**Not required at Study Treatment Discontinuation visit; ECOG Performance Status may be assessed and captured via phone contact.**	**X**			**X**							
Table continued on next page												

**12-lead ECG[11]**	**ECGs allowed up to 4 days prior to treatment visits after C1D1 and can be done locally but must be reviewed prior to dosing; if dosing visits are conducted at local facility, SoC ECG monitoring would be acceptable; if capecitabine only is dispensed and ECG is not able to be performed, this is acceptable; Study Treatment Discontinuation Visit and 30-Day Safety Follow-up Visit required only if clinically indicated.**	**X**			**If clinically indicated and/or per local requirements**							
Table continued on next page												
**Image Assessment[12]**	**At select visits, efficacy assessment using radiological examinations are required. Independent central reading of locally obtained scans can be facilitated by sharing of Image Acquisition Guidelines from the study site to local site, if applicable. Imaging assessment can be done locally but must be available for submission to central imaging vendor. Investigational site will be requested to re-read the scan performed at local site. If investigational site read is not an option, the investigator should discuss the case with the local institution radiologist. The local site imaging report is required.**	**Every 9 weeks ±7 days from C1D1 for the first 54 weeks and then every 12 weeks ±7 days thereafter**										
**Subject Contact**	**Long Term Survival and Safety follow-up visits can be conducted via phone.**											**X**
Table continued on next page												
**Laboratory Tests**												
**Biochemistry[13]**	**Sample may be collected up to 4 days prior to treatment visit; collection of samples at local facility acceptable if results can be made available to investigative site.**	**X**			**X**			**X**	**X**			
**TSH and Free T4[13]**	**If this testing is unable to be performed, this is acceptable**	**If clinically indicated**										

**Hematology[13]**	**Sample may be collected up to 4 days prior to treatment visit; collection of samples at local facility acceptable if results can be made available to investigative site.**	**X**			**X**			**X**	**X**			
**Coagulation Parameters (PT, PTT and INR)[14]**	**None as protocol allows SoC if clinically indicated. If the subject is not on a concomitant medication that affects these parameters, it is acceptable if these are not done.**	**If clinically indicated**										
**Urinalysis[13]**	**Sample may be collected up to 4 days prior to treatment visit; collection at local facility also allowed.**	**X**			**X**			**X**	**X**			
**Serum Pregnancy Test[15]**	**Collection at local facility also allowed.**	**If clinically indicated and/or per local requirements**										
Table continued on next page												
**Urine Pregnancy Test[16]**	**Sample may be collected up to 4 days prior to treatment visit; collection at local facility also allowed if results can be made available to investigative site.**	**X**			**X**			**X**	**X**			
**Sampling**												
**Pharmacokinetics of Zolbetuximab (Serum)[23]**	**Samples at predose/End of Infusion will be collected if subject receives treatment at investigative site. If central lab cannot receive samples, samples can be stored at sites in -70°C freezer until shipping is accepted again. Follow-up samples will be collected if subject visits the investigative site.**	**X**			**X**				**X**	**X**		
**Antidrug-Antibodies (ADA) for Immunogenicity[24]**	**If subject is dosed or visits investigative site, ADA samples should be collected. Samples cannot be collected at local facility. Sample collection prioritized if clinically indicated. If central lab cannot receive samples, samples can be stored at sites in -70°C freezer until shipping is accepted again.**	**X**			**X**				**X**	**X**		
**Concomitant Medication[25]**	**Remote/Virtual/Telemedicine "Visits" allowed for non-dosing visits. Please refer to protocol schedule of assessments.**	**X**			**X**			**X**	**X**	**X**		
Table continued on next page												

**AE/SAE[26]**	**Remote/Virtual/Telemedicine "Visits" allowed for non-dosing visits. Please refer to protocol schedule of assessments.**	**X**			**X**			**X**	**X**	**X**		

ADA: antidrug antibody; AE: adverse event; βhCG: beta human chorionic gonadotropin; C1D1: Cycle 1 Day 1; CAPOX: capecitabine and oxaliplatin; CLDN: claudin; CT: computerized tomography; DPD: dihydropyrimidine dehydrogenase; eCOA: electronic Clinical Outcomes Assessment; ECG: electrocardiogram; ECOG: Eastern Cooperative Oncology Group; eCRF: electronic case report form; FFPE: formalin fixed paraffin embedded; HRQoL: health-related quality of life; HRU: Health Resource Utilization; ICF: informed consent form: INR: international normalized ratio; IRC: independent review committee; IRR: infusion-related reaction; IRT: interactive response technology; IV: intravenous; MRI: magnetic resonance imaging; OS: overall survival; PD: progressive disease; PFS: progression free survival; PFS2: progression free survival following subsequent anticancer treatment; PGx: pharmacogenomics; PT: prothrombin time; PTT: partial thromboplastin time; RECIST: Response Evaluation Criteria In Solid Tumors; SAE: serious adverse event; SoC: standard of care; T4: thyroxine; TSH: thyroid stimulating hormone

*** +7 calendar day visit window does not apply to C1D1.**

Footnotes continued on next page

1. **Screening: The Screening period is 45 days from full main ICF signature. Retesting of lab values is allowed within the 45-day Screening period. Re-screening outside the 45-day window under a new subject number may be allowed once and upon discussion with the medical monitor.**
   - **Optional partial screening: A partial screening ICF may be available for central testing of tissue for CLDN18.2 only.**
   - **Laboratory testing:**
     - **Eligibility can be determined based on central and/or local laboratory testing; however:**
       - **The most recent laboratory data must be used to confirm the subject's eligibility.**
       - **Central labs must be collected and submitted to the central laboratory during the Screening period.**
       - **If retesting of lab values is necessary to confirm eligibility, local labs can be used without requiring additional sample collection for central laboratory submission.**
     - **The screening labs used to determine eligibility should be collected within 14 days prior to randomization.**
   - **Radiologic imaging used to confirm eligibility must be conducted within 28 days prior to randomization.**
2. **CLDN18.2 Testing: FFPE tumor tissue will be collected for central testing to determine CLDN18.2 status. Archival tumor tissue from the primary tumor (gastric or GEJ) is preferred. If primary tumor tissue is not available, tumor tissue from a metastatic site (excluding bone metastasis) may be used. Five FFPE unstained slides are required as allowed per local policy. The slides should be freshly cut from the FFPE block within the time frame described in the laboratory manual. If the specimen is insufficient or unavailable, a biopsy may be performed to obtain primary tumor tissue (preferred) or tumor tissue from metastatic site (excluding bone metastasis). Sponsor pre-approval is required when the sole purpose of the biopsy procedure is to assess eligibility for this study. See [Section 5.7.1 Tumor Tissue Samples].**
3. **Confirmation of Inclusion/Exclusion Criteria must be completed prior to randomization.**
4. **Randomization: After confirmation of eligibility, the blinded site user will perform the randomization IRT transaction. The unblinded pharmacist/designee will be notified by the IRT system about the randomly assigned treatment. Randomization may be performed prior to C1D1. If C1D1 cannot be performed within 5**

calendar days from Randomization please contact the Medical Monitor for discussion. Details of infusion preparation and storage requirements are defined in the Pharmacy Manual and Infusion Guidelines.

5. **<u>Antiemetic Pretreatment</u>: Prophylactic antiemetics should be given according to institutional standard of care, published guidelines and the respective product package insert(s). All antiemetic premedication should be initiated at a <u>minimum of 30 minutes</u> prior to treatment. For further details, see [Section 5.1.1.2 Antiemetics].**
6. **<u>Zolbetuximab/placebo</u> will be administered as a minimum 2-hour intravenous infusion every 3 weeks starting on C1D1. Please refer to Pharmacy Manual and Infusion Guidelines for more detailed information. Zolbetuximab/placebo should be administered prior to CAPOX. For further details, see [Section 5.1.1.1 Zolbetuximab/Placebo].**

Footnotes continued on next page

7. **<u>Post-infusion Observation Period</u>: Following the first dose of zolbetuximab/placebo on C1D1, the subject must be observed for 2 hours post-zolbetuximab/placebo infusion. The post-infusion observation period can be conducted during the CAPOX administration. If any ≥ grade 2 AEs are observed during infusion or during the post-infusion observation period, subsequent zolbetuximab/placebo infusion times should be extended and subjects should continue to be observed for 2 hours post zolbetuximab/placebo infusion. If the subject does not develop any grade ≥ 2 AEs, the subject should be observed for 1 hour post-infusion for their subsequent zolbetuximab/placebo infusions. The subject should be instructed to notify site personnel if they develop any AEs during this observation time period. In the event of an IRR with features of anaphylaxis (regardless of grade) or grade 3 or 4 IRR, blood samples for cytokine/chemokine panel and serum total tryptase level (levels typically peak within 3 hours after the onset of symptoms) should be collected once the subject has stabilized, for shipment to the central laboratory. See Observation Period following zolbetuximab/placebo infusion [Section 5.4.2] for further details.**
8. **<u>CAPOX</u> is a combination of oxaliplatin intravenous infusion and capecitabine tablets and will be administered starting at C1D1 for up to 8 treatments. See [Section 5.1.1.3].**
9. **<u>Physical Exam</u>: should include height (at Screening only), <u>weight</u> and <u>ECOG performance status</u>. A full physical exam is required at Screening. The physical exam only needs to be repeated on C1D1 if clinically significant changes from screening are observed (in the opinion of the investigator). Targeted (symptom driven) physical exams should be conducted every 3 weeks on day 1 of each cycle. For further details, see [Section 5.4.4 Physical Examination]**
10. **<u>Vital signs</u> (pulse, blood pressure, temperature) should be taken during every visit at the following time points (see [Section 5.4.1 Vital Signs]):**
    - **Predose at every visit**
    - **C1D1: Every 30 (±10) minutes during zolbetuximab/placebo infusion**
    - **Subsequent zolbetuximab/placebo infusions: every 60 (±10) minutes during zolbetuximab/placebo infusions if the subject did not develop any ≥ grade 2 AEs during the C1D1 zolbetuximab/placebo infusion or Post-infusion Observation Period.**
    - **Every 60 (±10) minutes post zolbetuximab/placebo infusion during the Post-infusion Observation Period (for 1 or 2 hours. See footnote 7)**
    - **Unscheduled if clinically indicated**

11. **ECGs: ECGs will be locally assessed. When collected on the same day, ECG should be collected prior to pharmacokinetic samples. For further details, see [Section 5.4.5 Electrocardiogram]. A single ECG will be performed at the following time points:**
    - **Screening**
    - **Up to 48 hours prior to every oxaliplatin infusion (before any antiemetic treatment administration)**
    - **Up to 6 hours following completion of every oxaliplatin infusion**
    - **Zolbetuximab/placebo Discontinuation Visit**
    - **Zolbetuximab/placebo 30-day Follow-up Visit**
    - **If clinically indicated or per local requirements**

Footnotes continued on next page

12. **Imaging Assessments: Radiologic imaging will be evaluated at Screening (must be conducted within 28 days prior to randomization) and every 9 weeks (± 7 days) counting from C1D1 for the first 54 weeks and then every 12 weeks (± 7 days) thereafter until subject develops radiological disease progression per RECIST 1.1 by IRC or starts other systemic anticancer treatment, whichever comes earlier. Imaging schedule should be maintained regardless of treatment delay. Imaging will include CT scans with contrast of the thorax, abdomen, and pelvis. If CT scan with contrast is medically not feasible, MRI may be used for imaging. Bone scans (or focal X-ray) or brain imaging should be performed if metastatic disease in bone or brain is suspected, respectively. The same mode of imaging should be utilized throughout the study unless medical necessity requires a change. For randomized subjects, screening imaging should be sent to the central imaging vendor no later than at the time of submission of the first on-treatment imaging. All imaging acquired post randomization will be sent to the central imaging vendor within 7 days of scanning for the blinded independent central assessment of radiological tumor response based on RECIST 1.1. The investigator should make every effort to immediately submit radiologic assessments for IRC review when PD is suspected. See [Section 5.3 Efficacy Assessments]. Refer to Imaging Acquisition Guidelines for further detail on scan modality and contrast options.**
13. **Laboratory Assessments: See [Section 5.4.3 Laboratory Assessments] for list of laboratory assessments. Laboratory tests must be sent to the central laboratory for analysis. For screening/eligibility laboratory assessments, see footnote number 1.**
    - **Laboratory test results (central or local) will be reviewed by the investigator prior to any study treatment. Clinical significance of out-of-range laboratory findings is to be determined and documented by the investigator/sub-investigator who is a qualified physician.**
    - **Local laboratory results may be used for treatment decisions; however, central laboratory samples should also be drawn per protocol and sent to the central laboratory unless otherwise approved by the sponsor.**
    - **Labs may be collected up to 48 hours prior to study treatment.**
    - **Holidays and weekends should be taken into account when scheduling these blood draws.**
    - **Additional assessments may be done centrally or locally to monitor AEs or as clinically indicated.**
14. **Coagulation (PT, PTT and INR): Coagulation tests should be done at Screening and during study treatment period if clinically indicated. Local or central lab results may be used to confirm eligibility. Ongoing evaluation should be continued for subjects who are receiving therapeutic anticoagulation according to local standard of care. See [Section 5.4.3 Laboratory Assessments].**
15. **Serum Pregnancy Test: Serum pregnancy tests will be collected for female subjects of childbearing potential only. Serum pregnancy tests collected at Screening, during study treatment period and if clinically indicated or per local requirements. (Note: For Screening, subjects with elevated serum βHCG and a demonstrated non-pregnant status through additional testing are eligible.)**

16. Urine Pregnancy Test: Urine pregnancy tests will be collected for female subjects of childbearing potential only. Local urine pregnancy tests to be performed during the treatment period every 3 weeks on day 1 of each cycle and at the zolbetuximab/placebo Study Treatment Discontinuation and 30-day Safety Follow-up Visits. Additional urine pregnancy testing for 6 months after the final study treatment administration may be conducted based on local requirements.

17. HRQoL and HRU questionnaires: eCOA questionnaires should be completed by the subject at Screening (except for HRU), on day 1 of each cycle (or up to 48 hours) before any antiemetic or drug treatment and before the disease status is discussed with the subject, using the electronic tablet device provided. When completion by the subject is not possible, the questionnaires may be administered to the subject by site personnel using the electronic tablet device. For subjects with low literacy or situations where required translation is not available, please contact the sponsor for further guidance.

Footnotes continued on next page

18. Study Treatment Discontinuation Visit (End of Study Treatment): The Study Treatment Discontinuation Visit will take place ≤ 7 days following the decision to discontinue study treatment (zolbetuximab/placebo and CAPOX [both components]). If zolbetuximab/placebo and CAPOX (both components) are discontinued on a different day, subjects will have separate Study Treatment Discontinuation Visits following each treatment's discontinuation. Laboratory tests must be sent to the central laboratory for analysis. HRQoL and HRU questionnaires are not required at CAPOX treatment discontinuation visit. A combined visit can be completed if zolbetuximab/placebo are discontinued on the same day.

19. 30-day Safety Follow-up Visit: A 30-day Safety Follow-up Visit should occur 30 days after the last dose of zolbetuximab/placebo and will include the assessments as shown in the Schedule of Assessments above. A 30-day Safety Follow-up Visit should occur 30 days after the last dose of CAPOX (both components) and may be conducted by phone if the subject is unable to visit the site and will require contact for AE/SAE collection only. HRQoL and HRU questionnaires are not required at CAPOX 30-day safety follow up visit. A combined visit can be completed if zolbetuximab/placebo and both components of CAPOX are discontinued on the same day and HRQoL and HRU questionnaires should be completed for a combined visit.

20. 90-day Safety Follow-up Visits: A 90-day Safety Follow-up Visit should occur 90 days after the last dose of zolbetuximab/placebo and will include the assessments as shown in the Schedule of Assessments above. A 90-day Safety Follow-up Visit should occur 90 days after the last dose of CAPOX (both components) and may be conducted by phone if the subject is unable to visit the site and will require contact for AE/SAE collection only. HRQoL and HRU questionnaires are not required at CAPOX 90-day safety follow up visit. A combined visit can be completed if zolbetuximab/placebo and both components of CAPOX are discontinued on the same day and HRQoL and HRU questionnaires should be completed for a combined visit.

21. Post-treatment Follow-up: if a subject discontinues all study treatments (zolbetuximab/placebo and both components of CAPOX) prior to IRC-confirmed radiological disease progression, the subject will enter the Post-treatment Follow-up Period and continue to undergo imaging assessments every 9 weeks (±7 days) (or every 12 weeks [±7 days] if subject has been on study over 54 weeks) until radiologic disease progression (i.e., PFS) or the subject starts subsequent anticancer treatment, whichever occurs earlier. If study treatments (zolbetuximab/placebo and both components of CAPOX) are discontinued due to PD, the subject will enter the Long-term and Survival Follow-up Period.

22. Long-term and Survival Follow-up Period: Following disease progression on first-line treatment or start of subsequent anticancer treatment, subjects will be followed in the Long-term and Survival Follow-up Period per institutional guidelines, but not less than every 12 weeks. Radiologic imaging will be done per standard of care and read locally until PFS2 is documented. Survival Follow-up Period will continue until death (from any cause). All post-progression details including subsequent anticancer treatment and date and site of progression will be recorded on the eCRF. Subject contact by phone or other remote methods is sufficient during Long-term and Survival Follow-up.

Footnotes continued on next page

23. **Pharmacokinetics: Serum samples for zolbetuximab/placebo will be taken at the below time points and sent to the central laboratory. The date and time of each blood sample collection will be recorded to the nearest minute.**
    - **Cycle 1 Day 1: End of zolbetuximab/placebo infusion**
    - **Cycle 2 Day 1: Predose**
    - **Cycle 3 Day 1: End of zolbetuximab/placebo infusion**
    - **Predose on Day 1 of Cycles 5, 9, 13 and 17**
    - **Zolbetuximab/placebo 30-Day Safety Follow-up visit**
    - **Zolbetuximab/placebo 90-Day Safety Follow-up visit**
    - **Unscheduled pharmacokinetic blood samples may be taken at any time during the study to evaluate drug exposure following a safety event**

    Pharmacokinetic Sampling Window:
    - **Predose: within 60 minutes prior to dosing**
    - **End of Infusion: within 10 minutes after the end of the infusion**
24. **ADA: Blood samples (Serum) for ADA will be taken at the below time points and sent to the central laboratory.**
    - **Cycle 1 Day 1: Predose**
    - **Cycle 2 Day 1: Predose**
    - **Predose on Day 1 of Cycles 5, 9, 13 and 17**
    - **Zolbetuximab/placebo 30-Day Safety Follow-up visit**
    - **Zolbetuximab/placebo 90-Day Safety Follow-up visit**

    ADA Sampling Window: Predose: within 60 minutes prior to dosing
25. **Concomitant medications will be collected from the time of full main informed consent through 90 days following the last dose of study treatment.**
26. **AEs/SAEs: AEs and SAEs (regardless of causality) will be collected from the time of full main informed consent through 90 days following the last dose of study treatment. See [Section 5.5.5 Reporting of Serious Adverse Events].**

IMP SUPPLY

If any of the conditions outlined above in the Participants Procedures Assessment are met, one or all of the following mitigating strategies will be employed, as needed, to ensure continuity of IMP supply to the participants:

- **Increase stock of IMP on site to reduce number of shipments required, if site space will allow, as cold storage space is needed.**

DATA COLLECTION REQUIREMENTS

Additional data may be collected in order to indicate how participation in the study may have been affected by a crisis and to accommodate data collection resulting from alternate measures implemented to manage the conduct of the study and participant safety.

- **Critical assessments for safety and efficacy based on study endpoints to be identified as missing or altered (performed virtually, at alternative locations, out of window, or other modifications) due to the crisis.**

14 COORDINATING INVESTIGATOR'S SIGNATURE

A Phase 3, Global, Multi-Center, Double-Blind, Randomized, Efficacy Study of Zolbetuximab (IMAB362) Plus CAPOX Compared with Placebo Plus CAPOX as First-line Treatment of Subjects with Claudin (CLDN)18.2-Positive, HER2-Negative, Locally Advanced Unresectable or Metastatic Gastric or Gastroesophageal Junction (GEJ) Adenocarcinoma

ISN/Protocol 8951-CL-0302

Version 4.1 [CN] Incorporating Country-specific Nonsubstantial Amendment 2

11 Jan 2021

I have read all pages of this clinical study protocol for which Astellas is the sponsor. I agree that it contains all the information required to conduct this study.

Coordinating Investigator:

Signature: ______________________________ ______________________
<Insert name, department/affiliation, name of institution> Date (DD Mmm YYYY)

Printed Name: ______________________________

Address: ______________________________

____

15 SPONSOR'S SIGNATURES

Astellas Signatories

(Electronic signatures are attached at the end of the document)

PPD	Development Medical Science - Oncology
PPD	Biostatistics

13 ATTACHMENT 1: NONSUBSTANTIAL AMENDMENT 1

I. The purpose of this amendment is:

**Non-Substantial Changes**
**1. Adjust Dosing Sequence Language**
DESCRIPTION OF CHANGE:
Relaxed requirement around dosing sequence to say that zolbetuximab/placebo should be administered before chemotherapy.
RATIONALE:
There is no significant impact to subject safety anticipated with sequence of dosing administration.
**2. Update to Dose Modification Tables**
DESCRIPTION OF CHANGE:
Clarify that dose modification must be applied regardless of relationship to the drug.
RATIONALE:
Language added to assist in understanding of application of dose modifications irrespective of causality.
**3. Clarifications to Cytokine/Chemokines/Tryptase Collection**
DESCRIPTION OF CHANGE:
Added clarification that cytokines/chemokines/tryptase need to be collected centrally.
RATIONALE:
To assist in understanding of required lab study procedures when infusion-related reaction criteria is met.
**4. Changed Lab Collection Window Before Cycle 1 Day 1**
DESCRIPTION OF CHANGE:
Changed lab collection on Cycle 1 Day 1 48-hour window to start at Cycle 1 Day 1.
RATIONALE:
This change is made to define a clinically reasonable window for pre-dosing lab collection.

**5. Update Adverse Event and Conmed Collection to Begin at Full Informed Consent Form**
DESCRIPTION OF CHANGE:
Defined adverse event (AE) collection to begin at the time of full informed consent form (ICF).
RATIONALE:
Clarification for AE and Conmed collection to begin at time of full ICF signature as opposed to Partial Screening ICF signature.
**6. Updated Electronic Clinical Outcomes Assessment Visit Language**
DESCRIPTION OF CHANGE:
Clarified that electronic clinical outcomes assessments (eCOAs) only need to be collected for zolbetuximab/placebo visits if CAPOX is discontinued at a different timepoint.
RATIONALE:
To clarify study eCOA assessments are only collected on zolbetuximab/placebo treatment visits, end of treatment, and 30 and 90 Day Safety Follow-up Visits.
**7. Updated Window for the 30-Day Safety Follow-up Visit**
DESCRIPTION OF CHANGE:
Updated window for 30-Day Safety Follow-up Visit from + 7 days to ± 7 days.
RATIONALE:
To establish a larger visit window for the 30 Day Safety Follow-up Visit and align with the visit window for the 90 Day Safety Follow-up Visit.
**8. Adjusted Language Around CAPOX Dose Re-escalation**
DESCRIPTION OF CHANGE:
Adjusted restrictions around CAPOX dose re-escalation after dose adjustment due to drug-related AEs.
RATIONALE:
To indicate CAPOX dose re-escalation is not recommended if the AE was related to CAPOX.

**9. Clarified Discontinuation Criteria and Dosing Guidance**
DESCRIPTION OF CHANGE:
Clarified discontinuation criteria and dosing guidance for zolbetuximab/placebo and chemotherapy if greater than 28 days from next planned dose passes.
RATIONALE:
To assist in understanding of study requirement for discontinuation related to dose interruption duration.
**10. Clarified the Maximum Number of Oxaliplatin Doses Allowed**
DESCRIPTION OF CHANGE:
Added language to make the maximum number of oxaliplatin doses allowed more clear.
RATIONALE:
To emphasize the maximum number of oxaliplatin treatments per oxaliplatin SmPC.
**11. Added Infusion Rate Adjustment to Table 2**
DESCRIPTION OF CHANGE:
Added instruction to reduce the infusion rate in the guidance for nausea/vomiting management.
RATIONALE:
Additional AE management guidance for common AEs observed during study drug infusion.
**12. Adjusted Language Around Zolbetuximab Infusion**
DESCRIPTION OF CHANGE:
Clarified timing of zolbetuximab infusion.
RATIONALE:
Removed maximum infusion time frame to align with revised zolbetuximab stability requirements in Investigator's Brochure version 5.0.

**13. Added Appendix 12.9**
DESCRIPTION OF CHANGE:
Added Appendix 12.9 to detail accepted alternate approaches during times when the standard procedures cannot be followed.
RATIONALE:
To outline procedures to be prioritized and alternate methods of assessing safety and efficacy in the event of disruptions to trial operations at study sites.
**14. Adjusted Vaccine Restrictions**
DESCRIPTION OF CHANGE:
Added language regarding potential COVID-19 vaccination.
RATIONALE:
Language added to provide guidance for cases where live vaccine is needed for COVID-19 prevention.
**15. Adjusted Oxaliplatin Dose Modification for Associated Neurotoxicity**
DESCRIPTION OF CHANGE
Adjusted dose modification after acute laryngopharyngeal dysesthesia.
RATIONALE
For clarity on AE management.
**16. Adjusted Language Around Placebo Infusion**
DESCRIPTION OF CHANGE
Provided additional language around the sodium chloride injection for use as placebo.
RATIONALE
To clarify use of 0.9% sodium chloride injection for both treatment arms and sourcing by investigational sites.

**17. Minor Administrative-type Changes**
DESCRIPTION OF CHANGE:
Include minor administrative-type changes (e.g., typos, format, numbering and consistency throughout the protocol).
RATIONALE:
To provide clarifications to the protocol and to ensure complete understanding of study procedures.

II. Amendment Summary of Changes:

**IV Synopsis, Study Design Overview**<br>*2.2.1 Study Design*
WAS:
<u>Screening:</u><br>The Screening period is 45 days from informed consent form (ICF) signature.<br><br>AND<br><br>An optional partial screening ICF is available to allow central testing of tissue for CLDN18.2 and HER2 only.
IS AMENDED TO:
<u>Screening:</u><br>The Screening period is 45 days from **full main** informed consent form (ICF) signature.<br><br>AND<br><br>An optional partial screening ICF ~~is~~ **may be** available to allow central testing of tissue for CLDN18.2 and HER2 only.

**IV Synopsis, Study Design Overview**<br>*2.2.1 Study Design*
WAS:
<u>Treatment Period:</u><br>Subjects will also receive up to 8 treatments of CAPOX treatment. Oxaliplatin is administered on day 1 of each cycle, whereas capecitabine is taken twice daily on days 1 through 14. After 8 treatments, subjects may continue to receive capecitabine taken twice daily on days 1 through 14 of each cycle at the investigator’s discretion until the subject meets study treatment discontinuation criteria.

IS AMENDED TO:
Treatment Period:<br>Subjects will also receive up to 8 treatments of CAPOX treatment. Oxaliplatin is administered on day 1 of each cycle, whereas capecitabine is taken twice daily on days 1 through 14. After **a maximum of** 8 treatments **of oxaliplatin**, subjects may continue to receive capecitabine taken twice daily on days 1 through 14 of each cycle at the investigator's discretion until the subject meets study treatment discontinuation criteria.

**IV Synopsis, Investigational Product(s), Zolbetuximab/Placebo**
WAS:
Placebo: Sites should use 0.9 % Sodium Chloride Injection as placebo.
IS AMENDED TO:
Placebo: **Placebo will not be manufactured or provided by the sponsor. 0.9% Sodium Chloride Injection will be used for placebo treatment arm as a placebo infusion solution.** ~~Sites should use 0.9 % Sodium Chloride Injection as placebo~~.

**IV Synopsis, Investigational Product(s), Dosing Schedule**
WAS:
Zolbetuximab/placebo to be administered after antiemetic premedication but prior to CAPOX.
IS AMENDED TO:
Zolbetuximab/placebo ~~to~~ **should** be administered after antiemetic premedication but prior to CAPOX.

**IV Synopsis, Investigational Product(s), Mode of Administration**
WAS:
Intravenous infusion of zolbetuximab/placebo as a minimum 2-hour infusion. It is recommended that zolbetuximab/placebo infusion not exceed 6 hours from start of infusion.<br>Intravenous infusion may be slowed or interrupted to manage toxicity. Please refer to Pharmacy Manual and Infusion Guidelines for more detailed information, if zolbetuximab/placebo infusion cannot be completed.

IS AMENDED TO:
Intravenous infusion of zolbetuximab/placebo as a minimum 2-hour infusion. ~~It is recommended that zolbetuximab/placebo infusion not exceed 6 hours from start of infusion.~~<br>Intravenous infusion may be slowed or interrupted to manage toxicity. Please refer to Pharmacy Manual and Infusion Guidelines for more detailed information~~, if zolbetuximab/placebo infusion cannot be completed~~.

**IV Synopsis, Other Product(s), 0.9% Sodium Chloride Injection**
ADDED:
0.9% Sodium Chloride Injection will be used for infusion solution in this study for both zolbetuximab arm and placebo arm. Details of preparation of infusion solution are provided in the Pharmacy Manual and Infusion Guidelines.<br>***SPECIFIC TO JAPAN:*** In this study, 0.9% Sodium Chloride Injection is not considered an 'investigational product' as defined in J-GCP.

**IV Synopsis, Other Product(s), Oxaliplatin**
WAS:
CAPOX is to be administered after zolbetuximab/placebo infusion.
IS AMENDED TO:
CAPOX ~~is to~~ **should** be administered after zolbetuximab/placebo infusion.

**IV Synopsis, Other Product(s), Oxaliplatin**
WAS:
130 mg/m^2 intravenous infusion on day 1 of each cycle over 2 hours (or longer per institutional standard of care) for up to 8 treatments.
IS AMENDED TO:
130 mg/m^2 intravenous infusion on day 1 of each cycle over 2 hours (or longer per institutional standard of care) for ~~up to~~ **a maximum of** 8 treatments.

**IV Synopsis, Other Product(s), Antiemetic Pre-medications**
WAS:
• All antiemetic premedication should be given at minimum 30 minutes prior to zolbetuximab/placebo treatment.

IS AMENDED TO:

- All antiemetic premedication should be ~~given~~ **initiated** at **a** minimum **of** 30 minutes prior to zolbetuximab/placebo treatment.

IV Synopsis, Concomitant Medication Restrictions or Requirements, Prohibited Concomitant Treatment

WAS:

- Live vaccines should be avoided during the treatment period in which subject is receiving capecitabine and up to 6 months after final capecitabine dose.

IS AMENDED TO:

- Live vaccines should be avoided during the treatment period in which subject is receiving capecitabine and up to 6 months after final capecitabine dose. **In cases where a live vaccine is needed for COVID-19 prevention, please contact the Medical Monitor for discussion.**

IV Synopsis, Concomitant Medication Restrictions or Requirements, Cautionary Concomitant Treatment

WAS:

The following should be avoided or used with caution and closely monitored during CAPOX administration:

- CytochromeP450 (CYP) 2C9 substrates (Subjects taking coumarin-derivative anticoagulants concomitantly with capecitabine should have PT/INR monitored regularly and anticoagulant dose adjusted accordingly).
- Anti-epileptic medications (e.g. phenobarbital, phenytoin and primidone)
- Medications known to prolong the QT or QTc interval (refer to https://www.crediblemeds.org for a list of these medications)

IS AMENDED TO:

The following should be avoided or used with caution and closely monitored during ~~CAPOX~~ **capecitabine** administration:

- CytochromeP450 (CYP) 2C9 substrates (Subjects taking coumarin-derivative anticoagulants concomitantly with capecitabine should have PT/INR monitored regularly and anticoagulant dose adjusted accordingly).
- Anti-epileptic medications (e.g. phenobarbital, phenytoin and primidone)

The following should be avoided or used with caution and closely monitored during oxaliplatin administration:

- Medications known to prolong the QT or QTc interval (refer to https://www.crediblemeds.org for a list of these medications)

**IV Synopsis, Study Treatment Discontinuation Criteria**
WAS:
• Subject has a delay of zolbetuximab/placebo and both components CAPOX for > 28 days from when the next zolbetuximab/placebo and both components CAPOX treatment was scheduled to be administered.
IS AMENDED TO:
• Subject has a delay of zolbetuximab/placebo and both **of** components CAPOX for > 28 days from when the next zolbetuximab/placebo and both components **of** CAPOX treatment was scheduled to be ~~administered~~ **begin (> 49 days from when the last dose of zolbetuximab/placebo and both components of CAPOX treatment began).**

V Schedule of Assessments

WAS:

VISIT	Screening[1]	Study Treatment Period (Each Cycle = approximately 21 Days)						Follow-up Period				
		**Cycles 1-8**<br>Zolbetuximab/Placebo<br>+<br>CAPOX			**Cycle 9+**<br>Zolbetuximab/Placebo<br>+<br>Capecitabine<br>(at investigator discretion)			Study Treatment Discontinuation Visit[18]	30-Day Safety Follow-up Visit(s)[19]	90-Day Safety Follow-up Visit(s)[20]	Post-treatment Follow-up Period [21]	Long-term and Survival Follow-up Periods [22]
**Day**		1	2-14	15-21	1	2-14	15-21					
**Visit Window (calendar days)**	-45 to -1	+7*	(no visit)	(no visit)	+7	(no visit)	(no visit)	+7	+7	±7	±7	±14
Informed Consent	X											
CLDN18.2 Tumor Sample[2]	X											
HER2 Tumor Sample[2]	X											
Biopsy (if applicable)[2]	X											
Medical and Disease History	X											
Confirmation of Inclusion/Exclusion Criteria[3]	X											
Randomization[4]		X										
**Treatments**												
Antiemetic Pretreatment[5]		X			X							
Zolbetuximab/Placebo[6]		X			X							
Post-infusion Observation Period[7]		X			X							
Oxaliplatin } CAPOX[8]		X										
Capecitabine } CAPOX[8]		X	X		X	X						
**Safety Assessments**												
Physical Examination[9]	X	X			X			X	X			
Weight[9]	X	X			X			X	X			
Vital Signs[10]	X	X			X			X	X			
ECOG Performance Status[9]	X	X			X			X				
12-lead ECG[11]	X	X	If clinically indicated and/or per local requirements					X	X			
**Image Assessment[12]**	X[1]	Every 9 weeks ±7 days from C1D1 for the first 54 weeks and then every 12 weeks ±7 days thereafter										

Table continued on next page												
**Subject Contact**												X
**Laboratory Tests**												
Biochemistry[13]	X	X			X			X	X			
TSH and Free T4[13]	X	If clinically indicated						X				
Hematology[13]	X	X			X			X	X			
Coagulation Parameters (PT, PTT and INR)[14]	X	If clinically indicated										
Urinalysis[13]	X	X			X			X	X			
Serum Pregnancy Test[15]	X	If clinically indicated and/or per local requirements										
Urine Pregnancy Test[16]		X			X			X	X			
DPD testing per local requirements	X											
Cytokine/Chemokine and/or Tryptase		If clinically indicated										
**Electronic Clinical Outcomes Assessments (eCOA)**												
HRQoL[17]	X	X			X			X	X	X		
Health Resource Utilization (HRU)[17]		X			X			X	X	X		
**Sampling**												
Pharmacokinetics of Zolbetuximab (Serum)[23]		X			X				X	X		
Antidrug-Antibodies (ADA) for Immunogenicity[24]		X			X				X	X		
Genetic Immune Polymorphisms (Whole Blood)[25]		X										
Exploratory Biomarkers (Serum)[26]		X						X				
Exploratory Biomarkers (Plasma)[26]		X						X				
Whole Blood Sample for PGx (optional)[27]		X										
Post-progression Tumor Sample (optional)[28]								X				
**Concomitant Medication[29]**	X	X			X			X	X	X		
**AE/SAE[30]**	X	X			X			X	X	X		

ADA: antidrug antibody; AE: adverse event; βhCG: beta human chorionic gonadotropin; C1D1: Cycle 1 Day 1; CAPOX: capecitabine and oxaliplatin; CLDN: claudin; CT: computerized tomography; DPD: dihydropyrimidine dehydrogenase; eCOA: electronic Clinical Outcomes Assessment; ECG: electrocardiogram;
ECOG: Eastern Cooperative Oncology Group; eCRF: electronic case report form; FFPE: formalin fixed paraffin embedded; HER2: human epidermal growth factor receptor 2; HRQoL: health-related quality of life; HRU: Health Resource Utilization; ICF: informed consent form: INR: international normalized ratio; IRC: independent review committee; IRR: infusion-related reaction; IRT: interactive response technology; IV: intravenous; MRI: magnetic resonance imaging; OS: overall survival; PD: progressive disease; PFS: progression free survival; PFS2: progression free survival following subsequent anticancer treatment; PGx: pharmacogenomics; PT: prothrombin time; PTT: partial thromboplastin time; RECIST: Response Evaluation Criteria In Solid Tumors; SAE: serious adverse event; T4: thyroxine; TSH: thyroid stimulating hormone

* +7 calendar day visit window does not apply to C1D1.

1. Screening: The Screening period is 45 days from ICF signature. Retesting of lab values is allowed within the 45-day Screening period. Re-screening outside the 45-day window under a new subject number may be allowed once and upon discussion with the medical monitor.
    - Optional partial screening: A partial screening ICF is available for central testing of tissue for CLDN18.2 and HER2 only.
    - Laboratory testing:

- Eligibility can be determined based on central and/or local laboratory testing; **however**:
  - The most recent laboratory data must be used to confirm the subject's eligibility.
  - Central labs must be collected and submitted to the central laboratory during the Screening period.
  - If retesting of lab values is necessary to confirm eligibility, local labs can be used without requiring additional sample collection for central laboratory submission.
- The screening labs used to determine eligibility should be collected within 14 days prior to randomization.
- Radiologic imaging used to confirm eligibility must be conducted within 28 days prior to randomization.

2. CLDN18.2 and HER2 Testing: FFPE tumor tissue will be collected for central testing to determine CLDN18.2 and HER2 status. Archival tumor tissue from the primary tumor (gastric or GEJ) is preferred. If primary tumor tissue is not available, tumor tissue from a metastatic site (excluding bone metastasis) may be used. A minimum of 1 FFPE tumor tissue block (preferred) OR a minimum of 15 FFPE unstained slides are required as allowed per local policy. If slides are submitted, the slides should be freshly cut from the FFPE block within the time frame described in the laboratory manual. If local HER2 results are already available from local testing, a minimum of 12 FFPE unstained slides are required to be submitted to the central lab as allowed per local policy. If the specimen is insufficient or unavailable, a biopsy may be performed to obtain primary tumor tissue (preferred) or tumor tissue from metastatic site (excluding bone metastasis). Sponsor pre-approval is required when the sole purpose of the biopsy procedure is to assess eligibility for this study. If the required number of slides cannot be provided, the sponsor or designee should be contacted for further guidance. See [Section 5.7.3 Tumor Tissue Samples].
3. Confirmation of Inclusion/Exclusion Criteria must be completed prior to randomization.
4. Randomization: After confirmation of eligibility, the blinded site user will perform the randomization IRT transaction. The unblinded pharmacist/designee will be notified by the IRT system about the randomly assigned treatment. Randomization may be performed prior to C1D1. Details of infusion preparation and storage requirements are defined in the Pharmacy Manual and Infusion Guidelines.
5. Antiemetic Pretreatment: Prophylactic antiemetics should be given according to institutional standard of care, published guidelines and the respective product package insert(s). All antiemetic premedication should be given at minimum 30 minutes prior to treatment. For further details, see [Section 5.1.1.2 Antiemetics].

Footnotes continued on next page

6. Zolbetuximab/placebo will be administered as a minimum 2-hour intravenous infusion every 3 weeks starting on C1D1. It is recommended that zolbetuximab/placebo infusion not exceed 6 hours from start of infusion. Please refer to Pharmacy Manual and Infusion Guidelines for more detailed information, if zolbetuximab/placebo cannot be completed. Zolbetuximab/placebo should be administered prior to CAPOX. For further details, see [Section 5.1.1.1 Zolbetuximab/Placebo].
7. Post-infusion Observation Period: Following the first dose of zolbetuximab/placebo on C1D1, the subject must be observed for 2 hours post zolbetuximab/placebo infusion. The post-infusion observation period can be conducted during the CAPOX administration. If any ≥ grade 2 AEs are observed during infusion or during the post-infusion observation period, subsequent zolbetuximab/placebo infusion times should be extended and subjects should continue to be observed for 2 hours post zolbetuximab/placebo infusion. If the subject does not develop any grade ≥ 2 AEs, the subject should be observed for 1 hour post-infusion for their subsequent zolbetuximab/placebo infusions. The subject should be instructed to notify site personnel if they develop any AEs during this observation time period. In the event of an IRR with features of anaphylaxis (regardless of grade) or grade 3 or 4 IRR, blood samples for cytokine/chemokine panel and serum total tryptase level (levels typically peak within 3 hours after the onset of symptoms) should be collected once the subject has stabilized, for shipment to the central laboratory. See Observation Period following zolbetuximab/placebo [Section 5.4.2] for further details.
8. CAPOX is a combination of oxaliplatin intravenous infusion and capecitabine tablets and will be administered starting at C1D1 for up to 8 treatments. See [Section 5.1.1.3].
9. Physical Exam: should include height (at Screening only), weight and ECOG performance status. A full physical exam is required at Screening. The physical exam only needs to be repeated on C1D1 if clinically significant changes from screening are observed (in the opinion of the investigator). Targeted (symptom driven) physical exams should be conducted every 3 weeks on day 1 of each cycle. For further details, see [Section 5.4.4 Physical Examination]
10. Vital signs (pulse, blood pressure, temperature) should be taken during every visit at the following time points (see [Section 5.4.1 Vital Signs]):
    - Predose at every visit
    - C1D1: Every 30 (±10) minutes during zolbetuximab/placebo infusion
    - Subsequent zolbetuximab/placebo infusions: every 60 (±10) minutes during zolbetuximab/placebo infusions if the subject did not develop any ≥ grade 2 AEs during the C1D1 zolbetuximab/placebo infusion or Post-infusion Observation Period.
    - Every 60 (±10) minutes post zolbetuximab/placebo infusion during the Post-infusion Observation Period (for 1 or 2 hours. See footnote 7)
    - Unscheduled if clinically indicated
11. ECGs: ECGs will be locally assessed. When collected on the same day, ECG should be collected prior to pharmacokinetic samples. For further details, see [Section 5.4.5 Electrocardiogram]. A single ECG will be performed at the following time points:
    - Screening
    - Up to 48 hours prior to every oxaliplatin infusion (before any antiemetic treatment administration)
    - Up to 6 hours following completion of every oxaliplatin infusion
    - Zolbetuximab/placebo Discontinuation Visit
    - Zolbetuximab/placebo 30-day Follow-up Visit
    - If clinically indicated or per local requirements

Footnotes continued on next page

12. Imaging Assessments: Radiologic imaging will be evaluated at Screening (must be conducted within 28 days prior to randomization) and every 9 weeks (± 7 days) counting from C1D1 for the first 54 weeks and then every 12 weeks (± 7 days) thereafter until subject develops radiological disease progression per RECIST 1.1 by IRC or starts other

systemic anticancer treatment, whichever comes earlier. Imaging schedule should be maintained regardless of treatment delay. Imaging will include CT scans with contrast of the thorax, abdomen, and pelvis. If CT scan with contrast is medically not feasible, MRI may be used for imaging. Bone scans (or focal X-ray) or brain imaging should be performed if metastatic disease in bone or brain is suspected, respectively. The same mode of imaging should be utilized throughout the study unless medical necessity requires a change. For randomized subjects, screening imaging should be sent to the central imaging vendor no later than at the time of submission of the first on-treatment imaging. All imaging acquired post randomization will be sent to the central imaging vendor within 7 days of scanning for the blinded independent central assessment of radiological tumor response based on RECIST 1.1. The investigator should make every effort to immediately submit radiologic assessments for IRC review when PD is suspected. See [Section 5.3 Efficacy Assessments]. Refer to Imaging Acquisition Guidelines for further detail on scan modality and contrast options.

13. Laboratory Assessments: See [Section 5.4.3 Laboratory Assessments] for list of laboratory assessments. Laboratory tests must be sent to the central laboratory for analysis. For screening/eligibility laboratory assessments, see footnote number 1.
    - Laboratory test results (central or local) will be reviewed by the investigator prior to any study treatment. Clinical significance of out-of-range laboratory findings is to be determined and documented by the investigator/sub-investigator who is a qualified physician.
    - Local laboratory results may be used for treatment decisions; however, central laboratory samples must also be drawn per protocol and sent to the central laboratory.
    - From cycle 2 onwards central and local labs may be collected up to 48 hours prior to study treatment.
    - Holidays and weekends should be taken into account when scheduling these blood draws.
    - Additional assessments may be done centrally or locally to monitor AEs or as clinically indicated.
14. Coagulation (PT, PTT and INR): Coagulation tests should be done at Screening and during study treatment period if clinically indicated. Local or central lab results may be used to confirm eligibility. Ongoing evaluation should be continued for subjects who are receiving therapeutic anticoagulation according to local standard of care. See [Section 5.4.3 Laboratory Assessments].
15. Serum Pregnancy Test: Serum pregnancy tests will be collected for female subjects of childbearing potential only. Serum pregnancy tests collected at Screening, during study treatment period and if clinically indicated or per local requirements. (Note: For Screening, subjects with elevated serum βHCG and a demonstrated non-pregnant status through additional testing are eligible.) Local or central laboratory results may be used to confirm eligibility.
16. Urine Pregnancy Test: Urine pregnancy tests will be collected for female subjects of childbearing potential only. Local urine pregnancy tests to be performed during the treatment period every 3 weeks on day 1 of each cycle and at the zolbetuximab/placebo Study Treatment Discontinuation and 30-day Safety Follow-up Visits. Additional urine pregnancy testing for 6 months after the final study treatment administration may be conducted based on local requirements.
17. HRQoL and HRU questionnaires: eCOA questionnaires should be administered at Screening (except for HRU), on day 1 of each cycle (or up to 48 hours) before any antiemetic or drug treatment and before the disease status is discussed with the subject. For subjects with low literacy or situations where required translation is not available, please contact the sponsor for further guidance.
18. Study Treatment Discontinuation Visit (End of Study Treatment): The Study Treatment Discontinuation Visit will take place ≤ 7 days following the decision to discontinue study treatment (zolbetuximab/placebo and CAPOX [both components]). If zolbetuximab/placebo and CAPOX (both components) are discontinued on a different day, subjects will have separate Study Treatment Discontinuation Visits following each treatment's discontinuation. Laboratory tests must be sent to the central laboratory for analysis.

Footnotes continued on next page

19. 30-day Safety Follow-up Visit: A 30-day Safety Follow-up Visit should occur 30 days after the last dose of zolbetuximab/placebo and will include the assessments as shown the in the Schedule of Assessments above. A 30-day Safety Follow-up Visit should occur 30 days after the last dose of CAPOX (both components) and may be conducted by phone if the subject is unable to visit the site and will require contact for AE/SAE collection only.

20. 90-day Safety Follow-up Visits: A 90-day Safety Follow-up Visit should occur 90 days after the last dose of zolbetuximab/placebo and will include the assessments as shown the in the Schedule of Assessments above. A 90-day Safety Follow-up Visit should occur 90 days after the last dose of CAPOX (both components) and may be conducted by phone if the subject is unable to visit the site and will require contact for AE/SAE collection only.
21. Post-treatment Follow-up: if a subject discontinues all study treatments (zolbetuximab/placebo and both components of CAPOX) prior to IRC-confirmed radiological disease progression, the subject will enter the Post-treatment Follow-up Period and continue to undergo imaging assessments every 9 weeks (±7 days) (or every 12 weeks [±7 days] if subjects has been on study over 54 weeks) until radiologic disease progression (i.e., PFS) or the subject starts subsequent anticancer treatment, whichever occurs earlier. If study treatments (zolbetuximab/placebo and both components of CAPOX) are discontinued due to PD, the subject will enter the Long-term and Survival Follow-up Period.
22. Long-term and Survival Follow-up Period: Following disease progression on first-line treatment or start of subsequent anticancer treatment, subjects will be followed in the Long-term and Survival Follow-up Period per institutional guidelines, but not less than every 12 weeks. Radiologic imaging will be done per standard of care and read locally until PFS2 is documented. Survival Follow-up Period will continue until death (from any cause). All post-progression details including subsequent anticancer treatment and date and site of progression will be recorded on the eCRF. Subject contact by phone or other remote methods is sufficient during Long-term and Survival Follow-up.
23. Pharmacokinetics: Serum samples for zolbetuximab/placebo will be taken at the below time points and sent to the central laboratory. The date and time of each blood sample collection will be recorded to the nearest minute.
    - Cycle 1 Day 1: End of zolbetuximab/placebo infusion
    - Cycle 2 Day 1: Predose
    - Cycle 3 Day 1: End of zolbetuximab/placebo infusion
    - Predose on Day 1 of Cycles 5, 9, 13 and 17
    - Zolbetuximab/placebo 30-Day Safety Follow-up visit
    - Zolbetuximab/placebo 90-Day Safety Follow-up visit
    - Unscheduled pharmacokinetic blood samples may be taken at any time during the study to evaluate drug exposure following a safety event

    Pharmacokinetic Sampling Window:
    - Predose: within 60 minutes prior to dosing
    - End of Infusion: within 10 minutes after the end of the infusion
24. ADA: Blood samples (Serum) for ADA will be taken at the below time points and sent to the central laboratory.
    - Cycle 1 Day 1: Predose
    - Cycle 2 Day 1: Predose
    - Predose on Day 1 of Cycles 5, 9, 13 and 17
    - Zolbetuximab/placebo 30-Day Safety Follow-up visit
    - Zolbetuximab/placebo 90-Day Safety Follow-up visit

    ADA Sampling Window: Predose: within 60 minutes prior to dosing
25. Genetic Immune Polymorphism: To be collected per local policy. Whole blood sample taken at C1D1 will be sent to the central laboratory.

Footnotes continued on next page

26. Exploratory Biomarker (Serum and Plasma): To be collected per local policy. Samples should be taken at the below time points and sent to the central laboratory:
    - Cycle 1 Day 1: Predose
    - Cycle 2 Day 1: Predose

- Cycle 3 Day 1: Predose
- Cycle 4 Day 1: Predose
- Cycle 5 Day 1: Predose
- Cycle 6 Day 1: Predose
- Cycle 8 Day 1: Predose
- Zolbetuximab/placebo Study Treatment Discontinuation Visit

27. Optional PGx: For subjects who signed a separate ICF, an optional whole blood sample for PGx for exploratory biomarker analysis may be collected prior to first study drug administration. Sample collection is optional and only collected as allowed per local policy.
28. Optional Post-Progression Tumor Sample: For subjects who signed a separate ICF, an optional post-progression tumor sample for exploratory biomarker analysis may be collected following IRC confirmation of disease progression and prior to initiation of subsequent anticancer therapy. Sample collection is optional and only collected as allowed per local policy.
29. Concomitant medications will be collected from the time of informed consent through 90 days following the last dose of study treatment.
30. AEs/SAEs: AEs and SAEs (regardless of causality) will be collected from the time of informed consent through 90 days following the last dose of study treatment. See [Section 5.5.5 Reporting of Serious Adverse Events].

IS AMENDED TO:

VISIT	Screening[1]	Study Treatment Period (Each Cycle = approximately 21 Days)						Follow-up Period				
		**Cycles 1-8** Zolbetuximab/Placebo + CAPOX			**Cycle 9+** Zolbetuximab/Placebo + Capecitabine (at investigator discretion)			Study Treatment Discontinuation Visit[18]	30-Day Safety Follow-up Visit(s)[19]	90-Day Safety Follow-up Visit(s)[20]	Post-treatment Follow-up Period [21]	Long-term and Survival Follow-up Periods [22]
**Day**		1	2-14	15-21	1	2-14	15-21					
**Visit Window (calendar days)**	-45 to -1	+7*	(no visit)	(no visit)	+7	(no visit)	(no visit)	+7	~~+~~±7	±7	±7	±14
Informed Consent	X											
CLDN18.2 Tumor Sample[2]	X											
HER2 Tumor Sample[2]	X											
Biopsy (if applicable)[2]	X											
Medical and Disease History	X											
Confirmation of Inclusion/Exclusion Criteria[3]	X											

Randomization[4]		X										
**Treatments**												
Antiemetic Pretreatment[5]		X			X							
Zolbetuximab/Placebo[6]		X			X							
Post-infusion Observation Period[7]		X			X							
Oxaliplatin — CAPOX[8]		X										
Capecitabine — CAPOX[8]		X	X		X	X						
**Safety Assessments**												
Physical Examination[9]	X	X			X			X	X			
Weight[9]	X	X			X			X	X			
Vital Signs[10]	X	X			X			X	X			
ECOG Performance Status[9]	X	X			X			X				
12-lead ECG[11]	X	X	If clinically indicated and/or per local requirements					X	X			
**Image Assessment[12]**	X[1]	Every 9 weeks ±7 days from C1D1 for the first 54 weeks and then every 12 weeks ±7 days thereafter										
Table continued on next page												
**Subject Contact**												X
**Laboratory Tests**												
Biochemistry[13]	X	X			X			X	X			
TSH and Free T4[13]	X	If clinically indicated						X				
Hematology[13]	X	X			X			X	X			
Coagulation Parameters (PT, PTT and INR)[14]	X	If clinically indicated										
Urinalysis[13]	X	X			X			X	X			
Serum Pregnancy Test[15]	X	If clinically indicated and/or per local requirements										
Urine Pregnancy Test[16]		X			X			X	X			
DPD testing per local requirements	X											
Cytokine/Chemokine and/or Tryptase		If clinically indicated										
**Electronic Clinical Outcomes Assessments (eCOA)**												
HRQoL[17]	X	X			X			X	X	X		
Health Resource Utilization (HRU)[17]		X			X			X	X	X		
**Sampling**												
Pharmacokinetics of Zolbetuximab (Serum)[23]		X			X				X	X		
Antidrug-Antibodies (ADA) for Immunogenicity[24]		X			X				X	X		
Genetic Immune Polymorphisms (Whole Blood)[25]		X										
Exploratory Biomarkers (Serum)[26]		X						X				
Exploratory Biomarkers (Plasma)[26]		X						X				
Whole Blood Sample for PGx (optional)[27]		X										
Post-progression Tumor Sample (optional)[28]								X				
**Concomitant Medication[29]**	X	X			X			X	X	X		

**AE/SAE**[30]	X	X			X			X	X	X		

ADA: antidrug antibody; AE: adverse event; βhCG: beta human chorionic gonadotropin; C1D1: Cycle 1 Day 1; CAPOX: capecitabine and oxaliplatin; CLDN: claudin; CT: computerized tomography; DPD: dihydropyrimidine dehydrogenase; eCOA: electronic Clinical Outcomes Assessment; ECG: electrocardiogram;
ECOG: Eastern Cooperative Oncology Group; eCRF: electronic case report form; FFPE: formalin fixed paraffin embedded; HER2: human epidermal growth factor receptor 2; HRQoL: health-related quality of life; HRU: Health Resource Utilization; ICF: informed consent form: INR: international normalized ratio; IRC: independent review committee; IRR: infusion-related reaction; IRT: interactive response technology; IV: intravenous; MRI: magnetic resonance imaging; OS: overall survival; PD: progressive disease; PFS: progression free survival; PFS2: progression free survival following subsequent anticancer treatment; PGx: pharmacogenomics; PT: prothrombin time; PTT: partial thromboplastin time; RECIST: Response Evaluation Criteria In Solid Tumors; SAE: serious adverse event; T4: thyroxine; TSH: thyroid stimulating hormone

* +7 calendar day visit window does not apply to C1D1.

1. Screening: The Screening period is 45 days from **full main** ICF signature. Retesting of lab values is allowed within the 45-day Screening period. Re-screening outside the 45-day window under a new subject number may be allowed once and upon discussion with the medical monitor.
    - Optional partial screening: A partial screening ICF ~~is~~ **may be** available for central testing of tissue for CLDN18.2 and HER2 only.
    - Laboratory testing:
        - Eligibility can be determined based on central and/or local laboratory testing; **however**:
            - The most recent laboratory data must be used to confirm the subject's eligibility.
            - Central labs must be collected and submitted to the central laboratory during the Screening period.
            - If retesting of lab values is necessary to confirm eligibility, local labs can be used without requiring additional sample collection for central laboratory submission.
        - The screening labs used to determine eligibility should be collected within 14 days prior to randomization.
    - Radiologic imaging used to confirm eligibility must be conducted within 28 days prior to randomization.
2. CLDN18.2 and HER2 Testing: FFPE tumor tissue will be collected for central testing to determine CLDN18.2 and HER2 status. Archival tumor tissue from the primary tumor (gastric or GEJ) is preferred. If primary tumor tissue is not available, tumor tissue from a metastatic site (excluding bone metastasis) may be used. A minimum of 1 FFPE tumor tissue block (preferred) OR a minimum of 15 FFPE unstained slides are required as allowed per local policy. If slides are submitted, the slides should be freshly cut from the FFPE block within the time frame described in the laboratory manual. If local HER2 results are already available from local testing, a minimum of 12 FFPE unstained slides are required to be submitted to the central lab as allowed per local policy. If the specimen is insufficient or unavailable, a biopsy may be performed to obtain primary tumor tissue (preferred) or tumor tissue from metastatic site (excluding bone metastasis). Sponsor pre-approval is required when the sole purpose of the biopsy procedure is to assess eligibility for this study. If the required number of slides cannot be provided, the sponsor or designee should be contacted for further guidance. See [Section 5.7.3 Tumor Tissue Samples].
3. Confirmation of Inclusion/Exclusion Criteria must be completed prior to randomization.
4. Randomization: After confirmation of eligibility, the blinded site user will perform the randomization IRT transaction. The unblinded pharmacist/designee will be notified by the IRT system about the randomly assigned treatment. Randomization may be performed prior to C1D1. **If C1D1 cannot be performed within 5 calendar days from Randomization, please contact the Medical Monitor for discussion.** Details of infusion preparation and storage requirements are defined in the Pharmacy Manual and Infusion Guidelines.

5. Antiemetic Pretreatment: Prophylactic antiemetics should be given according to institutional standard of care, published guidelines and the respective product package insert(s). All antiemetic premedication should be ~~given~~ **initiated** at **a** minimum **of** 30 minutes prior to treatment. For further details, see [Section 5.1.1.2 Antiemetics].

Footnotes continued on next page

6. Zolbetuximab/placebo will be administered as a minimum 2-hour intravenous infusion every 3 weeks starting on C1D1. ~~It is recommended that zolbetuximab/placebo infusion not exceed 6 hours from start of infusion.~~ Please refer to Pharmacy Manual and Infusion Guidelines for more detailed information~~, if zolbetuximab/placebo infusion cannot be completed~~. Zolbetuximab/placebo should be administered prior to CAPOX. For further details, see [Section 5.1.1.1 Zolbetuximab/Placebo].
7. Post-infusion Observation Period: Following the first dose of zolbetuximab/placebo on C1D1, the subject must be observed for 2 hours post zolbetuximab/placebo infusion. The post-infusion observation period can be conducted during the CAPOX administration. If any ≥ grade 2 AEs are observed during infusion or during the post-infusion observation period, subsequent zolbetuximab/placebo infusion times should be extended and subjects should continue to be observed for 2 hours post zolbetuximab/placebo infusion. If the subject does not develop any grade ≥ 2 AEs, the subject should be observed for 1 hour post-infusion for their subsequent zolbetuximab/placebo infusions. The subject should be instructed to notify site personnel if they develop any AEs during this observation time period. In the event of an IRR with features of anaphylaxis (regardless of grade) or grade 3 or 4 IRR, blood samples for cytokine/chemokine panel and serum total tryptase level (levels typically peak within 3 hours after the onset of symptoms) should be collected once the subject has stabilized, for shipment to the central laboratory. See Observation Period following zolbetuximab/placebo **infusion** [Section 5.4.2] for further details.
8. CAPOX is a combination of oxaliplatin intravenous infusion and capecitabine tablets and will be administered starting at C1D1 for up to 8 treatments. See [Section 5.1.1.3].
9. Physical Exam: should include height (at Screening only), weight and ECOG performance status. A full physical exam is required at Screening. The physical exam only needs to be repeated on C1D1 if clinically significant changes from screening are observed (in the opinion of the investigator). Targeted (symptom driven) physical exams should be conducted every 3 weeks on day 1 of each cycle. For further details, see [Section 5.4.4 Physical Examination]
10. Vital signs (pulse, blood pressure, temperature) should be taken during every visit at the following time points (see [Section 5.4.1 Vital Signs]):
    - Predose at every visit
    - C1D1: Every 30 (±10) minutes during zolbetuximab/placebo infusion
    - Subsequent zolbetuximab/placebo infusions: every 60 (±10) minutes during zolbetuximab/placebo infusions if the subject did not develop any ≥ grade 2 AEs during the C1D1 zolbetuximab/placebo infusion or Post-infusion Observation Period.
    - Every 60 (±10) minutes post zolbetuximab/placebo infusion during the Post-infusion Observation Period (for 1 or 2 hours. See footnote 7)
    - Unscheduled if clinically indicated
11. ECGs: ECGs will be locally assessed. When collected on the same day, ECG should be collected prior to pharmacokinetic samples. For further details, see [Section 5.4.5 Electrocardiogram]. A single ECG will be performed at the following time points:
    - Screening
    - Up to 48 hours prior to every oxaliplatin infusion (before any antiemetic treatment administration)
    - Up to 6 hours following completion of every oxaliplatin infusion
    - Zolbetuximab/placebo Discontinuation Visit
    - Zolbetuximab/placebo 30-day Follow-up Visit
    - If clinically indicated or per local requirements

Footnotes continued on next page

12. Imaging Assessments: Radiologic imaging will be evaluated at Screening (must be conducted within 28 days prior to randomization) and every 9 weeks (± 7 days) counting from C1D1 for the first 54 weeks and then every 12 weeks (± 7 days) thereafter until subject develops radiological disease progression per RECIST 1.1 by IRC or starts other systemic anticancer treatment, whichever comes earlier. Imaging schedule should be maintained regardless of treatment delay. Imaging will include CT scans with contrast of the thorax, abdomen, and pelvis. If CT scan with contrast is medically not feasible, MRI may be used for imaging. Bone scans (or focal X-ray) or brain imaging should be performed if metastatic disease in bone or brain is suspected, respectively. The same mode of imaging should be utilized throughout the study unless medical necessity requires a change. For randomized subjects, screening imaging should be sent to the central imaging vendor no later than at the time of submission of the first on-treatment imaging. All imaging acquired post randomization will be sent to the central imaging vendor within 7 days of scanning for the blinded independent central assessment of radiological tumor response based on RECIST 1.1. The investigator should make every effort to immediately submit radiologic assessments for IRC review when PD is suspected. See [Section 5.3 Efficacy Assessments]. Refer to Imaging Acquisition Guidelines for further detail on scan modality and contrast options.
13. Laboratory Assessments: See [Section 5.4.3 Laboratory Assessments] for list of laboratory assessments. Laboratory tests must be sent to the central laboratory for analysis. For screening/eligibility laboratory assessments, see footnote number 1.
    - Laboratory test results (central or local) will be reviewed by the investigator prior to any study treatment. Clinical significance of out-of-range laboratory findings is to be determined and documented by the investigator/sub-investigator who is a qualified physician.
    - Local laboratory results may be used for treatment decisions; however, central laboratory samples must also be drawn per protocol and sent to the central laboratory.
    - ~~From cycle 2 onwards c~~**C**entral and local labs may be collected up to 48 hours prior to study treatment.
    - Holidays and weekends should be taken into account when scheduling these blood draws.
    - Additional assessments may be done centrally or locally to monitor AEs or as clinically indicated.
14. Coagulation (PT, PTT and INR): Coagulation tests should be done at Screening and during study treatment period if clinically indicated. Local or central lab results may be used to confirm eligibility. Ongoing evaluation should be continued for subjects who are receiving therapeutic anticoagulation according to local standard of care. See [Section 5.4.3 Laboratory Assessments].
15. Serum Pregnancy Test: Serum pregnancy tests will be collected for female subjects of childbearing potential only. Serum pregnancy tests collected at Screening, during study treatment period and if clinically indicated or per local requirements. (Note: For Screening, subjects with elevated serum βHCG and a demonstrated non-pregnant status through additional testing are eligible.) Local or central laboratory results may be used to confirm eligibility.
16. Urine Pregnancy Test: Urine pregnancy tests will be collected for female subjects of childbearing potential only. Local urine pregnancy tests to be performed during the treatment period every 3 weeks on day 1 of each cycle and at the zolbetuximab/placebo Study Treatment Discontinuation and 30-day Safety Follow-up Visits. Additional urine pregnancy testing for 6 months after the final study treatment administration may be conducted based on local requirements.
17. HRQoL and HRU questionnaires: eCOA questionnaires should be ~~administered~~ **completed by the subject** at Screening (except for HRU), on day 1 of each cycle (or up to 48 hours) before any antiemetic or drug treatment and before the disease status is discussed with the subject**, using the electronic tablet device provided. When completion by the subject is not possible, the questionnaires may be administered to the subject by site personnel using the electronic tablet device.** For subjects with low literacy or situations where required translation is not available, please contact the sponsor for further guidance.
18. Study Treatment Discontinuation Visit (End of Study Treatment): The Study Treatment Discontinuation Visit will take place ≤ 7 days following the decision to discontinue study treatment (zolbetuximab/placebo and CAPOX [both components]). If zolbetuximab/placebo and CAPOX (both components) are discontinued on a different day, subjects will have separate Study Treatment Discontinuation Visits following each treatment's discontinuation. Laboratory tests must be sent to the central laboratory for analysis.

HRQoL and HRU questionnaires are not required at CAPOX treatment discontinuation visit. A combined visit can be completed if zolbetuximab/placebo are discontinued on the same day.

Footnotes continued on next page

19. 30-day Safety Follow-up Visit: A 30-day Safety Follow-up Visit should occur 30 days after the last dose of zolbetuximab/placebo and will include the assessments as shown ~~the~~ in the Schedule of Assessments above. A 30-day Safety Follow-up Visit should occur 30 days after the last dose of CAPOX (both components) and may be conducted by phone if the subject is unable to visit the site and will require contact for AE/SAE collection only. **HRQoL and HRU questionnaires are not required at CAPOX 30-day safety follow up visit. A combined visit can be completed if zolbetuximab/placebo and both components of CAPOX are discontinued on the same day and HRQoL and HRU questionnaires should be completed for a combined visit.**
20. 90-day Safety Follow-up Visits: A 90-day Safety Follow-up Visit should occur 90 days after the last dose of zolbetuximab/placebo and will include the assessments as shown ~~the~~ in the Schedule of Assessments above. A 90-day Safety Follow-up Visit should occur 90 days after the last dose of CAPOX (both components) and may be conducted by phone if the subject is unable to visit the site and will require contact for AE/SAE collection only. **HRQoL and HRU questionnaires are not required at CAPOX 90-day safety follow up visit. A combined visit can be completed if zolbetuximab/placebo and both components of CAPOX are discontinued on the same day and HRQoL and HRU questionnaires should be completed for a combined visit.**
21. Post-treatment Follow-up: if a subject discontinues all study treatments (zolbetuximab/placebo and both components of CAPOX) prior to IRC-confirmed radiological disease progression, the subject will enter the Post-treatment Follow-up Period and continue to undergo imaging assessments every 9 weeks (±7 days) (or every 12 weeks [±7 days] if subject~~s~~ has been on study over 54 weeks) until radiologic disease progression (i.e., PFS) or the subject starts subsequent anticancer treatment, whichever occurs earlier. If study treatments (zolbetuximab/placebo and both components of CAPOX) are discontinued due to PD, the subject will enter the Long-term and Survival Follow-up Period.
22. Long-term and Survival Follow-up Period: Following disease progression on first-line treatment or start of subsequent anticancer treatment, subjects will be followed in the Long-term and Survival Follow-up Period per institutional guidelines, but not less than every 12 weeks. Radiologic imaging will be done per standard of care and read locally until PFS2 is documented. Survival Follow-up Period will continue until death (from any cause). All post-progression details including subsequent anticancer treatment and date and site of progression will be recorded on the eCRF. Subject contact by phone or other remote methods is sufficient during Long-term and Survival Follow-up.
23. Pharmacokinetics: Serum samples for zolbetuximab/placebo will be taken at the below time points and sent to the central laboratory. The date and time of each blood sample collection will be recorded to the nearest minute.
    - Cycle 1 Day 1: End of zolbetuximab/placebo infusion
    - Cycle 2 Day 1: Predose
    - Cycle 3 Day 1: End of zolbetuximab/placebo infusion
    - Predose on Day 1 of Cycles 5, 9, 13 and 17
    - Zolbetuximab/placebo 30-Day Safety Follow-up visit
    - Zolbetuximab/placebo 90-Day Safety Follow-up visit
    - Unscheduled pharmacokinetic blood samples may be taken at any time during the study to evaluate drug exposure following a safety event

    Pharmacokinetic Sampling Window:
    - Predose: within 60 minutes prior to dosing
    - End of Infusion: within 10 minutes after the end of the infusion
24. ADA: Blood samples (Serum) for ADA will be taken at the below time points and sent to the central laboratory.

- Cycle 1 Day 1: Predose
- Cycle 2 Day 1: Predose
- Predose on Day 1 of Cycles 5, 9, 13 and 17
- Zolbetuximab/placebo 30-Day Safety Follow-up visit
- Zolbetuximab/placebo 90-Day Safety Follow-up visit

ADA Sampling Window: Predose: within 60 minutes prior to dosing

25. Genetic Immune Polymorphism: To be collected per local policy. Whole blood sample taken at C1D1 will be sent to the central laboratory.

Footnotes continued on next page

26. Exploratory Biomarker (Serum and Plasma): To be collected per local policy. Samples should be taken at the below time points and sent to the central laboratory:
    - Cycle 1 Day 1: Predose
    - Cycle 2 Day 1: Predose
    - Cycle 3 Day 1: Predose
    - Cycle 4 Day 1: Predose
    - Cycle 5 Day 1: Predose
    - Cycle 6 Day 1: Predose
    - Cycle 8 Day 1: Predose
    - Zolbetuximab/placebo Study Treatment Discontinuation Visit
27. Optional PGx: For subjects who signed a separate ICF, an optional whole blood sample for PGx for exploratory biomarker analysis may be collected prior to first study drug administration. Sample collection is optional and only collected as allowed per local policy.
28. Optional Post-Progression Tumor Sample: For subjects who signed a separate ICF, an optional post-progression tumor sample for exploratory biomarker analysis may be collected following IRC confirmation of disease progression and prior to initiation of subsequent anticancer therapy. Sample collection is optional and only collected as allowed per local policy.
29. Concomitant medications will be collected from the time of **full main** informed consent through 90 days following the last dose of study treatment.
30. AEs/SAEs: AEs and SAEs (regardless of causality) will be collected from the time of **full main** informed consent through 90 days following the last dose of study treatment. See [Section 5.5.5 Reporting of Serious Adverse Events].

**1 Introduction**
WAS:
Approximately 70% to 80% of patients with metastatic or advanced unresectable gastric and GEJ adenocarcinoma in the first line setting have tumors that are HER2 negative and are not treatable with trastuzumab. These patients have an expected median survival of approximately 1 year [Shah, 2017]. Therefore, a significant unmet medical need exists for the first-line treatment of patients with HER2 negative locally advanced or metastatic unresectable gastric and GEJ cancers. Zolbetuximab is being developed with the goal of addressing this unmet medical need.
IS AMENDED TO:
Approximately 70% to 80% of patients with metastatic or advanced unresectable gastric and GEJ adenocarcinoma in the first line setting have tumors that are HER2 negative and are not treatable with trastuzumab. These patients have an expected median survival of approximately 1 year [Shah **et al**, 2017]. Therefore, a significant unmet medical need exists for the first-line treatment of patients with HER2 negative locally advanced or metastatic unresectable gastric and GEJ cancers. Zolbetuximab is being developed with the goal of addressing this unmet medical need.

**1 Introduction**<br>*<u>1.2.2 Clinical Data</u>*
WAS:
To date, 3 clinical studies have been completed and include GM-IMAB-001 (EudraCT No. 2008 004719-37, referred to as first-in-human [FIM]), PILOT, and GM IMAB 001 02 (EudraCT No. 2009-017365-36) referred to as MONO. Dosing is complete and final data analyses and reporting are ongoing for the FAST study.
IS AMENDED TO:
To date, ~~3~~ **4** clinical studies have been completed and include GM-IMAB-001 (EudraCT No. 2008 004719-37, referred to as first-in-human [FIM]), PILOT, **FAST** and GM IMAB 001 02 (EudraCT No. 2009-017365-36) referred to as MONO. ~~Dosing is complete and final data analyses and reporting are ongoing for the FAST study.~~

**4 Identification of Study Treatment(s)**<br>*4.2 0.9% Sodium Chloride Injection*
ADDED:
**4.2 0.9% Sodium Chloride Injection**<br>**0.9% Sodium Chloride Injection will be used for infusion solution preparation in this study for both zolbetuximab arm and placebo arm. 0.9% Sodium Chloride Injection will not be manufactured or provided to sites by the sponsor. Sites should use their own commercially obtained supply of 0.9% Sodium Chloride Injection. Details of preparation of infusion solution are provided in the Pharmacy Manual and Infusion**

**Guidelines.** ***SPECIFIC TO JAPAN:*** **In this study, 0.9% Sodium Chloride Injection is not considered an 'investigational product' as defined in J-GCP.**<br><br>**For manufacturing, formulation, storage, handling and preparation details please refer to the package insert, SPC or local product information supplied by the manufacturer.**

**4 Identification of Study Treatment(s)**<br>*4.3 Comparative Drug (Placebo)*
WAS:
Placebo will not be manufactured or provided by the sponsor. Sites should use their own commercial supply of 0.9% Sodium Chloride Injection as placebo. Details of preparation of placebo are provided in the Pharmacy Manual and Infusion Guidelines.<br><br>For manufacturing, formulation, storage, handling and preparation details please refer to the package insert, SPC or local product information supplied by the manufacturer.
IS AMENDED TO:
Placebo will not be manufactured or provided by the sponsor. Sites should use their own commercial supply of 0.9% Sodium Chloride Injection as placebo **infusion solution**. Details of preparation of placebo **infusion solution** are provided in the Pharmacy Manual and Infusion Guidelines.<br><br>~~For manufacturing, formulation, storage, handling and preparation details please refer to the package insert, SPC or local product information supplied by the manufacturer.~~

**5 Treatments and Evaluation**<br>*5.1.1.1 Zolbetuximab/Placebo*
WAS:
Subjects will be administered zolbetuximab/placebo as a minimum 2-hour intravenous infusion on day 1 of each cycle. Guidance for slowing the initial infusion to minimize toxicity can be found in the Pharmacy Manual and Infusion Guidelines. Subjects will be administered with a loading dose of 800 mg/m² of zolbetuximab at C1D1 followed by subsequent doses of 600 mg/m² every 3 weeks. Zolbetuximab/placebo must be administered prior to CAPOX. Flow rate data will be collected in the eCRF.<br><br>Please also refer to the dosing schematics for details [Section V].<br><br>It is recommended that zolbetuximab/placebo infusion not exceed 6 hours from start of infusion. Please refer to Pharmacy Manual and Infusion Guidelines for more detailed information, if zolbetuximab/placebo infusion cannot be completed.

IS AMENDED TO:
Subjects will be administered zolbetuximab/placebo as a minimum 2-hour intravenous infusion on day 1 of each cycle. Guidance for slowing the initial infusion to minimize toxicity can be found in the Pharmacy Manual and Infusion Guidelines. Subjects will be administered with a loading dose of 800 mg/m² of zolbetuximab at C1D1 followed by subsequent doses of 600 mg/m² every 3 weeks. Zolbetuximab/placebo ~~must~~ **should** be administered prior to CAPOX. Flow rate data will be collected in the eCRF.<br><br>Please also refer to the dosing schematics for details [Section V].<br><br>~~It is recommended that zolbetuximab/placebo infusion not exceed 6 hours from start of infusion.~~ **Intravenous infusion may be interrupted or slowed down to manage toxicity.** Please refer to Pharmacy Manual and Infusion Guidelines for more detailed information~~, if zolbetuximab/placebo infusion cannot be completed~~.

**5 Treatments and Evaluation**<br>*5.1.1.2 Antiemetics*
WAS:
• All antiemetic premedication should be given at minimum 30 minutes prior to zolbetuximab/placebo treatment.
IS AMENDED TO:
• All antiemetic premedication should be ~~given~~ **initiated** at **a** minimum **of** 30 minutes prior to zolbetuximab/placebo treatment.

**5 Treatments and Evaluation**<br>*5.1.1.3 CAPOX*
WAS:
CAPOX is to be administered after zolbetuximab/placebo infusion.<br>• Oxaliplatin: 130 mg/m2 intravenous infusion on day 1 of each cycle over 2 hours (or longer per institutional standard of care) for up to 8 treatments.
IS AMENDED TO:
CAPOX ~~is to~~ **should** be administered after zolbetuximab/placebo infusion.<br>• Oxaliplatin: 130 mg/m2 intravenous infusion on day 1 of each cycle over 2 hours (or longer per institutional standard of care) for ~~up to~~ **a maximum of** 8 treatments.

**5 Treatments and Evaluation**<br>*5.1.2.2 Zolbetuximab/Placebo Interruption or Permanent Discontinuation*
WAS:
A delay of zolbetuximab/placebo treatment of > 28 days from when the next zolbetuximab/placebo treatment was scheduled to be administered due to unresolved

toxicity associated with zolbetuximab/placebo will result in the subject discontinuing zolbetuximab/placebo.

Radiologic imaging is to be scheduled every 9 weeks (±7 days) counting from C1D1 for the first 54 weeks and then every 12 weeks (±7 days) thereafter; the schedule should be maintained regardless of treatment delay.

Note: Intravenous infusion of zolbetuximab/placebo should be administered as a minimum 2 hour infusion. It is recommended that zolbetuximab/placebo infusion not exceed 6 hours from start of infusion. Please refer to Pharmacy Manual and Infusion Guidelines for more detailed information, if zolbetuximab/placebo infusion cannot be completed.

Guidelines for zolbetuximab/placebo treatment modification due to non-hematologic and hematologic toxicities are described below in Table 2 and Table 3, respectively.

AND

Table 2 Guidelines for Zolbetuximab/Placebo Treatment Modification Due to Non-hematologic Toxicity

Toxicity	Grade 1	Grade 2	Grade 3	Grade 4
**Infusion-related reaction (IRR)** ***other than nausea, vomiting or abdominal pain.*** See Table 4 for further IRR management guidance	Continue Infusion	Interrupt infusion. Infusion may be resumed at a reduced rate when toxicity has improved to grade ≤ 1.	Stop the infusion immediately.<br>Institute appropriate medical management immediately based on the type of reaction.<br>Permanently Discontinue zolbetuximab/placebo	
**Nausea**	Continue Infusion		Interrupt infusion. Hold zolbetuximab/placebo treatment until toxicity improved to grade ≤ 1.<br>If the investigator determines that the toxicity is not related to zolbetuximab and the toxicity improves to grade ≤ 2, then infusion may be restarted at the investigator's discretion.	Not applicable*
**Vomiting**	Continue Infusion	Continue infusion; however, if infusion was held due to grade 3 vomiting, hold infusion until vomiting	Interrupt infusion. Hold zolbetuximab/placebo treatment until toxicity improved to grade ≤ 1.	Permanently Discontinue zolbetuximab/ placebo

		improves to ≤ grade 1.		
**Other Non-hematologic toxicity**	Continue Infusion		Interrupt infusion. Hold zolbetuximab/placebo treatment until toxicity improved to grade ≤ 1.<br>If the investigator determines that the toxicity is not related to zolbetuximab and the toxicity improves to grade ≤ 2, then infusion may be restarted at the investigator's discretion.	Permanently Discontinue zolbetuximab/ placebo
**PRES**	Discontinue zolbetuximab/placebo if PRES is suspected.			

* Grade 4 nausea is not defined in CTCAE v4.03. If investigator assesses nausea as grade 4, manage per local standard of care

IS AMENDED TO:

A delay of zolbetuximab/placebo treatment of > 28 days from when the next zolbetuximab/placebo treatment was scheduled **to begin (> 49 days from when the last dose of zolbetuximab/placebo began)** to be administered due to unresolved toxicity associated with zolbetuximab/placebo will result in the subject discontinuing zolbetuximab/placebo.

Radiologic imaging is to be scheduled every 9 weeks (±7 days) counting from C1D1 for the first 54 weeks and then every 12 weeks (±7 days) thereafter; the schedule should be maintained regardless of treatment delay.

Note: Intravenous infusion of zolbetuximab/placebo should be administered as a minimum 2 hour infusion. ~~It is recommended that zolbetuximab/placebo infusion not exceed 6 hours from start of infusion.~~ **Intravenous infusion may be interrupted or slowed down to manage toxicity.** Please refer to Pharmacy Manual and Infusion Guidelines for more detailed information~~, if zolbetuximab/placebo infusion cannot be completed~~.

Guidelines for zolbetuximab/placebo treatment modification due to non-hematologic and hematologic toxicities**, regardless of investigator assessment of relationship to zolbetuximab/placebo,** are described below in Table 2 and Table 3, respectively.

AND

Table 2 Guidelines for Zolbetuximab/Placebo Treatment Modification Due to Non-hematologic Toxicity

Toxicity	Grade 1	Grade 2	Grade 3	Grade 4
**Infusion-related reaction (IRR)** ***other than nausea,***	Continue Infusion	Interrupt infusion. Infusion may be resumed at a reduced rate	Stop the infusion immediately.<br>Institute appropriate medical management immediately based on the type of reaction.<br>Permanently Discontinue zolbetuximab/placebo	

vomiting or abdominal pain. See Table 4 for further IRR management guidance		when toxicity has improved to grade ≤ 1.		
**Nausea**	Continue Infusion		Interrupt infusion. Hold zolbetuximab/placebo treatment until toxicity **has** improved to grade ≤ 1**, then restart the infusion at a lower rate.**<br>If the investigator determines that the toxicity is not related to zolbetuximab and the toxicity **has** improve~~s~~**d** to grade ≤ 2, then infusion may be restarted at the investigator's discretion **at a lower rate.**	Not applicable*
**Vomiting**	Continue Infusion	Continue infusion; however, if infusion was held due to grade 3 vomiting, hold infusion until vomiting **has** improve~~s~~**d** to ≤ grade 1.	Interrupt infusion. Hold zolbetuximab/placebo treatment until toxicity **has** improved to grade ≤ 1 **and then restart infusion at a lower rate.**	Permanently Discontinue zolbetuximab/ placebo
**Other Non-hematologic toxicity**	Continue Infusion		Interrupt infusion. Hold zolbetuximab/placebo treatment until toxicity **has** improved to grade ≤ 1.<br>If the investigator determines that the toxicity is not related to zolbetuximab and the toxicity **has** improve~~s~~**d** to grade ≤ 2, then infusion may be restarted at the investigator's discretion.#	Permanently Discontinue zolbetuximab/ placebo
**PRES**	Discontinue zolbetuximab/placebo if PRES is suspected.			

* Grade 4 nausea is not defined in CTCAE v4.03. If investigator assesses nausea as grade 4, manage per local standard of care.

# For subjects with a pulmonary embolism, treatment can continue without resolving to grade 2 or less.

5 Treatments and Evaluation

5.1.2.3 Guidelines for Infusion-related Reactions for Zolbetuximab/Placebo

WAS:

Note: Intravenous infusion of zolbetuximab/placebo should be administered as a minimum 2 hour infusion. It is recommended that zolbetuximab/placebo infusion not exceed 6 hours from start of infusion. Please refer to Pharmacy Manual and Infusion Guidelines for more detailed information, if zolbetuximab/placebo infusion cannot be completed.

Table 4 Infusion-related Reactions

**Infusion-Related Reactions**	
Refer to Table 2 for management of infusion related reactions of nausea, vomiting or abdominal pain	
**CTCAE v4.03 Grade**	**Management**
Grade 1 standard infusion reactions ***other than nausea, vomiting or abdominal pain***#	Continue infusion and closely monitor the subject.
Grade 2 standard infusion reaction ***other than nausea, vomiting or abdominal pain***#	Interrupt.<br>Medical management as per type of reaction. Resume infusion once toxicity grade ≤ 1 and reduce the infusion rate for the remaining infusion.<br>For the next infusion:<br>• Increase total infusion time (reduce infusion rate).<br>• Pre-medicate as appropriate.*<br>• Closely monitor the subject for symptoms and signs of an infusion reaction.
Any infusion reaction with features of anaphylaxis<br>**OR**<br>Grade 3 or 4 standard infusion reactions ***other than nausea, vomiting or abdominal pain***#	Stop the infusion immediately.<br>Institute appropriate medical management immediately based on the type of reaction.<br>Permanently Discontinue zolbetuximab/placebo<br>*Once the subject has been stabilized, collect blood for cytokine/chemokine panel and serum total tryptase level (levels typically peak within 3 hours after the onset of symptoms*

CTCAE v4.03: Common Terminology Criteria For Adverse Events
* At the investigators discretion, antihistamines may be used as premedication for the next infusion. Systemic corticosteroids should be avoided or minimized while subject is on study treatment unless required for management of an emergent medical condition (e.g., severe nausea/vomiting or hypersensitivity reaction).
# For grade 3 or 4 IRR of nausea, vomiting or abdominal pain, collect blood for cytokine/chemokine panel and serum total tryptase level.

IS AMENDED TO:

Note: Intravenous infusion of zolbetuximab/placebo should be administered as a minimum 2 hour infusion. ~~It is recommended that zolbetuximab/placebo infusion not exceed 6 hours from start of infusion.~~ **Intravenous infusion may be interrupted or slowed down to manage toxicity.** Please refer to Pharmacy Manual and Infusion Guidelines for more detailed information~~, if zolbetuximab/placebo infusion cannot be completed~~.

Table 4 Infusion-related Reactions

**Infusion-Related Reactions**	
Refer to Table 2 for management of infusion related reactions of nausea, vomiting or abdominal pain	
**CTCAE v4.03 Grade**	**Management**
Grade 1 standard infusion reactions ***other than nausea, vomiting or abdominal pain#***	Continue infusion and closely monitor the subject.
Grade 2 standard infusion reaction ***other than nausea, vomiting or abdominal pain#***	Interrupt.<br>Medical management as per type of reaction. Resume infusion once toxicity grade ≤ 1 and reduce the infusion rate for the remaining infusion.<br>For the next infusion:<br>• Increase total infusion time (reduce infusion rate).<br>• Pre-medicate as appropriate.*<br>• Closely monitor the subject for symptoms and signs of an infusion reaction.
Any infusion reaction with features of anaphylaxis<br>**OR**<br>Grade 3 or 4 standard infusion reactions ***other than nausea, vomiting or abdominal pain#***	Stop the infusion immediately.<br>Institute appropriate medical management immediately based on the type of reaction.<br>Permanently Discontinue zolbetuximab/placebo<br>*Once the subject has been stabilized, collect blood for cytokine/chemokine panel and serum total tryptase level (levels typically peak within 3 hours after the onset of symptoms)* ***and send to the central laboratory***.

CTCAE v4.03: Common Terminology Criteria For Adverse Events
* At the investigators discretion, antihistamines may be used as premedication for the next infusion. Systemic corticosteroids should be avoided or minimized while subject is on study treatment unless required for management of an emergent medical condition (e.g., severe nausea/vomiting or hypersensitivity reaction).
# For grade 3 or 4 IRR of nausea, vomiting or abdominal pain, collect blood for cytokine/chemokine panel and serum total tryptase level **and send to the central laboratory**.

5 Treatments and Evaluation
5.1.2.4 CAPOX Dose Modification

WAS:

The first dose of CAPOX should not be modified. After the assessment of tolerability, dose adjustments should be performed based on investigator judgement utilizing institutional standard of care, approved package insert, SPC, or local product information and/or the recommended criteria in Table 5 based on maximum hematologic or non-hematologic toxicity data from the previous cycle as shown in Table 6 and Table 7, respectively. Dose reduction criteria for oxaliplatin-related neurotoxicity are presented in Table 8. Each drug may be dose reduced independently based on the specific types of toxicities observed. No more than 2 dose reductions will be allowed per drug per subject (see Table 5). Dose re-escalation is not permitted. If further dose reduction is required beyond the criteria in Table 5, that component of CAPOX should be discontinued.

In subjects experiencing toxicity requiring a delay or discontinuation of CAPOX, subject should continue to receive zolbetuximab/placebo as clinically appropriate. If CAPOX is interrupted, subject should be evaluated weekly (at a minimum) until the toxicity has

improved sufficiently at which time treatment can be restarted as described in the tables below (as applicable). A delay of CAPOX treatment for > 28 days from when the next CAPOX treatment was scheduled to be administered due to unresolved toxicity associated with CAPOX will result in the subject discontinuing CAPOX (both components).

IS AMENDED TO:

The first dose of CAPOX should not be modified. After the assessment of tolerability, dose adjustments should be performed based on investigator judgement utilizing institutional standard of care, approved package insert, SPC, or local product information and/or the recommended criteria in Table 5 based on maximum hematologic or non-hematologic toxicity data from the previous cycle as shown in Table 6 and Table 7, respectively. Dose reduction criteria for oxaliplatin-related neurotoxicity are presented in Table 8. Each drug may be dose reduced independently based on the specific types of toxicities observed. **It is recommended that** ~~N~~**n**o more than 2 dose reductions ~~will be allowed~~ per drug per subject **occur** (see Table 5). Dose re-escalation is not ~~permitted~~ **recommended after treatment-related AEs.** If further dose reduction is required beyond the criteria in Table 5, that component of CAPOX should be discontinued.

In subjects experiencing toxicity requiring a delay or discontinuation of CAPOX, subject should continue to receive zolbetuximab/placebo as clinically appropriate. If CAPOX is interrupted, subject should be evaluated weekly (at a minimum) until the toxicity has improved sufficiently at which time treatment can be restarted as described in the tables below (as applicable). A delay of CAPOX treatment for > 28 days from when the next CAPOX treatment was scheduled to **begin (> 49 days from when the last CAPOX dose began)** ~~be administered~~ due to unresolved toxicity associated with CAPOX will result in the subject discontinuing CAPOX (both components).

5 Treatments and Evaluation

5.1.2.5 CAPOX: Dose Modifications for Hematologic Toxicity

WAS:

The CAPOX dose modifications for hematologic toxicity are presented in Table 6. Dose modifications should be maintained until recovery from hematologic toxicity. A delay of CAPOX treatment for > 28 days from when the next CAPOX treatment was scheduled to be administered due to hematologic toxicity associated with CAPOX will result in the subject discontinuing CAPOX (both components).

IS AMENDED TO:

The CAPOX dose modifications for hematologic toxicity are presented in Table 6. Dose modifications should be maintained until recovery from hematologic toxicity. A delay of CAPOX treatment for > 28 days from when the next CAPOX treatment was scheduled to **begin (> 49 days from when the last CAPOX dose began)** ~~be administered~~ due to hematologic toxicity associated with CAPOX will result in the subject discontinuing CAPOX (both components).

5 Treatments and Evaluation
5.1.2.6 CAPOX: Dose Modification for Non-hematologic Toxicity

WAS:

CAPOX dose modifications for non-hematologic toxicity should be based on the most severe toxicity experienced during the last treatment (Table 7). Retreatment should be delayed until recovery of all non-hematologic toxicity to ≤ grade 2 with the exception of increased bilirubin or ALT, which must recover to grade 1 or baseline, whichever was higher. The maximum permitted treatment delay is 28 days from when the next study treatment was scheduled to be administered for recovery of non-hematologic toxicity. If the subject has not recovered sufficiently to meet retreatment criteria within that timeframe, CAPOX should be discontinued.

IS AMENDED TO:

CAPOX dose modifications for non-hematologic toxicity should be based on the most severe toxicity experienced during the last treatment (Table 7). Retreatment should be delayed until recovery of all non-hematologic toxicity to ≤ grade 2 with the exception of increased bilirubin or ALT, which must recover to grade 1 or baseline, whichever was higher. The maximum permitted treatment delay is 28 days from ~~when~~ the next study treatment was scheduled to **begin (49 days from when the last dose of study treatment began)** ~~be administered~~ for recovery of non-hematologic toxicity. If the subject has not recovered sufficiently to meet retreatment criteria within that timeframe, CAPOX should be discontinued.

5 Treatments and Evaluation
5.1.2.8 Oxaliplatin-induced Neurotoxicity

WAS:

Table 8 Oxaliplatin Dose Modification for Associated Neurotoxicity

Toxicity/Duration	Grade 1	Grade 2	Grade 3	Grade 4
**Paresthesia or dysesthesia**	Paresthesia or dysesthesia‡ that does not interfere with function	Paresthesia or dysesthesia‡, interfering with function, but not activities of daily living	Paresthesia or dysesthesia‡ with pain or with functional impairment that also interferes with activities of daily living	Persistent paresthesia or dysesthesia that is disabling or life-threatening
**1 to 7 Days**	No dose reduction	No dose reduction	First Event: Reduce oxaliplatin by 1 dose level at next treatment. Second Event: Reduce oxaliplatin by a second dose level at next treatment.	Discontinue oxaliplatin

**> 7 Days**			Discontinue oxaliplatin	
**Persistent between treatments†**		Reduce oxaliplatin by 1 dose level at next treatment	Discontinue oxaliplatin	
**Acute laryngopharyngeal dysesthesia‡** (during or after the 2-hour infusion)	Discontinue current infusion. At next treatment, increase duration of infusion to 6 hours; may also pretreat with benzodiazepines.			

IS AMENDED TO:

Table 8 Oxaliplatin Dose Modification for Associated Neurotoxicity

Toxicity/Duration	Grade 1	Grade 2	Grade 3	Grade 4
**Paresthesia or dysesthesia**	Paresthesia or dysesthesia‡ that does not interfere with function	Paresthesia or dysesthesia‡, interfering with function, but not activities of daily living	Paresthesia or dysesthesia‡ with pain or with functional impairment that also interferes with activities of daily living	Persistent paresthesia or dysesthesia that is disabling or life-threatening
**1 to 7 Days**	No dose reduction	No dose reduction	First Event: Reduce oxaliplatin by 1 dose level at next treatment. Second Event: Reduce oxaliplatin by a second dose level at next treatment.	Discontinue oxaliplatin
**> 7 Days**			Discontinue oxaliplatin	
**Persistent between treatments†**		Reduce oxaliplatin by 1 dose level at next treatment	Discontinue oxaliplatin	
**Acute laryngopharyngeal dysesthesia‡** (during or after the 2-hour infusion)	Discontinue current infusion. At next treatment, **consider pretreatment with benzodiazepines and** ~~increase~~ **increasing** duration of infusion ~~to 6 hours; may also pretreat with benzodiazepines~~ **as clinically indicated per investigator discretion.**			

5 Treatments and Evaluation

5.1.5 Previous and Concomitant Treatment (Medication and Non-Medication Therapy)

WAS:

All medications and concomitant treatments administered from the time of informed consent through the 90-day safety follow-up visit must be recorded in the eCRF.

Documentation will include the medication name, indication, route and dates of administration.

AND

- Live vaccines should be avoided during the treatment period in which subject is receiving capecitabine and up to 6 months after final capecitabine dose.

AND

The following should be avoided or used with caution and closely monitored during CAPOX administration:

- Cytochrome P450 2C9 substrates (Subjects taking coumarin-derivative anticoagulants concomitantly with capecitabine should have PT/INR monitored regularly and anticoagulant dose adjusted accordingly).
- Anti-epileptic medications (e.g., phenobarbital, phenytoin and primidone)
- Medications known to prolong the QT or QTc interval (refer to https://www.crediblemeds.org for a list of these medications

IS AMENDED TO:

All medications and concomitant treatments administered from the time of **full main** informed consent through the 90-day safety follow-up visit must be recorded in the eCRF. Documentation will include the medication name, indication, route and dates of administration.

AND

- Live vaccines should be avoided during the treatment period in which subject is receiving capecitabine and up to 6 months after final capecitabine dose. **In cases where a live vaccine is needed for COVID-19 prevention, please contact the Medical Monitor for discussion.**

AND

The following should be avoided or used with caution and closely monitored during ~~CAPOX~~ **capecitabine** administration:

- Cytochrome P450 2C9 substrates (Subjects taking coumarin-derivative anticoagulants concomitantly with capecitabine should have PT/INR monitored regularly and anticoagulant dose adjusted accordingly).
- Anti-epileptic medications (e.g., phenobarbital, phenytoin and primidone)

The following should be avoided or used with caution and closely monitored during oxaliplatin administration:

• Medications known to prolong the QT or QTc interval (refer to https://www.crediblemeds.org for a list of these medications

**5 Treatments and Evaluation**<br>*5.2.2 Medical History*
WAS:
Medical history includes all significant medical conditions per the judgement of the investigator that have resolved prior to informed consent or are ongoing at the time of consent.
IS AMENDED TO:
Medical history includes all significant medical conditions per the judgement of the investigator that have resolved prior to informed consent or are ongoing at the time of **full main** consent.

**5 Treatments and Evaluation**<br>*5.4.3 Laboratory Assessments*
WAS:
• From cycle 2 onwards central and local labs may be collected up to 48 hours prior to study treatment.
IS AMENDED TO:
• ~~From cycle 2 onwards c~~**C**entral and local labs may be collected up to 48 hours prior to study treatment.

**5 Treatments and Evaluation**<br>*5.4.6 Performance Status*
WAS:
The ECOG Scale [Oken, 1982] will be used to assess performance status. Refer to [Appendix 12.8].
IS AMENDED TO:
The ECOG Scale [Oken **et al**, 1982] will be used to assess performance status. Refer to [Appendix 12.8].

**5 Treatments and Evaluation**<br>*5.5 Adverse Events and Other Safety Aspects*
WAS:
AE collection will begin from time of informed consent and continue through the 90 days following the last dose of zolbetuximab/placebo and CAPOX (both components).

Serious adverse events (SAEs), regardless of causality will be collected from the time of informed consent through 90 days following the last dose of zolbetuximab/placebo and CAPOX (both components).
IS AMENDED TO:
AE collection will begin from time of **full main** informed consent and continue through the 90 days following the last dose of zolbetuximab/placebo and CAPOX (both components).<br><br>Serious adverse events (SAEs), regardless of causality will be collected from the time of **full main** informed consent through 90 days following the last dose of zolbetuximab/placebo and CAPOX (both components).

**5 Treatments and Evaluation**<br>*5.5.1 Definition of Adverse Events*
WAS:
In order to identify any events that may be associated with study procedures and could lead to a change in the conduct of the study, Astellas collects AEs even if the subject has not received study drug treatment. AE collection begins after the signing of the informed consent and will be collected until 90 days after the last dose of study drug or the subject is determined to be a screen failure.
IS AMENDED TO:
In order to identify any events that may be associated with study procedures and could lead to a change in the conduct of the study, Astellas collects AEs even if the subject has not received study drug treatment. AE collection begins after the signing of the **full main** informed consent and will be collected until 90 days after the last dose of study drug or the subject is determined to be a screen failure.

**5 Treatments and Evaluation**<br>*5.5.5 Reporting of Serious Adverse Events*
WAS:
The collection of AEs and the expedited reporting of SAEs will start following receipt of the signed informed consent and will continue until 90 days after the last dose of study treatment or the subject is determined to be a screen failure.
IS AMENDED TO:
The collection of AEs and the expedited reporting of SAEs will start following receipt of the signed **full main** informed consent and will continue until 90 days after the last dose of study treatment or the subject is determined to be a screen failure.

**5 Treatments and Evaluation**<br>*5.5.8 Adverse Events of Special Interest*
WAS:
In case of zolbetuximab induced nausea, vomiting or hypersensitivity/IRR, infusion rate of zolbetuximab/placebo may be reduced or infusion paused or discontinued based on investigator's clinical judgement about severity of toxicity and local standard of care. See [Section 5.1.2 Study Treatment Dose Modifications, Delays and Interruption].
IS AMENDED TO:
In case of zolbetuximab induced nausea, vomiting or hypersensitivity/IRR, infusion rate of zolbetuximab/placebo may be reduced or infusion paused or discontinued based on investigator's clinical judgement about severity of toxicity and local standard of care. See [Section 5.1.2 Study Treatment Dose Modifications, Delays and Interruption**s**] **and the Pharmacy Manual and Infusion Guidelines.**

**5 Treatments and Evaluation**<br>*5.7.5 Optional Samples for Banked PGx Sample Analysis*
WAS:
5.7.5 Optional Samples for Banked PGx Sample Analysis
IS AMENDED TO:
5.7.5 Optional Samples for Banked ~~PGx~~ **Pharmacogenomics** Sample Analysis

**5 Treatments and Evaluation**<br>*5.7.6 Electronic Clinical Outcome Assessments*
WAS:
Subjects will be asked to complete HRQoL and HRU questionnaires as specified in the Schedule of Assessments [Table 1]. The electronic Clinical Outcomes Assessment (eCOA) questionnaires should be administered during the visit (or up to 48 hours) before any antiemetic or drug treatment and before the disease status is discussed with the subject. For subjects with low literacy or situations where required translation is not available, please contact the sponsor for further guidance. Assessments will be collected at Screening (except for HRU), every 3 weeks, at study treatment discontinuation and 30 and 90 days post zolbetuximab/placebo treatment. HRQoL will be measured by EORTC QLQ-C30, QLQ OG25 plus STO22 Belching subscale, GP and the EQ5D-5L.
IS AMENDED TO:
Subjects will be asked to complete HRQoL and HRU questionnaires as specified in the Schedule of Assessments [Table 1]. The electronic Clinical Outcomes Assessment (eCOA) questionnaires should be ~~administered during the visit~~ **completed by the subject at Screening (except for HRU), day 1 of each cycle** (or up to 48 hours) before any antiemetic or drug treatment and before the disease status is discussed with the subject **using the electronic device provided. When completion by the subject is not possible,**

the questionnaires may be administered to the subject by site personnel using the electronic tablet device. For subjects with low literacy or situations where required translation is not available, please contact the sponsor for further guidance. Assessments will **also** be collected at ~~Screening (except for HRU), every 3 weeks, at~~ study treatment discontinuation and 30 and 90 days post zolbetuximab/placebo treatment. **HRQoL and HRU questionnaires are not required at CAPOX dosing visits (if different from zolbetuximab/placebo dosing visit), CAPOX treatment discontinuation, 30 day safety follow-up and 90 day safety follow-up visits. A combined visit can be completed if zolbetuximab/placebo and both components of CAPOX are discontinued on the same day and HRQoL and HRU questionnaires will be required at those combined visits.** HRQoL will be measured by EORTC QLQ-C30, QLQ OG25 plus STO22 Belching subscale, GP and the EQ5D-5L.

6 Discontinuation

6.1 Discontinuation of Individual Subject(s) From Study Treatment

WAS:

- Subject has a delay of zolbetuximab/placebo and both components CAPOX treatment for > 28 days from when the next zolbetuximab/placebo and both components CAPOX treatment was scheduled to be administered.

IS AMENDED TO:

- Subject has a delay of zolbetuximab/placebo and both components **of** CAPOX treatment for > 28 days from when the next zolbetuximab/placebo and both components **of** CAPOX treatment was scheduled **to begin (> 49 days from when the last dose of zolbetuximab/placebo and both components of CAPOX treatment began)** ~~to be administered~~.

8 Operational Considerations

8.1.1.2 Electronic Clinical Outcomes Assessment

WAS:

eCOA assessments will be performed according to the Schedule of Assessments [Table 1]. Subject HRQoL and HRU questionnaires will be completed by the subject on an electronic tablet device during site visits.

IS AMENDED TO:

eCOA assessments will be performed according to the Schedule of Assessments [Table 1]. Subject HRQoL and HRU questionnaires will be completed by the subject **or, if not possible, administered to the subject by site personnel** on an electronic tablet device ~~during site~~ **for subject** visits.

11 References

WAS:

Ohtsu A, Shah MA, Van Cutsem E, Rha SY, Sawaki A, Park SR, et al. Bevacizumab in combination with chemotherapy as first-line therapy in advanced gastric cancer: a randomized, double-blind, placebo-controlled phase III study. J Clin Oncol. 2011;29:3968-3976.

Pasini F, Fraccon AP, De Manzoni G. The role of chemotherapy in metastatic gastric cancer. Cancer Res. 2011;31:3543-3554.

IS AMENDED TO:

Ohtsu A, Shah MA, Van Cutsem E, Rha SY, Sawaki A, Park SR, et al. Bevacizumab in combination with chemotherapy as first-line therapy in advanced gastric cancer: a randomized, double-blind, placebo-controlled phase III study. J Clin Oncol. 2011;29:3968-3976.

Oken MM, Creech RH, Tormey DC, Horton J, Davis TE, McFadden ET, et al. Toxicity and response criteria of the Eastern Cooperative Oncology Group. Am J Clin Oncol. 1982;5(6):649-55.

Pasini F, Fraccon AP, De Manzoni G. The role of chemotherapy in metastatic gastric cancer. Cancer Res. 2011;31:3543-3554.

Appendix 12.1 Ethical, Regulatory, and Study Oversight Considerations
12.1.4.1 Subject Information and Consent

WAS:

An optional partial screening ICF is available to allow central testing of tissue for CLDN18.2 and HER2 only.

IS AMENDED TO:

An optional partial screening ICF ~~is~~ **may be** available to allow central testing of tissue for CLDN18.2 and HER2 only.

Appendix 12.4 Concomitant Medication Restrictions or Requirements
Prohibited Concomitant Treatment

WAS:

- Live vaccines should be avoided during the treatment period in which subject is receiving capecitabine and up to 6 months after final capecitabine dose.

AND

The following should be avoided or used with caution and closely monitored during CAPOX administration:

- Cytochrome P450 2C9 substrates (Subjects taking coumarin-derivative anticoagulants concomitantly with capecitabine should have PT/INR monitored regularly and anticoagulant dose adjusted accordingly).

• Anti-epileptic medications (e.g., phenobarbital, phenytoin and primidone)<br>• Medications known to prolong the QT or QTc interval (refer to https://www.crediblemeds.org for a list of these medications
IS AMENDED TO:
• Live vaccines should be avoided during the treatment period in which subject is receiving capecitabine and up to 6 months after final capecitabine dose. **In cases where a live vaccine is needed for COVID-19 prevention, please contact the Medical Monitor for discussion.**<br><br>AND<br><br>The following should be avoided or used with caution and closely monitored during ~~CAPOX~~ **capecitabine** administration:<br>• Cytochrome P450 2C9 substrates (Subjects taking coumarin-derivative anticoagulants concomitantly with capecitabine should have PT/INR monitored regularly and anticoagulant dose adjusted accordingly).<br>• Anti-epileptic medications (e.g., phenobarbital, phenytoin and primidone)<br>**The following should be avoided or used with caution and closely monitored during oxaliplatin administration:**<br>• Medications known to prolong the QT or QTc interval (refer to https://www.crediblemeds.org for a list of these medications

**Appendix 12.7 Pharmacogenomic (PGx) Analysis With Banked Samples**
WAS:
Appendix 12.7 Pharmacogenomic (PGx) Analysis With Banked Samples<br><br>Introduction<br><br>The PGx research aims to provide information regarding how naturally occurring differences in a subject’s gene and/or expression of genes based on genetic variation may impact what treatment options are best suited for the subject.
IS AMENDED TO:
Appendix 12.7 Pharmacogenomic ~~(PGx)~~ Analysis With Banked Samples<br><br>Introduction<br><br>The **pharmacogenomic (**PGx**)** research aims to provide information regarding how naturally occurring differences in a subject’s gene and/or expression of genes based on genetic variation may impact what treatment options are best suited for the subject.

Appendix 12.9 Clinical Study Continuity

ADDED:

INTRODUCTION

The purpose of this appendix is to provide acceptable alternate methods to assess safety and efficacy parameters, as appropriate, in the event the clinical study is interrupted at the country, state, site or participant level during any crisis (e.g., natural disaster, pandemic).

BENEFIT-RISK RATIONALE

Maintaining the safety of clinical study participants and delivering continuity of care in the clinical study setting is paramount during any crisis. The site is expected to follow the protocol and associated Schedule of Assessments [Table 1] unless the site PI determines the need to implement the alternate measures. The PI should notify Astellas and/or their CRA when these alternate measures are needed.

The approach outlined within this appendix defines which assessments are required to maintain a favorable benefit/risk to the participant, to maintain overall study integrity and to provide acceptable alternate methods to complete the study required assessments and procedures if study activities are unable to be performed as described in Table 1 due to a crisis.

INFORMED CONSENT

Participants who need to follow any or all of the alternate measures outlined in this Appendix will be required to provide informed consent, which explicitly informs them of the nature of, and rationale for these changes, and gain their agreement to continue participation in the study prior to the implementation of any of these changes. In the event the urgency of implementing the alternate measures does not allow for the participant to provide written consent prior to implementation, the PI or designee will obtain oral agreement from the subject followed by written documentation as soon as is feasible. A separate addendum to the study informed consent will be provided to document the participant's consent of the changes.

PARTICIPANT PROCEDURES ASSESSMENT

Sites with participants who are currently enrolled into this clinical study may consider implementing the alternate methods outlined below if one or more of the following conditions are met due to the crisis:

- **Regional or local travel has been restricted, inclusive of mandatory shelter in place measures, which makes participant travel to/from the study site nearly impossible**

- **Site facilities have been closed for clinical study conduct**
- **Site has been restricted to treating patients with conditions outside of the scope of the study**
- **Site personnel have temporarily relocated the conduct of the study to a location that place a burden on the participant with respect to time and travel**
- **Participant(s) have temporarily relocated from the current study site to an alternate study site to avoid placing a burden on the participant with respect to travel**
- **Participant(s) have temporarily relocated from their home location and the new distances from the site would cause undue burden with respect to time and travel**
- **Participant has risk factors for which traveling to the site poses an additional risk to the participant's health and safety**

Adherence to the original protocol as reflected in the Schedule of Assessments [Table 1] is expected, where plausible, in the case of a crisis. The alternate measures as noted in [Table 10] below are only permissible in the event of a crisis, and after discussing the need with the Astellas Medical Monitor to implement the alternate measures. This is to allow for continuity of receiving investigational medicinal product (IMP) and maintaining critical safety and efficacy assessments for patients participating in the study at a time of crisis.

If one or more of the alternate measures noted below is implemented for a participant, the site should document in the participant's source document the justification for implementing the alternate measure and the actual alternate measures that were implemented, along with the corresponding time point(s).

Table 10 Alternative Schedule of Assessments in Response to a Crisis

Critical Assessment	Alternate Approach(es)	Critical Timepoint										
		Cycles 1-8			Cycle 9+			Study Treatment Discontinuation Visit[18]	30-Day Safety Follow-up Visit(s)[19]	90-Day Safety Follow-up Visit(s)[20]	Post-treatment Follow-up Period [21]	Long-term and Survival Follow-up Periods [22]
**Cycle Day**		1	2-14	15-21	1	2-14	15-21					
**Treatments**												
**Antiemetic Pretreatment[5]**	Oral antiemetics can be administered at home as per SoC	X			X							
**Zolbetuximab/Placebo[6]**	Window of -2 days acceptable	X			X							
**Post-infusion Observation Period[7]**	Decrease of initial observation period to 1 hour and subsequent observation period to 30 min is acceptable if there are no AEs of ≥ grade 2	X			X							
Table continued on next page												
**Oxaliplatin** } **CAPOX[8]**	If previous cycle Day 1 zolbetuximab/placebo administration was conducted at the study site, the following cycle can be administered (oxaliplatin) and dispensed (capecitabine) locally per SoC only (not zolbetuximab/placebo administration) by oncology qualified personnel, and if dosing records can be obtained from the treating facility. The next cycle then must be administered at the study site	X										

**Capecitabine**	**for zolbetuximab/placebo administration. At least every other cycle must be conducted at the study site for zolbetuximab/placebo and oxaliplatin administration and capecitabine dispensing.**	**X**	**X**		**X**	**X**						
Table continued on next page												
**Safety Assessments**												
**Physical Examination**[9]	**Targeted exam is allowed after C1D1. Physical exam not completed at Study Treatment Discontinuation Visit and 30-Day Safety Follow-up Visit acceptable if no active AE.**	**X**			**X**							
**Weight**[9]	**If there are no associated active AEs, it is acceptable if weight is not done at Study Treatment Discontinuation Visit and 30-Day Safety Follow-up Visit.**	**X**			**X**							
**Vital Signs**[10]	**If a cycle is administered at a local facility for SoC regimen administration, SoC can be applied. Missed assessments at Study Treatment Discontinuation Visit and 30-Day Safety Follow-up visit acceptable if there are no associated active AEs. Vital sign frequency during post observation period can be decreased.**	**X**			**X**							
**ECOG Performance Status**[9]	**Not required at Study Treatment Discontinuation visit; ECOG Performance Status may be assessed and captured via phone contact.**	**X**			**X**							
Table continued on next page												

**12-lead ECG[11]**	**ECGs allowed up to 4 days prior to treatment visits after C1D1 and can be done locally but must be reviewed prior to dosing; if dosing visits are conducted at local facility, SoC ECG monitoring would be acceptable; if capecitabine only is dispensed and ECG is not able to be performed, this is acceptable; Study Treatment Discontinuation Visit and 30-Day Safety Follow-up Visit required only if clinically indicated.**	**X**			**If clinically indicated and/or per local requirements**							
Table continued on next page												
**Image Assessment[12]**	**At select visits, efficacy assessment using radiological examinations are required. Independent central reading of locally obtained scans can be facilitated by sharing of Image Acquisition Guidelines from the study site to local site, if applicable. Imaging assessment can be done locally but must be available for submission to central imaging vendor. Investigational site will be requested to re-read the scan performed at local site. If investigational site read is not an option, the investigator should discuss the case with the local institution radiologist. The local site imaging report is required.**	**Every 9 weeks ±7 days from C1D1 for the first 54 weeks and then every 12 weeks ±7 days thereafter**										
**Subject Contact**	**Long Term Survival and Safety follow-up visits can be conducted via phone.**											**X**
Table continued on next page												
**Laboratory Tests**												
**Biochemistry[13]**	**Sample may be collected up to 4 days prior to treatment visit; collection of samples at local facility acceptable if results can be made available to investigative site.**	**X**			**X**			**X**	**X**			
**TSH and Free T4[13]**	**If this testing is unable to be performed, this is acceptable**	**If clinically indicated**										

**Hematology[13]**	**Sample may be collected up to 4 days prior to treatment visit; collection of samples at local facility acceptable if results can be made available to investigative site.**	**X**			**X**			**X**	**X**			
**Coagulation Parameters (PT, PTT and INR)[14]**	**None as protocol allows SoC if clinically indicated. If the subject is not on a concomitant medication that affects these parameters, it is acceptable if these are not done.**	**If clinically indicated**										
**Urinalysis[13]**	**Sample may be collected up to 4 days prior to treatment visit; collection at local facility also allowed.**	**X**			**X**			**X**	**X**			
**Serum Pregnancy Test[15]**	**Collection at local facility also allowed.**	**If clinically indicated and/or per local requirements**										
Table continued on next page												
**Urine Pregnancy Test[16]**	**Sample may be collected up to 4 days prior to treatment visit; collection at local facility also allowed if results can be made available to investigative site.**	**X**			**X**			**X**	**X**			
**Sampling**												
**Pharmacokinetics of Zolbetuximab (Serum)[23]**	**Samples at predose/End of Infusion will be collected if subject receives treatment at investigative site. If central lab cannot receive samples, samples can be stored at sites in -70°C freezer until shipping is accepted again. Follow-up samples will be collected if subject visits the investigative site.**	**X**			**X**				**X**	**X**		
**Antidrug-Antibodies (ADA) for Immunogenicity[24]**	**If subject is dosed or visits investigative site, ADA samples should be collected. Samples cannot be collected at local facility. Sample collection prioritized if clinically indicated. If central lab cannot receive samples, samples can be stored at sites in -70°C freezer until shipping is accepted again.**	**X**			**X**				**X**	**X**		
Table continued on next page												

**Genetic Immune Polymorphisms (Whole Blood)[25]**	**In general, biomarker samples are collected on zolbetuximab treatment days and do not require a unique visit to the study site. If samples can be collected but central labs cannot receive samples, samples can be stored at sites until shipping is accepted again.**	**X**										
**Exploratory Biomarkers (Serum)[26]**	**In general, biomarker samples are collected on zolbetuximab treatment days and do not require a unique visit to the study site. If samples can be collected but central labs cannot receive samples, samples can be stored at sites until shipping is accepted again.**	**X**						**X**				
**Exploratory Biomarkers (Plasma)[26]**	**In general, biomarker samples are collected on zolbetuximab treatment days and do not require a unique visit to the study site. If samples can be collected but central labs cannot receive samples, samples can be stored at sites until shipping is accepted again.**	**X**						**X**				
Table continued on next page												
**Whole Blood Sample for PGx (optional)[27]**	**In general, biomarker samples are collected on zolbetuximab treatment days and do not require a unique visit to the study site. If samples can be collected but central labs cannot receive samples, samples can be stored at sites until shipping is accepted again.**	**X**										
**Post-progression Tumor Sample (optional)[28]**	**In general, biomarker samples are collected on zolbetuximab treatment days and do not require a unique visit to the study site. If samples can be collected but central labs cannot receive samples, samples can be stored at sites until shipping is accepted again.**							**X**				
**Concomitant Medication[29]**	**Remote/Virtual/Telemedicine "Visits" allowed for non-dosing visits. Please refer to protocol schedule of assessments.**	**X**			**X**			**X**	**X**	**X**		

**AE/SAE[30]**	**Remote/Virtual/Telemedicine "Visits" allowed for non-dosing visits. Please refer to protocol schedule of assessments.**	**X**			**X**			**X**	**X**	**X**		

ADA: antidrug antibody; AE: adverse event; βhCG: beta human chorionic gonadotropin; C1D1: Cycle 1 Day 1; CAPOX: capecitabine and oxaliplatin; CLDN: claudin; CT: computerized tomography; DPD: dihydropyrimidine dehydrogenase; eCOA: electronic Clinical Outcomes Assessment; ECG: electrocardiogram; ECOG: Eastern Cooperative Oncology Group; eCRF: electronic case report form; FFPE: formalin fixed paraffin embedded; HER2: human epidermal growth factor receptor 2; HRQoL: health-related quality of life; HRU: Health Resource Utilization; ICF: informed consent form: INR: international normalized ratio; IRC: independent review committee; IRR: infusion-related reaction; IRT: interactive response technology; IV: intravenous; MRI: magnetic resonance imaging; OS: overall survival; PD: progressive disease; PFS: progression free survival; PFS2: progression free survival following subsequent anticancer treatment; PGx: pharmacogenomics; PT: prothrombin time; PTT: partial thromboplastin time; RECIST: Response Evaluation Criteria In Solid Tumors; SAE: serious adverse event; SoC: standard of care; T4: thyroxine; TSH: thyroid stimulating hormone

* **+7 calendar day visit window does not apply to C1D1.**

1. **Screening: The Screening period is 45 days from full main ICF signature. Retesting of lab values is allowed within the 45-day Screening period. Re-screening outside the 45-day window under a new subject number may be allowed once and upon discussion with the medical monitor.**
   - **Optional partial screening: A partial screening ICF may be available for central testing of tissue for CLDN18.2 and HER2 only.**
   - **Laboratory testing:**
     - **Eligibility can be determined based on central and/or local laboratory testing; however:**
       - **The most recent laboratory data must be used to confirm the subject's eligibility.**
       - **Central labs must be collected and submitted to the central laboratory during the Screening period.**
       - **If retesting of lab values is necessary to confirm eligibility, local labs can be used without requiring additional sample collection for central laboratory submission.**
     - **The screening labs used to determine eligibility should be collected within 14 days prior to randomization.**
   - **Radiologic imaging used to confirm eligibility must be conducted within 28 days prior to randomization.**
2. **CLDN18.2 and HER2 Testing: FFPE tumor tissue will be collected for central testing to determine CLDN18.2 and HER2 status. Archival tumor tissue from the primary tumor (gastric or GEJ) is preferred. If primary tumor tissue is not available, tumor tissue from a metastatic site (excluding bone metastasis) may be used. A minimum of 1 FFPE tumor tissue block (preferred) OR a minimum of 15 FFPE unstained slides are required as allowed per local policy. If slides are submitted, the slides should be freshly cut from the FFPE block within the time frame described in the laboratory manual. If local HER2 results are already available from local testing, a minimum of 12 FFPE unstained slides are required to be submitted to the central lab as allowed per local policy. If the specimen is insufficient or unavailable, a biopsy may be performed to obtain primary tumor tissue (preferred) or tumor tissue from metastatic site (excluding bone metastasis). Sponsor pre-approval is required when the sole purpose of the biopsy procedure is to assess eligibility for this study. If the required number of slides cannot be provided, the sponsor or designee should be contacted for further guidance. See [Section 5.7.3 Tumor Tissue Samples].**
3. **Confirmation of Inclusion/Exclusion Criteria must be completed prior to randomization.**
4. **Randomization: After confirmation of eligibility, the blinded site user will perform the randomization IRT transaction. The unblinded pharmacist/designee will be notified by the IRT system about the randomly assigned treatment. Randomization may be performed prior to C1D1. If C1D1 cannot be performed within 5**

calendar days from Randomization please contact the Medical Monitor for discussion. Details of infusion preparation and storage requirements are defined in the Pharmacy Manual and Infusion Guidelines.

Footnotes continued on next page

5. **Antiemetic Pretreatment: Prophylactic antiemetics should be given according to institutional standard of care, published guidelines and the respective product package insert(s). All antiemetic premedication should be initiated at a minimum of 30 minutes prior to treatment. For further details, see [Section 5.1.1.2 Antiemetics].**
6. **Zolbetuximab/placebo will be administered as a minimum 2-hour intravenous infusion every 3 weeks starting on C1D1. Please refer to Pharmacy Manual and Infusion Guidelines for more detailed information. Zolbetuximab/placebo should be administered prior to CAPOX. For further details, see [Section 5.1.1.1 Zolbetuximab/Placebo].**
7. **Post-infusion Observation Period: Following the first dose of zolbetuximab/placebo on C1D1, the subject must be observed for 2 hours post-zolbetuximab/placebo infusion. The post-infusion observation period can be conducted during the CAPOX administration. If any ≥ grade 2 AEs are observed during infusion or during the post-infusion observation period, subsequent zolbetuximab/placebo infusion times should be extended and subjects should continue to be observed for 2 hours post zolbetuximab/placebo infusion. If the subject does not develop any grade ≥ 2 AEs, the subject should be observed for 1 hour post-infusion for their subsequent zolbetuximab/placebo infusions. The subject should be instructed to notify site personnel if they develop any AEs during this observation time period. In the event of an IRR with features of anaphylaxis (regardless of grade) or grade 3 or 4 IRR, blood samples for cytokine/chemokine panel and serum total tryptase level (levels typically peak within 3 hours after the onset of symptoms) should be collected once the subject has stabilized, for shipment to the central laboratory. See Observation Period following zolbetuximab/placebo infusion [Section 5.4.2] for further details.**
8. **CAPOX is a combination of oxaliplatin intravenous infusion and capecitabine tablets and will be administered starting at C1D1 for up to 8 treatments. See [Section 5.1.1.3].**
9. **Physical Exam: should include height (at Screening only), weight and ECOG performance status. A full physical exam is required at Screening. The physical exam only needs to be repeated on C1D1 if clinically significant changes from screening are observed (in the opinion of the investigator). Targeted (symptom driven) physical exams should be conducted every 3 weeks on day 1 of each cycle. For further details, see [Section 5.4.4 Physical Examination]**
10. **Vital signs (pulse, blood pressure, temperature) should be taken during every visit at the following time points (see [Section 5.4.1 Vital Signs]):**
    - **Predose at every visit**
    - **C1D1: Every 30 (±10) minutes during zolbetuximab/placebo infusion**
    - **Subsequent zolbetuximab/placebo infusions: every 60 (±10) minutes during zolbetuximab/placebo infusions if the subject did not develop any ≥ grade 2 AEs during the C1D1 zolbetuximab/placebo infusion or Post-infusion Observation Period.**
    - **Every 60 (±10) minutes post zolbetuximab/placebo infusion during the Post-infusion Observation Period (for 1 or 2 hours. See footnote 7)**
    - **Unscheduled if clinically indicated**

Footnotes continued on next page

11. **ECGs: ECGs will be locally assessed. When collected on the same day, ECG should be collected prior to pharmacokinetic samples. For further details, see [Section 5.4.5 Electrocardiogram]. A single ECG will be performed at the following time points:**
    - **Screening**
    - **Up to 48 hours prior to every oxaliplatin infusion (before any antiemetic treatment administration)**
    - **Up to 6 hours following completion of every oxaliplatin infusion**
    - **Zolbetuximab/placebo Discontinuation Visit**
    - **Zolbetuximab/placebo 30-day Follow-up Visit**
    - **If clinically indicated or per local requirements**
12. **Imaging Assessments: Radiologic imaging will be evaluated at Screening (must be conducted within 28 days prior to randomization) and every 9 weeks (± 7 days) counting from C1D1 for the first 54 weeks and then every 12 weeks (± 7 days) thereafter until subject develops radiological disease progression per RECIST 1.1 by IRC or starts other systemic anticancer treatment, whichever comes earlier. Imaging schedule should be maintained regardless of treatment delay. Imaging will include CT scans with contrast of the thorax, abdomen, and pelvis. If CT scan with contrast is medically not feasible, MRI may be used for imaging. Bone scans (or focal X-ray) or brain imaging should be performed if metastatic disease in bone or brain is suspected, respectively. The same mode of imaging should be utilized throughout the study unless medical necessity requires a change. For randomized subjects, screening imaging should be sent to the central imaging vendor no later than at the time of submission of the first on-treatment imaging. All imaging acquired post randomization will be sent to the central imaging vendor within 7 days of scanning for the blinded independent central assessment of radiological tumor response based on RECIST 1.1. The investigator should make every effort to immediately submit radiologic assessments for IRC review when PD is suspected. See [Section 5.3 Efficacy Assessments]. Refer to Imaging Acquisition Guidelines for further detail on scan modality and contrast options.**
13. **Laboratory Assessments: See [Section 5.4.3 Laboratory Assessments] for list of laboratory assessments. Laboratory tests must be sent to the central laboratory for analysis. For screening/eligibility laboratory assessments, see footnote number 1.**
    - **Laboratory test results (central or local) will be reviewed by the investigator prior to any study treatment. Clinical significance of out-of-range laboratory findings is to be determined and documented by the investigator/sub-investigator who is a qualified physician.**
    - **Local laboratory results may be used for treatment decisions; however, central laboratory samples must also be drawn per protocol and sent to the central laboratory.**
    - **Central and local labs may be collected up to 48 hours prior to study treatment.**
    - **Holidays and weekends should be taken into account when scheduling these blood draws.**
    - **Additional assessments may be done centrally or locally to monitor AEs or as clinically indicated.**
14. **Coagulation (PT, PTT and INR): Coagulation tests should be done at Screening and during study treatment period if clinically indicated. Local or central lab results may be used to confirm eligibility. Ongoing evaluation should be continued for subjects who are receiving therapeutic anticoagulation according to local standard of care. See [Section 5.4.3 Laboratory Assessments].**
15. **Serum Pregnancy Test: Serum pregnancy tests will be collected for female subjects of childbearing potential only. Serum pregnancy tests collected at Screening, during study treatment period and if clinically indicated or per local requirements. (Note: For Screening, subjects with elevated serum βHCG and a demonstrated non-pregnant status through additional testing are eligible.) Local or central laboratory results may be used to confirm eligibility.**

Footnotes continued on next page

16. **<u>Urine Pregnancy Test</u>: Urine pregnancy tests will be collected for female subjects of childbearing potential only. Local urine pregnancy tests to be performed during the treatment period every 3 weeks on day 1 of each cycle and at the zolbetuximab/placebo Study Treatment Discontinuation and 30-day Safety Follow-up Visits. Additional urine pregnancy testing for 6 months after the final study treatment administration may be conducted based on local requirements.**
17. **<u>HRQoL and HRU questionnaires</u>: eCOA questionnaires should be completed by the subject at Screening (except for HRU), on day 1 of each cycle (or up to 48 hours) before any antiemetic or drug treatment and before the disease status is discussed with the subject using the electronic tablet device provided. When completion by the subject is not possible, the questionnaires may be administered to the subject by site personnel using the electronic tablet device. For subjects with low literacy or situations where required translation is not available, please contact the sponsor for further guidance.**
18. **<u>Study Treatment Discontinuation Visit (End of Study Treatment)</u>: The Study Treatment Discontinuation Visit will take place ≤ 7 days following the decision to discontinue study treatment (zolbetuximab/placebo and CAPOX [both components]). If zolbetuximab/placebo and CAPOX (both components) are discontinued on a different day, subjects will have separate Study Treatment Discontinuation Visits following each treatment's discontinuation. Laboratory tests must be sent to the central laboratory for analysis. HRQoL and HRU questionnaires are not required at CAPOX treatment discontinuation visit. A combined visit can be completed if zolbetuximab/placebo are discontinued on the same day.**
19. **<u>30-day Safety Follow-up Visit</u>: A 30-day Safety Follow-up Visit should occur 30 days after the last dose of zolbetuximab/placebo and will include the assessments as shown in the Schedule of Assessments above. A 30-day Safety Follow-up Visit should occur 30 days after the last dose of CAPOX (both components) and may be conducted by phone if the subject is unable to visit the site and will require contact for AE/SAE collection only. HRQoL and HRU questionnaires are not required at CAPOX 30-day safety follow up visit. A combined visit can be completed if zolbetuximab/placebo and both components of CAPOX are discontinued on the same day and HRQoL and HRU questionnaires should be completed for a combined visit.**
20. **<u>90-day Safety Follow-up Visits</u>: A 90-day Safety Follow-up Visit should occur 90 days after the last dose of zolbetuximab/placebo and will include the assessments as shown in the Schedule of Assessments above. A 90-day Safety Follow-up Visit should occur 90 days after the last dose of CAPOX (both components) and may be conducted by phone if the subject is unable to visit the site and will require contact for AE/SAE collection only. HRQoL and HRU questionnaires are not required at CAPOX 90-day safety follow up visit. A combined visit can be completed if zolbetuximab/placebo and both components of CAPOX are discontinued on the same day and HRQoL and HRU questionnaires should be completed for a combined visit.**
21. **<u>Post-treatment Follow-up</u>: if a subject discontinues all study treatments (zolbetuximab/placebo and both components of CAPOX) prior to IRC-confirmed radiological disease progression, the subject will enter the Post-treatment Follow-up Period and continue to undergo imaging assessments every 9 weeks (±7 days) (or every 12 weeks [±7 days] if subject has been on study over 54 weeks) until radiologic disease progression (i.e., PFS) or the subject starts subsequent anticancer treatment, whichever occurs earlier. If study treatments (zolbetuximab/placebo and both components of CAPOX) are discontinued due to PD, the subject will enter the Long-term and Survival Follow-up Period.**
22. **<u>Long-term and Survival Follow-up Period</u>: Following disease progression on first-line treatment or start of subsequent anticancer treatment, subjects will be followed in the Long-term and Survival Follow-up Period per institutional guidelines, but not less than every 12 weeks. Radiologic imaging will be done per standard of care and read locally until PFS2 is documented. Survival Follow-up Period will continue until death (from any cause). All post-progression details including subsequent anticancer treatment and date and site of progression will be recorded on the eCRF. Subject contact by phone or other remote methods is sufficient during Long-term and Survival Follow-up.**

Footnotes continued on next page

23. <u>Pharmacokinetics</u>: Serum samples for zolbetuximab/placebo will be taken at the below time points and sent to the central laboratory. The date and time of each blood sample collection will be recorded to the nearest minute.

- **Cycle 1 Day 1: End of zolbetuximab/placebo infusion**
- **Cycle 2 Day 1: Predose**
- **Cycle 3 Day 1: End of zolbetuximab/placebo infusion**
- **Predose on Day 1 of Cycles 5, 9, 13 and 17**
- **Zolbetuximab/placebo 30-Day Safety Follow-up visit**
- **Zolbetuximab/placebo 90-Day Safety Follow-up visit**
- **Unscheduled pharmacokinetic blood samples may be taken at any time during the study to evaluate drug exposure following a safety event**

Pharmacokinetic Sampling Window:

- **Predose: within 60 minutes <u>prior</u> to dosing**
- **End of Infusion: within 10 minutes <u>after</u> the end of the infusion**

24. <u>ADA</u>: Blood samples (Serum) for ADA will be taken at the below time points and sent to the central laboratory.

- **Cycle 1 Day 1: Predose**
- **Cycle 2 Day 1: Predose**
- **Predose on Day 1 of Cycles 5, 9, 13 and 17**
- **Zolbetuximab/placebo 30-Day Safety Follow-up visit**
- **Zolbetuximab/placebo 90-Day Safety Follow-up visit**

ADA Sampling Window: Predose: within 60 minutes <u>prior</u> to dosing

25. <u>Genetic Immune Polymorphism</u>: To be collected per local policy. Whole blood sample taken at C1D1 will be sent to the central laboratory.

26. <u>Exploratory Biomarker</u> (Serum and Plasma): To be collected per local policy. Samples should be taken at the below time points and sent to the central laboratory:

- **Cycle 1 Day 1: Predose**
- **Cycle 2 Day 1: Predose**
- **Cycle 3 Day 1: Predose**
- **Cycle 4 Day 1: Predose**
- **Cycle 5 Day 1: Predose**
- **Cycle 6 Day 1: Predose**
- **Cycle 8 Day 1: Predose**
- **Zolbetuximab/placebo Study Treatment Discontinuation Visit**

27. <u>Optional PGx</u>: For subjects who signed a separate ICF, an optional whole blood sample for PGx for exploratory biomarker analysis may be collected prior to first study drug administration. Sample collection is optional and only collected as allowed per local policy.

28. <u>Optional Post-Progression Tumor Sample</u>: For subjects who signed a separate ICF, an optional post-progression tumor sample for exploratory biomarker analysis may be collected following IRC confirmation of disease progression and prior to initiation of subsequent anticancer therapy. Sample collection is optional and only collected as allowed per local policy.

Footnotes continued on next page

29. Concomitant medications will be collected from the time of full main informed consent through 90 days following the last dose of study treatment.

30. <u>AEs/SAEs</u>: AEs and SAEs (regardless of causality) will be collected from the time of full main informed consent through 90 days following the last dose of study treatment. See [Section 5.5.5 Reporting of Serious Adverse Events].

IMP SUPPLY

If any of the conditions outlined above in the Participants Procedures Assessment are met, one or all of the following mitigating strategies will be employed, as needed, to ensure continuity of IMP supply to the participants:

- **Increase stock of IMP on site to reduce number of shipments required, if site space will allow, as cold storage space is needed.**

DATA COLLECTION REQUIREMENTS

Additional data may be collected in order to indicate how participation in the study may have been affected by a crisis and to accommodate data collection resulting from alternate measures implemented to manage the conduct of the study and participant safety.

- **Critical assessments for safety and efficacy based on study endpoints to be identified as missing or altered (performed virtually, at alternative locations, out of window, or other modifications) due to the crisis.**

14 COORDINATING INVESTIGATOR'S SIGNATURE

A Phase 3, Global, Multi-Center, Double-Blind, Randomized, Efficacy Study of Zolbetuximab (IMAB362) Plus CAPOX Compared with Placebo Plus CAPOX as First-line Treatment of Subjects with Claudin (CLDN)18.2-Positive, HER2-Negative, Locally Advanced Unresectable or Metastatic Gastric or Gastroesophageal Junction (GEJ) Adenocarcinoma

ISN/Protocol 8951-CL-0302

Version 3.1 Incorporating Nonsubstantial Amendment 1

06 Jan 2021

I have read all pages of this clinical study protocol for which Astellas is the sponsor. I agree that it contains all the information required to conduct this study.

Coordinating Investigator:

Signature: ______________________________ ______________________
<Insert name, department/affiliation, name of institution> Date (DD Mmm YYYY)

Printed Name: ____

Address: ____

____

15 SPONSOR'S SIGNATURES

Astellas Signatories

(Electronic signatures are attached at the end of the document)

PPD	Development Medical Science - Oncology
PPD	Biostatistics

13 ATTACHMENT 1: COUNTRY-SPECIFIC SUBSTANTIAL AMENDMENT 3 FOR CHINA

I. The purpose of this amendment is:

**Substantial Changes**
**1. Update Inclusion Criterion #14**
DESCRIPTION OF CHANGE:
Inclusion criterion #14 is updated to specify that the subject has a known human epidermal growth factor reception 2 (HER2)-negative gastric or gastroesophageal junction tumor.
RATIONALE:
Investigators in China will rely on known HER2 status to determine eligibility, as HER2 is routinely tested as standard of care in advanced gastric cancer in China.
**2. Update CLDN18.2 Slide Requirement and Removal of Central Analysis of HER2 Tumor Samples**
DESCRIPTION OF CHANGE:
The tissue requirements have been reduced to 5 formalin-fixed paraffin embedded (FFPE) unstained slides. The option to submit FFPE tumor tissue blocks has been removed. The option for central HER2 testing has been removed.
RATIONALE:
In order to reduce the burden to subjects in China, the tissue requirements have been limited to the 5 slides that is the minimum number needed to centrally test for the CLDN18.2 biomarker to determine eligibility. The reduction in the required number of FFPE slides is a result of the removal of central exploratory and HER2 testing. Investigators in China will rely on known HER2 status to determine study eligibility as HER2 is routinely tested as standard of care in advanced gastric cancer in China.<br>The removal of submission of a FFPE tumor tissue block to the central laboratory reflects the site's local requirement restricting tissue blocks from being sent outside the hospital setting.
**3. Remove Collection and Analysis of Exploratory Biomarker Samples, Optional Pharmacogenomics (PGx) and Post Progression Tumor Samples**
DESCRIPTION OF CHANGE:
Collection and analyses of exploratory biomarker samples, optional PGx and post-progression tumor samples are removed from the protocol. The protocol has been updated to remove reference to the testing and analysis of these samples. A note is added to the exploratory objectives/endpoints to specify that genomic and exploratory biomarker testing will not be performed on samples from subjects in China.

RATIONALE:
In order to reduce the burden to subjects in China, exploratory biomarker samples, optional PGx and post-progression tumor samples testing have been removed. The removal reduces the number of slides and blood samples required for central laboratory submission.

**Non-Substantial Changes**
**1. Add China Specific Information**
DESCRIPTION OF CHANGE:
Text is added to specify that approximately 50 of the 175 global study centers will be located in China, and approximately 250 of the 550 total subjects will be in China.
RATIONALE:
The amount of subjects and institutional sites in China is identified for the China specific amendment.
**2. Update to Sponsor Pre-approval for Biopsy to Meet Eligibility**
DESCRIPTION OF CHANGE:
Additionally, sponsor pre-approval is requested, not required, when a biopsy will be done for the sole purpose of assessing study eligibility.
RATIONALE:
Updated for China, as the requirement for sponsor pre-approval will not be mandatory in China.
**3. Change in Requirement for Central Laboratory Testing**
DESCRIPTION OF CHANGE:
For treatment visits, the request for sponsor approval for use of local laboratory testing only was added.
RATIONALE:
During treatment visits, the protocol has been updated to allow flexibility for local laboratory analysis, with prior approval by sponsor, to address those sites and that will not allow central laboratory analyses.
**4. Update Total Amount of Blood**
DESCRIPTION OF CHANGE:
The maximum amount of blood collected during the study is decreased to 10 mL at screening and less at later visits.

RATIONALE:
The removal of exploratory analyses in blood samples subsequently reduced the amount of blood to be collected for subjects in China.
**5. Minor Administrative-type Changes**
DESCRIPTION OF CHANGE:
Include minor administrative-type changes (e.g., typos, format, numbering and consistency throughout the protocol). Remove *or provided* in Section 4.2, Comparative Drug (Placebo). Change the word *must* to *should* as permitted in Sections 8.1.1, Data Collection and 12.1.5, Source Documents.
RATIONALE:
To provide clarifications to the protocol and to ensure complete understanding of study procedures.

II. Amendment Summary of Changes:

IIA. Substantial Changes

**IV Synopsis, Study Design Overview**
WAS:
Biomarkers and Other Sampling<br>Samples for pharmacokinetics, immunogenicity and biomarkers, as well as FFPE tumor tissue specimens (for eligibility) will be collected. Optional pharmacogenomics and post-progression tumor samples may be collected for those subjects who sign a separate ICF.
IS AMENDED TO:
~~*Biomarkers and*~~ *Other Sampling*<br>Samples for pharmacokinetics~~,~~ **and** immunogenicity ~~and biomarkers, as well as FFPE tumor tissue specimens (for eligibility)~~ will be collected. ~~Optional pharmacogenomics and post-progression tumor samples may be collected for those subjects who sign a separate ICF.~~

V Flow Chart, Study Schematics, and Schedule of Assessments

Table 1 Schedule of Assessments

WAS:

VISIT	Screening[1]	Study Treatment Period (Each Cycle = approximately 42 Days)								Follow-up Period				
		Cycles 1 to 4<br>Zolbetuximab/Placebo<br>+<br>CAPOX				Cycle 5+<br>Zolbetuximab/Placebo<br>+<br>5FU + Folinic Acid<br>(at investigator discretion)				Study Treatment Discontinuation Visit[18]	30-Day Safety Follow-up Visit(s)[19]	90-Day Safety Follow-up Visit(s)[20]	Post Treatment Follow-up Period[21]	Long Term and Survival Follow-up Periods[22]
Day		1[5]	15	22	29	1	15	22	29					
Visit Window (calendar days)	-45 to -1	+ 7*	+ 7	+ 7	+ 7	+ 7	+ 7	+ 7	+ 7	+ 7	+ 7	± 7	± 7	± 14
HER2 Tumor Sample[2]	X													
Sampling														
Genetic Immune Polymorphisms (Whole Blood)[25]		X												
Exploratory Biomarkers (Serum)[26]		X		X						X				
Exploratory Biomarkers (Plasma)[26]		X		X						X				
Whole Blood Sample for PGx (optional)[27]		X												
Post-Progression Tumor Sample (optional)[28]										X				
Concomitant Medication[29]	X	X	X	X	X	X	X	X	X	X	X	X		
AEs/SAEs[30]	X	X	X	X	X	X	X	X	X	X	X	X		

HER2: human epidermal growth factor reception 2; PGx: pharmacogenomics

1. Screening: The Screening period is 45 days from ICF signature. Re-testing of lab values is allowed within the 45-day screening period. Re-screening outside of the 45-day window under a new subject number may be allowed upon discussion with the Medical Monitor.
   - Optional Partial Screening: A partial screening ICF is available for central testing of tissue for CLDN18.2 and HER2 only.
   - Laboratory testing:

     Eligibility can be determined based on central and/or local testing, **however**:
     - The most recent laboratory data must be used to confirm the subject's eligibility.
     - Central labs must be collected and submitted to the central laboratory during the Screening period.
     - If retesting of lab values is necessary to confirm eligibility, local labs can be used without requiring additional sample collection for central laboratory submission.
     - The screening labs used to determine eligibility should be collected within 14 days prior to randomization.
   - Radiologic imaging used to confirm eligibility must be conducted within 28 days prior to randomization.
2. CLDN18.2 and HER2 Testing: FFPE tumor tissue will be collected for central testing to determine CLDN18.2 and HER2 status. Archival tumor tissue from the primary tumor (gastric or GEJ) is preferred. If primary tumor tissue is not available, tumor tissue from a metastatic site (excluding bone metastasis) may be used. A minimum of 1 FFPE tumor tissue block (preferred) OR a minimum of 15 FFPE unstained slides are required, as allowed per local policy. If slides are submitted, the slides should be freshly cut from the FFPE block within the time frame described in the laboratory manual. If local HER2 results are already available from local testing, a minimum of 12 FFPE unstained slides are

required to be submitted to the central lab, as allowed per local policy. If the specimen is insufficient or unavailable, a biopsy may be performed to obtain primary tumor tissue (preferred) or tumor tissue from metastatic site (excluding bone metastasis). Sponsor pre-approval is required when the sole purpose of the biopsy procedure is to assess eligibility for this study. See [Section 5.7.3 Tumor Tissue Samples].

If the required number of slides cannot be provided, the sponsor or designee should be contacted for further guidance.

13. Laboratory Assessments: See [Section 5.4.3 Laboratory Assessments] for list of laboratory assessments. Laboratory tests must be sent to the central laboratory for analysis unless otherwise approved by the sponsor. For screening/eligibility laboratory assessments, see footnote number 1.
    - Laboratory test results (central or local) will be reviewed by the investigator prior to any study treatment. Clinical significance of out-of-range laboratory findings is to be determined and documented by the investigator/sub-investigator who is a qualified physician.
    - Local laboratory results may be used for treatment decisions; however, central laboratory samples must also be drawn per protocol and sent to the central laboratory.
    - From cycle 2 onwards, central and local labs may be collected up to 48 hours prior to study treatment.
    - Holidays and weekends should be taken into account when scheduling these blood draws.
    - Additional assessments may be done centrally or locally to monitor AEs or as clinically indicated.
14. Coagulation (PT, PTT and INR): Coagulation tests should be done at Screening and during study treatment period if clinically indicated. Local or central lab results may be used to confirm eligibility. Ongoing evaluation should be continued for Subjects who are receiving therapeutic anticoagulation according to local standard of care. See [Section 5.4.4 Laboratory Assessments].
15. Serum Pregnancy Test: Serum pregnancy tests will be collected for female subjects of child bearing potential only. Serum pregnancy tests collected at Screening, during study treatment period and if clinically indicated or per local requirements. (Note: For Screening, subjects with elevated serum βHCG and a demonstrated non-pregnant status through additional testing are eligible.) Local or central laboratory results may be used to confirm eligibility.
25. Genetic Immune Polymorphism: To be collected per local policy. Whole blood sample taken at C1D1 will be sent to the central laboratory.
26. Exploratory Biomarker (Serum and Plasma): To be collected per local policy. Samples should be taken at the below timepoints and sent to the central laboratory:
    - Cycle 1 Day 1: Predose
    - Cycle 1 Day 22: Predose
    - Cycle 2 Day 1: Predose
    - Cycle 2 Day 22: Predose
    - Cycle 3 Day 1: Predose
    - Cycle 3 Day 22: Predose
    - Cycle 4 Day 22: Predose
    - Zolbetuximab/placebo Study Treatment Discontinuation Visit
27. Optional PGx: for subjects who signed a separate ICF, an optional whole blood sample for PGx for exploratory biomarker analysis may be collected at C1D1 prior to first study drug administration. Sample collection is optional and only collected as allowed per local policy.
28. Optional Post-Progression Tumor Sample: for subjects who signed a separate ICF, an optional post-progression tumor sample for exploratory biomarker analysis may be collected following IRC confirmation of disease progression and prior to initiation of subsequent anti-cancer therapy. Sample collection is optional and only collected as allowed per local policy.

IS AMENDED TO:

VISIT	Screening[1]	Study Treatment Period (Each Cycle = approximately 42 Days)								Follow-up Period				
		Cycles 1 to 4<br>Zolbetuximab/Placebo<br>+<br>CAPOX				Cycle 5+<br>Zolbetuximab/Placebo<br>+<br>5FU + Folinic Acid<br>(at investigator discretion)				Study Treatment Discontinuation Visit[18]	30-Day Safety Follow-up Visit(s)[19]	90-Day Safety Follow-up Visit(s)[20]	Post Treatment Follow-up Period[21]	Long Term and Survival Follow-up Periods[22]
Day		1[5]	15	22	29	1	15	22	29					
Visit Window (calendar days)	-45 to -1	+ 7*	+ 7	+ 7	+ 7	+ 7	+ 7	+ 7	+ 7	+ 7	+ 7	± 7	± 7	± 14
~~HER2 Tumor Sample[2]~~	~~X~~													
Sampling														
~~Genetic Immune Polymorphisms (Whole Blood)[25]~~		~~X~~												
~~Exploratory Biomarkers (Serum)[26]~~		~~X~~		~~X~~						~~X~~				
~~Exploratory Biomarkers (Plasma)[26]~~		~~X~~		~~X~~						~~X~~				
~~Whole Blood Sample for PGx (optional)[27]~~		~~X~~												
~~Post Progression Tumor Sample (optional)[28]~~										~~X~~				
Concomitant Medication[~~29~~25]	X	X	X	X	X	X	X	X	X	X	X	X		
AEs/SAEs[~~30~~26]	X	X	X	X	X	X	X	X	X	X	X	X		

~~HER2: human epidermal growth factor reception 2; PGx: pharmacogenomics~~

1. Screening: The Screening period is 45 days from ICF signature. Re-testing of lab values is allowed within the 45-day screening period. Re-screening outside of the 45-day window under a new subject number may be allowed upon discussion with the Medical Monitor.
   - Optional Partial Screening: A partial screening ICF is available for central testing of tissue for CLDN18.2 ~~and HER2~~ only.
   - Laboratory testing:

     Eligibility can be determined based on central and/or local testing, **however**:
     - The most recent laboratory data must be used to confirm the subject's eligibility.
     - Central labs must be collected and submitted to the central laboratory during the Screening period.
     - If retesting of lab values is necessary to confirm eligibility, local labs can be used without requiring additional sample collection for central laboratory submission.
     - The screening labs used to determine eligibility should be collected within 14 days prior to randomization.
   - Radiologic imaging used to confirm eligibility must be conducted within 28 days prior to randomization.
2. CLDN18.2 ~~and HER2~~ Testing: FFPE tumor tissue will be collected for central testing to determine CLDN18.2 ~~and HER2~~ status. Archival tumor tissue from the primary tumor (gastric or GEJ) is preferred. If primary tumor tissue is not available, tumor tissue from a metastatic site (excluding bone metastasis) may be used. ~~A minimum of 1 FFPE tumor tissue block (preferred) OR a minimum of 15~~**Five** FFPE unstained slides are required, as allowed per local policy. ~~If slides are submitted, t~~**T**he slides should be freshly cut from the FFPE block within the time frame described in the laboratory manual. ~~If local HER2 results are already available from local testing, a minimum of 12 FFPE unstained slides are required to be submitted to the central lab, as allowed per local policy.~~ If the specimen is insufficient or unavailable, a biopsy may be performed to obtain primary tumor tissue (preferred) or tumor tissue from metastatic site (excluding bone metastasis). Sponsor pre-approval is **requested**~~required~~ when the sole purpose of the biopsy procedure is to assess

eligibility for this study. See [Section 5.7.3 Tumor Tissue Samples].

~~If the required number of slides cannot be provided, the sponsor or designee should be contacted for further guidance.~~

13. Laboratory Assessments: See [Section 5.4.3 Laboratory Assessments] for list of laboratory assessments. ~~Laboratory tests must be sent to the central laboratory for analysis unless otherwise approved by the sponsor.~~ For screening/eligibility laboratory assessments, see footnote number 1.
    - Laboratory test results ~~(central or local)~~ will be reviewed by the investigator prior to any study treatment. Clinical significance of out-of-range laboratory findings is to be determined and documented by the investigator/sub-investigator who is a qualified physician.
    - Local laboratory results may be used for treatment decisions; however, central laboratory samples **should**~~must~~ also be drawn per protocol and sent to the central laboratory unless otherwise approved by the sponsor.
    - From cycle 2 onwards, central and local labs may be collected up to 48 hours prior to study treatment.
    - Holidays and weekends should be taken into account when scheduling these blood draws.
    - Additional assessments may be done centrally or locally to monitor AEs or as clinically indicated.
14. Coagulation (PT, PTT and INR): Coagulation tests should be done at Screening and during study treatment period if clinically indicated. ~~Local or central lab results may be used to confirm eligibility.~~ Ongoing evaluation should be continued for Subjects who are receiving therapeutic anticoagulation according to local standard of care. See [Section 5.4.3 Laboratory Assessments].
15. Serum Pregnancy Test: Serum pregnancy tests will be collected for female subjects of child bearing potential only. Serum pregnancy tests collected at Screening, during study treatment period and if clinically indicated or per local requirements. (Note: For Screening, subjects with elevated serum βHCG and a demonstrated non-pregnant status through additional testing are eligible.) ~~Local or central laboratory results may be used to confirm eligibility.~~

~~25. Genetic Immune Polymorphism: To be collected per local policy. Whole blood sample taken at C1D1 will be sent to the central laboratory.~~

~~26. Exploratory Biomarker (Serum and Plasma): To be collected per local policy. Samples should be taken at the below timepoints and sent to the central laboratory:~~
- ~~Cycle 1 Day 1: Predose~~
- ~~Cycle 1 Day 22: Predose~~
- ~~Cycle 2 Day 1: Predose~~
- ~~Cycle 2 Day 22: Predose~~
- ~~Cycle 3 Day 1: Predose~~
- ~~Cycle 3 Day 22: Predose~~
- ~~Cycle 4 Day 22: Predose~~
- ~~Zolbetuximab/placebo Study Treatment Discontinuation Visit~~

~~27. Optional PGx: for subjects who signed a separate ICF, an optional whole blood sample for PGx for exploratory biomarker analysis may be collected at C1D1 prior to first study drug administration. Sample collection is optional and only collected as allowed per local policy.~~

~~28. Optional Post Progression Tumor Sample: for subjects who signed a separate ICF, an optional post progression tumor sample for exploratory biomarker analysis may be collected following IRC confirmation of disease progression and prior to initiation of subsequent anti-cancer therapy. Sample collection is optional and only collected as allowed per local policy.~~

**IV Synopsis, Study Objective(s) and 2 Study Objective(s), Design, and Endpoints**<br>*2.1.3 Exploratory Objectives*
WAS:
• To evaluate potential genomic and/or other biomarkers that may correlate with treatment outcome to zolbetuximab and CAPOX.
IS AMENDED TO:
• To evaluate potential genomic and/or other biomarkers that may correlate with treatment outcome to zolbetuximab and CAPOX. **[NOTE: Genomic and exploratory biomarker testing will not be performed on samples from subjects in China.]**

**IV Synopsis, Study Design Overview and 2 Study Objective(s), Design, and Endpoints**<br>*2.2.1 Study Design*
WAS:
Screening:<br>The Screening period is 45 days from informed consent form (ICF) signature. Retesting of lab values is allowed within the 45-day Screening period. Re-screening outside the 45-day window under a new subject number may be allowed once and upon discussion with the medical monitor.<br>An optional partial screening ICF is available to allow central testing of tissue for CLDN18.2 and HER2 only.<br>Formalin fixed paraffin embedded (FFPE) tumor tissue will be collected for central testing to determine CLDN18.2 and HER2 status.<br>• Archival tumor tissue is preferred.<br>o A minimum of 1 FFPE tumor tissue block (preferred) OR a minimum of 15 FFPE unstained slides are required, as allowed per local policy. If slides are submitted, the slides should be freshly cut from the FFPE block within the time frame described in the laboratory manual.<br>o If local HER2 results are already available from local testing, a minimum* of 12 FFPE unstained slides are required to be submitted to the central laboratory, as allowed per local policy.<br>*If the required minimum number of slides is not able to be submitted, sponsor notification and approval is required.
IS AMENDED TO:
Screening:<br>The Screening period is 45 days from informed consent form (ICF) signature. Retesting of lab values is allowed within the 45-day Screening period. Re-screening outside the 45-day window under a new subject number may be allowed once and upon discussion with the medical monitor.<br>An optional partial screening ICF is available to allow central testing of tissue for CLDN18.2 ~~and HER2~~ only.<br>Formalin fixed paraffin embedded (FFPE) tumor tissue will be collected for central testing to

determine CLDN18.2 ~~and HER2~~ status.

- Archival tumor tissue is preferred.
  - ~~A minimum of 1 FFPE tumor tissue block (preferred) OR a minimum of 15~~ **Five** FFPE unstained slides are required, as allowed per local policy. ~~If slides are submitted, t~~**T**he slides should be freshly cut from the FFPE block within the time frame described in the laboratory manual.
  - ~~If local HER2 results are already available from local testing, a minimum* of 12 FFPE unstained slides are required to be submitted to the central laboratory, as allowed per local policy.~~

    ~~*If the required minimum number of slides is not able to be submitted, sponsor notification and approval is required.~~

2 Study Objective(s), Design, and Endpoints
2.2.1 Study Design

WAS:

Re-screening

Subjects who have failed screening are allowed to be re-screened one time after consultation with the medical monitor. Upon re-screening, a new subject number will be assigned. Subjects have to re-consent to the study and all screening procedures must be repeated, with the exception of the CLDN18.2 and HER 2 testing, as well as the radiologic imaging procedure to confirm eligibility if the scan is within 28 days prior to randomization.

IS AMENDED TO:

Re-screening

Subjects who have failed screening are allowed to be re-screened one time after consultation with the medical monitor. Upon re-screening, a new subject number will be assigned. Subjects have to re-consent to the study and all screening procedures must be repeated, with the exception of the CLDN18.2 ~~and HER 2~~ testing, as well as the radiologic imaging procedure to confirm eligibility if the scan is within 28 days prior to randomization.

IV Synopsis, Endpoints for Evaluation and 2 Study Objective(s), Design, and Endpoints
2.3.3 Exploratory Endpoints

WAS:

- Potential genomic and/or other exploratory biomarkers that may be related to treatment outcome of zolbetuximab.

IS AMENDED TO:

- Potential genomic and/or other exploratory biomarkers that may be related to treatment outcome of zolbetuximab. **[NOTE: Genomic and exploratory biomarker testing will not be performed on samples from subjects in China.]**

**IV Synopsis, Inclusion/Exclusion Criteria and 3 Study Population**<br>*3.2 Inclusion Criterion #14*
WAS:
Subject has a HER2-Negative tumor as determined by local or central testing on a gastric or GEJ tumor specimen.
IS AMENDED TO:
Subject has a **known** HER2-Negative **gastric or GEJ** tumor ~~as determined by local or central testing on a gastric or GEJ tumor specimen~~.

**5 Treatments and Evaluation**<br>*5.6 Test Drug Concentration*
DELETED:
~~Samples remaining after pharmacokinetic assessments may be used for additional biomarker analysis described in [Section 5.7.1 Biomarkers].~~

**5 Treatments and Evaluation**<br>*5.7.1 Biomarkers, 5.7.2 Blood, Serum and Plasma Samples*
DELETED:
~~5.7.1 Biomarkers~~<br>~~Tumor tissue and blood/serum/plasma samples described in [Sections 5.7.2 Blood, Serum and Plasma Samples and 5.7.3 Tumor Tissue Samples] may be used for research purposes as allowed per local policy to identify genomic and/or other biomarkers that may be associated with clinical outcome or dynamic changes associated with zolbetuximab treatment (in terms of dose, safety, tolerability and efficacy). Since the identification of exploratory biomarkers that correlate with the efficacy or safety of zolbetuximab treatment may continue to evolve as new findings becomes available, additional analyses related to zolbetuximab activity on tumor signaling pathways or clinical outcomes may be conducted as allowed per local policy. Tumor tissue and blood/serum samples remaining after the specified biomarker assessments (e.g., aliquots of tumor cell RNA or DNA) may be used for re-testing, additional analyses as defined above or developing, and validating assays related to prediction of response or dynamic changes associated with zolbetuximab treatment. The tumor tissue and blood/serum/plasma samples (e.g., aliquots of tumor cell RNA or DNA, peripheral blood mononuclear cells) will be stored at the study sponsors' facility or a contract laboratory facility for up to 15 years after database closure, at which time the samples will be destroyed. The procedures for the collection, handling and shipping of laboratory samples being submitted to the central laboratory will be specified in a laboratory manual.~~<br>~~5.7.2 Blood, Serum and Plasma Samples~~<br>~~Blood, serum and plasma samples will be collected as allowed per local policy according to the Schedule of Assessments [Table 1] for exploratory biomarker measurements. Blood, serum and plasma samples may be analyzed for biomarkers including but not limited to chemokines, cytokines, CDC activation, circulating DNA soluble factors and genetic markers.~~

5 Treatments and Evaluation
5.7.3 Tumor Tissue Samples

WAS:

5.7..3 Tumor Tissue Samples

FFPE tumor tissue samples will be obtained for all subjects and sent for central IHC testing to evaluate for CLDN18.2, and HER2 status if a previously documented HER2 test result is not available. Tissue from the primary site is preferred; however, if a metastatic site is used (excluding bone metastasis), the sample should be gastric or GEJ in origin. Archival tumor tissue is preferred, but if the specimen is insufficient or unavailable, a biopsy may be performed to obtain a primary tumor tissue or tumor tissue from metastatic site (excluding bone metastasis). Sponsor preapproval is required when a biopsy procedure is needed for the sole purpose of determining study eligibility. Optional post-progression tumor tissue sample for exploratory biomarker analysis may be collected, as allowed per local policy following IRC confirmation of disease progression and prior to initiation of subsequent anti-cancer therapy for subjects who sign a separate ICF. Tumor specimens may be analyzed for exploratory biomarkers including but not limited to CLDN18.2 expression, immune cells, genetic markers and gene/protein expression, as allowed per local policy.

Tumor Tissue Requirements

Visit	Tumor Tissue Requirement
Screening	A minimum of 1 FFPE tumor tissue block (preferred) OR a minimum* of 15 FFPE unstained slides are required, as allowed per local policy.<br>If local HER2 results are available, a minimum* of 12 slides are required along with the pathology report/documented test results. If local HER2 results are unavailable, follow guidance above.
Post-Progression (optional)	A minimum of 1 FFPE tumor tissue block (preferred) OR a minimum* of 15 FFPE unstained slides are required, as allowed per local policy.

FFPE: formalin-fixed paraffin embedded; HER2: human epidermal growth factor receptor 2
*If the required minimum number of slides is not able to be submitted, sponsor notification and approval is required.

IS AMENDED TO:

5.7.1~~5.7..3~~ Tumor Tissue Samples

FFPE tumor tissue samples will be obtained for all subjects and sent for central IHC testing to evaluate for CLDN18.2~~, and HER2~~ status~~ if a previously documented HER2 test result is not available~~. Tissue from the primary site is preferred; however, if a metastatic site is used (excluding bone metastasis), the sample should be gastric or GEJ in origin. Archival tumor tissue is preferred, but if the specimen is insufficient or unavailable, a biopsy may be performed to obtain a primary tumor tissue or tumor tissue from metastatic site (excluding bone metastasis). Sponsor preapproval is required when a biopsy procedure is needed for the sole purpose of determining study eligibility. ~~Optional post-progression tumor tissue sample for exploratory biomarker analysis may be collected, as allowed per local policy following IRC confirmation of disease progression and prior to initiation of subsequent anti-cancer therapy for subjects who sign a separate ICF. Tumor specimens may be analyzed for~~

~~exploratory biomarkers including but not limited to CLDN18.2 expression, immune cells, genetic markers and gene/protein expression, as allowed per local policy.~~

The tumor tissue will be stored at the study sponsor's facility or at a contract laboratory facility for up to 10 years after study completion, at which time the samples will be destroyed, as allowed per local policy. The procedures for the collection, handling and shipping of laboratory samples being submitted to the central laboratory will be specified in a laboratory manual.

Tumor Tissue Requirements

Visit	Tumor Tissue Requirement
Screening	~~A minimum of 1 FFPE tumor tissue block (preferred) OR a minimum* of 15~~ **Five** FFPE unstained slides are required, as allowed per local policy.<br>~~If local HER2 results are available, a minimum* of 12 slides are required along with the pathology report/documented test results. If local HER2 results are unavailable, follow guidance above.~~
~~Post Progression (optional)~~	~~A minimum of 1 FFPE tumor tissue block (preferred) OR a minimum* of 15 FFPE unstained slides are required, as allowed per local policy.~~

FFPE: formalin-fixed paraffin embedded~~; HER2: human epidermal growth factor receptor 2~~

~~*If the required minimum number of slides is not able to be submitted, sponsor notification and approval is required.~~

5 Treatments and Evaluation
5.7.4 Immunogenicity Assessment (ADA)

DELETED:

~~Samples remaining after immunogenicity assessments may be used for additional biomarker analysis, as allowed per local policy, as described in [Section 5.7.1 Biomarkers].~~

5 Treatments and Evaluation
5.7.5 Optional Samples for Future PGx Analysis

DELETED:

~~5.7.5 Optional Samples for Future PGx Analysis~~

~~For subjects who signed a separate ICF, an optional whole blood sample for pharmacogenomics will be collected at C1D1 prior to first study drug administration, as allowed per local policy. PGx research may be conducted in the future to analyze or determine genes of relevance to clinical response, pharmacokinetics and toxicity/safety issues. A sample of whole blood for possible retrospective PGx analysis will be collected and processed, as allowed per local policy. Blood sampling, processing, storage and shipment instructions will be provided in the Laboratory Manual. Samples will be shipped to a sponsor-designated analytical storage laboratory. Please refer to the Laboratory Manual for more detailed information.~~

~~See [Appendix 12.7, Retrospective PGx Sub-study] for further details on the banking procedures.~~

**12 Appendices**<br>*12.1.4.1 Subject Information and Consent*
WAS:
An optional partial screening ICF is available to allow central testing of tissue for CLDN18.2 and HER2 only.
IS AMENDED TO:
An optional partial screening ICF is available to allow central testing of tissue for CLDN18.2 ~~and HER2~~ only.

**12 Appendices**<br>*12.7 Pharmcogenomic (PGx) Analysis with Banked Samples (Optional)*
DELETED:
~~12.7 Pharmacogenomic (PGx) Analysis with Banked Samples (Optional)~~<br>~~INTRODUCTION~~<br>~~PGx research aims to provide information regarding how naturally occurring changes in a subject's gene and/or expression based on genetic variation may impact what treatment options are best suited for the subject. Through investigation of PGx by technologies such as genotyping, gene sequencing, statistical genetics and Genome-Wide Association Studies, the relationship between gene profiles and a drug's kinetics, efficacy or toxicity may be better understood. As many diseases may be influenced by 1 or more genetic variations, PGx research may identify which genes are involved in determining the way a subject may or may not respond to a drug. Samples for PGx are optional and will only be collected as allowed per local policy.~~<br>~~OBJECTIVES~~<br>~~The PGx research that may be conducted in the future with acquired blood samples is exploratory. The objective of this research will be to analyze or determine genes of relevance to clinical response, pharmacokinetics and toxicity/safety issues.~~<br>~~By analyzing genetic variations, it may be possible to predict an individual subject's response to treatment in terms of efficacy and/or toxicity.~~<br>~~SUBJECT PARTICIPATION~~<br>~~Subjects who have consented to participate in this study may participate in this PGx sub-study, if applicable per local policy. Subjects must provide written consent prior to providing any blood samples that may be used at a later time for PGx analysis.~~<br>~~**SAMPLE COLLECTION AND STORAGE**~~<br>~~Subjects who consent to participate in this sub-study will provide 1 approximately 4–6 mL tube of whole blood per Astellas' instructions. Each sample will be identified by the unique subject number. Samples will be shipped to a designated banking CRO as directed by Astellas.~~<br>~~PGx ANALYSIS~~

~~Details on the potential PGx analysis cannot be established yet. Astellas may initiate the PGx analysis if evidence suggests that genetic variants may be influencing the drug's kinetics, efficacy and/or safety.~~
~~DISPOSAL OF PGx SAMPLES / DATA~~
~~All PGx samples collected will be stored for a period of up to 15 years following study database hard-lock. If there is no requirement for analysis, the whole blood sample will be destroyed after the planned storage period. The subject has the right to withdraw consent at any time. When a subject's withdraw notification is received, the PGx sample will be destroyed. The results of any PGx analysis conducted on a sample prior to its withdrawal will be retained at Astellas indefinitely unless otherwise specified by local regulation.~~
~~INFORMATION DISCLOSURE TO THE SUBJECTS~~
~~Exploratory PGx analysis may be conducted following the conclusion of the clinical study, if applicable. The results of the PGx analysis will not be provided to any investigators or subjects, nor can the results be requested at a later date. Any information that is obtained from the PGx analysis will be the property of Astellas.~~

IIB. Non-Substantial Changes

**IV Synopsis, Planned Total Number of Study Centers and Location(s)**
WAS:
Approximately 175 centers globally.
IS AMENDED TO:
Approximately 175 centers globally**, with approximately 50 centers in China**.

**IV Synopsis, Number of Subjects to be Enrolled/Randomized**
WAS:
Approximately 500 subjects.
IS AMENDED TO:
Approximately 500 subjects**, with approximately 250 subjects in China**.

**IV Synopsis, Study Design Overview and 2 Study Objective(s), Design, and Endpoints**<br>*2.2.1 Study Design*
WAS:
Screening:<br>• If the specimen is insufficient or unavailable, a biopsy may be performed to obtain tumor sample.<br>○ Sponsor pre-approval is required when the sole purpose of the biopsy procedure is to assess eligibility for this study.

o If the required number of slides cannot be provided, the sponsor or designee should be contacted for further guidance.
IS AMENDED TO:
Screening:<br>• If the specimen is insufficient or unavailable, a biopsy may be performed to obtain tumor sample.<br>o Sponsor pre-approval is **requested**~~required~~ when the sole purpose of the biopsy procedure is to assess eligibility for this study.<br>o If the required number of slides cannot be provided, the sponsor or designee should be contacted for further guidance.

**4 Identification of Study Treatment(s)**<br>***4.2 Comparative Drug (Placebo)***
WAS:
Placebo will not be manufactured or provided by the sponsor.
IS AMENDED TO:
Placebo will not be manufactured ~~or provided~~ by the sponsor.

**5 Treatments and Evaluation**<br>*5.4.3 Laboratory Assessments*
WAS:
Laboratory tests will be performed according to the Schedule of Assessments [Table 1] and must be sent to the central laboratory for analysis.<br>Eligibility can be determined based on central and/or local laboratory testing.<br>• The most recent laboratory data must be used to confirm the subject's eligibility.<br>o Central labs **must** be collected and submitted to the central laboratory during the Screening period.<br>o If retesting of lab values is necessary to confirm eligibility, local labs can be used without requiring additional sample collection for central laboratory submission.<br>o The screening labs used to determine eligibility should be collected within 14 days prior to randomization.<br>• Laboratory test results (central or local) will be reviewed by the investigator prior to any study treatment. Clinical significance of out of range laboratory findings is to be determined and documented by the investigator/subinvestigator who is a qualified physician.<br>• Local laboratory results may be used for treatment decisions; however, central laboratory samples must also be drawn per protocol and sent to the central laboratory.<br>• From Cycle 2 onwards, central and local labs may be collected up to 48 hours prior to study treatment.

IS AMENDED TO:
Laboratory tests will be performed according to the Schedule of Assessments [Table 1] ~~and must be sent to the central laboratory for analysis~~.<br>Eligibility can be determined based on central and/or local laboratory testing.<br>• The most recent laboratory data must be used to confirm the subject's eligibility.<br>• Central labs **must** be collected and submitted to the central laboratory during the Screening period.<br>• If retesting of lab values is necessary to confirm eligibility, local labs can be used without requiring additional sample collection for central laboratory submission.<br>• The screening labs used to determine eligibility should be collected within 14 days prior to randomization.<br>• Laboratory test results ~~(central or local)~~ will be reviewed by the investigator prior to any study treatment. Clinical significance of out of range laboratory findings is to be determined and documented by the investigator/subinvestigator who is a qualified physician.<br>• Local laboratory results may be used for treatment decisions; however, central laboratory samples **should**~~must~~ also be drawn per protocol and sent to the central laboratory**, unless otherwise approved by sponsor**.<br>• From Cycle 2 onwards, ~~central and local~~ labs may be collected up to 48 hours prior to study treatment.

**5 Treatments and Evaluation**<br>*5.8 Total Amount of Blood*
WAS:
The maximum amount of blood collected is approximately 40 mL in cycle 1, and less in later cycles.
IS AMENDED TO:
The maximum amount of blood collected is approximately **10**~~40~~ mL **at screening**~~in cycle 1~~, and less **at**~~in~~ later **visits**~~cycles~~.

**8 Operational and Administrative Considerations**<br>*8.1.1 Data Collection*
WAS:
Local laboratory results may be used for treatment decisions; however, central laboratory samples must also be drawn per protocol and send to the central laboratory, unless otherwise approved by sponsor.
IS AMENDED TO:
Local laboratory results may be used for treatment decisions; however, central laboratory samples **should**~~must~~ also be drawn per protocol and send to the central laboratory, unless otherwise approved by sponsor.

**12 Appendices**<br>*12.1.5 Source Documents*
WAS:
Paper records from electronic systems used in place of electronic format must be certified copies. A certified copy must be an exact copy and must have all the same attributes and information as the original. Certified copies must include signature and date of the individual completing the certification. Certified copies must be a complete and chronological set of study records (including notes, attachments, and audit trail information (if applicable). All printed records must be kept in the subject file and available for archive.
IS AMENDED TO:
Paper records from electronic systems used in place of electronic format **should**~~must~~ be certified copies. A certified copy **should**~~must~~ be an exact copy and must have all the same attributes and information as the original. Certified copies **should**~~must~~ include signature and date of the individual completing the certification. Certified copies **should**~~must~~ be a complete and chronological set of study records (including notes, attachments, and audit trail information (if applicable). All printed records **should**~~must~~ be kept in the subject file and available for archive.

14 COORDINATING INVESTIGATOR’S SIGNATURE

A Phase 3, Global, Multi-Center, Double-Blind, Randomized, Efficacy Study of Zolbetuximab (IMAB362) Plus CAPOX Compared with Placebo Plus CAPOX as First-line Treatment of Subjects with Claudin (CLDN)18.2-Positive, HER2-Negative, Locally Advanced Unresectable or Metastatic Gastric or Gastroesophageal Junction (GEJ) Adenocarcinoma

ISN/Protocol 8951-CL-0302

Version 4.0 [CN] Incorporating Country-specific Substantial Amendment 3

29 Aug 2019

I have read all pages of this clinical study protocol for which Astellas is the sponsor. I agree that it contains all the information required to conduct this study.

Coordinating Investigator:

Signature: ______________________________ ______________
<Insert name, department/affiliation, name of institution> Date (DD Mmm YYYY)

Printed Name: ______________________________

Address: ______________________________

____

15 SPONSOR'S SIGNATURES

13 ATTACHMENT 1: SUBSTANTIAL AMENDMENT 1

I. The purpose of this amendment is:

**Substantial Changes**
**1. Update Inclusion Criteria regarding physical or laboratory findings.**
DESCRIPTION OF CHANGE:
Update Inclusion Criteria regarding physical or laboratory findings to remove serum creatinine value and test estimated creatinine clearance (≥ 30 mL/min) instead of estimated glomerular filtration rate (≥ 50 mL/min/1.73m^2). Add a bullet to exclude subjects with an estimated creatinine clearance < 30 mL/min. Add creatinine clearance to the list of biochemistry laboratory tests.
RATIONALE:
To align with the requirement in the updated oxaliplatin SmPC, dated 02Jan2018, that patients with severely impaired renal function (defined as creatinine clearance < 30 ml/min) not be administered oxaliplatin.
**2. Clarify Exclusion Criterion 4 regarding allowable use of systemic corticosteroids.**
DESCRIPTION OF CHANGE:
Clarify the exclusion of subjects who have received systemic immunosuppressive therapy, including systemic corticosteroids, within 14 days prior to the first dose of study treatment in Exclusion Criterion 4 to allow a single dose of systemic corticosteroids.<br>Add clarification to Concomitant Medication Restrictions or Requirements to allow a single dose of systemic corticosteroids.
RATIONALE:
To clarify that patients requiring only a single dose of corticosteroids within 14 days prior to the first dose of study treatment are allowed to participate as this level of corticosteroid exposure is unlikely to affect zolbetuximab efficacy.
**3. Update Exclusion Criterion 6 to include reaction to known ingredients of zolbetuximab.**
DESCRIPTION OF CHANGE:
Exclusion Criterion 6 is revised to add that this criterion applies to known ingredients of zolbetuximab, as well as other monoclonal antibodies.
RATIONALE:
To clarify that patients with a known severe allergic reaction or intolerance to zolbetuximab specifically should be excluded.

**4. Update Exclusion Criterion 9 and Schedule of Assessments (SOA) to include screening for dihydropyrimidine dehydrogenase (DPD) deficiency. Remove the Note regarding treatment with capecitabine.**
DESCRIPTION OF CHANGE:
Update Exclusion Criterion 9 and SOA to clarify that sites should include screening test for DPD deficiency according to local requirements. Remove the note regarding treatment with capecitabine. Add DPD deficiency alleles to the list of tests in the section describing laboratory testing.
RATIONALE:
To clarify that testing for DPD deficiency should be conducted per local requirements based on variable standards of care by geographical region.
**5. Update safety measures and exclude subjects with a history or a family history of congenital long QT syndrome.**
DESCRIPTION OF CHANGE:
Add a bullet to Exclusion Criterion 15 to exclude subjects with a history or a family history of congenital long QT syndrome.<br>Delete instructions to avoid the use of antiemetic premedications in subjects with congenital long QT syndrome.<br>Remove the stipulation specific to subjects with congenital long QT syndrome in the caution regarding the use of the class of 5-HT3-blockers.
RATIONALE:
To exclude subjects at increased risk for ventricular arrhythmias due to QT prolongation with oxaliplatin administration as recommended per the updated oxaliplatin SmPC, dated 02 Jan 2018. The instructions regarding avoidance of use of antiemetic premedications in subjects with congenital long QT syndrome was deleted as these subjects will now be excluded based on exclusion criteria 15.
**6. Add cautionary language regarding the use of medications known to prolong the QT and QTc interval.**
DESCRIPTION OF CHANGE:
Add the following to the list of medication to be avoided or used with caution and closely monitored during CAPOX (capecitabine and oxaliplatin) administration.<br>• Cautionary language regarding the use of medications known to prolong the QT and QTc interval<br>• Cross-reference to the website listing medications known to prolong QT or QTc interval<br>Add a reference to Appendix 12.4 Concomitant Medical Restrictions or Requirements in Section 5.1.5.

RATIONALE:
To minimize the risk of ventricular arrhythmias due to QT prolongation by avoiding or using with caution medications known to prolong the QT interval.
**7. Clarify the timing of major surgical procedures and complete recovery from them in Exclusion Criterion 18.**
DESCRIPTION OF CHANGE:
Clarify Exclusion Criterion 18 by separately addressing the timing allowed before the start of study treatment after having a major surgical procedure (≤ 28 days) and after having complete recovery from a major surgical procedure (≤ 14 days).
RATIONALE:
To clarify that subjects with a major surgical procedure ≤ 28 days before start of study treatment, irrespective of recovery status, should be excluded. To clarify that subjects without complete recovery from a major surgical procedure at least 14 days before start of study treatment, should be excluded.
**8. Clarify ECG collection relative to oxaliplatin infusion.**
DESCRIPTION OF CHANGE:
Add text to require ECG collection prior to every oxaliplatin infusion (before any antiemetic treatment) and following completion of every oxaliplatin infusion. Ensure that potassium, magnesium and calcium levels are within normal limits prior to oxaliplatin infusion.<br>Add that additional ECG monitoring should be initiated for subjects who experience syncope, presyncope, palpitations and/or bradycardia per local standard of care. Add medical management per local standard of care and correction of hypokalemia, hypomagnesemia and/or hypocalcemia for QTc interval > 450 msec. Add medical management per local standard of care for QTc interval > 500 msec, including withholding oxaliplatin and Capecitabine treatment, ensuring continuous ECG monitoring and obtaining a cardiology consult.<br>Clarify the schedule of performance of ECGs in the footnotes to the SOA.
RATIONALE:
To minimize the risk of ventricular arrhythmias due to QT prolongation by closely monitoring the QT interval before and after administration of oxaliplatin and by monitoring electrolytes as per the updated oxaliplatin SmPC, dated 02Jan2018.<br>To provide guidance for additional ECG monitoring based on patient symptoms and/or QT interval prolongation.

**9. Clarify the timing of assessment of adverse events (AEs) and serious adverse events (SAEs). Clarify the timing of the collection of concomitant medication usage data.**
DESCRIPTION OF CHANGE:
Clarify that the assessment of AEs/SAEs and concomitant medications will be done from the time of informed consent through 90 days following the last dose of study treatment.
RATIONALE:
The proposed 90-day notification for AEs is equivalent to 5.2 to 6.2 times $t_{1/2}$, which is necessary to capture AEs that may be related to treatment with zolbetuximab.
**10. Update criteria for discontinuation of zolbetuximab/placebo and CAPOX based on treatment delay.**
DESCRIPTION OF CHANGE:
Update in study treatment and discontinuation criteria and in study treatment dose modification and discontinuation criteria that a delay of study treatment > 28 days from when the next study treatment was scheduled to be administered for any reason will result in treatment discontinuation.
RATIONALE:
To ensure a favorable benefit/risk balance for subjects, the study treatment discontinuation criteria was modified to require that all subjects with a delay of study treatment > 28 days from when the next study treatment was scheduled to be administered must discontinue study treatment.
**11. Update text regarding SAE reporting of fatal death.**
DESCRIPTION OF CHANGE:
Update the text regarding disease progression in Section 5.5.1.3 to include that all deaths that occur up to 90 days after the last dose of study drug must be reported as SAE, even if attributed to disease progression.
RATIONALE:
To ensure consistent capture of fatal SAEs irrespective of attribution to disease progression.

**Non-Substantial Changes**
**1. Minor Administrative-type Changes**
DESCRIPTION OF CHANGE:
Include minor administrative-type changes, e.g., typos, format, spell out on first use, list of abbreviations, numbering and edits for consistency throughout the protocol.

RATIONALE:
To provide clarifications to the protocol and to ensure complete understanding of study procedures.
**2. Changes to contact details of key sponsor's personnel.**
DESCRIPTION OF CHANGE:
Changes to contact details of key sponsor's personnel.
RATIONALE:
To update the sponsor personnel and contact details in Japan.
**3. Update to include the generic name for IMAB362.**
DESCRIPTION OF CHANGE:
Update to include the generic name for IMAB362, zolbetuximab.
RATIONALE:
Administrative update to include the generic name for IMAB362, zolbetuximab.
**4. Update the number of study centers.**
DESCRIPTION OF CHANGE:
Update the planned number of study centers from 125 to 130.
RATIONALE:
Updated to increase the number of participating centers.
**5. Remove restriction that CLDN18.2 and HER2 status should be obtained prior to initiation of screening procedures.**
DESCRIPTION OF CHANGE:
Remove restriction that confirmation of CLDN18.2 and HER2 status should be obtained prior to allowing subjects to proceed to any of the other screening procedures.
RATIONALE:
Removing restriction that CLDN18.2 and HER2 status should be obtained prior to initiation of screening procedures to allow for concurrent testing as per institutional guidelines and standard of care.
**6. Clarify that the timing of image assessments is counted from Cycle 1 Day 1 (C1D1).**
DESCRIPTION OF CHANGE:
Clarify that the timing of image assessments is counted from C1D1.

RATIONALE:
Administrative change to align with footnote to #12 of the SOA where the timing of imaging assessments is specified as starting from C1D1.
**7. Describing cytokine/chemokine and/or tryptase testing in SOA to be done, if clinically indicated.**
DESCRIPTION OF CHANGE:
Providing clarification that cytokine/chemokine and/or tryptase testing in the SOA should be performed, if clinically indicated. Add cytokine/chemokine and tryptase testing to the list of laboratory panel assessments.
RATIONALE:
To provide consistent guidance within the protocol for specific laboratory tests (cytokine/chemokine and/or tryptase testing) that are described in Table 4 Infusion Related Reactions.
**8. Clarify the subject randomization process.**
DESCRIPTION OF CHANGE:
Clarify the subject randomization process in the footnote of the SOA and in Section 4.7 Assignment and Allocation to explain who performs randomization via interactive response technology (IRT) and who will be notified of the randomly assigned treatment by the IRT system.
RATIONALE:
Updated to allow blinded site personnel to perform the randomization transaction in IRT in alignment with the pharmacy manual.
**9. Clarify sample collection of genetic immune polymorphism.**
DESCRIPTION OF CHANGE:
Clarify sample collection of genetic immune polymorphism is to be done per local policy.
RATIONALE:
Change to clarify sample collection as allowed per local standards.
**10. Clarify the usage of local versus central laboratory testing results.**
DESCRIPTION OF CHANGE:
Clarify that local laboratory results may be used for treatment decisions; however, central laboratory samples must be collected per protocol and sent to the central laboratory.

RATIONALE:
To provide clarification on the guidance of collection and use of local and central labs for treatment decisions.
**11. Update cross-references to the Investigator's Brochure (IB) and the wording of the potential risk/benefit of zolbetuximab treatment in combination with CAPOX.**
DESCRIPTION OF CHANGE:
Update the cross-references to the IB section describing potential zolbetuximab toxicities and expected adverse drug reactions in Section 1.3 and update the wording of the summary of potential risks and benefits of zolbetuximab in combination with CAPOX in Section 1.5.
RATIONALE:
To update section numbers for cross-references to IB sections for key safety information and to clarify risk/benefit assessment to include identified and potential risks.
**12. Remove details regarding reconstitution of investigational drug to Appendix and add a cross-reference.**
DESCRIPTION OF CHANGE:
Remove directions regarding reconstitution of investigational drug in the section describing the investigational product zolbetuximab. Add a cross-reference to the Pharmacy Manual in the section describing the comparative drug (placebo) as preparation details are described there.
RATIONALE:
Directions regarding reconstitution of investigational drug are described in detail in the Pharmacy Manual, which is the primary source of information for investigational drug preparation and storage.
**13. Update language regarding the availability of zolbetuximab after conclusion of the study or upon premature study termination.**
DESCRIPTION OF CHANGE:
Update the language in Sections 5.1.4 Criteria for Continuation of Treatment and 6.3 Discontinuation of Study to allow potential availability of zolbetuximab to subjects who are still receiving and benefitting from it, in cases of premature study termination or following conclusion of the study.
RATIONALE:
The change was made to clarify that zolbetuximab may be made available following conclusion of the study or in case of premature study termination for subjects still benefitting from treatment.

**14. Remove details regarding zolbetuximab concentration and immunogenicity testing.**
DESCRIPTION OF CHANGE:
Delete zolbetuximab quantification details and immunogenicity assessment details from Sections 5.6 and 5.7.4, respectively.
RATIONALE:
The specific details regarding quantification of zolbetuximab and immunogenicity assessment are not necessary to be specified in the protocol.
**15. Clarify the maximum amount of blood to be collected during the study.**
DESCRIPTION OF CHANGE:
Clarify the maximum amount of blood to be collected during cycle 1 as approximately 40 mL, and less in later cycles.
RATIONALE:
Updated to align with current sample collection schedule.
**16. Clarify that the assessment of the relationship between zolbetuximab immunogenicity and zolbetuximab pharmacokinetics, efficacy and safety in subjects may be assessed.**
DESCRIPTION OF CHANGE:
Clarify that the assessment of the relationship between zolbetuximab immunogenicity and zolbetuximab pharmacokinetics, efficacy and safety in subjects may be assessed.
RATIONALE:
To allow flexibility for analysis based upon data availability.

II. Amendment Summary of Changes:

IIA. Substantial Changes

**IV Synopsis, Inclusion Criterion and 3 Study Population**<br>*3.2 Inclusion Criterion*
WAS:
o Either serum creatinine ≤ 1.5 x ULN or estimated glomerular filtration rate ≥ 50 mL/min/1.73m^2
IS AMENDED TO:
o ~~Either serum creatinine ≤ 1.5 x ULN or E~~estimated ~~glomerular filtration rate ≥ 50 mL/min/1.73m^2~~ **creatinine clearance ≥ 30 mL/min**

**IV Synopsis, Exclusion Criterion and 3 Study Population**<br>*3.3 Exclusion Criterion 4*
WAS:
4. Subject has received systemic immunosuppressive therapy, including systemic corticosteroids within 14 days prior to first dose of study treatment. Subject using a physiologic replacement dose of hydrocortisone or its equivalent (defined as up to 30 mg per day of hydrocortisone or up to 10 mg per day of prednisone) is eligible.
IS AMENDED TO:
4. Subject has received systemic immunosuppressive therapy, including systemic corticosteroids within 14 days prior to first dose of study treatment. Subject using a physiologic replacement dose of hydrocortisone or its equivalent (defined as up to 30 mg per day of hydrocortisone or up to 10 mg per day of prednisone) **or a single dose of systemic corticosteroids** is eligible.

**IV Synopsis, Exclusion Criteria and 3 Study Population**<br>*3.3 Exclusion Criterion*
WAS:
6. Subject has prior severe allergic reaction or intolerance to monoclonal antibody, including humanized or chimeric antibodies.
IS AMENDED TO:
6. Subject has prior severe allergic reaction or intolerance to **known ingredients of zolbetuximab or other** monoclonal antibod~~y~~**ies**, including humanized or chimeric antibodies.

**IV Synopsis, Exclusion Criteria and 3 Study Population**<br>*3.3 Inclusion Criterion*
WAS:

9. Subject has known dihydropyrimidine dehydrogenase (DPD) deficiency. (NOTE: Subjects with low or absent DPD should not be treated with capecitabine due to increased risk for severe, life-threatening, or fatal adverse reactions.)

IS AMENDED TO:

9. Subject has known dihydropyrimidine dehydrogenase (DPD) deficiency. (NOTE: **Screening for DPD deficiency should be conducted per local requirements.** ~~Subjects with low or absent DPD should not be treated with capecitabine due to increased risk for severe, life-threatening, or fatal adverse reactions.~~)

IV Synopsis, Exclusion Criteria and 3 Study Population
3.3 Exclusion Criterion #15

ADDED:

d) History or family history of congenital long QT syndrome

IV Synopsis, Exclusion Criteria and 3 Study Population
3.3 Exclusion Criterion

WAS:

18. Subject has had a major surgical procedure and has not completely recovered within 28 days prior to the first dose of study treatment.

IS AMENDED TO:

18. Subject has had a major surgical procedure ~~and has not completely recovered within~~≤ 28 days prior to the first dose of study treatment.
    a) **Subject is without complete recovery from a major surgical procedure ≤ 14 days prior to the first dose of study treatment.**

IV Synopsis, Other Products; Sections 2 Study Objective(s), Design, and Endpoints; and 5 Treatments and Evaluation
Sections 2.2.1 Study Design, 5.1.1.3 CAPOX and 5.1.2.6 CAPOX: Dose Modification for Non-Hematologic Toxicity

ADDED:

(NOTE: ECG is required to be performed and assessed locally prior to every oxaliplatin infusion [before any antiemetic treatment] and following completion of every oxaliplatin infusion. Ensure that potassium, magnesium and calcium levels are within normal limits prior to oxaliplatin infusion.)

IV Synopsis, Other Products and Section 5 Treatments and Evaluation
Section 5.1.1.2 Antiemetics

WAS:

*To minimize the risk of Torsades de Pointes, avoid use in subjects with congenital long QT syndrome and administer with caution to subjects who have or may develop QTc prolongation.

IS AMENDED TO:

*To minimize the risk of Torsades de Pointes, ~~avoid use in subjects with congenital long QT syndrome and~~ administer with caution to subjects who have or may develop QTc prolongation.

IV Synopsis, Concomitant Medication Restrictions or Requirements; Section 5 Treatments and Evaluation; and Appendices

Section 5.1.5 Previous and Concomitant Treatment (Medication and Non-Medication Therapy)

Section 12.4 Concomitant Medication Restrictions or Requirements

WAS:

All medications and concomitant treatments administered from 28 days prior to day 1 through the 90-day safety follow-up visit must be recorded in the eCRF. Documentation will include the medication name, indication, route and dates of administration.

Prohibited Concomitant Treatment

The following are strictly prohibited:

- Sorivudine or analogs (during capecitabine treatment)
- Systemic immunosuppressive agents:
  - Concurrent systemic immunosuppressive therapy, in particular systemic corticosteroids, should be stopped 14 days prior to first dose of study treatment.
  - Subjects are allowed to use a physiologic replacement dose of hydrocortisone or its equivalent (defined as up to 30 mg per day of hydrocortisone or up to 10 mg per day of prednisone).
- Live vaccines should be avoided during the treatment period in which subject is receiving capecitabine and up to 6 months after final capecitabine dose.
- Other systemic chemotherapy, immunotherapy, radiotherapy, herbal medications or other treatments intended for antitumor activity. Palliative radiotherapy for peripheral bone metastases is allowed.
- Investigational products or therapy other than IMAB362.

Cautionary Concomitant Treatment

Considerations should be given to avoid or minimize the use of the following concomitant medications, if possible, during IMAB362/placebo treatment:

f) Systemic corticosteroids, because their impact on the potential efficacy of IMAB362 is not known.
   - Systemic corticosteroids should be avoided or minimized while subject is on study treatment unless required for management of an emergent medical condition (e.g., severe nausea/vomiting or hypersensitivity reaction).
   - For a subject's first dose of IMAB362/placebo, it is recommended that the prophylactic use of corticosteroids be avoided.
   - Inhaled, intranasal and topically applied steroids are allowed.

g) Avoid the class of 5-HT3 blockers in subjects with congenital long QT syndrome. Administer these drugs with caution in subjects who have or may develop QTc prolongation.

h) Nonsteroidal anti-inflammatory drugs (NSAIDs) because of the potential to cause gastric ulcers and covert bleeding.

   a. In such cases where NSAID use is necessary, the use of NSAIDs with lower gastric ulcerogenic potential is preferred and concomitant gastric protection with proton pump inhibitors is recommended.

The following should be avoided or used with caution and closely monitored during CAPOX administration:

i) CytochromeP450 (CYP) 2C9 substrates (Subjects taking coumarin-derivative anticoagulants concomitantly with capecitabine should have PT/INR monitored regularly and anticoagulant dose adjusted accordingly).

j) Anti-epileptic medications (e.g. phenobarbital, phenytoin and primidone)

IS AMENDED TO:

All medications and concomitant treatments administered from 28 days prior to day 1 through the 90-day safety follow-up visit must be recorded in the eCRF. Documentation will include the medication name, indication, route and dates of administration.

Prohibited Concomitant Treatment

The following are strictly prohibited:

- Sorivudine or analogs (during capecitabine treatment)
- Systemic immunosuppressive agents:
  - Concurrent systemic immunosuppressive therapy, in particular systemic corticosteroids, should be stopped 14 days prior to first dose of study treatment.
  - Subjects are allowed to use a physiologic replacement dose of hydrocortisone or its equivalent (defined as up to 30 mg per day of hydrocortisone or up to 10 mg per day of prednisone) **or a single dose of systemic corticosteroids**.
- Live vaccines should be avoided during the treatment period in which subject is receiving capecitabine and up to 6 months after final capecitabine dose.
- Other systemic chemotherapy, immunotherapy, radiotherapy, herbal medications or other treatments intended for antitumor activity. Palliative radiotherapy for peripheral bone metastases is allowed.
- Investigational products or therapy other than **zolbetuximab**.

Cautionary Concomitant Treatment

Considerations should be given to avoid or minimize the use of the following concomitant medications, if possible, during **zolbetuximab**/placebo treatment:

k) Systemic corticosteroids, because their impact on the potential efficacy of **zolbetuximab** is not known.

   a. Systemic corticosteroids should be avoided or minimized while subject is on study treatment unless required for management of an emergent medical condition (e.g., severe nausea/vomiting or hypersensitivity reaction).

   b. For a subject's first dose of **zolbetuximab**/placebo, it is recommended that the prophylactic use of corticosteroids be avoided.

   c. Inhaled, intranasal and topically applied steroids are allowed.

l) Avoid the class of 5-HT3 blockers ~~in subjects with congenital long QT syndrome~~.

Administer these drugs with caution in subjects who have or may develop QTc prolongation.

m) Nonsteroidal anti-inflammatory drugs (NSAIDs) because of the potential to cause gastric ulcers and covert bleeding.

   a. In such cases where NSAID use is necessary, the use of NSAIDs with lower gastric ulcerogenic potential is preferred and concomitant gastric protection with proton pump inhibitors is recommended.

The following should be avoided or used with caution and closely monitored during CAPOX administration:

n) CytochromeP450 (CYP) 2C9 substrates (Subjects taking coumarin-derivative anticoagulants concomitantly with capecitabine should have PT/INR monitored regularly and anticoagulant dose adjusted accordingly).

o) Anti-epileptic medications (e.g. phenobarbital, phenytoin and primidone)

p) Medications known to prolong the QT or QTc interval (refer to https://www.crediblemeds.org for a list of these medications)

Prohibited and cautionary concomitant treatments are described in Appendix 12.4.

**IV Synopsis, Study Treatment Discontinuation Criteria and Section 6 Discontinuation**<br>*Section 6.1 Discontinuation of Individual Subject(s) from Study Treatment*
WAS:
• Subject has a delay of study treatment (IMAB362/placebo and both components CAPOX) for > 28 days.
IS AMENDED TO:
• Subject has a delay of study treatment (**zolbetuximab**/placebo and both components CAPOX) for > 28 days **from when the next study treatment was scheduled to be administered**.

IV Synopsis Table 1, Schedule of Assessments

WAS:

VISIT	Screening[1]	Study Treatment Period (Each Cycle = 21 Days)						Follow-up Period				
		**Cycles 1-8** IMAB362/Placebo + CAPOX			**Cycle 9+** IMAB362/Placebo + Capecitabine (at investigator discretion)			Study Treatment Discontinuation Visit[18]	30-Day Safety Follow-up Visit(s)[19]	90-Day Safety Follow-up Visit(s)[20]	Post-treatment Follow-up Period [21]	Long-term and Survival Follow-up Periods [22]
**Day**		**1**	**2-14**	**15-21**	**1**	**2-14**	**15-21**					
**Visit Window (calendar days)**	**-45 to -1**	**+7***	**(no visit)**	**(no visit)**	**+7**	**(no visit)**	**(no visit)**	**+7**	**+7**	**±7**	**±7**	**±14**
**…**												
**Safety Assessments**												
Physical Examination[9]	X	X			X			X	X			
Weight[9]	X	X			X			X	X			
Vital Signs[10]	X	X			X			X	X			
ECOG Performance Status[9]	X	X			X			X				
12-lead ECG[11]	X		If clinically indicated and/or per local requirements					X	X			
**Image Assessment[12]**	X[1]	Every 9 weeks ±7 days for first 54 weeks and then every 12 weeks ±7 days thereafter										
**Laboratory Tests**												
Biochemistry[13]	X	X			X			X	X			
TSH and T4[13]	X	If clinically indicated						X				
Hematology[13]	X	X			X			X	X			
Coagulation Parameters (PT, PTT and INR)[14]	X	If clinically indicated										
Urinalysis[13]	X	X			X			X	X			
Serum Pregnancy Test[15]	X	If clinically indicated and/or per local requirements										
Urine Pregnancy Test[16]		X			X			X	X			
**…**												
Concomitant Medication[29]	X	X			X			X	X			

VISIT	Screening[1]	Study Treatment Period (Each Cycle = 21 Days)<br>Cycles 1-8<br>IMAB362/Placebo + CAPOX			Cycle 9+<br>IMAB362/Placebo + Capecitabine (at investigator discretion)			Follow-up Period<br>Study Treatment Discontinuation Visit[18]	30-Day Safety Follow-up Visit(s)[19]	90-Day Safety Follow-up Visit(s)[20]	Post-treatment Follow-up Period [21]	Long-term and Survival Follow-up Periods [22]
**Day**		**1**	**2-14**	**15-21**	**1**	**2-14**	**15-21**					
**Visit Window (calendar days)**	**-45 to -1**	**+7***	**(no visit)**	**(no visit)**	**+7**	**(no visit)**	**(no visit)**	**+7**	**+7**	**±7**	**±7**	**±14**
AE	X	X			X			X	X	X		

IS AMENDED TO:

		Cycles 1-8<br>Zolbetuximab/Placebo + CAPOX			Cycle 9+<br>Zolbetuximab/Placebo + Capecitabine (at investigator discretion)			Study Treatment Discontinuation Visit[18]	30-Day Safety Follow-up Visit(s)[19]	90-Day Safety Follow-up Visit(s)[20]	Post-treatment Follow-up Period [21]	Long-term and Survival Follow-up Periods [22]
**Day**		**1**	**2-14**	**15-21**	**1**	**2-14**	**15-21**					
**Visit Window (calendar days)**	**-45 to -1**	**+7***	**(no visit)**	**(no visit)**	**+7**	**(no visit)**	**(no visit)**	**+7**	**+7**	**±7**	**±7**	**±14**
**…**												
**Safety Assessments**												
Physical Examination[9]	X	X			X			X	X			
Weight[9]	X	X			X			X	X			
Vital Signs[10]	X	X			X			X	X			

		**Cycles 1-8**<br>**Zolbetuximab/Placebo**<br>**+**<br>**CAPOX**			**Cycle 9+**<br>**Zolbetuximab/Placebo**<br>**+**<br>**Capecitabine**<br>(at investigator discretion)			**Study Treatment Discontinuation Visit**[18]	**30-Day Safety Follow-up Visit(s)**[19]	**90-Day Safety Follow-up Visit(s)**[20]	**Post-treatment Follow-up Period**[21]	**Long-term and Survival Follow-up Periods**[22]
**Day**		**1**	**2-14**	**15-21**	**1**	**2-14**	**15-21**					
**Visit Window (calendar days)**	**-45 to -1**	**+7***	**(no visit)**	**(no visit)**	**+7**	**(no visit)**	**(no visit)**	**+7**	**+7**	**±7**	**±7**	**±14**
ECOG Performance Status[9]	X	X			X			X				
12-lead ECG[11]	X	**X**	If clinically indicated and/or per local requirements					X	X			
**Image Assessment**[12]	X[1]	Every 9 weeks ±7 days **from C1D1** for **the** first 54 weeks and then every 12 weeks ±7 days thereafter										
**Laboratory Tests**												
Biochemistry[13]	X	X			X			X	X			
TSH and T4[13]	X	If clinically indicated						X				
Hematology[13]	X	X			X			X	X			
Coagulation Parameters (PT, PTT and INR)[14]	X	If clinically indicated										
Urinalysis[13]	X	X			X			X	X			
**DPD testing per local requirements**	**X**											
**Cytokine/Chemokine and/or Tryptase**		**If clinically indicated**										
Serum Pregnancy Test[15]	X	If clinically indicated and/or per local requirements										
Urine Pregnancy Test[16]		X			X			X	X			
…												
Concomitant Medication[29]	X	X			X			X	X	**X**		
**AE/SAE**[30]	X	X			X			X	X	X		

IV Synopsis Table 1, Schedule of Assessments Footnotes

WAS:

4. Randomization: After confirmation of eligibility, the unblinded pharmacist/designee will contact the interactive response technology (IRT) system ~~in~~ order to determine the randomly assigned treatment. Randomization may be performed prior to C1D1; however, the time between the beginning of IMAB362/placebo reconstitution and the beginning of IMAB362/placebo infusion must be within 4 hours at controlled room temperature (15°C to 25°C). If the infusion cannot be initiated with the 4 hours, the infusion bag must be stored at 2°C to 8°C and infusion must be initiated with 24 hours from the beginning of reconstitution.

IS AMENDED TO:

4. Randomization: After confirmation of eligibility, the **blinded site user will perform the randomization IRT transaction. The** unblinded pharmacist/designee will ~~contact~~**be notified by** the ~~interactive response technology (~~IRT~~)~~ system ~~in order to determine~~**about** the randomly assigned treatment. Randomization may be performed prior to C1D1~~; however, the time between the beginning of IMAB362/placebo reconstitution and the beginning of IMAB362/placebo infusion must be within 4 hours at controlled room temperature (15°C to 25°C). If the infusion cannot be initiated with the 4 hours, the infusion bag must be stored at 2°C to 8°C and infusion must be initiated with 24 hours from the beginning of reconstitution.~~. **Details of infusion preparation and storage requirements are provided in the Pharmacy Manual.**

WAS:

11. ECGs: a single ECG will be performed at Screening, the IMAB362/placebo Discontinuation Visit, the 30-Day Follow-up Visit, and if clinically indicated or per local requirements. ECGs will be locally assessed. When collected on the same day, ECG should be collected prior to pharmacokinetic samples. For further details, see [Section 5.4.5 Electrocardiogram].

IS AMENDED TO:

11. ECGs: ~~a single ECG will also be performed during the treatment period on each day in which oxaliplatin is administered (prior to any antiemetic treatment), and at the time of the IMAB362/placebo Discontinuation Visit, the 30-Day Follow-up Visit, and if clinically indicated or per local requirements.~~ ECGs will be locally assessed. When collected on the same day, ECG should be collected prior to pharmacokinetic samples. For further details, see [Section 5.4.5 Electrocardiogram]. **A single ECG will be performed at the following time points:**
    - **Screening**
    - **Prior to every oxaliplatin infusion (before any antiemetic treatment administration)**
    - **Following completion of every oxaliplatin infusion**
    - **Zolbetuximab/Placebo Discontinuation Visit**
    - **30-day Follow-up Visit**
    - **If clinical indicated or per local requirements**

WAS:

13. Laboratory Assessments: See [Section 5.4.3 Laboratory Assessments] for list of laboratory assessments. Laboratory tests must be sent to the central laboratory for analysis. Central laboratory results must be used to confirm eligibility. The screening labs used to determine eligibility should be collected within 14 days prior to C1D1. In situations where central laboratory results are outside of the permitted range, the investigator may opt to retest the subject and subsequent within range screening results may be used to confirm eligibility. In case of multiple laboratory data within the Screening period, the most recent central laboratory data should be used to confirm eligibility. Subjects requiring transfusions to meet eligibility criteria are not eligible. Laboratory tests will be reviewed by the investigator prior to each infusion. In the event that the central laboratory results are not available in time for treatment decisions, local certified laboratory tests may be used. Holidays and weekends should be taken into account when scheduling these blood draws. Additional assessments may be done centrally or locally to monitor AEs or as clinically indicated. Clinical significance of out-of-range laboratory findings is to be determined and documented by the investigator/sub-investigator who is a qualified physician.

IS AMENDED TO:

13. Laboratory Assessments: See [Section 5.4.3 Laboratory Assessments] for list of laboratory assessments. Laboratory tests must be sent to the central laboratory for analysis. Central laboratory results must be used to confirm eligibility. The screening labs used to determine eligibility should be collected within 14 days prior to C1D1. In situations where central laboratory results are outside of the permitted range, the investigator may opt to retest the subject and subsequent within range screening results may be used to confirm eligibility. In case of multiple laboratory data within the Screening period, the most recent central laboratory data should be used to confirm eligibility. Subjects requiring transfusions to meet eligibility criteria are not eligible. Laboratory test~~s~~ **results** will be reviewed by the investigator prior to ~~each infusion~~**any study treatment**. ~~In the event that the central laboratory results are not available in time for treatment decisions, local certified laboratory tests may be used.~~ **Local laboratory results may be used for treatment decisions; however, central laboratory samples must also be drawn per protocol and sent to the central laboratory.** Holidays and weekends should be taken into account when scheduling these blood draws. Additional assessments may be done centrally or locally to monitor AEs or as clinically indicated. Clinical significance of out-of-range laboratory findings is to be determined and documented by the investigator/sub-investigator who is a qualified physician.

WAS:

19. 30-Day Safety Follow-up Visit: A 30-Day Safety Follow-up Visit should occur 30 days after the last dose of IMAB362/placebo and will include the assessments as shown the in the Schedule of Assessments above. A 30-Day Safety Follow-up Visit should occur 30 days after the last dose of CAPOX (both components) and may be conducted by phone if

the subject is unable to visit the site and will require contact for AE collection only.

20. <u>90-Day Safety Follow-up Visits</u>: A 90-Day Safety Follow-up Visit should occur 90 days after the last dose of IMAB362/placebo and will include the assessments as shown the in the Schedule of Assessments above. A 90-Day Safety Follow-up Visit should occur 90 days after the last dose of CAPOX (both components) and may be conducted by phone if the subject is unable to visit the site and will require contact for SAE collection only.

IS AMENDED TO:

19. <u>30-Day Safety Follow-up Visit</u>: A 30-Day Safety Follow-up Visit should occur 30 days after the last dose of **Zolbetuximab**/Placebo and will include the assessments as shown the in the Schedule of Assessments above. A 30-Day Safety Follow-up Visit should occur 30 days after the last dose of CAPOX (both components) and may be conducted by phone if the subject is unable to visit the site and will require contact for AE/**SAE** collection only.
20. <u>90-Day Safety Follow-up Visits</u>: A 90-Day Safety Follow-up Visit should occur 90 days after the last dose of **Zolbetuximab**/placebo and will include the assessments as shown the in the Schedule of Assessments above. A 90-Day Safety Follow-up Visit should occur 90 days after the last dose of CAPOX (both components) and may be conducted by phone if the subject is unable to visit the site and will require contact for **AE**/SAE collection only.

WAS:

25. Genetic Immune Polymorphism: Whole blood sample taken at C1D1.

IS AMENDED TO:

25. Genetic Immune Polymorphism: **To be collected per local policy.** Whole blood sample taken at C1D1.

WAS:

29. <u>AEs</u>: AEs will be collected from the time of informed consent through 30 days following the last dose of study treatment or until initiation of a new anticancer treatment, whichever comes first. See [Section 5.5.5 Reporting of Serious Adverse Events].

IS AMENDED TO:

29. **Concomitant medications will be collected from the time of informed consent through 90 days following the last dose of study treatment.**
30. <u>AEs/**SAEs**</u>: AEs **and SAEs (regardless of causality)** will be collected from the time of informed consent through ~~30~~**90** days following the last dose of study treatment.~~ or until initiation of a new anticancer treatment, whichever comes first~~. ~~SAEs (regardless of causality) will be collected from the time of informed consent through 90 days following the last dose of study treatment or until initiation of a subsequent anticancer treatment,~~

~~whichever comes first.~~ See [Section 5.5.5 Reporting of Serious Adverse Events].

**Section 1 Introduction**<br>*Section 1.3 Summary of Key Safety Information for Study Drugs*
WAS:
Potential IMAB362 toxicities, based on nonclinical studies, and important identified risks along with important potential risks, based on observations from the clinical studies, are described in Section 5.2.3 of the Investigator's Brochure (IB). Expected adverse drug reactions, including reference safety information (RSI) used for expedited health authority reporting are described in Section 5.2.4 of the IB.
IS AMENDED TO:
Potential **Zolbetuximab** toxicities, based on nonclinical studies, and important identified risks along with important potential risks, based on observations from the clinical studies, are described in Section 5.2~~.3~~ of the Investigator's Brochure (IB). Expected adverse drug reactions, including reference safety information (RSI) used for expedited health authority reporting are described in Section 5.2.**3**4 of the IB.

**Section 1 Introduction**<br>*Section 1.5 Risk Benefit Assessment*
WAS:
Overall, the potential benefits of IMAB362 in combination with CAPOX outweigh the risks and the available nonclinical and clinical data support further clinical development for subjects with CL DN18.2-positive, HER2 negative locally advanced unresectable or metastatic gastric or GEJ adenocarcinoma who meet protocol eligibility criteria.
IS AMENDED TO:
Overall, the potential benefits of **Zolbetuximab** in combination with CAPOX outweigh the **identified and potential** risks ~~and the available nonclinical and clinical data support~~ further **allowing for** further ~~clinical~~ development ~~for~~**in patients** with CL DN18.2-positive, HER2 negative locally advanced unresectable or metastatic gastric or GEJ adenocarcinoma~~ who meet protocol eligibility criteria~~.

**Section 5 Treatments and Evaluation**<br>*Section 5.1.2.2 IMAB362/Placebo Interruption or Premanent Discontinuation*
WAS:
Permanently discontinue IMAB362/placebo treatment if delayed beyond 28 days from the last administered dose due to toxicity of IMAB362/placebo.
IS AMENDED TO:
~~Permanently discontinue IMAB362/placebo treatment if delayed beyond 28 days from the last administered dose due to toxicity of IMAB362/placebo.~~ **A delay of**

zolbetuximab/placebo treatment for > 28 days from when the next zolbetuximab/placebo treatment was scheduled to be administered due to unresolved toxicity associated with zolbetuximab/placebo will result in the subject discontinuing zolbetuximab/placebo.

<u>Section 5.1.2.4 CAPOX Dose Modification</u>

WAS:

In subjects experiencing toxicity requiring a delay or discontinuation of CAPOX, subject should continue to receive IMAB362/placebo as clinically appropriate. If CAPOX is interrupted, subject should be evaluated weekly (at a minimum) until the toxicity has improved sufficiently at which time treatment can be restarted as described in the tables below (as applicable). Unresolved toxicity > 28 days associated with CAPOX will result in the subject discontinuing CAPOX (both components).

IS AMENDED TO:

In subjects experiencing toxicity requiring a delay or discontinuation of CAPOX, subject should continue to receive **zolbetuximab**/placebo as clinically appropriate. If CAPOX is interrupted, subject should be evaluated weekly (at a minimum) until the toxicity has improved sufficiently at which time treatment can be restarted as described in the tables below (as applicable). **A delay of CAPOX treatment for > 28 days from when the next CAPOX treatment was scheduled to be administered due to** ~~U~~**u**nresolved toxicity ~~> 28 days~~ associated with CAPOX will result in the subject discontinuing CAPOX (both components).

<u>Section 5.1.2.5 CAPOX Dose Modification for Hematologic Toxicity</u>

WAS:

The CAPOX dose modifications for hematologic toxicity are presented in Table 6. Dose modifications should be maintained until recovery from hematologic toxicity. CAPOX should be permanently stopped in subjects not recovering from hematologic toxicity in 28 days.

IS AMENDED TO:

The CAPOX dose modifications for hematologic toxicity are prese nted in Table 6. Dose modifications should be maintained until recovery from hematologic toxicity. ~~CAPOX should be permanently stopped in subjects not recovering from hematologic toxicity in 28 days.~~ **A delay of CAPOX treatment for > 28 days from when the next CAPOX treatment was scheduled to be administered due to hematologic toxicity associated with CAPOX will result in the subject discontinuing CAPOX (both components).**

Section 5.1.2.6 CAPOX: Dose Modification for Non-Hematologic Toxicity
WAS:
CAPOX dose modifications for non-hematologic toxicity should be based on the most severe toxicity experienced during the last treatment (Table 7). Retreatment should be delayed until recovery of all non-hematologic toxicity to ≤ Grade 2 with the exception of increased bilirubin or ALT, which must recover to Grade 1 or baseline, whichever was higher. The maximum permitted treatment delay is 28 days for recovery of non-hematologic toxicity. If after a > 28-day treatment delay, the subject has not recovered sufficiently to meet retreatment criteria, CAPOX should be discontinued.
IS AMENDED TO:
CAPOX dose modifications for non-hematologic toxicity should be based on the most severe toxicity experienced during the last treatment (Table 7). Retreatment should be delayed until recovery of all non-hematologic toxicity to ≤ Grade 2 with the exception of increased bilirubin or ALT, which must recover to Grade 1 or baseline, whichever was higher. The maximum permitted treatment delay is 28 days **from when the next study treatment was scheduled to be administered** for recovery of non-hematologic toxicity. If after a > 28-day treatment delay, the subject has not recovered sufficiently to meet retreatment criteria, CAPOX should be discontinued.
Section 5.1.2.6 CAPOX: Dose Modification for Non-Hematologic Toxicity
ADDED:
**During or following study treatment, additional ECG monitoring should be initiated per local standard of care for subjects who experience syncope, presyncope, palpitations and/or bradycardia.**<br>• **If the QTc interval is > 450 msec, medically manage per local standard of care, including correction of hypokalemia, hypomagnesemia, and/or hypocalcemia.**<br>• **If the QTc interval is > 500 msec, medically manage per local standard of care, withhold capecitabine and oxaliplatin treatment, ensure appropriate (continuous) ECG monitoring, and obtain cardiology consultation. If QTc interval resolves to ≤ 450 msec, the subject may resume treatment at 1 reduced dose level.**

**Section 5 Treatments and Evaluation**<br>*Section 5.4.3 Laboratory Assessments*	
ADDED:	
Biochemistry	**Creatinine Clearance**
**Grade 3 or 4 Infusion-related Reactions (IRR)**	**Cytokine/Chemokine Panel†**
**Any reaction with features of anaphylaxis**	**Serum total tryptase†**
**Dihydropyrimidine dehydrogenase (DPD)**	**DPD deficiency alleles**

**deficiency screening†**	
**Urine Pregnancy Test**	**Human Chorionic gonadotropin (HCG)**
**† As applicable**	
WAS:	
Urinalysis	Urobilirubin
IS AMENDED TO:	
Urinalysis	~~Urobilirubin~~**Urobilinogen**

**Section 5 Treatments and Evaluation**<br>*Sections 5.5 Adverse Events, Other Safety Aspects, 5.5.1 3 Disease Progression and Study Endpoints*
WAS:
AE collection will begin from time of informed consent and continue through the 30 days following the last dose of IMAB362/placebo and CAPOX (bothcomponents), or until initiation of a subsequent anticancer therapy.<br>Serious adverse events (SAEs), regardless of causality will be collected from the time of informed consent through 90 days following the last dose of IMAB362/placebo and CAPOX (both components), or until initiation of a subsequent anticancer therapy, whichever comes first.
IS AMENDED TO:
AE collection will begin from time of informed consent and continue through the ~~30~~**90** days following the last dose of **zolbetuximab**/placebo and CAPOX (both components)~~, or until initiation of a subsequent anticancer therapy~~.<br>Serious adverse events (SAEs), regardless of causality will be collected from the time of informed consent through 90 days following the last dose of **zolbetuximab**/placebo and CAPOX (both components)~~, or until initiation of a subsequent anticancer therapy, whichever comes first~~.
ADDED:
**All deaths up to 90 days after the last dose of study drug must be reported as an SAE, even if attributed to disease progression.**

IIB. Non-Substantial Changes

**Section II Contact Details of Key Sponsor's Personnel**<br>*Clinical Research Contacts Japan*
WAS:
Corporate Name: Astellas Pharma Inc.<br>Location: 2-5-1, Nihonbashi-Honcho, Chuo-ku, Tokyo<br>PPD [redacted]<br>[redacted]
IS AMENDED TO:
Corporate Name: Astellas Pharma Inc.<br>Location: 2-5-1, Nihonbashi-Honcho, Chuo-ku, Tokyo<br>PPD [redacted]<br>PPD [redacted]

**IV Synopsis, Title of Study**
WAS:
A Phase 3, Global, Multi-Center, Double-Blind, Randomized, Efficacy Study of IMAB362 Plus CAPOX Compared with Placebo Plus CAPOX as First-line Treatment of Subjects with Claudin (CLDN)18.2-Positive, HER2-Negative, Locally Advanced Unresectable or Metastatic Gastric or Gastroesophageal Junction (GEJ) Adenocarcinoma
IS AMENDED TO:
A Phase 3, Global, Multi-Center, Double-Blind, Randomized, Efficacy Study of **Zolbetuximab** (IMAB362) Plus CAPOX Compared with Placebo Plus CAPOX as First-line Treatment of Subjects with Claudin (CLDN)18.2-Positive, HER2-Negative, Locally Advanced Unresectable or Metastatic Gastric or Gastroesophageal Junction (GEJ) Adenocarcinoma

**IV Synopsis, Planned Total Number of Study Centers and Location(s)**
WAS:
Approximately 125 centers globally.
IS AMENDED TO:
Approximately ~~125~~**130** centers globally.

IV Synopsis Study Design Overview and Schedule of Assessments (footnotes), Sections 2 Study Objective(s), Design and Endpoints
<u>Section 2.2.1 Study Design</u>

WAS:

Formalin fixed paraffin embedded (FFPE) tumor tissue will be collected for central testing to determine CLDN18.2 and HER2 status. Confirmation of CLDN18.2-positive and HER2-negative status is to be obtained prior to subjects proceeding to any other Screening procedures.

IS AMENDED TO:

Formalin fixed paraffin embedded (FFPE) tumor tissue will be collected for central testing to determine CLDN18.2 and HER2 status. ~~Confirmation of CLDN18.2-positive and HER2-negative status is to be obtained prior to subjects proceeding to any other Screening procedures.~~

IV Synopsis Table 1, Schedule of Assessments (Definitions of Abbreviations Footnote)

WAS:

ECOG: European Cooperative Oncology Group;

IS AMENDED TO:

ECOG: ~~European~~ **Eastern** Cooperative Oncology Group;

IV Synopsis Pharmacokinetics and Section 7 Statistical Methods
<u>Section 7.6.2 Immunogenicity</u>

WAS:

Immunogenicity of IMAB362 will be summarized using the frequency of ADA positive subjects. The potential relationship between IMAB362 immunogenicity and IMAB362 pharmacokinetics, efficacy, safety profile in subjects will be assessed.

Additional model-based analyses may be performed and reported separately for IMAB362 pharmacokinetics.

IS AMENDED TO:

Immunogenicity of **zolbetuximab** will be summarized using the frequency of ADA positive subjects. The potential relationship between **zolbetuximab** immunogenicity and **zolbetuximab** pharmacokinetics, efficacy, safety profile in subjects **may**~~will~~ be assessed.

Additional model-based analyses may be performed and reported separately for **zolbetuximab** pharmacokinetics.

**Section 4 Identification of Study Treatment(s)**<br>*Section 4.1 IMAB362 (Investigational Product)*
DELETED:
~~The investigational product has to be reconstituted with 5.0 mL water for injection to a concentration of 20 mg/mL. Further dilution in an IV bag with sterile 0.9% sodium chloride to a final concentration of 2 mg/mL is required. The time between the beginning of reconstitution and the beginning of infusion must be within 4 hours at controlled room temperature (15°C to 25°C). If the infusion cannot be initiated within the 4 hours, the infusion bag must be stored at 2°C to 8°C and infusion must be initiated within 24 hours from the beginning of reconstitution.~~

**Section 4 Identification of Study Treatment(s)**<br>*Section 4.2 Comparative Drug (Placebo)*
ADDED:
**Details of preparation of placebo are provided in the Pharmacy Manual.**

**Section 4 Identification of Study Treatment(s)**<br>*Section 4.7 Assignment and Allocation*
WAS:
Subject randomization will be performed via IRT and treatment assigned in a 1:1 ratio to IMAB362 or placebo. Prior to the initiation of the study treatment, the unblinded pharmacist/designee will contact the IRT system in order to determine the randomly assigned treatment. Specific procedures for randomization through the IRT are contained in the IRT manual.
IS AMENDED TO:
Subject randomization will be performed **by the blinded site user** via IRT and treatment assigned in a 1:1 ratio to **zolbetuximab** or placebo. Prior to the initiation of the study treatment, the unblinded pharmacist/designee will ~~contact~~**be notified by** the IRT system ~~in order to determine~~**about** the randomly assigned treatment. Specific procedures for randomization through the IRT are contained in the IRT manual.

**Section 5 Treatments and Evaluation**<br>*Section 5.1.4 Criteria for Continuation of Treatment*
WAS:
IMAB362 may be made available after conclusion of the study to subjects who are still receiving and benefitting from study treatment until study defined treatment discontinuation criterion is met in countries where the drug does not have marketing approval nor is commercially available.

IS AMENDED TO:
**Zolbetuximab** may be made available after conclusion of the study to subjects who are still receiving and benefitting from study treatment until **a** study**-**defined treatment discontinuation criterion is met~~ in countries where the drug does not have marketing approval nor is commercially available~~.

**Section 5 Treatments and Evaluation**<br>*Section 5.4.3*
WAS:
Laboratory tests will be reviewed by the investigator prior to each infusion. In the event that the central laboratory results are not available in time for treatment decisions, local certified laboratory tests may be used.
IS AMENDED TO:
Laboratory tests will be reviewed by the investigator prior to each infusion. ~~In the event that the central laboratory results are not available in time for treatment decisions, local certified laboratory tests may be used.~~ **Local laboratory results may be used for treatment decisions; however, central laboratory samples must also be drawn per protocol and sent to the central laboratory.**

**Section 5 Treatments and Evaluation**<br>*Section 5.5.1.2 Potential Cases of Drug-Induced Liver Injury*
WAS:
Refer to [Appendix 12.4 Liver Safety Monitoring and Assessment] for detailed instructions on drug induced liver injury. Abnormal values in AST and/or ALT concurrent or with abnormal elevations in total bilirubin that meet the criteria outlined in [Appendix 12.4 Liver Safety Monitoring and Assessment] in the absence of other causes of liver injury, are considered potential cases of drug-induced liver injury (potential Hy's Law cases) and are always to be considered important medical events and reported per [Section 5.5.5 Reporting of Serious Adverse Events].
IS AMENDED TO:
Refer to [Appendix ~~12.4~~**12.5** Liver Safety Monitoring and Assessment] for detailed instructions on drug induced liver injury. Abnormal values in AST and/or ALT concurrent or with abnormal elevations in total bilirubin that meet the criteria outlined in [Appendix ~~12.4~~**12.5** Liver Safety Monitoring and Assessment] in the absence of other causes of liver injury, are considered potential cases of drug-induced liver injury (potential Hy's Law cases) and are always to be considered important medical events and reported per [Section 5.5.5 Reporting of Serious Adverse Events].

**Section 5 Treatments and Evaluation**<br>*Section 5.6 Testing Drug Concentration*
DELETED:
~~IMAB362 in serum will be quantified using a ligand binding assay with electrochemiluminescense (ECL) detection.~~
**Section 5 Treatments and Evaluation**<br>*Section 5.7.4 Immunogenicity Assessment (Anti-drug Antibody)*
DELETED:
~~ADAs against IMAB362 in serum will be detected using a ligand binding assay with ECI detection. A tiered approach will be followed for screening, confirming and titering the samples.~~

**Section 5 Treatments and Evaluation**<br>*Section 5.8 Total Amount of Blood*
WAS:
The maximum amount of blood collected is approximately 88 mL in cycle 1.
IS AMENDED TO:
The maximum amount of blood collected is approximately ~~88~~**40** mL in cycle 1**, and less in later cycles**.

**Section 6 Discontinuation**<br>*Section 6.3 Discontinuation of the Study*
ADDED:
**In case of premature study termination, zolbetuximab may be made available to subjects who are still receiving and benefitting from study treatment until study defined treatment discontinuation criterion is met.**

14 COORDINATING INVESTIGATOR'S SIGNATURE

A Phase 3, Global, Multi-Center, Double-Blind, Randomized, Efficacy Study of Zolbetuximab (IMAB362) Plus CAPOX Compared with Placebo Plus CAPOX as First-line Treatment of Subjects with Claudin (CLDN)18.2-Positive, HER2-Negative, Locally Advanced Unresectable or Metastatic Gastric or Gastroesophageal Junction (GEJ) Adenocarcinoma

ISN/Protocol 8951-CL-0302

Version 2.0

29 June 2018

I have read all pages of this clinical study protocol for which Astellas is the sponsor. I agree that it contains all the information required to conduct this study.<br>**Coordinating Investigator:**
Signature: ------------------------------------ ------------------------<br>*<Insert name, department/affiliation, name of institution>* Date (DD Mmm YYYY)<br><br>Printed Name: ------------------------------------<br><br>Address: ------------------------------------<br>------------------------------------

15 SPONSOR'S SIGNATURES

STATISTICAL ANALYSIS PLAN

Final Version 1.0, 04-Oct-2018

A Phase 3, Global, Multi-Center, Double-Blind, Randomized, Efficacy Study of Zolbetuximab (IMAB362) Plus CAPOX Compared with Placebo Plus CAPOX as First-line Treatment of Subjects with Claudin (CLDN) 18.2-Positive, HER2-Negative, Locally Advanced Unresectable or Metastatic Gastric or Gastroesophageal Junction (GEJ) Adenocarcinoma

ISN/Protocol 8951-CL-0302

IND 129598

EudraCT 2018-000519-26

Sponsor:

Astellas Pharma Global Development, Inc. (APGD)
1 Astellas Way
Northbrook, IL 60062

This document contains confidential information which is the intellectual property of Astellas. By accepting or reviewing this document, you agree to hold this information in confidence and not copy or disclose it to others or use it for unauthorized purposes except (1) as otherwise agreed to in writing; (2) where required by applicable law; (3) where disclosure is directly related to the care and safety of the research participant; and (4) where disclosure of such information is made to a member of the investigator’s team who agrees to hold this information in confidence.

Table of Contents

I. LIST OF ABBREVIATIONS AND KEY TERMS ······ 5
1 INTRODUCTION ······ 8
2 STUDY OBJECTIVES AND DESIGN ······ 8
2.1 Study Objectives ······ 8
2.1.1 Primary Objective ······ 8
2.1.2 Secondary Objectives ······ 8
2.1.3 Exploratory Objectives ······ 8
2.2 Study Design ······ 9
2.3 Randomization ······ 9
3 SAMPLE SIZE ······ 9
4 ANALYSIS SETS ······ 10
4.1 Full Analysis Set ······ 10
4.2 Safety Analysis Set ······ 10
4.3 Per Protocol Set ······ 10
4.3.1 Reasons for Exclusion from Per Protocol Set ······ 10
4.4 Pharmacokinetics Analysis Set (PKAS) ······ 11
5 ANALYSIS ENDPOINTS ······ 11
5.1 Primary Efficacy Endpoint ······ 11
5.2 Secondary Efficacy Endpoints ······ 11
5.3 Exploratory Efficacy Endpoints ······ 12
5.4 Safety Endpoints ······ 12
5.4.1 AE ······ 12
5.4.2 Clinical Laboratory Variables ······ 13
5.4.3 Vital Signs ······ 13
5.4.4 12-lead electrocardiogram (ECG) ······ 13
5.4.5 ECOG performance score ······ 13
5.4.6 Physical examination ······ 13
5.5 Other Endpoints ······ 13
5.5.1 Pharmacokinetic Endpoints ······ 13
5.5.2 Immunogenicity ······ 13
5.5.3 Biomarkers Endpoints ······ 14
6 STATISTICAL METHODOLOGY ······ 14

6.1 General Considerations 14
6.2 Study Population 15
6.2.1 Disposition of Subjects 15
6.2.2 Protocol Deviations 15
6.2.3 Demographic and Other Baseline Characteristics 16
6.2.4 Transfusions 16
6.2.5 Previous and Concomitant Medications 16
6.2.6 Non-medication Therapies 16
6.2.7 New Anti-Cancer Therapies 16
6.2.8 Prior Radiation Therapy 16
6.2.9 Prior Procedures for Primary Cancer 17
6.2.10 Prior Cancer Chemotherapy 17
6.3 Study Drug Exposure and Compliance 17
6.4 Analysis of Efficacy 18
6.4.1 Analysis of Primary Efficacy Endpoint (PFS) 18
6.4.2 Analysis of Secondary Efficacy Endpoints 20
6.4.3 Analysis of Exploratory Efficacy Endpoints 25
6.5 Analysis of Safety 27
6.5.1 Adverse Events 27
6.5.2 AE of Special interest 29
6.5.3 Clinical Laboratory Evaluation 29
6.5.4 Vital Signs 30
6.5.5 Electrocardiograms 31
6.5.6 ECOG Performance Status 32
6.5.7 Physical Examination 32
6.5.8 Pregnancy 32
6.6 Analysis of Pharmacokinetics 32
6.7 Subgroup Analyses 32
6.8 Other Analyses 32
6.8.1 Immunogenicity 32
6.8.2 Exploratory Biomarkers 33
6.9 Interim Analysis (and Early Discontinuation of the Clinical Study) 33
6.10 Additional Conventions 33
6.10.1 Analysis Visits 33

6.10.2 Imputation Rules for Incomplete Dates 34
7 REVISION AND RATIONALE 34
7.1 List of Changes in SAP Version 2.0 from Version 1.0 (if applicable) 34
8 REFERENCES 34
9 APPENDICES 35
9.1 EORTC QLQ-C30 questionnaire (version 3) 35
9.2 EORTC QLQ-30 Scoring 37
9.3 EORTC QLQ-OG25 questionnaire 38
9.4 EQ-5D-5L Scoring 40
9.5 Global Pain 42
9.6 Health Resource Utilization 42
9.7 Author and Approver Signatories 45

I. LIST OF ABBREVIATIONS AND KEY TERMS

List of Abbreviations

Abbreviations	Description of abbreviations
ADA	anti-drug antibody
AE	adverse event
ALP	alkaline phosphatase
ALT	alanine aminotransferase (GPT)
AST	aspartate aminotransferase (GOT)
ATC	Anatomical Therapeutic Chemical Classification System
BMI	Body mass index
BSA	Body surface area
CAPOX	Capecitabine and Oxaliplatin
C1D1	Cycle 1 Day 1
CI	confidence interval
CLDN	Claudin
C_{trough}	trough concentration
CMH	Cochran-Mantel-Haenszel
CR	complete response
CSR	Clinical study report
CTCAE	Common Terminology Criteria For Adverse Events
DCR	disease control rate
DOR	duration of response
ECG	electrocardiogram
ECOG	Eastern Cooperative Oncology Group
eCRF	electronic case report form
EORTC	European Organization for Research and Treatment of Cancer
EQ-5D-5L	EuroQOL Five Dimensions Questionnaire 5L
FAS	full analysis set
GEJ	gastroesophageal junction
GP	Global Pain
HER2	human epidermal growth factor receptor 2
HR	Hazard ratio
HRQoL	health-related quality of life
HRU	Health Resource Utilization
IDAC	independent data analysis center
IDMC	independent data monitoring committee
INR	international normalized ratio

**Abbreviations**	**Description of abbreviations**
IRC	independent review committee
IRT	interactive response technology
ISN	international study number
IV	Intravenous, intravenously
NCI	National Cancer Institute
NE	not evaluable
ORR	objective response rate
OS	overall survival
PD	progressive disease
PFS	progression free survival
PFS2	progression free survival following second-line anti-cancer treatment
PGx	pharmacogenomics
PKAS	pharmacokinetics analysis set
PPS	per protocol set
PR	partial response
PT	preferred term
QLQ-C30	Quality of Life Questionnaire - Core Questionnaire
QLQ-OG25	Quality of Life Questionnaire - Oesophago-Gastric Module 25 (OG-25)
QoL	Quality of life
QTc	QT interval corrected
QTcF	Fridericia-corrected QT interval
RECIST	Response Evaluation Criteria In Solid Tumors
SAE	serious adverse event
SAF	safety analysis set
SAP	statistical analysis plan
SD	stable disease
SOC	system organ class
SOD	Sum of diameters
TEAE	treatment-emergent adverse event
TSH	thyroid stimulating hormone
TTP	time to progression
ULN	upper limit of normal
VAS	visual analog scale
WHO	World health organization

List of Key Terms

**Terms**	**Definition of terms**
Baseline	Assessments of subjects as they enter a trial before they receive any treatment.
Endpoint	Variable that pertains to the efficacy or safety evaluations of a trial.
Enroll	To register or enter a subject into a clinical trial. NOTE: Once a subject has been randomized, the clinical trial protocol applies to the subject.
Intervention	The drug, device, therapy or process under investigation in a clinical study that is believed to have an effect on outcomes of interest in a study (e.g., health-related quality of life, efficacy, safety, pharmacoeconomics).
Investigational period	Period of time where major interests of protocol objectives are observed, and where the test drug or comparative drug (sometimes without randomization) is usually given to a subject, and continues until the last assessment after completing administration of the test drug or comparative drug.
Post investigational period	Period of time after the last assessment of the protocol. Follow-up observations for sustained adverse events and/or survival are done in this period.
Randomization	The process of assigning trial subjects to treatment or control groups using an element of chance to determine assignments in order to reduce bias.
Screening	A process of active consideration of potential subjects for enrollment in a trial.
Screen failure	Potential subject who did not meet 1 or more criteria required for participation in a trial.
Screening period	Period of time before entering the investigational period, usually from the time when a subject signs the consent until just before the test drug or comparative drug (sometimes without randomization) is given to a subject.
Study period	Period of time from the first site initiation date to the last site completing the study.
Study treatment	Includes Zolbetuximab/placebo and all components of CAPOX
Variable	Any entity that varies; any attribute, phenomenon or event that can have different qualitative or quantitative values.

1 INTRODUCTION

This Statistical Analysis Plan (SAP) contains technical and detailed elaboration of the principal features of the analysis described in the protocol, and includes procedures for executing the statistical analysis to fulfil the objectives of the study. The final SAP will be approved prior to database hard lock for the final PFS (and interim OS) analysis.

Changes that affected the statistical analyses from the planned analyses in the SAP will be documented in the Clinical Study Report (CSR).

2 STUDY OBJECTIVES AND DESIGN

2.1 Study Objectives

2.1.1 Primary Objective

The primary objective is to evaluate the efficacy of Zolbetuximab plus CAPOX compared with placebo plus CAPOX (as first-line treatment) as measured by progression free survival (PFS) in subjects with Claudin (CLDN)18.2 positive, HER2–negative locally advanced unresectable or metastatic gastric and GEJ adenocarcinoma.

2.1.2 Secondary Objectives

The secondary objectives are:

- To evaluate efficacy as measured by Overall Survival (OS) as a key secondary objective
- To evaluate efficacy as measured by Objective Response Rate (ORR)
- To evaluate efficacy as measured by Duration of Response (DOR)
- To evaluate safety and tolerability of Zolbetuximab
- To evaluate health-related quality of life (HRQoL) using the parameters as measured by European Organization for Research and Treatment of Cancer (EORTC) QLQ-C30, QLQ-OG25 plus STO22 Belching subscale, Global Pain (GP) and the EuroQOL Five Dimensions (EQ-5D-5L) questionnaires
- To evaluate the pharmacokinetics of Zolbetuximab
- To evaluate the immunogenicity profile of Zolbetuximab

2.1.3 Exploratory Objectives

The exploratory objectives are:

- To evaluate efficacy as measured by Time to Progression (TTP)
- To evaluate PFS following second-line anti-cancer treatment (PFS2)
- To evaluate Disease Control Rate (DCR)
- To evaluate potential genomic and/or other biomarkers that may correlate with treatment outcome to Zolbetuximab and CAPOX.
- To evaluate Health Resource Utilization (HRU)

2.2 Study Design

This global, multi-center, double-blind, 1:1 randomized, phase 3 study will evaluate efficacy of Zolbetuximab plus CAPOX versus placebo plus CAPOX as first-line treatment in subjects with CLDN18.2-positive, HER2-negative locally advanced unresectable or metastatic gastric and GEJ adenocarcinoma.

PFS as assessed by the Independent Review Committee (IRC) is the primary outcome. Secondary outcomes include OS, ORR, DOR, safety and tolerability, HRQoL, pharmacokinetic and the immunogenicity profile of Zolbetuximab. Exploratory outcomes include TTP, PFS2, DCR, biomarkers and HRU.

One interim analysis and one final analysis are planned for OS, while only one analysis is planned for PFS as the final analysis. The OS interim analysis will occur at the same time of the final PFS analysis (after pre-specified number of PFS events) and final OS analysis will be performed after the pre-specified number of OS events are observed. Refer to Section 6.9 for details on interim analysis.

Details of the study flow chart, dosing schedule, schedule of assessments are available in the protocol Section V.

2.3 Randomization

Subject randomization will be performed via IRT and treatment is assigned in a 1:1 ratio to:

- Arm A (Zolbetuximab in combination with CAPOX chemotherapy)
- Arm B (placebo in combination with CAPOX chemotherapy)

Prior to the initiation of the study treatment, the unblinded pharmacist/designee will be notified by the IRT system about the randomly assigned treatment. The unblinded pharmacist/designee will dispense the treatment according to the IRT system's assignment. Specific procedures for randomization through the IRT are contained in the IRT manual.

Randomization of subjects will use blocked randomization and be stratified by the following factors:

- Region (Asia vs. Non-Asia)
- Number of Metastatic Sites (0 to 2 vs. ≥ 3)
- Prior Gastrectomy (Yes vs. No)

3 SAMPLE SIZE

Approximately 500 subjects will be randomized in a 1:1 ratio to receive Zolbetuximab in combination with CAPOX chemotherapy (Arm A) or placebo in combination with CAPOX chemotherapy (Arm B). The planned 344 PFS events during the study will provide 96% power to detect a difference in PFS between Arm A (Zolbetuximab + CAPOX) with the assumption of 9 months median PFS time and Arm B (placebo + CAPOX) with the assumption of 6 months median PFS time (hazard ratio = 0.67) at the overall 1-sided 0.025 significance level. Similarly, the planned 386 OS events during the study will provide 80%

power to detect a difference in OS between Arm A (Zolbetuximab + CAPOX) with the assumption of 14.7 months median OS time and Arm B (placebo + CAPOX) with the assumption of 11 months median OS time (hazard ratio = 0.75) at the overall 1-sided 0.025 significance level.

4 ANALYSIS SETS

In accordance with International Conference on Harmonization (ICH) recommendations in guidelines E3 and E9, the following analysis sets will be used for the analyses.

The determination of whether subjects are included or excluded from the safety and efficacy analysis sets will be made prior to database hard-lock and unblinding. Inclusions and exclusions from the pharmacokinetics analysis set (PKAS) may occur after unblinding.

4.1 Full Analysis Set

The Full Analysis Set (FAS) will consist of all subjects who are randomized to 1 of the treatment arms. Subjects would be analyzed according to the treatment they were randomized to. The FAS will be used for summaries of demographic and baseline characteristics and all efficacy analyses. FAS in this study is identical to intent-to-treat (ITT) set (only the name "FAS" will be used).

4.2 Safety Analysis Set

The safety analysis set (SAF) will consist of all subjects who received at least 1 dose of any study drug (Zolbetuximab/placebo/CAPOX). Subjects would be analyzed according to the actual treatment they received. The SAF will be used for summaries of demographic and baseline characteristics and all safety and tolerability-related variables. In case that SAF and FAS are identical, summaries of demographic and baseline characteristics will not be repeated on SAF.

4.3 Per Protocol Set

The per protocol set (PPS) will consist of the subset of the FAS who do not meet criteria for PPS exclusion. These criteria are to capture relevant non-adherence to the protocol and will be defined below or in Classification Specifications. In addition, subjects in the PPS are required to have both valid baseline imaging and at least 1 evaluable post-baseline imaging assessment (i.e., not a "Not evaluable (NE)" assessment). If a subject died before his/her first scheduled imaging, he/she will be included in the PPS unless he/she meets other PPS exclusion criteria.

Final judgments on exclusion of subjects from the PPS will be made prior to database hard lock and will be documented in the Classification Specifications. The PPS will be used for sensitivity analyses of the primary (PFS) and select secondary efficacy endpoints (OS, ORR, DOR).

4.3.1 Reasons for Exclusion from Per Protocol Set

The following reasons may lead to subject's exclusion from PPS:

1. Subject has no valid imaging assessment at baseline
2. Subject has no measureable disease at baseline
3. Subject does not have at least 1 evaluable imaging assessment post-baseline
4. Subject received wrong study drug (e.g., randomized to Arm A but received Arm B drug)
5. Subject did not complete at least two doses of Zolbetuximab (arm A only) and 2 doses of CAPOX.
6. Subject violates major entry criteria and should not have been randomized.
7. Subject was randomized but received no study drug.
8. Subject received concomitant treatment(s) prohibited by protocol or other anti-cancer treatment prior to the first scheduled imaging assessment.

4.4 Pharmacokinetics Analysis Set (PKAS)

The PKAS consists of the subset of the SAF for which at least one concentration data is available. Additional subjects may be excluded from the PKAS at the discretion of the pharmacokineticist. The PKAS will be used for all summaries of the pharmacokinetic data.

5 ANALYSIS ENDPOINTS

5.1 Primary Efficacy Endpoint

The primary endpoint is PFS, which is defined as the time from the date of randomization until the date of radiological PD (per Response Evaluation Criteria In Solid Tumors [RECIST] 1.1 by independent review committee [IRC]) or death from any cause, whichever is earliest.

5.2 Secondary Efficacy Endpoints

- OS, defined as the time from the date of randomization until the date of death from any cause.
- ORR, defined as the proportion of subjects who have a best overall response (BOR) of complete response (CR) or partial response (PR) as assessed by IRC per RECIST 1.1.
- DOR, defined as the time from the date of the first response (CR/PR) until the date of PD as assessed by IRC per RECIST 1.1 or date of death from any cause, whichever is earliest.
- Health-related Quality of Life questionnaires:
    - EORTC QLQ-C30 questionnaire - a 30-item cancer-specific instrument consisting of 5 functional scales (physical, role, emotional, social and cognitive), 9 symptom scales/items and a global health status scale;
    - EORTC QLQ-OG25 questionnaire - a 25-item instrument that evaluates gastric and GEJ cancer-specific symptoms. This module consists of 6 scales: dysphagia (3 items), eating restrictions (4 items), reflux (2 items),

odynophagia (2 items), pain and discomfort (2 items) and anxiety (2 items), as well as 10 single items: eating in front of others, dry mouth, trouble with taste, body image, trouble swallowing saliva, choked when swallowing, trouble with coughing, trouble talking, weight loss and hair loss. In addition a belching and a bile or acid coming in your mouth question from the STO-22 follows the OG-25 questionnaire.

- Global Pain (GP) questionnaire: a single assessment of overall pain.
- EQ-5D-5L questionnaire: a standardized 6-item instrument that cover 5 main domains (mobility, self-care, usual activities, pain/discomfort and anxiety/depression) and a general visual analog scale (VAS) for health status.

5.3 Exploratory Efficacy Endpoints

The exploratory endpoints are:

- TTP, defined as the time from the date of randomization until the date of PD as assessed by IRC per RECIST 1.1
- PFS2, defined as the time from the date of randomization until the date of PD (per subject's local physician) following subsequent anti-cancer therapy, death from any cause, or start of any other anti-cancer therapy, whichever is earliest.
- DCR, defined as the proportion of subjects who have a BOR of SD, CR or PR as assessed by IRC per RECIST 1.1.
- Health Resource Utilization (HRU) questionnaire: includes number of ER visits, number and duration of hospital stays, and number of doctor office visits that occur outside of the clinical trial.

5.4 Safety Endpoints

Safety and tolerability endpoints include AEs, laboratory test results, vital signs, electrocardiograms (ECGs) and Eastern Cooperative Oncology Group (ECOG) performance status.

5.4.1 AE

AE will be assessed by evaluation of the following variables:

- Treatment-emergent adverse events (TEAEs; frequency, severity, seriousness, and relationship to study drug)
  - TEAE is defined as an adverse event observed after starting administration of the study drug through 30 days after the last dose of study drug.
  - If the adverse event occurs on Day 1 and the onset check box is marked "Onset after first dose of study drug" or the onset check box is left blank, then the adverse event will be considered treatment emergent.
  - If the adverse event occurs on Day 1 and the onset check box is marked "Onset before first dose of study drug", then the adverse event will not be considered treatment emergent.

    - If a subject experiences an event both during the pre-investigational period and during the investigational period, the event will be considered as TEAE only if it is reported with a new start date (i.e., as a new AE).
    - Any AEs with onset dates completely missing will be considered TEAEs in summaries. AEs with partially missing onset dates will be assumed TEAEs unless the available portion of the date indicates that the onset was strictly before start of study medication.
    - A drug-related TEAE is defined as any TEAE with possible relationship to study treatment as assessed by the investigator or with missing assessment of the causal relationship.
- Serious adverse events (SAEs) include adverse events that are flagged as serious by the investigator on eCRF, or upgraded by the Sponsor based on review of the Sponsor's list of Always Serious terms.

5.4.2 Clinical Laboratory Variables

Refer to protocol Section 5.4.3 for a table of the laboratory tests that will be performed during the conduct of the study. Refer to the Protocol Schedule of Assessments for evaluation schedule.

5.4.3 Vital Signs

Vital signs will include systolic and diastolic blood pressure (mmHg), radial pulse (beats/min) and body temperature. Serial vital signs will be collected during Zolbetuximab dosing visits.

5.4.4 12-lead electrocardiogram (ECG)

A single 12-lead ECG will be performed at the time points outlined in the Protocol Schedule of Assessments. ECGs will be assessed locally.

5.4.5 ECOG performance score

ECOG performance scores will be collected.

5.4.6 Physical examination

Targeted (symptom driven) physical exams should be conducted every 3 weeks on day 1 of each cycle. If clinically significant worsening of findings from baseline is noted at any study visit, the changes will be documented as AEs on the AE eCRF.

5.5 Other Endpoints

5.5.1 Pharmacokinetic Endpoints

PK of Zolbetuximab as measured by C_{trough}.

5.5.2 Immunogenicity

Immunogenicity of Zolbetuximab as measured by the frequency of anti-drug antibody (ADA) positive subjects.

5.5.3 Biomarkers Endpoints

Potential genomic and/or other exploratory biomarkers that may be related to treatment outcome of Zolbetuximab.

6 STATISTICAL METHODOLOGY

6.1 General Considerations

Continuous data will be summarized descriptively including the number of subjects, mean, standard deviation, median, minimum and maximum. Categorical data will be summarized by frequencies and percentages. Percentages by categories will be based on the number of subjects with no missing data, i.e. the percentages for the non-missing categories will add up to 100%. All non-coded free-text variables will be displayed in data listings only.

Summaries based on FAS and PPS (e.g. disposition, baseline characteristics and efficacy endpoints) will be presented by randomized treatment. Safety summaries based on SAF and summaries based on PKAS will be presented by actual treatment received.

All statistical comparisons will be made using one-sided test at the α=0.025 significance level unless specifically stated otherwise. All null hypotheses will be: Arm A is not better than Arm B, all alternative hypotheses will be: Arm A is better than Arm B, unless specifically stated otherwise.

All data summarization and analyses will be performed using SAS® Version 9.3 or higher on UNIX. Specifications for table, figures, and data listing formats can be found in the TLF specifications document for this study.

Study day for safety assessments (e.g., laboratory assessment, onset of adverse events, vital signs, etc.) will be calculated in reference to the first dose date. For assessments conducted before the first dose, study day will be calculated as (assessment date – first dose date). For assessments conducted on or after the first dose, study day will be calculated as (assessment date – first dose date + 1). Study day for efficacy events (progression, death, tumor responses CR/PR) will be calculated in reference to the randomization date (event/assessment date – randomization date + 1).

For efficacy evaluation, baseline is defined as the last available measurement before randomization. For safety evaluation, baseline is defined as the last available measurement before the first dose. Unless otherwise specified, all summaries will be presented by treatment arm.

Study drug is defined as any one of the 3 components (Zolbetuximab, capecitabine and oxaliplatin) for Arm A and any one of the 3 components (placebo, capecitabine and oxaliplatin) for Arm B. Date of first dose of study drug is the date of start of infusion of the first component of study drug administered, or oral dosing of Capecitabine, whichever is earlier. Date of last dose of study drug is the date of stop of infusion of the last component of study drug administered or oral dosing of Capecitabine, whichever is later.

All by-visit summaries will use CRF visit (e.g., Cycle 3 Day 15) as described in Section 6.10.1.

6.2 Study Population

In general, data such as patient disposition, demographics and baseline characteristics will be summarized for FAS and SAF by treatment arm and overall, unless specifically stated otherwise. In the event when FAS is identical to SAF (i.e., no one received the wrong study drug), then these data summaries will not repeated for SAF.

6.2.1 Disposition of Subjects

The following summaries will be presented. A table may include one or more of the summaries.

- Number and percentage of subjects with informed consent, discontinued before randomization (screening failures), randomized (overall only);
- Number and percentage of randomized subjects in each analysis set, by treatment group and overall;
- Number and percentage of subjects completed/discontinued CAPOX regimen, by primary reason for treatment discontinuation;
- Number and percentage of subjects discontinued Zolbetuximab/placebo, by primary reason for treatment discontinuation;
- Number and percentage of subjects completed/not completed the 30-day post- CAPOX safety follow-up visit, by primary reason for not completing the visit;
- Number and percentage of subjects completed/not completed the 30-day post-Zolbetuximab/placebo safety follow-up visit, by primary reason for not completing the visit;
- Number and percentage of subjects completed/not completed the 90-day post- CAPOX follow-up visit, by primary reason for not completing the visit;
- Number and percentage of subjects completed/not completed the 90-day post-Zolbetuximab/placebo follow-up visit, by primary reason for not completing the visit;
- Number and percentage of subjects completed/not completed post-treatment follow-up, by primary reason for completing or not completing post-treatment follow-up;
- Number and percentage of subjects completed or discontinued survival follow-up period, by primary reason for survival follow-up discontinuation;
- Number and percentage of subjects excluded from PPS by reason for exclusion defined in Section 4.3.1 for FAS.

6.2.2 Protocol Deviations

The number and percentage of subjects with the following major protocol deviation criteria will be summarized for each criterion and overall, by treatment group and overall as well as by investigative site, for FAS and SAF. Subjects deviating from a criterion more than once will be counted once for the corresponding criterion. Any subjects who have more than one protocol deviation will be counted once in the overall summary.

The unique identifiers for major protocol deviation will be as follows:

PD1 - Entered into the study even though they did not satisfy entry criteria,

PD2 - Developed withdrawal criteria during the study and was not withdrawn,

PD3 - Received wrong treatment or incorrect dose,

PD4 - Received excluded concomitant treatment.

6.2.3 Demographic and Other Baseline Characteristics

Demographic variables (sex, age, age groups defined in the subgroup section, race, ethnicity, country), height, weight, BMI, BSA, tobacco history, baseline ECOG status, and the three stratification factors (listed in Section 2.3) will be summarized for FAS, SAF and PPS.

Primary diagnosis and will be summarized for FAS.

Medical history is coded in MedDRA, and will be summarized by System Organ Class (SOC) and Preferred Term (PT) by treatment arm for FAS and SAF.

6.2.4 Transfusions

The blood product, duration, number of units will be summarized for FAS.

6.2.5 Previous and Concomitant Medications

Previous and concomitant medications will be summarized in separate tables by therapeutic subgroup (ATC 2nd level) and chemical subgroup (ATC 4th level) and preferred WHO name by treatment group for the FAS. Subjects taking the same medication multiple times will be counted once per medication and investigational period. A medication that can be classified into several chemical and/or therapeutic subgroups is presented in all chemical and therapeutic subgroups.

Previous medications are defined as medications that patients started prior to first administration of study medication. Concomitant medications are defined as any medications that patients took after the first dose of study medication. Medications that started prior to first administration of study drug and continued while study drug was given will be counted as both previous and concomitant medications.

6.2.6 Non-medication Therapies

Reason for use will be summarized for FAS.

6.2.7 New Anti-Cancer Therapies

Subsequent anti-cancer therapies will be summarized by treatment arm by drug class for FAS.

6.2.8 Prior Radiation Therapy

The following variables will be summarized for FAS: whether subject received prior radiation therapy, area, duration, and reason of radiation therapy.

6.2.9 Prior Procedures for Primary Cancer

Frequency tabulations of subjects with surgery or procedures for the treatment of the primary cancer will be presented by treatment group and overall for FAS.

6.2.10 Prior Cancer Chemotherapy

The following variables will be summarized for FAS: whether subject received any chemotherapy medication, name and duration of chemotherapy, and reason for discontinuing medication.

6.3 Study Drug Exposure and Compliance

Duration and compliance of study drug will be summarized for SAF and PPS by treatment group and overall.

The following variables will be derived and summarized:

- Duration of Zolbetuximab or placebo and Oxaliplatin, defined as (date of last infusion – date of first infusion + 1). For those who did not receive any dose, duration will be 0.
- Duration of Capecitabine, defined as (date of last dose – date of first dose + 1). For those who did not receive any dose, duration will be 0.
- Duration of period for which all components are administered and duration of period for Any Component Administered
- Proportion of subjects who completed the 8 cycles of CAPOX treatment.
- Number of infusions administered, number of infusions entirely administered and number of infusion not entirely administered, number of infusions with dose adjustment, Number of infusions with dose adjustment due to AE, number of infusions with delay, Number of infusions with delay due to AE, number of infusions with interruption, Number of infusions with interruption due to AE, number of infusions prematurely discontinued for Zolbetuximab and Oxaliplatin and Number of infusions prematurely discontinued for Zolbetuximab and Oxaliplatin due to AE.
- Number of cycle administered for oral dosing of capecitabine, number of cycle entirely administered and number of cycle not entirely administered, number of cycle with dose adjustment, Number of cycle with dose adjustment due to AE, number of cycle with delay, number of cycle with delay due to AE , number of cycle with interruption, number of cycle with interruption due to AE, number of cycle prematurely discontinued for Capecitabine, number of cycle prematurely discontinued for Capecitabine due to AE.
- Average infusion time, calculated as (stop time – start time), for Zolbetuximab, placebo and Oxaliplatin.
- Cumulative actual dose of Zolbetuximab/placebo and each component of CAPOX.
- Average amount of dose per infusion for each component administered via infusion.
- Average amount of dose per planned dosing day for Capecitabine.
- Relative dose intensity (RDI), defined as (Cumulative actual dose/cumulative dose if initially assigned dose had been given to all scheduled dosing visits).

- RDI category: <50, 50 to 80 inclusive, >80, unknown
- Number and percentages of subjects with the following cumulative categories of study drug(each component) duration will be summarized: ≥ 1d, > 6w, > 12w, > 24w, > 36w, > 48w, > 72w

6.4 Analysis of Efficacy

To address multiplicity, a gatekeeping testing strategy will be used for PFS (primary efficacy endpoint) and OS (key secondary endpoint). PFS will be tested once at 1-sided significance level of 0.025. Only if PFS is significant, hypothesis testing for OS interim and OS final analyses will be performed. An O'Brien-Fleming type alpha-spending function [Lan & DeMets, 1983] will be utilized to control the overall 1-sided 0.025 significance level for the OS interim and final analyses. Other secondary endpoints' testing will not be multiplicity adjusted.

For imaging assessments of tumor, date of assessment for each timepoint is defined as the date of last scan (if there are multiple scans over several days) for that timepoint, not the date when the overall timepoint response is recorded by radiologist in the system, with the exception of PD date. PD date is defined in Table 1. Date of time point assessment of tumor as determined by IRC/investigators will be used for analysis.

6.4.1 Analysis of Primary Efficacy Endpoint (PFS)

6.4.1.1 Primary Analysis for Primary Efficacy Endpoint

The primary analysis of PFS will use radiological assessment of PD by the IRC and be performed for FAS. The hypothesis to be tested is:

- H_0: PFS of Arm A is not prolonged compared to that of Arm B
- H_a: PFS of Arm A is prolonged compared to that of Arm B

Comparison of Arm A and Arm B will be tested at 1-sided significance level of 0.025.

The distribution and median of PFS will be estimated for each treatment arm using Kaplan-Meier methodology. PFS rates at 6m, 12m, and 18m will be presented by Kaplan-Meier method too. In addition, numbers of subjects with PFS events and censored and 95% CI for median PFS and PFS rates will be presented.

Hypotheses testing between Arm A and Arm B will be performed using log-rank test stratified by:

- Region (Asia vs. Non-Asia)
- Number of Metastatic Sites (0 to 2 vs. ≥ 3)
- Prior Gastrectomy (Yes vs. No)

In addition, stratified Cox proportional hazards model will be used to estimate the hazard ratio and the corresponding 95% confidence interval. Table 1 defines PFS endpoint for the primary analysis. Evaluable radiological assessments include all assessments except those assessed by IRC as NE (not evaluable).

Table 1 PFS Primary Analysis Definition (based on IRC radiological assessments only)

Situation	Date of Event or Censoring	Outcome
No baseline imaging assessments	Date of randomization	Censored
No evaluable post-baseline imaging assessments, no death	Date of randomization	Censored
**Subject did not receive new anti-cancer therapy (ACT):**		
Radiological PD documented per RECIST v1.1	Date of first radiological PD (defined as earliest of date of scan showing new lesion if PD is based on new lesion or date of last scan of target lesions if PD is based on increase in sum of diameters (SOD) of target lesions)	Event
No radiological PD, but death recorded on eCRF	Date of death	Event
Neither radiological PD nor death	Date of last radiological assessment	Censored
**Subject received new anti-cancer therapy (ACT)*:**		
Radiological PD per RECIST v1.1 or death documented only after start of new ACT	Date of last radiological assessment before start of new ACT	Censored
Radiological PD documented per RECIST v1.1 before start of new ACT	Date of first radiological PD	Event
No radiological PD nor death	Date of last radiological assessment before start of new ACT	Censored
**Missed >=2 scheduled radiological assessments:**		
If radiological PD or death occurs after missing 2 or more scheduled radiological assessments**	Date of last radiological assessment	Censored

Note: PFS = date of event or censoring – date of randomization + 1. Patient cannot be censored at “NE”. If last radiological assessment is NE, then censor at the assessment before NE. If NE is the only previous assessment, then PFS will be censored at Day 1.

*New ACT includes new anti-cancer surgery, radiotherapy, chemo and immunotherapy (other than Zolbetuximab and CAPOX components) after randomization. If a subject in Arm B switches from placebo to Zolbetuximab, it is also considered start of new ACT.

**If the first radiological assessment after subject missed >=2 imaging assessments is SD or better and it’s confirmed that subject did not take any other ACT during the missing period, the following imaging assessments will be used rather than censored.

6.4.1.2 Sensitivity Analysis 1 for Primary Efficacy Endpoint

The primary analysis will be repeated for PPS.

6.4.1.3 Sensitivity Analysis 2 for Primary Efficacy Endpoint

The primary analysis will be repeated using radiologic PD assessments by local investigators only. In addition, a summary of discordance between IRC’s and local investigator’s PD assessments will be presented.

6.4.1.4 Sensitivity Analysis 3 for Primary Efficacy Endpoint

This analysis will treat likely informative censoring as PFS events. The primary analysis will be repeated with the following cases treated as PFS events rather than censored (whichever earliest):

- If subject dropped out of imaging follow-up without documented PD by IRC and there was investigator-reported clinical progression around or after the time of last imaging assessment, then the next scheduled date of imaging (i.e., date of last imaging assessment + 9w or 12w) will be treated as date of PFS event (even though the next scheduled imaging did not take place).
- If subject dropped out of imaging follow-up without documented PD by IRC and there was ECOG performance status worsening from baseline (from 0-1 to >=2) around or after the time of last imaging assessment, then the next scheduled date of imaging will be treated as date of PFS event.
- Start date of new ACT will be treated as date of PFS event, when there is no prior documented PD by IRC.
- If subject missed >=2 scheduled imaging assessments without prior documented PD by IRC and there was investigator-reported clinical progression around or after the time of last imaging assessment prior to missing, then the next scheduled date of imaging after the last non-missing assessment will be treated as date of PFS event (even though the next scheduled imaging did not take place).
- If subject missed >=2 scheduled imaging assessments without prior documented PD by IRC and there was ECOG performance status worsening from baseline (from 0-1 to >=2) around or after the time of last imaging assessment prior to missing, then the next scheduled date of imaging after the last non-missing assessment will be treated as date of PFS event.

6.4.1.5 Other Analysis of Primary Efficacy Endpoint

Imaging assessment interval (weeks) will be summarized for each treatment arm for the two periods: <=54 weeks and >54 weeks. If imbalance of imaging assessment interval is observed between the two arms, additional sensitivity analysis may be performed using protocol-planned dates of imaging assessments instead of actual dates of assessments. In addition, duration of imaging follow-up, defined as (date of the last on-study imaging assessment – date of randomization + 1), will be summarized for each treatment arm.

6.4.2 Analysis of Secondary Efficacy Endpoints

6.4.2.1 Overall Survival

A key secondary endpoint OS is defined as the time from date of randomization to the documented date of death from any cause. All deaths will be included, regardless of whether death occurred while the subject is still taking study drug or after the subject discontinue study drug. OS analysis will be performed for FAS.

Table 2 OS Definition

**Situation**	**Date of Event or Censoring**	**Outcome**
Death before analysis cutoff date	Date of death	Event
Last known alive date is before cutoff date	Last known alive date	Censored
Death after analysis cutoff date	Analysis cutoff date	Censored
Last known alive date is after cutoff date	Analysis cutoff date	Censored

OS = Date of Event or Censor – Date of Randomization +1

To maintain the overall Type I error rate at the 0.025 significance level, the hypothesis testing for OS interim and OS final analyses will be performed only if the null hypothesis in PFS primary analysis is rejected at the overall 1-sided 0.025 significance level.

The formal OS interim analysis is planned when the final PFS analysis occurs with the pre-specified number of PFS events. A group sequential design using the O'Brien-Fleming type alpha-spending function [Lan & DeMets, 1983] will be utilized to control the overall 1-sided 0.025 significance level (East®) for the OS analysis. The independent data monitoring committee (IDMC) may recommend terminating the trial for favorable results at the formal OS interim analysis. In the case of favorable results, the 1-sided significance level for superiority is 0.0074 for the interim OS analysis and 0.0228 for the final OS analysis. These alpha boundaries are based on an information factor of 70% and are subject to adjustment if observed information factor deviates from 70%. If the 1-sided P-value of the interim analysis is less than 0.0074, the IDMC may recommend terminating the trial for success without the need to conduct the final OS analysis. If the study is not stopped after the interim analysis, the final OS analysis will occur after 100% of the planned number of deaths have been observed.

The distribution and median of OS, as well as OS rates at 12m, 18m, and 24m, will be estimated for each treatment arm using Kaplan-Meier methodology. 95% CI for median OS and milestone OS rates will be presented. Arm A and Arm B will be compared using the log-rank test stratified by the same stratification factors used for PFS analysis. The hypothesis to be tested is:

- H_0: OS of Arm A is not prolonged compared to Arm B
- H_a: OS of Arm A is prolonged compared to Arm B

In addition, stratified Cox proportional hazard model will be used to estimate the hazard ratio and the corresponding 95% CI.

As a sensitivity analysis, OS analysis will be repeated on the PPS.

6.4.2.2 Objective Response Rate

The ORR is defined as the proportion of subjects with a BOR of complete response (CR) or partial response (PR) based on IRC per RECIST V1.1.

Best overall response (BOR) will be determined once all tumor response data for the subject is available. Subjects' BOR will be determined as outlined in RECIST V1.1 criteria based on

IRC assessments. BOR is defined as the best response among all timepoints' overall responses excluding NE responses (CR is better than PR and PR is better than SD and SD is better than PD). If all timepoint overall responses are NE, BOR is NE. For BOR of SD, SD must be documented as present at least once and at least 8 weeks after randomization. If the first assessment of SD does not meet the minimum "8 weeks from randomization" time window, that assessment of SD will be treated as NE in analysis. Timepoint responses after start of new ACT will not be used in determining BOR.

The comparison of ORR between Arm A and Arm B will be performed using stratified Cochran-Mantel-Haenszel (CMH) test with the same stratification factor used for the PFS analysis. In addition, ORR for each arm will be estimated and corresponding 95% CI will be constructed using Clopper-Pearson method.

In addition, percent of subjects with BOR of CR, PR or SD as well as ORR and DCR will be summarized.

Sensitivity analyses include:

- Analysis for PPS
- Analysis of ORR with confirmation, defined as the proportion of subjects with best overall response as confirmed CR or confirmed PR based on the RECIST v1.1 as assessed by IRC. Confirmation of CR or PR should occur at the next scheduled assessment (>= 4 weeks following the initial assessment at which CR or PR is observed). See Table 3 for rules of confirmation of CR and PR.

Table 3 Confirmation of overall response:

Overall response at current timepoint	Overall response at next timepoint	Confirmed overall response at current timepoint
CR	CR	CR
PR	CR/PR	PR
CR/PR	PD	SD provided minimum criteria for SD duration met, otherwise, PD
PR	SD	SD
SD	CR/PR	SD
SD	SD/PD/NE	SD provided minimum criteria for SD duration met, otherwise, PD or NE.
CR	NE, followed by CR at the following assessment	CR
PR	NE, followed by CR/PR at the following assessment	PR
CR	NE	SD provided minimum criteria for SD duration met, otherwise, NE
PR	NE	SD provided minimum criteria for SD duration met, otherwise, NE
NE	NE	NE

6.4.2.3 Duration of Response

DOR is defined as the time from the date of the first CR or PR (whichever is first recorded) as assessed by IRC to the date of radiological PD as documented by IRC or death, whichever is earlier. The DOR analysis will be performed on the subset of FAS who have at least one CR or PR documented by IRC. If a subject has not progressed, the subject will be censored at the date of last evaluable radiological assessment or at the date of first CR/PR if no later evaluable radiological assessment is available. Other censoring used for the PFS analysis (see Table 1) will apply to DOR too.

DOR = Date of Event or Censor – Date of the first CR/PR + 1.

The distribution of DOR will be estimated for each treatment arm using Kaplan-Meier methodology and compared between Arm A and Arm B using the log-rank test stratified by the same stratification factors used for the PFS analysis. In addition, stratified Cox proportional hazard model will be used to estimate the hazard ratio and the corresponding 95% CI.

As sensitivity analyses, DOR analysis will be repeated on the PPS as well as for confirmed responses. For confirmed CR/PR, date of first confirmed CR/PR is defined as the date of first assessment of CR/PR (not the later assessment confirming the previous CR/PR).

6.4.2.4 Health-Related Quality of Life

6.4.2.4.1 Scoring of HRQoL Questionnaires

For EORTC QLQ-C30, scores for 5 functional scales (physical, role, emotional, social and cognitive), 9 symptom scales/items, and global health status scale will be calculated according to the EORTC scoring manual in Appendix 9.2 (Fayers, et al, 2001). These scores will be standardized to a 0-100 scale. A high score for a functional scale and the global health status represents a healthy level of functioning (high QoL), while a high score for a symptom scale or item represents a severe level of symptoms (low QoL). In addition, the QLQ-C30 Summary Score (Giesinger et al, 2016) will be calculated using 27 out of 30 items as follows (excluding 3 items on financial impact and global health status):

QLQ-C30 Summary Score = (Physical Functioning+ Role Functioning+ Social Functioning+ Emotional Functioning+ Cognitive Functioning+ 100-Fatigue+ 100-Pain+ 100-Nausea_Vomiting+ 100-Dyspnoea+ 100-Sleeping Disturbances+ 100-Appetite Loss+ 100-Constipation+ 100-Diarrhoea)/13.

The QLQ-C30 Summary Score should only be calculated if all of the required 13 scale/item scores are available (using scale scores based on the completed items, provided that at least 50% of the items in that scale have been completed). A high Summary Score represents high QoL.

EORTC QLQ-OG25 questionnaire consists of 6 scales: dysphagia (items 1-3), eating restrictions (items 4-7), reflux (items 8-9), odynophagia (items 10-11), pain and discomfort (items 12-13) and anxiety (items 14-15), as well as 10 single items: eating in front of others

(item 16), dry mouth (item 17), trouble with taste (item 18), body image (item 19), trouble swallowing saliva (item 20), choked when swallowing (item 21), trouble with coughing (item 22), trouble talking (item 23), weight loss (item 24) and hair loss (item 25).

EORTC QLQ-OG25 6 scales plus 10 single items will be scored in the same way as QLQ-C30 symptoms scales/items. The scores will be transformed to a 0-100 scale too. Higher score means severer level of symptoms.

In addition, the EORTC QLQ-C30 and OG25 individual symptom scores and the Belching questionnaire will be categorized as: 1-2="None/Slight", 3="Moderate", 4= "Severe". The EQ-5D-5L scores (on 1-5 scale) will be categorized as: 1-2="None/Slight", 3=" Moderate", 4-5="Severe/Extreme".

6.4.2.4.2 Analysis of HRQoL Questionnaires

Analyses will be by treatment arm and based on FAS. Completion rate for each questionnaire, defined as proportion of FAS subjects who completed that questionnaire, will be summarized by visit.

<u>For QLQ-C30's 5 functional scales, global health status, Summary Score, and EQ-5D-5L VAS</u>

Scores (on 0-100 range) as well as change from baseline will be summarized by visit for each visit where at least 10 subjects are evaluable for any treatment arm. Summary statistics will not be provided for the treatment arm for the visit if that arm has less than 10 subjects at that visit. Plot of mean change from baseline over time will be presented by treatment arm for each score. Highest change from baseline of each subject (highest value post baseline – baseline value) will be summarized by treatment arm.

<u>For QLQ-C30 and QLQ-OG25 symptom:</u>

Scores (on 0-100 range) will be summarized separately in the same way as above. In addition, lowest change from baseline of each subject (lowest value post baseline – baseline value) will be summarized by treatment arm. Frequency distribution of categorized QLQ-C30 and QLQ-OG25 symptom scores will be presented by treatment arm and visit. Shift from baseline category to subject's worst post-baseline category will be tabulated.

<u>EQ-5D-5L 5 questions</u>

Scores (on 1-5 range) will be analyzed using descriptive statistics including change from baseline. Categorized scores will be tabulated using shift table (shift from baseline to worst category post baseline).

<u>Global pain</u>

Score and change from baseline will be summarized by visit.

<u>Belching questionnaire</u>

Score and change from baseline will be summarized by visit.

6.4.3 Analysis of Exploratory Efficacy Endpoints

6.4.3.1 Time to Progression

TTP is defined as the time from the date of randomization until the date of PD per RECIST 1.1 by IRC. TTP does not include deaths as event. For deaths before the first documented PD by IRC, subjects will be censored at the time of last evaluable radiological assessment. The TTP analysis assumes that deaths without documented PD are not related to tumor progression and estimates TTP for these dead subjects (who did not have documented PD prior to death) as if the subjects had not die. See Table 4 for derivation of the TTP endpoint. Kaplan-Meier and log-rank methods will be applied to the TTP endpoint.

Table 4 TTP Definition (based on IRC radiological assessments only)

Situation	Date of Event or Censoring	Outcome
No baseline imaging assessments	Date of randomization	Censored
No evaluable post-baseline imaging assessments, no death	Date of randomization	Censored
**Subject did not receive new anti-cancer therapy (ACT):**		
Radiological progression documented per RECIST v1.1	Date of radiological PD (defined as earliest of date of scan showing new lesion if PD is based on new lesion or date of last scan of target lesions if PD is based on increase in sum of diameters (SOD) of target lesions)	Event
No radiological progression, but death recorded on eCRF	Date of last radiological assessment	Censored
Neither radiological progression nor death	Date of last radiological assessment	Censored
**Subject received new anti-cancer therapy (ACT):**		
Radiological progression documented per RECIST v1.1 after new ACT	Date of last radiological assessment before start of new anti-cancer therapy	Censored
Radiological progression documented per RECIST v1.1 before new ACT	Date of radiological PD	Event
No radiological progression before new ACT but death recorded	Date of last radiological assessment before start of new anti-cancer therapy	Censored
No radiological progression nor death	Date of last radiological assessment before start of new anti-cancer therapy	Censored
**Missed >=2 scheduled radiological assessments:**		
If radiological progression or death occurs after missing 2 or more scheduled radiological assessments	Date of last radiological assessment	Censored

6.4.3.2 Progression Free Survival After Subsequent Therapy (PFS2)

PFS2 is defined as the time from the date of randomization until the date of radiological/objective PD (per subject's local physician) following subsequent (2nd line) anti-cancer therapy or death from any cause, whichever is earliest. In cases where PFS2 cannot be reliably determined, end date of subsequent (2nd line) ACT or start date of 3rd line ACT may

be used as the event date. Otherwise, subjects will be censored. Subjects who are alive and for whom a PFS2 event has not been observed should be censored at the last time of radiological assessment (IRC or local) showing no objective PD.

The distribution of PFS2 will be estimated for each treatment arm using Kaplan-Meier methodology and compared between Arm A and Arm B using stratified log-rank test with the same stratification factors used for the PFS analysis. In addition, stratified Cox proportional hazard model will be used to estimate the hazard ratio and the corresponding 95% CI.

Table 5 PFS2 Definition (as compared to PFS)

Situation	PFS date (Event/Censored)	PFS2 date (Event/Censored)
Subject died before the first PD by IRC	Date of death (Event)	Date of death (Event)
Subject had PD by IRC then died before receiving new ACT	Date of PD (Event)	Date of death (Event)
Subject had first PD by IRC then received new ACT then had second PD or death	Date of first PD (Event)	Date of second PD or death, whichever earlier (Event)
Subject had no PD by IRC or death and had not started new ACT	Date of last radiological assessment by IRC (Censored)	Date of last radiological assessment by IRC or local physician, whichever later (Censored)
Subject had no PD by IRC or death but started new ACT and had no subsequent PD or death	Date of last radiological assessment by IRC before the start of new ACT (Censored)	Date of last radiological assessment by IRC or local physician, whichever later (Censored)
Subject had no PD by IRC or death but started new ACT and had subsequent PD or death	Date of last radiological assessment by IRC before the start of new ACT (Censored)	Date of PD or death after the start of new ACT, whichever earlier (Event)

6.4.3.3 Disease Control Rate

The DCR is defined as the proportion of subjects with a BOR of SD, CR or PR based on RECIST 1.1 by IRC.

The comparison of DCR between Arm A and Arm B will be performed using CMH test with the same stratification factor used for PFS analysis. In addition, DCR for each arm will be estimated and corresponding 95% CI will be constructed.

6.4.3.4 Health Resource Utilization

HRU variables by visit and their sums over all visits, will be summarized by treatment arm:

- Number of ER visits
- Number of ER visits with hospitalization
- Duration of ER hospitalizations
- Number of hospital admissions
- Duration of stay in hospital

- Duration of general practitioner visits
- Number of specialist visits

The above summaries will be repeated for the time period before subjects' radiological PD.

6.5 Analysis of Safety

Safety analyses will be based on SAF and by treatment arm and overall. All SAF subjects will be analyzed according to the actual treatment they received. Astellas Standard TLF templates should be followed wherever applicable.

Safety analyses will only include data collected during and after the first dose of the first study drug component given, up to 30 days after the last dose of the last study drug component.

6.5.1 Adverse Events

AEs will be coded using the Medical Dictionary for Regulatory Activities (MedDRA) and graded using NCI-CTCAE. MedDRA Version used will be presented in the title of the related TLFs.

Only treatment emergent adverse events (TEAEs) will be summarized, while all AEs will be listed. TEAE is defined as an AE observed after starting administration of the study treatment and within 30 days after the last dose of the last administered component of study treatment. A drug-related TEAE is defined as any TEAE with a reasonable possibility that the event may have been caused by any component of the study drug by the investigator. Serious TEAE summaries include both investigator-assessed and Astellas upgraded SAEs. The non-TEAEs (except SAEs) will be listed only.

Drug related TEAE will be summarized by related to any component of study drug and by each component of study drug.

An overview summary table will include the following details:

- Number and percentage of subjects with TEAEs,
- Number and percentage of subjects with drug-related TEAEs
- Number and percentage of subjects with serious TEAEs,
- Number and percentage of subjects with serious drug-related TEAEs,
- Number and percentage of subjects with TEAEs leading to interruption of any component of study drug and by component
- Number and percentage of subjects with TEAEs leading to permanent discontinuation of any component of study drug and by component
- Number and percentage of subjects with drug-related TEAEs leading to permanent discontinuation of any component of study drug and by component
- Late adverse event occurred beyond 30 days from the last study treatment (all components)

- Number and percentage of subjects with NCI CTCAE grade 3 or higher TEAEs
- Number and percentage of subjects with drug-related NCI CTCAE grade 3 or higher TEAEs
- Number of deaths from first dose of study drug up to 30 days after the last dose of the last administered component of study drug
- Number and percentage of subjects with TEAE leading to death
- Number and percentage of subjects with drug-related TEAE leading to death
- Number of all deaths up to analysis cutoff date

The number and percentage of subjects with TEAEs, as classified by SOC and PT will be summarized. Summaries will be provided for:

- TEAEs
- Drug-related TEAEs,
- AEs collected after 30 days post last dose of study drug,
- Serious TEAEs,
- Serious AEs collected after 30 days post last dose of study drug,
- Drug-related serious TEAEs,
- Number and percentage of subjects with TEAEs leading to interruption of any component of study drug and by component
- TEAEs leading to permanent discontinuation of any component of study drug and by component,
- Drug-related TEAEs leading to permanent discontinuation of any component of study drug and by component,
- TEAEs excluding serious adverse events that have a frequency of >=10% in any treatment arm, and
- TEAE with NCI CTCAE Grade 3 or higher
- Drug-related TEAE with NCI CTCAE Grade 3 or higher
- Drug-related TEAEs with treatment group difference in incidence (Arm A - Arm B) >10%
- Drug-related serious TEAEs with treatment group difference in incidence (Arm A - Arm B) >5%

The number and percentage of subjects with TEAEs and TEAEs leading to death, as classified by PT only, will be summarized by treatment group and overall.

AE summary tables will include subject counts as opposed to AE counts, except for serious TEAE and TEAE leading to death where AE counts will also be presented. If a subject

experiences more than one episode of a particular AE, that subject will be counted only once for that event. If a subject has more than one AE that code to the same preferred term, the subject will be counted only once for that preferred term. Similarly, if a subject has more than one AE within a body system, the subject will be counted only once in that body system.

The number and percentage of subjects with TEAEs, as classified by SOC and PT, will also be summarized by NCI-CTCAE severity grade and by relationship to study drug. Drug-related TEAEs will be presented in a similar way by severity grade only. If an adverse event changes in severity grade or relationship, then the subject will be counted only once with the worst severity grade and highest degree of relationship. If a subject has an event more than once with missing severity grade and non-missing severity grade, then the subject will be counted as the highest non-missing grade. If a subject has an event more than once with missing relationship and non-missing relationship, then the subject will be counted as "related".

The number and percentage of subjects with TEAEs of interest [listed below], as classified by SOC and PT, will also be summarized. The list of adverse events of interest to be summarized may change during the course of the study due to ongoing pharmacovigilance. It will be finalized before the database hard lock.

6.5.2 AE of Special interest

The list of adverse events of interest to be summarized may change during the course of the study due to ongoing pharmacovigilance. AEs of special interest will include the following with a NCI CTCAE Grade 3 or higher:

- Nausea and vomiting and abdominal pain based on PT terms
- Hypersensitivity reactions based on HS SMQ
- Infusion-related reactions (IRRs)
- Anemia
- Neutropenia

The number and percentage of subjects with AESI(AE of Special Interest), as classified by SOC and PT will be summarized. Summaries will be provided for:

- AESI
- Serious AESI
- AESI leading to permanent discontinuation
- AESI leading to dose interruption
- AESI leading to death

6.5.3 Clinical Laboratory Evaluation

Quantitative values evaluated by the central laboratory including hematology, biochemistry, urinalysis and coagulation will be summarized using mean, standard deviation, minimum, maximum and median by treatment group at each visit. Additionally, a within-subject change will be calculated as the post-baseline measurement minus the baseline measurement and

summarized in the same way. Frequency tabulations of selected qualitative clinical laboratory variables (i.e., urinalysis) will be presented by treatment arm at each visit.

Central laboratory results will also be graded using NCI-CTCAE, where possible. Laboratory parameters that have criteria available for both low and high values, i.e., hypo- and hyper-, will be summarized for both criteria. The same subject can be counted for both criteria if the subject has different laboratory values meeting each criterion. NCI-CTCAE grade of laboratory results will be summarized by number and percentage of subjects for each visit. Shift table of NCI-CTCAE grade change from baseline to each post-baseline visit will be presented by treatment arm and visit. Shift table of NCI-CTCAE grade change from baseline to worst post-baseline grade will also be presented by treatment arm. Number and percent of subjects with treatment-emergent NCI-CTCAE grade of 3 or 4 laboratory results will be presented by laboratory parameter. "Treatment-emergent NCI-CTCAE grade of 3 or 4 laboratory results" are defined as: subject having a maximum NCI-CTCAE grade of 3 or 4 post baseline for a parameter and that grade is higher than the subject's baseline grade for that parameter (or his/her baseline grade is missing).

The list of laboratory parameters to be summarized may change during the course of the study due to ongoing pharmacovigilance. It will be finalized before the database hard lock.

6.5.3.1 Liver Safety Assessment

The liver safety assessments will be summarized by the following categories below based on the measurements from Alkaline Phosphatase (ALP), Alanine Transaminase (ALT), total bilirubin, Aspartate Transaminase (AST) and their combination as defined. These parameters will be measurement from central laboratory.

The subject's highest value post-baseline of each parameter will be used.

- ALT: > 3xULN, > 5xULN, > 10xULN, > 20xULN
- AST: > 3xULN, > 5xULN, > 10xULN, > 20xULN
- ALT or AST: > 3xULN, > 5xULN, > 10xULN, > 20xULN
- ALP: > 1.5xULN
- Total Bilirubin: > 2xULN
- (ALT or AST > 3xULN) and Total Bilirubin > 2xULN
- (ALT or AST > 3xULN) and Total Bilirubin > 2xULN and ALP < 2xULN

The last 2 criteria where 2 or more parameters are evaluated will use the measurements on the same day or up to 1 day apart. The number and percentage of subjects meeting the criteria post-baseline will be summarized by treatment arm.

6.5.4 Vital Signs

Vital signs will be summarized using mean, standard deviation, minimum, maximum and median by treatment arm and visit. Additionally, a within-subject change will be calculated per visit as the post-baseline measurement minus the baseline measurement and summarized by treatment group and visit. For serial vital signs measured values and within-subject change from pre-dose value on that visit will be summarized by visit and timepoint in separate tables.

Tables for potentially clinically significant vital signs will be generated using baseline value and highest value obtained during treatment for each subject.

The following potentially clinically significant criteria are defined for each parameter:

**Vital Sign Variable**	**Criteria**
SBP	≥180 mmHg AND ≥20 mmHg change from baseline
SBP	≤ 80 mmHg
DBP	≥105 mmHg AND ≥15 mmHg change from baseline
Pulse Rate	≥120 bpm AND ≥15 bpm change from baseline

6.5.5 Electrocardiograms

ECG variables will be summarized using mean, standard deviation, minimum, maximum and median for each treatment group at each visit, including changes from baseline.

Number and percentage of subjects with normal and abnormal results for the overall interpretation will be tabulated by treatment group and time point. A shift analysis table showing shift in overall ECG interpretation from baseline to each time point will be provided. Percent of subjects on different kind of abnormality will also be reported.

The QT interval corrected by Fridericia's Correction formula (QTcF interval) will be summarized using frequency tables for values of clinical importance using the range criteria below.

**QTcF Interval Criteria**	**QTcF Interval Value (msec)**
Normal	≤ 450
Borderline	> 450 to <=480
Prolonged	> 480 to <=500
Clinically significant	> 500

QTcF interval: Fridericia-corrected QT interval

Cumulative tabulation for >450, >480 and >500 msec will also be presented.

The QTcF interval will also be summarized by the frequencies of subjects with a change from baseline of clinical importance using the criteria identified below.

**Variable**	**Change from Baseline**
QTcF Interval (msec)	<=0<br>>0 to <=30<br>>30 to <=60<br>> 60

QTcF interval: Fridericia-corrected QT interval

Baseline value is from Cycle 1 Day 1 pre-dose assessment. Cumulative tabulation for >0, >30 and >60 msec will also be presented.

6.5.6 ECOG Performance Status

Number and percent of subjects of for each ECOG performance status grade will be presented at each visit. The change from baseline to each post baseline visit will be summarized. Negative change scores indicate an improvement. Positive change scores indicate a decline in performance. Shift tables of ECOG performance status change from baseline to worst post-baseline grade will also be presented.

6.5.7 Physical Examination

Weight and change from baseline will be summarized by treatment arm and visit.

6.5.8 Pregnancy

A listing of all pregnancy tests will be provided.

6.6 Analysis of Pharmacokinetics

Descriptive statistics (e.g., N, mean, standard deviation, minimum, median, maximum, coefficient of variation [CV], geometric mean, and geometric CV) will be provided for serum concentrations of Zolbetuximab by scheduled sampling visit and time.

Trough concentrations versus visit profile will be described using box and whisker plots.

Additional model-based analyses may be performed and reported separately.

6.7 Subgroup Analyses

PFS, ORR and DOR based on IRC assessments and OS will be summarized for the following subgroups:

- Age group 1: <=65, >65 years
- Age group 2: <=75, >75 years
- Sex: male, female
- Region: Asia, Non-Asia
- Number of metastatic sites: 0-2, ≥ 3
- Prior gastrectomy (total or partial): No, Yes
- Histology (tumor type): diffuse vs. intestinal vs. mixed/other
- Tumor location: Gastric vs GEJ; Gastric proximal vs. Gastric distal ; GEJ proximal vs GEJ distal;
- Country: Japan vs non-Japan; China vs non China

Forest plots for PFS and OS will be produced to summarize the treatment effect (HR) across subgroups. In addition, Japan vs. non-Japan will be added to the forest plots.

6.8 Other Analyses

6.8.1 Immunogenicity

Immunogenicity of Zolbetuximab will be summarized using the frequency of ADA positive subjects. The potential relationship between Zolbetuximab immunogenicity and Zolbetuximab pharmacokinetics, efficacy, safety profile in subjects may be assessed.

6.8.2 Exploratory Biomarkers

Biomarkers may be summarized graphically or descriptively, and summary statistics may be tabulated. Associations between biomarkers and clinical (e.g., efficacy, safety, pharmacodynamics, or pharmacokinetics) measures may be performed on subjects who have sufficient baseline and on-study measurements to provide interpretable results for specific parameters. Analysis will be further described in a biomarker SAP.

6.9 Interim Analysis (and Early Discontinuation of the Clinical Study)

To evaluate whether Zolbetuximab + CAPOX (Arm A) is beneficial compared to the concurrent placebo + CAPOX (Arm B) while the study is ongoing, a formal OS interim analysis is planned when the final PFS analysis occurs with the pre-specified number of PFS events. A group sequential design using the O'Brien-Fleming type alpha-spending function [Lan & DeMets, 1983] will be utilized to control the overall 1-sided 0.025 significance level (East®) for the OS endpoint. The OS interim and final analyses will be performed only if primary PFS analysis is significant.

The IDMC may recommend terminating the trial for favorable results at the formal efficacy interim analysis using OS. The IDMC may also recommend terminating the trial for negative PFS results. In the case of favorable results, the 1-sided significance level for superiority is 0.0074 for the interim OS analysis and 0.0228 for the final OS analysis. If the 1-sided P-value of the interim analysis is less than 0.0074, the IDMC may recommend terminating the trial for success. If the study is not stopped after the interim analysis, a final OS analysis will occur after 100% of the planned death events have been observed. Note that the 0.0074 alpha boundary is based on an information factor of 70% (270 death out of planned 386 events) observed at the PFS final/OS interim analysis and may be adjusted prior to interim analysis if the number of observed deaths deviates from that number.

The interim analysis will be run by an Independent Data Analysis Center (IDAC) for IDMC. In addition, safety data reviews during the trial will be conducted by the IDMC on a periodic basis. For example, the IDMC will have its first safety data review 6 weeks after the 40th subject has been randomized and on study drug for 2 cycle (6 weeks) and meetings will be conducted regularly thereafter, as specified in the IDMC Charter.

The full procedures for IDMC safety review will be described in a separate IDMC Charter. An interim analysis plan (IAP) will describe specific analyses to be presented for safety and the efficacy interim reviews.

6.10 Additional Conventions

6.10.1 Analysis Visits

Nominal visits as recorded on eCRF will be used in the by-visit summaries. Values from unscheduled visits will be included in the summary of extreme cases (e.g., summary of worst value post-baseline, summary of minimum value post-baseline, summary of maximum value post-baseline). For efficacy endpoints, all values (scheduled and unscheduled) will be included in the analysis.

For time course plots, actual study day (calculated using actual visit date – date of first dose +1) will be used.

6.10.2 Imputation Rules for Incomplete Dates

Every effort will be made to resolve missing or incomplete dates for adverse events and concomitant medications. If a partial date cannot be resolved, the most conservative imputation methods will be used to complete the missing information. As a general rule, if the month or year is missing, imputation should be avoided if possible. More details on date imputation, if needed, would be placed in the TLF specifications.

7 REVISION AND RATIONALE

7.1 List of Changes in SAP Version 2.0 from Version 1.0 (if applicable)

The changes from the approved SAP Version 1.0 (Dated dd-MMM-yyyy) to Version 2.0 that impact analyses are listed with the rationale in the table below.

SAP Section(s)	Description of Change(s)	Rationale

8 REFERENCES

ICH Harmonized Tripartite Guideline E 3. Structure and Content of Clinical Study Reports, November 1995. (www.ich.org; Guidelines; "Efficacy" Topics)

ICH Harmonized Tripartite Guideline E 9. Statistical Principles for Clinical Trials, February 1998. (www.ich.org; Guidelines; "Efficacy" Topics)

Fayers PM, Aaronson NK, Bjordal K, Groenvold M, Curran D, Bottomley A, on behalf of the EORTC Quality of Life Group. The EORTC QLQ-C30 Scoring Manual (3rd Edition). Published by: European Organisation for Research and Treatment of Cancer. Brussels 2001.

Giesinger JM et al. Replication and validation of higher order models demonstrated that a summary score for the EORTC QLQ-C30 is robust. J. Clin. Epidemiol. 69:79-88, 2016.

9 APPENDICES

9.1 EORTC QLQ-C30 questionnaire (version 3)

	Not at All	A Little	Quite a Bit	Very Much
1. Do you have any trouble doing strenuous activities, like carrying a heavy shopping bag or a suitcase?	1	2	3	4
2. Do you have any trouble taking a long walk?	1	2	3	4
3. Do you have any trouble taking a short walk outside of the house?	1	2	3	4
4. Do you need to stay in bed or a chair during the day?	1	2	3	4
5. Do you need help with eating, dressing, washing yourself or using the toilet?	1	2	3	4

**During the past week:**	Not at All	A Little	Quite a Bit	Very Much
6. Were you limited in doing either your work or other daily activities?	1	2	3	4
7. Were you limited in pursuing your hobbies or other leisure time activities?	1	2	3	4
8. Were you short of breath?	1	2	3	4
9. Have you had pain?	1	2	3	4
10. Did you need to rest?	1	2	3	4
11. Have you had trouble sleeping?	1	2	3	4
12. Have you felt weak?	1	2	3	4
13. Have you lacked appetite?	1	2	3	4
14. Have you felt nauseated?	1	2	3	4
15. Have you vomited?	1	2	3	4
16. Have you been constipated?	1	2	3	4

Please go on to the next page

**During the past week:**	**Not at All**	**A Little**	**Quite a Bit**	**Very Much**
17. Have you had diarrhea?	1	2	3	4
18. Were you tired?	1	2	3	4
19. Did pain interfere with your daily activities?	1	2	3	4
20. Have you had difficulty in concentrating on things, like reading a newspaper or watching television?	1	2	3	4
21. Did you feel tense?	1	2	3	4
22. Did you worry?	1	2	3	4
23. Did you feel irritable?	1	2	3	4
24. Did you feel depressed?	1	2	3	4
25. Have you had difficulty remembering things?	1	2	3	4
26. Has your physical condition or medical treatment interfered with your family life?	1	2	3	4
27. Has your physical condition or medical treatment interfered with your social activities?	1	2	3	4
28. Has your physical condition or medical treatment caused you financial difficulties?	1	2	3	4

For the following questions please circle the number between 1 and 7 that best applies to you

29. How would you rate your overall health during the past week?

1	2	3	4	5	6	7
Very poor						Excellent

30. How would you rate your overall quality of life during the past week?

1	2	3	4	5	6	7
Very poor						Excellent

9.2 EORTC QLQ-30 Scoring

Scoring the EORTC QLQ-C30 version 3.0

Table 1: Scoring the QLQ-C30 version 3.0

	Scale	Number of items	Item range*	**Version 3.0** Item numbers	Function scales
**Global health status / QoL**					
Global health status/QoL (revised)†	QL2	2	6	29, 30	
**Functional scales**					
Physical functioning (revised)†	PF2	5	3	1 to 5	F
Role functioning (revised)†	RF2	2	3	6, 7	F
Emotional functioning	EF	4	3	21 to 24	F
Cognitive functioning	CF	2	3	20, 25	F
Social functioning	SF	2	3	26, 27	F
**Symptom scales / items**					
Fatigue	FA	3	3	10, 12, 18	
Nausea and vomiting	NV	2	3	14, 15	
Pain	PA	2	3	9, 19	
Dyspnoea	DY	1	3	8	
Insomnia	SL	1	3	11	
Appetite loss	AP	1	3	13	
Constipation	CO	1	3	16	
Diarrhoea	DI	1	3	17	
Financial difficulties	FI	1	3	28	

* *Item range* is the difference between the possible maximum and the minimum response to individual items; most items take values from 1 to 4, giving *range* = 3.
† (revised) scales are those that have been changed since version 1.0, and their short names are indicated in this manual by a suffix "2" – for example, PF2.

For all scales, the *RawScore*, *RS*, is the mean of the component items:

$$RawScore = RS = \left(I_1 + I_2 + ... + I_n\right)/n$$

Then for **Functional scales**:

$$Score = \left\{1 - \frac{(RS-1)}{range}\right\} \times 100$$

and for **Symptom scales / items** and **Global health status / QoL:**

$$Score = \left\{(RS-1)/range\right\} \times 100$$

Examples:

Emotional functioning

$$RawScore = (Q_{21} + Q_{22} + Q_{23} + Q_{24})/4$$
$$\text{EF } Score = \{1 - (RawScore - 1)/3\} \times 100$$

Fatigue

$$RawScore = (Q_{10} + Q_{12} + Q_{18})/3$$
$$\text{FA } Score = \{(RawScore - 1)/3\} \times 100$$

9.3 EORTC QLQ-OG25 questionnaire

EORTC IL11 – QLQ–OG25+2

Patients sometimes report that they have the following symptoms or problems. Please indicate the extent to which you have experienced these symptoms or problems during the past week. Please answer by circling the number that best applies to you.

During the past week:	Not at all	A little	Quite a bit	Very much
31. Have you had problems eating solid foods?	1	2	3	4
32. Have you had problems eating liquidised or soft foods?	1	2	3	4
33. Have you had problems drinking liquids?	1	2	3	4
34. Have you had trouble enjoying your meals?	1	2	3	4
35. Have you felt full up too quickly after beginning to eat?	1	2	3	4
36. Has it taken you a long time to complete your meals?	1	2	3	4
37. Have you had difficulty eating?	1	2	3	4
38. Have you had acid indigestion or heartburn?	1	2	3	4
39. Has acid or bile coming into your mouth been a problem?	1	2	3	4
40. Have you had discomfort when eating?	1	2	3	4
41. Have you had pain when you eat?	1	2	3	4
42. Have you had pain in your stomach area?	1	2	3	4
43. Have you had discomfort in your stomach area?	1	2	3	4
44. Have you been thinking about your illness?	1	2	3	4
45. Have you worried about your health in the future?	1	2	3	4
46. Have you had trouble with eating in front of other people?	1	2	3	4
47. Have you had a dry mouth?	1	2	3	4
48. Have you had problems with your sense of taste?	1	2	3	4
49. Have you felt physically less attractive as a result of your disease or treatment?	1	2	3	4

During the past week:	Not at all	A little	Quite a bit	Very much	Not applicable
50. Have you had difficulty swallowing your saliva?	1	2	3	4	
51. Have you choked when swallowing?	1	2	3	4	
52. Have you coughed?	1	2	3	4	
53. Have you had difficulty talking?	1	2	3	4	
54. Have you worried about your weight being too low?	1	2	3	4	
55. Answer this question only if you lost any hair: If so, were you upset by the loss of your hair?	1	2	3	4	N/A
56. Have you had trouble with bile or acid coming into your mouth?	1	2	3	4	
57. Have you had trouble with belching?	1	2	3	4	

9.4 EQ-5D-5L Scoring

2. Scoring the EQ-5D-5L descriptive system

The EQ-5D-5L descriptive system should be scored, for example, as follows:

Under each heading, please tick the ONE box that best describes your health TODAY

MOBILITY

I have no problems in walking about	✓
I have slight problems in walking about	☐
I have moderate problems in walking about	☐
I have severe problems in walking about	☐
I am unable to walk about	☐

SELF-CARE

I have no problems washing or dressing myself	☐
I have slight problems washing or dressing myself	✓
I have moderate problems washing or dressing myself	☐
I have severe problems washing or dressing myself	☐
I am unable to wash or dress myself	☐

USUAL ACTIVITIES *(e.g. work, study, housework, family or leisure activities)*

I have no problems doing my usual activities	☐
I have slight problems doing my usual activities	☐
I have moderate problems doing my usual activities	✓
I have severe problems doing my usual activities	☐
I am unable to do my usual activities	☐

PAIN / DISCOMFORT

I have no pain or discomfort	☐
I have slight pain or discomfort	☐
I have moderate pain or discomfort	☐
I have severe pain or discomfort	✓
I have extreme pain or discomfort	☐

ANXIETY / DEPRESSION

I am not anxious or depressed	☐
I am slightly anxious or depressed	☐
I am moderately anxious or depressed	☐
I am severely anxious or depressed	☐
I am extremely anxious or depressed	✓

Levels of perceived problems are coded as follows:

Level 1 is coded as a '1'

Level 2 is coded as a '2'

Level 3 is coded as a '3'

Level 4 is coded as a '4'

Level 5 is coded as a '5'

This example identifies the health state '12345'.

NB: There should be only ONE response for each dimension

*NB: **Missing values** can be coded as '9'.*

*NB: **Ambiguous values** (e.g. 2 boxes are ticked for a single dimension) should be treated as missing values.*

- We would like to know how good or bad your health is TODAY.
- This scale is numbered from 0 to 100.
- 100 means the <u>best</u> health you can imagine.
  0 means the <u>worst</u> health you can imagine.
- Mark an X on the scale to indicate how your health is TODAY.
- Now, please write the number you marked on the scale in the box below.

YOUR HEALTH TODAY = [ ]

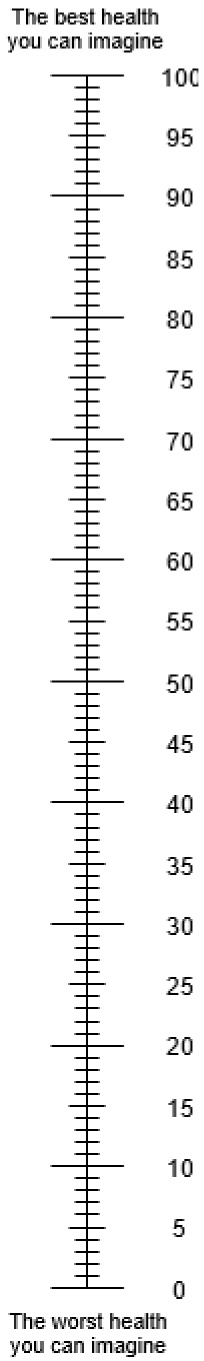

9.5 Global Pain

The Global Pain item is below (the eCOA team can format with radial dials or check boxes)

Please rate your pain by selecting the one number that best describes your pain at its worst in the last 24 hours

0	1	2	3	4	5	6	7	8	9	10
No Pain										Pain as bad as you can imagine

9.6 Health Resource Utilization

1. Since your last study visit, have you had any visits to the emergency room (ER)?

   ☐ No (if no, go to 4)
   ☐ Yes (if yes, go to 2)

2. Since your last visit, how many emergency room visits have you had?
   ____

3. For each emergency room visit please complete the following:

	Result in a Hospital Admission (more than a 24 hour stay)?	Length of stay in hospital (number of days)
ER Visit 1	Yes/No	
ER Visit 2	Yes/No	
ER Visit 3	Yes/No	

4. Since your last study visit, have you had any hospital admissions (more than a 24 hour stay) that occurred <u>without</u> first going to the emergency room (ER)?

   ☐ No (if no, go to 7)
   ☐ Yes (if yes, go to 5)

5. How many hospital admissions (more than 24 hour stay; without previous ER transferal)? ___________

6. For each hospital admission (more than 24 hour stay; without previous ER transferal) visit please complete the following:

	Length of stay in hospital (number of days)
Hospital Visit 1	
Hospital Visit 2	
Hospital Visit 3	

7. Since your last study visit, have you had any visits to a general practitioner (primary care physician)?

   ☐ No (if no, go to 9)
   ☐ Yes (if yes, go to 8)

8. How many visits have you had to a general practitioner (primary care physician)? ___________

9. Since your last study visit, did you have any visits to a specialist physician (e.g., oncologist, rheumatologist, endocrinologist, orthopedic surgeon, etc.)?

   ☐ No
   ☐ Yes (if yes, go to 10)

How many visits have you had to a specialist physician (e.g., oncologist, rheumatologist, endocrinologist, orthopedic surgeon, etc.)?

9.7 Author and Approver Signatories

E-signatures are attached at end of document (next page). Wet signatures, if any, are provided on this page.

Author: ______________________ Date: ____________

PPD

Approved by: ______________________ Date: ____________

PPD

Approved by: ______________________ Date: ____________

PPD

STATISTICAL ANALYSIS PLAN

Final Version 2.0, 13-Sep-2022

A Phase 3, Global, Multi-Center, Double-Blind, Randomized, Efficacy Study of Zolbetuximab (IMAB362) Plus CAPOX Compared with Placebo Plus CAPOX as First-line Treatment of Subjects with Claudin (CLDN) 18.2-Positive, HER2-Negative, Locally Advanced Unresectable or Metastatic Gastric or Gastroesophageal Junction (GEJ) Adenocarcinoma

ISN/Protocol 8951-CL-0302

IND 129598

EudraCT 2018-000519-26

Sponsor:

Astellas Pharma Global Development, Inc. (APGD)
1 Astellas Way
Northbrook, IL 60062

This document contains confidential information which is the intellectual property of Astellas. By accepting or reviewing this document, you agree to hold this information in confidence and not copy or disclose it to others or use it for unauthorized purposes except (1) as otherwise agreed to in writing; (2) where required by applicable law; (3) where disclosure is directly related to the care and safety of the research participant; and (4) where disclosure of such information is made to a member of the investigator's team who agrees to hold this information in confidence.

Table of Contents

I. LIST OF ABBREVIATIONS AND KEY TERMS 5

1 INTRODUCTION 8

2 STUDY OBJECTIVES AND DESIGN 8

2.1 Study Objectives 8

2.1.1 Primary Objective 8

2.1.2 Secondary Objectives 8

2.1.3 Exploratory Objectives 8

2.2 Study Design 9

2.3 Randomization 9

3 SAMPLE SIZE 9

4 ANALYSIS SETS 10

4.1 Full Analysis Set 10

4.2 Safety Analysis Set 10

4.3 Pharmacokinetics Analysis Set (PKAS) 10

5 ANALYSIS ENDPOINTS 10

5.1 Primary Efficacy Endpoint 10

5.2 Secondary Efficacy Endpoints 11

5.3 Exploratory Efficacy Endpoints 11

5.4 Safety Endpoints 12

5.4.1 AE 12

5.4.2 Clinical Laboratory Variables 12

5.4.3 Vital Signs 13

5.4.4 12-lead electrocardiogram (ECG) 13

5.4.5 ECOG performance score 13

5.4.6 Physical examination 13

5.5 Other Endpoints 13

5.5.1 Pharmacokinetic Endpoints 13

5.5.2 Immunogenicity 13

5.5.3 Biomarkers Endpoints 13

6 STATISTICAL METHODOLOGY 13

6.1 General Considerations 13

6.2 Study Population 14
6.2.1 Disposition of Subjects 14
6.2.2 Protocol Deviations 15
6.2.3 Demographic and Other Baseline Characteristics 15
6.2.4 Transfusions 15
6.2.5 Previous and Concomitant Medications 16
6.2.6 Non-medication Therapies 16
6.2.7 New Anti-Cancer Therapies 16
6.2.8 Prior Radiation Therapy 16
6.2.9 Prior Procedures for Primary Cancer 16
6.2.10 Prior Cancer Chemotherapy 16
6.3 Study Drug Exposure and Compliance 16
6.4 Analysis of Efficacy 17
6.4.1 Analysis of Primary Efficacy Endpoint (PFS) 18
6.4.2 Analysis of Secondary Efficacy Endpoints 20
6.4.3 Analysis of Exploratory Efficacy Endpoints 28
6.5 Analysis of Safety 30
6.5.1 Adverse Events 31
6.5.2 AE of Special interest 33
6.5.3 Clinical Laboratory Evaluation 33
6.5.4 Vital Signs 34
6.5.5 Electrocardiograms 35
6.5.6 ECOG Performance Status 35
6.5.7 Physical Examination 36
6.5.8 Pregnancy 36
6.6 Analysis of Pharmacokinetics 36
6.7 Subgroup Analyses 36
6.8 Other Analyses 36
6.8.1 Immunogenicity 36
6.8.2 Exploratory Biomarkers 37
6.9 Interim Analysis (and Early Discontinuation of the Clinical Study) 37
6.10 Additional Conventions 38
6.10.1 Analysis Visits 38
6.10.2 Imputation Rules for Incomplete Dates 38

7 REVISION AND RATIONALE 39

7.1 List of Changes in SAP Version 2.0 from Version 1.0 (if applicable) 39

8 REFERENCES 40

9 APPENDICES 41

9.1 EORTC QLQ-C30 questionnaire (version 3) 41

9.2 EORTC QLQ-30 Scoring 43

9.3 EORTC QLQ-OG25 questionnaire 44

9.4 EQ-5D-5L Scoring 45

9.5 Global Pain 47

9.6 Health Resource Utilization 47

9.7 Author and Approver Signatories 49

I. LIST OF ABBREVIATIONS AND KEY TERMS

List of Abbreviations

**Abbreviations**	**Description of abbreviations**
ADA	anti-drug antibody
AE	adverse event
AESI	adverse event of special interest
ALP	alkaline phosphatase
ALT	alanine aminotransferase (GPT)
AST	aspartate aminotransferase (GOT)
ATC	Anatomical Therapeutic Chemical Classification System
BMI	body mass index
BSA	body surface area
CAPOX	Capecitabine and Oxaliplatin
C1D1	Cycle 1 Day 1
CI	confidence interval
CLDN	Claudin
C_{trough}	trough concentration
CMH	Cochran-Mantel-Haenszel
CMQ	Customized Medical Dictionary for Regulatory Activities (MedDRA) Query
CR	complete response
CSR	Clinical study report
CTCAE	Common Terminology Criteria For Adverse Events
DCR	disease control rate
DOR	duration of response
ECG	electrocardiogram
ECOG	Eastern Cooperative Oncology Group
eCRF	electronic case report form
EORTC	European Organization for Research and Treatment of Cancer
EQ-5D-5L	EuroQOL Five Dimensions Questionnaire 5L
FAS	full analysis set
GEJ	gastroesophageal junction
GP	Global Pain
HER2	human epidermal growth factor receptor 2
HR	hazard ratio
HRQoL	health-related quality of life
HRU	Health Resource Utilization
IDAC	independent data analysis center
IDMC	independent data monitoring committee
INR	international normalized ratio

Abbreviations	Description of abbreviations
IRC	independent review committee
IRR	infusion-related reaction
IRT	interactive response technology
ISN	international study number
IV	Intravenous, intravenously
NCI	National Cancer Institute
NE	not evaluable
ORR	objective response rate
OS	overall survival
PD	progressive disease
PFS	progression free survival
PFS2	progression free survival following subsequent anti-cancer treatment
PGx	pharmacogenomics
PKAS	pharmacokinetics analysis set
PR	partial response
PT	preferred term
QLQ-C30	Quality of Life Questionnaire - Core Questionnaire
QLQ-OG25	Quality of Life Questionnaire - Oesophago-Gastric Module 25 (OG-25)
QoL	Quality of life
QTc	QT interval corrected
QTcF	Fridericia-corrected QT interval
RECIST	Response Evaluation Criteria in Solid Tumors
SAE	serious adverse event
SAF	safety analysis set
SAP	statistical analysis plan
SD	stable disease
SOC	system organ class
SOD	Sum of diameters
SSQ	Standardized Search Query
SMQ	Standardized MedDRA Query
TEAE	treatment-emergent adverse event
TSH	thyroid stimulating hormone
TTP	time to progression
ULN	upper limit of normal
VAS	visual analog scale
WHO	World health organization

List of Key Terms

Terms	Definition of terms
Baseline	Assessments of subjects as they enter a trial before they receive any treatment.
Endpoint	Variable that pertains to the efficacy or safety evaluations of a trial.
Enroll	To register or enter a subject into a clinical trial. NOTE: Once a subject has been randomized, the clinical trial protocol applies to the subject.
Intervention	The drug, device, therapy or process under investigation in a clinical study that is believed to have an effect on outcomes of interest in a study (e.g., health-related quality of life, efficacy, safety, pharmacoeconomics).
Investigational period	Period of time where major interests of protocol objectives are observed, and where the test drug or comparative drug (sometimes without randomization) is usually given to a subject, and continues until the last assessment after completing administration of the test drug or comparative drug.
Post investigational period	Period of time after the last assessment of the protocol. Follow-up observations for sustained adverse events and/or survival are done in this period.
Randomization	The process of assigning trial subjects to treatment or control groups using an element of chance to determine assignments in order to reduce bias.
Screening	A process of active consideration of potential subjects for enrollment in a trial.
Screen failure	Potential subject who did not meet 1 or more criteria required for participation in a trial.
Screening period	Period of time before entering the investigational period, usually from the time when a subject signs the consent until just before the test drug or comparative drug (sometimes without randomization) is given to a subject.
Study period	Period of time from the first site initiation date to the last site completing the study.
Study treatment	Includes Zolbetuximab/placebo and all components of CAPOX
Variable	Any entity that varies; any attribute, phenomenon or event that can have different qualitative or quantitative values.

1 INTRODUCTION

This Statistical Analysis Plan (SAP) contains technical and detailed elaboration of the principal features of the analysis described in the protocol, and includes procedures for executing the statistical analysis to fulfil the objectives of the study. The final SAP will be approved prior to database hard lock for the final PFS (and interim OS) analysis.

Changes that affected the statistical analyses from the planned analyses in the SAP will be documented in the Clinical Study Report (CSR).

2 STUDY OBJECTIVES AND DESIGN

2.1 Study Objectives

2.1.1 Primary Objective

The primary objective is to evaluate the efficacy of Zolbetuximab plus CAPOX compared with placebo plus CAPOX (as first-line treatment) as measured by progression free survival (PFS) in subjects with Claudin (CLDN)18.2 positive, human epidermal growth factor receptor 2 (HER2)–negative locally advanced unresectable or metastatic gastric and gastroesophageal junction (GEJ) adenocarcinoma.

2.1.2 Secondary Objectives

The secondary objectives are:

- To evaluate efficacy as measured by Overall Survival (OS) as a key secondary objective
- To evaluate the physical function (PF), OG25-Pain and GHS/QoL scores as measured by European Organization for Research and Treatment of Cancer (EORTC) as a key secondary objective
- To evaluate efficacy as measured by Objective Response Rate (ORR)
- To evaluate efficacy as measured by Duration of Response (DOR)
- To evaluate safety and tolerability of Zolbetuximab
- To further evaluate other health-related quality of life (HRQoL) using additional parameters as measured by EORTC QLQ-C30 and QLQ-OG25 plus STO22 Belching subscale, Global Pain (GP) and the EuroQOL Five Dimensions Questionnaire 5L (EQ-5D-5L) questionnaires
- To evaluate the pharmacokinetics of Zolbetuximab
- To evaluate the immunogenicity profile of Zolbetuximab

2.1.3 Exploratory Objectives

The exploratory objectives are:

- To evaluate efficacy as measured by Time to Progression (TTP)
- To evaluate PFS following subsequent anti-cancer treatment (PFS2)
- To evaluate Disease Control Rate (DCR)
- To evaluate potential genomic and/or other biomarkers that may correlate with treatment outcome to Zolbetuximab and CAPOX.

- To evaluate Health Resource Utilization (HRU)

2.2 Study Design

This global, multi-center, double-blind, 1:1 randomized, phase 3 study will evaluate efficacy of Zolbetuximab plus CAPOX versus placebo plus CAPOX as first-line treatment in subjects with CLDN18.2-positive, HER2-negative locally advanced unresectable or metastatic gastric and GEJ adenocarcinoma.

PFS as assessed by the Independent Review Committee (IRC) is the primary outcome. Secondary outcomes include OS, time to confirmed deterioration (TTCD) in PF, OG25-PA and GHS/QoL, ORR, DOR, safety and tolerability, other HRQoL, pharmacokinetic and the immunogenicity profile of Zolbetuximab. Exploratory outcomes include TTP, PFS2, DCR, biomarkers and HRU.

One interim analysis and one final analysis are planned for OS, while only one analysis is planned for PFS as the final analysis. The OS interim analysis will occur at the same time of the final PFS analysis (after pre-specified number of PFS events) and final OS analysis will be performed after the pre-specified number of OS events are observed. Refer to Section 6.9 for details on interim analysis.

Details of the study flow chart, dosing schedule, schedule of assessments are available in the protocol Section V.

2.3 Randomization

Subject randomization will be performed via IRT and treatment is assigned in a 1:1 ratio to:

- Arm A (Zolbetuximab in combination with CAPOX chemotherapy)
- Arm B (placebo in combination with CAPOX chemotherapy)

Prior to the initiation of the study treatment, the unblinded pharmacist/designee will be notified by the IRT system about the randomly assigned treatment. The unblinded pharmacist/designee will dispense the treatment according to the IRT system's assignment. Specific procedures for randomization through the IRT are contained in the IRT manual.

Randomization of subjects will use blocked randomization and be stratified by the following factors:

- Region (Asia vs. Non-Asia)
- Number of Organs with Metastatic Sites (0 to 2 vs. ≥ 3)
- Prior Gastrectomy (Yes vs. No)

3 SAMPLE SIZE

Approximately 500 subjects will be randomized in a 1:1 ratio to receive Zolbetuximab in combination with CAPOX chemotherapy (Arm A) or placebo in combination with CAPOX chemotherapy (Arm B). The planned 300 PFS events during the study will provide 93.4% power to detect a difference in PFS between Arm A (Zolbetuximab + CAPOX) with the assumption of 9 months median PFS time and Arm B (placebo + CAPOX) with the

assumption of 6 months median PFS time (hazard ratio = 0.67) at the overall 1-sided 0.025 significance level. Similarly, the planned 386 OS events during the study will provide 80% power to detect a difference in OS between Arm A (Zolbetuximab + CAPOX) with the assumption of 14.7 months median OS time and Arm B (placebo + CAPOX) with the assumption of 11 months median OS time (hazard ratio = 0.75) at the overall 1-sided 0.025 significance level.

4 ANALYSIS SETS

In accordance with International Conference on Harmonization (ICH) recommendations in guidelines E3 and E9, the following analysis sets will be used for the analyses.

The determination of whether subjects are included or excluded from the safety and efficacy analysis sets will be made prior to database hard-lock and unblinding. Inclusions and exclusions from the pharmacokinetics analysis set (PKAS) may occur after unblinding.

4.1 Full Analysis Set

The Full Analysis Set (FAS) will consist of all subjects who are randomized to 1 of the treatment arms. Subjects would be analyzed according to the treatment arm to which they were randomized to. The FAS will be used for summaries of demographic and baseline characteristics and all efficacy analyses. FAS in this study is identical to intent-to-treat (ITT) set (only the name “FAS” will be used).

4.2 Safety Analysis Set

The safety analysis set (SAF) will consist of all subjects who received at least 1 dose of any study drug (Zolbetuximab/placebo/CAPOX). Subjects would be analyzed according to the actual treatment they received. The SAF will be used for summaries of demographic and baseline characteristics and all safety and tolerability-related variables. In case that SAF and FAS are identical, summaries of demographic and baseline characteristics will not be repeated on SAF.

4.3 Pharmacokinetics Analysis Set (PKAS)

The PKAS consists of the subset of the SAF for which at least one concentration data is available. Additional subjects may be excluded from the PKAS at the discretion of the pharmacokineticist. The PKAS will be used for all summaries of the pharmacokinetic data.

5 ANALYSIS ENDPOINTS

5.1 Primary Efficacy Endpoint

The primary endpoint is PFS, which is defined as the time from the date of randomization until the date of radiological PD (per Response Evaluation Criteria In Solid Tumors [RECIST] 1.1 by independent review committee [IRC]) or death from any cause, whichever is earliest.

5.2 Secondary Efficacy Endpoints

- OS, defined as the time from the date of randomization until the date of death from any cause.
- Time to confirmed deterioration (TTCD) in PF, OG25-PA and GHS/QoL, as collected via EORTC QLQ-C30 and QLQ-OG25 plus STO22 Belching subscale, defined as the time from the date of randomization until the date of first clinically meaningful deterioration, which is also observed at the next consecutive scheduled visit, or followed by drop-out resulting in missing data or death.
- ORR, defined as the proportion of subjects who have a best overall response (BOR) of complete response (CR) or partial response (PR) as assessed by IRC per RECIST 1.1.
- DOR, defined as the time from the date of the first response (CR/PR) until the date of PD as assessed by IRC per RECIST 1.1 or date of death from any cause, whichever is earliest.
- Health-related Quality of Life questionnaires:
    - EORTC QLQ-C30 questionnaire - a 30-item cancer-specific instrument consisting of 5 functional scales (physical, role, emotional, social and cognitive), 9 symptom scales/items and a global health status scale;
    - EORTC QLQ-OG25 questionnaire - a 25-item instrument that evaluates gastric and GEJ cancer-specific symptoms. This module consists of 6 scales: dysphagia (3 items), eating restrictions (4 items), reflux (2 items), odynophagia (2 items), pain and discomfort (2 items) and anxiety (2 items), as well as 10 single items: eating in front of others, dry mouth, trouble with taste, body image, trouble swallowing saliva, choked when swallowing, trouble with coughing, trouble talking, weight loss and hair loss. In addition a belching and a bile or acid coming in your mouth question from the STO-22 follows the OG-25 questionnaire.
    - Global Pain (GP) questionnaire: a single assessment of overall pain.
    - EQ-5D-5L questionnaire: a standardized 6-item instrument that cover 5 main domains (mobility, self-care, usual activities, pain/discomfort and anxiety/depression) and a general visual analog scale (VAS) for health status.

5.3 Exploratory Efficacy Endpoints

The exploratory endpoints are:

- TTP, defined as the time from the date of randomization until the date of PD as assessed by IRC per RECIST 1.1
- PFS2, defined as the time from the date of randomization until the date of PD (per subject's local physician) following subsequent anti-cancer therapy, death from any cause, or start of any other anti-cancer therapy, whichever is earliest.
- DCR, defined as the proportion of subjects who have a BOR of SD, CR or PR as assessed by IRC per RECIST 1.1.
- Potential genomic and/or other biomarkers that may correlate with treatment outcome to Zolbetuximab and CAPOX.

- Health Resource Utilization (HRU) questionnaire: includes number of ER visits, number and duration of hospital stays, and number of doctor office visits that occur outside of the clinical trial.

5.4 Safety Endpoints

Safety and tolerability endpoints include AEs, laboratory test results, vital signs, electrocardiograms (ECGs) and Eastern Cooperative Oncology Group (ECOG) performance status.

5.4.1 AE

AE will be assessed by evaluation of the following variables:

- Treatment-emergent adverse events (TEAEs; frequency, severity, seriousness, and relationship to study drug)
  - TEAE is defined as an adverse event observed after starting administration of the study drug through 30 days after the last dose of study drug.
  - If the adverse event occurs on Day 1 and the onset check box is marked "Onset after first dose of study drug" or the onset check box is left blank, then the adverse event will be considered treatment emergent.
  - If the adverse event occurs on Day 1 and the onset check box is marked "Onset before first dose of study drug", then the adverse event will not be considered treatment emergent.
  - If a subject experiences an event both during the pre-investigational period and during the investigational period, the event will be considered as TEAE only if it is reported with a new start date (i.e., as a new AE).
  - Any AEs with onset dates completely missing will be considered TEAEs in summaries. AEs with partially missing onset dates will be assumed TEAEs unless the available portion of the date indicates that the onset was strictly before start of study medication.
  - A drug-related TEAE is defined as any TEAE with possible relationship to study treatment as assessed by the investigator or with missing assessment of the causal relationship.
- Serious adverse events (SAEs) include adverse events that are flagged as serious by the investigator on eCRF or for which the SAE flag by the investigator on CRF is missing, or upgraded by the Sponsor based on review of the Sponsor's list of Always Serious terms or Important Medical Events.

5.4.2 Clinical Laboratory Variables

Refer to protocol section 5.4.3 for a table of the laboratory tests that will be performed during the conduct of the study. Refer to the Protocol Schedule of Assessments for evaluation schedule.

5.4.3 Vital Signs

Vital signs will include systolic and diastolic blood pressure (mmHg), radial pulse (beats/min) and body temperature. Serial vital signs will be collected during Zolbetuximab dosing visits.

5.4.4 12-lead electrocardiogram (ECG)

A single 12-lead ECG will be performed at the time points outlined in the Protocol Schedule of Assessments. ECGs will be assessed locally.

5.4.5 ECOG performance score

ECOG performance scores will be collected.

5.4.6 Physical examination

Targeted (symptom driven) physical exams should be conducted every 3 weeks on day 1 of each cycle. If clinically significant worsening of findings from baseline is noted at any study visit, the changes will be documented as AEs on the AE eCRF.

5.5 Other Endpoints

5.5.1 Pharmacokinetic Endpoints

PK of Zolbetuximab as measured by C_{trough}.

5.5.2 Immunogenicity

Immunogenicity of Zolbetuximab as measured by the frequency of anti-drug antibody (ADA) positive subjects.

5.5.3 Biomarkers Endpoints

Potential genomic and/or other exploratory biomarkers that may be related to treatment outcome of
Zolbetuximab.

6 STATISTICAL METHODOLOGY

6.1 General Considerations

Continuous data will be summarized descriptively including the number of subjects, mean, standard deviation, median, minimum and maximum. Categorical data will be summarized by frequencies and percentages. Percentages by categories will be based on the number of subjects with no missing data, i.e. the percentages for the non-missing categories will add up to 100%. All non-coded free-text variables will be displayed in data listings only.

Summaries based on FAS (e.g. disposition, baseline characteristics and efficacy endpoints) will be presented by randomized treatment. Safety summaries based on SAF and summaries based on PKAS will be presented by actual treatment received.

All statistical comparisons will be made using one-sided test at the α=0.025 significance level unless specifically stated otherwise. All null hypotheses will be: Arm A is not better than

Arm B, all alternative hypotheses will be: Arm A is better than Arm B, unless specifically stated otherwise.

All data summarization and analyses will be performed using SAS® Version 9.3 or higher on LINUX. Specifications for table, figures, and data listing formats can be found in the TLF specifications document for this study.

Study day for safety assessments (e.g. laboratory assessment, onset of adverse events, vital signs, etc.) will be calculated in reference to the first dose date. For assessments conducted before the first dose, study day will be calculated as (assessment date – first dose date). For assessments conducted on or after the first dose, study day will be calculated as (assessment date – first dose date + 1). Study day for efficacy events (progression, death, tumor responses CR/PR) will be calculated in reference to the randomization date (event/assessment date – randomization date + 1).

For efficacy evaluation (except for PRO analysis), baseline is defined as the last available measurement before randomization. For safety evaluation and PRO analysis, baseline is defined as the last available measurement before the first dose. Unless otherwise specified, all summaries will be presented by treatment arm.

Study drug is defined as any one of the 3 components (Zolbetuximab, capecitabine and oxaliplatin) for Arm A and any one of the 3 components (placebo, capecitabine and oxaliplatin) for Arm B. Date of first dose of study drug is the date of start of infusion of the first component of study drug administered, or oral dosing of Capecitabine, whichever is earlier. Date of last dose of study drug is the date of stop of infusion of the last component of study drug administered or oral dosing of Capecitabine, whichever is later.

All by-visit summaries will use CRF visit (e.g., Cycle 3 Day 15) as described in Section 6.10.1.

6.2 Study Population

In general, data such as patient disposition, demographics and baseline characteristics will be summarized for FAS and SAF by treatment arm and overall, unless specifically stated otherwise. In the event when FAS is identical to SAF (i.e., no one received the wrong study drug), then these data summaries will not repeated for SAF.

6.2.1 Disposition of Subjects

The following summaries will be presented. A table may include one or more of the summaries.

- Number and percentage of subjects with informed consent, discontinued before randomization (screening failures), randomized (overall only);
- Number and percentage of randomized subjects in each analysis set, by treatment group and overall;
- Number and percentage of subjects completed/discontinued CAPOX regimen, by primary reason for treatment discontinuation;

- Number and percentage of subjects discontinued Zolbetuximab/placebo, by primary reason for treatment discontinuation;
- Number and percentage of subjects completed/not completed the 30-day post- CAPOX safety follow-up visit, by primary reason for not completing the visit;
- Number and percentage of subjects completed/not completed the 30-day post-Zolbetuximab/placebo safety follow-up visit, by primary reason for not completing the visit;
- Number and percentage of subjects completed/not completed the 90-day post- CAPOX follow-up visit, by primary reason for not completing the visit;
- Number and percentage of subjects completed/not completed the 90-day post-Zolbetuximab/placebo follow-up visit, by primary reason for not completing the visit;
- Number and percentage of subjects completed/not completed post-treatment follow-up, by primary reason for completing or not completing post-treatment follow-up;
- Number and percentage of subjects completed or discontinued survival follow-up period, by primary reason for survival follow-up discontinuation;

6.2.2 Protocol Deviations

The number and percentage of subjects with the following major protocol deviation criteria will be summarized for each criterion and overall, by treatment group and overall as well as by investigative site, for FAS and SAF. Subjects deviating from a criterion more than once will be counted once for the corresponding criterion. Any subjects who have more than one protocol deviation will be counted once in the overall summary.

The unique identifiers for major protocol deviation will be as follows:

PD1 - Entered into the study even though they did not satisfy entry criteria,

PD2 - Developed withdrawal criteria during the study and was not withdrawn,

PD3 - Received wrong treatment or incorrect dose,

PD4 - Received excluded concomitant treatment.

6.2.3 Demographic and Other Baseline Characteristics

Demographic variables (sex, age, age groups defined in the subgroup section, race, ethnicity, country), height, weight, BMI, BSA, tobacco history, baseline ECOG status, and the three stratification factors (listed in Section 2.3) will be summarized for FAS and SAF.

Primary diagnosis and tobacco history will be summarized for FAS.

Medical history is coded in MedDRA, and will be summarized by System Organ Class (SOC) and Preferred Term (PT) by treatment arm for FAS and SAF.

6.2.4 Transfusions

The blood product, duration, number of units will be summarized for FAS.

6.2.5 Previous and Concomitant Medications

Previous and concomitant medications will be summarized in separate tables by therapeutic subgroup (ATC 2nd level) and chemical subgroup (ATC 4th level) and preferred WHO name by treatment group for the FAS. Subjects taking the same medication multiple times will be counted once per medication and investigational period. A medication that can be classified into several chemical and/or therapeutic subgroups is presented in all chemical and therapeutic subgroups.

Previous medications are defined as medications that patients started prior to first administration of study medication. Concomitant medications are defined as any medications that patients took after the first dose of study medication (inclusive) and before the date of last dose of study medication (inclusive). Medications that started prior to first administration of study drug and continued while study drug was given will be counted as both previous and concomitant medications.

6.2.6 Non-medication Therapies

Reason for use will be summarized for FAS.

6.2.7 New Anti-Cancer Therapies

Subsequent anti-cancer therapies will be summarized by treatment arm by drug class for FAS.

6.2.8 Prior Radiation Therapy

The following variables will be summarized for FAS: whether subject received prior radiation therapy, area, duration, and reason of radiation therapy.

6.2.9 Prior Procedures for Primary Cancer

Frequency tabulations of subjects with surgery or procedures for the treatment of the primary cancer will be presented by treatment group and overall for FAS.

6.2.10 Prior Cancer Chemotherapy

The following variables will be summarized for FAS: whether subject received any chemotherapy medication, name and duration of chemotherapy, and reason for discontinuing medication.

6.3 Study Drug Exposure and Compliance

Duration and compliance of study drug will be summarized for SAF by treatment group and overall.

The following variables will be derived and summarized:

- Duration of Zolbetuximab or placebo and Oxaliplatin, defined as (date of last infusion – date of first infusion + 1). For those who did not receive any dose, duration will be 0.
- Duration of Capecitabine, defined as (date of last dose – date of first dose + 1). For those who did not receive any dose, duration will be 0.

- Duration of period for which all components are administered and duration of period for Any Component Administered
- Proportion of subjects who completed the 8 cycles of CAPOX treatment.
- Number of infusions administered, number of infusions entirely administered and number of infusion not entirely administered, number of infusions with dose adjustment, Number of infusions with dose adjustment due to AE, number of infusions with delay, Number of infusions with delay due to AE, number of infusions with interruption, Number of infusions with interruption due to AE, number of infusions prematurely discontinued for Zolbetuximab and Oxaliplatin and Number of infusions prematurely discontinued for Zolbetuximab and Oxaliplatin due to AE.
  For the interruptions that involve overnight infusion, only one infusion will be counted
- Number of cycle administered for oral dosing of capecitabine, number of cycle with dose adjustment, Number of cycle with dose adjustment due to AE, number of cycle with delay, number of cycle with delay due to AE, number of cycle with interruption, number of cycle with interruption due to AE, number of cycle prematurely discontinued for Capecitabine, number of cycle prematurely discontinued for Capecitabine due to AE.
- Average infusion time, calculated as (stop time – start time), for Zolbetuximab, placebo and Oxaliplatin. Interruption time is included in the infusion time if the infusion were finished within one day; for interruptions that goes overnight, interruption time will not be included.
- Cumulative actual dose of Zolbetuximab/placebo and each component of CAPOX.
- Average amount of dose per infusion for each component administered via infusion.
- Average amount of dose per planned dosing day for Capecitabine.
- Relative dose intensity (RDI), defined as (Cumulative actual dose/Planned cumulative dose). Where Planned Cummulative Dose is defined as Protocol specified planned cumulative dose.
- RDI category: <50, 50 to 80 inclusive, >80, unknown
- Number and percentages of subjects with the following cumulative categories of study drug (each component) duration will be summarized: ≥ 1d, > 6w, > 12w, > 24w, > 36w, > 48w, > 72w

6.4 Analysis of Efficacy

To address multiplicity, a gatekeeping testing strategy will be used for PFS (primary efficacy endpoint) and OS (key secondary endpoint). PFS will be tested once at 1-sided significance level of 0.025. Only if PFS is significant, hypothesis testing for OS interim and OS final analyses will be performed. An O'Brien-Fleming type alpha-spending function [Lan & DeMets, 1983] will be utilized to control the overall 1-sided 0.025 significance level for the OS interim and final analyses. Other secondary endpoints' testing will not be multiplicity adjusted.

For imaging assessments of tumor, date of assessment for each timepoint is defined as the date of last scan (if there are multiple scans over several days) for that timepoint, not the date when the overall timepoint response is recorded by radiologist in the system, with the

exception of PD date. PD date is defined in Table 1. Date of time point assessment of tumor as determined by IRC/investigators will be used for analysis.

6.4.1 Analysis of Primary Efficacy Endpoint (PFS)

6.4.1.1 Primary Analysis for Primary Efficacy Endpoint

The primary analysis of PFS will use radiological assessment of PD by the IRC and be performed for FAS. The hypothesis to be tested is:

- H_0: PFS of Arm A is not prolonged compared to that of Arm B
- H_a: PFS of Arm A is prolonged compared to that of Arm B

Comparison of Arm A and Arm B will be tested at 1-sided significance level of 0.025.

The distribution and median of PFS will be estimated for each treatment arm using Kaplan-Meier methodology. PFS rates at 6m, 12m, and 18m will be presented by Kaplan-Meier method too. In addition, numbers of subjects with PFS events and censored and 95% CI for median PFS and PFS rates will be presented.

Hypotheses testing between Arm A and Arm B will be performed using log-rank test stratified by:

- Region (Asia vs. Non-Asia)
- Number of Organs with Metastatic Sites (0 to 2 vs. ≥ 3)
- Prior Gastrectomy (Yes vs. No)

In addition, stratified Cox proportional hazards model will be used to estimate the hazard ratio and the corresponding 95% confidence interval. Table 1 defines PFS endpoint for the primary analysis. Evaluable radiological assessments include all assessments except those assessed by IRC as NE (not evaluable).

Table 1 PFS Primary Analysis Definition (based on IRC radiological assessments only)

Situation	Date of Event or Censoring	Outcome
No baseline imaging assessments	Date of randomization	Censored
No evaluable post-baseline imaging assessments, no death	Date of randomization	Censored
**Subject did not receive new anti-cancer therapy (ACT):**		
Radiological PD documented per RECIST v1.1	Date of first radiological PD (defined as earliest of date of scan showing new lesion if PD is based on new lesion or date of last scan of target lesions if PD is based on increase in sum of diameters (SOD) of target lesions)	Event
No radiological PD, but death recorded on eCRF	Date of death	Event
Neither radiological PD nor death	Date of last radiological assessment	Censored

Situation	Date of Event or Censoring	Outcome
**Subject received new anti-cancer therapy (ACT)*:**		
Radiological PD per RECIST v1.1 documented only after start of new ACT	Date of last radiological assessment before start of new ACT	Censored
Radiological PD documented per RECIST v1.1 before start of new ACT	Date of first radiological PD	Event
No radiological PD nor death	Date of last radiological assessment before start of new ACT	Censored
**Missed >=2 scheduled radiological assessments:**		
If radiological PD or death occurs after missing 2 or more scheduled radiological assessments**	Date of last radiological assessment	Censored

Note: PFS = date of event or censoring – date of randomization + 1. NE will be treated as missing in the derivation described in this table *New ACT includes new anti-cancer surgery, radiotherapy, chemo, immunotherapy (other than Zolbetuximab and CAPOX components) and on study tumor directed procedures after randomization. If a subject in Arm B switches from placebo to Zolbetuximab, it is also considered start of new ACT. **If the first radiological assessment after subject missed >=2 imaging assessments is SD or better and it's confirmed that subject did not take any other ACT during the missing period, the following imaging assessments will be used rather than censored.

6.4.1.2 Sensitivity Analysis 1 for Primary Efficacy Endpoint

The primary analysis will be repeated using radiologic PD assessments by local investigators only. In addition, a summary of discordance between IRC's and local investigator's PD assessments will be presented.

6.4.1.3 Sensitivity Analysis 2 for Primary Efficacy Endpoint

This analysis will treat likely informative censoring as PFS events. The primary analysis will be repeated with the following cases treated as PFS events rather than censored (whichever earliest):

- If subject dropped out of imaging follow-up without documented PD by IRC and there was investigator-reported radiological progression at the last imaging assessment, then the next scheduled date of imaging (i.e., date of last imaging assessment + 9w or 12w) will be treated as date of PFS event (even though the next scheduled imaging did not take place).
- If subject dropped out of imaging follow-up without documented PD by IRC and there was investigator-reported clinical progression around or after the time of last imaging assessment, then the next scheduled date of imaging (i.e., date of last imaging assessment + 9w or 12w) will be treated as date of PFS event (even though the next scheduled imaging did not take place).

- If subject dropped out of imaging follow-up without documented PD by IRC and there was ECOG performance status worsening from baseline (from 0-1 to >=2) around or after the time of last imaging assessment, then the next scheduled date of imaging will be treated as date of PFS event.
- Start date of new ACT will be treated as date of PFS event, when there is no prior documented PD by IRC.
- If subject missed >=2 scheduled imaging assessments without prior documented PD by IRC and there was investigator-reported clinical progression around or after the time of last imaging assessment prior to missing, then the next scheduled date of imaging after the last non-missing assessment will be treated as date of PFS event (even though the next scheduled imaging did not take place).
- If subject missed >=2 scheduled imaging assessments without prior documented PD by IRC and there was ECOG performance status worsening from baseline (from 0-1 to >=2) around or after the time of last imaging assessment prior to missing, then the next scheduled date of imaging after the last non-missing assessment will be treated as date of PFS event.

6.4.1.4 Sensitivity Analysis 3 for Primary Efficacy Endpoint

The primary analysis will be repeated where death after new ACT will be censored at date of last radiological assessment before start of new ACT.

6.4.1.5 ther Analysis of Primary Efficacy Endpoint

Imaging assessment interval (weeks) will be summarized for each treatment arm for the two periods: <=54 weeks and >54 weeks. If imbalance of imaging assessment interval is observed between the two arms, additional sensitivity analysis may be performed using protocol-planned dates of imaging assessments instead of actual dates of assessments. In addition, duration of imaging follow-up, defined as (date of the last on-study imaging assessment – date of randomization + 1), will be summarized for each treatment arm.

6.4.2 Analysis of Secondary Efficacy Endpoints

6.4.2.1 Overall Survival

A key secondary endpoint OS is defined as the time from date of randomization to the documented date of death from any cause. All deaths will be included, regardless of whether death occurred while the subject is still taking study drug or after the subject discontinue study drug. OS analysis will be performed for FAS.

Table 2 OS Definition

Situation	Date of Event or Censoring	Outcome
Death before analysis cutoff date	Date of death	Event
Last known alive date is before cutoff date	Last known alive date	Censored
Death after analysis cutoff date	Analysis cutoff date	Censored
Last known alive date is after cutoff date	Analysis cutoff date	Censored

OS = Date of Event or Censor – Date of Randomization +1

To maintain the overall Type I error rate at the 0.025 significance level, the hypothesis testing for OS interim and OS final analyses will be performed only if the null hypothesis in PFS primary analysis is rejected at the overall 1-sided 0.025 significance level.

The formal OS interim analysis is planned when the final PFS analysis occurs with the pre-specified number of PFS events. A group sequential design using the O'Brien-Fleming type alpha-spending function [Lan & DeMets, 1983] will be utilized to control the overall 1-sided 0.025 significance level (East®) for the OS analysis. The independent data monitoring committee (IDMC) may recommend terminating the trial for favorable results at the formal OS interim analysis. In the case of favorable results, the 1-sided significance level for superiority is 0.0074 for the interim OS analysis and 0.0228 for the final OS analysis. These alpha boundaries are based on an information factor of 70% and are subject to adjustment if observed information factor deviates from 70%. If the 1-sided P-value of the interim analysis is less than 0.0074, the IDMC may recommend terminating the trial for success without the need to conduct the final OS analysis. If the study is not stopped after the interim analysis, the final OS analysis will occur after 100% of the planned number of deaths have been observed.

The distribution and median of OS, as well as OS rates at 12m, 18m, 24m, 30m and 36m will be estimated for each treatment arm using Kaplan-Meier methodology. 95% CI for median OS and milestone OS rates will be presented. Arm A and Arm B will be compared using the log-rank test stratified by the same stratification factors used for PFS analysis. The hypothesis to be tested is:

- H_0: OS of Arm A is not prolonged compared to Arm B
- H_a: OS of Arm A is prolonged compared to Arm B

In addition, stratified Cox proportional hazard model will be used to estimate the hazard ratio and the corresponding 95% CI.

The median of time on study using reverse Kaplan-Meier approach and range (min and max) of time on study will be provided. Subjects alive up to date cut-off date will be considered as events and death of subjects on or prior to data cut-off date will be censored in the reverse Kaplan-Meier approach for estimation of median of time on study.

6.4.2.2 Health-Related Quality of Life

6.4.2.2.1 Scoring of HRQoL Questionnaires

For EORTC QLQ-C30, scores for 5 functional scales (physical, role, emotional, social and cognitive), 9 symptom scales/items, and global health status scale will be calculated according to the EORTC scoring manual in Appendix 9.2 (Fayers, et al, 2001). These scores will be standardized to a 0-100 scale. A high score for a functional scale and the global health status represents a healthy level of functioning (high QoL), while a high score for a symptom scale or item represents a severe level of symptoms (low QoL).

In addition, the QLQ-C30 Summary Score (Giesinger et al, 2016) will be calculated using 27 out of 30 items as follows (excluding 3 items on financial impact and global health status):

QLQ-C30 Summary Score = (Physical Functioning+ Role Functioning+ Social Functioning+ Emotional Functioning+ Cognitive Functioning+ 100-Fatigue+ 100-Pain+ 100-Nausea_Vomiting+ 100-Dyspnoea+ 100-Sleeping Disturbances+ 100-Appetite Loss+ 100-Constipation+ 100-Diarrhoea)/13.

The QLQ-C30 Summary Score should only be calculated if all of the required 13 scale/item scores are available (using scale scores based on the completed items, provided that at least 50% of the items in that scale have been completed). A high Summary Score represents high QoL.

EORTC QLQ-OG25 questionnaire consists of 6 scales: dysphagia (items 1-3), eating restrictions (items 4-7), reflux (items 8-9), odynophagia (items 10-11), pain and discomfort (items 12-13) and anxiety (items 14-15), as well as 10 single items: eating in front of others (item 16), dry mouth (item 17), trouble with taste (item 18), body image (item 19), trouble swallowing saliva (item 20), choked when swallowing (item 21), trouble with coughing (item 22), trouble talking (item 23), weight loss (item 24) and hair loss (item 25). Also, we added two items from EORTC QLQ-STO22 related to belching (item 26-27)

EORTC QLQ-OG25 7 scales plus 10 single items will be scored in the same way as QLQ-C30 symptoms scales/items. The scores will also be transformed to a 0-100 scale. Higher score means severer level of symptoms.

In addition, the EORTC QLQ-C30 and OG25 individual symptom scores and the Belching questionnaire will be categorized as the following:

a. For questions which have a 4 point scales with 1 being the best, and 4 being the worst, categorized as 1=”None”, 2=”Slight”, 3=”Moderate”, 4= “Severe”.

The EQ-5D-5L scores (on 1-5 scale) will be categorized as: 1=”None”, 2=”Slight”, 3=” Moderate”, 4=”Severe”, 5=”Extreme”.

6.4.2.2.2 Analysis of HRQoL Questionnaires

Analyses will be by treatment arm and based on FAS. All the PRO assessments while on study drug, as well as end of treatment will be considered.

6.4.2.2.2.1 Patient disposition

The subject disposition by treatment group for all PRO assessment time-points (e.g. analysis visits) will be provided:

- The number of subjects with PRO assessment expected
- The number and % of subjects with PRO assessment not expected due to progression
- The number and % of subjects with PRO assessment not expected due to death
- The number and % of subjects with PRO assessment not expected due to other reasons

The subject disposition by treatment group per analysis visit will also be provided graphically by means of a stacked bar chart.

6.4.2.2.2.2 Completion rate

Instrument completion rate at each analysis visit will be reported for each instrument:

- Completion rate (i.e. unadjusted) at each analysis visit will be calculated as the number of subjects meeting the minimum requirements for scoring at least one domain of the instrument divided by the number of subjects in the FAS population.
- Compliance rate (i.e. adjusted) at each analysis visit will be calculated among subjects who are expected to have PRO assessments. The following will be provided:
  - The number and % of subjects with all questions completed
  - The number and % of subjects meeting at least the minimum requirements for scoring of the instrument; these requirements are as follows:
  - EQ-5D-5L: 1) the utility index 2) the EQ-VAS is calculated
  - EORTC QLQ-C30: at least one subscale can be calculated
  - EORTC QLQ-OG25 plus STO22 Belching: at least one subscale can be calculated
  - The number and % of subjects with at least one question completed

The completion (unadjusted) and compliance (adjusted) rates by treatment group at each analysis visit will also be provided graphically by means of a line graph.

6.4.2.2.2.3 Time to deterioration for GHS/QoL, PF and OG25-Pain

Definition

Time to clinically meaningful symptom worsening or HRQoL deterioration (PRO deterioration) will be analysed for each scale of the PRO instruments collected in this study separately. For convenience, a generic term “time to clinically meaningful deterioration” will be used both for symptom worsening and HRQoL deterioration, with an understanding of a specific meaning depending on the scale or subscale analysed. The following definition will be considered for the secondary endpoints of time to deterioration in GHS/QoL, PF and OG25-Pain.

Time to confirmed deterioration (TTCD) will be defined as the duration of time from the date of randomization to the date of the first deterioration in PRO scores of at least one threshold unit as compared to the baseline score if the deterioration of at least one threshold unit as compared to the baseline score is also observed at the next consecutive scheduled visit (e.g., after the first deterioration is observed) or if the patient dropped out after deterioration or died, resulting in missing data.

For those patients who experienced first confirmed clinically meaningful deterioration, TTCD will be computed as follows and then converted to months:

TTCD = Date of assessment when first confirmed clinically meaningful deterioration was observed – Date of randomization + 1

Patients with a non-missing baseline assessment will be censored at the last available PRO assessment. Patients with no baseline PRO assessment, or without post-baseline PRO questionnaire, or whose baseline scores do not allow for further deterioration will be

censored at the randomization. Death or progression will not be considered deterioration events.

Given the absence of reliable and well-accepted thresholds for within-patient clinically meaningful change, the clinically meaningful threshold denoting a deterioration will be defined based on anchor-based analyses performed on the same trial data. These analyses are described in a separate pre-specified analysis plan for an exit survey dated 09-Mar-2022. Sensitivity analyses with the next higher threshold value that will provide a different classification for deterioration for each scale will also be performed. The reason of this approach is that EORTC values are discrete in nature due to the transformation of raw scores to 0-100, therefore not all values are possible within this range and certain threshold values will result in the same categorization for patients, e.g., for the two-item OG25-Pain domain, the possible values are 0, 16.7, 33.3, 50, 66.7, 83.3 and 100, therefore applying a threshold of 10 and 14 will result in the same classification of patients into deterioration and no deterioration. If the resulting threshold value from the anchor-based analysis is for example 10, then the sensitivity threshold will be 17.

The above analyses will be also repeated by employing the sensitivity/secondary thresholds.

Alternative definitions for the time to deterioration, such as time to first deterioration, or time to definitive deterioration, will also be described in a separate SAP for PRO-related analyses. Briefly, time to first deterioration (TTFD) will be defined as the duration of time from the date of randomization to the date of the first deterioration in PRO scores of at least one threshold point as compared to the baseline score. Time to first definitive deterioration (TTDD) will be defined as the duration of time from the date of randomization to the date of the first deterioration in PRO scores of at least one threshold unit as compared to the baseline score, which is:

- also observed at all time points thereafter (e.g., after the first deterioration is observed) or
- followed by drop-out, resulting in monotone missing data.

In addition, the TTCD, TTFD and TTDD definition will be repeated where death (due to any cause) will be counted as an event if the patient does not experience PRO deterioration prior to death and where death occurs within 2 scheduled assessments (e.g., 42 days; this corresponds to the maximal time interval between 2 consecutive scheduled visits for PRO assessment) after the last available PRO assessment; progression will not be considered deterioration event.

Details of the sensitivity/secondary analysis will be included in the PRO related SAP.

Analysis

If the null hypothesis on the OS is rejected at the overall 1-sided 0.025 significance level, then the TTCD will be tested using the gatekeeping procedure with the following order:

1. Non-inferiority testing for TTCD in PF at 0.025 significance level
2. Non-inferiority testing for TTCD in OG25-Pain at 0.025 significance level

3. Non-inferiority testing for TTCD in GHS/QoL at 0.025 significance level
4. Superiority testing for TTCD in PF at 0.025 significance level
5. Superiority testing for TTCD in OG25-PA at 0.025 significance level
6. Superiority testing for TTCD in GHS/QoL at 0.025 significance level

A stratified Cox proportional hazard model will be used to estimate the hazard ratio and the corresponding 95% CI for the three time-to-event PRO endpoints.

To determine whether Zolbetuximab (IMAB362) in combination with CAPOX chemotherapy is inferior to Placebo in combination with CAPOX chemotherapy, a non-inferiority margin of 1.33 in terms of hazard ratio with respect to experiencing a definitive deterioration as defined in the previous section will be used.

The primary hypothesis (H1) is defined as:

H0: HR ≥ 1.33 vs. H1: HR < 1.33

The hypothesis of non-inferiority will be tested at a one-sided significance level of 0.025 in the FAS population using a stratified Cox Proportional Hazards model with adjusting for randomization stratification factors: Region (Asia vs Non-Asia), Number of organs with metastatic sites (0 to 2 vs ≥ 3), Prior gastrectomy (Yes or No). The 95% confidence interval for the hazard ratio of Zolbetuximab (IMAB362) Plus CAPOX to Placebo Plus CAPOX will be estimated. If the upper limit of the 95% confidence interval for the estimated HR for the stratified Cox model is below the non-inferiority margin of 1.33, then non-inferiority of the TTCD endpoints in patients treated with Zolbetuximab (IMAB362) Plus CAPOX compared to that of patients treated with Placebo Plus CAPOX will be declared.

If the non-inferiority hypothesis are met for all 3 HEOR endpoints, the hypothesis of superiority for these endpoints will be tested.

The hypothesis (H1) is defined as: H0: HR ≥ 1 vs. H1: HR < 1

Kaplan-Meier curves will be used to estimate the distribution of TTCD. The 50th percentile of Kaplan-Meier estimates will be used to estimate the median duration of TTCD, respectively. A two-sided 95% confidence interval will be provided for these estimates. Median TTCD will be compared using stratified log rank test adjusting for randomization stratification factors: Region (Asia vs Non-Asia), Number of organs with metastatic sites (0 to 2 vs ≥ 3), Prior gastrectomy (Yes or No). A Kaplan-Meier plot by treatment group will be presented.

Additionally, the benefit of Zolbetuximab (IMAB362) Plus CAPOX compared to Placebo Plus CAPOX will be evaluated by a single hazard ratio (HR) (Zolbetuximab vs Placebo) with its 95% confidence interval based on a stratified Cox regression model with the same strata as above. The proportional hazards assumption will be tested by examining plots of complementary log(-log(survival)) versus log(time). In case departures from the assumption are observed, only the KM and the quartiles of the survival distribution will be presented.

Kaplan-Meier analysis will be performed using PROC LIFETEST (SAS procedure). Cox proportional hazard regression model will be performed using PROC PHREG (SAS procedure).

6.4.2.2.2.4 Descriptive analysis

For QLQ-C30's 5 functional scales, global health status, Summary Score, and EQ-5D-5L VAS

Scores (on 0-100 range) as well as change from baseline will be summarized by visit for each visit where at least 10 subjects are evaluable for any treatment arm. Summary statistics will not be provided for the treatment arm for the visit if that arm has less than 10 subjects at that visit. Plot of mean change from baseline over time will be presented by treatment arm for each score. The highest and lowest change from baseline of each subject (highest value post baseline –baseline value and lowest value post baseline – baseline value) will be summarized by treatment arm.

For QLQ-C30 and QLQ-OG25 plus STO-22 belching subscale symptoms:

Scores (on 0-100 range) will be summarized separately in the same way as above. Frequency distribution of categorized QLQ-C30 and QLQOG25 symptom scores will be presented by treatment arm and visit. Shift from baseline category to subject's worst post-baseline category will be tabulated (except for 7 points scale questionnaire).

EQ-5D-5L 5 questions

Scores (on 1-5 range) will be analyzed using descriptive statistics including change from baseline. Categorized scores will be tabulated using shift table (shift from baseline to worst category post baseline).

Global pain

Score and change from baseline will be summarized by visit.

6.4.2.3 Objective Response Rate

The ORR is defined as the proportion of subjects with a BOR of complete response (CR) or partial response (PR) based on IRC per RECIST V1.1.

Best overall response (BOR) will be determined once all tumor response data for the subject is available. Subjects' BOR will be determined as outlined in RECIST V1.1 criteria based on IRC assessments. BOR is defined as the best response among all timepoints' overall responses excluding NE responses (CR is better than PR and PR is better than SD and SD is better than PD). If all timepoint overall responses are NE, BOR is NE. For BOR of SD, SD must be documented as present at least once and at least 8 weeks after randomization. If the first assessment of SD does not meet the minimum "8 weeks from randomization" time window, that assessment of SD will be treated as NE in analysis. Timepoint responses after start of new ACT will not be used in determining BOR.

The comparison of ORR between Arm A and Arm B will be performed using stratified Cochran-Mantel-Haenszel (CMH) test with the same stratification factor used for the PFS

analysis. In addition, ORR for each arm will be estimated and corresponding 95% CI will be constructed using Clopper-Pearson method.

In addition, percent of subjects with BOR of CR, PR or SD as well as ORR and DCR will be summarized.

Sensitivity analyses include:

- Analysis of ORR with confirmation, defined as the proportion of subjects with best overall response as confirmed CR or confirmed PR based on the RECIST v1.1 as assessed by IRC. Confirmation of CR or PR should occur at the next scheduled assessment (>= 4 weeks following the initial assessment at which CR or PR is observed). See Table 3 for rules of confirmation of CR and PR.

Table 3 Confirmation of overall response:

Overall response at current timepoint	Overall response at next timepoint	Confirmed overall response at current timepoint
CR	CR	CR
PR	CR/PR	PR
CR/PR	PD	SD provided minimum criteria for SD duration met, otherwise, PD
PR	SD	SD
SD	CR/PR	SD
SD	SD/PD/NE	SD provided minimum criteria for SD duration met, otherwise, PD or NE.
CR	NE, followed by CR at the following assessment	CR
PR	NE, followed by CR/PR at the following assessment	PR
CR	NE	SD provided minimum criteria for SD duration met, otherwise, NE
PR	NE	SD provided minimum criteria for SD duration met, otherwise, NE
NE	NE	NE

- Analysis of ORR per investigator assessment for both confirmed and unconfirmed response.

6.4.2.4 Duration of Response

DOR is defined as the time from the date of the first CR or PR (whichever is first recorded) as assessed by IRC to the date of radiological PD as documented by IRC or death, whichever is earlier. The DOR analysis will be performed on the subset of FAS who have at least one CR or PR documented by IRC. If a subject has not progressed, the subject will be censored at the date of last evaluable radiological assessment or at the date of first CR/PR if no later

evaluable radiological assessment is available. Other censoring used for the PFS analysis (see Table 1) will apply to DOR too.

DOR = Date of Event or Censor – Date of the first CR/PR + 1.

The distribution of DOR will be estimated for each treatment arm using Kaplan-Meier methodology and compared between Arm A and Arm B using the log-rank test stratified by the same stratification factors used for the PFS analysis. In addition, stratified Cox proportional hazard model will be used to estimate the hazard ratio and the corresponding 95% CI.

As sensitivity analyses, DOR analysis will be repeated for confirmed responses. For confirmed CR/PR, date of first confirmed CR/PR is defined as the date of first assessment of CR/PR (not the later assessment confirming the previous CR/PR).

DOR analysis will also be repeated using investigator assessment, and it will include both analysis for confirmed and unconfirmed response.

6.4.3 Analysis of Exploratory Efficacy Endpoints

6.4.3.1 Time to Progression

TTP is defined as the time from the date of randomization until the date of PD per RECIST 1.1 by IRC. TTP does not include deaths as event. For deaths before the first documented PD by IRC, subjects will be censored at the time of last evaluable radiological assessment. The TTP analysis assumes that deaths without documented PD are not related to tumor progression and estimates TTP for these dead subjects (who did not have documented PD prior to death) as if the subjects had not die. See Table 4 for derivation of the TTP endpoint. Kaplan-Meier and log-rank methods will be applied to the TTP endpoint.

Table 4 TTP Definition (based on IRC radiological assessments only)

Situation	Date of Event or Censoring	Outcome
No baseline imaging assessments	Date of randomization	Censored
No evaluable post-baseline imaging assessments	Date of randomization	Censored
**Subject did not receive new anti-cancer therapy (ACT):**		
Radiological progression documented per RECIST v1.1	Date of radiological PD (defined as earliest of date of scan showing new lesion if PD is based on new lesion or date of last scan of target lesions if PD is based on increase in sum of diameters (SOD) of target lesions)	Event
No radiological progression, but death recorded on eCRF	Date of last radiological assessment	Censored
Neither radiological progression nor death	Date of last radiological assessment	Censored

Situation	Date of Event or Censoring	Outcome
**Subject received new anti-cancer therapy (ACT) *:**		
Radiological progression documented per RECIST v1.1 after new ACT	Date of last radiological assessment before start of new anti-cancer therapy	Censored
Radiological progression documented per RECIST v1.1 before new ACT	Date of radiological PD	Event
No radiological progression before new ACT but death recorded	Date of last radiological assessment before start of new anti-cancer therapy	Censored
No radiological progression nor death	Date of last radiological assessment before start of new anti-cancer therapy	Censored
**Missed >=2 scheduled radiological assessments:**		
If radiological progression occurs after missing 2 or more scheduled radiological assessments**	Date of last radiological assessment	Censored

NE will be treated as missing in the derivation described in this table *New ACT includes new anti-cancer surgery, radiotherapy, chemo, immunotherapy (other than Zolbetuximab and CAPOX components) and on study tumor directed procedures after randomization. If a subject in Arm B switches from placebo to Zolbetuximab, it is also considered start of new ACT. **If the first radiological assessment after subject missed >=2 imaging assessments is SD or better and it’s confirmed that subject did not take any other ACT during the missing period, the following imaging assessments will be used rather than censored.

As sensitivity analyses, TTP analysis will be repeated using investigator assessment.

6.4.3.2 Progression Free Survival After Subsequent Therapy (PFS2)

PFS2 is defined as the time from the date of randomization until the date of radiological/objective PD (per subject’s local physician) following subsequent (2nd line) anti-cancer therapy or death from any cause, whichever is earliest. In cases where PFS2 cannot be reliably determined, end date of subsequent (2nd line) ACT or start date of 3rd line ACT may be used as the event date. Otherwise, subjects will be censored. Subjects who are alive and for whom a PFS2 event has not been observed should be censored at the last time of time known to be alive. Here last know alive date is used as a surrogate to the last radiological assessment date as the later may not be available in the database after 2nd line ACT.

The distribution of PFS2 will be estimated for each treatment arm using Kaplan-Meier methodology and compared between Arm A and Arm B using stratified log-rank test with the same stratification factors used for the PFS analysis. In addition, stratified Cox proportional hazard model will be used to estimate the hazard ratio and the corresponding 95% CI.

Table 5 PFS2 Definition (as compared to PFS)

Situation	PFS2 event/censor	PFS2 date
Subject did not take new ACT:		
Subject died	Event	Death date
Subject did not die	Censor	Last known alive date
Subject started new ACT:		
PD (based on investigator) after new ACT or Death	Event	Date of PD after new ACT or Date of death, whichever earlier
No PD (based on investigator) after new ACT, No Death. Ended 2nd or started 3rd line ACT.	Event	End date of 2nd line ACT, or start date of 3rd line ACT, whichever earlier.
No PD (based on investigator) after new ACT, No Death. Not record of End date of 2nd line ACT or start date of 3rd line ACT.	Censor	Last known alive date

6.4.3.3 Disease Control Rate

The DCR is defined as the proportion of subjects with a BOR of SD, CR or PR based on RECIST 1.1 by IRC.

The comparison of DCR between Arm A and Arm B will be performed using CMH test with the same stratification factor used for PFS analysis. In addition, DCR for each arm will be estimated and corresponding 95% CI will be constructed.

As sensitivity analyses, DCR analysis will be repeated using investigator assessment.

6.4.3.4 Health Resource Utilization

HRU variables by visit and their sums over all visits, will be summarized by treatment arm:

- Number of ER visits
- Number of ER visits with hospitalization
- Duration of ER hospitalizations
- Number of hospital admissions
- Duration of stay in hospital
- Number of general practitioner visits
- Number of specialist visits

The above summaries will be repeated for the time period before subjects' radiological PD.

6.5 Analysis of Safety

Safety analyses will be based on SAF and by treatment arm and overall. All SAF subjects will be analyzed according to the actual treatment they received. Astellas Standard TLF templates should be followed wherever applicable.

Safety analyses will only include data collected during and after the first dose of the first study drug component given, up to 30 days after the last dose of the last study drug component.

6.5.1 Adverse Events

AEs will be coded using the Medical Dictionary for Regulatory Activities (MedDRA) and graded using NCI-CTCAE. MedDRA Version used will be presented in the title of the related TLFs.

TEAE is defined as an AE observed after starting administration of the study treatment and within 30 days after the last dose of the last administered component of study treatment. Late AE/SAE is defined as AE/SAE that is collected after 30 days post last dose of study drug. Serious TEAE summaries include both investigator-assessed and Astellas upgraded SAEs.

Separate summaries for AE/SAE related to any component of the study drug and AE/SAE related to each component of study drug will be provided for Drug-related AE/SAE summaries.

An overview summary table will include the following details:

- Number and percentage of subjects with TEAEs,
- Number and percentage of subjects with drug-related TEAEs
- Number and percentage of subjects with serious TEAEs,
- Number and percentage of subjects with serious drug-related TEAEs,
- Number and percentage of subjects with TEAEs leading to permanent discontinuation of any component of study drug and by component
- Number and percentage of subjects with drug-related TEAEs leading to permanent discontinuation of any component of study drug and by component
- Late adverse event occurred beyond 30 days from the last study treatment (all components)
- Late serious adverse event occurred beyond 30 days from the last study treatment (all components)
- Number and percentage of subjects with NCI CTCAE grade 3 or higher TEAEs
- Number and percentage of subjects with drug-related NCI CTCAE grade 3 or higher TEAEs
- Number of deaths from first dose of study drug up to 30 days after the last dose of the last administered component of study drug
- Number and percentage of subjects with TEAE leading to death
- Number and percentage of subjects with drug-related TEAE leading to death
- Number of all deaths up to analysis cutoff date

The number and percentage of subjects with TEAEs, as classified by SOC and PT will be summarized. Summaries will be provided for:

- TEAEs
- Drug-related TEAEs,
- AEs collected after 30 days post last dose of study drug,
- Serious TEAEs,
- Serious AEs collected after 30 days post last dose of study drug,
- Drug-related serious TEAEs,

- TEAEs leading to permanent discontinuation of any component of study drug and by component,
- Drug-related TEAEs leading to permanent discontinuation of any component of study drug and by component,
- TEAE Leading to Dose Interruption of any component of study drug and by component
- Drug-Related TEAE Leading to Dose Interruption of any component of study drug and by component
- TEAE Leading to Dose Reduction of any component of study drug and by component
- Drug-Related TEAE Leading to Dose Reduction of any component of study drug and by component
- TEAE Leading to Dose Rate reduction of any component of study drug and by component
- Drug-Related TEAE Leading to Dose Rate reduction of any component of study drug and by component
- TEAEs excluding serious adverse events that have a frequency of >=10% in any treatment arm, and
- TEAE with NCI CTCAE Grade 3 or higher
- Drug-related TEAE with NCI CTCAE Grade 3 or higher
- Drug-related TEAEs with treatment group difference in incidence (Arm A - Arm B) >10%
- Drug-related serious TEAEs with treatment group difference in incidence (Arm A - Arm B) >5%

The number and percentage of subjects with TEAEs and TEAEs leading to death, as classified by PT only, will be summarized by treatment group and overall.

AE summary tables will include subject counts as opposed to AE counts, except for serious TEAE and TEAE leading to death where AE counts will also be presented. If a subject experiences more than one episode of a particular AE, that subject will be counted only once for that event. If a subject has more than one AE that code to the same preferred term, the subject will be counted only once for that preferred term. Similarly, if a subject has more than one AE within a body system, the subject will be counted only once in that body system.

The number and percentage of subjects with TEAEs, as classified by SOC and PT, will also be summarized by NCI-CTCAE severity grade and by relationship to study drug. Drug-related TEAEs will be presented in a similar way by severity grade only. If an adverse event changes in severity grade or relationship, then the subject will be counted only once with the worst severity grade and highest degree of relationship. If a subject has an event more than once with missing severity grade and non-missing severity grade, then the subject will be counted as the highest non-missing grade. If a subject has an event more than once with missing relationship and non-missing relationship, then the subject will be counted as "related".

The number and percentage of subjects with TEAEs of interest [listed below], as classified by SOC and PT, will also be summarized. The list of adverse events of interest to be

summarized may change during the course of the study due to ongoing pharmacovigilance. It will be finalized before the database hard lock.

6.5.2 AE of Special interest

Adverse events of special interest (AESI) include the following:

- Nausea
- Vomiting
- Abdominal pain
- Hypersensitivity reactions
- Infusion-related reactions (IRRs)
- Anemia
- Neutropenia

The list of adverse events of special interest to be summarized may change during the course of the study due to ongoing pharmacovigilance. AESIs except for infusion related reactions (IRRs), are classified by SSQ/CMQ or SMQ and PT. IRRs are classified as follows:

- Infusion-related reactions (IRRs)
    - Infusion-related reactions (IRRs) flagged by investigators
    - Potential IRR defined as all AE that have a start date the same as a study treatment day

The number and percentage of subjects with AESI (AE of Special Interest), as classified by SOC and PT will be summarized. Summaries will be provided for:

- AESI
- Serious AESI
- AESI by NCI-CTCAE Grade
- AESI leading to permanent discontinuation
- AESI leading to dose interruption
- AESI leading to dose reduction
- AESI leading to dose rate reduction
- AESI leading to death
-

6.5.3 Clinical Laboratory Evaluation

Quantitative values evaluated by the central laboratory including hematology, biochemistry, urinalysis and coagulation will be summarized using mean, standard deviation, minimum, maximum and median by treatment group at each visit. Additionally, a within-subject change will be calculated as the post-baseline measurement minus the baseline measurement and summarized in the same way. Frequency tabulations of selected qualitative clinical laboratory variables (i.e. urinalysis) will be presented by treatment arm at each visit.

Central laboratory results will also be graded using NCI-CTCAE, where possible. Laboratory parameters that have criteria available for both low and high values, i.e., hypo- and hyper-, will be summarized for both criteria. The same subject can be counted for both criteria if the

subject has different laboratory values meeting each criterion. NCI-CTCAE grade of laboratory results will be summarized by number and percentage of subjects for each visit. Shift table of NCI-CTCAE grade change from baseline to each post-baseline visit will be presented by treatment arm and visit. Shift table of NCI-CTCAE grade change from baseline to worst post-baseline grade will also be presented by treatment arm. Number and percent of subjects with treatment-emergent NCI-CTCAE grade of 3 or 4 laboratory results will be presented by laboratory parameter. "Treatment-emergent NCI-CTCAE grade of 3 or 4 laboratory results" are defined as: subject having a maximum NCI-CTCAE grade of 3 or 4 post baseline for a parameter and that grade is higher than the subject's baseline grade for that parameter (or his/her baseline grade is missing).

The list of laboratory parameters to be summarized may change during the course of the study due to ongoing pharmacovigilance. It will be finalized before the database hard lock.

6.5.3.1 Liver Safety Assessment

The liver safety assessments will be summarized by the following categories below based on the measurements from Alkaline Phosphatase (ALP), Alanine Transaminase (ALT), total bilirubin, Aspartate Transaminase (AST) and their combination as defined.

The subject's highest value post-baseline of each parameter will be used.

- ALT: > 3xULN, > 5xULN, > 10xULN, > 20xULN
- AST: > 3xULN, > 5xULN, > 10xULN, > 20xULN
- ALT or AST: > 3xULN, > 5xULN, > 10xULN, > 20xULN
- ALP: > 1.5xULN
- Total Bilirubin: > 2xULN
- (ALT or AST > 3xULN) and Total Bilirubin > 2xULN
- (ALT or AST > 3xULN) and Total Bilirubin > 2xULN and ALP < 2xULN

The last 2 criteria where 2 or more parameters are evaluated will use the measurements on the same day or up to 1 day apart. The number and percentage of subjects meeting the criteria post-baseline will be summarized by treatment arm.

6.5.4 Vital Signs

Vital signs will be summarized using mean, standard deviation, minimum, maximum and median by treatment arm and visit. Additionally, a within-subject change will be calculated per visit as the post-baseline measurement minus the baseline measurement and summarized by treatment group and visit. For serial vital signs measured values and within-subject change from pre-dose value on that visit will be summarized by visit and timepoint in separate tables.

Tables for potentially clinically significant vital signs will be generated using baseline value and highest value obtained during treatment for each subject.

The following potentially clinically significant criteria are defined for each parameter:

Vital Sign Variable	Criteria
SBP	≥180 mmHg AND ≥20 mmHg change from baseline
SBP	≤ 80 mmHg
DBP	≥105 mmHg AND ≥15 mmHg change from baseline
Pulse Rate	≥120 bpm AND ≥15 bpm change from baseline

6.5.5 Electrocardiograms

ECG variables will be summarized using mean, standard deviation, minimum, maximum and median for each treatment group at each visit, including changes from baseline.

Number and percentage of subjects with normal and abnormal results for the overall interpretation will be tabulated by treatment group and time point. A shift analysis table showing shift in overall ECG interpretation from baseline to each time point will be provided. Percent of subjects on different kind of abnormality will also be reported.

The QT interval corrected by Fridericia's Correction formula (QTcF interval) will be summarized using frequency tables for values of clinical importance using the range criteria below.

QTcF Interval Criteria	QTcF Interval Value (msec)
Normal	≤ 450
Borderline	> 450 to <=480
Prolonged	> 480 to <=500
Clinically significant	> 500

QTcF interval: Fridericia-corrected QT interval

Cumulative tabulation for >450, >480 and >500 msec will also be presented.

The QTcF interval will also be summarized by the frequencies of subjects with a change from baseline of clinical importance using the criteria identified below.

Variable	Change from Baseline
QTcF Interval (msec)	<=0
	>0 to <=30
	>30 to <=60
	> 60

QTcF interval: Fridericia-corrected QT interval

Baseline value is from Cycle 1 Day 1 pre-dose assessment. Cumulative tabulation for >0, >30 and >60 msec will also be presented.

6.5.6 ECOG Performance Status

Number and percent of subjects of for each ECOG performance status grade will be presented at each visit. The change from baseline to each post baseline visit will be summarized. Negative change scores indicate an improvement. Positive change scores

indicate a decline in performance. Shift tables of ECOG performance status change from baseline to worst post-baseline grade will also be presented.

6.5.7 Physical Examination

Weight and change from baseline will be summarized by treatment arm and visit.

6.5.8 Pregnancy

A listing of all pregnancy tests will be provided.

6.6 Analysis of Pharmacokinetics

Descriptive statistics (e.g., N, mean, standard deviation, minimum, median, maximum, coefficient of variation [CV], geometric mean, and geometric CV) will be provided for serum concentrations of Zolbetuximab by scheduled sampling visit and time.

Trough concentrations versus visit profile will be described using box and whisker plots.

Additional model-based analyses may be performed and reported separately.

6.7 Subgroup Analyses

PFS, ORR and DOR based on IRC assessments and OS will be summarized for the following subgroups:

- Age group 1: <=65, >65 years
- Age group 2: <=75, >75 years
- Sex: male, female
- Race: White, Asian
- Tobacco history: Never, current, former
- Region: Asia, Non-Asia
- Number of organs with metastatic sites: 0-2, ≥ 3
- Prior gastrectomy (total or partial): No, Yes
- Histology (tumor type): diffuse vs. intestinal vs. mixed/other
- Tumor location: Gastric vs GEJ; Gastric proximal vs. Gastric distal; GEJ proximal vs GEJ distal;
- Country: Japan vs non-Japan; China vs non China

Forest plots for PFS and OS will be produced to summarize the treatment effect (HR) across subgroups.

6.8 Other Analyses

6.8.1 Immunogenicity

Immunogenicity of Zolbetuximab will be summarized using the frequency of ADA positive subjects. The potential relationship between Zolbetuximab immunogenicity and Zolbetuximab pharmacokinetics, efficacy, safety profile in subjects may be assessed.

Listing of efficacy variable will also be generated for the following subgroup:

- Subjects with negative ADA at baseline and positive at after receiving Zolbetuximab, or subjects with positive ADA and increased ADA titer after receiving Zolbetuximab

6.8.2 Exploratory Biomarkers

Biomarkers may be summarized graphically or descriptively, and summary statistics may be tabulated.

CLDN18.2 status will be summarized by different demographic characteristics as well as by some primary diagnosis variables. Associations between biomarkers and clinical (e.g., efficacy, safety, pharmacodynamics, or pharmacokinetics) measures may be performed on subjects who have sufficient baseline and on-study measurements to provide interpretable results for specific parameters. Analysis will be further described in a biomarker SAP.

6.9 Interim Analysis (and Early Discontinuation of the Clinical Study)

To evaluate whether Zolbetuximab + CAPOX (Arm A) is beneficial compared to the concurrent placebo + CAPOX (Arm B) while the study is ongoing, a formal OS interim analysis is planned when the final PFS analysis occurs with the pre-specified number of PFS events. A group sequential design using the O'Brien-Fleming type alpha-spending function [Lan & DeMets, 1983] will be utilized to control the overall 1-sided 0.025 significance level (East®) for the OS endpoint. The OS interim and final analyses will be performed only if primary PFS analysis is significant.

The IDMC may recommend terminating the study for favorable results at the formal efficacy interim analysis using OS. In the case of favorable results, the 1-sided significance level for superiority is 0.0112, assuming about 78% of the target number of OS events is obtained, for the interim OS analysis and 0.0217 for the final OS analysis (Note: The OS significance level will be adjusted depending on the number of OS event at the time of interim analysis). If the 1-sided P value of the interim analysis is less than the significance level (and PFS is also significant at 1-sided 0.025 alpha),) the IDMC may recommend terminating the study for success. If the study is not stopped after the interim analysis, a final OS analysis will occur after 100% of the planned death events have been observed.

The interim analysis will be run by an Independent Data Analysis Center (IDAC) for IDMC. In addition, safety data reviews during the trial will be conducted by the IDMC on a periodic basis. For example, the IDMC will have its first safety data review 6 weeks after the 40th subject has been randomized and on study drug for 2 cycle (6 weeks) and meetings will be conducted regularly thereafter, as specified in the IDMC Charter.

The full procedures for IDMC safety review will be described in a separate IDMC Charter. An interim analysis plan (IAP) will describe specific analyses to be presented for safety and the efficacy interim reviews.

6.10 Additional Conventions

6.10.1 Analysis Visits

Nominal visits as recorded on eCRF will be used in the by-visit summaries. Values from unscheduled visits will be included in the summary of extreme cases (e.g., summary of worst value post-baseline, summary of minimum value post-baseline, summary of maximum value post-baseline). For efficacy endpoints, all values (scheduled and unscheduled) will be included in the analysis.

For time course plots, actual study day (calculated using actual visit date – date of first dose +1) will be used.

6.10.2 Imputation Rules for Incomplete Dates

Every effort will be made to resolve missing or incomplete dates for adverse events and concomitant medications. If a partial date cannot be resolved, the most conservative imputation methods will be used to complete the missing information. As a general rule, if the month or year is missing, imputation should be avoided if possible. More details on date imputation, if needed, would be placed in the TLF specifications.

7 REVISION AND RATIONALE

7.1 List of Changes in SAP Version 2.0 from Version 1.0 (if applicable)

The changes from the approved SAP Version 1.0 (Dated dd-MMM-yyyy) to Version 2.0 that impact analyses are listed with the rationale in the table below.

SAP Section(s)	Description of Change(s)	Rationale
Section 4.3 in V1.0	Removal of section 4.3 Per Protocol set	The PPS is defined as the subjects who do not meet predetermined study entry and treatment criteria as well as those with lack of imaging assessment.<br>The data from subjects meeting these criteria are unlikely to allow adequate assessment of potential impact on treatment benefit, possibly resulting in risk of bias. Therefore, the robustness of treatment benefit in Primary Endpoint will instead be assessed through sensitivity analyses applying different censoring rules.
Section 3	The number of PFS events required for the interim analysis of overall survival is reduced from 344 to 300.	The number of required PFS events has been adjusted based on the enrollment and event accrual rates to maintain the timing of Primary Analysis with adequate power which is > 93%.
Section 2.1.2, 2.2, 5.2, 6.4.2.2	Addition of health economics and outcomes research (HEOR) related key secondary endpoints, including physical function, Pain, and Global Health Score.	A key secondary endpoint for QOL measures has been added after FDA interaction in order to more specifically address the effect of Zolbetuximab in gastric/GEJ cancer, which impacts the risk/benefit assessment.
Section 6.4.1	Censoring rule has been updated from 'NACT before radiologic progression or death' to 'NACT before radiologic progression'	To be consistent with protocol v5.0, Section 7.4.1.1

8 REFERENCES

ICH Harmonized Tripartite Guideline E 3. Structure and Content of Clinical Study Reports, November 1995. (www.ich.org; Guidelines; "Efficacy" Topics)

ICH Harmonized Tripartite Guideline E 9. Statistical Principles for Clinical Trials, February 1998. (www.ich.org; Guidelines; "Efficacy" Topics)

Fayers PM, Aaronson NK, Bjordal K, Groenvold M, Curran D, Bottomley A, on behalf of the EORTC Quality of Life Group. The EORTC QLQ-C30 Scoring Manual (3rd Edition). Published by: European Organisation for Research and Treatment of Cancer. Brussels 2001.

Giesinger JM et al. Replication and validation of higher order models demonstrated that a summary score for the EORTC QLQ-C30 is robust. J. Clin. Epidemiol. 69:79-88, 2016.

Lan KKG, DeMets DL. Discrete sequential boundaries for clinical trials. Biometrika.1983;70:659-63.

9 APPENDICES

9.1 EORTC QLQ-C30 questionnaire (version 3)

	Not at All	A Little	Quite a Bit	Very Much
1. Do you have any trouble doing strenuous activities, like carrying a heavy shopping bag or a suitcase?	1	2	3	4
2. Do you have any trouble taking a long walk?	1	2	3	4
3. Do you have any trouble taking a short walk outside of the house?	1	2	3	4
4. Do you need to stay in bed or a chair during the day?	1	2	3	4
5. Do you need help with eating, dressing, washing yourself or using the toilet?	1	2	3	4

**During the past week:**	Not at All	A Little	Quite a Bit	Very Much
6. Were you limited in doing either your work or other daily activities?	1	2	3	4
7. Were you limited in pursuing your hobbies or other leisure time activities?	1	2	3	4
8. Were you short of breath?	1	2	3	4
9. Have you had pain?	1	2	3	4
10. Did you need to rest?	1	2	3	4
11. Have you had trouble sleeping?	1	2	3	4
12. Have you felt weak?	1	2	3	4
13. Have you lacked appetite?	1	2	3	4
14. Have you felt nauseated?	1	2	3	4
15. Have you vomited?	1	2	3	4
16. Have you been constipated?	1	2	3	4

Please go on to the next page

**During the past week:**	**Not at All**	**A Little**	**Quite a Bit**	**Very Much**
17. Have you had diarrhea?	1	2	3	4
18. Were you tired?	1	2	3	4
19. Did pain interfere with your daily activities?	1	2	3	4
20. Have you had difficulty in concentrating on things, like reading a newspaper or watching television?	1	2	3	4
21. Did you feel tense?	1	2	3	4
22. Did you worry?	1	2	3	4
23. Did you feel irritable?	1	2	3	4
24. Did you feel depressed?	1	2	3	4
25. Have you had difficulty remembering things?	1	2	3	4
26. Has your physical condition or medical treatment interfered with your family life?	1	2	3	4
27. Has your physical condition or medical treatment interfered with your social activities?	1	2	3	4
28. Has your physical condition or medical treatment caused you financial difficulties?	1	2	3	4

For the following questions please circle the number between 1 and 7 that best applies to you

29. How would you rate your overall health during the past week?

1	2	3	4	5	6	7
Very poor						Excellent

30. How would you rate your overall quality of life during the past week?

1	2	3	4	5	6	7
Very poor						Excellent

9.2 EORTC QLQ-30 Scoring

Scoring the EORTC QLQ-C30 version 3.0

Table 1: Scoring the QLQ-C30 version 3.0

	Scale	Number of items	Item range*	**Version 3.0** Item numbers	Function scales
**Global health status / QoL**					
Global health status/QoL (revised)†	QL2	2	6	29, 30	
**Functional scales**					
Physical functioning (revised)†	PF2	5	3	1 to 5	F
Role functioning (revised)†	RF2	2	3	6, 7	F
Emotional functioning	EF	4	3	21 to 24	F
Cognitive functioning	CF	2	3	20, 25	F
Social functioning	SF	2	3	26, 27	F
**Symptom scales / items**					
Fatigue	FA	3	3	10, 12, 18	
Nausea and vomiting	NV	2	3	14, 15	
Pain	PA	2	3	9, 19	
Dyspnoea	DY	1	3	8	
Insomnia	SL	1	3	11	
Appetite loss	AP	1	3	13	
Constipation	CO	1	3	16	
Diarrhoea	DI	1	3	17	
Financial difficulties	FI	1	3	28	

* *Item range* is the difference between the possible maximum and the minimum response to individual items; most items take values from 1 to 4, giving *range* = 3.

† (revised) scales are those that have been changed since version 1.0, and their short names are indicated in this manual by a suffix "2" – for example, PF2.

For all scales, the *RawScore*, *RS*, is the mean of the component items:

$$RawScore = RS = (I_1 + I_2 + ... + I_n)/n$$

Then for **Functional scales**:

$$Score = \left\{1 - \frac{(RS-1)}{range}\right\} \times 100$$

and for **Symptom scales / items** and **Global health status / QoL:**

$$Score = \{(RS-1)/range\} \times 100$$

Examples:

Emotional functioning

$$RawScore = (Q_{21} + Q_{22} + Q_{23} + Q_{24})/4$$
$$\text{EF } Score = \{1 - (RawScore - 1)/3\} \times 100$$

Fatigue

$$RawScore = (Q_{10} + Q_{12} + Q_{18})/3$$
$$\text{FA } Score = \{(RawScore - 1)/3\} \times 100$$

9.3 EORTC QLQ-OG25 questionnaire

EORTC QLQ – OG25 plus STO22 Belching subscale

Patients sometimes report that they have the following symptoms or problems. Please indicate the extent to which you have experienced these symptoms or problems during the past week. Please answer by circling the number that best applies to you.

During the past week	Not at all	A little	Quite a bit	Very much
1. Have you had problems eating solid foods?	1	2	3	4
2. Have you had problems eating liquidised or soft foods?	1	2	3	4
3. Have you had problems drinking liquids?	1	2	3	4
4. Have you had trouble enjoying your meals?	1	2	3	4
5. Have you felt full up too quickly after beginning to eat?	1	2	3	4
6. Has it taken you a long time to complete your meals?	1	2	3	4
7. Have you had difficulty eating?	1	2	3	4
8. Have you had acid indigestion or heartburn?	1	2	3	4
9. Has acid or bile coming into your mouth been a problem?	1	2	3	4
10. Have you had discomfort when eating?	1	2	3	4
11. Have you had pain when you eat?	1	2	3	4
12. Have you had pain in your stomach area?	1	2	3	4
13. Have you had discomfort in your stomach area?	1	2	3	4
14. Have you been thinking about your illness?	1	2	3	4
15. Have you worried about your health in the future?	1	2	3	4
16. Have you had trouble with eating in front of other people?	1	2	3	4
17. Have you had a dry mouth?	1	2	3	4
18. Have you had problems with your sense of taste?	1	2	3	4
19. Have you felt physically less attractive as a result of your disease or treatment?	1	2	3	4
20. Have you had difficulty swallowing your saliva?	1	2	3	4
21. Have you choked when swallowing?	1	2	3	4
22. Have you coughed?	1	2	3	4
23. Have you had difficulty talking?	1	2	3	4
24. Have you worried about your weight being too low?	1	2	3	4
25. Answer this question only if you lost any hair: If so, were you upset by the loss of your hair?	1	2	3	4
26. Have you had trouble with bile or acid coming into your mouth?	1	2	3	4
27 have you had trouble with belching?	1	2	3	4

9.4 EQ-5D-5L Scoring

2. Scoring the EQ-5D-5L descriptive system

The EQ-5D-5L descriptive system should be scored, for example, as follows:

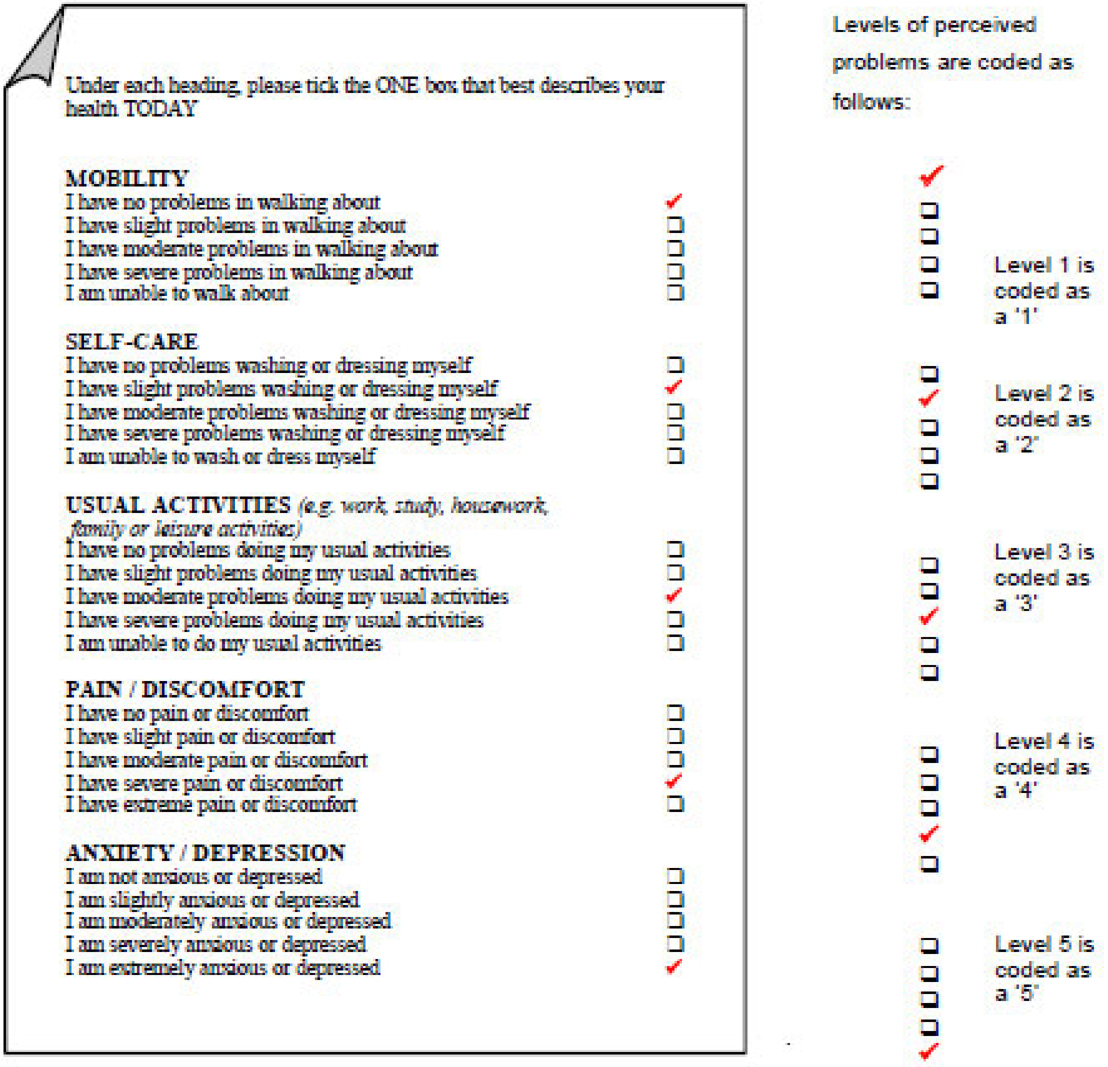

Under each heading, please tick the ONE box that best describes your health TODAY

MOBILITY
- I have no problems in walking about ✔
- I have slight problems in walking about ☐
- I have moderate problems in walking about ☐
- I have severe problems in walking about ☐
- I am unable to walk about ☐

SELF-CARE
- I have no problems washing or dressing myself ☐
- I have slight problems washing or dressing myself ✔
- I have moderate problems washing or dressing myself ☐
- I have severe problems washing or dressing myself ☐
- I am unable to wash or dress myself ☐

USUAL ACTIVITIES *(e.g. work, study, housework, family or leisure activities)*
- I have no problems doing my usual activities ☐
- I have slight problems doing my usual activities ☐
- I have moderate problems doing my usual activities ✔
- I have severe problems doing my usual activities ☐
- I am unable to do my usual activities ☐

PAIN / DISCOMFORT
- I have no pain or discomfort ☐
- I have slight pain or discomfort ☐
- I have moderate pain or discomfort ☐
- I have severe pain or discomfort ✔
- I have extreme pain or discomfort ☐

ANXIETY / DEPRESSION
- I am not anxious or depressed ☐
- I am slightly anxious or depressed ☐
- I am moderately anxious or depressed ☐
- I am severely anxious or depressed ☐
- I am extremely anxious or depressed ✔

Levels of perceived problems are coded as follows:

Level 1 is coded as a '1'

Level 2 is coded as a '2'

Level 3 is coded as a '3'

Level 4 is coded as a '4'

Level 5 is coded as a '5'

This example identifies the health state '12345'.

NB: There should be only ONE response for each dimension

NB: ***Missing values*** *can be coded as '9'.*

NB: ***Ambiguous values*** *(e.g. 2 boxes are ticked for a single dimension) should be treated as missing values.*

- We would like to know how good or bad your health is TODAY.
- This scale is numbered from 0 to 100.
- 100 means the best health you can imagine.
  0 means the worst health you can imagine.
- Mark an X on the scale to indicate how your health is TODAY.
- Now, please write the number you marked on the scale in the box below.

YOUR HEALTH TODAY = [ ]

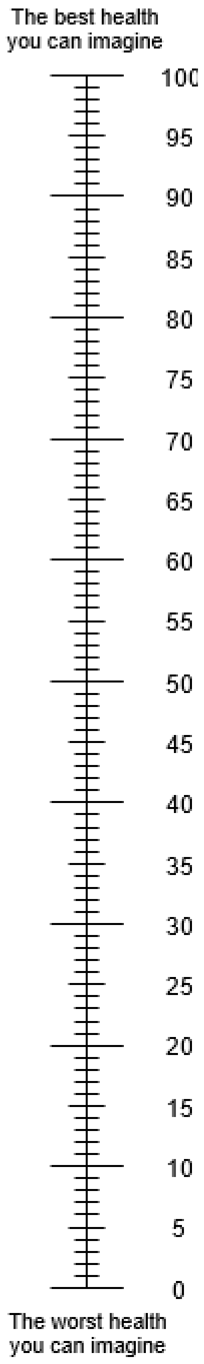

9.5 Global Pain

The Global Pain item is below (the eCOA team can format with radial dials or check boxes)

Please rate your pain by selecting the one number that best describes your pain at its worst in the last 24 hours

0	1	2	3	4	5	6	7	8	9	10
No Pain										Pain as bad as you can imagine

9.6 Health Resource Utilization

1. Since your last study visit, have you had any visits to the emergency room (ER)?

   ☐ No (if no, go to 4)
   ☐ Yes (if yes, go to 2)

2. Since your last visit, how many emergency room visits have you had?
   ____

3. For each emergency room visit please complete the following:

	Result in a Hospital Admission (more than a 24 hour stay)?	Length of stay in hospital (number of days)
ER Visit 1	Yes/No	
ER Visit 2	Yes/No	
ER Visit 3	Yes/No	

4. Since your last study visit, have you had any hospital admissions (more than a 24 hour stay) that occurred without first going to the emergency room (ER)?

☐ No (if no, go to 7)

☐ Yes (if yes, go to 5)

5. How many hospital admissions (more than 24 hour stay; without previous ER transferal)? ___________

6. For each hospital admission (more than 24 hour stay; without previous ER transferal) visit please complete the following:

	Length of stay in hospital (number of days)
Hospital Visit 1	
Hospital Visit 2	
Hospital Visit 3	

7. Since your last study visit, have you had any visits to a general practitioner (primary care physician)?

☐ No (if no, go to 9)

☐ Yes (if yes, go to 8)

8. How many visits have you had to a general practitioner (primary care physician)? ___________

9. Since your last study visit, did you have any visits to a specialist physician (e.g., oncologist, rheumatologist, endocrinologist, orthopedic surgeon, etc.)?

☐ No

☐ Yes (if yes, go to 10)

How many visits have you had to a specialist physician (e.g., oncologist, rheumatologist, endocrinologist, orthopedic surgeon, etc.)?

9.7 Author and Approver Signatories

E-signatures are attached at end of document (next page). Wet signatures, if any, are provided on this page.

Author: ______________________ Date: ______________

PPD

Approved by: ______________________ Date: ______________

PPD

Approved by: ______________________ Date: ______________

PPD

INTERIM ANALYSIS PLAN

Version 1.0, 28-Feb-2019

A Phase 3, Global, Multi-Center, Double-Blind, Randomized, Efficacy Study of Zolbetuximab (IMAB362) Plus CAPOX Compared with Placebo Plus CAPOX as First-line Treatment of Subjects with Claudin (CLDN)18.2-Positive, HER2-Negative, Locally Advanced Unresectable or Metastatic Gastric or Gastroesophageal Junction (GEJ) Adenocarcinoma

ISN/Protocol 8951-CL-0302

IND 129598

EudraCT 2018-000519-26

Sponsor:

Astellas Pharma Global Development, Inc. (APGD)

1 Astellas Way

Northbrook, IL 60062

This document contains confidential information which is the intellectual property of Astellas. By accepting or reviewing this document, you agree to hold this information in confidence and not copy or disclose it to others or use it for unauthorized purposes except (1) as otherwise agreed to in writing; (2) where required by applicable law; (3) where disclosure is directly related to the care and safety of the research participant; and (4) where disclosure of such information is made to a member of the investigator's team who agrees to hold this information in confidence.

Table of Contents

I. LIST OF ABBREVIATIONS AND KEY TERMS ··· 4

1 INTRODUCTION ··· 7

2 STUDY OBJECTIVES AND DESIGN ··· 7

2.1 Study Objectives ··· 7

2.1.1 Primary Objective ··· 7

2.1.2 Secondary Objectives ··· 7

2.1.3 Exploratory Objectives ··· 7

2.2 Study Design ··· 7

2.3 Randomization ··· 8

3 SAMPLE SIZE ··· 8

4 ANALYSIS SETS ··· 9

4.1 Full Analysis Set ··· 9

4.2 Safety Analysis Set ··· 9

4.3 Per Protocol Set ··· 9

4.4 Pharmacokinetics Analysis Set (PKAS) ··· 9

5 SELECT ENDPOINTS FOR INTERIM ANALYSIS ··· 9

5.1 Primary Efficacy Endpoint ··· 9

5.2 Secondary Efficacy Endpoints ··· 9

5.3 Safety Endpoints ··· 10

5.3.1 AE ··· 10

5.3.2 Clinical Laboratory Variables ··· 10

5.3.3 Vital Signs ··· 10

5.3.4 12-lead electrocardiogram (ECG) ··· 11

5.3.5 ECOG performance score ··· 11

5.3.6 Physical examination ··· 11

6 INTERIM ANALYSES ··· 11

6.1 Periodical Safety Reviews ··· 11

6.1.1 Scope of Periodical Safety Analysis ··· 11

6.2 Interim OS analysis/Final PFS analysis ··· 13

6.2.1 Scope of Interim OS analysis/Final PFS analysis ··· 13

6.3 Subgroup Analyses ··· 13

6.4 Other Analyses 13
7 REVISION AND RATIONALE 13
7.1 List of Changes in IAP Version 2.0 from Version 1.0 (if applicable) 13
8 APPENDICES 14
8.1 TABLES, LISTINGS AND FIGURES (TLFs) SPECIFICATIONS 14
8.2 TABLES, LISTINGS AND FIGURES (ONLY UPON REQUEST BY IDMC) 44
8.3 Author and Approver Signatories 47

I. LIST OF ABBREVIATIONS AND KEY TERMS

List of Abbreviations

Abbreviations	Description of abbreviations
CAPOX	Capecitabine and Oxaliplatin
ADA	anti-drug antibody
AE	adverse event
ALP	alkaline phosphatase
ALT	alanine aminotransferase (GPT)
AST	aspartate aminotransferase (GOT)
ATC	Anatomical Therapeutic Chemical Classification System
BMI	Body mass index
BSA	Body surface area
C1D1	Cycle 1 Day 1
CI	confidence interval
CLDN	Claudin
C_{max}	maximum concentration
CMH	Cochran-Mantel-Haenszel
CR	complete response
CSR	Clinical study report
CTCAE	Common Terminology Criteria For Adverse Events
DCR	disease control rate
DOR	duration of response
ECG	electrocardiogram
ECOG	Eastern Cooperative Oncology Group
eCRF	electronic case report form
EORTC	European Organization for Research and Treatment of Cancer
EQ-5D-5L	EuroQOL Five Dimensions Questionnaire 5L
FAS	full analysis set
GEJ	gastroesophageal junction
GP	Global Pain
HER2	human epidermal growth factor receptor 2
HR	Hazard ratio
HRQoL	health-related quality of life
HRU	Health Resource Utilization
IAP	Interim analysis plan
IDAC	independent data analysis center
IDMC	independent data monitoring committee
INR	international normalized ratio
IRC	independent review committee
IRT	interactive response technology
ISN	international study number
IV	Intravenous, intravenously

NCI	National Cancer Institute
NE	not evaluable
ORR	objective response rate
OS	overall survival
PD	progressive disease
PFS	progression free survival
PFS2	progression free survival following second-line anti-cancer treatment
PGx	pharmacogenomics
PKAS	pharmacokinetics analysis set
PPS	per protocol set
PR	partial response
PT	preferred term
QLQ-C30	Quality of Life Questionnaire - Core Questionnaire
QLQ-OG25	Quality of Life Questionnaire - Oesophago-Gastric Module 25 (OG-25)
QoL	Quality of life
QTc	QT interval corrected
QTcF	Fridericia-corrected QT interval
RECIST	Response Evaluation Criteria In Solid Tumors
SAE	serious adverse event
SAF	safety analysis set
SAP	statistical analysis plan
SD	stable disease
SOC	system organ class
SOD	Sum of diameters
TEAE	treatment-emergent adverse event
TSH	thyroid stimulating hormone
TTP	time to progression
ULN	upper limit of normal
VAS	visual analog scale
WHO	World health organization

List of Key Terms

Terms	Definition of terms
Baseline	Assessments of subjects as they enter a trial before they receive any treatment.
Endpoint	Variable that pertains to the efficacy or safety evaluations of a trial.
Enroll	To register or enter a subject into a clinical trial. NOTE: Once a subject has been randomized, the clinical trial protocol applies to the subject.
Intervention	The drug, device, therapy or process under investigation in a clinical study that is believed to have an effect on outcomes of interest in a study (e.g., health-related quality of life, efficacy, safety, pharmacoeconomics).
Investigational period	Period of time where major interests of protocol objectives are observed, and where the test drug or comparative drug (sometimes without randomization) is usually given to a subject, and continues until the last assessment after completing administration of the test drug or comparative drug.
Post investigational period	Period of time after the last assessment of the protocol. Follow-up observations for sustained adverse events and/or survival are done in this period.
Randomization	The process of assigning trial subjects to treatment or control groups using an element of chance to determine assignments in order to reduce bias.
Screening	A process of active consideration of potential subjects for enrollment in a trial.
Screen failure	Potential subject who did not meet 1 or more criteria required for participation in a trial.
Screening period	Period of time before entering the investigational period, usually from the time when a subject signs the consent until just before the test drug or comparative drug (sometimes without randomization) is given to a subject.
Study period	Period of time from the first site initiation date to the last site completing the study.
Study treatment	Includes zolbetuximab/placebo and all components of CAPOX
Variable	Any entity that varies; any attribute, phenomenon or event that can have different qualitative or quantitative values.

1 INTRODUCTION

This Interim Analysis Plan (IAP) describes the content of the analysis intended for IDMC periodical safety reviews and the final PFS/interim OS analysis. The final IAP will be approved prior to the first IDMC safety review meeting. For technical details of analysis, please refer to the study Statistical Analysis Plan (SAP).

2 STUDY OBJECTIVES AND DESIGN

2.1 Study Objectives

2.1.1 Primary Objective

The primary objective is to evaluate the efficacy of zolbetuximab plus CAPOX compared with placebo plus CAPOX (as first-line treatment) as measured by progression free survival (PFS) in subjects with Claudin (CLDN)18.2 positive, HER2–negative locally advanced unresectable or metastatic gastric and GEJ adenocarcinoma.

2.1.2 Secondary Objectives

The secondary objectives are:

- To evaluate efficacy as measured by Overall Survival (OS) as a key secondary objective
- To evaluate efficacy as measured by Objective Response Rate (ORR)
- To evaluate efficacy as measured by Duration of Response (DOR)
- To evaluate safety and tolerability of zolbetuximab
- To evaluate health-related quality of life (HRQoL) using the parameters as measured by European Organization for Research and Treatment of Cancer (EORTC) QLQ-C30, QLQ-OG25 plus STO22 Belching subscale, Global Pain (GP) and the EuroQOL Five Dimensions (EQ-5D-5L) questionnaires
- To evaluate the pharmacokinetics of zolbetuximab
- To evaluate the immunogenicity profile of zolbetuximab

2.1.3 Exploratory Objectives

The exploratory objectives are:

- To evaluate efficacy as measured by Time to Progression (TTP)
- To evaluate PFS following second-line anti-cancer treatment (PFS2)
- To evaluate Disease Control Rate (DCR)
- To evaluate potential genomic and/or other biomarkers that may correlate with treatment outcome to zolbetuximab and CAPOX.
- To evaluate Health Resource Utilization (HRU)

2.2 Study Design

This global, multi-center, double-blind, 1:1 randomized, phase 3 study will evaluate efficacy of zolbetuximab plus CAPOX versus placebo plus CAPOX as first-line treatment in subjects

with CLDN18.2-positive, HER2-negative locally advanced unresectable or metastatic gastric and GEJ adenocarcinoma.

PFS as assessed by the Independent Review Committee (IRC) is the primary outcome. Secondary outcomes include OS (key secondary endpoint), ORR, DOR, safety and tolerability, HRQoL, pharmacokinetic and the immunogenicity profile of zolbetuximab. Exploratory outcomes include TTP, PFS2, DCR, biomarkers and HRU.

One interim analysis and one final analysis are planned for OS, while only one analysis is planned for PFS as the final analysis. The OS interim analysis will occur at the same time of the final PFS analysis (after pre-specified number of PFS events) and final OS analysis will be performed after the pre-specified number of OS events are observed. Refer to Section 6 for details on interim analysis.

Details of the study flow chart, dosing schedule, schedule of assessments are available in the protocol Section V.

2.3 Randomization

Subject randomization will be performed via IRT and treatment is assigned in a 1:1 ratio to:

- Arm A (Zolbetuximab in combination with CAPOX chemotherapy)
- Arm B (placebo in combination with CAPOX chemotherapy)

Prior to the initiation of the study treatment, the unblinded pharmacist/designee will contact the IRT system in order to determine the randomly assigned treatment. The unblinded pharmacist/designee will dispense the treatment according to the IRT system's assignment. Specific procedures for randomization through the IRT are contained in the IRT manual.

Randomization of subjects will use blocked randomization and be stratified by the following factors:

- Region (Asia vs. Non-Asia)
- Number of Metastatic Sites (0 to 2 vs. ≥ 3)
- Prior Gastrectomy (Yes vs. No)

3 SAMPLE SIZE

Approximately 500 subjects will be randomized in a 1:1 ratio to receive Zolbetuximab in combination with CAPOX chemotherapy (Arm A) or placebo in combination with CAPOX chemotherapy (Arm B). The planned 344 PFS events during the study will provide 96% power to detect a difference in PFS between Arm A (Zolbetuximab + CAPOX) with the assumption of 9 months median PFS time and Arm B (placebo + CAPOX) with the assumption of 6 months median PFS time (hazard ratio = 0.67) at the overall 1-sided 0.025 significance level. Similarly, the planned 386 OS events during the study will provide 80% power to detect a difference in OS between Arm A (Zolbetuximab + CAPOX) with the assumption of 14.7 months median OS time and Arm B (placebo + CAPOX) with the assumption of 11 months median OS time (hazard ratio = 0.75) at the overall 1-sided 0.025 significance level.

4 ANALYSIS SETS

In accordance with International Council for Harmonization (ICH) recommendations in guidelines E3 and E9, the following analysis sets will be used for the analyses.

The determination of whether subjects are included or excluded from the safety and efficacy analysis sets will be made prior to each IDMC meeting in a blinded manner.

4.1 Full Analysis Set

The Full Analysis Set (FAS) will consist of all subjects who are randomized to one of the treatment arms. Subjects would be analyzed according to the treatment they were randomized to. The FAS will be used for summaries of demographics and baseline characteristics and all efficacy analyses. FAS in this study is identical to intent-to-treat (ITT) set (only the name "FAS" will be used).

4.2 Safety Analysis Set

The safety analysis set (SAF) will consist of all subjects who received at least one dose of any study drug (Zolbetuximab/placebo/CAPOX). Subjects would be analyzed according to the actual treatment they received. The SAF will be used for summaries of demographic and baseline characteristics and all safety and tolerability-related variables.

4.3 Per Protocol Set

Not applicable to the IAP.

4.4 Pharmacokinetics Analysis Set (PKAS)

Not applicable to the IAP.

5 SELECT ENDPOINTS FOR INTERIM ANALYSIS

5.1 Primary Efficacy Endpoint

The primary endpoint is PFS, which is defined as the time from the date of randomization until the date of radiological PD (per Response Evaluation Criteria In Solid Tumors [RECIST] 1.1 by independent review committee [IRC]) or death from any cause, whichever is earlier.

5.2 Secondary Efficacy Endpoints

- OS, defined as the time from the date of randomization until the date of death from any cause.
- ORR, defined as the proportion of subjects who have a best overall response (BOR) of complete response (CR) or partial response (PR) as assessed by IRC per RECIST 1.1.
- DOR, defined as the time from the date of the first response (CR/PR) until the date of PD as assessed by IRC per RECIST 1.1 or date of death from any cause, whichever is earliest.

5.3 Safety Endpoints

Safety and tolerability endpoints include AEs, laboratory test results, vital signs, electrocardiograms (ECGs) and Eastern Cooperative Oncology Group (ECOG) performance status.

5.3.1 AE

AE will be assessed by evaluation of the following variables:

- Treatment-emergent adverse events (TEAEs; frequency, severity, seriousness, and relationship to study drug)
    - TEAE is defined as an adverse event observed after starting administration of the study drug through 30 days after the last dose of study drug.
    - If the adverse event occurs on Cycle 1 Day 1 and the onset check box is marked “Onset after first dose of study drug” or the onset check box is left blank, then the adverse event will be considered treatment emergent.
    - If the adverse event occurs on Day 1 and the onset check box is marked “Onset before first dose of study drug”, then the adverse event will not be considered treatment emergent.
    - If a subject experiences an event both during the pre-investigational/screening period and during the investigational period, the event will be considered as TEAE only if it is reported with a new start date (i.e., as a new AE).
    - Any AEs with onset dates completely missing will be considered TEAEs in summaries. AEs with partially missing onset dates will be assumed TEAEs unless the available portion of the date indicates that the onset was strictly before start of study medication.
    - A drug-related TEAE is defined as any TEAE with possible relationship to study treatment as assessed by the investigator or with missing assessment of the causal relationship.
- Serious adverse events (SAEs) include adverse events that are flagged as serious by the investigator on eCRF, or upgraded by the Sponsor based on review of the Sponsor’s list of Always Serious terms.

5.3.2 Clinical Laboratory Variables

Refer to protocol Section 5.4.3 for a table of the laboratory tests that will be performed during the conduct of the study. Refer to the Protocol Schedule of Assessments for evaluation schedule.

5.3.3 Vital Signs

Vital signs will include systolic and diastolic blood pressure (mmHg), radial pulse (beats/min) and body temperature. Serial vital signs will be collected during zolbetuximab dosing visits.

5.3.4 12-lead electrocardiogram (ECG)

A single 12-lead ECG will be performed at the time points outlined in the Protocol Schedule of Assessments. ECGs will be read locally.

5.3.5 ECOG performance score

ECOG performance scores will be collected.

5.3.6 Physical examination

Targeted (symptom driven) physical exams should be conducted every 3 weeks on day 1 of each cycle. If clinically significant worsening of findings from baseline is noted at any study visit, the changes will be documented as AEs on the AE eCRF.

6 INTERIM ANALYSES

6.1 Periodical Safety Reviews

There will be approximately 4 periodical safety review meetings by the IDMC, with the first meeting scheduled to take place approximately 6 weeks after the 40th subject has been randomized and on study drug for 2 cycles (6 weeks). Regular meetings will be conducted approximately every 6 months from the first IDMC review meeting. IDMC has the option to shorten the interval between meetings. The Sponsor study team will not have access to the unblinded randomization schedule or study results. All unblinded study results will be generated by the IDAC using validated programs provided by the Sponsor, and provided to IDMC members prior to each meeting.

At each safety review meeting, IDMC may recommend continuation of trial, or termination/modification of trial based on overwhelming negative safety signals. Trial may be terminated for early efficacy success only at the formal interim OS/final PFs analysis.

Refer to the IDMC charter for more information on the IDMC meetings.

6.1.1 Scope of Periodical Safety Analysis

Table 1 describes analyses that will be included for periodical safety reviews. And Table 2 describes efficacy analyses that will be provided to IDMC only if requested by IDMC post-hoc. If efficacy results are requested, p-values or confidence intervals for hazard ratios will not be included in any efficacy tables or figures for periodical safety reviews. Full TLFs with p-values will only be provided at the formal interim OS/final PFS analysis.

Table 1 TLFs to be included for periodical safety reviews (all based on SAF)

Table No.	Title
12.1.1.2	Subject Disposition
12.1.1.4.1	End of Treatment Summary of CAPOX
12.1.1.5.1	End of Treatment Summary of zolbetuximab/placebo
12.1.2.1.1	Demographic Characteristics
12.1.2.3.1	Stratification Factors Reported at Randomization
12.2.1.1.1	Study Drug Treatment – Duration of Each Component
12.2.1.2.1&12.2.1.3.1	Study Drug Treatment – Summary of Infusions and Dose for Zolbetuximab/Placebo and Oxaliplatin
12.2.1.4.1	Study Drug Treatment – Summary of Dose for Capecitabine
12.2.2.2	Concomitant Medications
12.6.1.1	Overview of Treatment-Emergent Adverse Events and Death
12.6.1.2	Treatment-Emergent Adverse Events (MedDRA V20.1)
12.6.1.6	Serious Treatment-Emergent Adverse Events (MedDRA V20.1)
12.6.1.8	Drug-related Serious Treatment-Emergent Adverse Events (MedDRA V20.1)
12.6.1.18	Treatment-Emergent Adverse Events (MedDRA V20.1) by Preferred Term
12.6.1.7.1	Adverse Events Collected After 30 Days of the Last Dose (MedDRA V20.1)
12.6.1.7.2	Serious Adverse Events Collected After 30 Days of the Last Dose (MedDRA V20.1)
12.6.1.19	Treatment-Emergent Adverse Events Leading to Death (MedDRA V20.1) by Preferred Term
12.6.1.14	Treatment-Emergent Adverse Events with NCI-CTCAE >=3 (MedDRA V20.1)
12.6.1.15	Drug-related Treatment-Emergent Adverse Events with NCI-CTCAE >=3 (MedDRA V20.1)
12.6.2.2.2.x	Shift Table for Laboratory Test Results From Baseline to Worst Post-Baseline NCI CTCAE Grade (V4.03)
12.6.2.3	Potentially Clinically Significant Values in Liver Enzymes and Total Bilirubin
12.6.5.2.2	Shift Table of ECOG Performance Status
13.2.7.4	Listing of Serious Adverse Events
13.2.7.3	Listing of Treatment-Emergent Adverse Events Leading to Death
13.2.8.1.x	Listing of Central Laboratory Tests Results in SI Units
13.2.6.16	Listing of ECOG performance status

Table 2 TLFs to be provided to IDMC only if requested after IDMC meetings

Figure No.	Title
12.3.1.1	Kaplan-Meier Plot of Progression-Free Survival, Independent Radiologic Review (without p-value and CI) - FAS
12.3.1.2	Kaplan-Meier Plot of Progression-Free Survival, Investigator Assessment (without p-value and CI) - FAS
12.3.2	Kaplan-Meier Plot of Overall Survival (without p-value and CI) - FAS

6.2 Interim OS analysis/Final PFS analysis

To evaluate whether Zolbetuximab + CAPOX (Arm A) is beneficial compared to the concurrent placebo + CAPOX (Arm B) while the study is ongoing, a formal OS interim analysis is planned when the final PFS analysis occurs with the pre-specified number (344) of PFS events. A group sequential design using the O'Brien-Fleming type alpha-spending function [Lan & DeMets, 1983] will be utilized to control the overall 1-sided 0.025 significance level (East®) for the OS endpoint. The OS interim and final analyses will be performed only if primary PFS analysis is significant.

The IDMC may recommend terminating the trial for favorable results at the formal efficacy interim analysis using OS. The IDMC may also recommend terminating the trial for negative PFS results. In the case of favorable results, the 1-sided significance level for superiority is 0.0074 for the interim OS analysis and 0.0228 for the final OS analysis. If the 1-sided P value of the interim analysis is less than 0.0074, the IDMC may recommend terminating the trial for success. If the study is not stopped after the interim analysis, a final OS analysis will occur after 100% of the planned death events (386 events) have been observed. Note that the 0.0074 alpha boundary is based on an information factor of 70% (270 death out of planned 386 events) observed at the PFS final/OS interim analysis and may be adjusted prior to interim analysis if the number of observed deaths deviates from that number.

6.2.1 Scope of Interim OS analysis/Final PFS analysis

The full set of TLFs will be produced.

6.3 Subgroup Analyses

Not applicable.

6.4 Other Analyses

Not applicable.

7 REVISION AND RATIONALE

7.1 List of Changes in IAP Version 2.0 from Version 1.0 (if applicable)

The changes from the approved IAP Version 1.0 (Dated dd-MMM-yyyy) to Version 2.0 that impact analyses are listed with the rationale in the table below.

SAP Section(s)	Description of Change(s)	Rationale

8 APPENDICES

8.1 TABLES, LISTINGS AND FIGURES (TLFs) SPECIFICATIONS

Study 8951-CL-0302 Table 12.1.1.2 Source: <Listing / Dataset>

Subject Disposition

All Randomized Subjects

Analysis Set	Arm A (N=xx)	Arm B (N=xx)	Overall (N=xx)
Randomized	xx (xx x%)	xx (xx x%)	xx (xx.x%)
Subjects Who Did Not Take Study Drug	xx (xx x%)	xx (xx x%)	xx (xx.x%)
Safety Analysis Set [1]	xx (xx x%)	xx (xx x%)	xx (xx.x%)

[1] All subjects who received at least one dose of any study drug (Zolbetuximab/CAPOX).

Study 8951-CL-0302

Table 12.1.1.4.1

Source: <Listing / Dataset>

End of Treatment – CAPOX

Safety Analysis Set

Parameter	Category	Arm A (N=xx)	Arm B (N=xx)	Overall (N=xx)
CAPOX Discontinuation	Yes	xx (xx.x%)	xx (xx x%)	xx (xx x%)
	No	xx (xx.x%)	xx (xx x%)	xx (xx x%)
Primary Reason for Discontinuation	Completed	xx (xx.x%)	xx (xx x%)	xx (xx x%)
	Adverse Event	xx (xx.x%)	xx (xx x%)	xx (xx x%)
	Death	xx (xx.x%)	xx (xx x%)	xx (xx x%)
	Lost to Follow-Up	xx (xx.x%)	xx (xx x%)	xx (xx x%)
	Progressive Disease	xx (xx.x%)	xx (xx x%)	xx (xx x%)
	Protocol Deviation	xx (xx.x%)	xx (xx x%)	xx (xx x%)
	Withdrawal by Subject	xx (xx.x%)	xx (xx x%)	xx (xx x%)
	Pregnancy	xx (xx.x%)	xx (xx x%)	xx (xx x%)
	Other	xx (xx.x%)	xx (xx x%)	xx (xx x%)

Study 8951-CL-0302

Table 12.1.1.5.1

Source: <Listing / Dataset>

End of Treatment – Zolbetuximab/Placebo

Safety Analysis Set

Parameter	Category	Arm A (N=xx)	Arm B (N=xx)	Overall (N=xx)
Zolbetuximab/Placebo Discontinuation	Yes	xx (xx.x%)	xx (xx x%)	xx (xx x%)
	No	xx (xx.x%)	xx (xx x%)	xx (xx x%)
Primary Reason for Discontinuation	Completed	xx (xx.x%)	xx (xx x%)	xx (xx x%)
	Adverse Event	xx (xx.x%)	xx (xx x%)	xx (xx x%)
	Death	xx (xx.x%)	xx (xx x%)	xx (xx x%)
	Lost to Follow-Up	xx (xx.x%)	xx (xx x%)	xx (xx x%)
	Progressive Disease	xx (xx.x%)	xx (xx x%)	xx (xx x%)
	Protocol Deviation	xx (xx.x%)	xx (xx x%)	xx (xx x%)
	Withdrawal by Subject	xx (xx.x%)	xx (xx x%)	xx (xx x%)
	Pregnancy	xx (xx.x%)	xx (xx x%)	xx (xx x%)
	Other	xx (xx.x%)	xx (xx x%)	xx (xx x%)
If Progressive Disease or Death	Radiographic Progression Only	xx (xx.x%)	xx (xx x%)	xx (xx x%)
	Clinical Progression Only	xx (xx.x%)	xx (xx x%)	xx (xx x%)

Study 8951-CL-0302 Table 12.1.2.1.1 Source: <Listing / Dataset>
Demographic Characteristics
Safety Analysis Set

Parameter	Category/ Statistic	Arm A (N=xx)	Arm B (N=xx)	Overall (N=xx)
Sex	Male	xx (xx.x%)	xx (xx x%)	xx (xx x%)
	Female	xx (xx.x%)	xx (xx x%)	xx (xx x%)
Ethnicity	Hispanic or Latino	xx (xx.x%)	xx (xx x%)	xx (xx x%)
	Not Hispanic or Latino	xx (xx.x%)	xx (xx x%)	xx (xx x%)
Race	White	xx (xx.x%)	xx (xx x%)	xx (xx x%)
	Black or African American	xx (xx.x%)	xx (xx x%)	xx (xx x%)
	Asian	xx (xx.x%)	xx (xx x%)	xx (xx x%)
	American Indian or Alaska Native	xx (xx.x%)	xx (xx x%)	xx (xx x%)
	Native Hawaiian or Other Pacific Islander	xx (xx.x%)	xx (xx x%)	xx (xx x%)
	Other	xx (xx.x%)	xx (xx x%)	xx (xx x%)
Age (Years)	n	xx	xx	xx
	Mean	xx x	xx x	xx x
	SD	xx x	xx x	xx x
	Min	xx	xx	xx
	Median	xx x	xx x	xx x
	Max	xx	xx	xx
Age Group 1 (Years)	<=65	xx (xx.x%)	xx (xx x%)	xx (xx x%)
	>65	xx (xx.x%)	xx (xx x%)	xx (xx x%)
Age Group 2 (Years)	<=75	xx (xx.x%)	xx (xx x%)	xx (xx x%)
	>75	xx (xx.x%)	xx (xx x%)	xx (xx x%)
ECOG Status at Baseline	0	xx (xx.x%)	xx (xx x%)	xx (xx x%)
	1	xx (xx.x%)	xx (xx x%)	xx (xx x%)
Weight(kg)	n	xx	xx	xx
	Mean	xx x	xx x	xx x
	SD	xx x	xx x	xx x
	Min	xx	xx	xx
	Median	xx x	xx x	xx x
	Max	xx	xx	xx

Table 12.1.2.1.1 (continued)
Demographic Characteristics
Safety Analysis Set

Parameter	Category/ Statistic	Arm A (N=xx)	Arm B (N=xx)	Overall (N=xx)
Height(cm)	n	xx	xx	xx
	Mean	xx x	xx x	xx x
	SD	xx x	xx x	xx x
	Min	xx	xx	xx
	Median	xx x	xx x	xx x
	Max	xx	xx	xx
BMI (kg/m^2)	n	xx	xx	xx
	Mean	xx x	xx x	xx x
	SD	xx x	xx x	xx x
	Min	xx	xx	xx
	Median	xx x	xx x	xx x
	Max	xx	xx	xx
BSA (m^2)	n	xx	xx	xx
	Mean	xx x	xx x	xx x
	SD	xx x	xx x	xx x
	Min	xx	xx	xx
	Median	xx x	xx x	xx x
	Max	xx	xx	xx
Tobacco History	Never	xx (xx x%)	xx (xx x%)	xx (xx x%)
	Current	xx (xx x%)	xx (xx x%)	xx (xx x%)
	Former	xx (xx x%)	xx (xx x%)	xx (xx x%)

Study 8951-CL-0302 Table 12.1.2.3.1 Source: <Listing / Dataset>
Stratification Factors Reported at Randomization
Safety Analysis Set

Parameter, n (%)	Arm A (N = XXX)	Arm B (N=XXX)	Overall (N = XXX)
Region= Asia	xxx (xx x%)	xxx (xx x%)	xxx (xx x%)
Region= Non-Asia	xxx (xx x%)	xxx (xx x%)	xxx (xx x%)
Number of Metastatic Sites=0-2	xxx (xx x%)	xxx (xx x%)	xxx (xx x%)
Number of Metastatic Sites=>=3	xxx (xx x%)	xxx (xx x%)	xxx (xx x%)
Prior Gastrectomy=Yes	xxx (xx x%)	xxx (xx x%)	xxx (xx x%)
Prior Gastrectomy=No	xxx (xx x%)	xxx (xx x%)	xxx (xx x%)
Region=Asia, Number of Metastatic Sites=0-2, Prior Gastrectomy=Yes	xxx (xx x%)	xxx (xx x%)	xxx (xx x%)
Region=Asia, Number of Metastatic Sites=0-2, Prior Gastrectomy=No	xxx (xx x%)	xxx (xx x%)	xxx (xx x%)
Region=Asia, Number of Metastatic Sites=>=3, Prior Gastrectomy=Yes	xxx (xx x%)	xxx (xx x%)	xxx (xx x%)
Region=Asia, Number of Metastatic Sites=>=3, Prior Gastrectomy=No	xxx (xx x%)	xxx (xx x%)	xxx (xx x%)
Region=Non-Asia, Number of Metastatic Sites=0-2, Prior Gastrectomy=Yes	xxx (xx x%)	xxx (xx x%)	xxx (xx x%)
Region=Non-Asia, Number of Metastatic Sites=0-2, Prior Gastrectomy=No	xxx (xx x%)	xxx (xx x%)	xxx (xx x%)
Region=Non-Asia, Number of Metastatic Sites=>=3, Prior Gastrectomy=Yes	xxx (xx x%)	xxx (xx x%)	xxx (xx x%)
Region=Non-Asia, Number of Metastatic Sites=>=3, Prior Gastrectomy=No	xxx (xx x%)	xxx (xx x%)	xxx (xx x%)

Study 8951-CL-0302 Table 12.2.1.1.1 Source: <Listing / Dataset>
Study Drug Treatment - Duration of Each Component
Safety Analysis Set

Characteristic		Arm A (N=xx)	Arm B (N=xx)	Overall (N=xx)
Duration of Zolbetuximab/Placebo [1] (days)	n	XX	XX	XX
	Mean	XX.XX	XX.XX	XX.XX
	SD	XX.XX	XX.XX	XX.XX
	Min	XX.X	XX.X	XX.X
	Median	XX.XX	XX.XX	XX.XX
	Max	XX.X	XX.X	XX.X
Duration of Oxaliplatin [1] (days)	n	XX	XX	XX
	Mean	XX.XX	XX.XX	XX.XX
	SD	XX.XX	XX.XX	XX.XX
	Min	XX.X	XX.X	XX.X
	Median	XX.XX	XX.XX	XX.XX
	Max	XX.X	XX.X	XX.X
Duration of Capecitabine [2] (days)	n	XX	XX	XX
	Mean	XX.XX	XX.XX	XX.XX
	SD	XX.XX	XX.XX	XX.XX
	Min	XX.X	XX.X	XX.X
	Median	XX.XX	XX.XX	XX.XX
	Max	XX.X	XX.X	XX.X
Duration of All Components Administered (days) [3]	N	XX	XX	XX
	Mean	XX.XX	XX.XX	XX.XX
	SD	XX.XX	XX.XX	XX.XX
	Min	XX.X	XX.X	XX.X
	Median	XX.XX	XX.XX	XX.XX
	Max	XX.X	XX.X	XX.X
Duration of Any Component Administered (days) [4]	N	XX	XX	XX
	Mean	XX.XX	XX.XX	XX.XX
	SD	XX.XX	XX.XX	XX.XX
	Min	XX.X	XX.X	XX.X
	Median	XX.XX	XX.XX	XX.XX
	Max	XX.X	XX.X	XX.X
CAPOX (24 weeks)Treatment Completed	N	XX (XX.X%)	XX (XX.X%)	XX (XX.X%)

Arm A components: Zolbetuximab, Oxaliplatin and Capecitabine; Arm B components: Oxaliplatin and Capecitabine.
[1] Duration of Zolbetuximab or placebo and Oxaliplatin, defined as (date of last infusion - date of first infusion + 1). For those who did not receive any dose, duration will be 0.
[2] Duration of Capecitabine, defined as (date of last dose - date of first dose + 1). For those who did not receive any dose, duration will be 0.
Footnotes continued on next page

[3] Duration of all components administered is defined as (date of last dose of the earliest component discontinued) - (date of Cycle 1 Day 1).
[4] Duration of any component administered is defined as (date of last dose of the last component discontinued) - (date of Cycle 1 Day 1).

Study 8951-CL-0302

Table 12.2.1.2.1

Source: <Listing / Dataset>

Study Drug Treatment – Zolbetuximab/Placebo
Safety Analysis Set

Characteristic		Arm A (N=xx)	Arm B (N=xx)	Overall (N=xx)
Cumulative Actual Dose (mg)	n	XX	XX	XX
	Mean	XX.XX	XX.XX	XX.XX
	SD	XX.XX	XX.XX	XX.XX
	Min	XX.X	XX.X	XX.X
	Median	XX.XX	XX.XX	XX.XX
	Max	XX.X	XX.X	XX.X
Relative Dose Intensity (%) [1]	n	XX	XX	XX
	Mean	XX.X	XX.X	XX.X
	SD	XX.X	XX.X	XX.X
	Min	XX	XX	XX
	Median	XX.X	XX.X	XX.X
	Max	XX	XX	XX
Relative Dose Intensity Category (%)	<50	XX (XX.X%)	XX (XX.X%)	XX (XX.X%)
	>=50 to <=80	XX (XX.X%)	XX (XX.X%)	XX (XX.X%)
	>80	XX (XX.X%)	XX (XX.X%)	XX (XX.X%)
	Unknonwn	XX (XX.X%)	XX (XX.X%)	XX (XX.X%)
Number of Infusions Administered [2]	n	XX	XX	XX
	Mean	XX.X	XX.X	XX.X
	SD	XX.X	XX.X	XX.X
	Min	XX	XX	XX
	Median	XX.X	XX.X	XX.X
	Max	XX	XX	XX
Number of Infusions Entirely Administered	n	XX	XX	XX
	Mean	XX.X	XX.X	XX.X
	SD	XX.X	XX.X	XX.X
	Min	XX	XX	XX
	Median	XX.X	XX.X	XX.X
	Max	XX	XX	XX
Number of Infusions Not Entirely Administered	n	XX	XX	XX
	Mean	XX.X	XX.X	XX.X
	SD	XX.X	XX.X	XX.X
	Min	XX	XX	XX
	Median	XX.X	XX.X	XX.X
	Max	XX	XX	XX

Study 8951-CL-0302

Table 12.2.1.2.1 (continued)
Study Drug Treatment - Zolbetuximab/Placebo
Safety Analysis Set

Source: <Listing / Dataset>

Characteristic		Arm A (N=xx)	Arm B (N=xx)	Overall (N=xx)
Average Infusion Time per Infusion (hr/infusion) [3]	n	XX	XX	XX
	Mean	XX.X	XX.X	XX.X
	SD	XX.X	XX.X	XX.X
	Min	XX	XX	XX
	Median	XX.X	XX.X	XX.X
	Max	XX	XX	XX
Average Dose Per Infusion (mg/infusion) [4]	n	XX	XX	XX
	Mean	XX.X	XX.X	XX.X
	SD	XX.X	XX.X	XX.X
	Min	XX	XX	XX
	Median	XX.X	XX.X	XX.X
	Max	XX	XX	XX
Number of Infusions Not Administered due to AE	n	XX	XX	XX
	Mean	XX.X	XX.X	XX.X
	SD	XX.X	XX.X	XX.X
	Min	XX	XX	XX
	Median	XX.X	XX.X	XX.X
	Max	XX	XX	XX
...	...	...	...	...
Duration of Treatment Cumulative Category (days)	>=1	XX (XX.X%)	XX (XX.X%)	XX (XX.X%)
	>=43	XX (XX.X%)	XX (XX.X%)	XX (XX.X%)
	>=85	XX (XX.X%)	XX (XX.X%)	XX (XX.X%)
	>=169	XX (XX.X%)	XX (XX.X%)	XX (XX.X%)
	>=253	XX (XX.X%)	XX (XX.X%)	XX (XX.X%)
	>=337	XX (XX.X%)	XX (XX.X%)	XX (XX.X%)
	>=505	XX (XX.X%)	XX (XX.X%)	XX (XX.X%)

[1] Defined as (Cumulative actual dose/cumulative dose if initially assigned dose had been given to all scheduled dosing visits)* 100%.
[2] Number of infusions per subject over the entire study period.
[3] Defined as [(stop infusion time) - (start infusion time)]/ (number of infusions administered).
[4] Defined as (cumulative dose)/(number of infusions administered).

Note: Repeat for
Program: </directory/directory/directory/program.sas>

<Draft/Final Version>

Study 8951-CL-0302

Table 12.2.1.3.1
Study Drug Treatment - Oxaliplatin
Safety Analysis Set

Source: <Listing / Dataset>

Study 8951-CL-0302 Table 12.2.1.4.1 Source: <Listing / Dataset>

Study Drug Treatment – Capecitabine
Safety Analysis Set

Characteristic		Arm A (N=xx)	Arm B (N=xx)	Overall (N=xx)
Cumulative Actual Dose (mg)	n	XX	XX	XX
	Mean	XX.XX	XX.XX	XX.XX
	SD	XX.XX	XX.XX	XX.XX
	Min	XX.X	XX.X	XX.X
	Median	XX.XX	XX.XX	XX.XX
	Max	XX.X	XX.X	XX.X
Relative Dose Intensity (%) [1]	n	XX	XX	XX
	Mean	XX.X	XX.X	XX.X
	SD	XX.X	XX.X	XX.X
	Min	XX	XX	XX
	Median	XX.X	XX.X	XX.X
	Max	XX	XX	XX
Relative Dose Intensity Category (%)	<50	XX (XX.X%)	XX (XX.X%)	XX (XX.X%)
	>=50 to <=80	XX (XX.X%)	XX (XX.X%)	XX (XX.X%)
	>80	XX (XX.X%)	XX (XX.X%)	XX (XX.X%)
	Unknonwn	XX (XX.X%)	XX (XX.X%)	XX (XX.X%)
Number of Cycles [2]	n	XX	XX	XX
	Mean	XX.X	XX.X	XX.X
	SD	XX.X	XX.X	XX.X
	Min	XX	XX	XX
	Median	XX.X	XX.X	XX.X
	Max	XX	XX	XX
Number of Cycles with Dose Adjustment	n	XX	XX	XX
	Mean	XX.X	XX.X	XX.X
	SD	XX.X	XX.X	XX.X
	Min	XX	XX	XX
	Median	XX.X	XX.X	XX.X
	Max	XX	XX	XX
...	...	...	...	...

[1] Defined as (Cumulative actual dose/cumulative dose if initially assigned dose had been given to all scheduled dosing visits)* 100%.
[2] Number of completed cycles by the subject over the entire study period.
Study 8951-CL-0302 Table 12.2.1.4.1 (continue) Source: <Listing / Dataset>

Study Drug Treatment – Capecitabine
Safety Analysis Set

Characteristic		Arm A (N=xx)	Arm B (N=xx)	Overall (N=xx)
Average Amount of Dose per Planned Dosing Day	n	XX	XX	XX
	Mean	XX.X	XX.X	XX.X
	SD	XX.X	XX.X	XX.X
	Min	XX	XX	XX
	Median	XX.X	XX.X	XX.X
	Max	XX	XX	XX
Duration of Treatment Cumulative Category (days)	>=1	XX (XX.X%)	XX (XX.X%)	XX (XX.X%)
	>=43	XX (XX.X%)	XX (XX.X%)	XX (XX.X%)
	>=85	XX (XX.X%)	XX (XX.X%)	XX (XX.X%)
	>=169	XX (XX.X%)	XX (XX.X%)	XX (XX.X%)
	>=253	XX (XX.X%)	XX (XX.X%)	XX (XX.X%)
	>=337	XX (XX.X%)	XX (XX.X%)	XX (XX.X%)
	>=505	XX (XX.X%)	XX (XX.X%)	XX (XX.X%)

[1] Defined as (Cumulative actual dose/cumulative dose if initially assigned dose had been given to all scheduled dosing visits)* 100%.
[2] Number of oral dosing per subject over the entire study period.

Study 8951-CL-0302 Table 12.2.2.2 Source: <Listing / Dataset>
Concomitant Medications
Safety Analysis Set

Therapeutic Subgroup (ATC 2nd level)<br>Chemical Subgroup (ATC 4th level)<br>Preferred WHO Name	Arm A<br>(N=xx)	Arm B<br>(N=xx)
Overall	XX (XX.X%)	XX (XX.X%)
Therapeutic Subgroup 1	XX (XX.X%)	XX (XX.X%)
Chemical Subgroup 1	XX (XX.X%)	XX (XX.X%)
Preferred WHO Name A	XX (XX.X%)	XX (XX.X%)
Preferred WHO Name B	XX (XX.X%)	XX (XX.X%)
Chemical Subgroup 2	XX (XX.X%)	XX (XX.X%)
Preferred WHO Name C	XX (XX.X%)	XX (XX.X%)
Preferred WHO Name D	XX (XX.X%)	XX (XX.X%)
Therapeutic Subgroup 2	XX (XX.X%)	XX (XX.X%)
...	...	...
...	...	...

Number of subjects and percentage of subjects (%) are shown.

Sorting order: alphabetical order by Therapeutic Subgroup and Chemical Subgroup and Preferred WHO Name .

Medications used on the date of first dose of study drug or later are shown.

Medications that started prior to first administration of study drug and continued while study drug was given will be counted as both previous and concomitant medications.

A medication which can be classified into several chemical and/or therapeutic subgroups is presented in all chemical and therapeutic subgroups.

Study 8951-CL-0302 Table 12.6.1.1 Source: <Listing / Dataset>
Overview of Treatment-Emergent Adverse Events and Death
Safety Analysis Set

	Arm A (N=xx) N (%)	Arm B (N=xx) n (%)	Overall (N=xx) N (%)
TEAE	XX (XX.X%)	XX (XX.X%)	XX (XX.X%)
Drug-Related [1] TEAE	XX (XX.X%)	XX (XX.X%)	XX (XX.X%)
Zolbetuximab-Related Treatment-Emergent Adverse Events (MedDRA V20.1)	XX (XX.X%)	XX (XX.X%)	XX (XX.X%)
Oxaliplatin-Related Treatment-Emergent Adverse Events (MedDRA V20.1)	XX (XX.X%)	XX (XX.X%)	XX (XX.X%)
Capecitabine-Related Treatment-Emergent Adverse Events (MedDRA V20.1)	XX (XX.X%)	XX (XX.X%)	XX (XX.X%)
Serious TEAE [2]	XX (XX.X%)	XX (XX.X%)	XX (XX.X%)
Drug-Related [1] Serious TEAE [2]	XX (XX.X%)	XX (XX.X%)	XX (XX.X%)
TEAE Leading to Permanent Discontinuation of Study Drug [3]	XX (XX.X%)	XX (XX.X%)	XX (XX.X%)
TEAE Leading to Permanent Discontinuation of Zolbetuximab/Placebo	XX (XX.X%)	XX (XX.X%)	XX (XX.X%)
TEAE Leading to Permanent Discontinuation of Oxaliplatin	XX (XX.X%)	XX (XX.X%)	XX (XX.X%)
TEAE Leading to Permanent Discontinuation of Capecitabine	XX (XX.X%)	XX (XX.X%)	XX (XX.X%)
Drug-Related [1] TEAE Leading to Permanent Discontinuation of Study Drug [3]	XX (XX.X%)	XX (XX.X%)	XX (XX.X%)
Drug-Related [1] TEAE Leading to Permanent Discontinuation of Zolbetuximab/Placebo	XX (XX.X%)	XX (XX.X%)	XX (XX.X%)
Drug-Related [1] TEAE Leading to Permanent Discontinuation of Oxaliplatin	XX (XX.X%)	XX (XX.X%)	XX (XX.X%)
Drug-Related [1] TEAE Leading to Permanent Discontinuation of Capecitabine	XX (XX.X%)	XX (XX.X%)	XX (XX.X%)
Late Adverse Event [4]	XX (XX.X%)	XX (XX.X%)	XX (XX.X%)
Worst CTCAE Grade TEAE			
>=3	XX (XX.X%)	XX (XX.X%)	XX (XX.X%)
3	XX (XX.X%)	XX (XX.X%)	XX (XX.X%)
4	XX (XX.X%)	XX (XX.X%)	XX (XX.X%)
5	XX (XX.X%)	XX (XX.X%)	XX (XX.X%)
Drug-Related [1] Worst CTCAE Grade TEAE			
>=3	XX (XX.X%)	XX (XX.X%)	XX (XX.X%)
3	XX (XX.X%)	XX (XX.X%)	XX (XX.X%)
4	XX (XX.X%)	XX (XX.X%)	XX (XX.X%)
5	XX (XX.X%)	XX (XX.X%)	XX (XX.X%)
Death up to 30 Days After the Last Dose	XX (XX.X%)	XX (XX.X%)	XX (XX.X%)
TEAE Leading to Death	XX (XX.X%)	XX (XX.X%)	XX (XX.X%)
Drug-Related [1] TEAE Leading to Death	XX (XX.X%)	XX (XX.X%)	XX (XX.X%)
Death [5]	XX (XX.X%)	XX (XX.X%)	XX (XX.X%)

Footnotes appear on next page

Number of subjects (n) and percentage of subjects (%) are shown.

TEAE: Treatment Emergent Adverse Event.

[1] Possible or probable, as assessed by the investigator, or records where relationship is missing.

[2] Includes SAEs upgraded by the sponsor based on review of the Sponsor's list of Always Serious terms, if any upgrade was done.

[3] Zolbetuximab and any component of CAPOX.

[4] Adverse Event occurred beyond 30 days from the last study treatment.

[5] All reported deaths after the first study drug administration up to analysis cutoff date.

Study 8951-CL-0302 Table 12.6.1.2 Source: <Listing / Dataset>
Treatment-Emergent Adverse Events (MedDRA V20.1)
Safety Analysis Set

System Organ Class<br>Preferred Term	Arm A<br>(N=xx)<br>n (%)	Arm B<br>(N=xx)<br>n (%)	Overall<br>(N=xx)<br>n (%)
Overall	XX (XX.X%)	XX (XX.X%)	XX (XX.X%)
System Organ Class 1	XX (XX.X%)	XX (XX.X%)	XX (XX.X%)
Preferred Term A	XX (XX.X%)	XX (XX.X%)	XX (XX.X%)
Preferred Term B	XX (XX.X%)	XX (XX.X%)	XX (XX.X%)
System Organ Class 2	XX (XX.X%)	XX (XX.X%)	XX (XX.X%)
Preferred Term C	XX (XX.X%)	XX (XX.X%)	XX (XX.X%)
...	...	...	...

Number of subjects (n) and percentage of subjects (%) are shown.
Sorting order: alphabetical by system organ class and decreasing order of preferred term. In case of ties, alphabetical order should be used.

This shell will be used for the following tables:

```
Program: </directory/directory/directory/program.sas>                <Draft/Final Version>
Study 8951-CL-0302                  Table 12.6.1.7.1                 Source: <Listing / Dataset>
            AEs Collected After 30 Days Post Last Dose of Study Drug (MedDRA V20.1)
                                  Safety Analysis Set
```

Study 8951-CL-0302 Table 12.6.1.6 Source: <Listing / Dataset>
Serious Treatment-Emergent Adverse Events (MedDRA V20.1)
Safety Analysis Set

System Organ Class Preferred Term	Arm A<br>(N=XX)<br>n (%) #E	Arm B<br>(N=XX)<br>n (%) #E	Overall<br>(N=XX)<br>n (%) #E
Overall	XX (XX.X%) E	XX (XX.X%) E	XX (XX.X%) E
System Organ Class 1	XX (XX.X%) E	XX (XX.X%) E	XX (XX.X%) E
Preferred Term A	XX (XX.X%) E	XX (XX.X%) E	XX (XX.X%) E
Preferred Term B	XX (XX.X%) E	XX (XX.X%) E	XX (XX.X%) E
System Organ Class 2	XX (XX.X%) E	XX (XX.X%) E	XX (XX.X%) E
Preferred Term C	XX (XX.X%) E	XX (XX.X%) E	XX (XX.X%) E
...	...	...	...

Number of subjects (n), percentage of subjects (%), and number of events (#E) are shown.
Sorting order: alphabetical order by System Organ Class and descending by the number of subjects of Overall group by Preferred Term. In case of ties, alphabetical order by Preferred Term is applied.

This shell will be used for the following tables:

```
Program: </directory/directory/directory/program.sas>                          <Draft/Final Version>
Study 8951-CL-0302                        Table 12.6.1.8                  Source: <Listing / Dataset>
              Drug-Related Serious Treatment-Emergent Adverse Events (MedDRA V20.1)
                                     Safety Analysis Set

Program: </directory/directory/directory/program.sas>                          <Draft/Final Version>
Study 8951-CL-0302                        Table 12.6.1.7.2                Source: <Listing / Dataset>
          Serious AEs Collected After 30 Days Post Last Dose of Study Drug (MedDRA V20.1)
                                     Safety Analysis Set
```

Study 8951-CL-0302 Table 12.6.1.18 Source: <Listing / Dataset>

Treatment-Emergent Adverse Events (MedDRA V20.1) by Preferred Term
Safety Analysis Set

Preferred Term	Arm A (N=XX) n (%)	Arm B (N=XX) n (%)	Overall (N=XX) n (%)
Overall	XX (XX.X%)	XX (XX.X%)	XX (XX.X%)
Preferred Term A	XX (XX.X%)	XX (XX.X%)	XX (XX.X%)
Preferred Term B	XX (XX.X%)	XX (XX.X%)	XX (XX.X%)
Preferred Term C	XX (XX.X%)	XX (XX.X%)	XX (XX.X%)
...	...	...	...

Number of subjects and percentage of subjects (%) are shown.
Sorting order: descending by the number of subjects of Overall group by Preferred Term. In case of ties, alphabetical order by Preferred Term is applied.

Study 8951-CL-0302 Table 12.6.1.19 Source: <Listing / Dataset>

Treatment-Emergent Adverse Events Leading to Death (MedDRA V20.1) by Preferred Term
Safety Analysis Set

Preferred Term	Arm A (N=XX) n (%) #E	Arm B (N=XX) n (%) #E	Overall (N=XX) n (%) #E
Overall	XX (XX.X%) E	XX (XX.X%) E	XX (XX.X%) E
Preferred Term A	XX (XX.X%) E	XX (XX.X%) E	XX (XX.X%) E
Preferred Term B	XX (XX.X%) E	XX (XX.X%) E	XX (XX.X%) E
Preferred Term C	XX (XX.X%) E	XX (XX.X%) E	XX (XX.X%) E
...	...	...	...

Number of subjects and percentage of subjects (%) and number of events (#E) are shown.
Sorting order: descending by the number of subjects of Overall group by Preferred Term. In case of ties, alphabetical order by Preferred Term Code is applied.

Study 8951-CL-0302 Table 12.6.1.14 Source: <Listing / Dataset>
Treatment-Emergent Adverse Events with NCI-CTCAE >=3 (MedDRA V20.1)
Safety Analysis Set

System Organ Class<br>Preferred Term	Maximum CTCAE Grade [1]	Arm A (N=XX) n (%) #E	Arm B (N=XX) n (%) #E	Overall (N=XX) n (%) #E
Overall	3	X (XX.X%) E	X (XX.X%) E	X (XX.X%) E
	4	X (XX.X%) E	X (XX.X%) E	X (XX.X%) E
	5	X (XX.X%) E	X (XX.X%) E	X (XX.X%) E
	Missing	XX	XX	XX
	Total	XX	XX	XX
		X (XX.X%) E	X (XX.X%) E	X (XX.X%) E
System Organ Class 1	3	X (XX.X%) E	X (XX.X%) E	X (XX.X%) E
	4	X (XX.X%) E	X (XX.X%) E	X (XX.X%) E
	5	X (XX.X%) E	X (XX.X%) E	X (XX.X%) E
	Missing	XX	XX	XX
	Total	XX	XX	XX
		X (XX.X%) E	X (XX.X%) E	X (XX.X%) E
Preferred Term A	3	X (XX.X%) E	X (XX.X%) E	X (XX.X%) E
	4	X (XX.X%) E	X (XX.X%) E	X (XX.X%) E
	5	X (XX.X%) E	X (XX.X%) E	X (XX.X%) E
	Missing	XX	XX	XX
	Total	XX	XX	XX
...	...	...	...	...

Number of subjects (n), percentage of subjects (%) are shown.
Sorting order: alphabetical order by System Organ Class and descending by the number of subjects of Overall group by Preferred Term. In case of ties, alphabetical order by Preferred Term is applied.
[1] Subject counted once under maximum severity. #E includes all events.

Study 8951-CL-0302 Table 12.6.1.15 Source: <Listing / Dataset>
Drug-related Treatment-Emergent Adverse Events with NCI-CTCAE >=3 (MedDRA V20.1)
Safety Analysis Set

System Organ Class<br>Preferred Term	Maximum<br>CTCAE Grade<br>[1]	Arm A<br>(N=XX)<br>n (%) #E	Arm B<br>(N=XX)<br>n (%) #E	Overall<br>(N=XX)<br>n (%) #E
Overall	3	X (XX.X%) E	X (XX.X%) E	X (XX.X%) E
	4	X (XX.X%) E	X (XX.X%) E	X (XX.X%) E
	5	X (XX.X%) E	X (XX.X%) E	X (XX.X%) E
	Missing	XX	XX	XX
	Total	XX	XX	XX
		X (XX.X%) E	X (XX.X%) E	X (XX.X%) E
System Organ Class 1	3	X (XX.X%) E	X (XX.X%) E	X (XX.X%) E
	4	X (XX.X%) E	X (XX.X%) E	X (XX.X%) E
	5	X (XX.X%) E	X (XX.X%) E	X (XX.X%) E
	Missing	XX	XX	XX
	Total	XX	XX	XX
		X (XX.X%) E	X (XX.X%) E	X (XX.X%) E
Preferred Term A	3	X (XX.X%) E	X (XX.X%) E	X (XX.X%) E
	4	X (XX.X%) E	X (XX.X%) E	X (XX.X%) E
	5	X (XX.X%) E	X (XX.X%) E	X (XX.X%) E
	Missing	XX	XX	XX
	Total	XX	XX	XX
...	...	...	...	...

Number of subjects (n), percentage of subjects (%) are shown.
Sorting order: alphabetical order by System Organ Class and descending by the number of subjects of Overall group by Preferred Term. In case of ties, alphabetical order by Preferred Term is applied.
[1] Subject counted once under maximum severity. #E includes all events.

Study 8951-CL-0302 Table 12.6.2.2.2.1 Source: <Listing / Dataset>

Shift Table for Laboratory Test Results From Baseline to Worst Post-Baseline NCI CTCAE Grade (V4.03), Hematology
Based on Central Assessment
Safety Analysis Set

--- Laboratory test = XXX (unit) for 'Low' ---

		---------- Baseline Grade							
Treatment Group	Worst Post-Baseline Grade	Grade 0 [1]	Grade 1	Grade 2	Grade 3	Grade 4	Grade 5	Total	No Data
Arm A (N=XX)	Grade 0 [1]	XX (XX.X%)	XX (XX.X%)	XX (XX.X%)	XX (XX.X%)	XX (XX.X%)	XX (XX.X%)	XX	XX
	Grade 1	XX (XX.X%)	XX (XX.X%)	XX (XX.X%)	XX (XX.X%)	XX (XX.X%)	XX (XX.X%)	XX	XX
	Grade 2	XX (XX.X%)	XX (XX.X%)	XX (XX.X%)	XX (XX.X%)	XX (XX.X%)	XX (XX.X%)	XX	XX
	Grade 3	XX (XX.X%)	XX (XX.X%)	XX (XX.X%)	XX (XX.X%)	XX (XX.X%)	XX (XX.X%)	XX	XX
	Grade 4	XX (XX.X%)	XX (XX.X%)	XX (XX.X%)	XX (XX.X%)	XX (XX.X%)	XX (XX.X%)	XX	XX
	Grade 5	XX (XX.X%)	XX (XX.X%)	XX (XX.X%)	XX (XX.X%)	XX (XX.X%)	XX (XX.X%)	XX	XX
	Total	XX	XX	XX	XX	XX	XX	XX	XX
	No Data	XX	XX	XX	XX	XX	XX	XX	XX
Arm B (N=XX)	Grade 0 [1]	XX (XX.X%)	XX (XX.X%)	XX (XX.X%)	XX (XX.X%)	XX (XX.X%)	XX (XX.X%)	XX	XX
	Grade 1	XX (XX.X%)	XX (XX.X%)	XX (XX.X%)	XX (XX.X%)	XX (XX.X%)	XX (XX.X%)	XX	XX
	Grade 2	XX (XX.X%)	XX (XX.X%)	XX (XX.X%)	XX (XX.X%)	XX (XX.X%)	XX (XX.X%)	XX	XX
	Grade 3	XX (XX.X%)	XX (XX.X%)	XX (XX.X%)	XX (XX.X%)	XX (XX.X%)	XX (XX.X%)	XX	XX
	Grade 4	XX (XX.X%)	XX (XX.X%)	XX (XX.X%)	XX (XX.X%)	XX (XX.X%)	XX (XX.X%)	XX	XX
	Grade 5	XX (XX.X%)	XX (XX.X%)	XX (XX.X%)	XX (XX.X%)	XX (XX.X%)	XX (XX.X%)	XX	XX
	Total	XX	XX	XX	XX	XX	XX	XX	XX
	No Data	XX	XX	XX	XX	XX	XX	XX	XX
Overall (N=XX)	Grade 0 [1]	XX (XX.X%)	XX (XX.X%)	XX (XX.X%)	XX (XX.X%)	XX (XX.X%)	XX (XX.X%)	XX	XX
	Grade 1	XX (XX.X%)	XX (XX.X%)	XX (XX.X%)	XX (XX.X%)	XX (XX.X%)	XX (XX.X%)	XX	XX
	Grade 2	XX (XX.X%)	XX (XX.X%)	XX (XX.X%)	XX (XX.X%)	XX (XX.X%)	XX (XX.X%)	XX	XX
	Grade 3	XX (XX.X%)	XX (XX.X%)	XX (XX.X%)	XX (XX.X%)	XX (XX.X%)	XX (XX.X%)	XX	XX
	Grade 4	XX (XX.X%)	XX (XX.X%)	XX (XX.X%)	XX (XX.X%)	XX (XX.X%)	XX (XX.X%)	XX	XX
	Grade 5	XX (XX.X%)	XX (XX.X%)	XX (XX.X%)	XX (XX.X%)	XX (XX.X%)	XX (XX.X%)	XX	XX
	Total	XX	XX	XX	XX	XX	XX	XX	XX
	No Data	XX	XX	XX	XX	XX	XX	XX	XX

Note: Percents in the table are based on column total (number of subjects who had particular grade at baseline).
Baseline NCI-CTCAE grade is the last non-missing grade before first dose.
[1] 'Grade 0' indicates lab test results that are outside CTC criteria.

This is a table shell TLB_003
Programming Note: for some lab values there are both 'Low' (i.e. Hypo) and 'High' (i.e. Hyper) results. Please specify this on the first subtitle (*Laboratory test = XXX (unit) for 'Low'*).

Program: </directory/directory/directory/program.sas> <Draft/Final Version>

Study 8951-CL-0302 Table 12.6.2.2.2.2 Source: <Listing / Dataset>

Shift Table for Laboratory Test Results From Baseline to Worst Post-Baseline NCI CTCAE Grade (V4.03), Biochemistry

Based on Central Assessment

Safety Analysis Set

Program: </directory/directory/directory/program.sas> <Draft/Final Version>

Study 8951-CL-0302 Table 12.6.2.2.2.3 Source: <Listing / Dataset>

Shift Table for Laboratory Test Results From Baseline to Worst Post-Baseline NCI CTCAE Grade (V4.03), Coagulation

Based on Central Assessment

Safety Analysis Set

Program: </directory/directory/directory/program.sas> <Draft/Final Version>

Study 8951-CL-0302 Table 12.6.2.2.2.4 Source: <Listing / Dataset>

Shift Table for Laboratory Test Results From Baseline to Worst Post-Baseline NCI CTCAE Grade (V4.03), Urinalysis

Based on Central Assessment

Safety Analysis Set

Study 8951-CL-0302 Table 12.6.2.3 Source: <Listing / Dataset>
Potentially Clinically Significant Values in Liver Enzymes and Total Bilirubin
Safety Analysis Set

Parameter [1]	Criteria	Arm A (N=XX)	Arm B (N=XX)
ALT	> 3xULN	XX/XX (XX.X%)	XX/XX (XX.X%)
	> 5xULN	XX/XX (XX.X%)	XX/XX (XX.X%)
	> 10xULN	XX/XX (XX.X%)	XX/XX (XX.X%)
	> 20xULN	XX/XX (XX.X%)	XX/XX (XX.X%)
AST	> 3xULN	XX/XX (XX.X%)	XX/XX (XX.X%)
	> 5xULN	XX/XX (XX.X%)	XX/XX (XX.X%)
	> 10xULN	XX/XX (XX.X%)	XX/XX (XX.X%)
	> 20xULN	XX/XX (XX.X%)	XX/XX (XX.X%)
ALT or AST	> 3xULN	XX/XX (XX.X%)	XX/XX (XX.X%)
	> 5xULN	XX/XX (XX.X%)	XX/XX (XX.X%)
	> 10xULN	XX/XX (XX.X%)	XX/XX (XX.X%)
	> 20xULN	XX/XX (XX.X%)	XX/XX (XX.X%)
Total Bilirubin	> 2xULN	XX/XX (XX.X%)	XX/XX (XX.X%)
ALP	> 1.5xULN	XX/XX (XX.X%)	XX/XX (XX.X%)
ALT and/or AST AND Total Bilirubin [1]	(ALT and/or AST > 3xULN) AND Total Bilirubin > 2xULN	XX/XX (XX.X%)	XX/XX (XX.X%)
ALT and/or AST AND Total Bilirubin AND ALP [1]	(ALT and/or AST > 3xULN) AND Total Bilirubin > 2xULN and ALP < 2xULN	XX/XX (XX.X%)	XX/XX (XX.X%)

Note: Percentages were calculated based on the total number of subjects with non-missing values at each visit.
[1] For each subject, the worst value among all post-baseline measurements at the visit or up to 1 day apart was used.
ULN = upper limit of normal range

Study 8951-CL-0302 Table 12.6.5.2.2 Source: <Listing / Dataset>
Shift Table of ECOG Performance Status
Full Analysis Set

		---------------------------- Baseline Grade ----------------------------							
Treatment Group	Worst Post-Baseline Grade	Grade 0	Grade 1	Grade 2	Grade 3	Grade 4	Grade 5	Total	No Data
Arm A (N=XX)	Grade 0	XX (XX.X%)	XX (XX.X%)	XX (XX.X%)	XX (XX.X%)	XX (XX.X%)	XX (XX.X%)	XX	XX
	Grade 1	XX (XX.X%)	XX (XX.X%)	XX (XX.X%)	XX (XX.X%)	XX (XX.X%)	XX (XX.X%)	XX	XX
	Grade 2	XX (XX.X%)	XX (XX.X%)	XX (XX.X%)	XX (XX.X%)	XX (XX.X%)	XX (XX.X%)	XX	XX
	Grade 3	XX (XX.X%)	XX (XX.X%)	XX (XX.X%)	XX (XX.X%)	XX (XX.X%)	XX (XX.X%)	XX	XX
	Grade 4	XX (XX.X%)	XX (XX.X%)	XX (XX.X%)	XX (XX.X%)	XX (XX.X%)	XX (XX.X%)	XX	XX
	Grade 5	XX (XX.X%)	XX (XX.X%)	XX (XX.X%)	XX (XX.X%)	XX (XX.X%)	XX (XX.X%)	XX	XX
	Total	XX	XX	XX	XX	XX	XX	XX	XX
	No Data	XX	XX	XX	XX	XX	XX	XX	XX
Arm B (N=XX)	Grade 0	XX (XX.X%)	XX (XX.X%)	XX (XX.X%)	XX (XX.X%)	XX (XX.X%)	XX (XX.X%)	XX	XX
	Grade 1	XX (XX.X%)	XX (XX.X%)	XX (XX.X%)	XX (XX.X%)	XX (XX.X%)	XX (XX.X%)	XX	XX
	Grade 2	XX (XX.X%)	XX (XX.X%)	XX (XX.X%)	XX (XX.X%)	XX (XX.X%)	XX (XX.X%)	XX	XX
	Grade 3	XX (XX.X%)	XX (XX.X%)	XX (XX.X%)	XX (XX.X%)	XX (XX.X%)	XX (XX.X%)	XX	XX
	Grade 4	XX (XX.X%)	XX (XX.X%)	XX (XX.X%)	XX (XX.X%)	XX (XX.X%)	XX (XX.X%)	XX	XX
	Grade 5	XX (XX.X%)	XX (XX.X%)	XX (XX.X%)	XX (XX.X%)	XX (XX.X%)	XX (XX.X%)	XX	XX
	Total	XX	XX	XX	XX	XX	XX	XX	XX
	No Data	XX	XX	XX	XX	XX	XX	XX	XX

Percentages in the table were based on column total (number of subjects who had particular severity at baseline).

Study 8951-CL-0302 Appendix 13.2.7.1 Source: <Listing / Dataset>
Adverse Events (MedDRA V20.1)
All Randomized Subjects

Subject ID	Actual Treatment Group	Age(years)/ Sex/ Race[1]/ Weight(kg)	Last Dose Day/Date	System Organ Class/ Preferred term/ Reported term	Onset Day/ Onset Date/ End Day/ End Date/ Onset Timing [2]/ TEAE	Serious (Reason) [3]/ Course of Event/ NCI CTC Grade	Relationship/ Outcome/ Treatment Required	Action taken for: Zolbetuximab/ Oxaliplatin/ Capecitabine
Investigator site = xxxxxx								
xxxxxx	xxxx	43/F/W/45	xxxx/YYYY-MM-DD	SOC/ xxxx/ Xxxx	xxxx/YYYY-MM-DD/ xxxx/YYYY-MM-DD/ Before/Yes	xxxx/ xxxx/ xxxx	Zolbetuximab: YES/ RECOVERED/ YES	Zolbetuximab: Interrupted/ Oxaliplatin: Unknown/ Capecitabine: Unknown
				SOC/ xxxx/ Xxxx	xxxx/YYYY-MM-DD/ xxxx/YYYY-MM-DD/ After/No	xxxx/ xxxx/ xxxx	xxxx/ xxxx/ Xxxx	
xxxxx	xxxx	43/F/W/45	xxxx/YYYY-MM-DD	Psychiatric disorders/ Nervousness / FEELING SHAKY	3/2007-09-03/ 9 E/2007-09	Yes (RPH, D)/ Single Episode/ Grade 1	NOT RELATED/ COMPLETELY RECOVERED/ NO	Xxxxx: xxxxxxxxxxxxxx/ Xxxxx: xxxxxxxxxxxxxx/ Xxxxx: xxxxxxxxxxxxxx/

E: estimated value.
[1] F=Female, M=Male; Race:W=White, B=Black/African American, P=Native Hawaiian or other Pacific islander, A=Asian, I=American Indian or Alaska native, O=Other; Weight in kilograms.
[2] 'Before' for 'Onset before first dose of study drug', 'After' for 'Onset after first dose of study drug'.
[3] AEs either were identified as serious by the Investigator, or upgraded by the sponsor based on review of the Sponsor's list of Always Serious terms. Reason for seriousness: D = Death, RPH = Requires or prolongs hospitalization, CA = Congenital anomaly, LT = Life-threatening, PSDI = Persistent or significant disability/incapacity, OMI = Other medical importance, AST = upgraded according to Sponsor's list of Always Serious terms

Programming Note: Treatment, Site, subject ID , Onset day, End day, alphabetically by Preferred term.

Generate for the following:

Program: </directory/directory/directory/program.sas> <Draft/Final Version>
Study 8951-CL-0302 Appendix 13.2.7.3 Source: <Listing / Dataset>
Adverse Events (MedDRA V20.1) Leading to Death
All Randomized Subjects

Program: </directory/directory/directory/program.sas> <Draft/Final Version>
Study 8951-CL-0302 Appendix 13.2.7.4 Source: <Listing / Dataset>
Serious Adverse Events (MedDRA V20.1)
All Randomized Subjects

Study 8951-CL-0302 Appendix 13.2.8.1.1 Source: <Listing / Dataset>
Central Laboratory Tests Results in SI Units - Hematology, Part x of y
All Randomized Subjects

Subject ID	Treatment	Age(years)/ Sex/ Race[1]/ Weight(kg)	Last Dose Day	Visit Day/Date	Analysis Visit	Visit Name	Test 1 (Units)	Test 2 (Units)	Test 3 (Units)	Test 4 (Units)	...
Investigator Site = xxxxxx											
xxxxxx	xxxx	43/F/W/45	xxxx	Xxx/YYYY-MM-DD	xxx	xxx	xxx	xxxH	xxx	xxx	...
				Xxx/YYYY-MM-DD	xxx	xxx	xxxL	xxx	xxx	xxx	
				Xxx/YYYY-MM-DD	xxx	xxx	xxx	xxx	xxx	xxxH G4	
				...	...	...	...	...	...	...	
xxxxxx	xxxx	xxxx/	xxxx	Xxx/YYYY-MM-DD	xxx	xxx	xxx	XxxH G3	xxx	xxx	...
		xxxx/		Xxx/YYYY-MM-DD	xxx	xxx	xxxL	xxx	xxx	xxx	
		xxxx/		Xxx/YYYY-MM-DD	xxx	xxx	xxx	xxx	xxx	xxxH	
		xxxx		...	...	...	...	...	...	...	

H: above central laboratory reference range, L: below central laboratory reference range. Refer to the listing in section 13.1.10 for the reference ranges for the central laboratory data.
[1] F=Female, M=Male; Race:W=White, B=Black/African American, P=Native Hawaiian or other Pacific islander, A=Asian, I=American Indian or Alaska native, O=Other; Weight in kilograms.

Programming Note:
- Sort by Treatment, Site, subject ID , Visit day.
- Listing 13.2.8.1.1 can be expended into 13.2.8.1.1.1 – 13.2.8.1.1.x depends number of lab tests collected. Please make sure tests of the same category are displayed in one listing.
- Specify the grade as 'Gn' (where n=0, 1, 2, 3, 4, 5) next to the lab result with one space in-between, e.g. 'xxx G0', 'xxxH G3'.

This shell will be used for:

Program: </directory/directory/directory/program.sas> <Draft/Final Version>
Study 8951-CL-0302 Appendix 13.2.8.1.2 Source: <Listing / Dataset>
Central Laboratory Tests Results in SI Units - Chemistry, Part x of y
All Randomized Subjects

Programming note: Listing 13.2.8.1.2 can be expended into 13.2.8.1.2.1 – 13.2.8.1.2.x depends on number of lab tests collected. Please make sure tests of the same category are displayed in one listing.
Program: </directory/directory/directory/program.sas> <Draft/Final Version>

Study 8951-CL-0302 Appendix 13.2.8.1.3 Source: <Listing / Dataset>
Central Laboratory Tests Results in SI Units - Urinalysis, Part x of y
All Randomized Subjects

Programming note: Listing 13.2.8.1.3 can be expended into 13.2.8.1.3.1 – 13.2.8.1.3.x depends on number of lab tests collected. Please make sure tests of the same category are displayed in one listing.

Program: </directory/directory/directory/program.sas> <Draft/Final Version>

Study 8951-CL-0302 Appendix 13.2.8.1.4 Source: <Listing / Dataset>
Central Laboratory Tests Results in SI Units - Coagulation, Part x of y
All Randomized Subjects

Programming note: Listing 13.2.8.1.4 can be expended into 13.2.8.1.4.1 – 13.2.8.1.4.x depends on number of lab tests collected. Please make sure tests of the same category are displayed in one listing.

Study 8951-CL-0302

Appendix 13.2.6.16
ECOG Performance Status
All Randomized Subject

Source: <Listing / Dataset>

Subject ID	Treatment	Age/ Sex/ Race [1]/ Weight	Last Dose Day	Analysis Visit	Assessment Day/Date	Subject ECOG Status	Chg From Baseline
Investigator site = xxxxx							
Xxxxxx	xxxx	43/F/W/45	xxxx	xxxxxxxxxxxxx	XX/YYYY-MM-DD	1 - xxxxxxx	
				xxxxxxxxxxxxx	XX/YYYY-MM-DD	X - xxxxxxx	X
...	...	...	...				

[1] F=Female, M=Male; Race:W=White, B=Black/African American, P=Native Hawaiian or other Pacific islander, A=Asian, I=American Indian or Alaska native, O=Other; Weight in kilograms..

Programming Note:
Sort by Treatment, Site, subject ID, assessment date.

8.2 TABLES, LISTINGS AND FIGURES (ONLY UPON REQUEST BY IDMC)

Study 8951-CL-0302 | Figure 12.3.1.1 | Source: <Listing / Dataset>

Kaplan-Meier Plot of Progression-Free Survival, Independent Radiologic Review (NO p-value or CI for HR will be provided)
Full Analysis Set

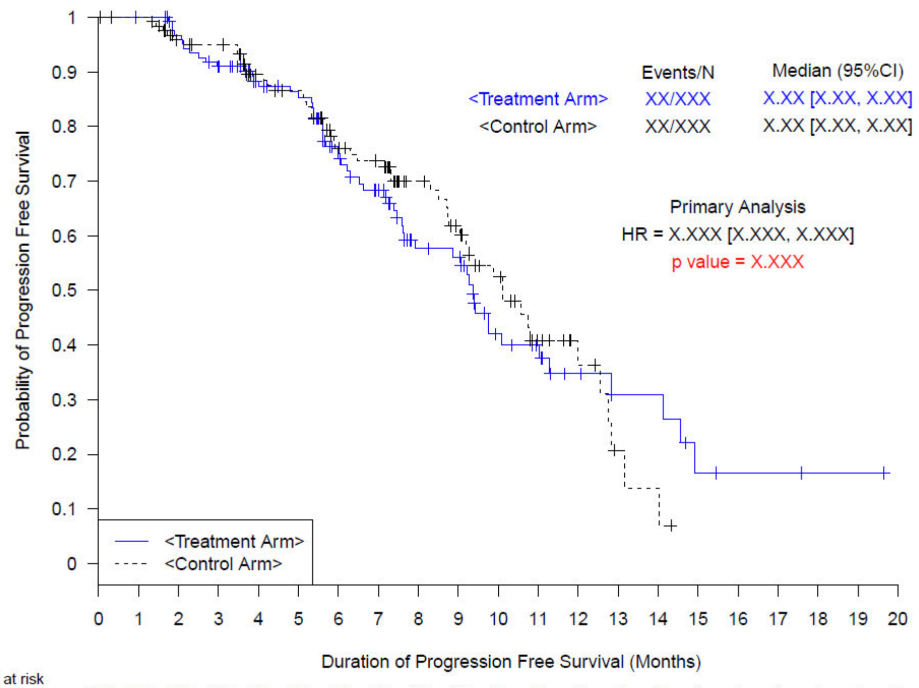

N at risk	0	1	2	3	4	5	6	7	8	9	10	11	12	13	14	15	16	17	18	19	20
<Treatment Arm>	133	125	118	109	94	87	67	56	38	36	21	17	10	7	7	3	2	2	1	1	0
<Control Arm>	132	124	110	107	91	86	68	62	44	36	25	14	8	3	2	0	0	0	0	0	0

Study 8951-CL-0302 | Figure 12.3.1.2 | Source: <Listing / Dataset>

Kaplan-Meier Plot of Progression-Free Survival, Investigator Assessment (NO p-value or CI for HR will be provided)
Full Analysis Set

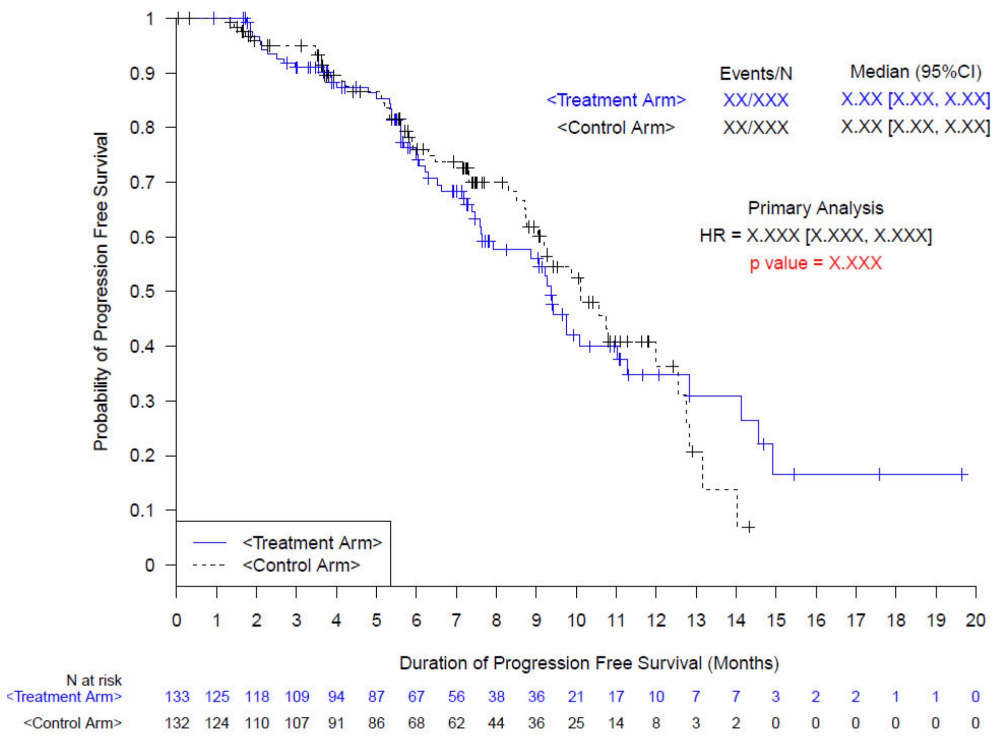

Study 8951-CL-0302

Figure 12.3.2

Source: <Listing / Dataset>

Kaplan-Meier Plot of Overall Survival (NO p-value or CI for HR will be provided)
Full Analysis Set

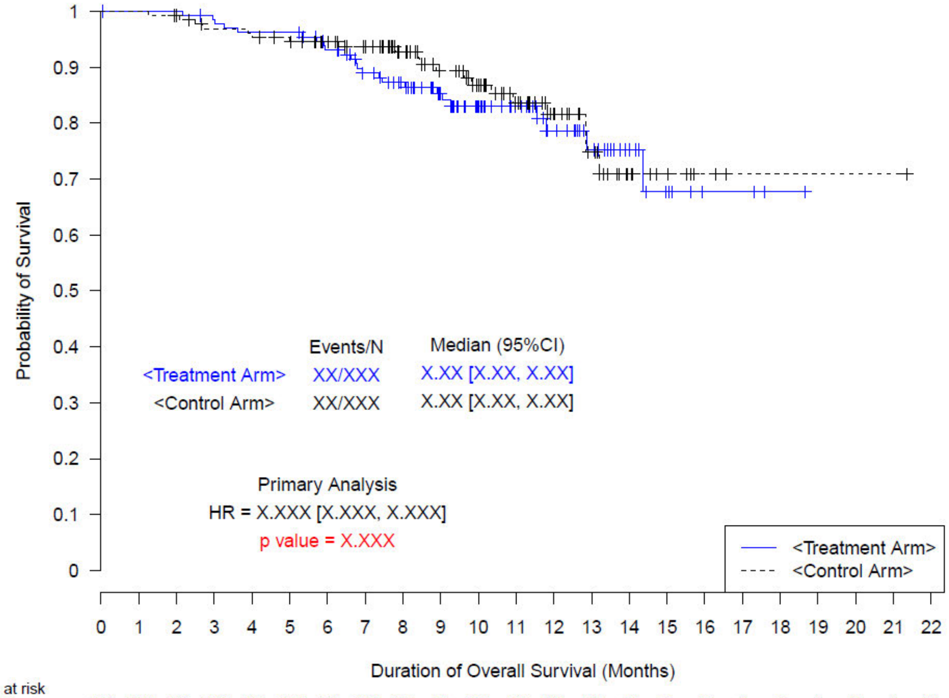

N at risk																						
<Treatment Arm>	133	132	132	129	126	126	118	107	95	78	59	47	32	22	12	7	3	3	1	0	0	0	0
<Control Arm>	132	132	130	124	123	119	113	105	92	76	63	51	36	21	11	7	3	1	1	1	1	1	0

8.3 Author and Approver Signatories

E-signatures are attached at end of document (next page). Wet signatures, if any, are provided on this page.

Author: ______________________ Date: ______________

PPD

Approved by: ______________________ Date: ______________

PPD

Approved by: ______________________ Date: ______________

PPD

INTERIM ANALYSIS PLAN

Version 2.0, 04-Nov-2021

A Phase 3, Global, Multi-Center, Double-Blind, Randomized, Efficacy Study of Zolbetuximab (IMAB362) Plus CAPOX Compared with Placebo Plus CAPOX as First-line Treatment of Subjects with Claudin (CLDN)18.2-Positive, HER2-Negative, Locally Advanced Unresectable or Metastatic Gastric or Gastroesophageal Junction (GEJ) Adenocarcinoma

ISN/Protocol 8951-CL-0302

IND 129598

EudraCT 2018-000519-26

Sponsor:

Astellas Pharma Global Development, Inc. (APGD)
1 Astellas Way
Northbrook, IL 60062

This document contains confidential information which is the intellectual property of Astellas. By accepting or reviewing this document, you agree to hold this information in confidence and not copy or disclose it to others or use it for unauthorized purposes except (1) as otherwise agreed to in writing; (2) where required by applicable law; (3) where disclosure is directly related to the care and safety of the research participant; and (4) where disclosure of such information is made to a member of the investigator's team who agrees to hold this information in confidence.

Table of Contents

I. LIST OF ABBREVIATIONS AND KEY TERMS ... 4
1 INTRODUCTION ... 7
2 STUDY OBJECTIVES AND DESIGN ... 7
2.1 Study Objectives ... 7
2.1.1 Primary Objective ... 7
2.1.2 Secondary Objectives ... 7
2.1.3 Exploratory Objectives ... 7
2.2 Study Design ... 7
2.3 Randomization ... 8
3 SAMPLE SIZE ... 8
4 ANALYSIS SETS ... 9
4.1 Full Analysis Set ... 9
4.2 Safety Analysis Set ... 9
4.3 Pharmacokinetics Analysis Set (PKAS) ... 9
5 SELECT ENDPOINTS FOR INTERIM ANALYSIS ... 9
5.1 Primary Efficacy Endpoint ... 9
5.2 Secondary Efficacy Endpoints ... 9
5.3 Safety Endpoints ... 10
5.3.1 AE ... 10
5.3.2 Clinical Laboratory Variables ... 10
5.3.3 Vital Signs ... 11
5.3.4 12-lead electrocardiogram (ECG) ... 11
5.3.5 ECOG performance score ... 11
5.3.6 Physical examination ... 11
6 INTERIM ANALYSES ... 11
6.1 Periodical Safety Reviews ... 11
6.1.1 Scope of Periodical Safety Analysis ... 11
6.2 Interim OS analysis/Final PFS analysis ... 19
6.2.1 Scope of Interim OS analysis/Final PFS analysis ... 19
6.3 Subgroup Analyses ... 20
6.4 Other Analyses ... 20

7 REVISION AND RATIONALE 20
7.1 List of Changes in IAP Version 2.0 from Version 1.0 (if applicable) 20
8 APPENDICES 21
8.1 TABLES, LISTINGS AND FIGURES (TLFs) SPECIFICATIONS 21
8.2 Author and Approver Signatories 73

I. LIST OF ABBREVIATIONS AND KEY TERMS

List of Abbreviations

Abbreviations	Description of abbreviations
CAPOX	Capecitabine and Oxaliplatin
ADA	anti-drug antibody
AE	adverse event
ALP	alkaline phosphatase
ALT	alanine aminotransferase (GPT)
AST	aspartate aminotransferase (GOT)
ATC	Anatomical Therapeutic Chemical Classification System
BMI	Body mass index
BSA	Body surface area
C1D1	Cycle 1 Day 1
CI	confidence interval
CLDN	Claudin
C_{max}	maximum concentration
CMH	Cochran-Mantel-Haenszel
CR	complete response
CSR	Clinical study report
CTCAE	Common Terminology Criteria For Adverse Events
DCR	disease control rate
DOR	duration of response
ECG	electrocardiogram
ECOG	Eastern Cooperative Oncology Group
eCRF	electronic case report form
EORTC	European Organization for Research and Treatment of Cancer
EQ-5D-5L	EuroQOL Five Dimensions Questionnaire 5L
FAS	full analysis set
GEJ	gastroesophageal junction
GP	Global Pain
HER2	human epidermal growth factor receptor 2
HR	Hazard ratio
HRQoL	health-related quality of life
HRU	Health Resource Utilization
IAP	Interim analysis plan
IDAC	independent data analysis center
IDMC	independent data monitoring committee
INR	international normalized ratio
IRC	independent review committee
IRT	interactive response technology
ISN	international study number
IV	Intravenous, intravenously

**Abbreviations**	**Description of abbreviations**
NCI	National Cancer Institute
NE	not evaluable
ORR	objective response rate
OS	overall survival
PD	progressive disease
PFS	progression free survival
PFS2	progression free survival following second-line anti-cancer treatment
PGx	pharmacogenomics
PKAS	pharmacokinetics analysis set
PPS	per protocol set
PR	partial response
PT	preferred term
QLQ-C30	Quality of Life Questionnaire - Core Questionnaire
QLQ-OG25	Quality of Life Questionnaire - Oesophago-Gastric Module 25 (OG-25)
QoL	Quality of life
QTc	QT interval corrected
QTcF	Fridericia-corrected QT interval
RECIST	Response Evaluation Criteria In Solid Tumors
SAE	serious adverse event
SAF	safety analysis set
SAP	statistical analysis plan
SD	stable disease
SOC	system organ class
SOD	Sum of diameters
TEAE	treatment-emergent adverse event
TSH	thyroid stimulating hormone
TTP	time to progression
ULN	upper limit of normal
VAS	visual analog scale
WHO	World health organization

List of Key Terms

**Terms**	**Definition of terms**
Baseline	Assessments of subjects as they enter a trial before they receive any treatment.
Endpoint	Variable that pertains to the efficacy or safety evaluations of a trial.
Enroll	To register or enter a subject into a clinical trial. NOTE: Once a subject has been randomized, the clinical trial protocol applies to the subject.
Intervention	The drug, device, therapy or process under investigation in a clinical study that is believed to have an effect on outcomes of interest in a study (e.g., health-related quality of life, efficacy, safety, pharmacoeconomics).
Investigational period	Period of time where major interests of protocol objectives are observed, and where the test drug or comparative drug (sometimes without randomization) is usually given to a subject, and continues until the last assessment after completing administration of the test drug or comparative drug.
Post investigational period	Period of time after the last assessment of the protocol. Follow-up observations for sustained adverse events and/or survival are done in this period.
Randomization	The process of assigning trial subjects to treatment or control groups using an element of chance to determine assignments in order to reduce bias.
Screening	A process of active consideration of potential subjects for enrollment in a trial.
Screen failure	Potential subject who did not meet 1 or more criteria required for participation in a trial.
Screening period	Period of time before entering the investigational period, usually from the time when a subject signs the consent until just before the test drug or comparative drug (sometimes without randomization) is given to a subject.
Study period	Period of time from the first site initiation date to the last site completing the study.
Study treatment	Includes zolbetuximab/placebo and all components of CAPOX
Variable	Any entity that varies; any attribute, phenomenon or event that can have different qualitative or quantitative values.

1 INTRODUCTION

This Interim Analysis Plan (IAP) describes the content of the analysis intended for IDMC periodical safety reviews and the final PFS/interim OS analysis. For technical details of analysis, please refer to the study Statistical Analysis Plan (SAP).

2 STUDY OBJECTIVES AND DESIGN

2.1 Study Objectives

2.1.1 Primary Objective

The primary objective is to evaluate the efficacy of zolbetuximab plus CAPOX compared with placebo plus CAPOX (as first-line treatment) as measured by progression free survival (PFS) in subjects with Claudin (CLDN)18.2 positive, HER2–negative locally advanced unresectable or metastatic gastric and GEJ adenocarcinoma.

2.1.2 Secondary Objectives

The secondary objectives are:

- To evaluate efficacy as measured by Overall Survival (OS) as a key secondary objective
- To evaluate the physical function (PF), OG25-Pain and GHS/QoL scores as measured by European Organization for Research and Treatment of Cancer (EORTC) as a key secondary objective
- To evaluate efficacy as measured by Objective Response Rate (ORR)
- To evaluate efficacy as measured by Duration of Response (DOR)
- To evaluate safety and tolerability of zolbetuximab
- To further evaluate other health-related quality of life (HRQoL) using additional parameters as measured by EORTC, QLQ-C30, QLQ-OG25 plus STO22 Belching subscale, Global Pain (GP) and the EuroQOL Five Dimensions (EQ-5D-5L) questionnaires
- To evaluate the pharmacokinetics of zolbetuximab
- To evaluate the immunogenicity profile of zolbetuximab

2.1.3 Exploratory Objectives

The exploratory objectives are:

- To evaluate efficacy as measured by Time to Progression (TTP)
- To evaluate PFS following second-line anti-cancer treatment (PFS2)
- To evaluate Disease Control Rate (DCR)
- To evaluate potential genomic and/or other biomarkers that may correlate with treatment outcome to zolbetuximab and CAPOX.
- To evaluate Health Resource Utilization (HRU)

2.2 Study Design

This global, multi-center, double-blind, 1:1 randomized, phase 3 study will evaluate efficacy of zolbetuximab plus CAPOX versus placebo plus CAPOX as first-line treatment in subjects

with CLDN18.2-positive, HER2-negative locally advanced unresectable or metastatic gastric and GEJ adenocarcinoma.

PFS as assessed by the Independent Review Committee (IRC) is the primary outcome. Secondary outcomes include OS (key secondary endpoint), time to confirmed deterioration, ORR, DOR, safety and tolerability, HRQoL, pharmacokinetic and the immunogenicity profile of zolbetuximab. Exploratory outcomes include TTP, PFS2, DCR, biomarkers and HRU.

One interim analysis and one final analysis are planned for OS, while only one analysis is planned for PFS as the final analysis. The OS interim analysis will occur at the same time of the final PFS analysis (after pre-specified number of PFS events) and final OS analysis will be performed after the pre-specified number of OS events are observed. Refer to Section 6 for details on interim analysis.

Details of the study flow chart, dosing schedule, schedule of assessments are available in the protocol Section V.

2.3 Randomization

Subject randomization will be performed via IRT and treatment is assigned in a 1:1 ratio to:

- Arm A (Zolbetuximab in combination with CAPOX chemotherapy)
- Arm B (placebo in combination with CAPOX chemotherapy)

Prior to the initiation of the study treatment, the unblinded pharmacist/designee will contact the IRT system in order to determine the randomly assigned treatment. The unblinded pharmacist/designee will dispense the treatment according to the IRT system's assignment. Specific procedures for randomization through the IRT are contained in the IRT manual.

Randomization of subjects will use blocked randomization and be stratified by the following factors:

- Region (Asia vs. Non-Asia)
- Number of Metastatic Sites (0 to 2 vs. ≥ 3)
- Prior Gastrectomy (Yes vs. No)

3 SAMPLE SIZE

Approximately 500 subjects will be randomized in a 1:1 ratio to receive Zolbetuximab in combination with CAPOX chemotherapy (Arm A) or placebo in combination with CAPOX chemotherapy (Arm B). The planned 300 PFS events during the study will provide 93.4% power to detect a difference in PFS between Arm A (Zolbetuximab + CAPOX) with the assumption of 9 months median PFS time and Arm B (placebo + CAPOX) with the assumption of 6 months median PFS time (hazard ratio = 0.67) at the overall 1-sided 0.025 significance level. Similarly, the planned 386 OS events during the study will provide 80% power to detect a difference in OS between Arm A (Zolbetuximab + CAPOX) with the assumption of 14.7 months median OS time and Arm B (placebo + CAPOX) with the

assumption of 11 months median OS time (hazard ratio = 0.75) at the overall 1-sided 0.025 significance level.

4 ANALYSIS SETS

In accordance with International Council for Harmonization (ICH) recommendations in guidelines E3 and E9, the following analysis sets will be used for the analyses.

The determination of whether subjects are included or excluded from the safety and efficacy analysis sets will be made prior to each IDMC meeting in a blinded manner.

4.1 Full Analysis Set

The Full Analysis Set (FAS) will consist of all subjects who are randomized to one of the treatment arms. Subjects would be analyzed according to the treatment they were randomized to. The FAS will be used for summaries of demographics and baseline characteristics and all efficacy analyses. FAS in this study is identical to intent-to-treat (ITT) set (only the name "FAS" will be used).

4.2 Safety Analysis Set

The safety analysis set (SAF) will consist of all subjects who received at least one dose of any study drug (Zolbetuximab/placebo/CAPOX). Subjects would be analyzed according to the actual treatment they received. The SAF will be used for summaries of demographic and baseline characteristics and all safety and tolerability-related variables.

4.3 Pharmacokinetics Analysis Set (PKAS)

Not applicable to the IAP.

5 SELECT ENDPOINTS FOR INTERIM ANALYSIS

5.1 Primary Efficacy Endpoint

The primary endpoint is PFS, which is defined as the time from the date of randomization until the date of radiological PD (per Response Evaluation Criteria In Solid Tumors [RECIST] 1.1 by independent review committee [IRC]) or death from any cause, whichever is earlier.

5.2 Secondary Efficacy Endpoints

- OS, defined as the time from the date of randomization until the date of death from any cause.
- TTCD, is defined for the following 3 HRQoL domains: physical functioning (PF) and Global Health Status/Quality of Life (GHS/QoL) as collected in the EORTC QLQ-C30, and abdominal pain and discomfort (OG25-Pain plus STO22) as collected in the EORTC QLQ-OG25 plus STO22). TTCD is defined as the time from the date of randomization until the date of first clinically meaningful deterioration that is confirmed at a next scheduled assessment or followed by drop-out resulting in missing data

- ORR, defined as the proportion of subjects who have a best overall response (BOR) of complete response (CR) or partial response (PR) as assessed by IRC per RECIST 1.1.
- DOR, defined as the time from the date of the first response (CR/PR) until the date of PD as assessed by IRC per RECIST 1.1 or date of death from any cause, whichever is earliest.

5.3 Safety Endpoints

Safety and tolerability endpoints include AEs, laboratory test results, vital signs, electrocardiograms (ECGs) and Eastern Cooperative Oncology Group (ECOG) performance status.

5.3.1 AE

AE will be assessed by evaluation of the following variables:

- Treatment-emergent adverse events (TEAEs; frequency, severity, seriousness, and relationship to study drug)
    - TEAE is defined as an adverse event observed after starting administration of the study drug through 30 days after the last dose of study drug.
    - If the adverse event occurs on Cycle 1 Day 1 and the onset check box is marked “Onset after first dose of study drug” or the onset check box is left blank, then the adverse event will be considered treatment emergent.
    - If the adverse event occurs on Day 1 and the onset check box is marked “Onset before first dose of study drug”, then the adverse event will not be considered treatment emergent.
    - If a subject experiences an event both during the pre-investigational/screening period and during the investigational period, the event will be considered as TEAE only if it is reported with a new start date (i.e., as a new AE).
    - Any AEs with onset dates completely missing will be considered TEAEs in summaries. AEs with partially missing onset dates will be assumed TEAEs unless the available portion of the date indicates that the onset was strictly before start of study medication.
    - A drug-related TEAE is defined as any TEAE with possible relationship to study treatment as assessed by the investigator or with missing assessment of the causal relationship.
- Serious adverse events (SAEs) include adverse events that are flagged as serious by the investigator on eCRF, or upgraded by the Sponsor based on review of the Important Medical Events.

5.3.2 Clinical Laboratory Variables

Refer to protocol Section 5.4.3 for a table of the laboratory tests that will be performed during the conduct of the study. Refer to the Protocol Schedule of Assessments for evaluation schedule.

5.3.3 Vital Signs

Vital signs will include systolic and diastolic blood pressure (mmHg), radial pulse (beats/min) and body temperature. Serial vital signs will be collected during zolbetuximab dosing visits.

5.3.4 12-lead electrocardiogram (ECG)

A single 12-lead ECG will be performed at the time points outlined in the Protocol Schedule of Assessments. ECGs will be read locally.

5.3.5 ECOG performance score

ECOG performance scores will be collected.

5.3.6 Physical examination

Targeted (symptom driven) physical exams should be conducted every 3 weeks on day 1 of each cycle. If clinically significant worsening of findings from baseline is noted at any study visit, the changes will be documented as AEs on the AE eCRF.

6 INTERIM ANALYSES

6.1 Periodical Safety Reviews

Regular meetings will be conducted approximately every 6 months from the first IDMC review meeting. IDMC has the option to adjustthe interval between meetings. The Sponsor study team will not have access to the unblinded randomization schedule or study results. All unblinded study results will be generated by the IDAC using validated programs provided by the Sponsor, and provided to IDMC members prior to each meeting.

At each safety review meeting, IDMC may recommend continuation of trial, or termination/modification of trial based on overwhelming negative safety signals. Trial may be terminated for early efficacy success only at the formal interim OS/final PFs analysis.

Refer to the IDMC charter for more information on the IDMC meetings.

6.1.1 Scope of Periodical Safety Analysis

Table 1 describes analyses that will be included for periodical safety reviews. And Table 2 describes efficacy analyses that will be provided to IDMC only if requested by IDMC post-hoc. If efficacy results are requested, p-values or confidence intervals for hazard ratios will not be included in any efficacy tables or figures for periodical safety reviews. TLFs with p-values will only be provided at the formal interim OS/final PFS analysis.

Table 1 TLFs to be included for periodical safety reviews

Table 9.1.1.1	Disposition Prior to Randomization	All Subjects With Informed Consent
Table 9.1.1.2	Subject Classification	All Randomized Subjects
Table 9.1.1.4.2	End of Treatment - mFOLFOX6	Safety Analysis Set
Table 9.1.1.5.2	End of Treatment - Zolbetuximab/Placebo	Safety Analysis Set
Table 9.1.1.5.3	Overall Treatment Discontinuation	Safety Analysis Set
Table 9.1.2.1.1	Demographic Characteristics	Full Analysis Set
Table 9.1.2.3.1	Stratification Factors Reported at Randomization	Full Analysis Set
Table 9.2.1.1	Study Drug Treatment - Duration of Each Component	Safety Analysis Set
Table 9.2.1.2	Study Drug Treatment - Zolbetuximab/Placebo	Safety Analysis Set
Table 9.2.1.3	Study Drug Treatment - Oxaliplatin	Safety Analysis Set
Table 9.2.1.4	Study Drug Treatment - Capecitabine	Safety Analysis Set
Table 9.2.2.2	Concomitant Medications	Safety Analysis Set
Table 9.6.1.1	Overview of Treatment-Emergent Adverse Events and Death	Safety Analysis Set
Table 9.6.1.2	Treatment-Emergent Adverse Events (MedDRA V23.0)	Safety Analysis Set
Table 9.6.1.6	Serious Treatment-Emergent Adverse Events (MedDRA V23.0)	Safety Analysis Set

Table 9.6.1.7.1	Drug-Related Serious Treatment-Emergent Adverse Events (MedDRA V23.0)	Safety Analysis Set
Table 9.6.1.7.2	Zolbetuximab-Related Serious Treatment-Emergent Adverse Events (MedDRA V23.0)	Safety Analysis Set
Table 9.6.1.7.3	Oxaliplatin-Related Serious Treatment-Emergent Adverse Events (MedDRA V23.0)	Safety Analysis Set
Table 9.6.1.7.4	Capecitabine-Related Serious Treatment-Emergent Adverse Events (MedDRA V23.0)	Safety Analysis Set
Table 9.6.1.8.1	AEs Collected After 30 Days Post Last Dose of Study Drug (MedDRA V23.0)	Safety Analysis Set
Table 9.6.1.8.2	Serious AEs Collected After 30 Days Post Last Dose of Study Drug (MedDRA V23.0)	Safety Analysis Set
Table 9.6.1.14	Treatment-Emergent Adverse Events with NCI-CTCAE >=3 (MedDRA V23.0)	Safety Analysis Set
Table 9.6.1.15.1	Drug-Related Treatment-Emergent Adverse Events with NCI-CTCAE >=3 (MedDRA V23.0)	Safety Analysis Set
Table 9.6.1.15.2	Zolbetuximab-Related Treatment-Emergent Adverse Events with NCI-CTCAE >=3 (MedDRA V23.0)	Safety Analysis Set
Table 9.6.1.15.3	Oxaliplatin-Related Treatment-Emergent Adverse Events with NCI-CTCAE >=3 (MedDRA V23.0)	Safety Analysis Set
Table 9.6.1.15.4	Capecitabine-Related Treatment-Emergent Adverse Events with NCI-CTCAE >=3 (MedDRA V23.0)	Safety Analysis Set
Table 9.6.1.18	Treatment-Emergent Adverse Events (MedDRA V23.0) by Preferred Term	Safety Analysis Set
Table 9.6.1.19	Treatment-Emergent Adverse Events Leading to Death (MedDRA V23.0) by Preferred Term	Safety Analysis Set
Table 9.6.1.21.1.1	Treatment-Emergent Adverse Event of Interest: Nausea or Vomiting or Abdominal Pain (MedDRA V23.0)	Safety Analysis Set
Table 9.6.1.21.1.2	Treatment-Emergent Adverse Event of Interest: Nausea or Vomiting (MedDRA V23.0)	Safety Analysis Set
Table 9.6.1.21.2.1	Serious Treatment-Emergent Adverse Event of Interest: Nausea or Vomiting or Abdominal Pain (MedDRA V23.0)	Safety Analysis Set

Table 9.6.1.21.2.2	Serious Treatment-Emergent Adverse Event of Interest: Nausea or Vomiting (MedDRA V23.0)	Safety Analysis Set
Table 9.6.1.21.3.1	Treatment-Emergent Adverse Event of Interest: Nausea or Vomiting or Abdominal Pain by NCI-CTCAE Grade (MedDRA V23.0)	Safety Analysis Set
Table 9.6.1.21.3.2	Treatment-Emergent Adverse Event of Interest: Nausea or Vomiting by NCI-CTCAE Grade (MedDRA V23.0)	Safety Analysis Set
Table 9.6.1.22.1	Treatment-Emergent Adverse Event of Interest: Hypersensitivity Reactions (MedDRA V23.0)	Safety Analysis Set
Table 9.6.1.22.2	Serious Treatment-Emergent Adverse Event of Interest: Hypersensitivity Reactions (MedDRA V23.0)	Safety Analysis Set
Table 9.6.1.22.3	Treatment-Emergent Adverse Event of Interest: Hypersensitivity Reactions by NCI-CTCAE Grade (MedDRA V23.0)	Safety Analysis Set
Table 9.6.1.23.1.1	Treatment-Emergent Adverse Event of Interest: Infusion-Related Reactions (IRR) by Investigators (MedDRA V23.0)	Safety Analysis Set
Table 9.6.1.23.1.2	Treatment-Emergent Adverse Event of Interest: Potential Infusion-Related Reactions (IRR) (MedDRA V23.0)	Safety Analysis Set
Table 9.6.1.23.2.1	Serious Treatment-Emergent Adverse Event of Interest: Infusion-Related Reactions (IRR) by Investigators (MedDRA V23.0)	Safety Analysis Set
Table 9.6.1.23.2.2	Serious Treatment-Emergent Adverse Event of Interest: Potential Infusion-Related Reactions (IRR) (MedDRA V23.0)	Safety Analysis Set
Table 9.6.1.23.3.1	Treatment-Emergent Adverse Event of Interest: Infusion-Related Reactions (IRR) by Investigators by NCI-CTCAE Grade (MedDRA V23.0)	Safety Analysis Set
Table 9.6.1.23.3.2	Treatment-Emergent Adverse Event of Interest: Potential Infusion-Related Reactions (IRR) by NCI-CTCAE Grade (MedDRA V23.0)	Safety Analysis Set

Table 9.6.1.24.1	Treatment-Emergent Adverse Event of Interest: Anemia (MedDRA V23.0)	Safety Analysis Set
Table 9.6.1.24.2	Serious Treatment-Emergent Adverse Event of Interest: Anemia (MedDRA V23.0)	Safety Analysis Set
Table 9.6.1.24.3	Treatment-Emergent Adverse Event of Interest: Anemia by NCI-CTCAE Grade (MedDRA V23.0)	Safety Analysis Set
Table 9.6.1.25.1	Treatment-Emergent Adverse Event of Interest: Neutropenia (MedDRA V23.0)	Safety Analysis Set
Table 9.6.1.25.2	Serious Treatment-Emergent Adverse Event of Interest: Neutropenia (MedDRA V23.0)	Safety Analysis Set
Table 9.6.1.25.3	Treatment-Emergent Adverse Event of Interest: Neutropenia by NCI-CTCAE Grade (MedDRA V23.0)	Safety Analysis Set
Table 9.6.2.2.2.1	Shift Table for Laboratory Test Results From Baseline to Worst Post-Baseline NCI CTCAE Grade (V4.03), Hematology	Safety Analysis Set
Table 9.6.2.2.2.2	Shift Table for Laboratory Test Results From Baseline to Worst Post-Baseline NCI CTCAE Grade (V4.03), Biochemistry	Safety Analysis Set
Table 9.6.2.2.2.3	Shift Table for Laboratory Test Results From Baseline to Worst Post-Baseline NCI CTCAE Grade (V4.03), Coagulation	Safety Analysis Set
Table 9.6.2.3	Potentially Clinically Significant Values in Liver Enzymes and Total Bilirubin- Central and Local Laboratory	Safety Analysis Set
Table 9.6.5.2.2	Shift Table of ECOG Performance Status	Full Analysis Set
Appendix 10.2.6.16	ECOG Performance Status	All Randomized Subjects

Appendix 10.2.7.3	Adverse Events (MedDRA V23.0) Leading to Death	All Randomized Subjects
Appendix 10.2.7.4	Serious Adverse Events (MedDRA V23.0)	All Randomized Subjects
Appendix 10.2.7.7	Adverse Events (MedDRA V23.0) of Special Interest	All Randomized Subjects
Appendix 10.2.8.1.1.1	Central and Local Laboratory Tests Results in SI Units - Hematology, Part 1 of 5	All Randomized Subjects
Appendix 10.2.8.1.1.2	Central and Local Laboratory Tests Results in SI Units - Hematology, Part 2 of 5	All Randomized Subjects
Appendix 10.2.8.1.1.3	Central and Local Laboratory Tests Results in SI Units - Hematology, Part 3 of 5	All Randomized Subjects
Appendix 10.2.8.1.1.4	Central and Local Laboratory Tests Results in SI Units - Hematology, Part 4 of 5	All Randomized Subjects
Appendix 10.2.8.1.1.5	Central and Local Laboratory Tests Results in SI Units - Hematology, Part 5 of 5	All Randomized Subjects
Appendix 10.2.8.1.2.1	Central and Local Laboratory Tests Results in SI Units - Chemistry, Part 1 of 5	All Randomized Subjects
Appendix 10.2.8.1.2.2	Central and Local Laboratory Tests Results in SI Units - Chemistry, Part 2 of 5	All Randomized Subjects
Appendix 10.2.8.1.2.3	Central and Local Laboratory Tests Results in SI Units - Chemistry, Part 3 of 5	All Randomized Subjects

Appendix 10.2.8.1.2.4	Central and Local Laboratory Tests Results in SI Units - Chemistry, Part 4 of 5	All Randomized Subjects
Appendix 10.2.8.1.2.5	Central and Local Laboratory Tests Results in SI Units - Chemistry, Part 5 of 5	All Randomized Subjects
Appendix 10.2.8.1.3.1	Central and Local Laboratory Tests Results in SI Units - Urinalysis, Part 1 of 7	All Randomized Subjects
Appendix 10.2.8.1.3.2	Central and Local Laboratory Tests Results in SI Units - Urinalysis, Part 2 of 7	All Randomized Subjects
Appendix 10.2.8.1.3.3	Central and Local Laboratory Tests Results in SI Units - Urinalysis, Part 3 of 7	All Randomized Subjects
Appendix 10.2.8.1.3.4	Central and Local Laboratory Tests Results in SI Units - Urinalysis, Part 4 of 7	All Randomized Subjects
Appendix 10.2.8.1.3.5	Central and Local Laboratory Tests Results in SI Units - Urinalysis, Part 5 of 7	All Randomized Subjects
Appendix 10.2.8.1.3.6	Central and Local Laboratory Tests Results in SI Units - Urinalysis, Part 6 of 7	All Randomized Subjects
Appendix 10.2.8.1.3.7	Central and Local Laboratory Tests Results in SI Units - Urinalysis, Part 7 of 7	All Randomized Subjects
Appendix 10.2.8.1.4	Central and Local Laboratory Tests Results in SI Units - Coagulation, Part 1 of 1	All Randomized Subjects
Appendix 10.2.8.1.5.1	Central and Local Laboratory Tests Results in SI Units - Chemokine, Cytokine and Tryptase, Part 1 of 3	All Randomized Subjects

Appendix 10.2.8.1.5.2	Central and Local Laboratory Tests Results in SI Units - Chemokine, Cytokine and Tryptase, Part 2 of 3	All Randomized Subjects
Appendix 10.2.8.1.5.3	Central and Local Laboratory Tests Results in SI Units - Chemokine, Cytokine and Tryptase, Part 3 of 3	All Randomized Subjects
Figure 9.6.2.1	Spaghetti Plot of Laboratory Test Results in SI Units by Time - Biochemistry	Safety Analysis Set
Figure 9.6.2.2	Spaghetti Plot of Laboratory Test Results in SI Units by Time - Hematology	Safety Analysis Set
Figure 9.6.2.3	Spaghetti Plot of Laboratory Test Results in SI Units by Time - Urinalysis	Safety Analysis Set
Figure 9.6.2.4	Spaghetti Plot of Laboratory Test Results in SI Units by Time - Other	Safety Analysis Set
Figure 9.7.1 (Ad-hoc DMC)	Kaplan-Meier Plot of Time to Cessation of Zolbetuximab/Placebo	Safety Analysis Set
Figure 9.7.2 (Ad-hoc DMC)	Kaplan-Meier Plot of Time to Cessation of Oxaliplatin	Safety Analysis Set
Figure 9.7.3(Ad-hoc DMC)	Kaplan-Meier Plot of Time to Cessation of Leucovorin	Safety Analysis Set
Figure 9.7.4 (Ad-hoc DMC)	Kaplan-Meier Plot of Time to Cessation of Fluorouracil Bolus	Safety Analysis Set
Figure 9.7.5 (Ad-hoc DMC)	Kaplan-Meier Plot of Time to Cessation of Fluorouracil	Safety Analysis Set

Table 2 TLFs to be provided to IDMC only if requested after IDMC meetings

Figure No.	Title	
9.3.1.1	Kaplan-Meier Plot of Progression-Free Survival, Independent Radiologic Review (without p-value and CI)	FAS
9.3.1.2	Kaplan-Meier Plot of Progression-Free Survival, Investigator Assessment (without p-value and CI)	FAS
9.3.2	Kaplan-Meier Plot of Overall Survival (without p-value and CI)	FAS

6.2 Interim OS analysis/Final PFS analysis

To evaluate whether Zolbetuximab + CAPOX (Arm A) is beneficial compared to the concurrent placebo + CAPOX (Arm B) while the study is ongoing, a formal OS interim analysis is planned when the final PFS analysis occurs with the pre-specified number (300) of PFS events. A group sequential design using the O'Brien-Fleming type alpha-spending function [Lan & DeMets, 1983] will be utilized to control the overall 1-sided 0.025 significance level (East®) for the OS endpoint. The OS interim and final analyses will be performed only if primary PFS analysis is significant.

The IDMC may recommend terminating the trial for favorable results at the formal efficacy interim analysis using OS. The IDMC may also recommend terminating the trial for negative PFS results. In the case of favorable results, the 1-sided significance level for superiority is 0.0074 for the interim OS analysis and 0.0228 for the final OS analysis. If the 1-sided P value of the interim analysis is less than 0.0074, the IDMC may recommend terminating the trial for success. If the study is not stopped after the interim analysis, a final OS analysis will occur after 100% of the planned death events (386 events) have been observed. Note that the 0.0074 alpha boundary is based on an information factor of 70% (270 death out of planned 386 events) observed at the PFS final/OS interim analysis and may be adjusted prior to interim analysis if the number of observed deaths deviates from that number.

6.2.1 Scope of Interim OS analysis/Final PFS analysis

Table 3 describes analyses that will be included for the interim analysis. The safety analysis listed in Table 1 will also be presented

Table 3 TLFs to be included for interim analysis efficacy part

Table 9.3.1.1	Summary of Progression-Free Survival, Independent Review	FAS
Table 9.3.1.2.1	Summary of Progression-Free Survival, Investigator Assessment	FAS
Table 9.3.2.1	Summary of Overall Survival	FAS
Appendix 10.2.6.2.1	Progression-Free Survival Results, Independent Review	All Randomized Subjects
Appendix 10.2.6.2.2	Progression-Free Survival Results, Investigator Assessment	All Randomized Subjects
Appendix 10.2.6.3	Overall Survival Results, Independent Review	All Randomized Subjects
Figure 9.3.1.1	Kaplan-Meier Plot of Progression-Free Survival, Independent Radiologic Review (with p-value and CI)	FAS
Figure 9.3.1.2	Kaplan-Meier Plot of Progression-Free Survival, Investigator Assessment (with p-value and CI)	FAS
Figure 9.3.2	Kaplan-Meier Plot of Overall Survival (with p-value and CI)	FAS

6.3 Subgroup Analyses

Not applicable.

6.4 Other Analyses

Not applicable.

7 REVISION AND RATIONALE

7.1 List of Changes in IAP Version 2.0 from Version 1.0 (if applicable)

The changes from the approved IAP Version 1.0 (Dated 04-Feb-2019) to Version 2.0 that impact analyses are listed with the rationale in the table below.

SAP Section(s)	Description of Change(s)	Rationale
1-5	Update to match protocol amendment	Protocol amendment on number of events and removal of PPS
6	Further specify scope of upcoming interim analysis	To prepare to interim analysis

8 APPENDICES

8.1 TABLES, LISTINGS AND FIGURES (TLFs) SPECIFICATIONS

Program: </directory/directory/directory/program.sas>
Study 8951-CL-0302

<Draft/Final Version>
Source: <Listing / Dataset>

Table 9.1.1.1
Disposition Prior to Randomization
All Subjects With Informed Consent

Analysis Set	Overall
Subjects with Informed Consent	xx (xx.x%)
Discontinued Before Randomization to Treatment [1]	xx (xx.x%)
Randomized to Treatment	xx (xx.x%)

[1] Patients who signed informed consent but discontinued during screening period or before randomization were screen failures.
For rescreened subjects, the results from the subject's latest screening are shown.

Program: </directory/directory/directory/program.sas>
Study 8951-CL-0302

<Draft/Final Version>
Source: <Listing / Dataset>

Table 9.1.1.2
Subject Classification
All Randomized Subjects

Analysis Set	Arm A (N=xx)	Arm B (N=xx)	Overall (N=xx)
Randomized	xx (xx.x%)	xx (xx.x%)	xx (xx.x%)
Subjects Who Took Study Drug	xx (xx.x%)	xx (xx.x%)	xx (xx.x%)
Subjects Who Did Not Take Study Drug	xx (xx.x%)	xx (xx.x%)	xx (xx.x%)
Safety Analysis Set [1]	xx (xx.x%)	xx (xx.x%)	xx (xx.x%)
Full Analysis Set [2]	xx (xx.x%)	xx (xx.x%)	xx (xx.x%)
Pharmacokinetics Analysis Set [3]	xx (xx.x%)	xx (xx.x%)	xx (xx.x%)

[1] All subjects who received at least one dose of any study drug (Zolbetuximab/Placebo and mFOLFOX6).
[2] All subjects who were randomized to 1 of the treatment arms.
[3] All subjects from the Safety Analysis Set for which at least one concentration data were available.

Program: </directory/directory/directory/program.sas>
Study 8951-CL-0302

<Draft/Final Version>
Source: <Listing / Dataset>

Table 9.1.1.4.1
End of Treatment – CAPOX
Safety Analysis Set

Parameter	Category	Arm A (N=xx)	Arm B (N=xx)	Overall (N=xx)
CAPOX Discontinuation	No <[1]>	xx (xx.x%)	xx (xx.x%)	xx (xx.x%)
	Yes	xx (xx.x%)	xx (xx.x%)	xx (xx.x%)
Primary Study Drug Treatment Status	Completed	xx (xx.x%)	xx (xx.x%)	xx (xx.x%)
	Adverse Event	xx (xx.x%)	xx (xx.x%)	xx (xx.x%)
	Death	xx (xx.x%)	xx (xx.x%)	xx (xx.x%)
	Lost to Follow-Up	xx (xx.x%)	xx (xx.x%)	xx (xx.x%)
	Progressive Disease	xx (xx.x%)	xx (xx.x%)	xx (xx.x%)
	Protocol Deviation	xx (xx.x%)	xx (xx.x%)	xx (xx.x%)
	Withdrawal by Subject	xx (xx.x%)	xx (xx.x%)	xx (xx.x%)
	Pregnancy	xx (xx.x%)	xx (xx.x%)	xx (xx.x%)
	Other	xx (xx.x%)	xx (xx.x%)	xx (xx.x%)

<[1] As the study is ongoing, counts of Discontinuation ‘No’ and Primary Study Drug Treatment Status ‘Completed’ do not match.>

Program: </directory/directory/directory/program.sas>
Study 8951-CL-0302

<Draft/Final Version>
Source: <Listing / Dataset>

Table 9.1.1.5.1
End of Treatment – Zolbetuximab/Placebo
Safety Analysis Set

Parameter	Category	Arm A (N=xx)	Arm B (N=xx)	Overall (N=xx)
Zolbetuximab/Placebo Discontinuation	No	xx (xx.x%)	xx (xx.x%)	xx (xx.x%)
	Yes	xx (xx.x%)	xx (xx.x%)	xx (xx.x%)
Primary Study Drug Treatment Status	Adverse Event	xx (xx.x%)	xx (xx.x%)	xx (xx.x%)
	Death	xx (xx.x%)	xx (xx.x%)	xx (xx.x%)
	Lost to Follow-Up	xx (xx.x%)	xx (xx.x%)	xx (xx.x%)
	Progressive Disease	xx (xx.x%)	xx (xx.x%)	xx (xx.x%)
	Protocol Deviation	xx (xx.x%)	xx (xx.x%)	xx (xx.x%)
	Withdrawal by Subject	xx (xx.x%)	xx (xx.x%)	xx (xx.x%)
	Pregnancy	xx (xx.x%)	xx (xx.x%)	xx (xx.x%)
	Other	xx (xx.x%)	xx (xx.x%)	xx (xx.x%)
If Progressive Disease or Death [1]	Radiographic Progression	xx (xx.x%)	xx (xx.x%)	xx (xx.x%)
	Clinical Progression	xx (xx.x%)	xx (xx.x%)	xx (xx.x%)

[1] Some patients have both radiographic and clinical progression.

Table 9.1.1.5.3
Overall Treatment Discontinuation
Full Analysis Set

Parameter	Category	Overall (N=xx)
Overall Treatment Discontinuation [1]	No	xx (xx.x%)
	Yes	xx (xx.x%)
Reason for Overall Treatment Discontinuation [2]	Adverse Event	xx (xx.x%)
	Death	xx (xx.x%)
	Lost to Follow-Up	xx (xx.x%)
	Progressive Disease	xx (xx.x%)
	Protocol Deviation	xx (xx.x%)
	Withdrawal by Subject	xx (xx.x%)
	Pregnancy	xx (xx.x%)
	Other	xx (xx.x%)

[1] Off both treatments
[2] Subjects with an overall treatment discontinuation are summarized using the reason of the latest discontinued compound.

Program: </directory/directory/directory/program.sas>
Study 8951-CL-0302

<Draft/Final Version>
Source: <Listing / Dataset>

Table 9.1.2.1.1
Demographic Characteristics
Full Analysis Set

Parameter	Category/ Statistic	Arm A (N=xx)	Arm B (N=xx)	Overall (N=xx)
Sex	Male	xx (xx.x%)	xx (xx.x%)	xx (xx.x%)
	Female	xx (xx.x%)	xx (xx.x%)	xx (xx.x%)
	Unknown [1]			
Ethnicity	Hispanic or Latino	xx (xx.x%)	xx (xx.x%)	xx (xx.x%)
	Not Hispanic or Latino	xx (xx.x%)	xx (xx.x%)	xx (xx.x%)
Race	White	xx (xx.x%)	xx (xx.x%)	xx (xx.x%)
	Black or African American	xx (xx.x%)	xx (xx.x%)	xx (xx.x%)
	Asian	xx (xx.x%)	xx (xx.x%)	xx (xx.x%)
	American Indian or Alaska Native	xx (xx.x%)	xx (xx.x%)	xx (xx.x%)
	Native Hawaiian or Other Pacific Islander	xx (xx.x%)	xx (xx.x%)	xx (xx.x%)
	Other	xx (xx.x%)	xx (xx.x%)	xx (xx.x%)
Age (Years)	n	xx	xx	xx
	Mean	xx.x	xx.x	xx.x
	SD	xx.x	xx.x	xx.x
	Min	xx	xx	xx
	Median	xx.x	xx.x	xx.x
	Max	xx	xx	xx
Age Group 1 (Years)	<=65	xx (xx.x%)	xx (xx.x%)	xx (xx.x%)
	>65	xx (xx.x%)	xx (xx.x%)	xx (xx.x%)
Age Group 2 (Years)	<=75	xx (xx.x%)	xx (xx.x%)	xx (xx.x%)
	>75	xx (xx.x%)	xx (xx.x%)	xx (xx.x%)
ECOG Status at Baseline	0	xx (xx.x%)	xx (xx.x%)	xx (xx.x%)
	1	xx (xx.x%)	xx (xx.x%)	xx (xx.x%)
	2	xx (xx.x%)	xx (xx.x%)	xx (xx.x%)
Weight (kg)	n	xx	xx	xx
	Mean	xx.x	xx.x	xx.x
	SD	xx.x	xx.x	xx.x
	Min	xx	xx	xx
	Median	xx.x	xx.x	xx.x
	Max	xx	xx	xx

Table 9.1.2.1.1 (continue)
Demographic Characteristics
Full Analysis Set

Parameter	Category/ Statistic	Arm A (N=xx)	Arm B (N=xx)	Overall (N=xx)
Height (cm)	n	xx	xx	xx
	Mean	xx.x	xx.x	xx.x
	SD	xx.x	xx.x	xx.x
	Min	xx	xx	xx
	Median	xx.x	xx.x	xx.x
	Max	xx	xx	xx
BMI (kg/m^2)	n	xx	xx	xx
	Mean	xx.x	xx.x	xx.x
	SD	xx.x	xx.x	xx.x
	Min	xx	xx	xx
	Median	xx.x	xx.x	xx.x
	Max	xx	xx	xx
BSA (m^2)	n	xx	xx	xx
	Mean	xx.x	xx.x	xx.x
	SD	xx.x	xx.x	xx.x
	Min	xx	xx	xx
	Median	xx.x	xx.x	xx.x
	Max	xx	xx	xx
Tobacco History	Never	xx (xx.x%)	xx (xx.x%)	xx (xx.x%)
	Current	xx (xx.x%)	xx (xx.x%)	xx (xx.x%)
	Former	xx (xx.x%)	xx (xx.x%)	xx (xx.x%)

[1] Unknown is a collected response on the CRF.

Study 8951-CL-0302

Table 9.1.2.3.1
Stratification Factors Reported at Randomization
Full Analysis Set

Source: <Listing / Dataset>

Parameter, n (%)	Arm A (N = XXX)	Arm B (N=XXX)	Overall (N = XXX)
Region=Asia	xxx (xx.x%)	xxx (xx.x%)	xxx (xx.x%)
Region=Non-Asia	xxx (xx.x%)	xxx (xx.x%)	xxx (xx.x%)
Number of Metastatic Sites=0-2	xxx (xx.x%)	xxx (xx.x%)	xxx (xx.x%)
Number of Metastatic Sites=>=3	xxx (xx.x%)	xxx (xx.x%)	xxx (xx.x%)
Prior Gastrectomy=Yes	xxx (xx.x%)	xxx (xx.x%)	xxx (xx.x%)
Prior Gastrectomy=No	xxx (xx.x%)	xxx (xx.x%)	xxx (xx.x%)
Region=Asia, Number of Metastatic Sites=0-2, Prior Gastrectomy=Yes	xxx (xx.x%)	xxx (xx.x%)	xxx (xx.x%)
Region=Asia, Number of Metastatic Sites=0-2, Prior Gastrectomy=No	xxx (xx.x%)	xxx (xx.x%)	xxx (xx.x%)
Region=Asia, Number of Metastatic Sites=>=3, Prior Gastrectomy=Yes	xxx (xx.x%)	xxx (xx.x%)	xxx (xx.x%)
Region=Asia, Number of Metastatic Sites=>=3, Prior Gastrectomy=No	xxx (xx.x%)	xxx (xx.x%)	xxx (xx.x%)
Region=Non-Asia, Number of Metastatic Sites=0-2, Prior Gastrectomy=Yes	xxx (xx.x%)	xxx (xx.x%)	xxx (xx.x%)
Region=Non-Asia, Number of Metastatic Sites=0-2, Prior Gastrectomy=No	xxx (xx.x%)	xxx (xx.x%)	xxx (xx.x%)
Region=Non-Asia, Number of Metastatic Sites=>=3, Prior Gastrectomy=Yes	xxx (xx.x%)	xxx (xx.x%)	xxx (xx.x%)
Region=Non-Asia, Number of Metastatic Sites=>=3, Prior Gastrectomy=No	xxx (xx.x%)	xxx (xx.x%)	xxx (xx.x%)

Program: </directory/directory/directory/program.sas> <Draft/Final Version>
Study 8951-CL-0302 Source: <Listing / Dataset>

Table 9.2.1.1
Study Drug Treatment – Duration of Each Component
Safety Analysis Set

Characteristic		Arm A (N=xx)	Arm B (N=xx)	Overall (N=xx)
Duration of Zolbetuximab/Placebo (days)	n	XX	XX	XX
	Mean	XX.X	XX.X	XX.X
	SD	XX.X	XX.X	XX.X
	Min	XX	XX	XX
	Median	XX.X	XX.X	XX.X
	Max	XX	XX	XX
Duration of Oxaliplatin (days)	n	XX	XX	XX
	Mean	XX.X	XX.X	XX.X
	SD	XX.X	XX.X	XX.X
	Min	XX	XX	XX
	Median	XX.X	XX.X	XX.X
	Max	XX	XX	XX
Duration of Capecitabine (days)	n	XX	XX	XX
	Mean	XX.X	XX.X	XX.X
	SD	XX.X	XX.X	XX.X
	Min	XX	XX	XX
	Median	XX.X	XX.X	XX.X
	Max	XX	XX	XX
Duration of All Components Administered (days) [1]	n	XX	XX	XX
	Mean	XX.X	XX.X	XX.X
	SD	XX.X	XX.X	XX.X
	Min	XX	XX	XX
	Median	XX.X	XX.X	XX.X
	Max	XX	XX	XX
Duration of Any Component Administered (days) [2]	n	XX	XX	XX
	Mean	XX.X	XX.X	XX.X
	SD	XX.X	XX.X	XX.X
	Min	XX	XX	XX
	Median	XX.X	XX.X	XX.X
	Max	XX	XX	XX
CAPOX (24 weeks) Treatment Completed	n	XX (XX.X%)	XX (XX.X%)	XX (XX.X%)

Duration of each component is defined as (date of last dose) – (date of first dose) + 1.

Arm A components: Zolbetuximab, Oxaliplatin and Capecitabine;
Arm B components: Placebo, Oxaliplatin and Capecitabine.
[1] Duration of all components administered is defined as (date of last dose of the earliest component discontinued) - (date of C1 D1) + 1 for subject who discontinued at least one drug and (date of last dose) – (date of C1 D1) + 1 for ongoing subjects.
[2] Duration of any component administered is defined as (date of last dose of the last component discontinued) - (date of C1 D1) + 1 for subject who discontinued at least one drug and (date of last dose - date of C1 D1) + 1 for ongoing subjects.

Study 8951-CL-0302

Table 9.2.1.2.1

Source: <Listing / Dataset>

Study Drug Treatment – Zolbetuximab/Placebo
Safety Analysis Set

Characteristic		Arm A (N=xx)	Arm B (N=xx)	Overall (N=xx)
Cumulative Actual Dose (mg)	n	XX	XX	XX
	Mean	XX.XX	XX.XX	XX.XX
	SD	XX.XX	XX.XX	XX.XX
	Min	XX.X	XX.X	XX.X
	Median	XX.XX	XX.XX	XX.XX
	Max	XX.X	XX.X	XX.X
Relative Dose Intensity (%) [1]	n	XX	XX	XX
	Mean	XX.X	XX.X	XX.X
	SD	XX.X	XX.X	XX.X
	Min	XX	XX	XX
	Median	XX.X	XX.X	XX.X
	Max	XX	XX	XX
Relative Dose Intensity Category (%)	<50	XX (XX.X%)	XX (XX.X%)	XX (XX.X%)
	>=50 to <=80	XX (XX.X%)	XX (XX.X%)	XX (XX.X%)
	>80	XX (XX.X%)	XX (XX.X%)	XX (XX.X%)
	Unknonwn	XX (XX.X%)	XX (XX.X%)	XX (XX.X%)
Number of Infusions Administered [2]	n	XX	XX	XX
	Mean	XX.X	XX.X	XX.X
	SD	XX.X	XX.X	XX.X
	Min	XX	XX	XX
	Median	XX.X	XX.X	XX.X
	Max	XX	XX	XX
Number of Infusions Entirely Administered	n	XX	XX	XX
	Mean	XX.X	XX.X	XX.X
	SD	XX.X	XX.X	XX.X
	Min	XX	XX	XX
	Median	XX.X	XX.X	XX.X
	Max	XX	XX	XX
Number of Infusions Not Entirely Administered	n	XX	XX	XX
	Mean	XX.X	XX.X	XX.X
	SD	XX.X	XX.X	XX.X
	Min	XX	XX	XX
	Median	XX.X	XX.X	XX.X
	Max	XX	XX	XX

Program: </directory/directory/directory/program.sas>
Study 8951-CL-0302

<Draft/Final Version>
Source: <Listing / Dataset>

Table 9.2.1.2
Study Drug Treatment – Zolbetuximab/Placebo
Safety Analysis Set

Characteristic		Arm A (N=xx)	Arm B (N=xx)	Overall (N=xx)
Cumulative Actual Dose (mg/m2)	n	XX	XX	XX
	Mean	XX.XXX	XX.XXX	XX.XXX
	SD	XX.XXX	XX.XXX	XX.XXX
	Min	XX.XX	XX.XX	XX.XX
	Median	XX.XXX	XX.XXX	XX.XXX
	Max	XX.XX	XX.XX	XX.XX
Relative Dose Intensity (%) [1]	n	XX	XX	XX
	Mean	XX.XXX	XX.XXX	XX.XXX
	SD	XX.XXX	XX.XXX	XX.XXX
	Min	XX.XX	XX.XX	XX.XX
	Median	XX.XXX	XX.XXX	XX.XXX
	Max	XX.XX	XX.XX	XXXX
Relative Dose Intensity Category (%)	<50	XX (XX.X%)	XX (XX.X%)	XX (XX.X%)
	>=50 to <=80	XX (XX.X%)	XX (XX.X%)	XX (XX.X%)
	>80	XX (XX.X%)	XX (XX.X%)	XX (XX.X%)
	Unknonwn	XX (XX.X%)	XX (XX.X%)	XX (XX.X%)
Number of Infusions Administered [2]	n	XX	XX	XX
	Mean	XX.X	XX.X	XX.X
	SD	XX.X	XX.X	XX.X
	Min	XX	XX	XX
	Median	XX.X	XX.X	XX.X
	Max	XX	XX	XX
Number of Infusions Entirely Administered [3]	n	XX	XX	XX
	Mean	XX.X	XX.X	XX.X
	SD	XX.X	XX.X	XX.X
	Min	XX	XX	XX
	Median	XX.X	XX.X	XX.X
	Max	XX	XX	XX
Number of Infusions Not Entirely Administered [3]	n	XX	XX	XX
	Mean	XX.X	XX.X	XX.X
	SD	XX.X	XX.X	XX.X
	Min	XX	XX	XX
	Median	XX.X	XX.X	XX.X
	Max	XX	XX	XX

Program: </directory/directory/directory/program.sas>
Study 8951-CL-0302

<Draft/Final Version>
Source: <Listing / Dataset>

Table 9.2.1.2 (continued)
Study Drug Treatment – Zolbetuximab/Placebo
Safety Analysis Set

Characteristic		Arm A (N=xx)	Arm B (N=xx)	Overall (N=xx)
Average Infusion Time per Infusion (hr/infusion) [4]	n	XX	XX	XX
	Mean	XX.XXX	XX.XXX	XX.XXX
	SD	XX.XXX	XX.XXX	XX.XXX
	Min	XX.XX	XX.XX	XX.XX
	Median	XX.XXX	XX.XXX	XX.XXX
	Max	XX.XX	XX.XX	XX.XX
	Max	XX.XX	XX.XX	XX.XX
Average Dose Per Infusion ((mg/m2)/infusion) [5]	n	XX	XX	XX
	Mean	XX.XXX	XX.XXX	XX.XXX
	SD	XX.XXX	XX.XXX	XX.XXX
	Min	XX.XX	XX.XX	XX.XX
	Median	XX.XXX	XX.XXX	XX.XXX
	Max	XX.XX	XX.XX	XX.XX
Average Flow Rate Adjustement Per Infusion [6]	n	XX	XX	XX
	Mean	XX.X	XX.X	XX.X
	SD	XX.X	XX.X	XX.X
	Min	XX	XX	XX
	Median	XX.X	XX.X	XX.X
	Max	XX	XX	XX
Reason for Flow Rate Adjusted	Adverse Event	XX (XX.X%)	XX (XX.X%)	XX (XX.X%)
	Adverse Event Prevention	XX (XX.X%)	XX (XX.X%)	XX (XX.X%)
	Other	XX (XX.X%)	XX (XX.X%)	XX (XX.X%)
Infusions Not Administered	n	XX (XX.X%)	XX (XX.X%)	XX (XX.X%)
Reason for Infusion Not Administered	Adverse Event	XX (XX.X%)	XX (XX.X%)	XX (XX.X%)
	COVID-19 Diagnosis Adverse Event	XX (XX.X%)	XX (XX.X%)	XX (XX.X%)
	Other COVID-19 Reasons/Control Measures/Quarantine	XX (XX.X%)	XX (XX.X%)	XX (XX.X%)
	Other	XX (XX.X%)	XX (XX.X%)	XX (XX.X%)
...	...	...	...	...
Duration of Treatment Cumulative Category	>=1 day	XX (XX.X%)	XX (XX.X%)	XX (XX.X%)
	>6 weeks	XX (XX.X%)	XX (XX.X%)	XX (XX.X%)
	>12 weeks	XX (XX.X%)	XX (XX.X%)	XX (XX.X%)
	>24 weeks	XX (XX.X%)	XX (XX.X%)	XX (XX.X%)

Characteristic	Arm A (N=xx)	Arm B (N=xx)	Overall (N=xx)
>36 weeks	XX (XX.X%)	XX (XX.X%)	XX (XX.X%)
>48 weeks	XX (XX.X%)	XX (XX.X%)	XX (XX.X%)
>72 weeks	XX (XX.X%)	XX (XX.X%)	XX (XX.X%)

[1] Defined as (actual cumulative dose / planned cumulative dose)* 100%. Planned dose intensity is protocol specified (Section 5.1.1.1).
[2] Number of infusions per subject over the entire study period.
[3] Subjects can contribute to both numbers (entirely administered and not entirely administered infusions), so that n’s do not sum up to N.
[4] Defined as [sum of (stop infusion) – (start infusion time) over different cycles]/(number of infustion administered). For infusions with interruptions that goes over night, overnight interruption time will be excluded from infusion time.
[5] Defined as (cumulative dose)/(number of infusions administered).
[6] Defined as the average over infusions of the number of flow rate adjustment.

Note: This shell will be used for the following tables:

Program: </directory/directory/directory/program.sas> <Draft/Final Version>
Study 8951-CL-0302 Source: <Listing / Dataset>

Table 9.2.1.3
Study Drug Treatment – Oxaliplatin
Safety Analysis Set

Program: </directory/directory/directory/program.sas> <Draft/Final Version>

Table 9.2.1.4
Study Drug Treatment – Capecitabine
Safety Analysis Set

Characteristic		Arm A (N=xx)	Arm B (N=xx)	Overall (N=xx)
Cumulative Actual Dose (mg)	n	XX	XX	XX
	Mean	XX.XXX	XX.XXX	XX.XXX
	SD	XX.XXX	XX.XXX	XX.XXX
	Min	XX.XX	XX.XX	XX.XX
	Median	XX.XXX	XX.XXX	XX.XXX
	Max	XX.XX	XX.XX	XX.XX
Relative Dose Intensity (%) [1]	n	XX	XX	XX
	Mean	XX.XXX	XX.XXX	XX.XXX
	SD	XX.XXX	XX.XXX	XX.XXX
	Min	XX.XX	XX.XX	XX.XX
	Median	XX.XXX	XX.XXX	XX.XXX
	Max	XX.XX	XX.XX	XX.XX
Relative Dose Intensity Category (%)	<50	XX (XX.X%)	XX (XX.X%)	XX (XX.X%)
	>=50 to <=80	XX (XX.X%)	XX (XX.X%)	XX (XX.X%)
	>80	XX (XX.X%)	XX (XX.X%)	XX (XX.X%)
	Unknonwn	XX (XX.X%)	XX (XX.X%)	XX (XX.X%)
Number of Cycles [2]	n	XX	XX	XX
	Mean	XX.X	XX.X	XX.X
	SD	XX.X	XX.X	XX.X
	Min	XX	XX	XX
	Median	XX.X	XX.X	XX.X
	Max	XX	XX	XX
Dose Adjustment	n	XX (XX.X%)	XX (XX.X%)	XX (XX.X%)
Reason for Dose Adjustment	Adverse Event	XX (XX.X%)	XX (XX.X%)	XX (XX.X%)
	Other	XX (XX.X%)	XX (XX.X%)	XX (XX.X%)
	COVID-19 Diagnosis Adverse Event	XX (XX.X%)	XX (XX.X%)	XX (XX.X%)

Characteristic		Arm A (N=xx)	Arm B (N=xx)	Overall (N=xx)
	Other COVID-19 Reasons/Control Measures/Quarantine	XX (XX.X%)	XX (XX.X%)	XX (XX.X%)

Program: </directory/directory/directory/program.sas>
Study 8951-CL-0302

<Draft/Final Version>
Source: <Listing / Dataset>

Table 9.2.1.4 (continue)
Study Drug Treatment – Capecitabine
Safety Analysis Set

Characteristic		Arm A (N=xx)	Arm B (N=xx)	Overall (N=xx)
Dose Interruption	n	XX (XX.X%)	XX (XX.X%)	XX (XX.X%)
Reason for Dose Interruption	Adverse Event	XX (XX.X%)	XX (XX.X%)	XX (XX.X%)
	Protocol Specified	XX (XX.X%)	XX (XX.X%)	XX (XX.X%)
	Other	XX (XX.X%)	XX (XX.X%)	XX (XX.X%)
	COVID-19 Diagnosis Adverse Event	XX (XX.X%)	XX (XX.X%)	XX (XX.X%)
	Other COVID-19 Reasons/Control Measures/Quarantine	XX (XX.X%)	XX (XX.X%)	XX (XX.X%)
...	...	...	...	...
Reason for Dose Discontinuation	Adverse Event	XX (XX.X%)	XX (XX.X%)	XX (XX.X%)
	Progressive Disease	XX (XX.X%)	XX (XX.X%)	XX (XX.X%)
	Other	XX (XX.X%)	XX (XX.X%)	XX (XX.X%)
	COVID-19 Diagnosis Adverse Event	XX (XX.X%)	XX (XX.X%)	XX (XX.X%)
	Other COVID-19 Reasons/Control Measures/Quarantine	XX (XX.X%)	XX (XX.X%)	XX (XX.X%)
Average Amount of Dose per Planned Dosing Day [3]	n	XX	XX	XX
	Mean	XX.XXX	XX.XXX	XX.XXX
	SD	XX.XXX	XX.XXX	XX.XXX
	Min	XX.XX	XX.XX	XX.XX
	Median	XX.XXX	XX.XXX	XX.XXX

Characteristic		Arm A (N=xx)	Arm B (N=xx)	Overall (N=xx)
	Max	XX.XX	XX.XX	XX.XX
Duration of Treatment Cumulative Category	>=1 day	XX (XX.X%)	XX (XX.X%)	XX (XX.X%)
	>6 weeks	XX (XX.X%)	XX (XX.X%)	XX (XX.X%)
	>12 weeks	XX (XX.X%)	XX (XX.X%)	XX (XX.X%)
	>24 weeks	XX (XX.X%)	XX (XX.X%)	XX (XX.X%)
	>36 weeks	XX (XX.X%)	XX (XX.X%)	XX (XX.X%)
	>48 weeks	XX (XX.X%)	XX (XX.X%)	XX (XX.X%)
	>72 weeks	XX (XX.X%)	XX (XX.X%)	XX (XX.X%)

[1] Defined as (actual cumulative dose/planned cumulative dose)*100%. Planned dose intensity is protocol specified (Section 5.1.1.3).
[2] Number of completed cycles by the subject over the entire study period.
[3] Defined as (cumulative dose)/(14*(number of cycles done)).

Program: </directory/directory/directory/program.sas>
Study 8951-CL-0302

<Draft/Final Version>
Source: <Listing / Dataset>

Table 9.2.2.2
Concomitant Medications
Safety Analysis Set

Therapeutic Subgroup (ATC 2nd level)<br>Chemical Subgroup (ATC 4th level)<br>Preferred WHO Name	Arm A<br>(N=xx)	Arm B<br>(N=xx)	Overall<br>(N=xx)
Overall	XX (XX.X%)	XX (XX.X%)	XX (XX.X%)
Therapeutic Subgroup 1	XX (XX.X%)	XX (XX.X%)	XX (XX.X%)
Chemical Subgroup 1	XX (XX.X%)	XX (XX.X%)	XX (XX.X%)
Preferred WHO Name A	XX (XX.X%)	XX (XX.X%)	XX (XX.X%)
Preferred WHO Name B	XX (XX.X%)	XX (XX.X%)	XX (XX.X%)
Chemical Subgroup 2	XX (XX.X%)	XX (XX.X%)	XX (XX.X%)
Preferred WHO Name C	XX (XX.X%)	XX (XX.X%)	XX (XX.X%)
Preferred WHO Name D	XX (XX.X%)	XX (XX.X%)	XX (XX.X%)
Therapeutic Subgroup 2	XX (XX.X%)	XX (XX.X%)	XX (XX.X%)
...	...	...	...
...	...	...	...

Number of subjects and percentage of subjects (%) are shown.
Sorting order: alphabetical order by Therapeutic Subgroup and Chemical Subgroup and Preferred WHO Name .
Medications used on the date of first dose of study drug or later are shown.
Medications that started prior to first administration of study drug and continued while study drug was given will be counted as both previous and concomitant medications.
A medication which can be classified into several chemical and/or therapeutic subgroups is presented in all chemical and therapeutic subgroups.

Program: </directory/directory/directory/program.sas>
Study 8951-CL-0302

<Draft/Final Version>
Source: <Listing / Dataset>

Table 9.3.1.1
Summary of Progression-Free Survival, Independent Review
Full Analysis Set

Measure	Arm A (N = XXX)	Arm B (N = XXX)
PFS Events, n (%)	XX (XX.X%)	XX (XX.X%)
Radiographical Progression	XX (XX.X%)	XX (XX.X%)
Death without Documented Progression	XX (XX.X%)	XX (XX.X%)
Censored, n (%)	XX (XX.X%)	XX (XX.X%)
Duration of PFS (months) [1]		
Median (95% CI)	XX.X (XX.X, XX.X)	XX.X (XX.X, XX.X)
1st Quartile (95% CI)	XX.X (XX.X, XX.X)	XX.X (XX.X, XX.X)
3rd Quartile (95% CI)	XX.X (XX.X, XX.X)	XX.X (XX.X, XX.X)
Range [2]	X.XX+, XX.XX	X.XX, XX.XX+
Stratified Analysis [3]		
1-sided P-value [4]	0.xxx	0.xxx
Hazard Ratio (95% CI) [5]	x.xxx (x.xxx, x.xxx)	x.xxx (x.xxx, x.xxx)
PFS Rate, % (95% CI) [6]		
6 months	XX.X% (XX.X%, XX.X%)	XX.X% (XX.X%, XX.X%)
12 months	XX.X% (XX.X%, XX.X%)	XX.X% (XX.X%, XX.X%)
18 months	XX.X% (XX.X%, XX.X%)	XX.X% (XX.X%, XX.X%)

PFS is defined as the time from randomization until death from any cause or radiographic disease progression assessed according to RECIST 1.1, whichever occurs first. For a subject with none of these events, progression free survival was censored based on rules defined in SAP.
[1] Based on Kaplan-Meier estimate. [2] + indicates censoring.
[3] Stratification factors are Region, Number of Metastatic Sites and Prior Gastrectomy from eCRF. [4] Based on log-rank test.
[5] Based on Cox proportional hazards model with treatment, region, number of metastatic sites, and prior gastrectomy as the explanatory variables. Assuming proportional hazards, a hazard ratio < 1 indicates a reduction in hazard rate in favor of treatment arm.
[6] PFS rate and 95% CI are estimated using Kaplan-Meier method and Greenwood formula.

This shell will be used for the following tables:

Program: </directory/directory/directory/program.sas> <Draft/Final Version>
Study 8951-CL-0302 Table 9.3.1.2.1 Source: <Listing / Dataset>

Summary of Progression-Free Survival, Investigator Assessment
Sensitivity Analysis
Full Analysis Set

Program: </directory/directory/directory/program.sas> <Draft/Final Version>
Study 8951-CL-0302 Source: <Listing / Dataset>

Table 9.3.2.1
Summary of Overall Survival
Full Analysis Set

Measure	Arm A (N = XXX)	Arm B (N = XXX)
Deaths, n (%)	xxx (xx.x%)	xxx (xx.x%)
Censored, n (%)	xxx (xx.x%)	xxx (xx.x%)
Censored at Cutoff Date, n (%)	xxx (xx.x%)	xxx (xx.x%)
Duration of Overall Survival, Months [1]		
Median (95% CI)	xx.xx (xx.xx, xx.xx)	NE (xx.xx, NE)
1st Quartile (95% CI)	xx.xx (xx.xx, xx.xx)	xx.xx (xx.xx, xx.xx)
3rd Quartile (95% CI)	xx.xx (xx.xx, xx.xx)	xx.xx (xx.xx, xx.xx)
Range [2]	<0.03+, 30.14>	<1.00, 10.13+>
Stratified Analysis (Primary) [3]		
1-sided P-value [4]	0.xxx	0.xxx
Hazard Ratio (95% CI) [5]	x.xxx (x.xxx, x.xxx)	x.xxx (x.xxx, x.xxx)
Overall Survival Rate, % (95% CI) [6]		
At 12 months	xx.x% (xx.x%, xx.x%)	xx.x% (xx.x%, xx.x%)
At 18 months	xx.x% (xx.x%, xx.x%)	xx.x% (xx.x%, xx.x%)
At 24 months	xx.x% (xx.x%, xx.x%)	xx.x% (xx.x%, xx.x%)

NE=Non-Estimable
[1] Based on Kaplan-Meier estimate. [2] + indicates censoring.
[3] Stratification factors were Region, Number of Metastatic Sites and Prior Gastrectomy from eCRF. [4] Based on log-rank test.
[5] Based on Cox proportional hazards model with treatment, region, number of metastatic sites, and prior gastrectomy as the explanatory variables. Assuming proportional hazards, a hazard ratio < 1 indicates a reduction in hazard rate in favor of treatment arm.
[6] Survival Rate and 95% CI were estimated using Kaplan-Meier method.

Table 9.6.1.1
Overview of Treatment-Emergent Adverse Events and Death
Safety Analysis Set

Source: <Listing / Dataset>

	Arm A (N=xx) N (%)	Arm B (N=xx) n (%)	Overall (N=xx) N (%)
TEAE	XX (XX.X%)	XX (XX.X%)	XX (XX.X%)
Drug-Related [1] TEAE	XX (XX.X%)	XX (XX.X%)	XX (XX.X%)
Zolbetuximab-Related Treatment-Emergent Adverse Events	XX (XX.X%)	XX (XX.X%)	XX (XX.X%)
Oxaliplatin-Related Treatment-Emergent Adverse Events	XX (XX.X%)	XX (XX.X%)	XX (XX.X%)
Capecitabine-Related Treatment-Emergent Adverse Events	XX (XX.X%)	XX (XX.X%)	XX (XX.X%)
Serious TEAE [2]	XX (XX.X%)	XX (XX.X%)	XX (XX.X%)
Drug-Related [1] Serious TEAE [2]	XX (XX.X%)	XX (XX.X%)	XX (XX.X%)
Zolbetuximab-Related Serious Treatment-Emergent Adverse Events	XX (XX.X%)	XX (XX.X%)	XX (XX.X%)
Oxaliplatin-Related Serious Treatment-Emergent Adverse Events	XX (XX.X%)	XX (XX.X%)	XX (XX.X%)
Capecitabine-Related Serious Treatment-Emergent Adverse Events	XX (XX.X%)	XX (XX.X%)	XX (XX.X%)
TEAE Leading to Death	XX (XX.X%)	XX (XX.X%)	XX (XX.X%)
Drug-Related [1] TEAE Leading to Death	XX (XX.X%)	XX (XX.X%)	XX (XX.X%)
Zolbetuximab-Related Treatment-Emergent Adverse Events Leading to Death	XX (XX.X%)	XX (XX.X%)	XX (XX.X%)
Oxaliplatin-Related Treatment-Emergent Adverse Events Leading to Death	XX (XX.X%)	XX (XX.X%)	XX (XX.X%)
Capecitabine-Related Treatment-Emergent Adverse Events Leading to Death	XX (XX.X%)	XX (XX.X%)	XX (XX.X%)
TEAE Leading to Permanent Discontinuation of Study Drug [3]	XX (XX.X%)	XX (XX.X%)	XX (XX.X%)
TEAE Leading to Permanent Discontinuation of Zolbetuximab/Placebo	XX (XX.X%)	XX (XX.X%)	XX (XX.X%)
TEAE Leading to Permanent Discontinuation of Oxaliplatin	XX (XX.X%)	XX (XX.X%)	XX (XX.X%)
TEAE Leading to Permanent Discontinuation of Capecitabine	XX (XX.X%)	XX (XX.X%)	XX (XX.X%)
Drug-Related [1] TEAE Leading to Permanent Discontinuation of Study Drug [3]	XX (XX.X%)	XX (XX.X%)	XX (XX.X%)
Drug-Related [1] TEAE Leading to Permanent Discontinuation of Zolbetuximab/Placebo	XX (XX.X%)	XX (XX.X%)	XX (XX.X%)
Drug-Related [1] TEAE Leading to Permanent Discontinuation of Oxaliplatin	XX (XX.X%)	XX (XX.X%)	XX (XX.X%)
Drug-Related [1] TEAE Leading to Permanent Discontinuation of Capecitabine	XX (XX.X%)	XX (XX.X%)	XX (XX.X%)
TEAE Leading to Interruption of Study Drug [3]	XX (XX.X%)	XX (XX.X%)	XX (XX.X%)
TEAE Leading to Interruption of Zolbetuximab/Placebo	XX (XX.X%)	XX (XX.X%)	XX (XX.X%)
TEAE Leading to Interruption of Oxaliplatin	XX (XX.X%)	XX (XX.X%)	XX (XX.X%)

TEAE Leading to Interruption of Capecitabine	XX (XX.X%)	XX (XX.X%)	XX (XX.X%)
Drug-Related [1] TEAE Leading to Interruption of Study Drug [3]	XX (XX.X%)	XX (XX.X%)	XX (XX.X%)
Drug-Related [1] TEAE Leading to Interruption of Zolbetuximab/Placebo	XX (XX.X%)	XX (XX.X%)	XX (XX.X%)
Drug-Related [1] TEAE Leading to Interruption of Oxaliplatin	XX (XX.X%)	XX (XX.X%)	XX (XX.X%)
Drug-Related [1] TEAE Leading to Interruption of Capecitabine	XX (XX.X%)	XX (XX.X%)	XX (XX.X%)
Late Adverse Event [4]	XX (XX.X%)	XX (XX.X%)	XX (XX.X%)
Late Serious Adverse Event [4]	XX (XX.X%)	XX (XX.X%)	XX (XX.X%)
Worst CTCAE Grade TEAE [5]			
>=3	XX (XX.X%)	XX (XX.X%)	XX (XX.X%)
3	XX (XX.X%)	XX (XX.X%)	XX (XX.X%)
4	XX (XX.X%)	XX (XX.X%)	XX (XX.X%)
5	XX (XX.X%)	XX (XX.X%)	XX (XX.X%)
Drug-Related [1] Worst CTCAE Grade TEAE [5]			
>=3	XX (XX.X%)	XX (XX.X%)	XX (XX.X%)
3	XX (XX.X%)	XX (XX.X%)	XX (XX.X%)
4	XX (XX.X%)	XX (XX.X%)	XX (XX.X%)
5	XX (XX.X%)	XX (XX.X%)	XX (XX.X%)
Zolbetuximab-Related Worst CTCAE Grade>=3 Treatment-Emergent Adverse	XX (XX.X%)	XX (XX.X%)	XX (XX.X%)
Oxaliplatin-Related Worst CTCAE Grade>=3 Treatment-Emergent Adverse	XX (XX.X%)	XX (XX.X%)	XX (XX.X%)
Capecitabine-Related Worst CTCAE Grade>=3 Treatment-Emergent Adverse	XX (XX.X%)	XX (XX.X%)	XX (XX.X%)
Death [6]	XX (XX.X%)	XX (XX.X%)	XX (XX.X%)

Number of subjects (n) and percentage of subjects (%) are shown. TEAE: Treatment Emergent Adverse Event.
[1] Possible or probable, as assessed by the investigator, or records where relationship is missing.
[2] Includes SAEs upgraded by the sponsor based on review of the Sponsor's list of Always Serious terms, if any upgrade was done.
[3] Zolbetuximab and any component of CAPOX. For drug-related TEAE of a particular study drug component, only TEAE related to that particular component will be counted. [4] Adverse Event/Serious Adverse Event occurred beyond 30 days from the last study treatment.
[5] If a subject has an event more than once with missing severity grade and non-missing severity grade, then the subject will be counted as the highest non-missing grade.
[6] All reported deaths after the first study drug administration up to analysis cutoff date.

Study 8951-CL-0302 Table 9.6.1.2 Source: <Listing / Dataset>

Treatment-Emergent Adverse Events (MedDRA V23.0)

Safety Analysis Set

System Organ Class<br>Preferred Term	Arm A<br>(N=xx)	Arm B<br>(N=xx)	Overall<br>(N=xx)
Overall	XX (XX.X%)	XX (XX.X%)	XX (XX.X%)
System Organ Class 1	XX (XX.X%)	XX (XX.X%)	XX (XX.X%)
Preferred Term A	XX (XX.X%)	XX (XX.X%)	XX (XX.X%)
Preferred Term B	XX (XX.X%)	XX (XX.X%)	XX (XX.X%)
System Organ Class 2	XX (XX.X%)	XX (XX.X%)	XX (XX.X%)
Preferred Term C	XX (XX.X%)	XX (XX.X%)	XX (XX.X%)
...	...	...	...

Number of subjects (n) and percentage of subjects (%) are shown.
Sorting order: alphabetical order by System Organ Class and descending by the number of subjects of Overall group by Preferred Term. In case of ties, alphabetical order by Preferred Term is applied.

This shell will be repeated for the following tables:

Program: </directory/directory/directory/program.sas> <Draft/Final Version>

Study 8951-CL-0302 Table 9.6.1.8.1 Source: <Listing / Dataset>

AEs Collected After 30 Days Post Last Dose of Study Drug (MedDRA V20.1)

Safety Analysis Set

Program: </directory/directory/directory/program.sas> <Draft/Final Version>
Study 8951-CL-0302 Table 9.6.1.6 Source: <Listing / Dataset>
Serious Treatment-Emergent Adverse Events (MedDRA V23.0)
Safety Analysis Set

System Organ Class<br>Preferred Term	Arm A<br>(N=XX)<br>n (%)	 <br>#E	Arm B<br>(N=XX)<br>n (%)	 <br>#E	Overall<br>(N=XX)<br>n (%)	 <br>#E
Overall	XX (XX.X%)	E	XX (XX.X%)	E	XX (XX.X%)	E
System Organ Class 1	XX (XX.X%)	E	XX (XX.X%)	E	XX (XX.X%)	E
Preferred Term A	XX (XX.X%)	E	XX (XX.X%)	E	XX (XX.X%)	E
Preferred Term B	XX (XX.X%)	E	XX (XX.X%)	E	XX (XX.X%)	E
System Organ Class 2	XX (XX.X%)	E	XX (XX.X%)	E	XX (XX.X%)	E
Preferred Term C	XX (XX.X%)	E	XX (XX.X%)	E	XX (XX.X%)	E
...	...		...		...	

Number of subjects (n), percentage of subjects (%), and number of events (#E) are shown.
Sorting order: alphabetical order by System Organ Class and descending by the number of subjects of Overall group by Preferred Term. In case of ties, alphabetical order by Preferred Term is applied.

This shell will be used for the following tables:

Program: </directory/directory/directory/program.sas> <Draft/Final Version>
Study 8951-CL-0302 Table 9.6.1.7.1 Source: <Listing / Dataset>
Drug-Related Serious Treatment-Emergent Adverse Events (MedDRA V23.0)
Safety Analysis Set

Program: </directory/directory/directory/program.sas> <Draft/Final Version>
Study 8951-CL-0302 Table 9.6.1.7.2 Source: <Listing / Dataset>
Zolbetuximab-Related Serious Treatment-Emergent Adverse Events (MedDRA V23.0)
Safety Analysis Set

Program: </directory/directory/directory/program.sas> <Draft/Final Version>
Study 8951-CL-0302 Table 9.6.1.7.3 Source: <Listing / Dataset>
Oxaliplatin -Related Serious Treatment-Emergent Adverse Events (MedDRA V23.0)
Safety Analysis Set
Program: </directory/directory/directory/program.sas> <Draft/Final Version>
Study 8951-CL-0302 Table 9.6.1.7.4 Source: <Listing / Dataset>
Capecitabine -Related Serious Treatment-Emergent Adverse Events (MedDRA V23.0)
Safety Analysis Set
Program: </directory/directory/directory/program.sas> <Draft/Final Version>

Study 8951-CL-0302

Table 9.6.1.8.2
Serious AEs Collected After 30 Days Post Last Dose of Study Drug (MedDRA V23.0)
Safety Analysis Set

Source: <Listing / Dataset>

Program: </directory/directory/directory/program.sas>
<Draft/Final Version>
Study 8951-CL-0302
Source: <Listing / Dataset>

Table 9.6.1.14
Treatment-Emergent Adverse Events with NCI-CTCAE >=3 (MedDRA V23.0)
Safety Analysis Set

System Organ Class<br>Preferred Term	Maximum<br>CTCAE Grade<br>[1]	Arm A<br>(N=XX)<br>n (%)	Arm B<br>(N=XX)<br>n (%)	Overall<br>(N=XX)<br>n (%)
Overall	Grade 3	X (XX.X%)	X (XX.X%)	X (XX.X%)
	Grade 4	X (XX.X%)	X (XX.X%)	X (XX.X%)
	Grade 5	X (XX.X%)	X (XX.X%)	X (XX.X%)
	Missing	X (XX.X%)	X (XX.X%)	X (XX.X%)
	Total	X (XX.X%)	X (XX.X%)	X (XX.X%)
System Organ Class 1	Grade 3	X (XX.X%)	X (XX.X%)	X (XX.X%)
	Grade 4	X (XX.X%)	X (XX.X%)	X (XX.X%)
	Grade 5	X (XX.X%)	X (XX.X%)	X (XX.X%)
	Missing	X (XX.X%)	X (XX.X%)	X (XX.X%)
	Total	X (XX.X%)	X (XX.X%)	X (XX.X%)
Preferred Term A	Grade 3	X (XX.X%)	X (XX.X%)	X (XX.X%)
	Grade 4	X (XX.X%)	X (XX.X%)	X (XX.X%)
	Grade 5	X (XX.X%)	X (XX.X%)	X (XX.X%)
	Missing	X (XX.X%)	X (XX.X%)	X (XX.X%)
	Total	X (XX.X%)	X (XX.X%)	X (XX.X%)
...	...	...	...	...

Number of subjects (n), percentage of subjects (%) are shown.
Sorting order: alphabetical order by System Organ Class and descending by the number of subjects of Overall group by Preferred Term. In case of ties, alphabetical order by Preferred Term is applied.
[1] Subject counted once under maximum severity. If a subject has an event more than once with missing severity grade and non-missing severity grade, then the subject will be counted as the highest non-missing grade.

Note: This shell is standard table TAE_003. This shell will be used for the following table:

Program: </directory/directory/directory/program.sas>
<Draft/Final Version>
Study 8951-CL-0302
Source: <Listing / Dataset>

Table 9.6.1.15.1
Drug-Related Treatment-Emergent Adverse Events with NCI-CTCAE >=3 (MedDRA V23.0)
Safety Analysis Set

Program: </directory/directory/directory/program.sas>
<Draft/Final Version>
Study 8951-CL-0302
Source: <Listing / Dataset>

Table 9.6.1.15.2
Zolbetuximab-Related Treatment-Emergent Adverse Events with NCI-CTCAE >=3 (MedDRA V23.0)

Safety Analysis Set

Program: </directory/directory/directory/program.sas> <Draft/Final Version>
Study 8951-CL-0302 Table 9.6.1.15.3 Source: <Listing / Dataset>
Oxaliplatin-Related Treatment-Emergent Adverse Events with NCI-CTCAE >=3 (MedDRA V23.0)
Safety Analysis Set

Program: </directory/directory/directory/program.sas> <Draft/Final Version>
Study 8951-CL-0302 Table 9.6.1.15.4 Source: <Listing / Dataset>
Capecitabine -Related Treatment-Emergent Adverse Events with NCI-CTCAE >=3 (MedDRA V23.0)
Safety Analysis Set

Program: </directory/directory/directory/program.sas>
Study 8951-CL-0302

<Draft/Final Version>
Source: <Listing / Dataset>

Table 9.6.1.18
Treatment-Emergent Adverse Events (MedDRA V23.0) by Preferred Term
Safety Analysis Set

Preferred Term	Arm A (N=XX) n (%)	Arm B (N=XX) n (%)	Overall (N=XX) n (%)
Overall	XX (XX.X%)	XX (XX.X%)	XX (XX.X%)
Preferred Term A	XX (XX.X%)	XX (XX.X%)	XX (XX.X%)
Preferred Term B	XX (XX.X%)	XX (XX.X%)	XX (XX.X%)
Preferred Term C	XX (XX.X%)	XX (XX.X%)	XX (XX.X%)
...	...	...	...

Number of subjects and percentage of subjects (%) are shown.
Sorting order: descending by the number of subjects of Overall group by Preferred Term. In case of ties, alphabetical order by Preferred Term is applied.

Program: </directory/directory/directory/program.sas>
Study 8951-CL-0302

<Draft/Final Version>
Source: <Listing / Dataset>

Table 9.6.1.19
Treatment-Emergent Adverse Events Leading to Death (MedDRA V23.0) by Preferred Term
Safety Analysis Set

Preferred Term	Arm A (N=XX) n (%)	#E	Arm B (N=XX) n (%)	#E	Overall (N=XX) n (%)	#E
Overall	XX (XX.X%)	E	XX (XX.X%)	E	XX (XX.X%)	E
Preferred Term A	XX (XX.X%)	E	XX (XX.X%)	E	XX (XX.X%)	E
Preferred Term B	XX (XX.X%)	E	XX (XX.X%)	E	XX (XX.X%)	E
Preferred Term C	XX (XX.X%)	E	XX (XX.X%)	E	XX (XX.X%)	E
...	...		...		...	

Number of subjects and percentage of subjects (%) and number of events (#E) are shown.
Sorting order: descending by the number of subjects of Overall group by Preferred Term. In case of ties, alphabetical order by Preferred Term is applied.

Program: </directory/directory/directory/program.sas> <Draft/Final Version>
Study 8951-CL-0302 Table 9.6.1.21.1.1 Source: <Listing / Dataset>
Treatment-Emergent Adverse Event of Interest: Nausea or Vomiting or Abdominal Pain (MedDRA V23.0)
Safety Analysis Set

System Organ Class<br>Preferred Term	Arm A<br>(N=XX)	Arm B<br>(N=XX)	Overall<br>(N=XX)
Nausea or vomiting or abdominal pain based on PT terms	XX (XX.X%)	XX (XX.X%)	XX (XX.X%)
System Organ Class 1	XX (XX.X%)	XX (XX.X%)	XX (XX.X%)
Preferred Term A	XX (XX.X%)	XX (XX.X%)	XX (XX.X%)
Preferred Term B	XX (XX.X%)	XX (XX.X%)	XX (XX.X%)
...	...	...	...

Number of subjects (n) and percentage of subjects (%) are shown.
Sorting order: alphabetical order by System Organ Class and descending by the number of subjects of Overall group by Preferred Term. In case of ties, alphabetical order by Preferred Term is applied.

This shell will be used for the following tables:

Program: </directory/directory/directory/program.sas> <Draft/Final Version>
Study 8951-CL-0302 Table 9.6.1.21.1.2 Source: <Listing / Dataset>
Treatment-Emergent Adverse Event of Interest: Nausea or Vomiting (MedDRA V23.0)
Safety Analysis Set

Program: </directory/directory/directory/program.sas> <Draft/Final Version>
Study 8951-CL-0302 Table 9.6.1.22.1 Source: <Listing / Dataset>
Treatment-Emergent Adverse Event of Interest: Hypersensitivity Reactions (MedDRA V23.0)
Safety Analysis Set

Program: </directory/directory/directory/program.sas> <Draft/Final Version>
Study 8951-CL-0302 Table 9.6.1.23.1.1 Source: <Listing / Dataset>
Treatment-Emergent Adverse Event of Interest: Infusion-Related Reactions (IRR) by Investigators (MedDRA V23.0)
Safety Analysis Set

Program: </directory/directory/directory/program.sas> <Draft/Final Version>
Study 8951-CL-0302 Table 9.6.1.23.1.2 Source: <Listing / Dataset>
Treatment-Emergent Adverse Event of Interest: Potential Infusion-Related Reactions (IRR) (MedDRA V23.0)
Safety Analysis Set

Program: </directory/directory/directory/program.sas> <Draft/Final Version>
Study 8951-CL-0302 Table 9.6.1.24.1 Source: <Listing / Dataset>
Treatment-Emergent Adverse Event of Interest: Anemia (MedDRA V23.0)
Safety Analysis Set

Program: </directory/directory/directory/program.sas> <Draft/Final Version>
Study 8951-CL-0302 Table 9.6.1.25.1 Source: <Listing / Dataset>
Treatment-Emergent Adverse Event of Interest: Neutropenia (MedDRA V23.0)
Safety Analysis Set

Program: </directory/directory/directory/program.sas> <Draft/Final Version>
Study 8951-CL-0302 Table 9.6.1.21.2.1 Source: <Listing / Dataset>
Serious Treatment-Emergent Adverse Event of Interest: Nausea or Vomiting or Abdominal Pain (MedDRA V23.0)
Safety Analysis Set

System Organ Class<br>Preferred Term	Arm A<br>(N=XX)<br>n (%) #E	Arm B<br>(N=XX)<br>n (%) #E	Overall<br>(N=XX)<br>n (%) #E
Nausea or vomiting or abdominal pain based on PT terms	XX (XX.X%) E	XX (XX.X%) E	XX (XX.X%) E
System Organ Class 1	XX (XX.X%) E	XX (XX.X%) E	XX (XX.X%) E
Preferred Term A	XX (XX.X%) E	XX (XX.X%) E	XX (XX.X%) E
Preferred Term B	XX (XX.X%) E	XX (XX.X%) E	XX (XX.X%) E
...	...	...	...

Number of subjects (n), percentage of subjects (%), and number of events (#E) are shown.
Sorting order: alphabetical order by System Organ Class and descending by the number of subjects of Overall group by Preferred Term. In case of ties, alphabetical order by Preferred Term is applied.

This shell will be used for the following tables:

Program: </directory/directory/directory/program.sas> <Draft/Final Version>
Study 8951-CL-0302 Table 9.6.1.21.2.2 Source: <Listing / Dataset>
Serious Treatment-Emergent Adverse Event of Interest: Nausea or Vomiting (MedDRA V23.0)
Safety Analysis Set

Program: </directory/directory/directory/program.sas> <Draft/Final Version>
Study 8951-CL-0302 Table 9.6.1.22.2 Source: <Listing / Dataset>
Serious Treatment-Emergent Adverse Event of Interest: Hypersensitivity Reactions (MedDRA V23.0)
Safety Analysis Set

Program: </directory/directory/directory/program.sas> <Draft/Final Version>
Study 8951-CL-0302 Table 9.6.1.23.2.1 Source: <Listing / Dataset>
Serious Treatment-Emergent Adverse Event of Interest: Infusion-Related Reactions (IRR) by Investigators (MedDRA V23.0)
Safety Analysis Set

Program: </directory/directory/directory/program.sas> <Draft/Final Version>
Study 8951-CL-0302 Table 9.6.1.23.2.2 Source: <Listing / Dataset>
Serious Treatment-Emergent Adverse Event of Interest: Potential Infusion-Related Reactions (IRR) (MedDRA V23.0)
Safety Analysis Set

Program: </directory/directory/directory/program.sas> <Draft/Final Version>
Study 8951-CL-0302 Table 9.6.1.24.2 Source: <Listing / Dataset>
Serious Treatment-Emergent Adverse Event of Interest: Anemia (MedDRA V23.0)
Safety Analysis Set

Program: </directory/directory/directory/program.sas> <Draft/Final Version>
Study 8951-CL-0302 Table 9.6.1.25.2 Source: <Listing / Dataset>
Serious Treatment-Emergent Adverse Event of Interest: Neutropenia (MedDRA V23.0)
Safety Analysis Set

Program: </directory/directory/directory/program.sas> <Draft/Final Version>
Study 8951-CL-0302 Table 9.6.1.21.3.1 Source: <Listing / Dataset>
Treatment-Emergent Adverse Event of Interest: Nausea or Vomiting or Abdominal Pain by NCI-CTCAE Grade (MedDRA V23.0)
Safety Analysis Set

System Organ Class<br>Preferred Term	Maximum<br>CTCAE Grade<br>[1]	Arm A<br>(N=XX)<br>n (%)	Arm B<br>(N=XX)<br>n (%)	Overall<br>(N=XX)<br>n (%)
Nausea or Vomiting or Abdominal Pain by NCI-CTCAE Grade	Grade 1	X (XX.X%)	X (XX.X%)	X (XX.X%)
	Grade 2	X (XX.X%)	X (XX.X%)	X (XX.X%)
	Grade 3	X (XX.X%)	X (XX.X%)	X (XX.X%)
	Grade 4	X (XX.X%)	X (XX.X%)	X (XX.X%)
	Grade 5	X (XX.X%)	X (XX.X%)	X (XX.X%)
	Missing	X (XX.X%)	X (XX.X%)	X (XX.X%)
	Total	X (XX.X%)	X (XX.X%)	X (XX.X%)
System Organ Class 1	Grade 1	X (XX.X%)	X (XX.X%)	X (XX.X%)
	Grade 2	X (XX.X%)	X (XX.X%)	X (XX.X%)
	Grade 3	X (XX.X%)	X (XX.X%)	X (XX.X%)
	Grade 4	X (XX.X%)	X (XX.X%)	X (XX.X%)
	Grade 5	X (XX.X%)	X (XX.X%)	X (XX.X%)
	Missing	X (XX.X%)	X (XX.X%)	X (XX.X%)
	Total	X (XX.X%)	X (XX.X%)	X (XX.X%)
...	...	...	...	...

Number of subjects (n), percentage of subjects (%) are shown.
Sorting order: alphabetical order by System Organ Class and descending by the number of subjects of Overall group by Preferred Term. In case of ties, alphabetical order by Preferred Term is applied.
[1] Subject counted once under maximum severity. If a subject has an event more than once with missing severity grade and non-missing severity grade, then the subject will be counted as the highest non-missing grade.

This shell will be used for the following tables:

Program: </directory/directory/directory/program.sas> <Draft/Final Version>
Study 8951-CL-0302 Table 9.6.1.21.3.2 Source: <Listing / Dataset>
Treatment-Emergent Adverse Event of Interest: Nausea or Vomiting by NCI-CTCAE Grade (MedDRA V23.0)
Safety Analysis Set

Program: </directory/directory/directory/program.sas> <Draft/Final Version>
Study 8951-CL-0302 Table 9.6.1.22.3 Source: <Listing / Dataset>
Treatment-Emergent Adverse Event of Interest: Hypersensitivity Reactions by NCI-CTCAE Grade (MedDRA V23.0)
Safety Analysis Set

Program: </directory/directory/directory/program.sas> <Draft/Final Version>
Study 8951-CL-0302 Table 9.6.1.23.3.1 Source: <Listing / Dataset>
Treatment-Emergent Adverse Event of Interest: Infusion-Related Reactions (IRR) by Investigators by NCI-CTCAE Grade (MedDRA V23.0)
Safety Analysis Set

Program: </directory/directory/directory/program.sas> <Draft/Final Version>
Study 8951-CL-0302 Table 9.6.1.23.3.2 Source: <Listing / Dataset>
Treatment-Emergent Adverse Event of Interest: Potential Infusion-Related Reactions (IRR) by NCI-CTCAE Grade (MedDRA V23.0)
Safety Analysis Set

Program: </directory/directory/directory/program.sas> <Draft/Final Version>
Study 8951-CL-0302 Table 9.6.1.24.3 Source: <Listing / Dataset>
Treatment-Emergent Adverse Event of Interest: Anemia by NCI-CTCAE Grade (MedDRA V23.0)
Safety Analysis Set

Program: </directory/directory/directory/program.sas> <Draft/Final Version>
Study 8951-CL-0302 Table 9.6.1.25.3 Source: <Listing / Dataset>
Treatment-Emergent Adverse Event of Interest: Neutropenia by NCI-CTCAE Grade (MedDRA V23.0)
Safety Analysis Set

Program: </directory/directory/directory/program.sas> <Draft/Final Version>
Study 8951-CL-0302 Table 9.6.2.2.2.1 Source: <Listing / Dataset>

Shift Table for Laboratory Test Results From Baseline to Worst Post-Baseline NCI CTCAE Grade (V4.03), Hematology
Based on Central and Local Assessment
Safety Analysis Set

Laboratory test = XXX (unit) for 'Low'

Treatment Group	Worst Post-Baseline Grade	Baseline Grade: Grade 0 [1]	Grade 1	Grade 2	Grade 3	Grade 4	Total	No Data
Arm A (N=XX)	Grade 0 [1]	XX (XX.X%)	XX (XX.X%)	XX (XX.X%)	XX (XX.X%)	XX (XX.X%)	XX	XX
	Grade 1	XX (XX.X%)	XX (XX.X%)	XX (XX.X%)	XX (XX.X%)	XX (XX.X%)	XX	XX
	Grade 2	XX (XX.X%)	XX (XX.X%)	XX (XX.X%)	XX (XX.X%)	XX (XX.X%)	XX	XX
	Grade 3	XX (XX.X%)	XX (XX.X%)	XX (XX.X%)	XX (XX.X%)	XX (XX.X%)	XX	XX
	Grade 4	XX (XX.X%)	XX (XX.X%)	XX (XX.X%)	XX (XX.X%)	XX (XX.X%)	XX	XX
	Total	XX	XX	XX	XX	XX	XX	XX
	No Data	XX	XX	XX	XX	XX	XX	XX
Arm B (N=XX)	Grade 0 [1]	XX (XX.X%)	XX (XX.X%)	XX (XX.X%)	XX (XX.X%)	XX (XX.X%)	XX	XX
	Grade 1	XX (XX.X%)	XX (XX.X%)	XX (XX.X%)	XX (XX.X%)	XX (XX.X%)	XX	XX
	Grade 2	XX (XX.X%)	XX (XX.X%)	XX (XX.X%)	XX (XX.X%)	XX (XX.X%)	XX	XX
	Grade 3	XX (XX.X%)	XX (XX.X%)	XX (XX.X%)	XX (XX.X%)	XX (XX.X%)	XX	XX
	Grade 4	XX (XX.X%)	XX (XX.X%)	XX (XX.X%)	XX (XX.X%)	XX (XX.X%)	XX	XX
	Total	XX	XX	XX	XX	XX	XX	XX
	No Data	XX	XX	XX	XX	XX	XX	XX
Overall (N=XX)	Grade 0 [1]	XX (XX.X%)	XX (XX.X%)	XX (XX.X%)	XX (XX.X%)	XX (XX.X%)	XX	XX
	Grade 1	XX (XX.X%)	XX (XX.X%)	XX (XX.X%)	XX (XX.X%)	XX (XX.X%)	XX	XX
	Grade 2	XX (XX.X%)	XX (XX.X%)	XX (XX.X%)	XX (XX.X%)	XX (XX.X%)	XX	XX
	Grade 3	XX (XX.X%)	XX (XX.X%)	XX (XX.X%)	XX (XX.X%)	XX (XX.X%)	XX	XX
	Grade 4	XX (XX.X%)	XX (XX.X%)	XX (XX.X%)	XX (XX.X%)	XX (XX.X%)	XX	XX
	Total	XX	XX	XX	XX	XX	XX	XX
	No Data	XX	XX	XX	XX	XX	XX	XX

Percentages in the table are based on column total (number of subjects who had particular grade at baseline).
Baseline NCI-CTCAE grade is the last non-missing grade before first dose.
[1] 'Grade 0' indicates lab test results that are outside CTCAE criteria.

Repeat for the following Table:

Program: </directory/directory/directory/program.sas> <Draft/Final Version>
Study 8951-CL-0302 Table 9.6.2.2.2.2 Source: <Listing / Dataset>

Shift Table for Laboratory Test Results From Baseline to Worst Post-Baseline NCI CTCAE Grade (V4.03), Biochemistry
Based on Central and Local Assessment
Safety Analysis Set

Program: </directory/directory/directory/program.sas> <Draft/Final Version>
Study 8951-CL-0302 Table 9.6.2.2.2.3 Source: <Listing / Dataset>

Shift Table for Laboratory Test Results From Baseline to Worst Post-Baseline NCI CTCAE Grade (V4.03), Coagulation
Based on Central and Local Assessment
Safety Analysis Set

Program: </directory/directory/directory/program.sas>
<Draft/Final Version>
Study 8951-CL-0302
Source: <Listing / Dataset>

Table 9.6.2.3
Potentially Clinically Significant Values in Liver Enzymes and Total Bilirubin – Central and Local Laboratory
Safety Analysis Set

Parameter [1]	Criteria	Arm A (N=XX)	Arm B (N=XX)	Overall (N=XX)
ALT	> 3xULN	XX/XX (XX.X%)	XX/XX (XX.X%)	XX/XX (XX.X%)
	> 5xULN	XX/XX (XX.X%)	XX/XX (XX.X%)	XX/XX (XX.X%)
	> 10xULN	XX/XX (XX.X%)	XX/XX (XX.X%)	XX/XX (XX.X%)
	> 20xULN	XX/XX (XX.X%)	XX/XX (XX.X%)	XX/XX (XX.X%)
AST	> 3xULN	XX/XX (XX.X%)	XX/XX (XX.X%)	XX/XX (XX.X%)
	> 5xULN	XX/XX (XX.X%)	XX/XX (XX.X%)	XX/XX (XX.X%)
	> 10xULN	XX/XX (XX.X%)	XX/XX (XX.X%)	XX/XX (XX.X%)
	> 20xULN	XX/XX (XX.X%)	XX/XX (XX.X%)	XX/XX (XX.X%)
ALT or AST	> 3xULN	XX/XX (XX.X%)	XX/XX (XX.X%)	XX/XX (XX.X%)
	> 5xULN	XX/XX (XX.X%)	XX/XX (XX.X%)	XX/XX (XX.X%)
	> 10xULN	XX/XX (XX.X%)	XX/XX (XX.X%)	XX/XX (XX.X%)
	> 20xULN	XX/XX (XX.X%)	XX/XX (XX.X%)	XX/XX (XX.X%)
Total Bilirubin	> 2xULN	XX/XX (XX.X%)	XX/XX (XX.X%)	XX/XX (XX.X%)
ALP	> 1.5xULN	XX/XX (XX.X%)	XX/XX (XX.X%)	XX/XX (XX.X%)
ALT and/or AST AND Total Bilirubin [1]	(ALT and/or AST > 3xULN) AND Total Bilirubin > 2xULN	XX/XX (XX.X%)	XX/XX (XX.X%)	XX/XX (XX.X%)
ALT and/or AST AND Total Bilirubin AND ALP [1]	(ALT and/or AST > 3xULN) AND Total Bilirubin > 2xULN and ALP < 2xULN	XX/XX (XX.X%)	XX/XX (XX.X%)	XX/XX (XX.X%)

Note: Percentages were calculated based on the total number of subjects who had at least one non-missing value during treatment.
[1] For each subject, the worst value among all post-baseline measurements at the visit or up to 1 day apart was used.
ULN = upper limit of normal range

Program: </directory/directory/directory/program.sas>
Study 8951-CL-0302

<Draft/Final Version>
Source: <Listing / Dataset>

Table 9.6.5.1.2
Shift Table of ECOG Performance Status
Full Analysis Set

		Baseline Value							
Treatment Group	Worst Post-Baseline Value	0	1	2	3	4	5	Total	No Data
Arm A (N=XX)	0	XX (XX.X%)	XX (XX.X%)	XX (XX.X%)	XX (XX.X%)	XX (XX.X%)	XX (XX.X%)	XX	XX
	1	XX (XX.X%)	XX (XX.X%)	XX (XX.X%)	XX (XX.X%)	XX (XX.X%)	XX (XX.X%)	XX	XX
	2	XX (XX.X%)	XX (XX.X%)	XX (XX.X%)	XX (XX.X%)	XX (XX.X%)	XX (XX.X%)	XX	XX
	3	XX (XX.X%)	XX (XX.X%)	XX (XX.X%)	XX (XX.X%)	XX (XX.X%)	XX (XX.X%)	XX	XX
	4	XX (XX.X%)	XX (XX.X%)	XX (XX.X%)	XX (XX.X%)	XX (XX.X%)	XX (XX.X%)	XX	XX
	5	XX (XX.X%)	XX (XX.X%)	XX (XX.X%)	XX (XX.X%)	XX (XX.X%)	XX (XX.X%)	XX	XX
	Total	XX	XX	XX	XX	XX	XX	XX	XX
	No Data	XX	XX	XX	XX	XX	XX	XX	XX
Arm B (N=XX)	0	XX (XX.X%)	XX (XX.X%)	XX (XX.X%)	XX (XX.X%)	XX (XX.X%)	XX (XX.X%)	XX	XX
	1	XX (XX.X%)	XX (XX.X%)	XX (XX.X%)	XX (XX.X%)	XX (XX.X%)	XX (XX.X%)	XX	XX
	2	XX (XX.X%)	XX (XX.X%)	XX (XX.X%)	XX (XX.X%)	XX (XX.X%)	XX (XX.X%)	XX	XX
	3	XX (XX.X%)	XX (XX.X%)	XX (XX.X%)	XX (XX.X%)	XX (XX.X%)	XX (XX.X%)	XX	XX
	4	XX (XX.X%)	XX (XX.X%)	XX (XX.X%)	XX (XX.X%)	XX (XX.X%)	XX (XX.X%)	XX	XX
	5	XX (XX.X%)	XX (XX.X%)	XX (XX.X%)	XX (XX.X%)	XX (XX.X%)	XX (XX.X%)	XX	XX
	Total	XX	XX	XX	XX	XX	XX	XX	XX
	No Data	XX	XX	XX	XX	XX	XX	XX	XX
Overall (N=XX)	0	XX (XX.X%)	XX (XX.X%)	XX (XX.X%)	XX (XX.X%)	XX (XX.X%)	XX (XX.X%)	XX	XX
	1	XX (XX.X%)	XX (XX.X%)	XX (XX.X%)	XX (XX.X%)	XX (XX.X%)	XX (XX.X%)	XX	XX
	2	XX (XX.X%)	XX (XX.X%)	XX (XX.X%)	XX (XX.X%)	XX (XX.X%)	XX (XX.X%)	XX	XX
	3	XX (XX.X%)	XX (XX.X%)	XX (XX.X%)	XX (XX.X%)	XX (XX.X%)	XX (XX.X%)	XX	XX
	4	XX (XX.X%)	XX (XX.X%)	XX (XX.X%)	XX (XX.X%)	XX (XX.X%)	XX (XX.X%)	XX	XX
	5	XX (XX.X%)	XX (XX.X%)	XX (XX.X%)	XX (XX.X%)	XX (XX.X%)	XX (XX.X%)	XX	XX
	Total	XX	XX	XX	XX	XX	XX	XX	XX
	No Data	XX	XX	XX	XX	XX	XX	XX	XX

Percentages in the table were based on column total (number of subjects who had particular severity at baseline).
Baseline value is from C1D1 if C1D1 is available, otherwise baseline value is from screening.

Program: </directory/directory/directory/program.sas> <Draft/Final Version>
Study 8951-CL-0302 Appendix 10.2.6.2.1 Source: <Listing / Dataset>
Progression-Free Survival Results, Independent Review
All Randomized Subject

Planned Arm	Subject ID	Age(years)/ Sex/ Race[1]/ Weight(kg)	Date of Randomization	Day/Date of Last Non-PD Assessment [2]	Day/ Date of PD	Day/Date of Death	Day/Date of New ACT [3]	Day/Date of Event After New ACT [4]	PFS (Months)	PFS (INF) (Months) [5]	PFS2 (months)
Country = xxxx, Investigator Site = xxxxxx											
XXXXXXXX	XXXXXXXXX	43/F/W/45	DDMMMYYYY	15/ DDMMMYYYY	22/ DDMMMYYYY	24/ DDMMMYYYY	xx/ DDMMMYYYY	xx/ DDMMMYYYY	x.x+	x.x+	x.x+
XXXXXXXX	XXXXXXXXX										

+ indicates censoring. INF= Informative Censoring, PFS2= Progression Free Survival After Subsequent Therapy (PFS2).
[1] F=Female, M=Male; Race:W=White, B=Black/African American, P=Native Hawaiian or other Pacific islander, A=Asian, I=American Indian or Alaska native, O=Other. [2] Last Non-PD assessment is the last imaging assessment prior to disease progression.
[3] Anti-cancer therapy. [4] All events/censoring are described in SAP Table 5. [5] Definition in SAP section 6.4.1.4.

This shell will be used for the following Listing:

Program: </directory/directory/directory/program.sas> <Draft/Final Version>
Study 8951-CL-0302 Appendix 10.2.6.2.2 Source: <Listing / Dataset>
Progression-Free Survival Results, Investigator Assessment
All Randomized Subject

Program: </directory/directory/directory/program.sas> <Draft/Final Version>
Study 8951-CL-0302 Source: <Listing / Dataset>

Appendix 10.2.6.3
Overall Survival Results, Independent Review
All Randomized Subject

Planned Arm	Subject ID	Age(years)/ Sex/ Race[1]/ Weight(kg)	Date of Enrollment	Day/ Date of Overall Survival [2]	Overall Survival [3] (months)
Country = xxxx, Investigator Site = xxxxxx					
XXXXXXXX	XXXXXXXXX #	43/F/W/45	DDMMMYYYY	24/ DDMMMYYYY	15/ DDMMMYYYY+
XXXXXXXX	XXXXXXXXX	...	...	...	...

[1] F=Female, M=Male; Race:W=White, B=Black/African American, P=Native Hawaiian or other Pacific islander, A=Asian, I=American Indian or Alaska native, O=Other.
[2] Day/Date of death or last known alive.
[3] + indicates censoring

Program: </directory/directory/directory/program.sas>
<Draft/Final Version>
Study 8951-CL-0302
Source: <Listing / Dataset>

Appendix 10.2.7.1
Adverse Events (MedDRA V23.0)
All Randomized Subjects

Actual Arm	Subject ID	Age(years)/ Sex/ Race[1]/ Weight(kg)	Last Dose Day/Date	System Organ Class/ Preferred term/ Reported term	Onset Day/ Onset Date&Time/ End Day/ End Date&Time/ Onset Timing[2]/ TEAE	Serious (Reason) [3]/ Course of Event/ NCI CTCAE Grade	Relationship/ Outcome/ Treatment Required (Medication/Non-Medication Therapy)	Action Taken	Previous Subject ID	Related to COVID-19 Pandemic
Country = xxxx, Investigator Site = xxxxxx										
xxxx	xxxxxx	43/F/W/45	xxxx/ YYYY-MM-DD	SOC/ xxxx/ Xxxx	xxxx/ YYYY-MM-DDThh:mm/ xxxx/ YYYY-MM-DDThh:mm/ Before/ Yes	xxxx/ xxxx/ xxxx	Z: YES/ RECOVERED/ YES(MEDICATION)	Z: Interrupted / Ox: Unknown/ Leu: Unknown/ FluoB: Unknown/ Fluo: Unknown		Yes
				SOC/ xxxx/ Xxxx	xxxx/ YYYY-MM-DDThh:mm/ xxxx/ YYYY-MM-DDThh:mm/ After/ No	xxxx/ xxxx/ xxxx	xxxx/ xxxx/ Xxxx			
xxxx	xxxxx	43/F/W/45	xxxx/ YYYY-MM-DD	Psychiatric disorders/ Nervousness / FEELING SHAKY	3/ 2007-09-03T16:04/ 9 E/ 2007-09	Yes (RPH, D)/ Single Episode/ Grade 1	NOT RELATED/ COMPLETELY RECOVERED/ YES(MEDICATION,NON-MEDICATION)	Xxxxx: xxxxxxxxxx/ Xxxxx: xxxxxxxxxx/ Xxxxx: xxxxxxxxxx/ Xxxxx: xxxxxxxxxx/ Xxxxx: xxxxxxxxxxx	xxxxxx	

E: estimated value, Fluo: Fluorouracil, FluoB: Fluorouracil Bolus, Leu: Leucovorin, Ox: Oxaliplatin, Z: Zolbetuximab.
[1] F=Female, M=Male; Race:W=White, B=Black/African American, P=Native Hawaiian or other Pacific islander, A=Asian, I=American Indian or Alaska native, O=Other.
[2] 'Before' for 'Onset before first dose of study drug', 'After' for 'Onset after first dose of study drug' and 'Late' for 'Onset 30 days after last dose of study drug'.

[3] AEs either were identified as serious by the Investigator, or upgraded by the sponsor based on review of the Sponsor’s list of Always Serious terms. Reason for seriousness: D = Death, RPH = Requires or prolongs hospitalization, CA = Congenital anomaly, LT = Life-threatening, PSDI = Persistent or significant disability/incapacity, OMI = Other medical importance, AST = upgraded according to Sponsor’s list of Always Serious terms

Generate for the following:

Program: </directory/directory/directory/program.sas> <Draft/Final Version>
Study 8951-CL-0302 Source: <Listing / Dataset>

Appendix 10.2.7.3
Adverse Events (MedDRA V23.0) Leading to Death
All Randomized Subjects

Program: </directory/directory/directory/program.sas> <Draft/Final Version>
Study 8951-CL-0302 Source: <Listing / Dataset>

Appendix 10.2.7.4
Serious Adverse Events (MedDRA V23.0)
All Randomized Subjects

.

Program: </directory/directory/directory/program.sas> <Draft/Final Version>
Study 8951-CL-0302 Source: <Listing / Dataset>

Appendix 10.2.7.7
Adverse Events (MedDRA V23.0) of Special Interest
All Randomized Subjects

Programming note: for special interest AEs please include the following (per SAP section 6.5.2) and add as footnote:
“AEs of special interest will include the following: Nausea or Vomiting or Abdominal Pain based on PT terms; Nausea or Vomiting based on PT terms; Hypersensitivity Reactions based on HS SMQ; infusion-Related Reactions (IRRs) by Investigators, Potential Infusion-Related Reactions (IRRs); Anemia; Neutropenia.”

Program: </directory/directory/directory/program.sas> <Draft/Final Version>
Study 8951-CL-0302 Appendix 10.2.8.1.1 Source: <Listing / Dataset>
Central and Local Laboratory Tests Results in SI Units - Hematology, Part x of y
All Randomized Subjects

Actual Arm	Subject ID	Age(years)/ Sex/ Race[1]/ Weight(kg)	Last Dose Day	Laboratory Type	Analysis Visit	Visit Day/Date&Time	Visit Name	Test 1 (Units)	Test 2 (Units)	Test 3 (Units)	Test 4 (Units)	...
Country = xxxx, Investigator Site = xxxxxx												
xxxx	xxxxxx	43/F/W/45	xxxx	Central	xxx	Xxx/YYYY-MM-DDThh:mm	xxx	xxx	xxxH	xxx	xxx	...
					xxx	Xxx/YYYY-MM-DDThh:mm	xxx	xxxL	xxx	xxx	xxx	
					xxx	Xxx/YYYY-MM-DDThh:mm	xxx	xxx	xxx	xxx	xxxH G4	
					...	...	...	...	...	...	...	
xxxx	xxxxxx	xxxx/	xxxx	Local	xxx	Xxx/YYYY-MM-DD	xxx	xxx	XxxH G3	xxx	xxx	...
		xxxx/			xxx	Xxx/YYYY-MM-DD	xxx	xxxL	xxx	xxx	xxx	
		xxxx/			xxx	Xxx/YYYY-MM-DD	xxx	xxx	xxx	xxx	xxxH	
		xxxx			...	...	...	...	...	...	...	

H: above laboratory reference range, L: below laboratory reference range. Refer to the listing in section 10.1.10.1/10.1.10.2 for the reference ranges for the central/local laboratory data. G1-G4 are based on CTCAE (version 4.03).
[1] F=Female, M=Male; Race:W=White, B=Black/African American, P=Native Hawaiian or other Pacific islander, A=Asian, I=American Indian or Alaska native, O=Other.

Program: </directory/directory/directory/program.sas> <Draft/Final Version>
Study 8951-CL-0302 Appendix 10.2.8.1.2 Source: <Listing / Dataset>
Central and Local Laboratory Tests Results in SI Units - Chemistry, Part x of y
All Randomized Subjects

Program: </directory/directory/directory/program.sas> <Draft/Final Version>
Study 8951-CL-0302 Appendix 10.2.8.1.3 Source: <Listing / Dataset>
Central and Local Laboratory Tests Results in SI Units - Urinalysis, Part x of y
All Randomized Subjects

Program: </directory/directory/directory/program.sas> <Draft/Final Version>
Study 8951-CL-0302 Appendix 10.2.8.1.4 Source: <Listing / Dataset>
Central and Local Laboratory Tests Results in SI Units - Coagulation, Part x of y
All Randomized Subjects

Program: </directory/directory/directory/program.sas> <Draft/Final Version>
Study 8951-CL-0302 Appendix 10.2.8.1.5 Source: <Listing / Dataset>

Central and Local Laboratory Tests Results in SI Units - Chemokine, Cytokine and Tryptase, Part x of y

Program: </directory/directory/directory/program.sas>
<Draft/Final Version>
Study 8951-CL-0302
Source: <Listing / Dataset>

Figure 9.3.1.1
Kaplan-Meier Plot of Progression-Free Survival, Independent Review
Full Analysis Set

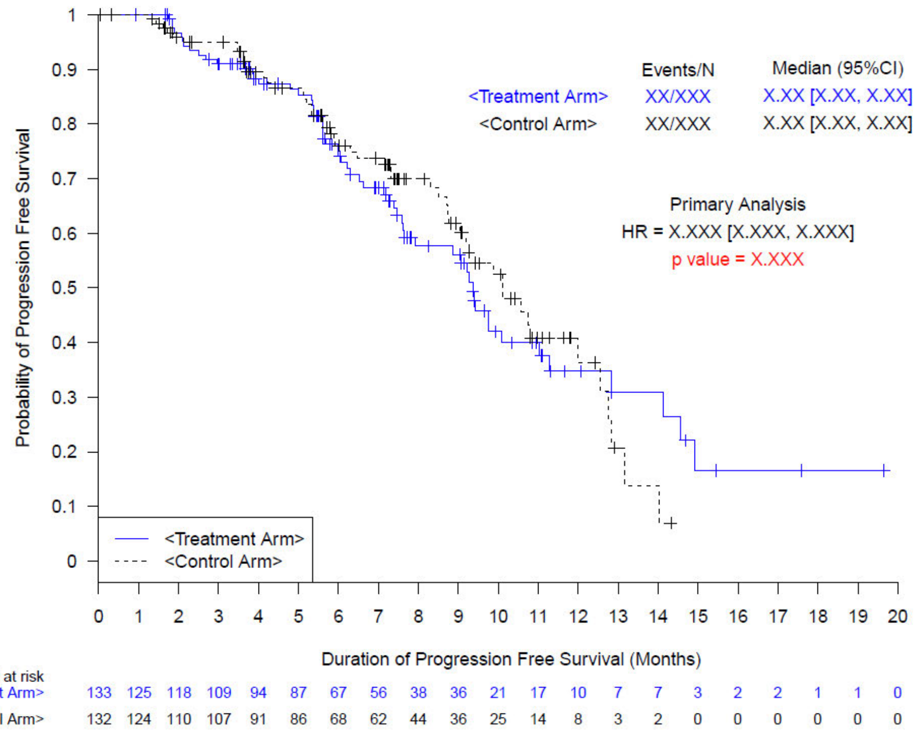

p value is generated from stratified log-rank test for the comparison of Arm A and Arm B

- This shell will be used for the following Figure:

Program: </directory/directory/directory/program.sas> <Draft/Final Version>
Study 8951-CL-0302 Figure 9.3.1.2 Source: <Listing / Dataset>
Kaplan-Meier Plot of Progression-Free Survival, Investigator Assessment
Full Analysis Set

Program: </directory/directory/directory/program.sas> <Draft/Final Version>
Study 8951-CL-0302 Figure 9.7.1 Source: <Listing / Dataset>
Kaplan-Meier Plot of Time to Cessation of Zolbetuximab/Placebo
Full Analysis Set

Program: </directory/directory/directory/program.sas> <Draft/Final Version>
Study 8951-CL-0302 Figure 9.7.2 Source: <Listing / Dataset>
Kaplan-Meier Plot of Time to Cessation of Oxaliplatin
Full Analysis Set

rogram: </directory/directory/directory/program.sas> <Draft/Final Version>
Study 8951-CL-0302 Figure 9.7.3 Source: <Listing / Dataset>
Kaplan-Meier Plot of Time to Cessation of Leucovorin
Full Analysis Set

rogram: </directory/directory/directory/program.sas> <Draft/Final Version>
Study 8951-CL-0302 Figure 9.7.4 Source: <Listing / Dataset>
Kaplan-Meier Plot of Time to Cessation of Fluorouracil Bolus
Full Analysis Set

Program: </directory/directory/directory/program.sas> <Draft/Final Version>
Study 8951-CL-0302 Figure 9.7.5 Source: <Listing / Dataset>
Kaplan-Meier Plot of Time to Cessation of Fluorouracil
Full Analysis Set

Program: </directory/directory/directory/program.sas> <Draft/Final Version>
Study 8951-CL-0302 Source: <Listing / Dataset>

Figure 9.3.2
Kaplan-Meier Plot of Overall Survival
Full Analysis Set

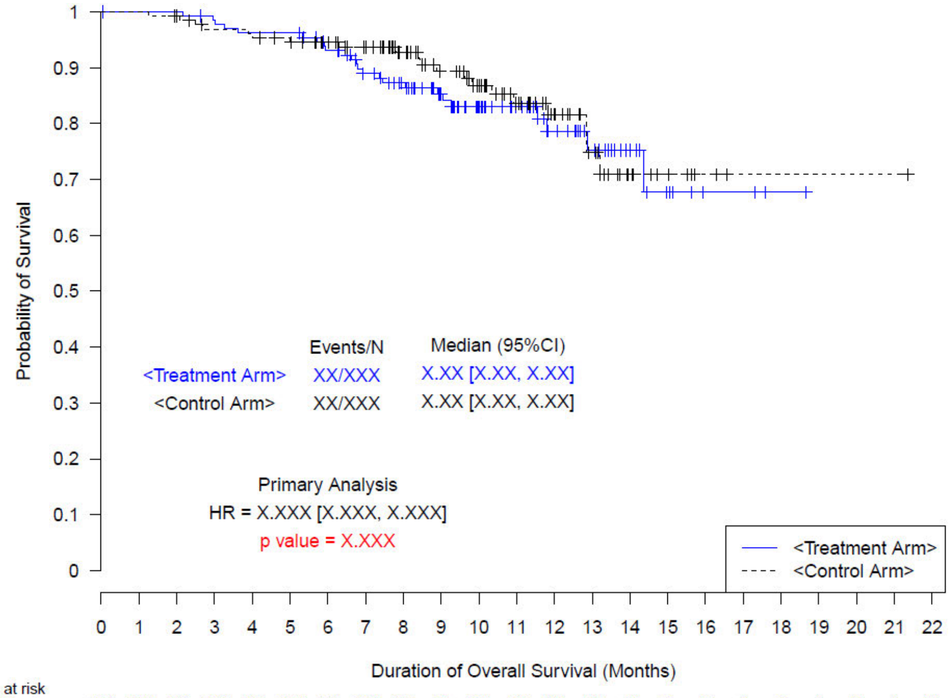

N at risk	0	1	2	3	4	5	6	7	8	9	10	11	12	13	14	15	16	17	18	19	20	21	22
<Treatment Arm>	133	132	132	129	126	126	118	107	95	78	59	47	32	22	12	7	3	3	1	0	0	0	0
<Control Arm>	132	132	130	124	123	119	113	105	92	76	63	51	36	21	11	7	3	1	1	1	1	1	0

p value is generated from stratified log-rank test for the comparison of Arm A and Arm B

Program: </directory/directory/directory/program.sas> <Draft/Final Version>
Study 8951-CL-0302 Figure 9.6.2.1 Source: <Listing / Dataset>
Spaghetti Plot of Laboratory Test Results in SI Units by Time - Biochemistry
Safety Analysis Set

-- < Laboratory Test = xxx, Treatment = Arm A > --

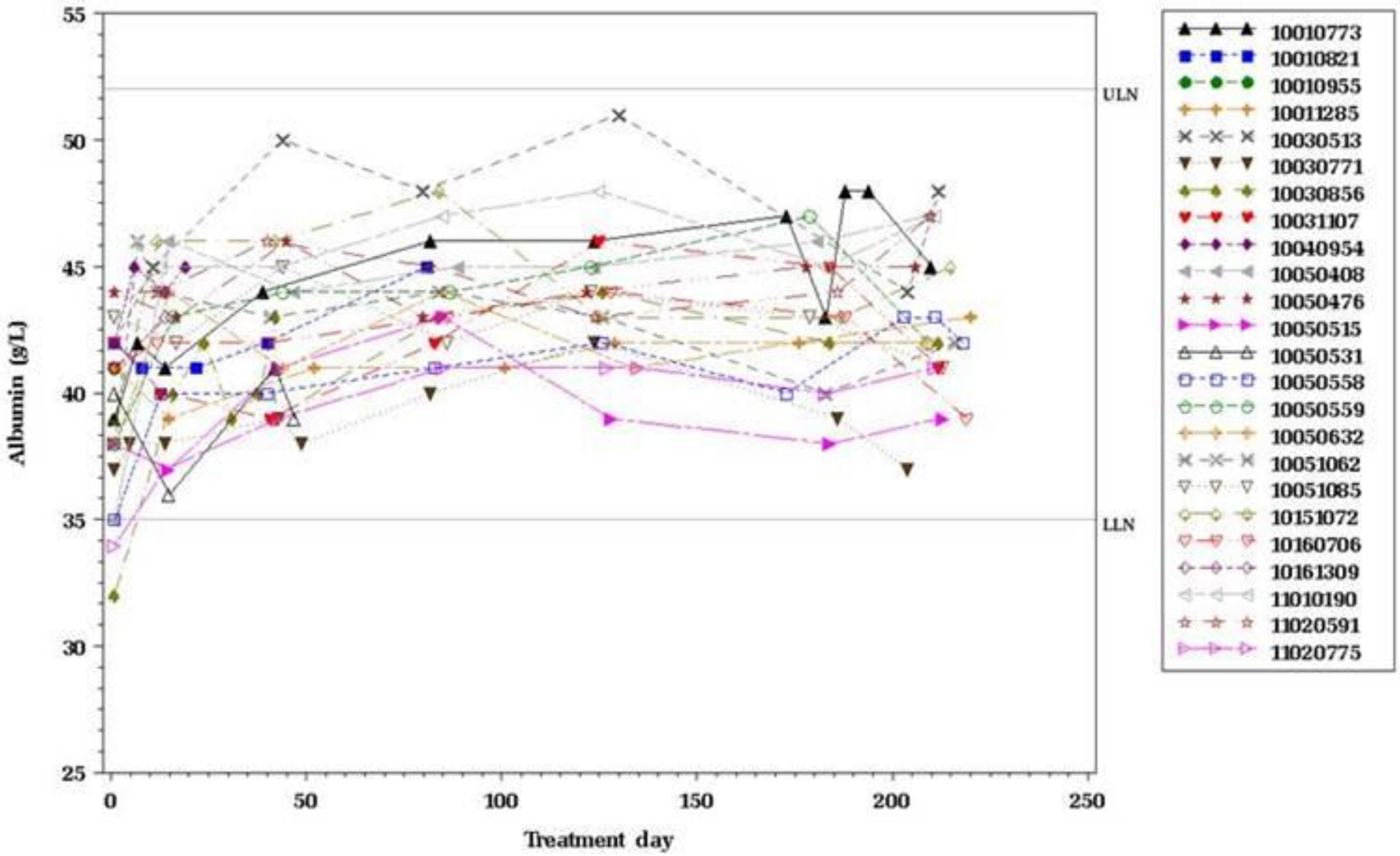

When shown, ULN is the maximum of all upper limit of normal range and LLN is the minimum of all lower limit of normal range.

- This shell will be used for the following figures:

Study 8951-CL-0302 Figure 9.6.2.2 Source: <Listing / Dataset>
Spaghetti Plot of Laboratory Test Results in SI Units by Time – Hematology
Safety Analysis Set

Study 8951-CL-0302 Figure 9.6.2.3 Source: <Listing / Dataset>
Spaghetti Plot of Laboratory Test Results in SI Units by Time – Urinalysis
Safety Analysis Set

Study 8951-CL-0302 Figure 9.6.2.4 Source: <Listing / Dataset>
Spaghetti Plot of Laboratory Test Results in SI Units by Time – Other
Safety Analysis Set

8.2 Author and Approver Signatories

E-signatures are attached at end of document (next page). Wet signatures, if any, are provided on this page.

Author: ______________________ Date: ______________

PPD

Approved by: ______________________ Date: ______________

PPD

Approved by: ______________________ Date: ______________

PPD
