# Supplementary material for: Zolbetuximab plus CAPOX in CLDN18.2-positive gastric or gastroesophageal junction adenocarcinoma: the randomized, phase 3 GLOW trial
Source: Nat Med. 2023 Jul 31;29(8):2133–41. doi: 10.1038/s41591-023-02465-7 (PMC10427418; doi:10.1038/s41591-023-02465-7)
Supplement: Supplementary file 2 — Reporting Summary [file 41591_2023_2465_MOESM2_ESM.pdf]

nature portfolio

Corresponding author(s): Prof. Rui-Hua Xu, M.D.

Last updated by author(s): Jun 14, 2023

Reporting Summary

Nature Portfolio wishes to improve the reproducibility of the work that we publish. This form provides structure for consistency and transparency in reporting. For further information on Nature Portfolio policies, see our Editorial Policies and the Editorial Policy Checklist.

Statistics

For all statistical analyses, confirm that the following items are present in the figure legend, table legend, main text, or Methods section.

n/a	Confirmed	
☐	☒	The exact sample size (*n*) for each experimental group/condition, given as a discrete number and unit of measurement
☒	☐	A statement on whether measurements were taken from distinct samples or whether the same sample was measured repeatedly
☐	☒	The statistical test(s) used AND whether they are one- or two-sided<br>*Only common tests should be described solely by name; describe more complex techniques in the Methods section.*
☒	☐	A description of all covariates tested
☐	☒	A description of any assumptions or corrections, such as tests of normality and adjustment for multiple comparisons
☐	☒	A full description of the statistical parameters including central tendency (e.g. means) or other basic estimates (e.g. regression coefficient) AND variation (e.g. standard deviation) or associated estimates of uncertainty (e.g. confidence intervals)
☐	☒	For null hypothesis testing, the test statistic (e.g. *F*, *t*, *r*) with confidence intervals, effect sizes, degrees of freedom and *P* value noted<br>*Give P values as exact values whenever suitable.*
☒	☐	For Bayesian analysis, information on the choice of priors and Markov chain Monte Carlo settings
☒	☐	For hierarchical and complex designs, identification of the appropriate level for tests and full reporting of outcomes
☒	☐	Estimates of effect sizes (e.g. Cohen's *d*, Pearson's *r*), indicating how they were calculated

Our web collection on statistics for biologists contains articles on many of the points above.

Software and code

Policy information about availability of computer code

Data collection	No specific software was used for data collection; the collected data were entered using the RAVE electronic data collection system.
Data analysis	Sample size calculations were performed with East® version 6.4. Statistical data analyses were performed with SAS® version 9.3 or higher.

For manuscripts utilizing custom algorithms or software that are central to the research but not yet described in published literature, software must be made available to editors and reviewers. We strongly encourage code deposition in a community repository (e.g. GitHub). See the Nature Portfolio guidelines for submitting code & software for further information.

Data

Policy information about availability of data

All manuscripts must include a data availability statement. This statement should provide the following information, where applicable:

- Accession codes, unique identifiers, or web links for publicly available datasets
- A description of any restrictions on data availability
- For clinical datasets or third party data, please ensure that the statement adheres to our policy

Upon request, and subject to certain criteria, conditions, and exceptions, Astellas will provide access to anonymized patient level data from completed Astellas sponsored phase 1 to 4 interventional clinical studies conducted for products and indications which have been approved in any country and also for studies conducted for terminated compounds. Approval must have been granted by the agencies of the main regions US, EU, and Japan. If approval is sought in only one or two regions, approval must have been granted by those agencies. Where available, the following anonymized patient level data and information is provided for

March 2021

each clinical study: raw dataset, analysis ready dataset, protocols with any amendments or addenda, annotated case report form, statistical analysis plan, dataset specifications, and clinical study report. Additionally, data may be available upon request. Researchers may request access to anonymized participant level data, trial level data and protocols from Astellas sponsored clinical trials at: www.clinicalstudydatarequest.com. For the Astellas criteria on data sharing see: https://clinicalstudydatarequest.com/Study-Sponsors/Study-Sponsors-Astellas.aspx. Patients remain on trial; for this reason, subject-level data are not available for this trial until completion.

Human research participants

Policy information about studies involving human research participants and Sex and Gender in Research.

Reporting on sex and gender	Sex was reported by study site staff through an interactive response technology system with options "male" or "female." The study enrolled 315 (62.1%) male patients (159 [62.6%] in the zolbetuximab plus CAPOX group and 156 [61.7%] in the placebo plus cAPOX group) and 192 (37.9%) female patients (95 [37.4%] in the zolbetuximab plus CAPOX group and 97 [38.3%] in the placebo plus CAPOX group). Subgroup analyses of PFS and OS by sex were pre-planned and were reported in Figures 2 and 3 of the manuscript.
Population characteristics	Population characteristics are provided in Table 1 of the manuscript.
Recruitment	Potential patients were identified by the investigators or staff at the institutions/centers participating in the study based on their diagnosis. Recruitment was limited to locations or regions where the study was being conducted.
Ethics oversight	The protocol and all amendments were approved by the appropriate ethics committee or institutional review board at each participating institution. Patients provided written informed consent before participating in the trial. All the authors attest that the trial was conducted in accordance with the Declaration of Helsinki and the standards of Good Clinical Practice.

Note that full information on the approval of the study protocol must also be provided in the manuscript.

Field-specific reporting

Please select the one below that is the best fit for your research. If you are not sure, read the appropriate sections before making your selection.

☒ Life sciences ☐ Behavioural & social sciences ☐ Ecological, evolutionary & environmental sciences

For a reference copy of the document with all sections, see nature.com/documents/nr-reporting-summary-flat.pdf

Life sciences study design

All studies must disclose on these points even when the disclosure is negative.

Sample size	The study aimed to enroll 500 patients. The final analysis of PFS was planned when 300 patients experienced disease progression or death to provide 93.4% power to detect a between-group difference with the assumption of median PFS of 9 months with zolbetuximab plus CAPOX versus 6 months with placebo plus CAPOX (HR = 0.67) at an overall one-sided alpha level of 0.025. An interim analysis of OS was planned at the final PFS analysis, and a final analysis of OS was planned after 386 deaths to provide 80% power to detect a between-group difference with the assumption of median OS of 14.7 months with zolbetuximab plus CAPOX versus 11 months with placebo plus CAPOX (HR = 0.75) at an overall one-sided alpha level of 0.025.
Data exclusions	No data were excluded up to the data cut-off date.
Replication	N/A
Randomization	Randomization was performed by blinded site staff using interactive response technology by block randomization with block sizes of two and was stratified according to region (Asia versus non-Asia), number of organs with metastases (0 to 2 versus ≥3), and prior gastrectomy (yes versus no).
Blinding	The randomization list and study drug blinding were maintained by the interactive response technology system. The sponsor, investigators, clinical staff, and patients remained blinded to treatment throughout the study. To maintain blinding, zolbetuximab and placebo, which were identical in appearance and form, were provided to investigators or designees by an unblinded pharmacist and administered in identical volumes, routes, and schedules.

Reporting for specific materials, systems and methods

We require information from authors about some types of materials, experimental systems and methods used in many studies. Here, indicate whether each material, system or method listed is relevant to your study. If you are not sure if a list item applies to your research, read the appropriate section before selecting a response.

Materials & experimental systems

n/a	Involved in the study
☒	☐ Antibodies
☒	☐ Eukaryotic cell lines
☒	☐ Palaeontology and archaeology
☒	☐ Animals and other organisms
☐	☒ Clinical data
☒	☐ Dual use research of concern

Methods

n/a	Involved in the study
☒	☐ ChIP-seq
☒	☐ Flow cytometry
☒	☐ MRI-based neuroimaging

Clinical data

Policy information about clinical studies

All manuscripts should comply with the ICMJE guidelines for publication of clinical research and a completed CONSORT checklist must be included with all submissions.

Clinical trial registration	The trial was registered at ClinicalTrials.gov (NCT03653507).
Study protocol	The study protocol, statistical analysis plan, and all amendments were provided as Supplementary Information with the manuscript.
Data collection	Tumor response was assessed by imaging at screening, every nine weeks for the first 54 weeks of treatment, and every 12 weeks thereafter until disease progression or start of another anticancer treatment. Survival was assessed at least every 12 weeks during follow-up. Patients completed health-related quality of life assessments, including the EuroQOL Five Dimensions Questionnaire (EQ-5D-5L), and the European Organization for Research and Treatment of Cancer QLQ-C30, QLQ-OG25 plus STO22, and Global Pain at screening, every three weeks during study treatment, at study treatment discontinuation, and 30 and 90 days following study treatment discontinuation. Adverse events, graded according to the National Cancer Institute Common Terminology Criteria for Adverse Events version 4.03, were evaluated throughout the trial and for 90 days following study treatment discontinuation. Data were collected at study sites where study treatment was administered. The recruitment period was from January 21, 2019 (first patient randomized) through February 18, 2022 (last patient randomized). The data cut-off was October 7, 2022.
Outcomes	The primary endpoint was PFS per RECIST version 1.1 as determined by an independent review committee (IRC). A key secondary endpoint was OS; additional secondary endpoints were ORR and DOR per RECIST version 1.1 as determined by an IRC, and safety and tolerability of zolbetuximab. Time to confirmed deterioration in scores for European Organization for Research and Treatment of Cancer global health status and quality of life, physical functioning, and abdominal pain and discomfort assessments as a key secondary endpoint was not reported in this manuscript due to the pending clinically meaningful threshold from the ongoing exit survey study per protocol. Additional secondary endpoints not reported in this manuscript were additional patient-reported outcomes, and pharmacokinetics and immunogenicity of zolbetuximab. Efficacy endpoints were assessed in the intent-to-treat population, which included all randomized patients. Safety was assessed in the safety analysis set, which included all patients who received at least one dose of any study drug.
